# Novel Small Molecule GLP-1R Agonists Based on 1*H*-Benzo[*d*]imidazole-5-Carboxylic Acid Scaffold

**DOI:** 10.3390/molecules31071129

**Published:** 2026-03-29

**Authors:** Elena V. Tolkacheva, Tagir L. Salakhov, Alexandr Yu. Saliev, Natalia D. Lebedeva, Alisa M. Krasnodubets, Eugene Y. Smirnov, Sergey A. Silonov, Konstantin V. Balakin, Vladimir V. Chernyshov, Roman A. Ivanov

**Affiliations:** 1 Medicinal Biotechnology Department, Sirius University of Science and Technology, Olimpiyskiy Ave. 1, Sirius 354340, Krasnodar Region, Russiavladimir.chernyshov2012@yandex.ru (V.V.C.); ivanov.ra@talantiuspeh.ru (R.A.I.); 2 Laboratory of Structural Dynamics, Stability and Folding of Proteins, Institute of Cytology, Russian Academy of Sciences, Tikhoretsky Ave. 4, St. Petersburg 194064, Russia; e.smirnov@incras.ru (E.Y.S.); silonovsa@incras.ru (S.A.S.); 3 Institute of Future Biophysics, Institutsky Lane, 9, Dolgoprudny 141700, Moscow Region, Russia

**Keywords:** T2DM, GLP-1RAs, small molecules, “next-in-class” agonists, 1*H*-benzo[*d*]imidazole-5-carboxylic acid derivatives, cAMP assay

## Abstract

Glucagon-like peptide-1 (GLP-1) is an incretin hormone secreted by intestinal endocrine L cells that activates the GLP-1 receptor (GLP-1R), leading to glucose-dependent insulin secretion and suppression of glucagon release. In recent years, GLP-1R agonists (GLP-1RAs) have become one of the leading therapeutic options for the treatment of type 2 diabetes mellitus; however, for a long time clinically approved GLP-1RAs were limited to peptide drugs unsuitable for oral administration. The discovery of the “first-in-class” small molecule agonist danuglipron in 2018 demonstrated the feasibility of orally available GLP-1RAs and stimulated the development of numerous danuglipron-like compounds, some of which showed increased efficacy over the prototype. In this study, we report the design and synthesis of novel GLP-1RAs based on a regioisomeric danuglipron scaffold, 1*H*-benzo[*d*]imidazole-5-carboxylic acid. A series of 35 compounds was synthesized and evaluated in vitro for cytotoxicity and GLP-1R agonistic activity using a cAMP accumulation assay. A potent lead compound **12r** (pEC_50_ = 7.72, pCC_50_ < 3.60) was found which is a close structural analog of danuglipron with reduced cytotoxicity and excellent selectivity over two other class B GPCRs, including GCGR and GIPR. Despite decreased potency compared to danuglipron, the obtained results hold promise for further optimization and provide valuable structure–activity relationship insights.

## 1. Introduction

Glucagon-like peptide-1 (GLP-1) is an incretin hormone secreted by intestinal endocrine L cells. It activates the glucagon-like peptide-1 receptor (GLP-1R), which, in turn, stimulates insulin release in a glucose-dependent manner and inhibits glucagon secretion. GLP-1 promotes satiety, reduces food intake, slows gastric emptying, and reduces human body weight [1,2,3,4,5]. Identification of GLP-1R as a valuable biological target and establishing its role in metabolic regulation significantly influenced type 2 diabetes (T2DM) and obesity treatment [6,7]. Glucagon-like peptide-1 receptor agonists (GLP-1RAs) have demonstrated superiority over previously used drugs targeting other targets in the treatment of T2DM (sulfonylureas [8], peroxisome proliferator-activated receptor-γ (PPARγ) agonists [9,10], α-glucosidase inhibitors [11], dipeptidyl peptidase-4 inhibitors (DPP-4i), and sodium glucose cotransporter 2 inhibitors (SGLT-2i)) [12,13,14,15,16]. They have also demonstrated the ability to exert cardio- and neuroprotective effects [17,18,19,20], reduce the risks of atherosclerosis and other cardiovascular diseases (CVD), and provide kidney protection [7,21]. For a long time, only peptide molecules were approved for clinical use, including exenatide (Eli Lilly), liraglutide (Novo Nordisk), dulaglutide (Eli Lilly), semaglutide (Novo Nordisk, the only drug for oral use since 2019), albiglutide (GlaxoSmithKline) and tirzepatide (Eli Lilly, a dual GIP/GLP-1 receptor agonist), which have a significant drawback—poor bioavailability in case of oral administration (<1% for semaglutide) [22,23,24]. The unmet medical need for effective oral drugs has led to the search for small molecule GLP-1RAs that combine high efficacy and safety with ease of use [25,26,27,28,29,30,31,32,33,34].

**Figure 1:**
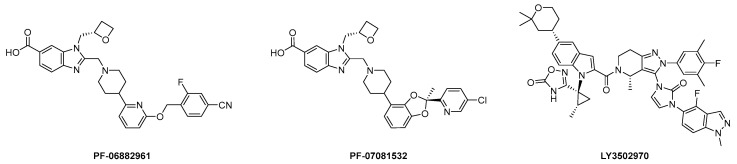
“First-in-class” small molecule GLP-1RAs: danuglipron (PF-06882961, [35,36]), lotiglipron (PF-07081532, [37]), orforglipron (LY3502970, [38,39]).

Pfizer and Eli Lilly were pioneers in the field of highly active small molecule GLP-1RAs. In 2018–2019, the former introduced two structurally similar “first-in-class” agonists, danuglipron (PF 06882961) [35,36] and lotiglipron (PF 07081532) [37], and the latter introduced orforglipron (LY3502970) [38,39] (Figure 1).

Lotiglipron was withdrawn from clinical trials in phase I studies due to dose-dependent increase in liver enzymes, indicating potential hepatotoxicity [40]. Danuglipron successfully completed Phase I trials and demonstrated dose-dependent, placebo-adjusted reductions in glycated hemoglobin (HbA1c), plasma glucose, and body weight in Phase II. However, a high incidence of gastrointestinal (GI) side effects, including nausea (73% of cases), vomiting (47% of cases), and diarrhea (25% of cases), led to the early withdrawal of 53% of volunteers from the clinical trials. Although the company attempted to optimize the dosing regimen of danuglipron to improve tolerability, its hepatotoxic properties identified during clinical trials in April 2025 terminated its development [41]. Thus, orforglipron became the first oral small molecule GLP-1RA drug to successfully complete phase III clinical trials in April 2025 [42].

**Figure 2:**
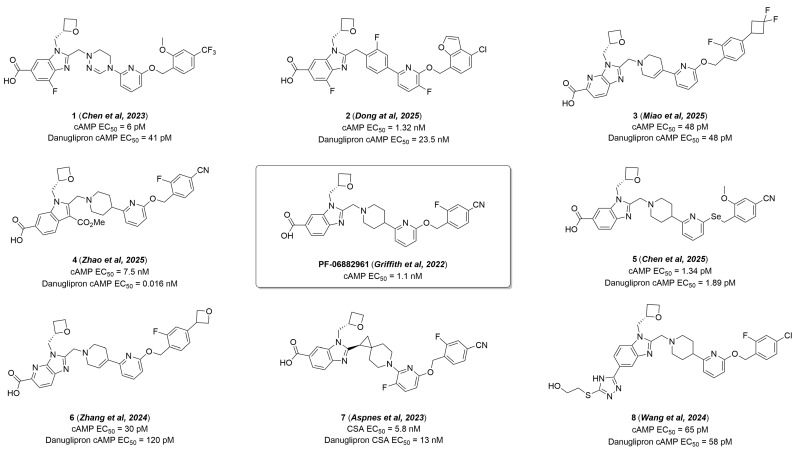
“Next-in-class” small molecules GLP-1RAs **1**–**8** structurally similar to danuglipron [35,44,45,46,47,48,49,50,51].

Since danuglipron and lotiglipron were disclosed as highly active small molecule GLP-1RAs in 2018–2019, a wide variety of “next-in-class” agonists have been reported in 2020–2025, as shown in our patent review [43] and some published papers [44,45,46,47,48,49,50,51]. The reported agonists can be divided into two groups: those structurally similar to “first-in-class” GLP-1RA, which were designed through evolutionary modifications of prototypes, and compounds obtained via significant transformations of the original scaffolds. According to structure–activity relationship (SAR) analysis, each of these groups yielded highly active GLP-1RAs with comparable or superior functional activity to their prototypes [43]. Figure 2 shows the structures of some reported “next-in-class” GLP-1RAs **1**–**8** (highly active lead compounds), structural analogs of danuglipron [44,45,46,47,48,49,50,51].

Based on cAMP assay data, most of the developed lead compounds **1**–**8** are superior to or equal to danuglipron in terms of EC_50_ values (Figure 2). The only exception is the 1*H*-indole derivative **4** [47], which, according to cAMP accumulation assay, is more than 450-fold less active than danuglipron, despite the presence in the structure of most of the key structural motifs inherent to the prototype (Figure 2). Despite a two-fold increase in the functional activity of compound **7** from Pfizer compared to danuglipron, high intrinsic clearance (Clint) combined with off-target PDE3A1 activity led to the discontinuation of its development (Figure 2) [50]. Compounds **3** [46], **5** [48], and **8** [51] are comparable in functional activity to the prototype (Figure 2). At the same time, the pharmacokinetic profile of compounds **3** and **5** has improved significantly compared to danuglipron; in particular, oral bioavailability has increased to 54% and 20%, respectively. The EC_50_ values for agonists **1** [44], **2** [45], and **6** [49] exceed those for danuglipron by 7, 18, and 4 times, respectively (Figure 2). The development of new 5,6-dihydro-1,2,4-triazine derivatives as GLP-1RAs has resulted in some improvement in the pharmacokinetic properties of the target compounds, highlighting the need for further effort in the search for the most promising drug candidate [44]. Meanwhile, the oral bioavailability of agonists **2** [45] and **6** [49] increased to 20% and 33%, respectively, and their pharmacokinetic profile was significantly improved compared to danuglipron, allowing these drug candidates to enter Phase I clinical trials. Available structure–activity data indicate that even minimal modifications of danuglipron can significantly improve the candidate’s pharmacokinetic properties while maintaining or improving their functional activity. In this regard, the development and synthesis of similar “next-in-class” GLP-1 receptor agonists is a highly pressing issue due to the discontinuation of danuglipron’s development.

Most of the reported potent GLP-1RAs contain a privileged 2-substituted 1-((*S*)-oxetan-2-ylmethyl)-1*H*-benzo[*d*]imidazole scaffold (or its aza analogs and fluoro-substituted derivatives) containing a carboxyl functional group, its bioisostere, or a heterocyclic substituent at the 6-position of the heterocycle. The number of described agonists with 5-substituents, mainly bioisosteric heteroaromatic analogs of the carboxyl group, is quite limited, although very promising compounds with activity in a subnanomolar range have been identified among them [51,52,53,54,55]. Therefore, it was reasonable to conduct further study of this underexplored area of chemical space by varying the 5-substituents and some other structural motifs typical of highly active compounds. The aim of this study was to design and synthesize new potent GLP-1RAs containing a 1*H*-benzo[*d*]imidazole-5-carboxylic acid scaffold or its heteroaromatic analogs and to identify key SAR trends in this structural series.

**Figure 3:**
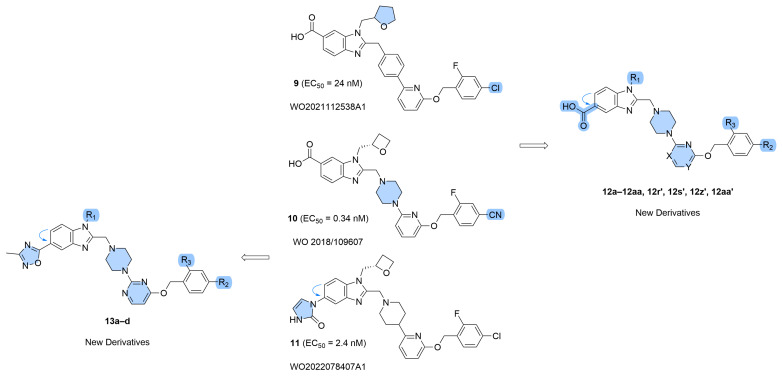
Design of the “next-in-class” target molecules of general formulas **12** and **13**. The blue arrows indicate a change in the position of the carboxyl group [36,54,56].

The design of the target compounds of general formula **12** was primarily aimed at introducing a carboxyl group into position 5 of the 1*H*-benzo[*d*]imidazole moiety, as a key modification of the prototype, for analysis of SAR trends. To avoid significant changes in the binding pattern of the resulting compounds to the biotarget, the remaining parts of the molecular scaffold were subjected only to evolutionary structural modifications relative to danuglipron structure. Specifically, we replaced the pyridine and piperidine moieties with pyrimidine and piperazine ones, respectively. As shown in our analysis of the patent literature [43], such modifications can lead to nanomolar agonists such as **10** [36] and **9** [56] (Figure 3). To simplify the analysis of SAR dependencies, we used a limited set of structural modifications of the terminal benzyl alcohol residue in this structural series.

Compounds **13a**–**d** represent another experimental series containing a heterocyclic 5-substituent in place of the carboxyl group (Figure 3). As previously shown, such compounds often exhibit nanomolar agonist activity [51,52,53,54,55]; one example is compound **11** [54] (Figure 3). The 3-methyl-1,2,4-oxadiazol-5-yl substituent was chosen due to its versatile pharmacological activity [57] and the absence of similar derivatives as GLP-1RAs in previously published studies. It should be noted that in this part of research our objectives were related not only to the discovery of active agonists but, first of all, to the development of effective synthetic approaches to key compounds. This explains the limited structural variability of compounds **13a**–**d**.

In addition to compounds of general formula **12**, GLP-1RAs **13a**–**d** containing a heterocyclic substituent in place of the carboxyl group were proposed (Figure 3). As previously shown, such compounds often exhibit nanomolar agonist activity [51,52,53,54,55]; one example is compound **11** [54] (Figure 3). The 3-methyl-1,2,4-oxadiazol-5-yl substituent was chosen due to its versatile pharmacological activity [57] and the absence of similar derivatives as GLP-1RAs in previously published studies.

## 2. Results and Discussion

### 2.1. Chemistry

**Figure 4:**
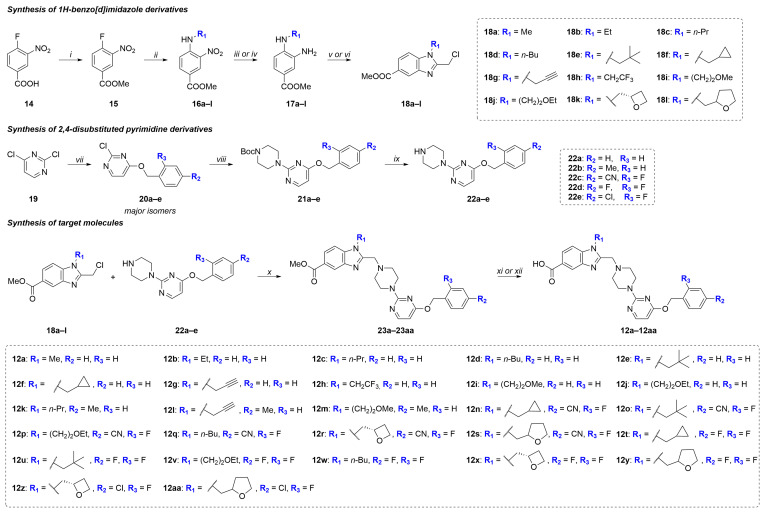
Synthetic route to intermediates **18a**–**l**, **22a**–**e**, and target molecules **12a**–**12aa**. Reagents and conditions: (*i*) MeOH, H_2_SO_4 (conc)_, reflux, 28 h, 95%; (*ii*) primary amine, DCM, DIPEA, N_2_, rt, 2 h, 90–98%; (*iii*) H_2_, Pd/C, EtOH, rt, 2 h, 85–98%; (*iv*) Fe, NH_4_Cl, THF:MeOH:H_2_O (6:3:1), reflux, 5 h, 85–90%; (*v*) (1) ClCH_2_COCl, DIPEA, THF, N_2_, rt, 1 h; (2) AcOH, 90 °C, 3 h, 40–75%; (*vi*) ClCH_2_CH(OMe)_3_, *p*TSA, AcN, 60 °C, 1 h, 55–75%; (*vii*) benzyl alcohol, *tert*-BuOK, THF, DMF, −50 °C, 3 h, summary yield 80–90%; (*viii*) N-Boc-piperazine, THF, reflux, 6 h, 75–85%; (*ix*) (1) TFA, DCM, reflux, 2 h; (2) Na_2_CO_3 (aq)_, 85–90%; (*x*) K_2_CO_3_, KI, AcN, rt, 24 h, 90–95%; (*xi*) (1) NaOH (1 M, H_2_O), MeOH, rt, 24 h; (2) aqueous citric acid (1 N), 85–90%; (*xii*) (1) TBD (0.97 M, H_2_O), AcN, rt, 24 h; (2) aqueous citric acid (1 N), 75–90%.

A total of 27 target 2-((4-(4-(benzyloxy)pyrimidin-2-yl)piperazin-1-yl)methyl)-1-substituted-1*H*-benzo[*d*]imidazole-5-carboxylic acids **12a**–**12aa** were obtained from the commercially available precursors **14** and **19** as shown on the scheme (Figure 4).

1*H*-Benzo[*d*]imidazole derivatives **18a**–**l** were prepared by the classical method [58,59] from commercially available 3-nitro-4-fluorobenzoic acid **10** in 4 steps (Figure 4). Esterification of acid **14** gave methyl ester **15** [60], which was then introduced into a S_N_Ar reaction with a series of primary amines according to a previously described method [61]. The resulting compounds **16a**–**l** were then reduced to *o*-phenylenediamine derivatives **17a**–**l** according to two reported protocols [62,63]. The target 1*H*-benzo[*d*]imidazole derivatives **18a**–**l** were prepared using two previously described protocols: acylation with chloroacetyl chloride [64] followed by heterocyclization under acidic conditions [65], and treatment with 2-chloro-1,1,1-trimethoxyethane in the presence of *p*TSA [35]. In the first case, we observed the formation of a by-product of intramolecular substitution of the chlorine atom in the acyl intermediates, and therefore the yield of the target 1*H*-benzo[*d*]imidazoles was lower compared to the second protocol.

2,4-Disubstituted pyrimidines **22a**–**e** were synthesized according to previously reported methods [66,67,68] from 2,4-dichloropyrimidine **19** in three steps: a regioselective, kinetically controlled reaction with a series of benzyl alcohols leading to compounds **20a**–**e**, then a reaction with *N*-Boc-piperazine leading to compounds **21a**–**e**, and removal of the Boc-protecting group in the final step (Figure 4).

The final steps of synthesis of target compounds **12a**–**12aa** included alkylation [69] of pyrimidine derivatives **22a**–**e** with benzo[*d*]imidazole derivatives **18a**–**l** to form esters **23a**–**23aa**, which were further hydrolyzed using NaOH solution (1 M in H_2_O), MeOH, or 1,5,7-triazabicyclo [4.4.0]dec-5-ene (TBD, 0.97 M in H_2_O) [35] in AcN solution (Figure 4).

In the first stage of our study, we prepared 1*H*-benzo[*d*]imidazole-5-carboxylic acid derivatives **12a**–**j** containing an unsubstituted benzyl alcohol residue (R_2_ = R_3_ = H) and various substituents at the nitrogen atom, including alkyl, cycloalkyl, fluorine-containing, and ether substituents (Figure 4). These compounds were obtained to refine the synthetic scheme and confirm the presence of functional activity in this chemotype, as well as to study the effect of substituents both in the benzyl alcohol and the 1*H*-benzo[*d*]imidazole fragments on the target activity. Then we focused on compounds **12k**–**m** containing the electron-donating methyl groups (R_2_ = Me, R_3_ = H) (EDG) and compounds **12n**–**12aa** containing electron-withdrawing groups (EWG) in the benzyl alcohol moiety (R_2_ = CN, R_3_ = F; R_2_ = R_3_ =F; R_2_ = Cl, R_3_ = F) (Figure 4). In the final step, R_1_ substituents in the 1*H*-benzo[*d*]imidazole scaffold, present in the structures of the most active molecules from the **9a**–**j** series, as well as privileged (*S*)-oxetan-2-ylmethyl and homologous (*rac*)-tetrahydrofuran-2-ylmethyl substituents, were introduced into the structures of the target compounds **9n**–**9aa** containing 2,4-EWG-disubstituted benzyl alcohol residues.

**Figure 5:**
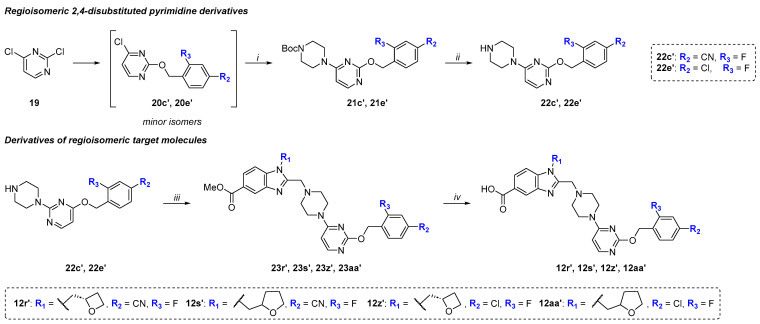
Synthetic route to intermediates **22c′**, **22e′**, target molecules **12r′**, **12s′**, **12z′**, **12aa′**. Reagents and conditions: (*i*) N-Boc-piperazine, THF, reflux, 6 h, 80–90%; (*ii*) (1) TFA, DCM, reflux, 2 h; (2) Na_2_CO_3 (aq)_, 85%; (*iii*) **18k**, **18l**, K_2_CO_3_, KI, AcN, rt, 24 h, 85–90%; (*iv*) (1) TBD (0.97 M, H_2_O), AcN, rt, 24 h; (2) aqueous citric acid (1 N), 80–90%.

At the stage of synthesis of compounds **21c** and **21e**, we isolated minor regioisomers **21c′** and **21e′** using column chromatography, which were used to obtain the target regioisomeric pyrimidine derivatives **22c′** and **22e′** (Figure 5). Using compounds **22c′**, **22e′** and 1*H*-benzo[*d*]imidazole derivatives **18k**, **18l**, the corresponding regioisomeric methyl esters **23r′**, **23s′**, **23z′**, **23aa′** and target acids **12r′**, **12s′**, **12z′**, **12aa′** were obtained according to the above-described protocol.

**Figure 6:**
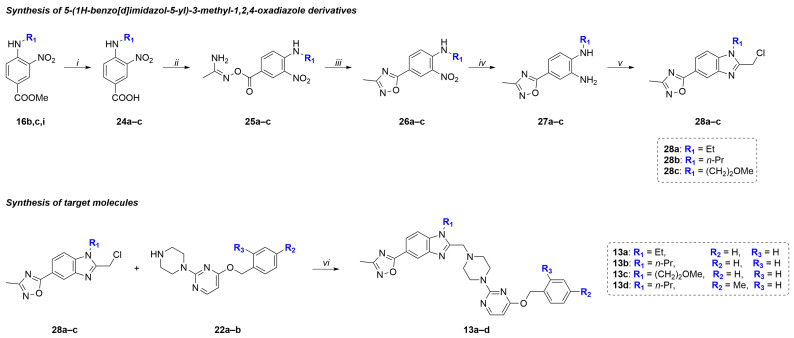
Synthetic route to intermediates **28a**–**c** and target molecules **13a**–**d**. Reagents and conditions: (*i*) (1) NaOH (1.5 M, H_2_O), THF, reflux, 2 h; (2) H_2_SO_4_ (10%, H_2_O), 90–95%; (*ii*) (1) CDI, DCM, rt, 1.5 h; (2) CH_3_C(=NOH)NH_2_, rt, 4–6 h, 80–85%; (*iii*) TBAF (1 M, THF), THF, reflux, 1 h, 90–95%; (*iv*) Fe, NH_4_Cl, THF:MeOH:H_2_O (6:3:1), reflux, 5 h, 85–90%; (*v*) (1) ClCH_2_COCl, DIPEA, THF, rt, 0.5 h; (2) AcOH, 90 °C, 3 h, 40–50%; (*vi*) K_2_CO_3_, KI, AcN, rt, 24 h, 70–75%.

The synthesis of target compounds **13a**–**d** began with the preparation of 5-(1*H*-benzo[*d*]imidazol-5-yl)-3-methyl-1,2,4-oxadiazole derivatives **28a**–**c** from the previously obtained compounds **16b**,**c**,**i** (Figure 6).

The synthesis of 1,2,4-oxadiazole derivatives is shown in Figure 6. First, esters **16b**,**c**,**i** were hydrolyzed to form benzoic acids **24a**–**c**, which were activated with *N*,*N*′-carbonyldiimidazole (CDI) and then reacted with acetoamidoxime. The resulting *O*-acylamidoximes **25a**–**c** were treated with tetrabutylammonium fluoride (TBAF) as described previously [70,71,72] to form 1,2,4-oxadiazole derivatives **26a**–**c**. The transformation of compounds **26a**–**c** into the target 5-(1*H*-benzo[*d*]imidazol-5-yl)-3-methyl-1,2,4-oxadiazole derivatives **28a**–**c** was carried out similarly to the scheme depicted in Figure 4. In the final step, compounds **28a**–**c** were used for the alkylation of 2,4-disubstituted pyrimidines **22a**–**b** to form the target compounds **13a**–**d**.

### 2.2. In Vitro Signaling Studies

GLP-1R belongs to the class B family of transmembrane G protein-coupled receptors (GPCRs). Upon activation by an agonist, the receptor competitively interacts with heterotrimeric G proteins, primarily Gαs, which in turn activates membrane-bound adenylyl cyclase (AC). This enhances the conversion of ATP to cAMP, which binds to the regulatory subunits of protein kinase A (PKA). PKA phosphorylates numerous proteins, including the transcription factor CREB, which is involved in the regulation of β-cell proliferation and survival [73]. Concurrently, cAMP activates EPAC (cAMP-regulated guanine nucleotide exchange factor), triggering other effector pathways that influence exocytosis, calcium signaling, and transcription [74]. Both PKA and EPAC mediate glucose-dependent insulin secretion via exocytosis in β-cells.

In the cell-based test system used in this study, expression of the firefly luciferase gene is regulated by CREB. Although an increase in cAMP levels and subsequent CREB activation can also be induced by other effectors, this reporter system remains highly informative for evaluating the functional activity of GLP-1R agonists. This is because the intensity of the luminescent signal, which is proportional to the amount of luciferase expressed, directly reflects the degree to which the tested compound activates the intracellular signaling cascade.

### 2.3. GLP-1R Agonist Activity and Cytotoxicity

All the synthesized target compounds, **12a**–**12aa**, **12r′**, **12s′**, **12z′**, **12aa′**, and **13a**–**d**, were further experimentally tested for cytotoxicity against a human embryonic kidney cell line (HEK293), and their GLP-1R activity in cAMP assay was measured and compared with danuglipron (entry 36, the negative logarithm of half-maximal effective concentration pEC_50_ 9.96, the negative logarithm of cytotoxic concentration pCC_50_ 3.94, Table 1).

Compounds **12a**–**12j**, containing an unsubstituted benzyl alcohol residue in the structure, were almost completely inactive and noncytotoxic (entries 1–10, Table 1). A noticeable increase in activity while maintaining moderate cytotoxicity was observed when comparing homologues **12g** and **12l**, **12i** and **12m** (positions 12–13). Compounds **12n**–**12aa**, containing 2,4-EWG-disubstituted benzyl alcohols, deserve special attention: almost all presented compounds exhibited GLP-1R activity in the submicromolar concentration range (entries 14–27). The presence of a 4-chloro-2-fluorobenzyl alcohol residue in the studied chemotype led to a nanomolar agonist **12z** and a submicromolar agonist **12aa**, but both compounds showed pCC_50_ values above 4.25, indicating their cytotoxicity (entries 26–27). The introduction of a 4-cyano-2-fluorobenzyl alcohol residue into the GLP-1RA structure was the most successful modification both in terms of activity and moderate cytotoxicity of the target compounds **12n**–**12s** (entries 14–19). The least successful were 2,4-difluorosubstituted benzyl derivatives (entries 20–25), both in terms of GLP-1R activity and cytotoxicity. Replacement of the (S)-oxetan-2-ylmethyl substituent with its linear ethoxyethyl analog significantly decreased activity (compounds **12v** and **12p**, entries 16, 22). Cyclopropylmethyl and butyl substitutions led to equipotent agonists **12n** and **12q**, **12t** and **12w** (entries 14, 17, 20, 23), while neopentyl derivatives **12o** and **12u** (entries 15, 21) had a decreased agonistic activity, probably due to the increased branching.

As expected, the use of the privileged (*S*)-oxetan-2-ylmethyl and homologous (*rac*)-tetrahydrofuran-2-ylmethyl substituents as the R_1_-group resulted in the most potent agonists **12r**, **12s**, **12x**, **12z**, **12aa**. The most preferred, both in terms of activity and safety, was the (*S*)-oxetan-2-ylmethyl moiety. The target activities of the regioisomeric compounds did not differ in the case of structures **12s′** and **12aa′** with (*rac*)-tetrahydrofuran-2-ylmethyl substituents (entries 29, 31) but differed more than 10-fold in the case of agonists **12r** and **12z** substituted with (*S*)-oxetan-2-ylmethyl group (entries 28, 30).

The replacement of the carboxyl group with a 1,2,4-oxadiazole fragment in compounds **13a**–**d** resulted in submicromolar agonistic activity of GLP-1R, but with pronounced cytotoxic effects (entries 32–35).

**Figure 7:**
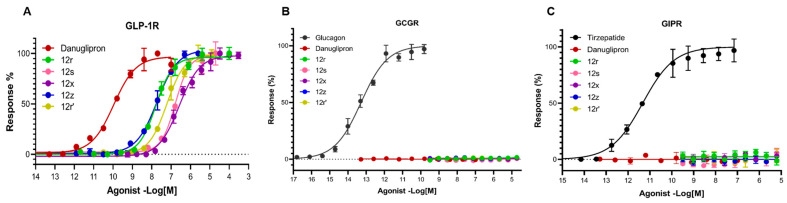
Dose–response curves of compounds **12r**, **12s**, **12x**, **12z**, **12r′** based on cAMP accumulation in HEK293T cells expressing GLP-1R (**A**), GCGR (**B**), and GIPR (**C**). Compounds **12r**, **12s**, **12x**, **12z**, **12r′** do not activate other class B1 GPCRs, and are selective GLP-1R agonists (**A**).

Based on the experimental results, five most interesting molecules can be identified among the tested compounds. The most active nanomolar agonist is compound **12r**; being the closest analog of danuglipron, it is inferior in activity but less cytotoxic. Secondly, nanomolar cytotoxic agonist **12z**, as well as three submicromolar agonists **12s**, **12x**, **12r′** with moderate to low cytotoxicity, deserve attention. The dose–effect curves of danuglipron and the mentioned agonists, obtained in our cAMP assay, are presented in Figure 7.

Compounds **12r**, **12s**, **12x**, **12z**, **12r′** were further evaluated for selectivity over other class B GPCRs, including GCGR and GIPR. No response to the tested compounds was detected at these receptors, while the native ligands of these receptors can effectively activate them, indicating the excellent selectivity of our agonists to GLP-1R (Figure 7). However, the results of cAMP assay showed that even the most active compounds are inferior in activity to danuglipron. This is likely due to the translocation of the carboxyl group from position 6 to position 5 of the 1*H*-benzo[*d*]imidazole scaffold.

## 3. Materials and Methods

### 3.1. Chemistry

All the experimental details, including procedures of synthesis of all the compounds described in the work, full physicochemical data for all compounds synthesized, as well as copies of their NMR and HRMS spectra are presented in the Supplementary Materials.

### 3.2. Plasmid Vectors

The firefly luciferase gene (Addgene plasmid #72684) was integrated into the pBabe plasmid vector (Addgene plasmid #15682) along with two cAMP-dependent CRE elements and a minimal CMV promoter (miniCMV) assembled from oligonucleotides using overlap PCR. The inserts were cloned using the SLIC method with overlapping primers and T4 polymerase (SE-E339 SibEnzyme, Novosibirsk, Russia). The resulting construct was named pBabe-CRE_Luc. The sequence of the human GLP-1R gene was amplified from cDNA of a human insulinoma provided by the FSBI “I.I. Dedov National Medical Research Center of Endocrinology” of the Ministry of Health of the Russian Federation. The plasmid construct for receptor expression was generated based on the pgRNA-humanized vector (Addgene plasmid #44248), from which the fragment containing the U6 promoter and the gRNA scaffold was removed. Using the restriction-ligation method, the vector was digested at the XhoI isoschizomer-Sfr274I (SE-E125 SibEnzyme, Novosibirsk, Russia) and PsiI (SE-E279 SibEnzyme, Novosibirsk, Russia) sites. Subsequently, the sticky end of Sfr274I was blunted using T4 polymerase, destroying the site, and ligated with T4 ligase (SE-E329 SibEnzyme, Russia). The human GLP-1R sequence was fused to GFP via a T2A peptide sequence using overlap PCR. The 5′ primer contained a NheI restriction site, the 3′ T2A primer contained an XhoI restriction site, as did the 5′ primer for GFP; the 3′ primer for GFP contained an EcoRI restriction site. The vector was digested using AsuNHI (SE-E063 SibEnzyme, Novosibirsk, Russia), which is an isoschizomer of NheI, and EcoRI (SE-E057 SibEnzyme, Novosibirsk, Russia). The amplified GLP1R-T2A fragment was digested using AsuNHI and Sfr274I. The amplified GFP gene was digested at the Sfr274I and EcoRI sites. The three resulting fragments were ligated using T4 ligase, and the constructs were transformed into NEB Stable Competent *E. coli* (High Efficiency) (C3040H, New England Biolabs, Ipswich, MA, USA). After colony screening and verification by Sanger sequencing, the resulting plasmid vector was used for lentiviral vector production. This vector was named pHumanized_GLP1R-T2A-GFP.

### 3.3. Transduction of HEK293T Cells

At all stages, cells were cultured in a DMEM medium (PanEco, Moscow, Russia) containing 4.5 g/L glucose, 2 mM L-glutamine (PanEco, Moscow, Russia), and 10% FBS (Cytiva (HyClone), Wilmington, DE, USA), under conditions of 37 °C and 5% CO_2_. In the first stage, a clone of 293T cell line from ATCC, transduced with retroviral vectors containing the firefly luciferase gene under the control of a minimal CMV promoter and two CRE elements, was generated. Viral vectors were obtained by transfecting the pBabe-CRE_Luc plasmid into the Phoenix helper-free retrovirus producer line [75] using the transfection reagent PEI MAX (24765, PolySciences, Warrington, PA, USA) according to the manufacturer’s protocol. For transduction, the supernatant from transfected cells collected at 24 and 48 h was pooled and filtered through a 0.45 µm syringe filter (PTF405013, Jet Biofil, Guangzhou, China). For transduction, 293T cells were seeded onto a 6 cm dish for adherent cultures (TCD010060, Jet Biofil, Guangzhou, China). Upon reaching 60% confluency, half of the medium was replaced with supernatant containing viral particles. To enhance transduction efficiency, 0.8 µg/mL of polybrene (TR 1003-50UL, Sigma-Aldrich, Moscow, Russia) was also added to the cells. Cells were incubated during transduction under standard conditions at 37 °C and 5% CO_2_ for 24 h, after which the cells were cultured in a fresh culture medium with 0.5 µg/mL puromycin (A1113803, Gibco™, Waltham, MA, USA) for selection for 7 d. Cloning was performed by limiting dilution; cells were seeded into 96-well plates (CAP011096, Jet Biofil, Guangzhou, China) at a density of 0.5 cells/well in 100 µL of a complete growth medium. The resulting clonal cell cultures were cultured in the presence of 0.3 µg/mL puromycin and analyzed for the functionality of the luciferase reporter using forskolin (TC472-25MG, HiMedia, Maharashtra, India), which promotes an increase in intracellular cAMP levels. A cell line whose luciferase response remained stable over a month of cultivation, with a luminescence signal-to-background ratio greater than 2, was selected for further work. These cells were named HEK CRE Luc. In the next stage, lentiviral vectors were produced in 293T cells. The transfer plasmid pHumanized_GLP1R-T2A-GFP and helper plasmids pMDLg/pRRE (Addgene plasmid #12251), pRSV-Rev (Addgene plasmid #12253), and pVSV-G (Addgene plasmid #138479) were used at a ratio of 10:5:3:1, respectively. The total amount of DNA was 10 µg per 6 cm diameter dish with cells at 70% confluency. The transfection reagent PEI MAX (24765, PolySciences, Warrington, PA, USA) was used at a 3:1 ratio to the total DNA mass. The HEK CRE Luc cells obtained earlier were transduced with the lentiviral vectors. For this, the supernatant from transfected cells collected at 24 and 48 h was pooled and filtered through a 0.45 µm syringe filter (PTF405013, Jet Biofil, Guangzhou, China). For transduction, HEK CRE Luc cells were seeded onto a 12-well plate for adherent cultures (TCP011012 Jet Biofil, Guangzhou, China). Upon reaching 60% confluency, 10, 25, 50, or 125 µL of supernatant with lentiviral vectors was added to the plate wells, bringing the volume of the culture medium in the well to 500 µL. Then, 0.4 µg of polybrene was added per well, and the plates were centrifuged for 1 h at 30 °C and 1000 g. After centrifugation, the plates were incubated under standard conditions at 37 °C and 5% CO_2_, with the culture medium replaced with a fresh medium after 24 h. After 48 h, the percentage of GFP-positive cells was assessed. Wells containing approximately 30–40% GFP-positive cells were analyzed by flow cytometry, and single cells were sorted into individual wells of a 96-well U-bottom plate (CAP012096, Jet Biofil, Guangzhou, China) using a BD FACSAria III (Becton Dickinson, Franklin Lakes, NJ, USA). From the obtained clones, the most suitable one for analyzing the activity of GLP-1R agonists activating the cAMP-dependent signaling pathway was selected. For this, the relative level of GLP-1R expression was determined, the presence of target inserts in the genome was confirmed by qualitative PCR, the functionality and specificity of the system were confirmed using GLP-1 and GIP agonists, and stability over 20 passages was confirmed. The resulting cell line was named HEK CRE Luc GLP-1R-GFP.

### 3.4. Analysis of GLP-1R Agonist Activity

HEK CRE Luc GLP-1R-GFP cells were collected, resuspended, and adjusted to a density of 0.5 × 10^6^ cells/mL in a complete DMEM medium (PanEco, Moscow, Russia) containing 4.5 g/L glucose, 2 mM L-glutamine, and 10% FBS (Cytiva(HyClone), Wilmington, DE, USA). Cells were seeded into a black opaque 96-well plate (30296, SPL Lifesciences, Gyeonggi-do, Korea) at 100 µL per well and incubated overnight in a CO_2_ incubator (37 °C, 5% CO_2_). The test compounds and the reference drug danuglipron (PF-06882961, Pfizer, New York, NY, USA) were dissolved in DMSO (131954.1611, PanReac Applichem, Barcelona, Spain). Immediately prior to the assay, compound solutions at a concentration of 1–3 mM were prepared in a complete DMEM medium, followed by serial dilutions in a complete DMEM. Then, 50 µL of the diluted compound was transferred to the plate wells with cells. After 20 h of incubation, 70 µL of the medium was removed from each well. Then, 40 µL of D-luciferin solution in lysis buffer was added to the remaining 80 µL using the AbiLux Firefly Luciferase Assay Kit (LUX-011-3-100ML, Abisense, Sirius, Russia). The plate was incubated on a shaker (PST-60HL-4, BIOSAN, Riga, Latvia) at 500 rpm for 5 min at room temperature, after which chemiluminescence was measured using a microplate reader (BMG LABTECH CLARIOstar Plus, Ortenberg, Germany). Each experiment included three technical and three biological replicates. pEC_50_ and 95% CI EC_50_ values were determined by fitting dose–response curves using a four-parameter equation in the GraphPad Prism 8.2.1 software (San Diego, CA, USA).

### 3.5. Analysis of Specific Agonist Activity

To confirm the specific activity of the tested small molecules, biological activity assays against GIPR and GCGR were performed. Reporter cells expressing GIPR or GCGR were generated analogously to the GLP-1R cell line, using HEK CRE Luc cells. Receptor genes were cloned from the following plasmid vectors: GIPR-Tango (Addgene plasmid #66294) and GCGR-Tango (Addgene plasmid #66291), both gifts from Bryan Roth. For the GIPR-expressing cell line, Tirzepatide (LY3298176, Eli Lilly, Indianapolis, IN, USA) was used as a positive control, as it functions as a dual GLP-1R/GIPR agonist. For the GCGR-expressing cell line, Glucagon (H04AA01, Novo Nordisk, Bagsværd, Denmark) was used as the agonist control. Test compounds were prepared from DMSO stock solutions as described in Section 3.4. The concentration range for testing was selected based on the compounds’ known activity against GLP-1R. A series of 3-fold dilutions was prepared, starting from the maximum effective concentration. The assay procedure followed the same protocol described for GLP-1R agonist activity testing (Section 3.4). Each assay was performed in three independent experiments, each with three technical replicates. Dose–response curves were plotted and analyzed using GraphPad Prism 8.2.1 software (San Diego, CA, USA).

### 3.6. Cytotoxicity Assay

The HEK293 cell line was kindly provided by the cell culture collection of the Institute of Cytology RAS. The day before the assay, cells were seeded at a density of 1 × 10^4^ cells per well of 96-well microplates (Wuxi NEST Biotechnology Co., Wuxi, China). The cells were incubated overnight at 37 °C with 5% CO_2_ in a humidified incubator. Various concentrations of test compounds were prepared in a complete culture medium (DMEM containing 10% FBS, 2 mM L-glutamine, and 50 μg/mL gentamicin; all from Biolot, Moscow, Russia) and added to the wells. After 48 h incubation, the medium was replaced with 200 μL of a fresh culture medium containing 0.5 mg/mL MTT (PanEko, Moscow, Russia) and plates were incubated for 2 h to allow formazan crystal formation. Subsequently, the medium was removed, and formazan crystals were dissolved in 150 μL of DMSO (Helicon, Moscow, Russia). Absorbance was measured at 595 nm using an iMark microplate absorbance reader (Bio-Rad, Hercules, CA, USA). Each experiment included three technical replicates per plate. Statistical analysis and curve fitting were performed using GraphPad Prism 7 software (GraphPad Software, San Diego, CA, USA). pCC_50_ and SEM_pCC50_ values were calculated using a four-parameter logistic equation.

## 4. Conclusions

In conclusion, in this work we attempted to look into the underexplored chemical space of 5-substituted regioisomeric analogs of danuglipron-like GLP-1R agonists. A series of novel “next-in-class” GLP-1RAs structurally similar to danuglipron were synthesized and evaluated in vitro for cytotoxicity and GLP-1R agonistic activity using a cAMP accumulation assay. Translocation of the carboxyl group from position 6 to position 5 of the 1*H*-benzo[*d*]imidazole core resulted in an overall decrease in agonistic activity compared to danuglipron, although in several cases this modification was accompanied by a decrease in cytotoxicity. More than half of the synthesized compounds demonstrated lower cytotoxicity than the prototype, with twelve compounds showing pCC_50_ values lower than 3.60. We have found that the position of the 2,4-disubstituted pyrimidine linker significantly influenced cytotoxicity, with compounds containing a 2-benzyloxypyrimidin-4-yl moiety exhibiting lower cytotoxicity than their 4-benzyloxypyrimidin-2-yl regioisomers.

Compound **12r** was identified as a nanomolar GLP-1R agonist (pEC_50_ = 7.80) with low cytotoxicity (pCC_50_ < 3.60) and excellent selectivity over two other class B GPCRs, including GCGR and GIPR. Thus, unlike dual agonists (e.g., Tirzepatide) or triple agonists, compound **12r** does not target GIPR or GCGR, making it a “pure” GLP-1R agent. Several additional nanomolar analogs were obtained which can be considered as a starting point for further optimization. This work also reports, for the first time in this chemotype, the replacement of the carboxyl group with a 3-methyl-1,2,4-oxadiazole-5-yl substituent. Collectively, these findings provide useful chemical and biological information that can guide the rational optimization of compounds within this chemotype and address current limitations in the field.

## Figures and Tables

**Table 1:** Structure, cAMP Activity, and Cytotoxicity of Compounds **12a**–**12aa**, **12r′**, **12s′**, **12z′**, **12aa′**, **13a**–**d** in experiments in vitro.

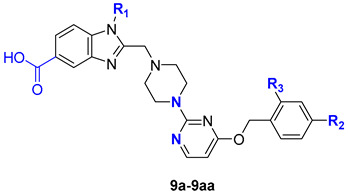								
**Entry**	**Cmpd**	**R_1_**	**R_2_**	**R_3_**	**pEC_50_ ^[a]^**	**95% CI ^[b]^**	**pCC_50_ ^[c]^**	**SEM** **pCC_50_ ^[d]^**
1	**12a**	Me	H	H	<5.00	ND ^[e]^	<3.60	ND
2	**12b**	Et	H	H	<5.00	ND	<3.60	ND
3	**12c**	*n*-Pr	H	H	<5.00	ND	3.91	3.73–4.09
4	**12d**	*n*-Bu	H	H	5.09	5.01–5.19	<3.60	ND
5	**12e**	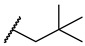	H	H	5.00	ND	4.12	3.86–4.38
6	**12f**	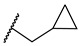	H	H	<5.00	ND	4.11	3.91–4.30
7	**12g**	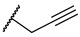	H	H	<5.00	ND	<3.60	ND
8	**12h**	CH_2_CF_3_	H	H	<5.00	ND	4.05	3.92–4.18
9	**12i**	(CH_2_)_2_OMe	H	H	<5.00	ND	<3.60	ND
10	**12j**	(CH_2_)_2_OEt	H	H	<5.00	ND	<3.60	ND
11	**12k**	*n*-Pr	Me	H	<5.00	ND	4.07	3.94–4.19
12	**12l**	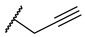	Me	H	5.76	5.60–5.79	3.85	3.50–4.21
13	**12m**	(CH_2_)_2_OMe	Me	H	5.43	5.30–5.57	<3.60	ND
14	**12n**	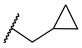	CN	F	5.98	5.86–6.08	4.09	3.95–4.22
15	**12o**	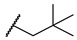	CN	F	5.42	5.32–5.504	<3.60	ND
16	**12p**	(CH_2_)_2_OEt	CN	F	6.08	6.00–6.16	3.90	3.53–4.26
17	**12q**	*n*-Bu	CN	F	6.38	6.28–6.5	4.07	3.50–4.64
18	**12r**	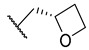	CN	F	**7.72**	**7.68–7.80**	**<3.60**	**ND**
19	**12s**	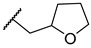	CN	F	**6.64**	**6.52–6.75**	**3.85**	**3.75–3.95**
20	**12t**	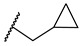	F	F	5.39	5.35–5.44	4.14	3.91–4.36
21	**12u**	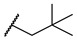	F	F	<5.00	ND	4.32	4.17–4.47
22	**12v**	(CH_2_)_2_OEt	F	F	5.18	5.13–5.24	4.39	4.02–4.76
23	**12w**	*n*-Bu	F	F	5.41	5.37–5.46	4.29	4.10–4.48
24	**12x**	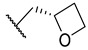	F	F	**6.57**	**6.47–6.66**	**3.87**	**3.48–4.27**
25	**12y**	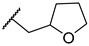	F	F	5.14	5.03–5.25	4.17	3.87–4.47
26	**12z**	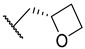	Cl	F	**7.80**	**7.60–7.96**	**4.40**	**4.27–4.53**
27	**12aa**	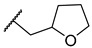	Cl	F	6.41	6.35–6.52	4.31	4.25–4.38
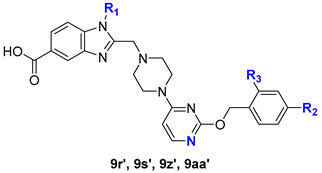								
**Entry**	**Cmpd**	**R_1_**	**R_2_**	**R_3_**	**pEC_50_** **^[a]^**	**95% CI** **^[b]^**	**pCC_50_** **^[c]^**	**SEM** **pCC_50_ ^[d]^**
28	**12r′**	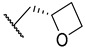	CN	F	**6.85**	**6.64–7.10**	**<3.60**	**ND** ^[e]^
29	**12s′**	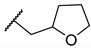	CN	F	6.51	6.23–6.70	<3.60	ND
30	**12z′**	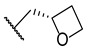	Cl	F	5.00	ND	<3.60	ND
31	**12aa′**	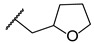	Cl	F	6.49	6.39–6.60	3.74	3.51–3.97
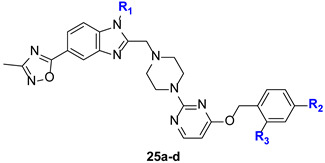								
**Entry**	**Cmpd**	**R_1_**	**R_2_**	**R_3_**	**pEC_50_** **^[a]^**	**95% CI** **^[b]^**	**pCC_50_** **^[c]^**	**SEM** **pCC_50_ ^[d]^**
32	**13a**	Et	H	H	7.00	6.72–7.30	4.80	4.63–4.97
33	**13b**	*n*-Pr	H	H	6.17	5.91–6.43	5.00	4.86–5.14
34	**13c**	(CH_2_)_2_OMe	H	H	5.44	5.35–5.47	5.06	4.92–5.21
35	**13d**	*n*-Pr	Me	H	5.30	5.18–5.50	4.34	4.08–4.60
36	danuglipron				**9.96**	**9.89–10.05**	**3.94**	**3.63–4.25**

^[a]^ A negative logarithm of half-maximal effective concentration; the concentration of a substance that gives 50% response of a biological pathway; pEC_50_ values represent the means of three independent experiments (R squared > 0.95). ^[b]^ 95% Confidence Interval pEC_50_; a range of values calculated from a sample that is likely to contain the true population value 95% of the time. ^[c]^ A negative logarithm of 50% cytotoxic concentration; the concentration resulting in 50% death of cells; pCC_50_ values represent the means of three independent experiments (R squared > 0.95). ^[d]^ Standard error of the mean. ^[e]^ Not Determined. The most active compounds and the prototype are highlighted in bold font.

## Data Availability

Data are contained within the article and Supplementary Materials.

## Supplementary Materials

The following supporting information can be downloaded at: https://www.mdpi.com/article/10.3390/molecules31071129/s1; Experimental details, including procedures of synthesis of all the compounds described in the work, full physicochemical data for all compounds synthesized, as well as copies of their NMR and HRMS spectra.

## Abbreviations

The following abbreviations are used in this manuscript:

GLP-1	Glucagon-like peptide-1
GLP-1RA	Glucagon-like peptide-1 receptor agonist
GCGR	G-protein-coupled receptor
GIPR	Gastric inhibitory polypeptide receptor
GPCRs	Transmembrane G protein-coupled receptors
AC	Adenylyl cyclase
PKA	Protein kinase A
cAMP	Cyclic adenosine monophosphate
EPAC	Exchange Protein directly Activated by cAMP
pEC_50_	A negative logarithm of half-maximal effective concentration
pCC_50_	A negative logarithm of 50% Cytotoxic concentration
SAR	Structure-activity relationship
