# Supplementary material for: Novel Small Molecule GLP-1R Agonists Based on 1H-Benzo[d]imidazole-5-Carboxylic Acid Scaffold
Source: Molecules. 2026 Mar 29;31(7):1129. doi: 10.3390/molecules31071129 (PMC13075017; doi:10.3390/molecules31071129)
Supplement: Supplementary file 1 [file molecules-31-01129-s001.zip › molecules-4188304-supplementary.pdf]

Supporting Information

Novel Small Molecule GLP-1R Agonists Based on 1*H*-Benzo[*d*]imidazole-5-Carboxylic Acid Scaffold

Elena V. Tolkacheva, Tagir L. Salakhov, Alexandr Yu. Saliev, Natalia D. Lebedeva, Alisa M. Krasnodubets, Eugene Y. Smirnov, Sergey A. Silonov, Konstantin V. Balakin, Vladimir V. Chernyshov, Roman A. Ivanov

Table of Contents

General 3

Synthetic Procedures and NMR spectra copies 4

Methyl 4-fluoro-3-nitrobenzoate **15** 4

4-Substituted methyl 3-nitrobenzoates **16a-l** 7

4-Substituted methyl 3-aminobenzoates **17a-l** 11

1-Substituted methyl 2-(chloromethyl)-1*H*-benzo[*d*]imidazole-5-carboxylates **18a-l** 15

1,2,4-Oxadiazole derivatives **28a-c** and intermediates of their synthesis **24a-c-27a-c** 42

2,4-Disubstituted pyrimidine derivatives **22a-e**, **22c'**, **22e'** and intermediates of their synthesis **20a-e**, **21a-e**, **21c'**, **21e'** 69

Target methyl esters **23a-23aa**, **23r'**, **23s'**, **23z'**, **23aa'** 111

Target 1*H*-benzo[*d*]imidazole-5-carboxylic acid derivatives **12a-12aa**, **12r'**, **12s'**, **12z'**, **12aa'** 178

Target 5-(1*H*-benzo[*d*]imidazol-5-yl)-3-methyl-1,2,4-oxadiazole derivatives **13a-d** 241

HRMS spectra copies 251

General

All solvents and reagents were obtained from commercial sources and used without further purification unless otherwise stated. ^1H and ^{13}C NMR spectra were recorded on a Bruker Avance Neo 400 MHz spectrometer (Bruker, Ettlingen, Germany, 400.1, 100.6 MHz, respectively)) in $CDCl_3$, DMSO-d_6 solutions. Chemical shifts δ are reported in parts per million (ppm); multiplicity: *s*, singlet; *d*, doublet; *t*, triplet; *q*, quartet; *dd*, double of doublets; *m*, multiplet; *br*, broad; the coupling constants *J* are reported in Hz. The structure of the products was determined by analyzing ^1H and ^{13}C NMR spectra; and assignments on a routine basis by a combination of 1D and 2D experiments (HSQC, HMBC). UPLS-MS analyses were performed on a ≪Vanquish Flex≫ chromatograph (Thermo Scientific, Waltham, MA, USA) with Diode Array Detector FG (DAD FG, Thermo Scientific, USA) combined with an ISQ EM Single Quadrupole Mass Spectrometer (Thermo Scientific, USA). A 6-minute gradient separation on an Agilent Poroshell 120 EC-C18 (Agilent Technologies, Santa Clara, CA, USA, 100 mm×2.0 mm, particle size 1.9 μm) column was run under the following conditions: solvent A = water with 0.1% formic acid, solvent B = acetonitrile with 0.1% formic acid, from 0 to 2.5 min—gradient elution from A:B = 9:1 to A:B = 1:9, from 2.5 min to 3.5 min—elution in A:B = 1:9, from 3.5 min to 6 min—equilibration of the chromatographic column in A:B = 19:1 at a flow rate 0.5 mL/min. The column temperature was 40°C, injection volume of the sample was 1 μL. An electrospray ionization source was used to ionize the samples. Ions of positive and negative polarities were detected in the full ion current recording mode; the range of recorded masses was 10–700 *m/z*. The absorption spectra were recorded on a diode array detector at 2 wavelengths: 220 nm and 254 nm. High-resolution mass spectrometry (HRMS) analyses were performed using a Bruker maXis II 4G ETD mass spectrometer (Bruker, Ettlingen, Germany) and an UltiMate 3000 chromatograph (Thermo Scientific, Waltham, MA, USA) equipped with Acclaim RSLC 120 C18 2.2 μm 2.1×100 mm column (Thermo Scientific,Waltham, MA, USA). Spectrum registration mode was electrospray ionization (ESI), with a full scan between *m/z* 100 and 1500, tandem MS (MS/MS) with selection of the three most intense ions, collision-induced dissociation (CID) at 10–40 eV, and nitrogen as a collision gas. Melting points were determined on Melting Point Apparatus SMP50 (Norrscope, Bicknacre, UK) in the following regime (1°C per minute). The target substances were lyophilized using a LABCONCO FreeZone 2.5 l freeze dryer (Labconco, Kansas City, MO, USA) samples were preliminarily frozen in a freezer at −80°C for 5 h, sublimation was carried out for 12 h, residual pressure 0.003 mbar). Thin-layer chromatography (TLC) was carried out on Merck silica gel 60 F254 precoated plates (Merck, Rahway, NJ USA); compounds on TLC were visualized with UV light (254 nm) or by ninhydrin or phosphomolybdic acid staining. Column chromatography was performed on silica gel (Silica 60, 0.04-0.063 mm, Macherey-Nagel, Germany).

Synthetic Procedures and NMR spectra copies

Methyl 4-fluoro-3-nitrobenzoate 15

Procedure to synthesis of methyl 4-fluoro-3-nitrobenzoate **15**. A weighted portion of 4-fluoro-3-nitrobenzoic acid **14** (5.2 g, 28.11 mmol, 1 eq) was dissolved in MeOH (200 ml) under vigorous stirring in a round-bottom flask. Concentrated H_2SO_4 (300 µl, 5.62 mmol, 0.2 eq) was added to the resulting solution, which were next refluxed under vigorous stirring for 28 h, the reaction was controlled by TLC (*n*-hexane:EtOAc = 1:1). After the reaction was completed, the reaction mixture was dissolved in EtOAc (250 ml) and washed with saturated $NaHCO_3$ aqueous solution. The organic layer was collected and dried with anhydrous Na_2SO_4 under vigorous stirring for 1 h, after which the precipitate was filtered off, the solvent was evaporated on a rotary evaporator under reduced pressure, and the residue was dried to form methyl ester **15** as a white powder (5.2 g, 95%).

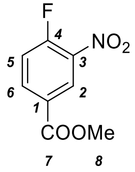

Methyl 4-fluoro-3-nitrobenzoate **15**, white solid, 5.2 g, 95%. **^{1}H NMR (400 MHz, CDCl$_3$, *δ ppm*)**: 8.66 (1H, *dd*, *J* = 7.2, 2.2 Hz, 2-CH), 8.25 (1H, *ddd*, *J* = 8.7, 4.2, 2.2 Hz, 6-CH), 7.32 (1H, *dd*, *J* = 10.2, 8.7 Hz, 5-CH), 3.90 (3H, *s*, 8-CH_3). **^{13}C NMR (101 MHz, CDCl$_3$, *δ ppm*)**: 164.1 (C-7), 158.0 (C-4, *d*, $J_{(C\text{-}F)}$ = 271.6 Hz), 137.3 (C-3, *d*, $J_{(C\text{-}F)}$ = 5.5 Hz), 136.5 (C-6, *d*, $J_{(C\text{-}F)}$ = 9.9 Hz), 127.8 (C-2, *d*, $J_{(C\text{-}F)}$ = 2.0 Hz), 127.3 (C-1, *d*, $J_{(C\text{-}F)}$ = 2.0 Hz), 118.8 (C-5, *d*, $J_{(C\text{-}F)}$ = 21.4 Hz), 52.9 (C-8).

^{1}H NMR spectrum of compound **15**

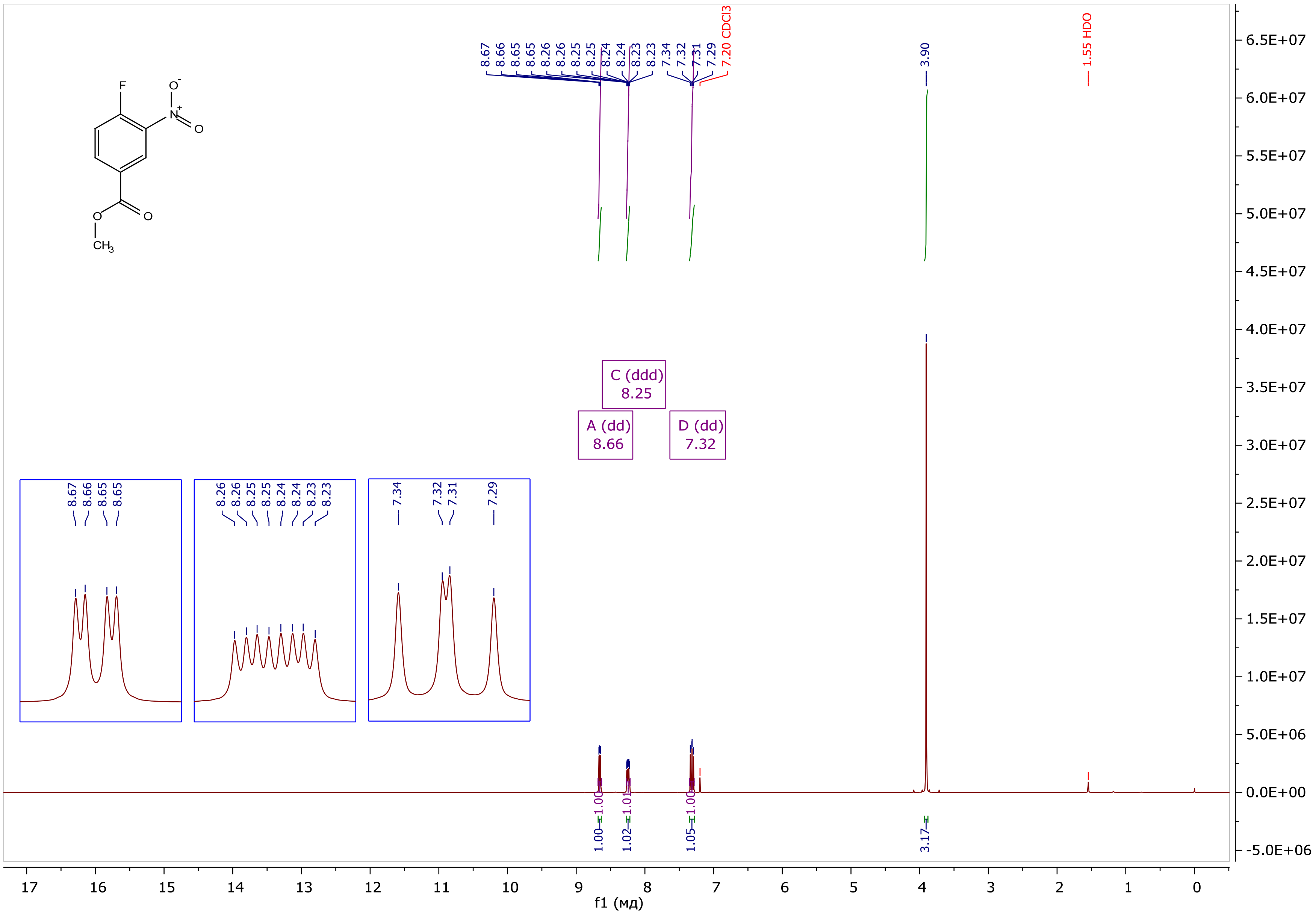

^{13}C NMR spectrum of compound **15**

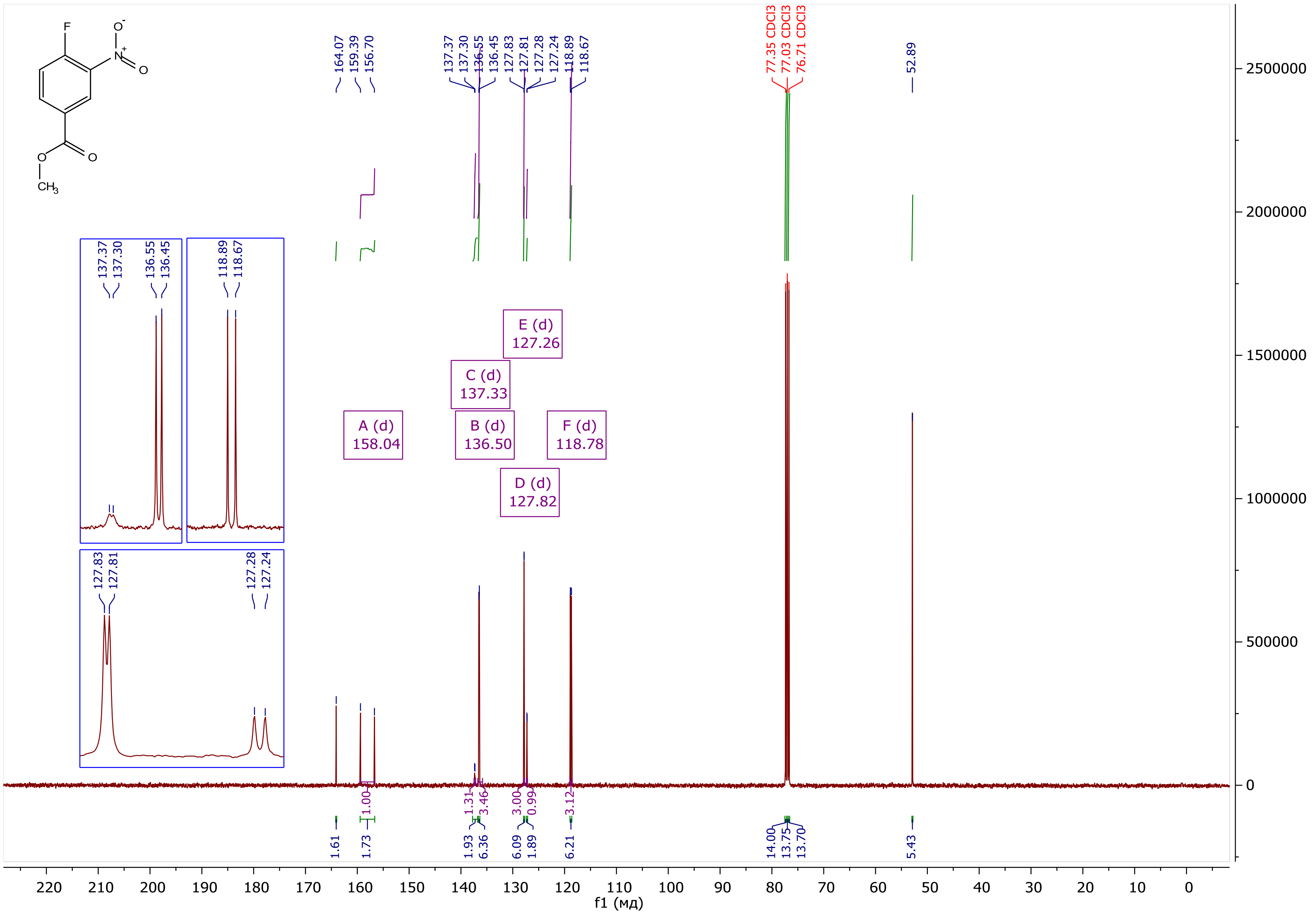

4-Substituted methyl 3-nitrobenzoates 16a-l

General procedure to synthesis of 4-substituted methyl 3-nitrobenzoates **16a-l**. A weighted portion of methyl ether **15** (1 eq) was dissolved in DCM (20 ml) under vigorous stirring in a round bottom flask in a N_2 atmosphere. The corresponding commercially available primary amine (2 eq) was added to the resulting solution, the yellow coloring of the reaction mixture was observed. Then DIPEA (2 eq) was added to the resulting solution and the reaction mixture was stirred at rt for 2 h in a N_2 atmosphere, the reaction was controlled by TLC (*n*-hexane:EtOAc = 4:1). After the reaction was completed, the mixture was sequentially washed with deionized water (2×20 ml), brine solution (1×20 ml), the organic layer was collected and dried with anhydrous Na_2SO_4 under vigorous stirring for 1 h, after which the precipitate was filtered off, and the solvent was evaporated on a rotary evaporator under reduced pressure. The resulting residue was purified by column chromatography on silica gel, eluent – *n*-hexane:EtOAc = 4:1, fractions containing the target product were collected, the solvent was evaporated on a rotary evaporator under reduced pressure, the yellow residue was dried to form the corresponding 4- substituted methyl 3-nitrobenzoate **16a-l** in an excellent yield.

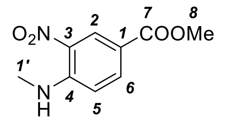

Methyl 4-(methylamino)-3-nitrobenzoate **16a**, yellow solid, 300 mg, 95%. **^{1}H NMR (400 MHz, $CDCl_3$, *δ ppm*)**: 8.81 (1H, *d*, *J* = 2.0 Hz, 2-CH), 8.27 (1H, *br.s.*, NH), 8.01 (1H, *ddd*, *J* = 9.0, 2.1, 0.7 Hz, 6-CH), 6.79 (1H, *d*, *J* = 9.0 Hz, 5-CH), 3.83 (3H, *s*, 8-CH_3), 3.02 (3H, *d*, *J* = 5.1 Hz, 1'-CH_3). **^{13}C NMR (101 MHz, $CDCl_3$, *δ ppm*)**: 165.6 (C-7), 148.5 (C-3), 136.4 (C-2), 131.4 (C-1), 129.4 (C-6), 117.2 (C-4), 113.1 (C-5), 52.1 (C-8), 29.9 (C-1').

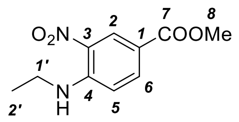

Methyl 4-(ethylamino)-3-nitrobenzoate **16b**, yellow solid, 335 mg, 98%. **^{1}H NMR (400 MHz, $CDCl_3$, *δ ppm*)**: 8.81 (1H, *d*, *J* = 2.1 Hz, 2-CH), 8.20 (1H, *br.s.*, NH), 7.98 (1H, *ddd*, *J* = 9.0, 2.1, 0.7 Hz, 6-CH), 6.79 (1H, *d*, *J* = 9.0 Hz, 5-CH), 3.83 (3H, *s*, 8-CH_3), 3.34 (2H, *qd*, *J* = 7.2, 5.1 Hz, 1'-CH_2), 1.33 (3H, *t*, *J* = 7.2 Hz, 2'-CH_3). **^{13}C NMR (101 MHz, $CDCl_3$, *δ ppm*)**: 165.6 (C-7), 147.6 (C-3), 136.3 (C-2), 131.2 (C-1), 129.5 (C-6), 117.1 (C-4), 113.5 (C-5), 52.1 (C-8), 37.9 (C-1'), 14.2 (C-2').

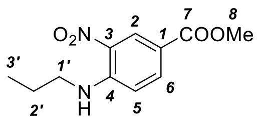

Methyl 4-(propylamino)-3-nitrobenzoate **16c**, yellow solid, 410 mg, 95%. **^{1}H NMR (400 MHz, $CDCl_3$, *δ ppm*)**: 8.81 (1H, *d*, *J* = 2.1 Hz, 2-CH), 8.30 (1H, *br.s.*, NH), 7.97 (1H, *ddd*, *J* = 9.0, 2.1, 0.7 Hz, 6-CH), 6.79 (1H, *d*, *J* = 9.1 Hz, 5-CH), 3.83 (3H, *s*, 8-CH_3), 3.26 (2H, *td*, *J* = 7.2, 5.3 Hz, 1'-CH_2), 1.72 (2H, *h*, *J* = 7.3 Hz, 2'-CH_2), 1.00 (3H, *t*, *J* = 7.4 Hz, 3'-CH_3). **^{13}C NMR (101 MHz, $CDCl_3$, *δ ppm*)**: 165.7 (C-7), 147.8 (C-3), 136.3 (C-2), 131.2 (C-1), 129.6 (C-6), 117.0 (C-4), 113.5 (C-5), 52.1 (C-8), 45.0 (C-1'), 22.2 (C-2'), 11.5 (C-3').

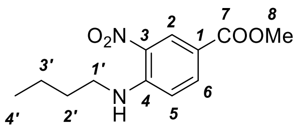

Methyl 4-(butylamino)-3-nitrobenzoate **16d**, yellow solid, 260 mg, 98%. **^{1}H NMR (400 MHz, $CDCl_3$, *δ ppm*)**: 8.81 (1H, *s*, 2-CH), 8.28 (1H, *br.s.*, NH), 7.98 (1H, *dd*, *J* = 9.1, 2.1 Hz, 6-CH), 6.80 (1H, *d*, *J* = 9.1 Hz, 5-CH), 3.63 (3H, *s*, 8-CH_3), 3.29 (2H, *td*, *J* = 7.1, 5.2 Hz, 1'-CH_2), 1.71-1.64 (2H, *m*, 3'-CH_2), 1.47-1.38 (2H, *m*, 2'-CH_2), 0.93 (3H, *t*, *J* = 7.4 Hz, 4'-CH_3). **^{13}C NMR (101 MHz, $CDCl_3$, *δ ppm*)**: 165.7 (C-7), 147.8 (C-3), 136.3 (C-2), 131.2 (C-1), 129.6 (C-6), 117.0 (C-4), 113.5 (C-5), 52.1 (C-8), 43.0 (C-1'), 30.9 (C-3'), 20.2 (C-2'), 13.7 (C-4').

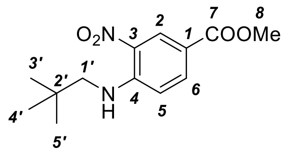

Methyl 4-(neopentylamino)-3-nitrobenzoate **16e**, yellow solid, 480 mg, 98%. **^{1}H NMR (400 MHz, $CDCl_3$, *δ ppm*)**: 8.82 (1H, *d*, *J* = 2.1 Hz, 2-CH), 8.50 (1H, *br.s.*, NH), 7.96 (1H, *ddd*, *J* = 9.1, 2.1, 0.7 Hz, 6-CH), 6.82 (1H, *d*, *J* = 9.1 Hz, 5-CH), 3.83 (3H, *s*, 8-CH_3), 3.07 (2H, *d*, *J* = 5.3 Hz, 1'-CH_2), 1.01 (9H, *s*, 3'-CH_3, 4'-CH_3, 5'-CH_3). **^{13}C NMR (101 MHz, $CDCl_3$, *δ ppm*)**: 165.7 (C-7), 148.2 (C-3), 136.3 (C-2), 131.1 (C-1), 129.6 (C-6), 116.9 (C-4), 113.5 (C-5), 54.9 (C-8), 52.1 (C-1'), 31.9 (C-2'), 27.6 (C-3', C-4', C-5').

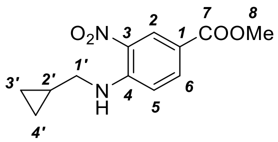

Methyl 4-((cyclopropylmethyl)amino)-3-nitrobenzoate **16f**, yellow solid, 415 mg, 90%. **^{1}H NMR (400 MHz, $CDCl_3$, *δ ppm*)**: 8.81 (1H, *d*, *J* = 2.1 Hz, 2-CH), 8.35 (1H, *br.s.*, NH), 7.97 (1H, *ddd*, *J* = 9.0, 2.1, 0.7 Hz, 6-CH), 6.76 (1H, *d*, *J* = 9.0 Hz, 5-CH), 3.83 (3H, *s*, 8-CH_3), 3.14 (2H, *dd*, *J* = 7.0, 4.9 Hz, 1'-CH_2), 1.17-1.08 (1H, *m*, 2'-CH), 0.63-0.59 (2H, *m*, 3'-CH_2, 4'-CH_2), 0.30-0.26 (2H, *m*, 3'-CH_2, 4'-CH_2). **^{13}C NMR (101 MHz, $CDCl_3$, *δ ppm*)**: 165.6 (C-7), 147.5 (C-3), 136.3 (C-2), 131.2 (C-1), 129.5 (C-6), 117.1 (C-4), 113.6 (C-5), 52.1 (C-8), 48.3 (C-1'), 10.2 (C-2'), 3.9 (C-3', C-4').

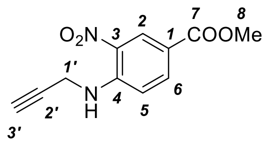

Methyl 3-nitro-4-(prop-2-yn-1-ylamino)benzoate **16g**, yellow solid, 380 мг, 98%. **^{1}H NMR (400 MHz, $CDCl_3$, *δ ppm*)**: 8.84 (1H, *d*, *J* = 2.0 Hz, 2-CH), 8.37 (1H, *br.s.*, NH), 8.06 (1H, *ddd*, *J* = 9.0, 2.1, 0.7 Hz, 6-CH), 6.93 (1H, *d*, *J* = 9.0 Hz, 5-CH), 4.11 (2H, *dd*, *J* = 5.8, 2.5 Hz, 1'-CH_2), 3.84 (3H, *s*, 8-CH_3), 2.27 (1H, *t*, *J* = 2.5 Hz, 3'-CH). **^{13}C NMR (125 MHz, $CDCl_3$, *δ ppm*)**: 165.4 (C-7), 146.6 (C-3), 136.4 (C-2), 132.2 (C-1), 129.3 (C-6), 118.5 (C-4), 113.7 (C-5), 78.0 (C-2'), 73.0 (C-3'), 52.2 (C-8), 32.8 (C-1').

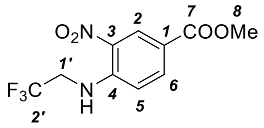

Methyl 3-nitro-4-((2,2,2-trifluoroethyl)amino)benzoate **16h**, yellow solid, 400 mg, 95%. **^{1}H NMR (400 MHz, $CDCl_3$, *δ ppm*)**: 8.84 (1H, *d*, *J* = 2.1 Hz, 2-CH), 8.48 (1H, *br.s.*, NH), 8.07 (1H, *dd*, *J* = 9.0, 1.5 Hz, 6-CH), 6.92 (1H, *d*, *J* = 9.0 Hz, 5-CH), 4.01-3.93 (2H, *m*, 1'-CH_2), 3.85 (3H, *s*, 8-CH_3). **^{13}C NMR (101 MHz, $CDCl_3$, *δ ppm*)**: 165.1 (C-7), 146.5 (C-3), 136.7 (C-2), 132.8 (C-1), 129.3 (C-6), 124.2 (C-2', *q*, $J_{(C-F)}$ = 280.0 Hz), 119.6 (C-4), 113.2 (C-5), 52.3 (C-8), 44.6 (C-1', *q*, $J_{(C-F)}$ = 34.9 Hz).

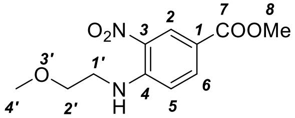

Methyl 4-((2-methoxyethyl)amino)-3-nitrobenzoate **16i**, yellow solid, 400 mg, 98%. **^{1}H NMR (400 MHz, $CDCl_3$, *δ ppm*)**: 8.81 (1H, *d*, *J* = 2.1 Hz, 2-CH), 8.45 (1H, *br.s.*, NH), 7.98 (1H, *ddd*, *J* = 9.0, 2.1, 0.7 Hz, 6-CH), 6.81 (1H, *d*, *J* = 9.0 Hz, 5-CH), 3.83 (3H, *s*, 8-CH_3), 3.64-3.61 (2H, *m*, 2'-CH_2), 3.49-3.45 (2H, *m*, 1'-CH_2), 3.37 (3H, *s*, 4'-CH_3). **^{13}C NMR (101 MHz, $CDCl_3$, *δ ppm*)**: 165.6 (C-7), 147.7 (C-3), 136.3 (C-2), 131.5 (C-1), 129.5 (C-6), 117.3 (C-4), 113.5 (C-5), 70.1 (C-2'), 59.1 (C-4'), 52.1 (C-8), 42.9 (C-1').

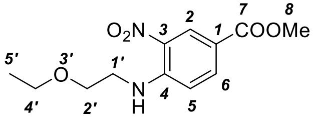

Methyl 4-((2-ethoxyethyl)amino)-3-nitrobenzoate **16j**, yellow solid, 430 mg, 98%. **^{1}H NMR (400 MHz, $CDCl_3$, *δ ppm*)**: 8.82 (1H, *d*, *J* = 2.0 Hz, 2-CH), 8.50 (1H, *br.s.*, NH), 7.98 (1H, *ddd*, *J* = 9.0, 2.1, 0.7 Hz, 6-CH), 6.81 (1H, *d*, *J* = 9.0 Hz, 5-CH), 3.83 (3H, *s*, 8-CH_3), 3.68-3.66 (2H, *m*, 2'-CH_2), 3.52 (2H, *q*, *J* = 7.0 Hz, 4'-CH_2), 3.49-3.45 (2H, *m*, 1'-CH_2), 1.18 (3H, *t*, *J* = 7.0 Hz, 5'-CH_3). **^{13}C NMR (101 MHz, $CDCl_3$, *δ ppm*)**: 165.6 (C-7), 147.7 (C-3), 136.3 (C-2), 131.5 (C-1), 129.5 (C-6), 117.2 (C-4), 113.6 (C-5), 68.0 (C-2'), 66.9 (C-4'), 52.1 (C-8), 43.1 (C-1'), 15.1 (C-5').

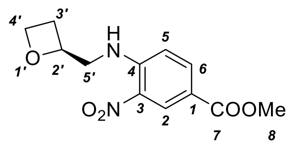

Methyl (S)-3-nitro-4-((oxetan-2-ylmethyl)amino)benzoate **16k**, yellow solid, 3 g, 95%. **^{1}H NMR (400 MHz, $CDCl_3$, *δ ppm*)**: 8.83 (1H, *d*, *J* = 2.1 Hz, 2-CH), 8.65 (1H, *br.s.*, NH), 7.99 (1H, *ddd*, *J* = 8.9, 2.1, 0.7 Hz, 6-CH), 6.88 (1H, *d*, *J* = 9.0 Hz, 5-CH), 5.15-5.01 (1H, *m*, 2'-CH), 4.68 (1H, *ddd*, *J* = 8.5, 7.5, 6.0 Hz, 4'-CH_2), 4.54 (1H, *dt*, *J* = 9.2, 6.0 Hz, 4'-CH_2), 3.83 (3H, *s*, 8-CH_3), 3.66-3.43 (2H, *m*, 5'-CH_2), 2.71 (1H, *dddd*, *J* = 11.3, 8.5, 7.7, 6.0 Hz, 3'-CH_2), 2.54 (1H, *ddt*, *J* = 11.4, 9.2, 7.2 Hz, 3'-CH_2). **^{13}C NMR (101 MHz, $CDCl_3$, *δ ppm*)**: 165.6 (C-7), 148.0 (C-4), 136.4 (C-6), 131.8 (C-3), 129.5 (C-2), 117.6 (C-1), 113.8 (C-5), 79.8 (C-2'), 68.8 (C-4'), 52.1 (C-8), 48.1 (C-5'), 24.7 (C-3').

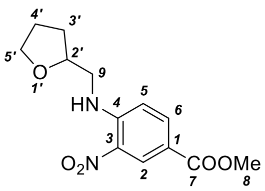

Methyl 3-nitro-4-(((tetrahydrofuran-2-yl)methyl)amino)benzoate **16l**, yellow solid, 3 g, 98%. **^{1}H NMR (400 MHz, $CDCl_3$, *δ ppm*)**: 8.81 (1H, *d*, *J* = 2.1 Hz, 2-CH), 8.48 (1H, *br.s.*, NH), 7.97 (1H, *dd*, *J* = 9.0, 1.9 Hz, 6-CH), 6.83 (1H, *d*, *J* = 9.1 Hz, 5-CH), 4.17-4.11 (1H, *m*, 2'-CH), 3.92-3.86 (1H, *m*, 5'-CH_2), 3.83 (3H, *s*, 8-CH_3), 3.79-3.73 (1H, *m*, 5'-CH_2), 3.49-3.43 (1H, *m*, 9-CH_2), 3.35-3.29 (1H, *m*, 9-CH_2), 2.08-1.97 (1H, *m*, 3'-CH_2), 1.96-1.87 (2H, *m*, 4'-CH_2), 1.68-1.59 (1H, *m*, 3'-CH_2). **^{13}C NMR (101 MHz, $CDCl_3$, *δ ppm*)**: 165.6 (C-7), 147.8 (C-3), 136.3 (C-2), 131.5 (C-1), 129.5 (C-6), 117.3 (C-4), 113.6 (C-5), 76.7 (C-2'), 68.6 (C-5'), 52.1 (C-8), 47.1 (C-9), 29.2 (C-3'), 25.9 (C-4'). **UPLC-MS (ESI+)**: found *m/z* 281.0 $[M + H]^+$; calculated $C_{13}H_{17}N_2O_5^+$ 281.1.

Typical ^{1}H NMR spectrum of 4-Substituted methyl 3-nitrobenzoate (compound **16k** as example)

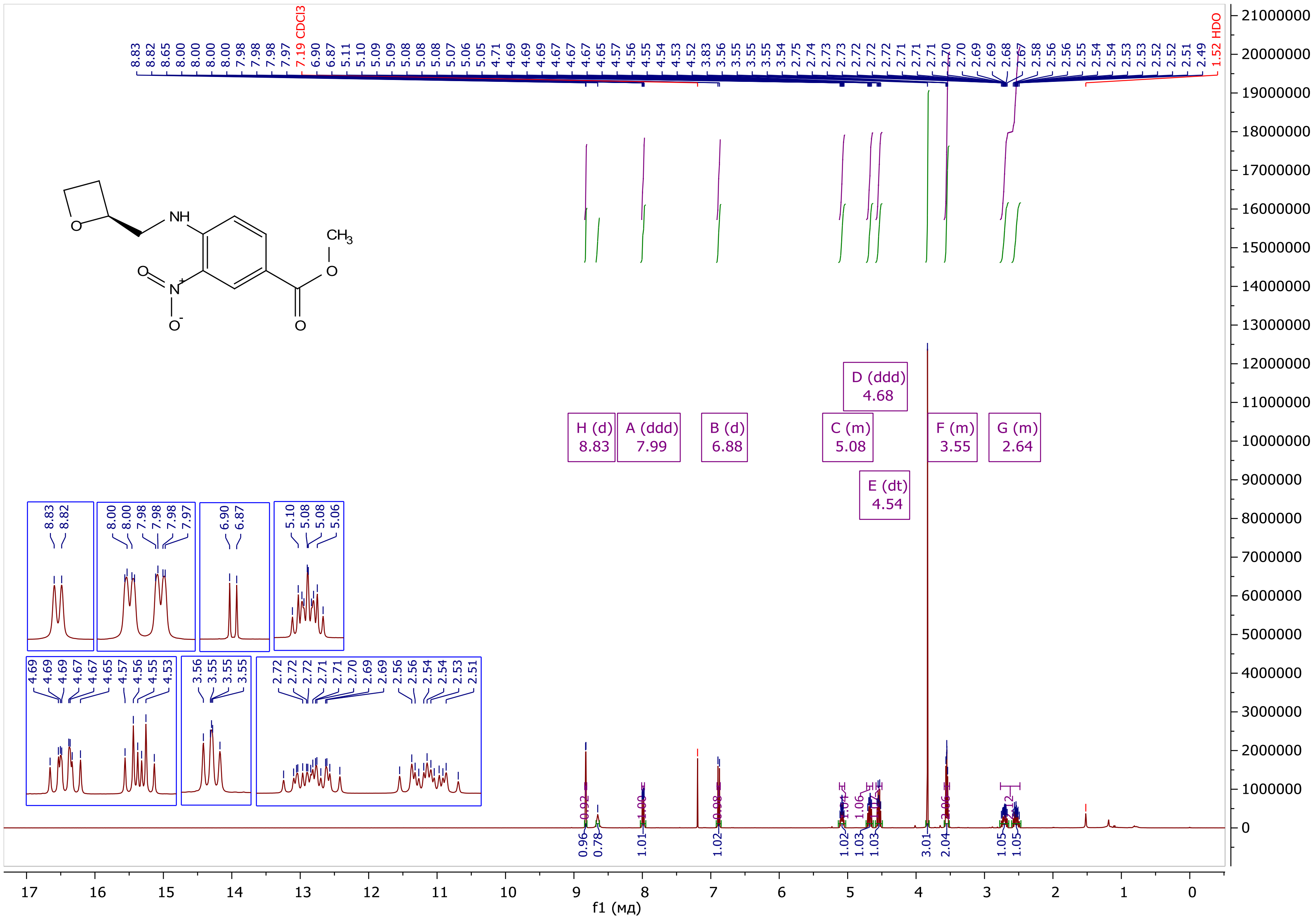

Typical ^{13}C NMR spectrum of 4-Substituted methyl 3-nitrobenzoate (compound **16k** as example)

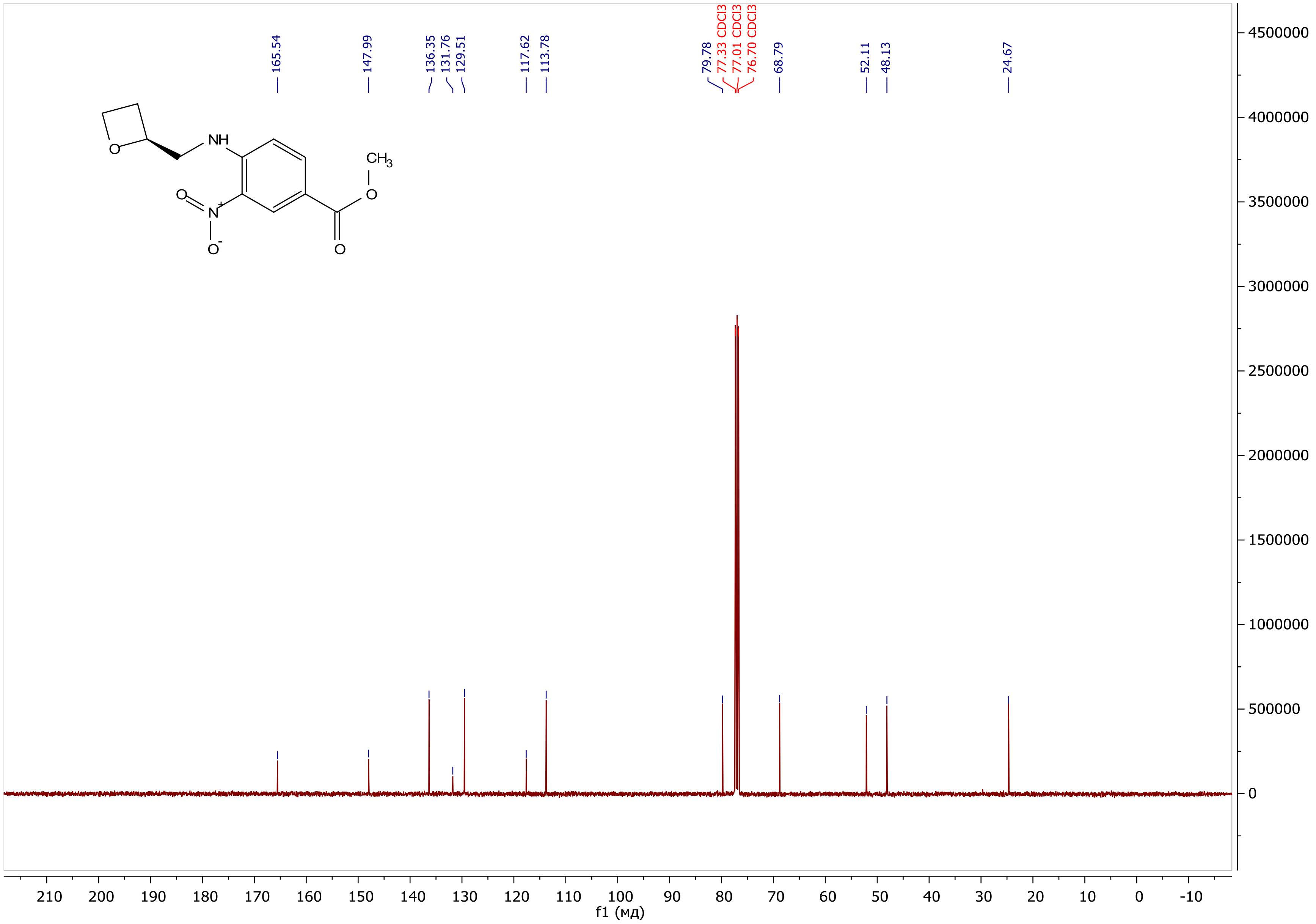

4-Substituted methyl 3-aminobenzoates 17a-l

General procedures to synthesis of 4-substituted methyl 3-aminobenzoates **17a-l**.

Method A. The corresponding compound **16a-e** (1 eq) was dissolved in EtOH at rt under vigorous stirring. A weighted portion of Pd/C (10%, 200 mg per 1 mmol of substrate) was added to the resulting solution, the mixture was hydrogenated at rt for 2 h, the reaction was controlled by TLC (*n*-hexane:EtOAc = 4:1). After the reaction was completed, the mixture was filtered through a celite, the solvent was evaporated to dryness under reduced pressure to form the corresponding technically pure 4-substituted methyl 3-aminobenzoate **17a-e**, which was used in the next stage without additional purification.

Method B. The corresponding compound **16f-l** (1 eq) was dissolved in a THF:MeOH:H_2O = 6:3:1 mixture. A weighted portions of NH_4Cl (5 eq) and Fe (3 eq) were added to the resulting solution. The reaction mixture was refluxed under vigorous stirring for 5 h, the reaction was controlled by TLC (*n*-hexane:EtOAc = 4:1). After the reaction was completed, the solution was filtered through a celite, the corresponding substituted *o*-phenylenediamine **17f-l** were extracted with EtOAc, the organic phase was additionally washed with brine solution (1×20 ml), and then dried with anhydrous Na_2SO_4 under vigorous stirring for 1 h. The precipitate was filtered off, the solvent was evaporated to dryness to form corresponding compound **17f-l**, which was used in the next stage without additional purification.

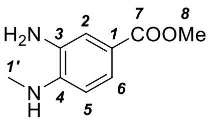

Methyl 3-amino-4-(methylamino)benzoate **17a**, obtained according to **Method A**, dark green solid, 250 mg, 90%. **^{1}H NMR (400 MHz, $CDCl_3$, *δ ppm*)**: 7.54 (1H, *dd*, *J* = 8.3, 1.9 Hz, 6-CH), 7.33 (1H, *d*, *J* = 2.0 Hz, 2-CH), 6.52 (1H, *d*, *J* = 8.3 Hz, 5-CH), 3.78 (3H, *s*, 8-CH_3), 2.85 (3H, *s*, 1'-CH_3). **^{13}C NMR (101 MHz, $CDCl_3$, *δ ppm*)**: 167.6 (C-7), 144.1 (C-3), 132.2 (C-1), 124.4 (C-6), 118.9 (C-4), 117.9 (C-2), 108.9 (C-5), 51.6 (C-8), 30.5 (C-1'). **UPLC-MS** (**ESI**+): found *m/z* 181.1 $[M + H]^+$; calculated $C_9H_{13}N_2O_2^+$ 181.1.

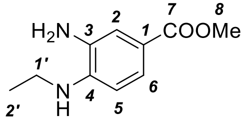

Methyl 3-amino-4-(ethylamino)benzoate **17b**, obtained according to **Method A**, dark green solid, 250 mg, 98%. **^{1}H NMR (400 MHz, $CDCl_3$, *δ ppm*)**: 7.51 (1H, *dd*, *J* = 8.3, 2.0 Hz, 6-CH), 7.34 (1H, *d*, *J* = 2.0 Hz, 2-CH), 6.53 (1H, *d*, *J* = 8.3 Hz, 5-CH), 3.77 (3H, *s*, 8-CH_3), 3.37 (3H, *br.s.*, NH, NH_2), 3.15 (2H, *q*, *J* = 7.1 Hz, 1'-CH_2), 1.25 (3H, *t*, *J* = 7.2 Hz, 2'-CH_3). **^{13}C NMR (101 MHz, $CDCl_3$, *δ ppm*)**: 167.5 (C-7), 142.9 (C-3), 132.0 (C-1), 124.3 (C-6), 118.9 (C-4), 118.2 (C-2), 109.5 (C-5), 51.6 (C-8), 38.3 (C-1'), 14.7 (C-2'). **UPLC-MS** (**ESI**+): found *m/z* 195.1 $[M + H]^+$; calculated $C_{10}H_{15}N_2O_2^+$ 195.1.

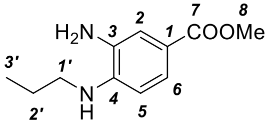

Methyl 3-amino-4-(propylamino)benzoate **17c**, obtained according to **Method A**, dark green solid, 300 mg, 85%. **^{1}H NMR (400 MHz, $CDCl_3$, *δ ppm*)**: 7.51 (1H, *dd*, *J* = 8.3, 2.0 Hz, 6-CH), 7.34 (1H, *d*, *J* = 2.0 Hz, 2-CH), 6.53 (1H, *d*, *J* = 8.3 Hz, 5-CH), 3.77 (3H, *s*, 8-CH_3), 3.27 (3H, *br.s.*, NH, NH_2), 3.07 (2H, *t*, *J* = 7.1 Hz, 1'-CH_2), 1.64 (2H, *h*, *J* = 7.4 Hz, 2'-CH_2), 0.96 (3H, *t*, *J* = 7.4 Hz, 3'-CH_3). **^{13}C NMR (101 MHz, $CDCl_3$, *δ ppm*)**: 167.5 (C-7), 143.1 (C-3), 131.9 (C-1), 124.4 (C-6), 118.7 (C-4), 118.3 (C-2), 109.4 (C-5), 51.6 (C-8), 45.6 (C-1'), 22.6 (C-2'), 11.7 (C-3'). **UPLC-MS** (**ESI**+): found *m/z* 209.0 $[M + H]^+$; calculated $C_{11}H_{17}N_2O_2^+$ 209.1.

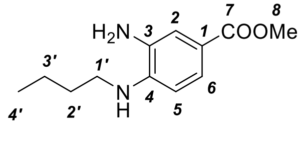

Methyl 3-amino-4-(butylamino)benzoate **17d**, obtained according to **Method A**, dark green solid, 200 mg, 90%. **^{1}H NMR (400 MHz, $CDCl_3$, *δ ppm*)**: 7.51 (1H, *dd*, *J* = 8.4, 1.9 Hz, 6-CH), 7.34 (1H, *d*, *J* = 1.9 Hz, 2-CH), 6.52 (1H, *d*, *J* = 8.4 Hz, 5-CH), 3.77 (3H, *s*, 8-CH_3), 3.38 (3H, *br.s.*, NH, NH_2), 3.10 (2H, *t*, *J* = 7.1 Hz, 1'-CH_2), 1.63-1.56 (2H, *m*, 3'-CH_2), 1.44-1.34 (2H, *m*, 2'-CH_2), 0.90 (3H, *t*, *J* = 7.3 Hz, 4'-CH_3). **^{13}C NMR (101 MHz, $CDCl_3$, *δ ppm*)**: 167.5 (C-7), 143.1 (C-3), 131.9 (C-1), 124.4 (C-6), 118.7 (C-4), 118.2 (C-2), 109.4 (C-5), 51.6 (C-8), 43.5 (C-1'), 31.5 (C-3'), 20.3 (C-2'), 13.9 (C-4'). **UPLC-MS** (**ESI**+): found *m/z* 223.1 $[M + H]^+$; calculated $C_{12}H_{19}N_2O_2^+$ 223.1.

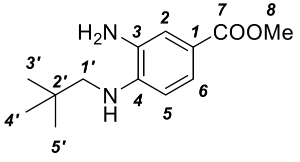

Methyl 3-amino-4-(neopentylamino)benzoate **17e**, obtained according to **Method A**, dark green solid, 400 mg, 90%. **^{1}H NMR (400 MHz, $CDCl_3$, *δ ppm*)**: 7.51 (1H, *dd*, *J* = 8.4, 2.0 Hz, 6-CH), 7.34 (1H, *d*, *J* = 2.0 Hz, 2-CH), 6.52 (1H, *d*, *J* = 8.4 Hz, 5-CH), 3.76 (3H, *s*, 8-CH_3), 3.46 (3H, *br.s.*, NH, NH_2), 2.87 (2H, *s*, 1'-CH_2), 0.95 (9H, *s*, 3'-CH_3, 4'-CH_3, 5'-CH_3). **^{13}C NMR (101 MHz, $CDCl_3$, *δ ppm*)**: 167.5 (C-7), 143.9 (C-3), 131.7 (C-1), 124.6 (C-6), 118.5 (C-2), 118.3 (C-4), 109.2 (C-5), 55.2 (C-8), 51.5 (C-1'), 31.8 (C-2'), 27.7 (C-3', C-4', C-5'). **UPLC-MS** (**ESI**+): found *m/z* 237.1 $[M + H]^+$; calculated $C_{13}H_{21}N_2O_2^+$ 237.3.

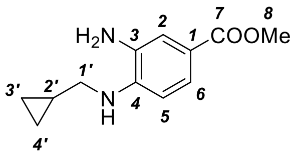

Methyl 3-amino-4-((cyclopropylmethyl)amino)benzoate **17f**, obtained according to **Method B**, dark green solid, 400 mg, 98%. **^{1}H NMR (400 MHz, CDCl$_3$, *δ ppm*)**: 7.50 (1H, *dd*, *J* = 8.4, 2.0 Hz, 6-CH), 7.34 (1H, *d*, *J* = 1.9 Hz, 2-CH), 6.49 (1H, *d*, *J* = 8.3 Hz, 5-CH), 3.73 (3H, *s*, 8-CH$_3$), 3.45 (3H, *br.s.*, NH, NH$_2$), 2.95 (2H, *d*, *J* = 6.9 Hz, 1'-CH$_2$), 1.12-1.02 (1H, *m*, 2'-CH), 0.54-0.50 (2H, *m*, 3'-CH$_2$, 4'-CH$_2$), 0.22-0.18 (2H, *m*, 3'-CH$_2$, 4'-CH$_2$). **^{13}C NMR (101 MHz, CDCl$_3$, *δ ppm*)**: 167.5 (C-7), 143.1 (C-3), 132.1 (C-1), 124.3 (C-6), 118.8 (C-4), 118.0 (C-2), 109.4 (C-5), 51.6 (C-8), 48.8 (C-1'), 10.7 (C-2'), 3.6 (C-3', C-4'). **UPLC-MS (ESI+)**: found *m/z* 221.0 $[M + H]^+$; calculated $C_{12}H_{17}N_2O_2^+$ 221.1.

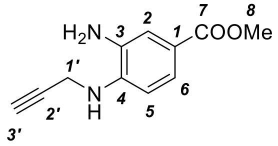

Methyl 3-amino-4-(prop-2-yn-1-ylamino)benzoate **17g**, obtained according to **Method B**, dark green solid, 380 mg, 98%. **^{1}H NMR (400 MHz, CDCl$_3$, *δ ppm*)**: 7.53 (1H, *dd*, *J* = 8.3, 2.0 Hz, 6-CH), 7.36 (1H, *d*, *J* = 1.9 Hz, 2-CH), 6.63 (1H, *d*, *J* = 8.3 Hz, 5-CH), 3.93 (2H, *d*, *J* = 2.5 Hz, 1'-CH$_2$), 3.78 (3H, *s*, 8-CH$_3$), 2.19 (1H, *t*, *J* = 2.4 Hz, 3'-CH). **^{13}C NMR (101 MHz, CDCl$_3$, *δ ppm*)**: 167.4 (C-7), 141.4 (C-3), 133.1 (C-1), 123.9 (C-6), 120.5 (C-4), 118.2 (C-2), 110.5 (C-5), 80.2 (C-2'), 71.9 (C-3'), 51.7 (C-8), 33.4 (C-1'). **UPLC-MS (ESI+)**: found *m/z* 205.0 $[M + H]^+$; calculated $C_{11}H_{13}N_2O_2^+$ 205.1.

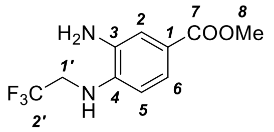

Methyl 3-amino-4-((2,2,2-trifluoroethyl)amino)benzoate **17h**, obtained according to **Method B**, orange solid, 280 mg, 90%. **^{1}H NMR (400 MHz, CDCl$_3$, *δ ppm*)**: 7.52 (1H, *dd*, *J* = 8.4, 1.9 Hz, 6-CH), 7.39 (1H, *d*, *J* = 2.0 Hz, 2-CH), 6.61 (1H, *d*, *J* = 8.3 Hz, 5-CH), 4.28 (1H, *br.s.*, NH), 3.82-3.71 (2H, *m*, 1'-CH$_2$), 3.79 (3H, *s*, 8-CH$_3$). **^{13}C NMR (101 MHz, CDCl$_3$, *δ ppm*)**: 167.1 (C-7), 141.1 (C-3), 132.8 (C-1), 124.8 (C-2', *q*, $J_{(C\text{-}F)}$ = 279.5 Hz), 124.1 (C-6), 121.1 (C-4), 119.3 (C-2), 110.2 (C-5), 51.8 (C-8), 45.5 (C-1', *q*, $J_{(C\text{-}F)}$ = 33.9 Hz). **UPLC-MS (ESI+)**: found *m/z* 249.0 $[M + H]^+$; calculated $C_{10}H_{12}F_3N_2O_2^+$ 249.1.

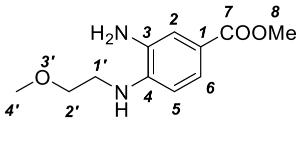

Methyl 3-amino-4-((2-methoxyethyl)amino)benzoate **17i**, obtained according to **Method B**, dark green solid, 290 mg, 90%. **^{1}H NMR (400 MHz, CDCl$_3$, *δ ppm*)**: 7.60 (1H, *dd*, *J* = 8.3, 2.0 Hz, 6-CH), 7.43 (1H, *d*, *J* = 1.9 Hz, 2-CH), 6.62 (1H, *d*, *J* = 8.3 Hz, 5-CH), 3.87 (3H, *s*, 8-CH$_3$), 3.70-3.67 (2H, *m*, 2'-CH$_2$), 3.54 (3H, *br.s.*, NH, NH$_2$), 3.42 (3H, *s*, 4'-CH$_3$), 3.39-3.36 (2H, *m*, 1'-CH$_2$). **^{13}C NMR (101 MHz, CDCl$_3$, *δ ppm*)**: 167.5 (C-7), 142.7 (C-3), 132.4 (C-1), 124.1 (C-6), 119.3 (C-4), 118.1 (C-2), 109.7 (C-5), 70.8 (C-2'), 58.8 (C-4'), 51.6 (C-8), 43.2 (C-1'). **UPLC-MS (ESI+)**: found *m/z* 225.1 $[M + H]^+$; calculated $C_{11}H_{17}N_2O_3^+$ 225.1.

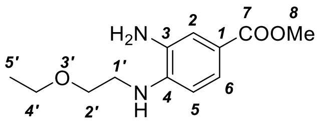

Methyl 3-amino-4-((2-ethoxyethyl)amino)benzoate **17j**, obtained according to **Method B**, dark green solid, 320 mg, 85%. **^{1}H NMR (400 MHz, CDCl$_3$, *δ ppm*)**: 7.50 (1H, *dd*, *J* = 8.3, 2.0 Hz, 6-CH), 7.33 (1H, *d*, *J* = 1.9 Hz, 2-CH), 6.53 (1H, *d*, *J* = 8.3 Hz, 5-CH), 3.77 (3H, *s*, 8-CH$_3$), 3.65-3.62 (2H, *m*, 2'-CH$_2$), 3.48 (2H, *q*, *J* = 7.0 Hz, 4'-CH$_2$), 3.29-3.26 (2H, *m*, 1'-CH$_2$), 1.16 (3H, *t*, *J* = 7.0 Hz, 5'-CH$_3$). **^{13}C NMR (101 MHz, CDCl$_3$, *δ ppm*)**: 167.5 (C-7), 142.7 (C-3), 132.7 (C-1), 124.0 (C-6), 119.2 (C-4), 117.9 (C-2), 109.6 (C-5), 68.7 (C-2'), 66.5 (C-4'), 51.6 (C-8), 43.4 (C-1'), 15.2 (C-5'). **UPLC-MS (ESI+)**: found *m/z* 239.1 $[M + H]^+$; calculated $C_{12}H_{19}N_2O_3^+$ 239.1.

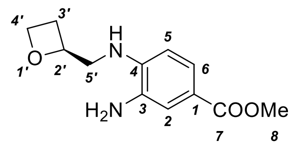

Methyl (S)-3-amino-4-((oxetan-2-ylmethyl)amino)benzoate **17k**, obtained according to **Method B**, dark green solid, 2.3 g, 85%. **^{1}H NMR (400 MHz, CDCl$_3$, *δ ppm*)**: 7.50 (1H, *dd*, *J* = 8.3, 2.0 Hz, 6-CH), 7.34 (1H, *d*, *J* = 1.9 Hz, 2-CH), 6.55 (1H, *d*, *J* = 8.3 Hz, 5-CH), 5.06-4.99 (1H, *m*, 2'-CH), 4.67 (1H, *ddd*, *J* = 8.5, 7.5, 6.0 Hz, 4'-CH$_2$), 4.52 (1H, *dt*, *J* = 9.2, 6.0 Hz, 4'-CH$_2$), 3.78 (3H, *s*, 8-CH$_3$), 3.51-3.26 (2H, *m*, 5'-CH$_2$), 2.71 (1H, *dtd*, *J* = 11.1, 8.1, 6.0 Hz, 3'-CH$_2$), 2.49 (1H, *ddt*, *J* = 11.1, 9.1, 7.1 Hz, 3'-CH$_2$). **^{13}C NMR (101 MHz, CDCl$_3$, *δ ppm*)**: 167.4 (C-7), 142.7 (C-4), 132.8 (C-3), 124.0 (C-6), 119.5 (C-1), 118.1 (C-2), 109.8 (C-5), 80.5 (C-2'), 68.9 (C-4'), 51.6 (C-8), 49.0 (C-5'), 24.9 (C-3'). **UPLC-MS (ESI+)**: found *m/z* 237.2 $[M + H]^+$; calculated $C_{12}H_{17}N_2O_3^+$ 237.1.

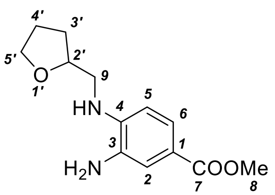

Methyl 3-amino-4-(((tetrahydrofuran-2-yl)methyl)amino)benzoate **17l**, obtained according to **Method B**, dark green solid, 2.4 g, 90%. **^{1}H NMR (400 MHz, CDCl$_3$, *δ ppm*)**: 7.49 (1H, *dd*, *J* = 8.3, 2.0 Hz, 6-CH), 7.32 (1H, *d*, *J* = 1.9 Hz, 2-CH), 6.52 (1H, *d*, *J* = 8.3 Hz, 5-CH), 4.14-4.08 (1H, *m*, 2'-CH), 3.86-3.79 (1H, *m*, 5'-CH$_2$), 3.77 (3H, *s*, 8-CH$_3$), 3.76-3.70 (1H, *m*, 5'-CH$_2$), 3.25-3.21 (1H, *m*, 9'-CH$_2$), 3.09-3.04 (1H, *m*, 9-CH$_2$), 2.05-1.96 (1H, *m*, 3'-CH$_2$), 1.91-1.83 (2H, *m*, 4'-CH$_2$), 1.65-1.58 (1H, *m*, 3'-CH$_2$). **^{13}C NMR (101 MHz, CDCl$_3$, *δ ppm*)**: 167.5 (C-7), 142.8 (C-3), 132.6 (C-1), 124.0 (C-6), 119.2 (C-4), 117.9 (C-2), 109.6 (C-5), 77.4 (C-2'), 68.1 (C-5'), 51.6 (C-8), 48.0 (C-9), 29.2 (C-3'), 25.8 (C-4'). **UPLC-MS (ESI+)**: found *m/z* 251.1 $[M + H]^+$; calculated $C_{13}H_{19}N_2O_3^+$ 251.1.

Typical ^{1}H NMR spectrum of 4-Substituted methyl 3-nitrobenzoate (compound **17k** as example)

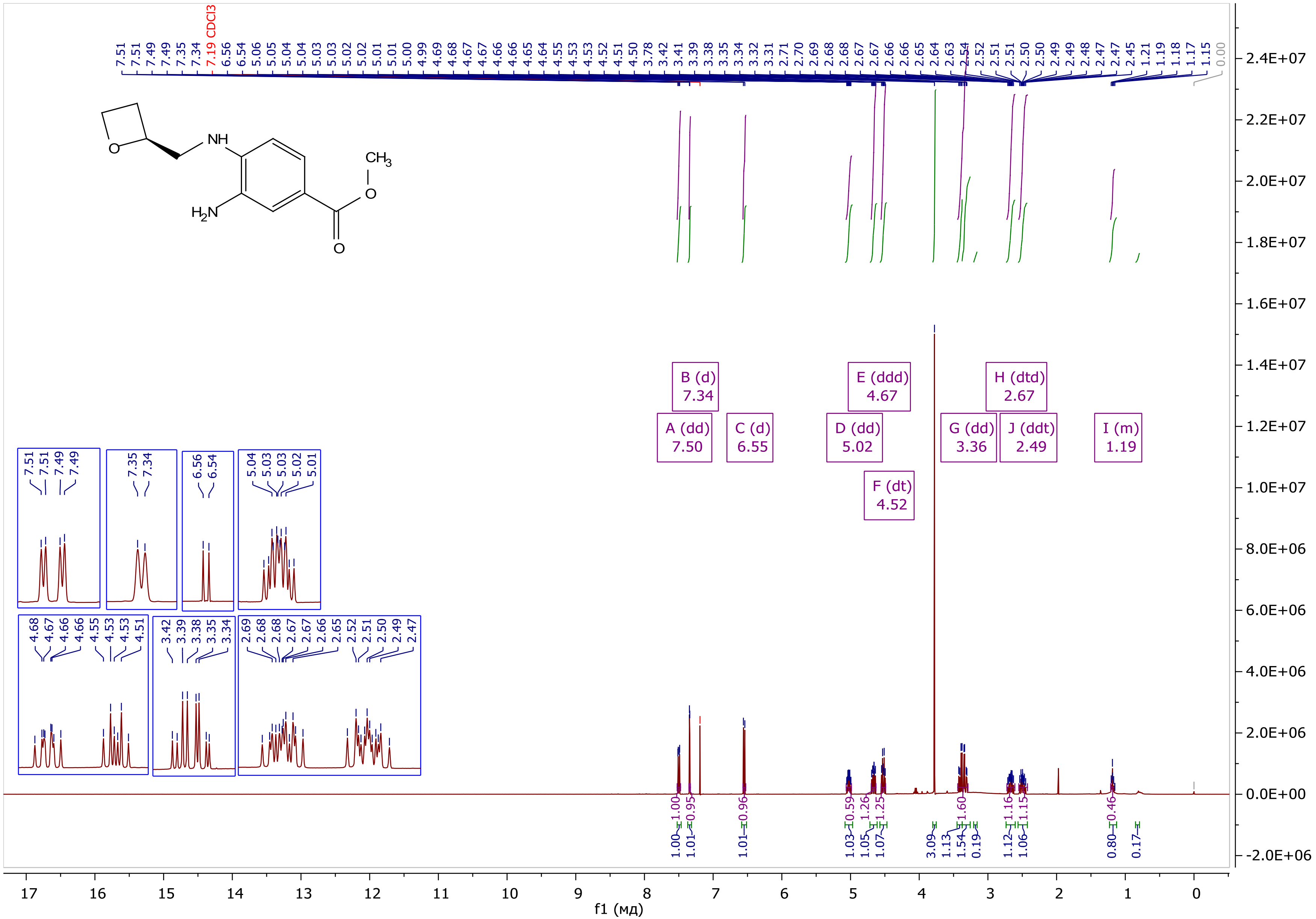

Typical ^{13}C NMR spectrum of 4-Substituted methyl 3-nitrobenzoate (compound **17k** as example)

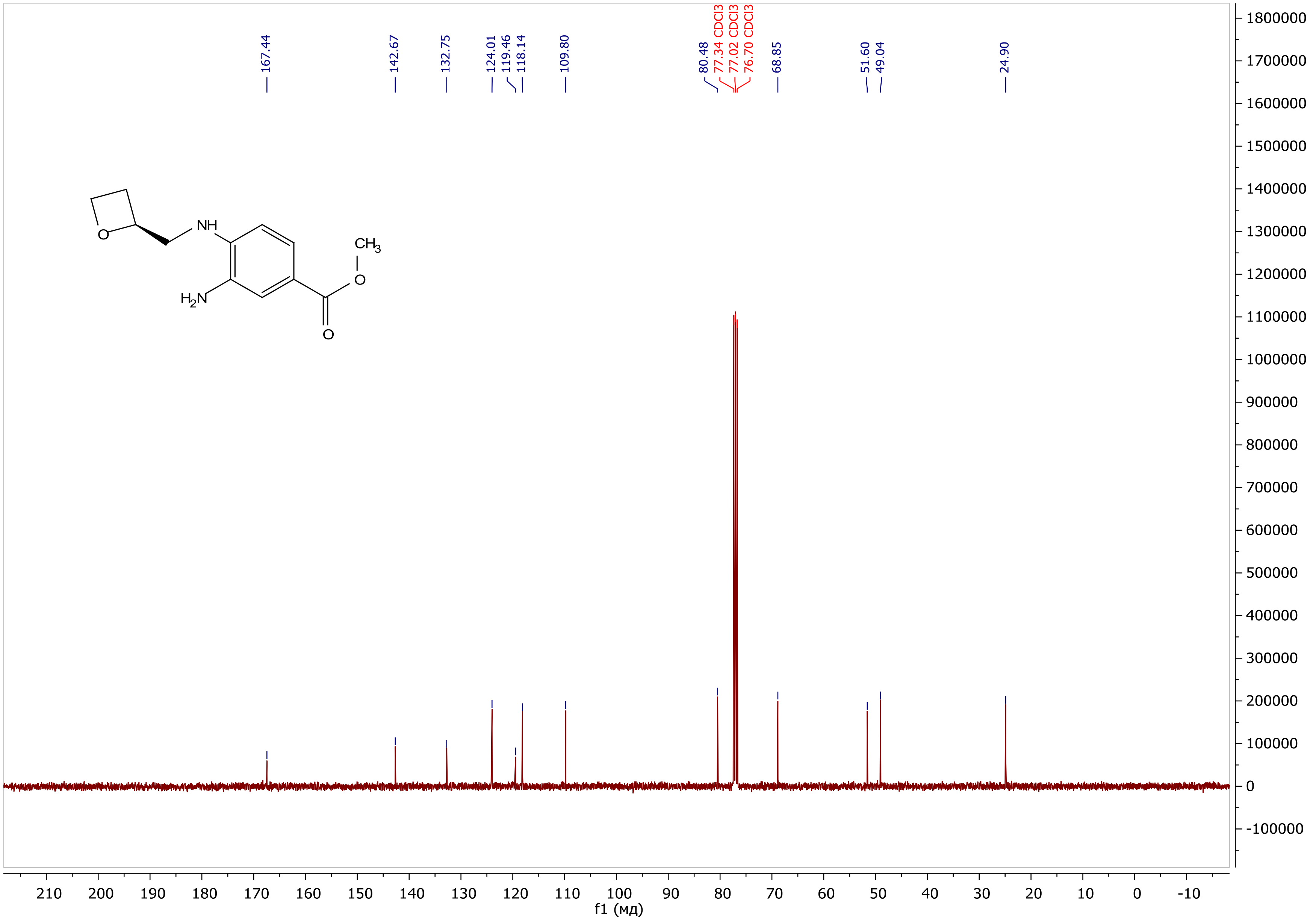

1-Substituted methyl 2-(chloromethyl)-1*H*-benzo[*d*]imidazole-5-carboxylates 18a-l

General procedures to synthesis of 1-Substituted methyl 2-(chloromethyl)-1H-benzo[d]imidazole-5-carboxylates **18a-l**.

Method A. A weighted portion of corresponding 4-substituted methyl 3-aminobenzoate **17a-f**, **17i-j** (1 eq) was dissolved in anhydrous THF (5 ml) in a round bottom flask under vigorous stirring at rt in a N_2 atmosphere. Then DIPEA (1.5 eq) was added to the resulting solution and the reaction mixture was stirred at rt for 10 min, after which chloroacetyl chloride (1.5 eq) was added to the solution. The reaction mixture was stirred in N_2 atmosphere at rt for 1 h, the reaction was controlled by TLC (DCM (sat. NH_3)). After the reaction was completed, the solution was evaporated to dryness on a rotary evaporator under reduced pressure, after which it was dissolved in glacial AcOH (10 ml) and the resulting solution was refluxed under vigorous stirring for 3 h, the reaction was controlled by TLC (DCM (sat. NH_3)). After the reaction was completed, the mixture was poured into cooled deionized water (20 ml) and neutralized with saturated $NaHCO_3$ aqueous solution to pH = 7-8, the corresponding 1*H*-benzo[*d*]imidazole derivative was extracted with EtOAc (3×20 ml). The organic layer was dried with anhydrous Na_2SO_4 under vigorous stirring for 1 h, after which the precipitate was filtered, the solvent was evaporated to dryness on a rotary evaporator under reduced pressure. The solid residue was purified by column chromatography on silica gel (*n*-hexane:EtOAc (gradient from 4:1 to 1:1). The fractions containing the target compound were combined, the solvent was evaporated on a rotary evaporator under reduced pressure to form the corresponding 1*H*-benzo[*d*]imidazole **18a-f**, **18i-j** in a good yields.

Method B. A mixture of the corresponding 4-substituted methyl 3-aminobenzoate **17h-g**, **17k-l** (1 eq), 2-chloro-1,1,1-trimethoxyethane (3 eq), pTSA (0.1 eq) was dissolved in AcN (10 ml). The reaction mixture was refluxed under vigorous stirring for 1 h, the reaction was controlled by TLC (DCM:MeOH = 9:1). After the reaction was completed, the solution was concentrated on a rotary evaporator under reduced pressure, the residue was purified by column chromatography on silica gel (DCM:MeOH (gradient from 100:0 to 85:15)). The fractions containing the target product were combined, the solvent was evaporated on a rotary evaporator under reduced pressure to form the corresponding 1-substituted methyl 2-(chloromethyl)-1*H*-benzo[*d*]imidazole-5-carboxylates **18h-g**, **18k-l** in a good yields.

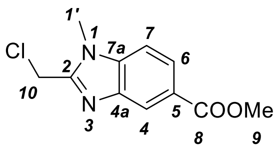

Methyl 2-(chloromethyl)-1-methyl-1H-benzo[d]imidazole-5-carboxylate **18a**, obtained according to **Method A**, light yellow solid, 130 mg, 40%, mp 127-128°C. **^{1}H NMR (400 MHz, CDCl₃, *δ ppm*)**: 8.39 (1H, *s*, 4-CH), 7.99 (1H, *d*, *J* = 8.6 Hz, 6-CH), 7.31 (1H, *d*, *J* = 8.6 Hz, 7-CH), 4.78 (2H, *s*, 10-CH_2), 3.88 (3H, *s*, 9-CH_3), 3.83 (3H, *s*, 1'-CH_3). **^{13}C NMR (101 MHz, $CDCl_3$, *δ ppm*)**: 167.4 (C-8), 150.8 (C-2), 141.7 (C-4a), 139.3 (C-7a), 125.1 (C-6), 125.0 (C-5), 122.7 (C-4), 109.2 (C-7), 52.1 (C-9), 36.7 (C-10), 30.5 (C-1'). **HRMS** (**ESI+**): found *m/z* 239.0633 $[M + H]^+$; calculated $C_{11}H_{12}{}^{35}ClN_2O_2^+$ 239.0509.

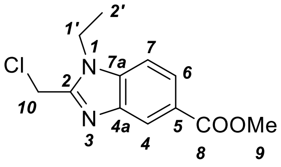

Methyl 2-(chloromethyl)-1-ethyl-1H-benzo[d]imidazole-5-carboxylate **18b**, obtained according to **Method A**, light yellow solid, 130 mg, 40%, mp 124-125°C. **^{1}H NMR (400 MHz, $CDCl_3$, *δ ppm*)**: 8.40 (1H, *d*, *J* = 1.6 Hz, 4-CH), 7.98 (1H, *dd*, *J* = 8.6, 1.6 Hz, 6-CH), 7.33 (1H, *d*, *J* = 8.5 Hz, 7-CH), 4.78 (2H, *s*, 10-CH_2), 4.27 (2H, *q*, *J* = 7.3 Hz, 1'-CH_2), 3.88 (3H, *s*, 9-CH_3), 1.46 (3H, *t*, *J* = 7.3 Hz, 2'-CH_3). **^{13}C NMR (101 MHz, $CDCl_3$, *δ ppm*)**: 167.4 (C-8), 150.4 (C-2), 142.0 (C-4a), 138.2 (C-7a), 125.0 (C-6), 124.9 (C-5), 122.8 (C-4), 109.5 (C-7), 52.1 (C-9), 36.6 (C-10), 39.4 (C-1'), 15.1 (C-2'). **HRMS** (**ESI+**): found *m/z* 253.0777 $[M + H]^+$; calculated $C_{12}H_{14}{}^{35}ClN_2O_2^+$ 253.0666.

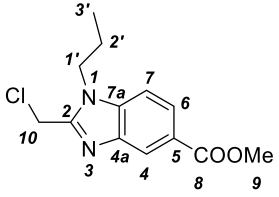

Methyl 2-(chloromethyl)-1-propyl--1H-benzo[d]imidazole-5-carboxylate **18c**, obtained according to **Method A**, light yellow solid, 290 mg, 75%, mp 95-96°C. **^{1}H NMR (400 MHz, $CDCl_3$, *δ ppm*)**: 8.40 (1H, *dd*, *J* = 1.6, 0.7 Hz, 4-CH), 7.97 (1H, *dd*, *J* = 8.6, 1.6 Hz, 6-CH), 7.32 (1H, *dd*, *J* = 8.6, 0.7 Hz, 7-CH), 4.78 (2H, *s*, 10-CH_2), 4.19-4.15 (2H, *m*, 1'-CH_2), 3.88 (3H, *s*, 9-CH_3), 1.88 (2H, *h*, *J* = 7.4 Hz, 2'-CH_2), 0.96 (3H, *t*, *J* = 7.4 Hz, 3'-CH_3). **^{13}C NMR (101 MHz, $CDCl_3$, *δ ppm*)**: 167.4 (C-8), 150.6 (C-2), 141.9 (C-4a), 138.6 (C-7a), 125.0 (C-6), 124.9 (C-5), 122.8 (C-4), 109.7 (C-7), 52.2 (C-9), 46.2 (C-1'), 36.6 (C-10), 23.2 (C-2'), 11.4 (C-3'). **HRMS** (**ESI+**): found *m/z* 267.0940 $[M + H]^+$; calculated $C_{13}H_{16}{}^{35}ClN_2O_2^+$ 267.0822.

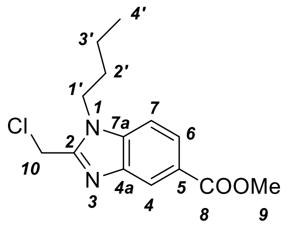

Methyl 1-butyl-2-(chloromethyl)-1H-benzo[d]imidazole-5-carboxylate **18d**, obtained according to **Method A**, light yellow solid, 126 мг, 50%, mp 78-79°C. **^{1}H NMR (400 MHz, $CDCl_3$, *δ ppm*)**: 8.40-8.39 (1H, *m*, 4-CH), 7.97 (1H, *dd*, *J* = 8.6, 1.5 Hz, 6-CH), 7.32 (1H, *dd*, *J* = 8.6, 0.7 Hz, 7-CH), 4.77 (2H, *s*, 10-CH_2), 4.21-4.18 (2H, *m*, 1'-CH_2), 3.88 (3H, *s*, 9-CH_3), 1.86-1.78 (2H, *m*, 3'-CH_2), 1.43-1.33 (2H, *m*, 2'-CH_2), 0.93 (3H, *t*, *J* = 7.4 Hz, 4'-CH_3). **^{13}C NMR (101 MHz, $CDCl_3$, *δ ppm*)**: 167.4 (C-8), 150.6 (C-2), 141.9 (C-4a), 138.6 (C-7a), 125.0 (C-6), 124.9 (C-5), 122.8 (C-4), 109.7 (C-7), 52.1 (C-9), 44.5 (C-1'), 36.6 (C-10), 31.9 (C-3'), 20.3 (C-2'), 13.7 (C-4'). **HRMS (ESI+)**: found *m/z* 281.1085 $[M + H]^+$; calculated $C_{14}H_{18}{}^{35}ClN_2O_2^+$ 281.0979.

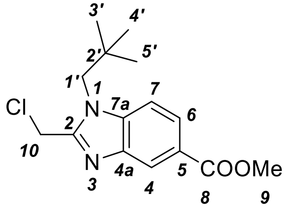

Methyl 2-(chloromethyl)-1-neopentyl-1H-benzo[d]imidazole-5-carboxylate **18e**, obtained according to **Method A**, yellow amorphous substance, 250 mg, 50%, mp 87-88°C. **^{1}H NMR (400 MHz, $CDCl_3$, *δ ppm*)**: 8.40 (1H, *dd*, *J* = 1.5, 0.6 Hz, 4-CH), 7.95 (1H, *dd*, *J* = 8.6, 1.6 Hz, 6-CH), 7.35 (1H, *dd*, *J* = 8.7, 0.6 Hz, 7-CH), 4.84 (2H, *s*, 10-CH_2), 4.06 (2H, *s*, 1'-CH_2), 3.88 (3H, *s*, 9-CH_3), 1.00 (9H, *s*, 3'-CH_3, 4'-CH_3, 5'-CH_3). **^{13}C NMR (101 MHz, $CDCl_3$, *δ ppm*)**: 167.3 (C-8), 151.7 (C-2), 141.9 (C-4a), 139.8 (C-7a), 124.8 (C-5), 124.8 (C-6), 122.6 (C-4), 111.1 (C-7), 55.5 (C-1'), 52.1 (C-9), 37.3 (C-10), 34.9 (C-2'), 28.6 (C-3', C-4', C-5'). **HRMS (ESI+)**: found *m/z* 295.1265 $[M + H]^+$; calculated $C_{15}H_{20}{}^{35}ClN_2O_2^+$ 295.1135.

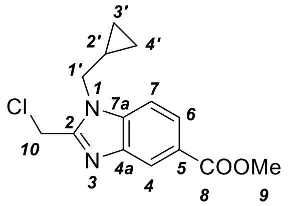

Methyl 2-(chloromethyl)-1-(cyclopropylmethyl)-1H-benzo[d]imidazole-5-carboxylate **18f**, obtained according to **Method A**, white solid, 325 mg, 65%, mp 91-92°C. **^{1}H NMR (400 MHz, $CDCl_3$, *δ ppm*)**: 8.30 (1H, *dd*, *J* = 1.5, 0.7 Hz, 4-CH), 7.88 (1H, *dd*, *J* = 8.6, 1.5 Hz, 6-CH), 7.27 (1H, *d*, *J* = 8.6 Hz, 7-CH), 4.70 (2H, *s*, 10-CH_2), 4.03 (2H, *d*, *J* = 6.7 Hz, 1'-CH_2), 3.78 (3H, *s*, 9-CH_3), 1.19-1.07 (1H, *m*, 2'-CH), 0.54-0.49 (2H, *m*, 3'-CH_2, 4'-CH_2), 0.32-0.28 (2H, *m*, 3'-CH_2, 4'-CH_2). **^{13}C NMR (101 MHz, $CDCl_3$, *δ ppm*)**: 167.4 (C-8), 150.6 (C-2), 141.9 (C-4a), 138.8 (C-7a), 125.0 (C-6), 124.9 (C-5), 122.8 (C-4), 109.9 (C-7), 52.1 (C-9), 48.8 (C-1'), 36.7 (C-10), 11.2 (C-2'), 4.6 (C-3', C-4'). **HRMS (ESI+)**: found *m/z* 279.0941 $[M + H]^+$; calculated $C_{14}H_{16}{}^{35}ClN_2O_2^+$ 279.0822.

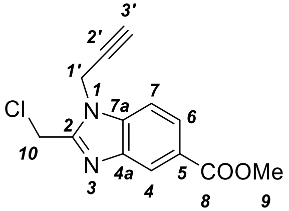

Methyl 2-(chloromethyl)-1-(prop-2-yn-1-yl)-1H-benzo[d]imidazole-5-carboxylate **18g**, obtained according to **Method B**, white solid, 365 mg, 75%, mp 132-133°C. **^{1}H NMR (400 MHz, $CDCl_3$, *δ ppm*)**: 8.41 (1H, *dd*, *J* = 1.6, 0.7 Hz, 4-CH), 8.02 (1H, *dd*, *J* = 8.6, 1.5 Hz, 6-CH), 7.44 (1H, *dd*, *J* = 8.6, 0.7 Hz, 7-CH), 5.00 (2H, *d*, *J* = 2.6 Hz, 1'-CH_2), 4.86 (2H, *s*, 10-CH_2), 3.89 (3H, *s*, 9-CH_3), 2.37 (1H, *t*, *J* = 2.6 Hz, 3'-CH). **^{13}C NMR (101 MHz, $CDCl_3$, *δ ppm*)**: 167.2 (C-8), 150.1 (C-2), 141.7 (C-4a), 138.2 (C-7a), 125.5 (C-6), 125.4 (C-5), 122.9 (C-4), 109.6 (C-7), 75.8 (C-2'), 74.5 (C-3'), 52.2 (C-9), 36.6 (C-10), 33.8 (C-1'). **HRMS (ESI+)**: found *m/z* 263.0631 $[M + H]^+$; calculated $C_{13}H_{12}{}^{35}ClN_2O_2^+$ 263.0509.

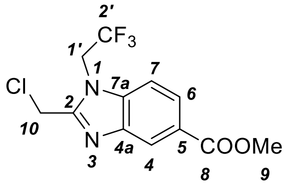

Methyl 2-(chloromethyl)-1-(2,2,2-trifluoroethyl)-1H-benzo[d]imidazole-5-carboxylate **18h**, obtained according to **Method B**, white solid, 190 mg, 55%, mp 124-125°C. **^{1}H NMR (400 MHz, $CDCl_3$, *δ ppm*)**: 8.42 (1H, *d*, *J* = 1.6 Hz, 4-CH), 8.05 (1H, *dd*, *J* = 8.6, 1.6 Hz, 6-CH), 7.36 (1H, *d*, *J* = 8.6 Hz, 7-CH), 4.87 (2H, *q*, *J* = 8.4 Hz, 1'-CH_2), 4.83 (2H, *s*, 10-CH_2), 3.89 (3H, *s*, 9-CH_3). **^{13}C NMR (101 MHz, $CDCl_3$, *δ ppm*)**: 167.0 (C-8), 150.5 (C-2), 141.7 (C-4a), 138.7 (C-7a), 126.2 (C-6), 126.0 (C-5), 123.2 (C-2', *q*, $J_{(C-F)}$ = 280.2 Hz), 123.1 (C-4), 109.5 (C-7), 52.3 (C-9), 45.6 (C-1', *q*, $J_{(C-F)}$ = 36.2 Hz), 36.4 (C-10). **HRMS (ESI+)**: found *m/z* 307.0505 $[M + H]^+$; calculated $C_{12}H_{11}{}^{35}ClF_3N_2O_2^+$ 307.0383.

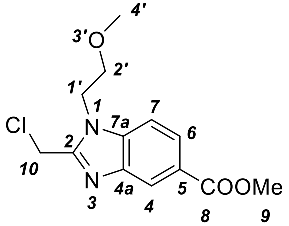

Methyl 2-(chloromethyl)-1-(2-methoxyethyl)-1H-benzo[d]imidazole-5-carboxylate **18i**, obtained according to **Method A**, white solid, 256 mg, 70%, mp 91-92°C. **^{1}H NMR (400 MHz, $CDCl_3$, *δ ppm*)**: 8.40 (1H, *d*, *J* = 1.3 Hz, 4-CH), 7.97 (1H, *dd*, *J* = 8.6, 1.6 Hz, 6-CH), 7.32 (1H, *d*, *J* = 8.6 Hz, 7-CH), 4.87 (2H, *s*, 10-CH_2), 4.42 (2H, *t*, *J* = 5.1 Hz, 1'-CH_2), 3.88 (3H, *s*, 9-CH_3), 3.65 (2H, *t*, *J* = 5.1 Hz, 2'-CH_2), 3.21 (3H, *s*, 4'-CH_3). **^{13}C NMR (101 MHz, $CDCl_3$, *δ ppm*)**: 167.4 (C-8), 151.8 (C-2), 141.9 (C-4a), 138.6 (C-7a), 125.0 (C-6), 124.9 (C-5), 122.7 (C-4), 109.5 (C-7), 70.7 (C-2'), 59.2 (C-4'), 52.2 (C-9), 44.6 (C-1'), 36.9 (C-10). **HRMS (ESI+)**: found *m/z* 283.0903 $[M + H]^+$; calculated $C_{13}H_{16}{}^{35}ClN_2O_3^+$ 283.0771.

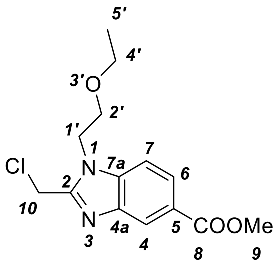

Methyl 2-(chloromethyl)-1-(2-ethoxyethyl)-1H-benzo[d]imidazole-5-carboxylate **18j**, obtained according to **Method A**, white solid, 300 mg, 75%, mp 54-55°C. **^{1}H NMR (400 MHz, CDCl$_3$, *δ ppm*)**: 8.40 (1H, *dd*, *J* = 1.6, 0.6 Hz, 4-CH), 7.97 (1H, *dd*, *J* = 8.5, 1.6 Hz, 6-CH), 7.33 (1H, *d*, *J* = 8.6, 0.7 Hz, 7-CH), 4.91 (2H, *s*, 10-CH_2), 4.42 (2H, *t*, *J* = 5.1 Hz, 1'-CH_2), 3.88 (3H, *s*, 9-CH_3), 3.69 (2H, *t*, *J* = 5.1 Hz, 2'-CH_2), 3.32 (2H, *q*, *J* = 7.0 Hz, 4'-CH_2), 1.02 (3H, *t*, *J* = 7.0 Hz, 5'-CH_3). **^{13}C NMR (101 MHz, CDCl$_3$, *δ ppm*)**: 167.4 (C-8), 151.9 (C-2), 141.7 (C-4a), 138.5 (C-7a), 125.0 (C-6), 124.9 (C-5), 122.6 (C-4), 109.6 (C-7), 68.6 (C-2'), 67.0 (C-4'), 52.1 (C-9), 44.8 (C-1'), 36.8 (C-10), 15.0 (C-5'). **HRMS** (**ESI+**): found *m/z* 297.1041 $[M + H]^+$; calculated $C_{14}H_{18}{}^{35}ClN_2O_3^+$ 297.0928.

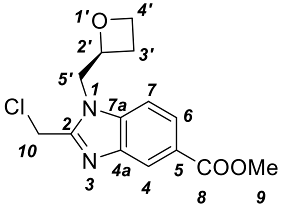

Methyl (S)-2-(chloromethyl)-1-(oxetan-2-ylmethyl)-1H-benzo[d]imidazole-5-carboxylate **18k**, obtained according to **Method B**, white solid, 2.1 г, 75%, mp 128-129°C. **^{1}H NMR (400 MHz, CDCl$_3$, *δ ppm*)**: 8.41 (1H, *d*, *J* = 1.5 Hz, 4-CH), 7.98 (1H, *dd*, *J* = 8.6, 1.6 Hz, 6-CH), 7.34 (1H, *d*, *J* = 8.6 Hz, 7-CH), 5.17-5.11 (1H, *m*, 2'-CH), 4.98-4.91 (2H, *m*, 10-CH_2), 4.57-4.51 (1H, *m*, 4'-CH_2), 4.54-4.41 (2H, *m*, 5'-CH_2), 4.24 (1H, *dt*, *J* = 9.2, 6.0 Hz, 4'-CH_2), 3.88 (3H, *s*, 9-CH_3), 2.67 (1H, *dtd*, *J* = 11.4, 8.1, 5.9 Hz, 3'-CH_2), 2.33 (1H, *ddt*, *J* = 11.5, 9.2, 7.2 Hz, 3'-CH_2). **^{13}C NMR (101 MHz, CDCl$_3$, *δ ppm*)**: 167.3 (C-8), 151.9 (C-2), 141.9 (C-4a), 139.1 (C-7a), 125.2 (C-6), 125.1 (C-5), 122.8 (C-4), 109.7 (C-7), 80.4 (C-2'), 68.4 (C-4'), 52.1 (C-9), 49.5 (C-5'), 37.1 (C-10), 24.4 (C-3'). **HRMS** (**ESI+**): found *m/z* 295.0843 [M + H]+; calculated $C_{14}H_{16}{}^{35}ClN_2O_3^+$ 295.0771

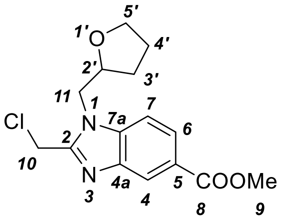

Methyl 2-(chloromethyl)-1-((tetrahydrofuran-2-yl)methyl)-1H-benzo[d]imidazole-5-carboxylate **18l**, obtained according to **Method B**, white solid, 2.1 г, 70%, mp 102-103°C. **^{1}H NMR (400 MHz, CDCl$_3$, *δ ppm*)**: 8.40-8.39 (1H, *m*, 4-CH), 7.97 (1H, *dd*, *J* = 8.6, 1.5 Hz, 6-CH), 7.33 (1H, *dd*, *J* = 8.6, 0.7 Hz, 7-CH), 4.92 (2H, *s*, 10-CH_2), 4.40 (1H, *dd*, *J* = 15.2, 2.8 Hz, 11-CH_2), 4.27 (1H, *dd*, *J* = 15.2, 7.1 Hz, 11-CH_2), 4.20-4.13 (1H, *m*, 2'-CH), 3.88 (3H, *s*, 9-CH_3), 3.79-3.74 (1H, *m*, 5'-CH_2), 3.69-3.63 (1H, *m*, 5'-CH_2), 2.08-1.99 (1H, *m*, 3'-CH_2), 1.88-1.73 (2H, *m*, 4'-CH_2), 1.57-1.50 (1H, *m*, 3'-CH_2). **^{13}C NMR (101 MHz, CDCl$_3$, *δ ppm*)**: 167.4 (C-8), 151.8 (C-2), 141.8 (C-4a), 139.0 (C-7a), 125.0 (C-6), 124.9 (C-5), 122.7 (C-4), 109.7 (C-7), 77.7 (C-2'), 68.3 (C-5'), 52.1 (C-9), 48.2 (C-11), 37.1 (C-10), 29.1 (C-3'), 25.7 (C-4'). **HRMS** (**ESI+**): found *m/z* 309.0997 [M + H]+; calculated $C_{15}H_{18}{}^{35}ClN_2O_3^+$ 309.0928.

^{1}H NMR spectrum of compound **18a**

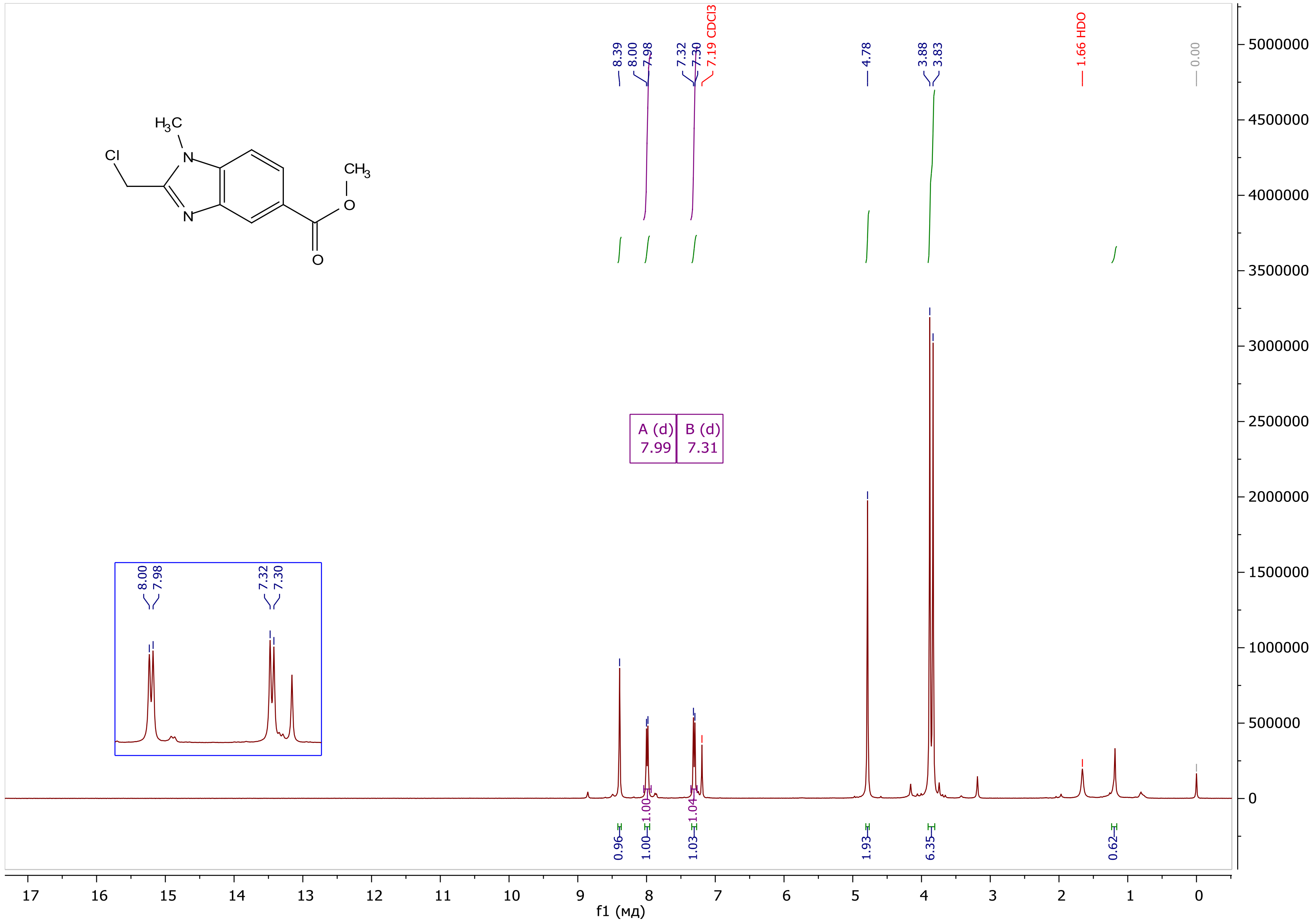

^{13}C NMR spectrum of compound **18a**

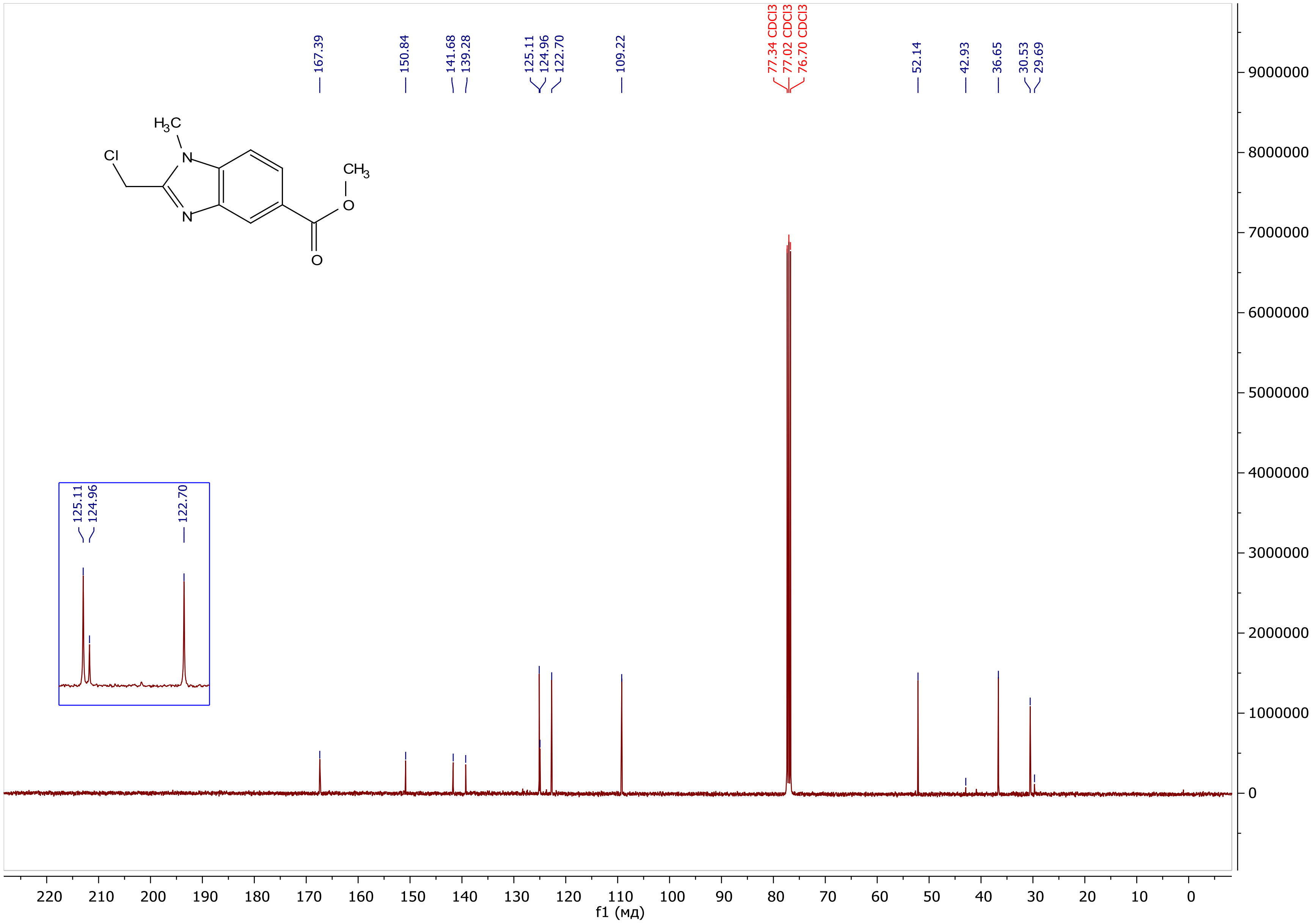

^{1}H NMR spectrum of compound **18b**

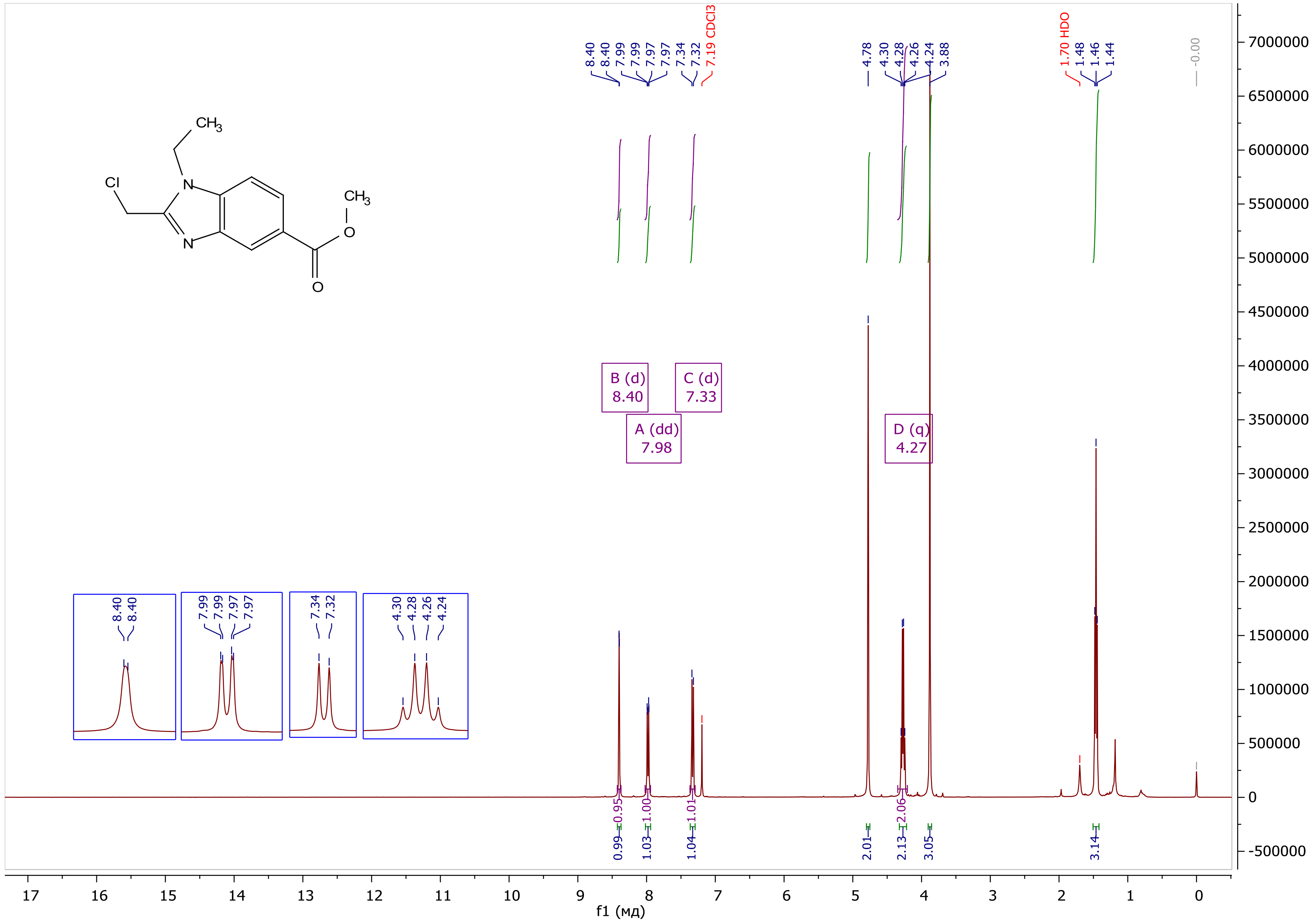

^{13}C NMR spectrum of compound **18b**

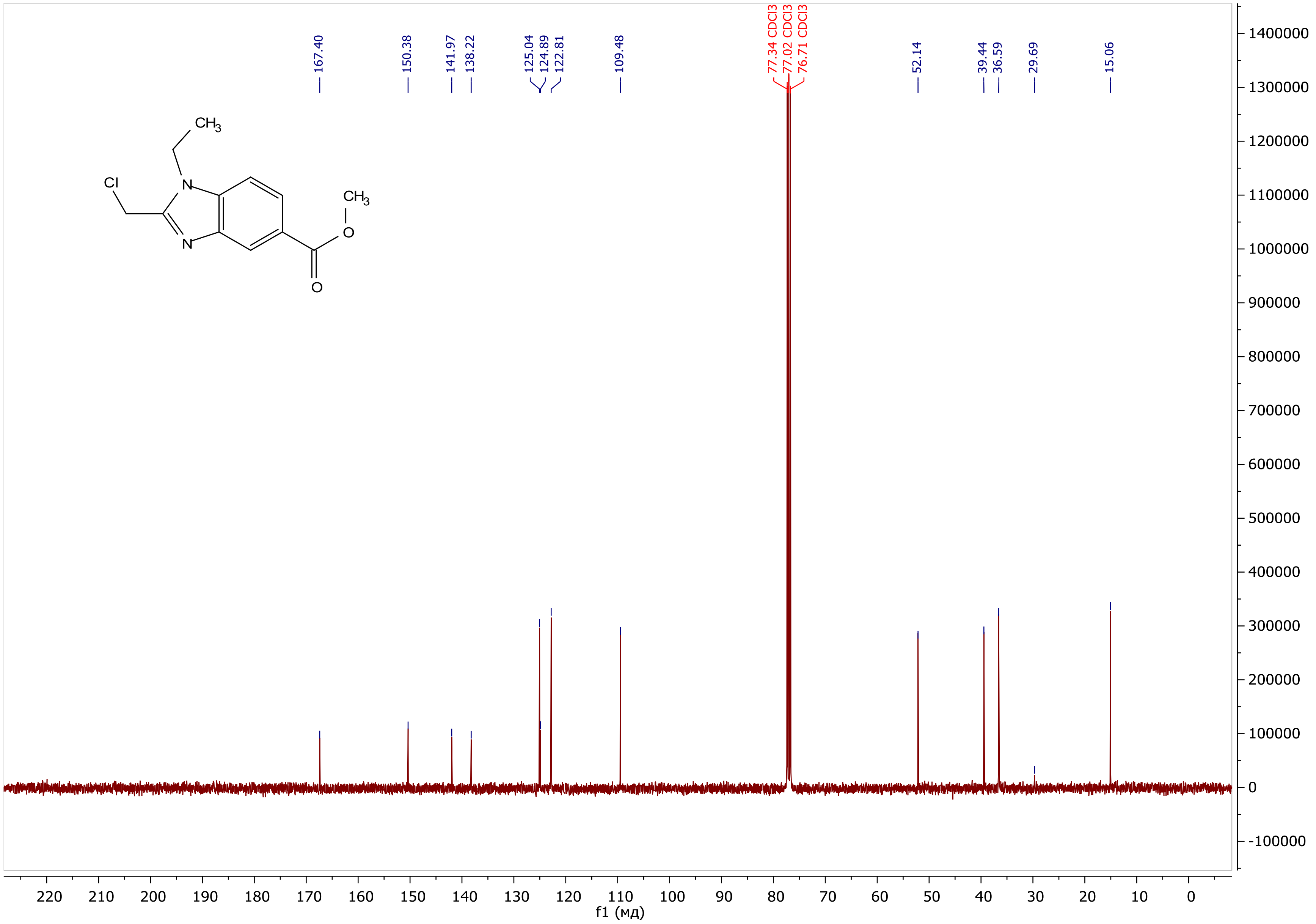

^{1}H NMR spectrum of compound **18c**

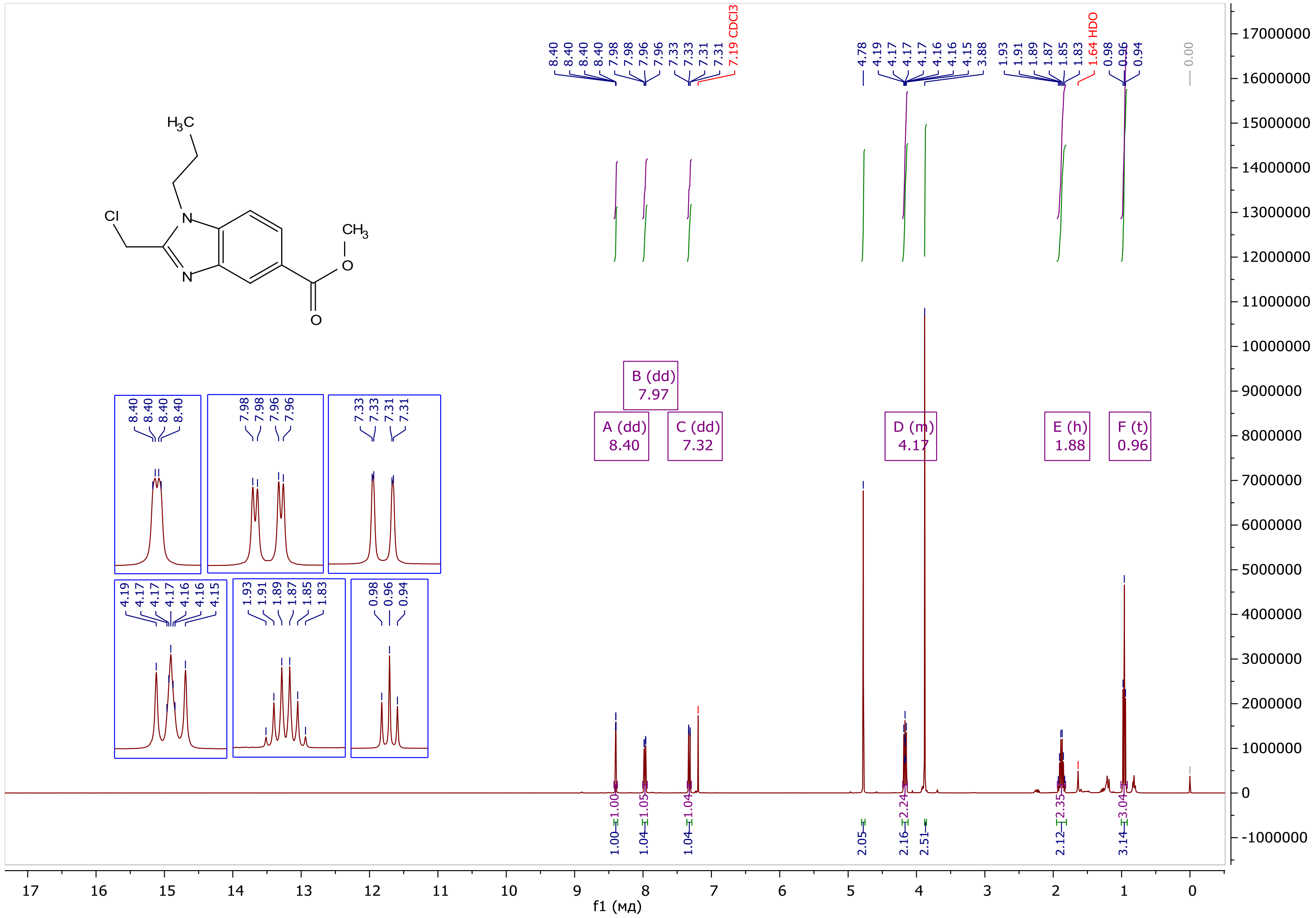

^{13}C NMR spectrum of compound **18c**

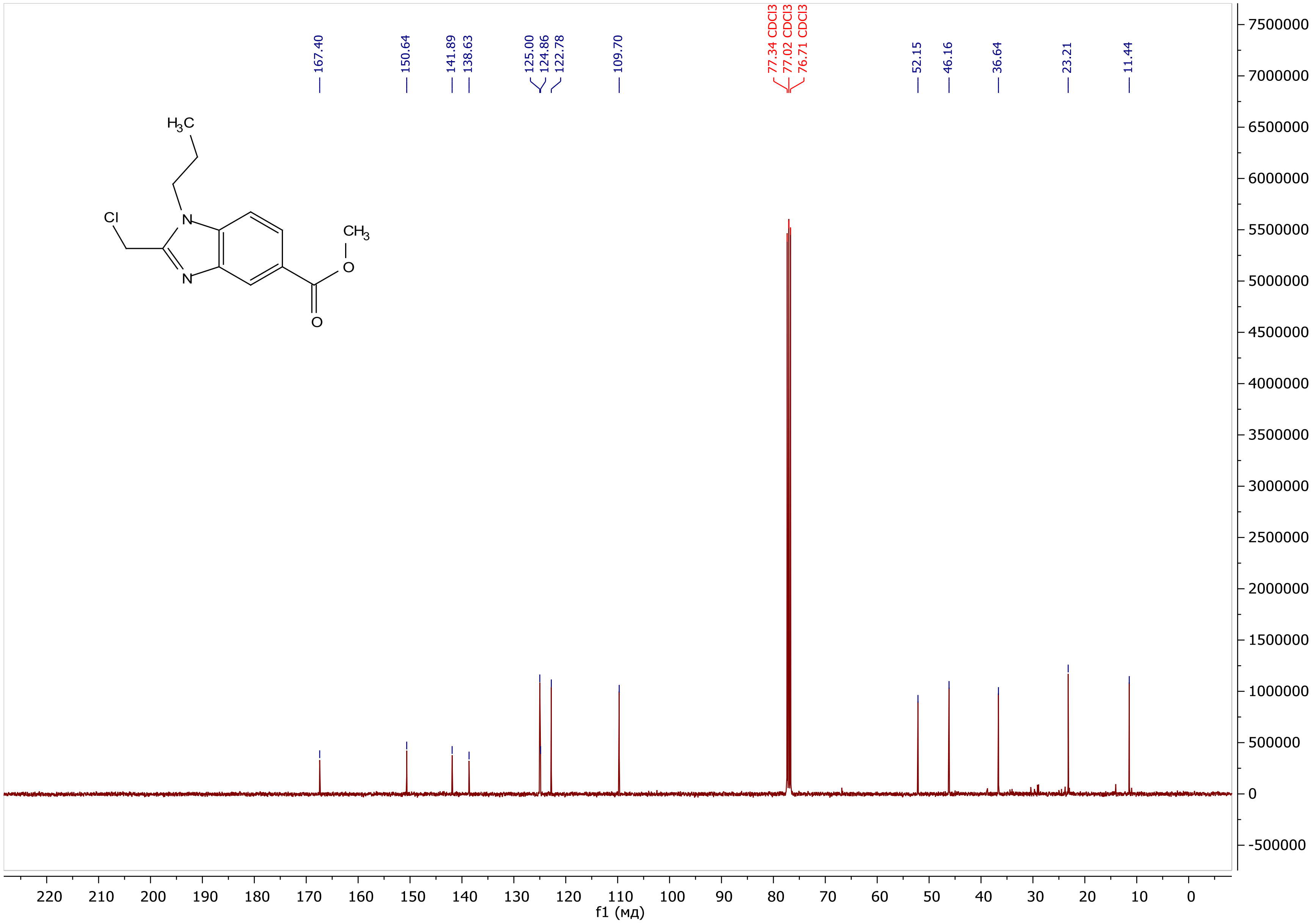

^{1}H NMR spectrum of compound **18d**

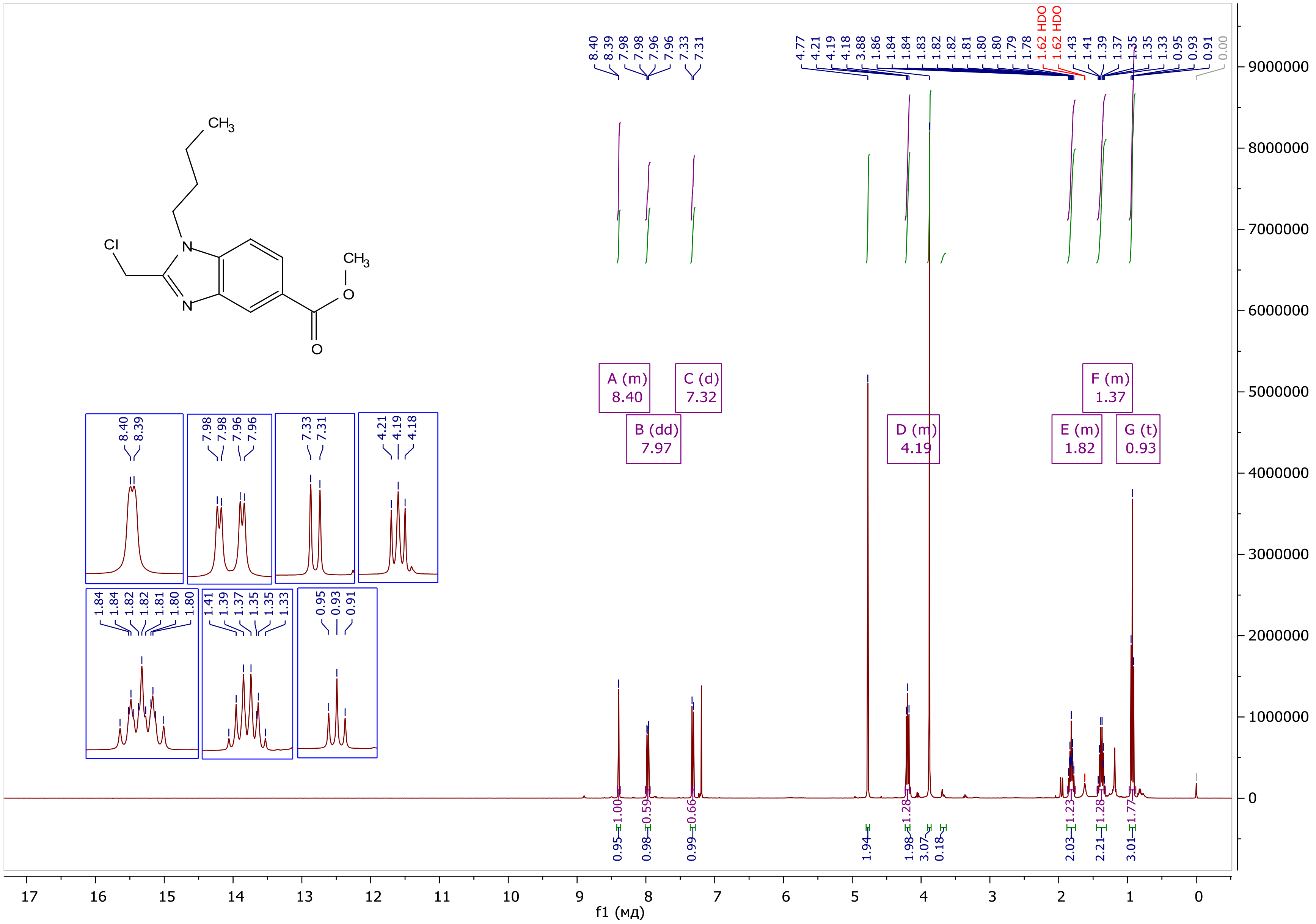

^{13}C NMR spectrum of compound **18d**

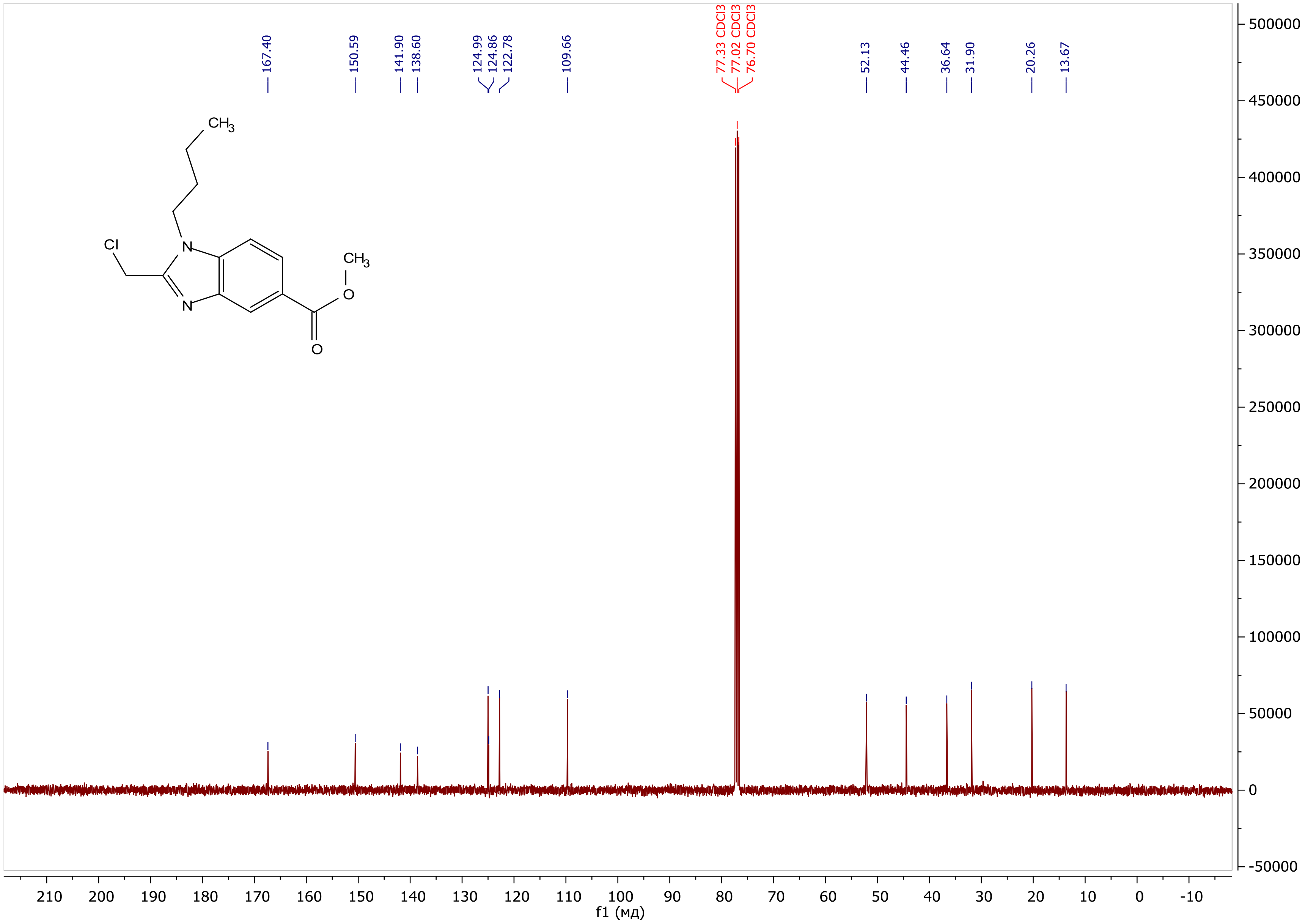

^{1}H NMR spectrum of compound **18e**

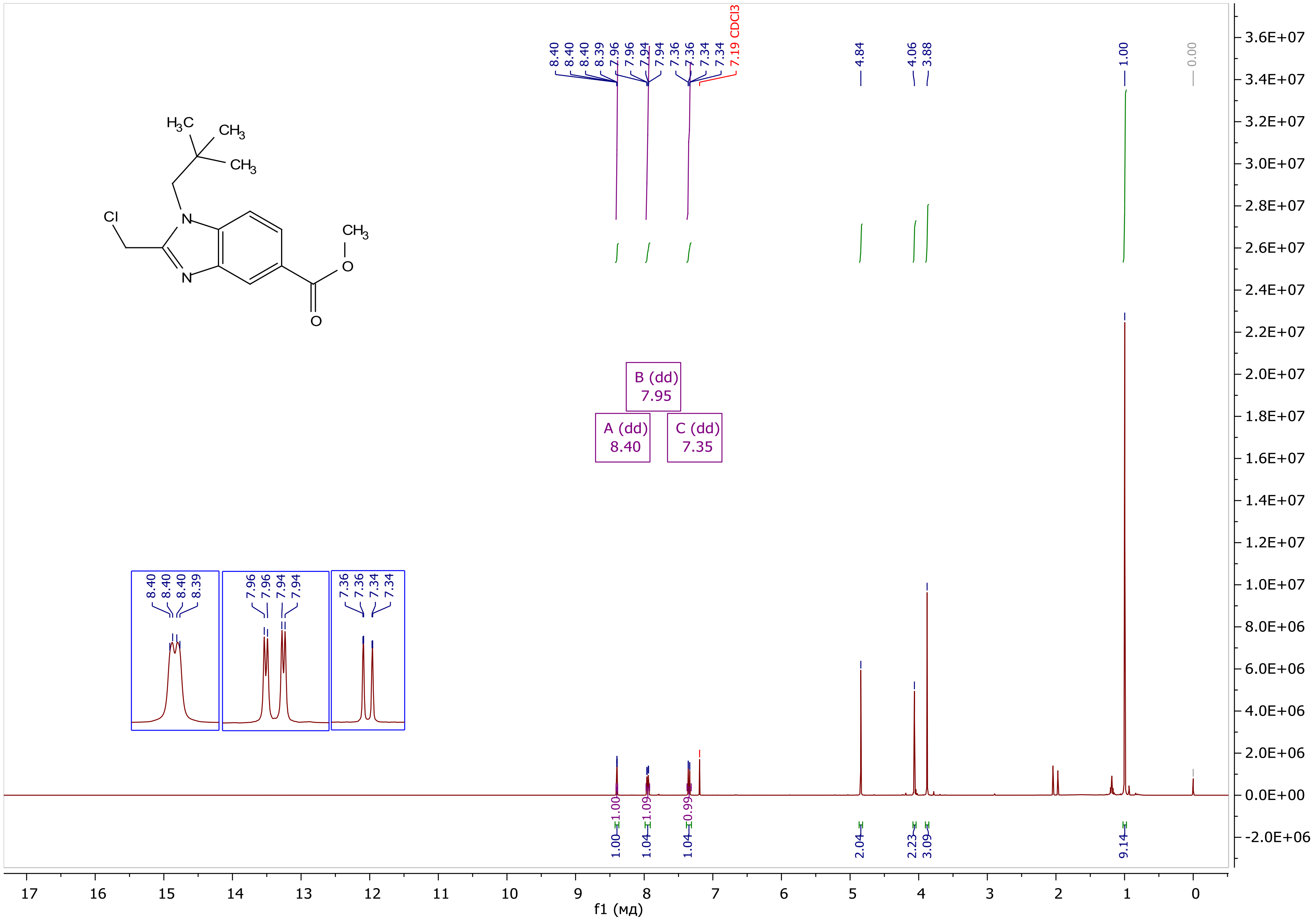

^{13}C NMR spectrum of compound **18e**

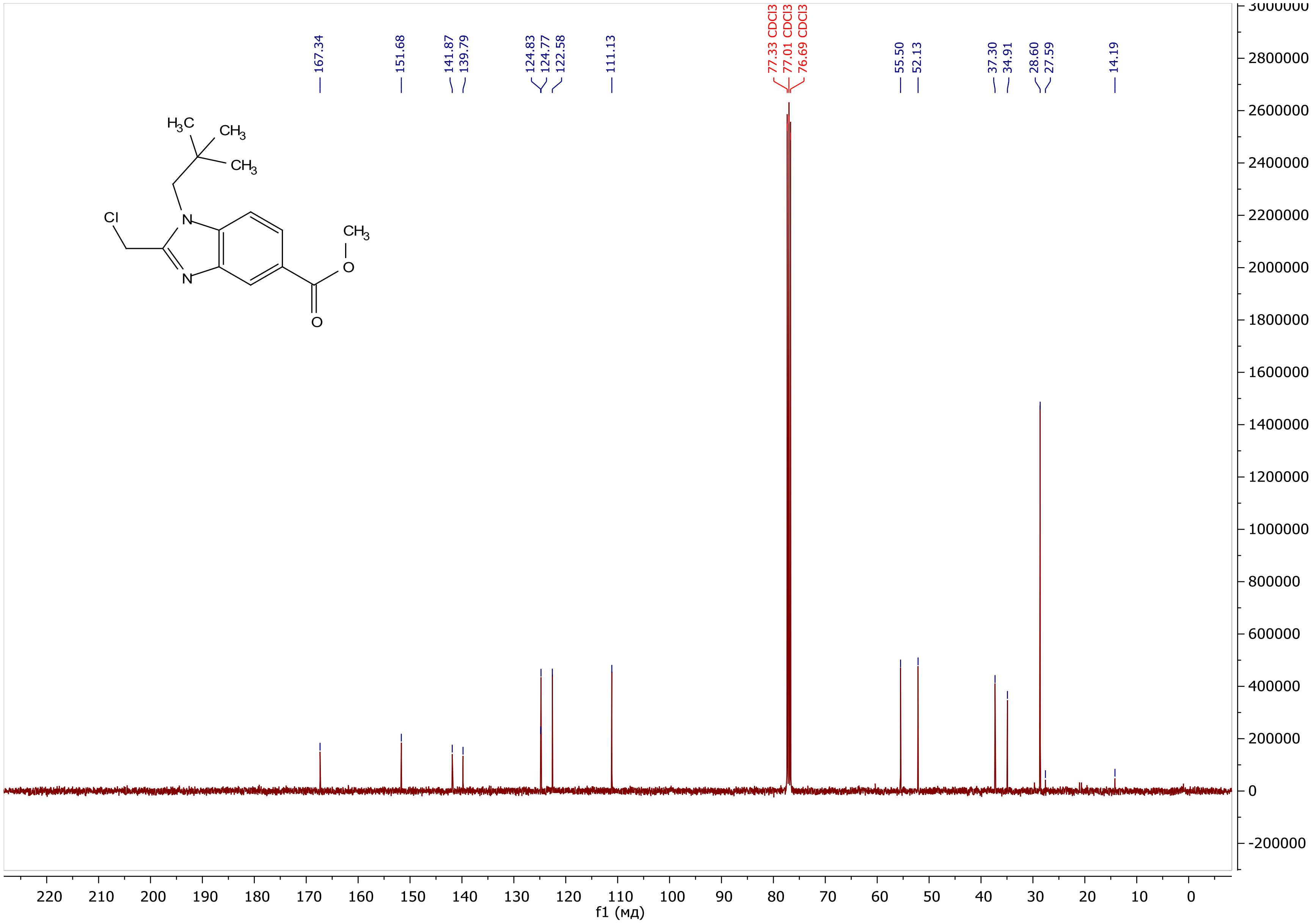

^{1}H NMR spectrum of compound **18f**

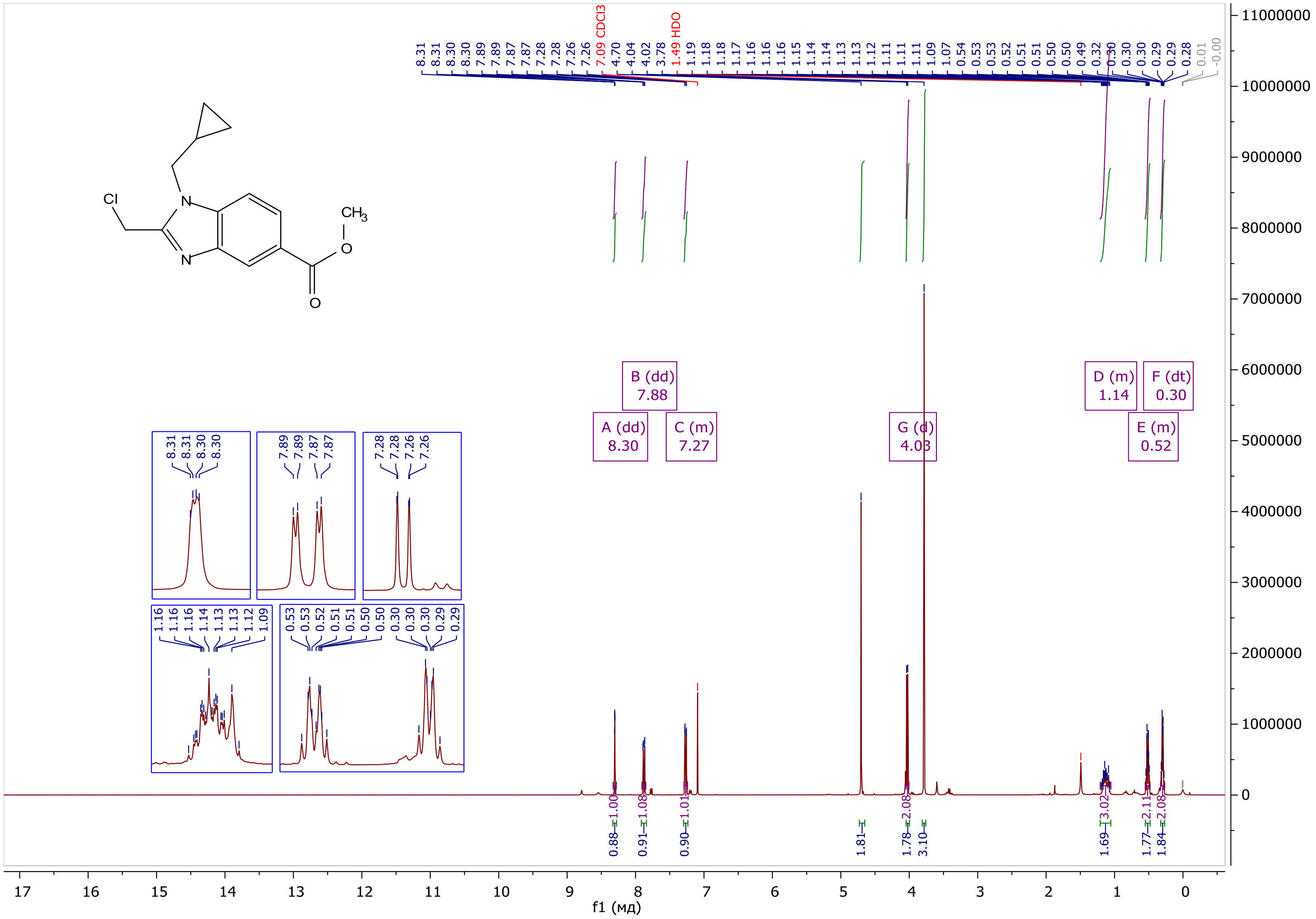

^{13}C NMR spectrum of compound **18f**

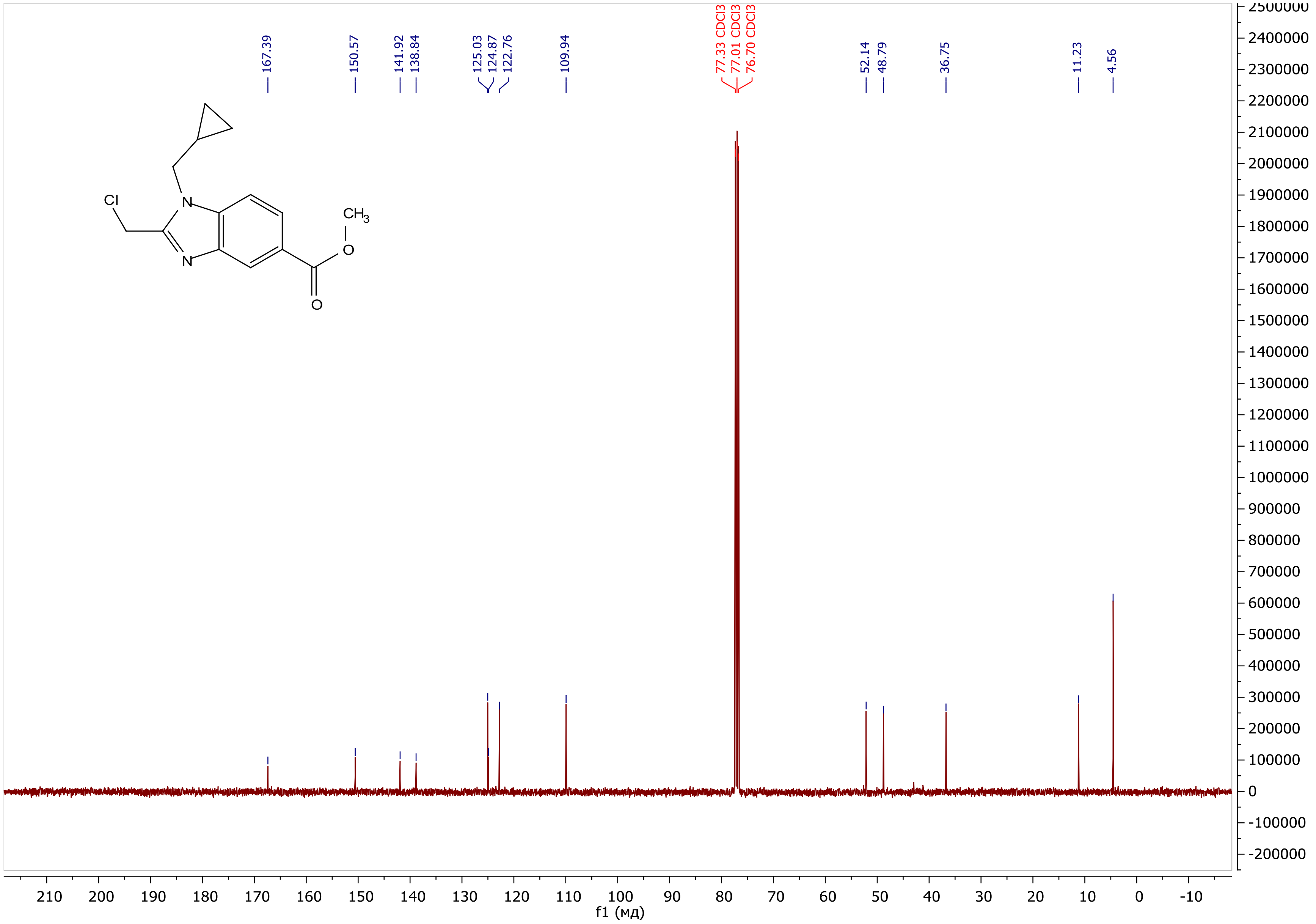

^{1}H NMR spectrum of compound **18g**

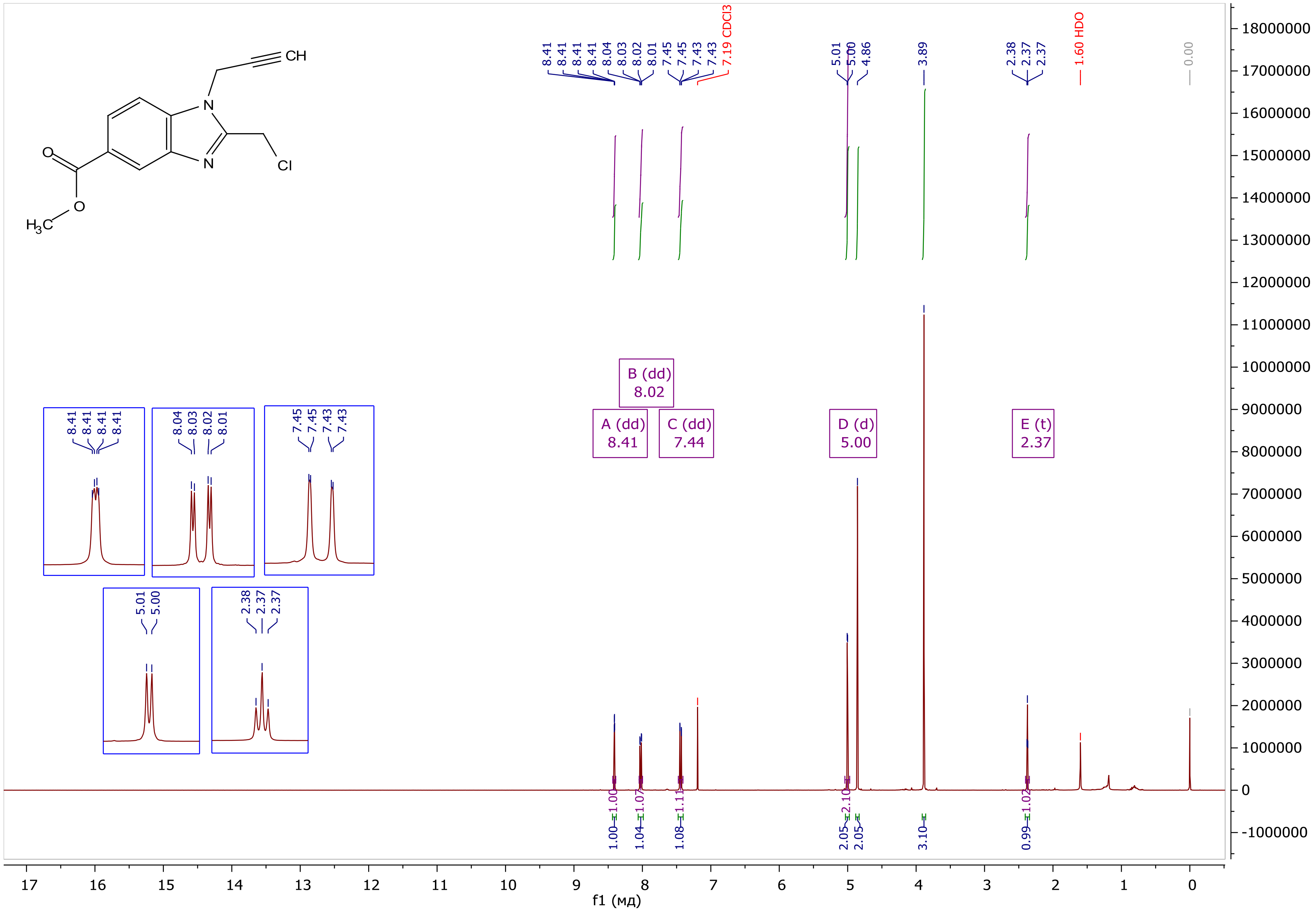

^{13}C NMR spectrum of compound **18g**

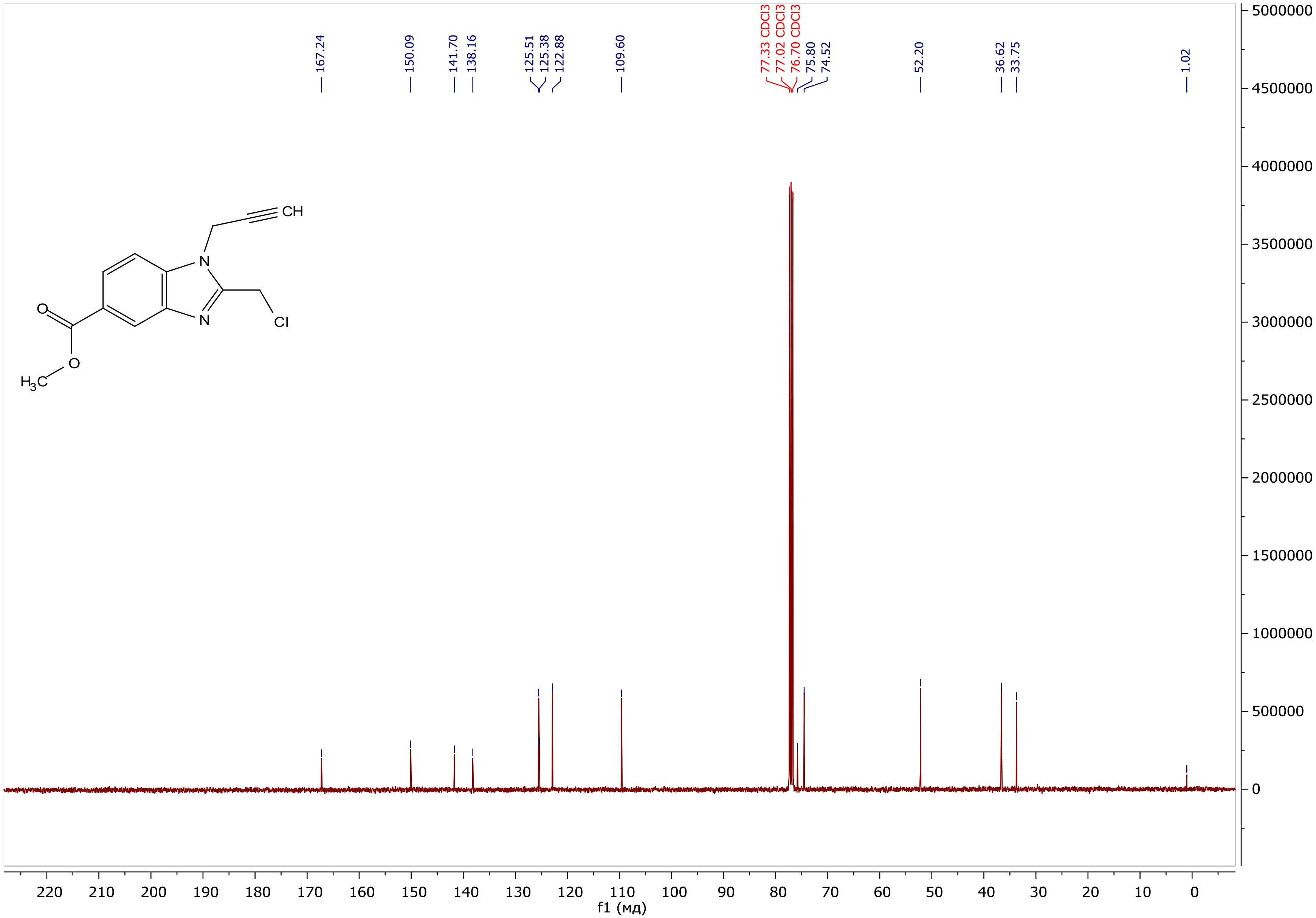

^{1}H NMR spectrum of compound **18h**

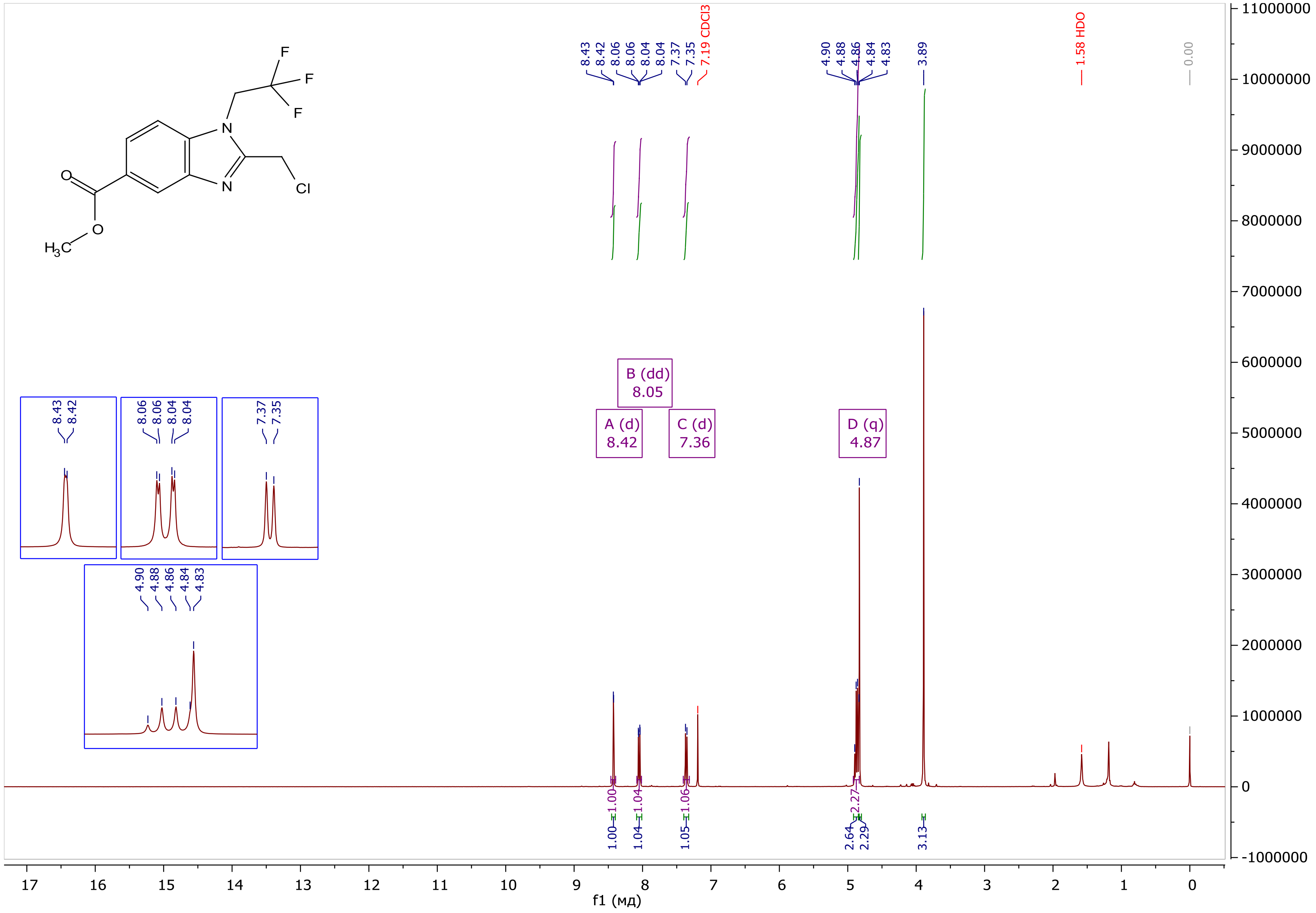

^{13}C NMR spectrum of compound **18h**

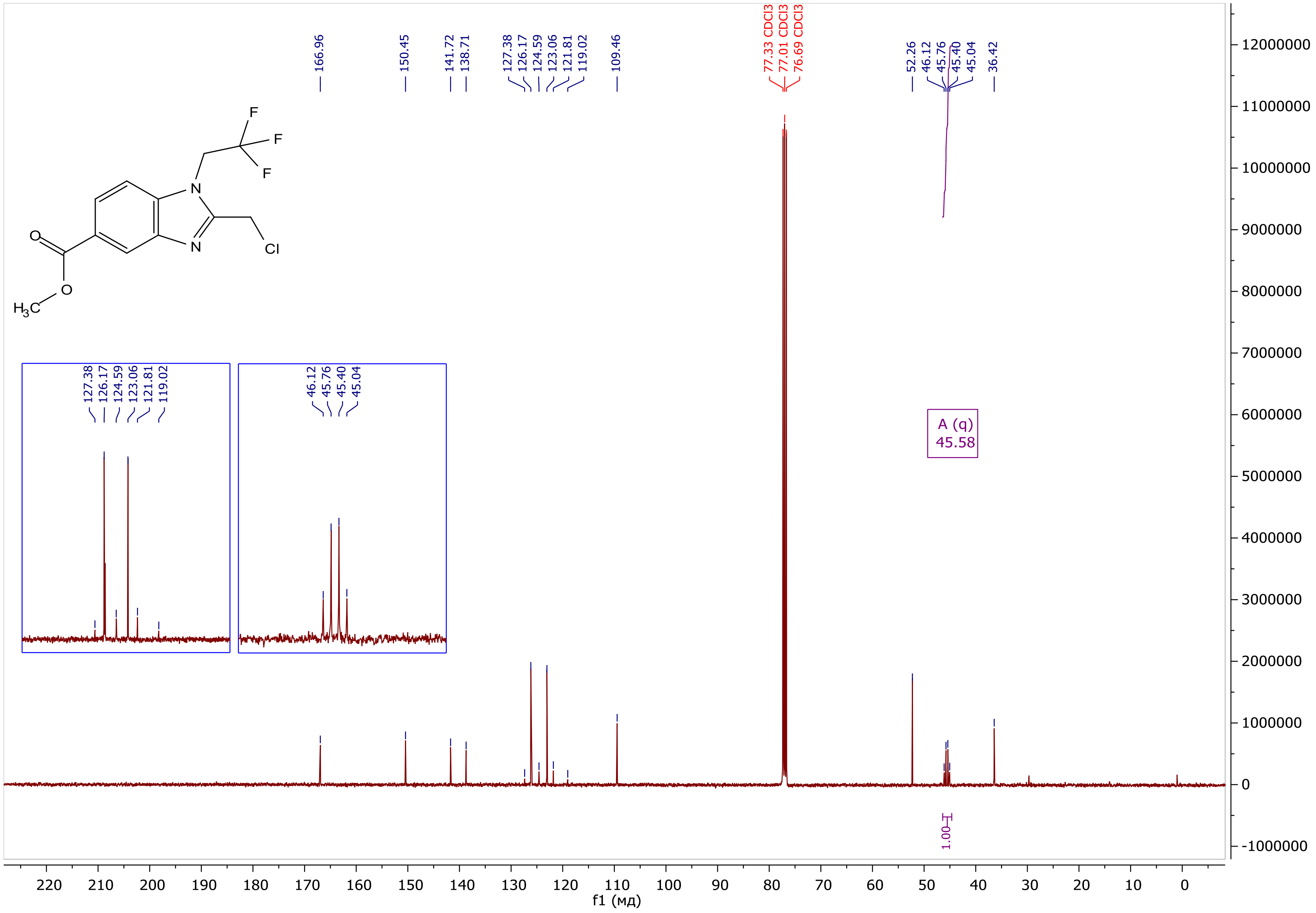

^{1}H NMR spectrum of compound **18i**

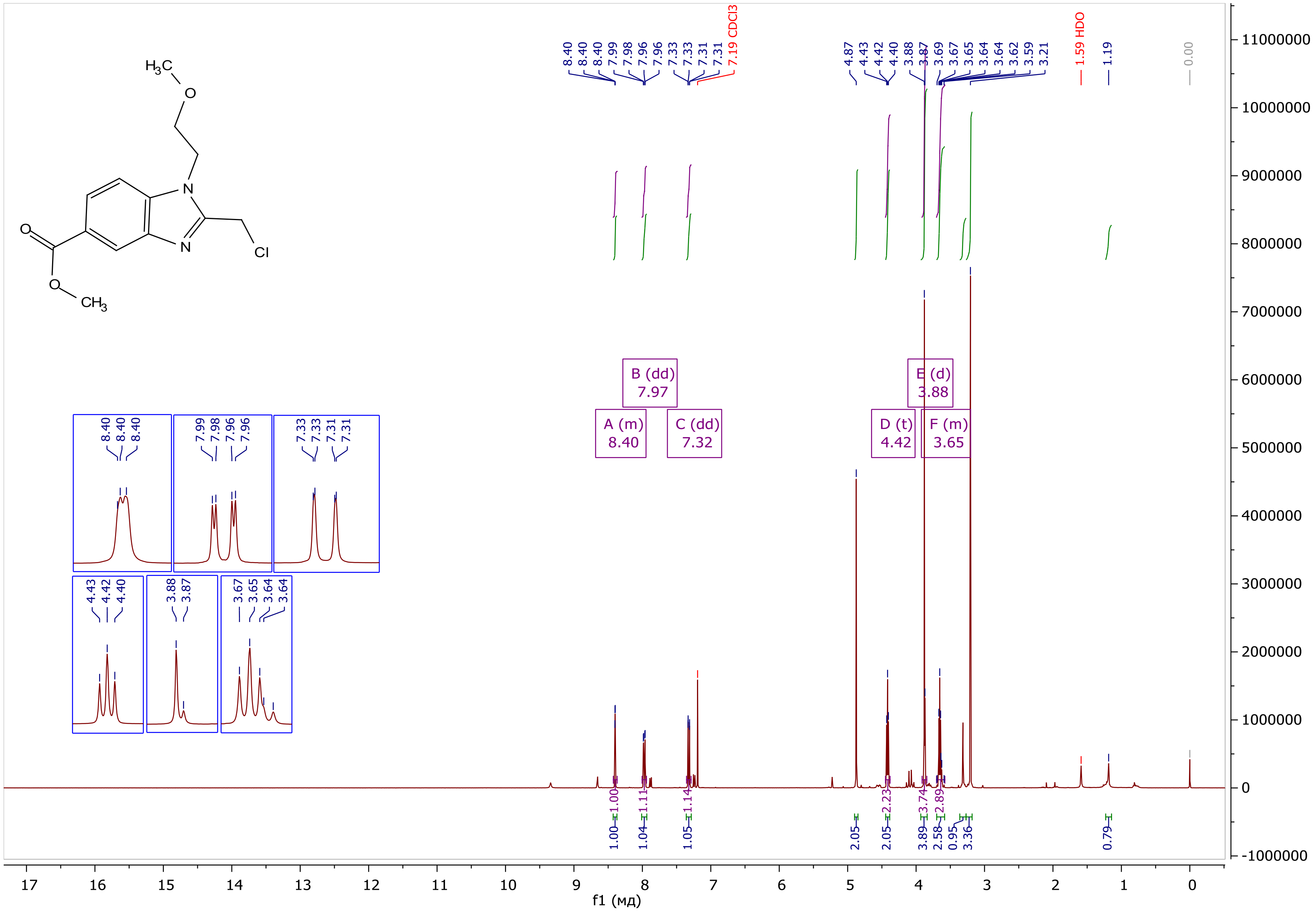

^{13}C NMR spectrum of compound **18i**

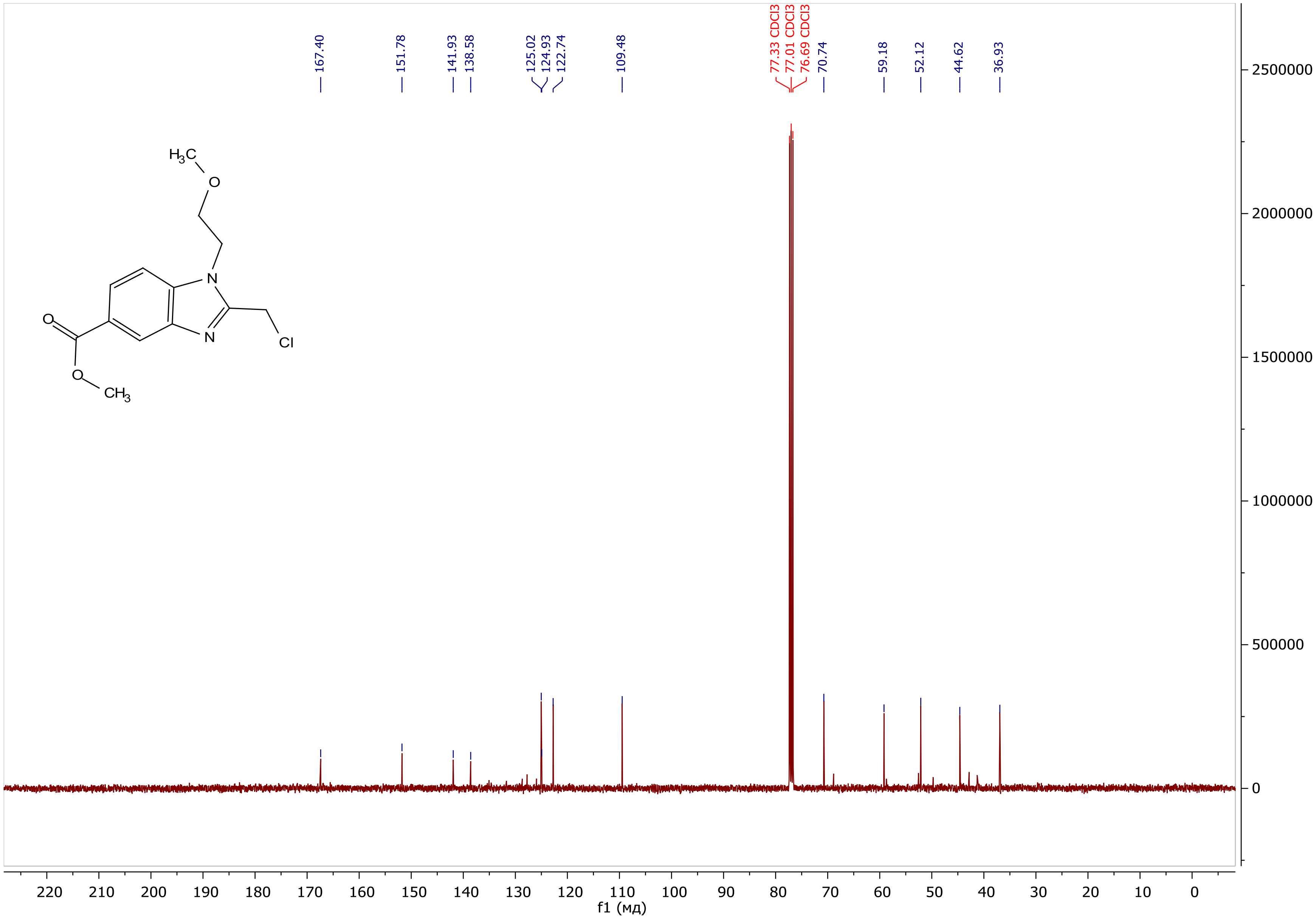

^{1}H NMR spectrum of compound **18j**

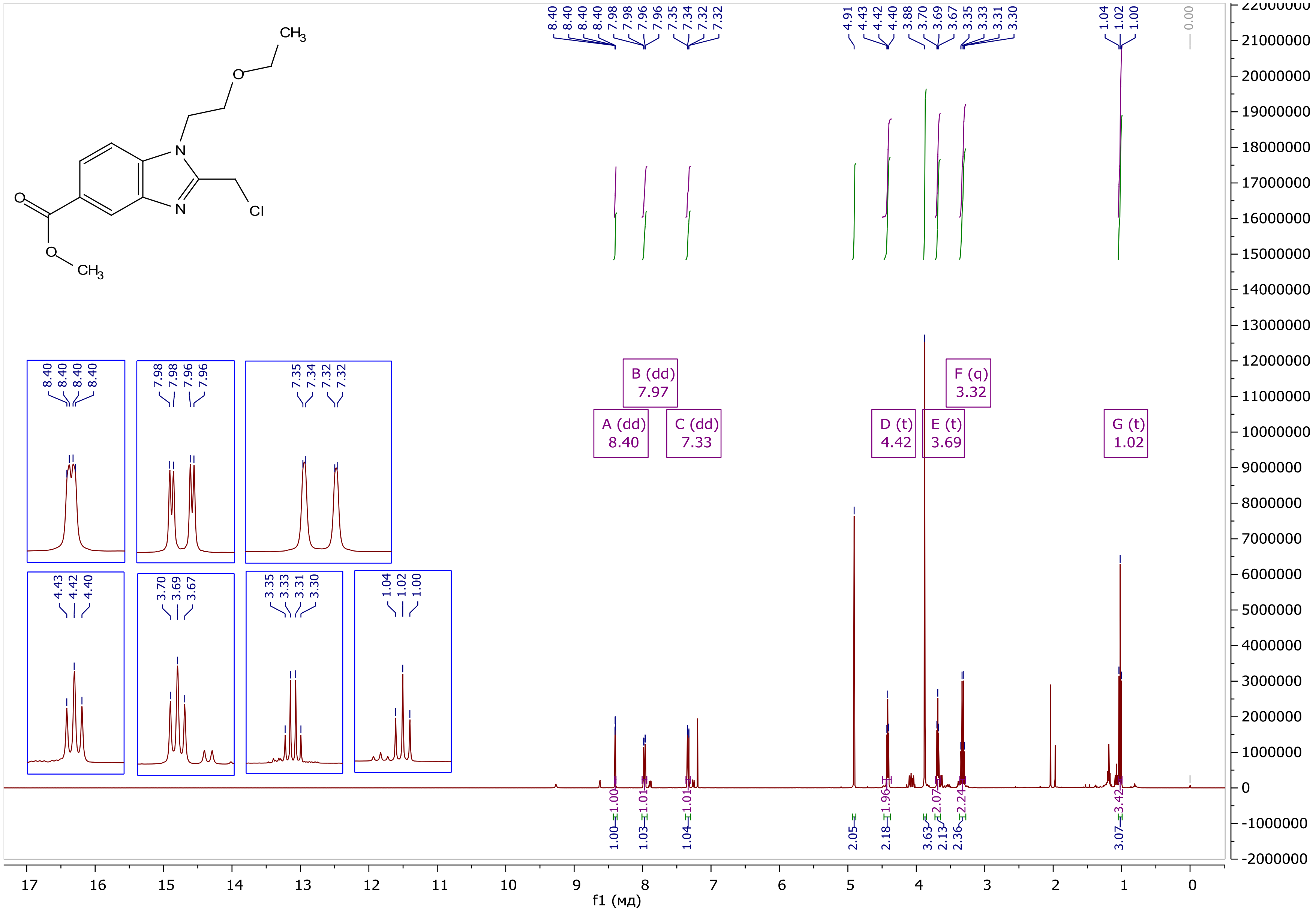

^{13}C NMR spectrum of compound **18j**

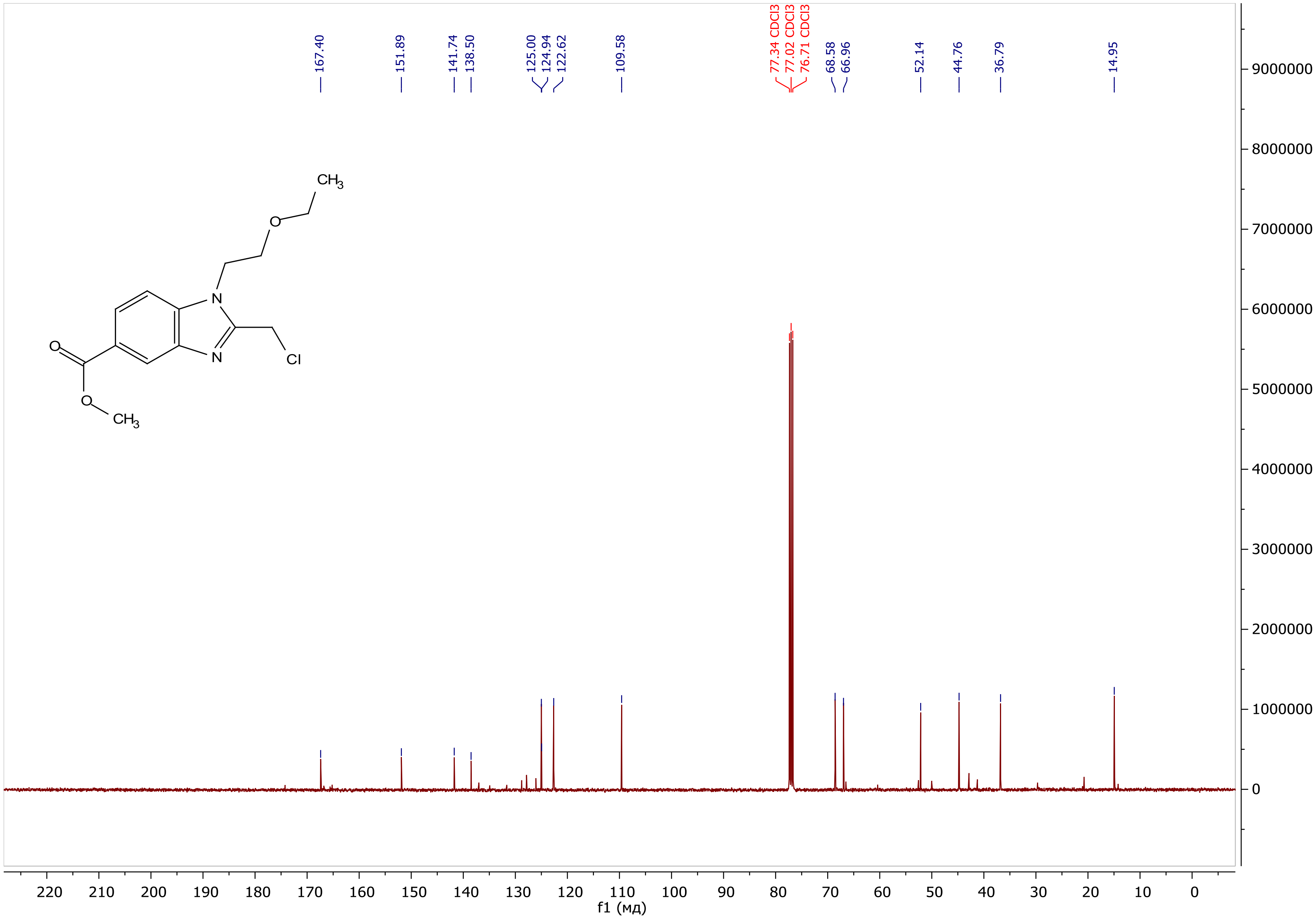

^{1}H NMR spectrum of compound **18k**

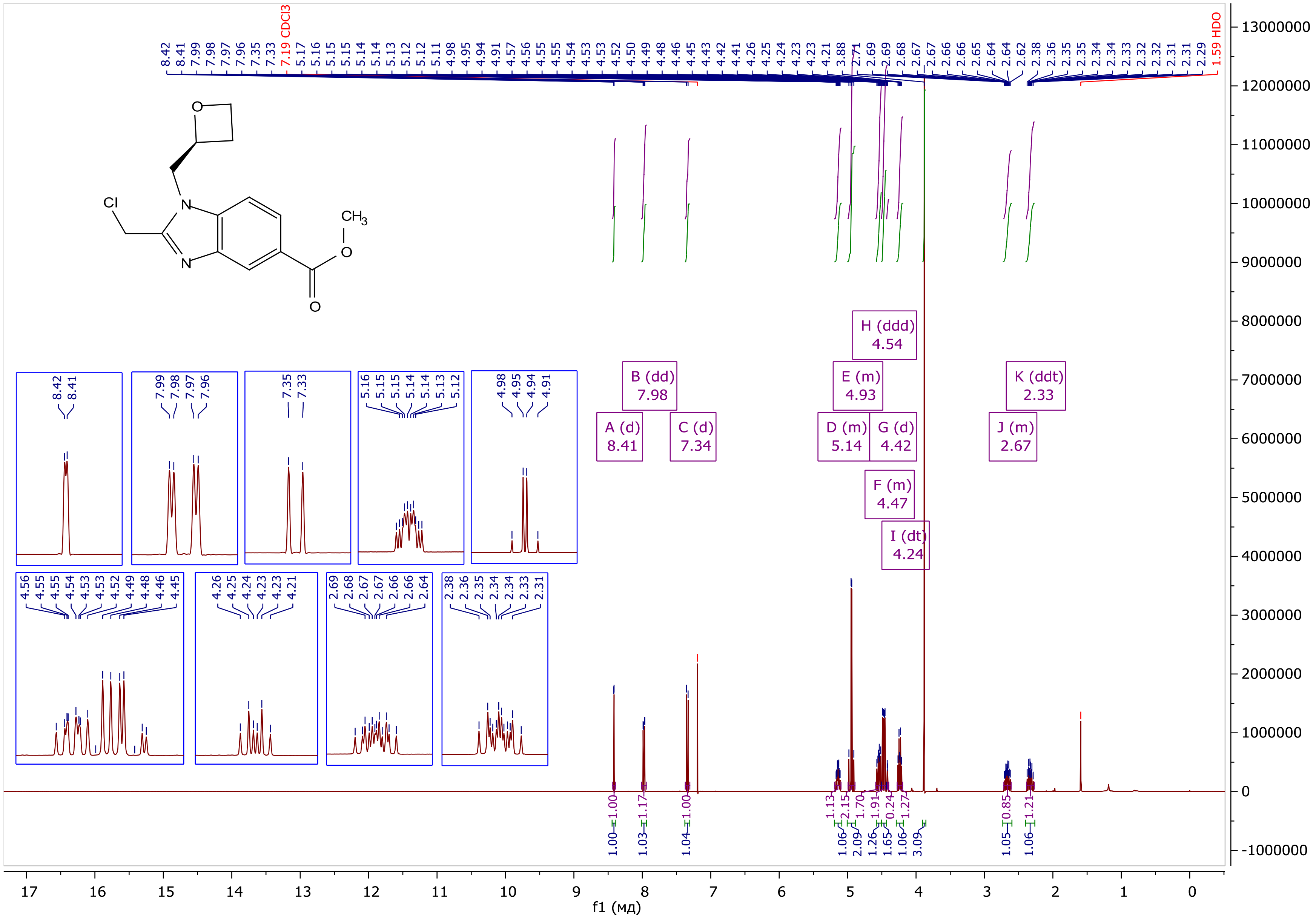

^{13}C NMR spectrum of compound **18k**

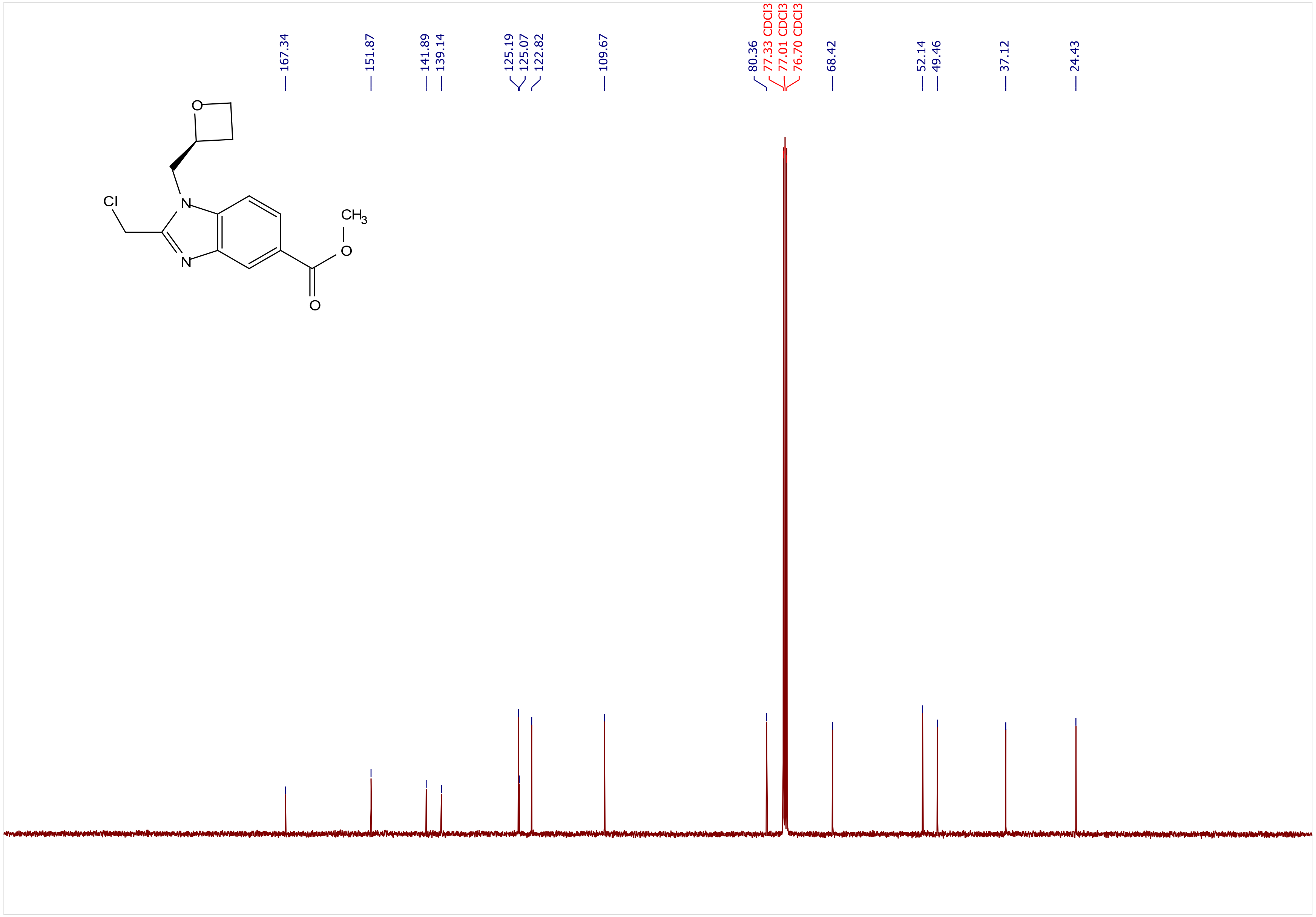

^{1}H NMR spectrum of compound **18l**

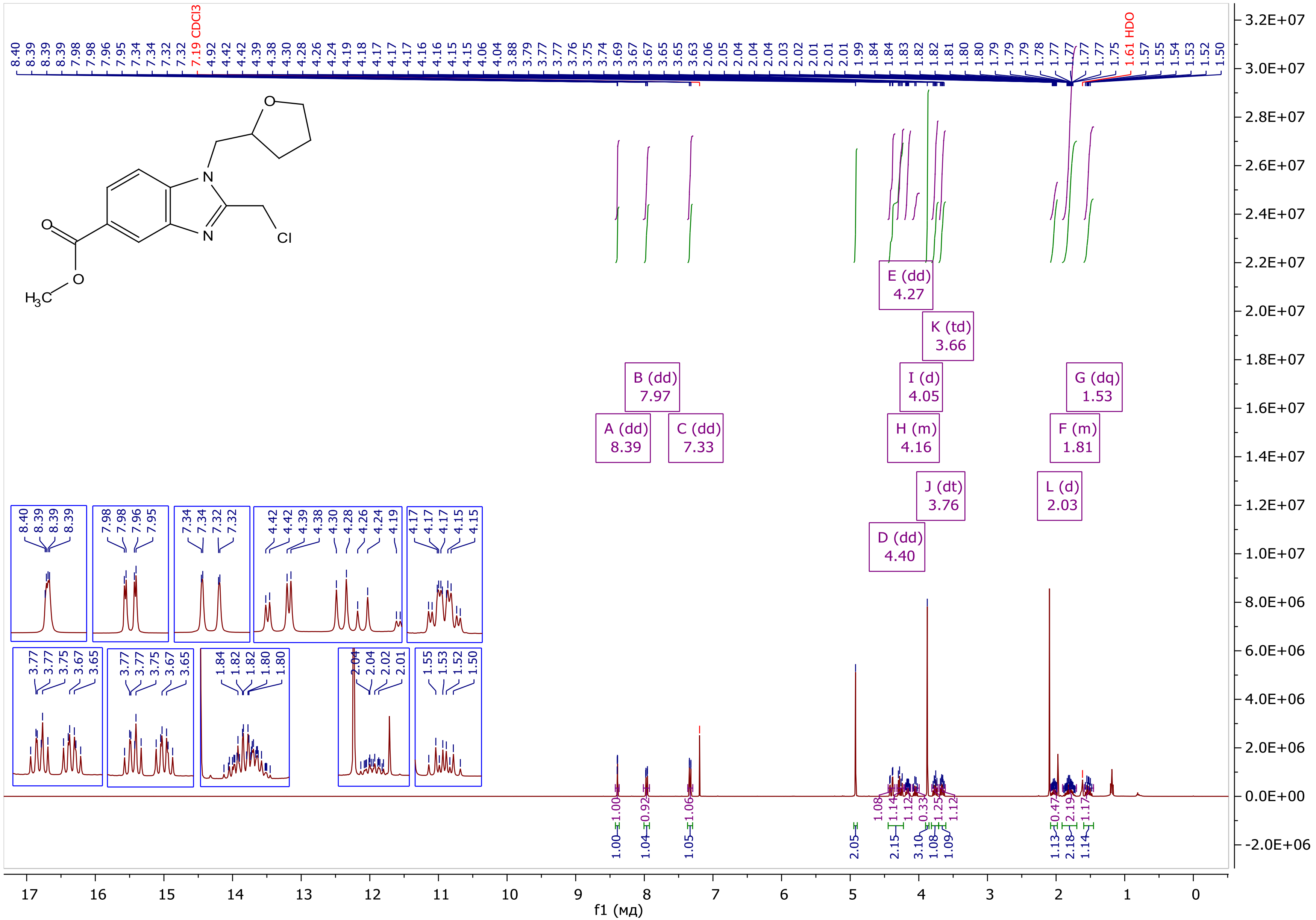

^{13}C NMR spectrum of compound **18l**

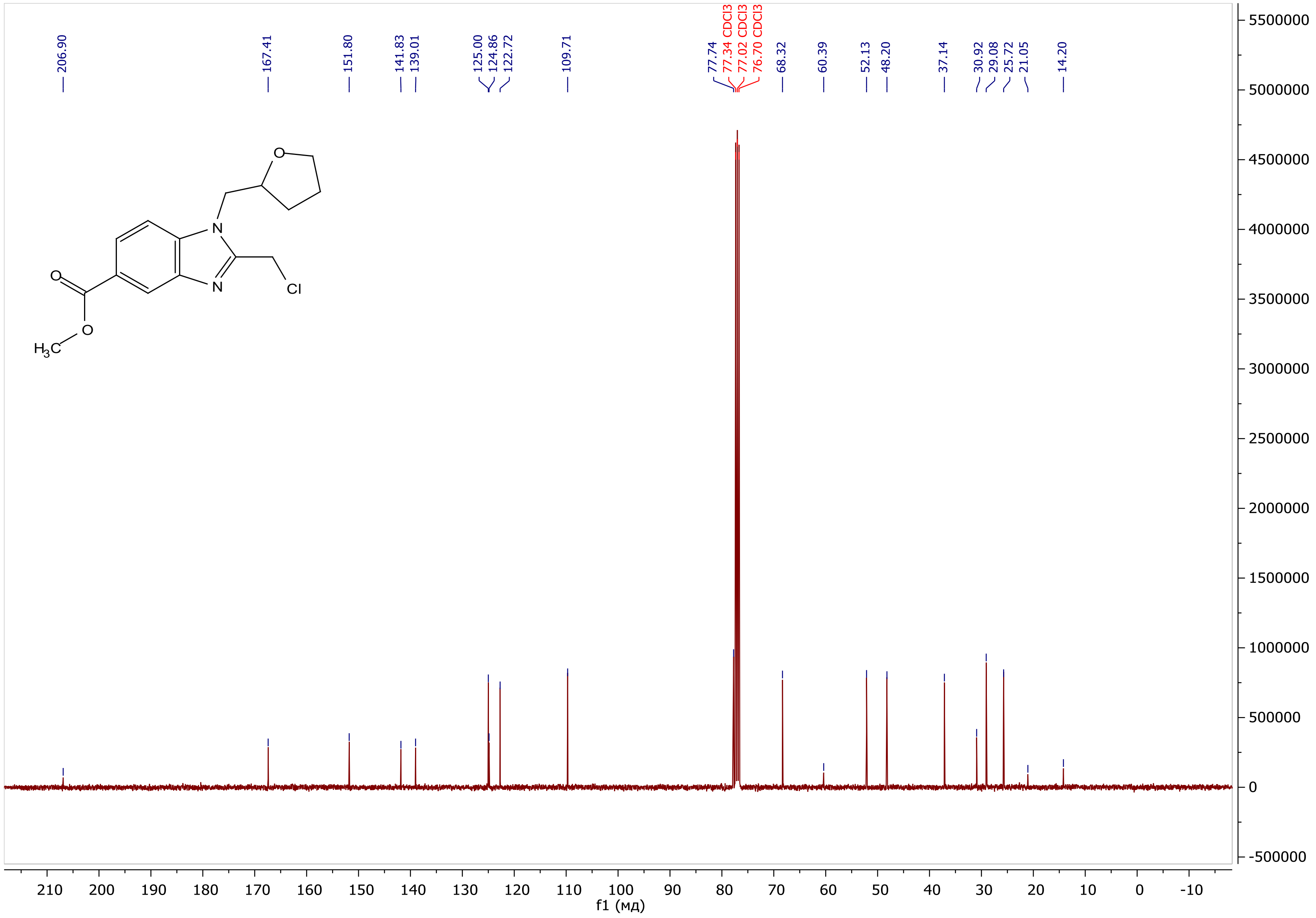

1,2,4-Oxadiazole derivatives 28a-c and intermediates of their synthesis 24a-c-27a-c

General procedure to synthesis of 4-substituted 3-nitrobenzoic acids **24a-c**. A weighted portion of the corresponding compound **16b**,**c**,**i** (1 eq) was dissolved in THF (5 ml) and an aqueous NaOH solution (1.5M, 20 eq) was added under vigorous stirring. The reaction mixture was refluxed under vigorous stirring for 2 h, the reaction was controlled by TLC (*n*-hexane:EtOAc = 4:1). After the reaction was completed, the mixture was cooled, and the organic layer was separated from the aqueous layer. The organic layer was washed twice with deionized water (2×10 ml), the combined aqueous layer was treated with EtOAc. Organic layers was combined, and an excess of H_2SO_4 aqueous solution (10%) was added to pH = 4-5. The precipitate formed was filtered and dried on the air to form the corresponding benzoic acid **24a-c** in a good yield.

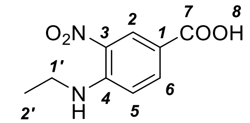

4-(Ethylamino-3-nitrobenzoic acid **24a**, yellow solid, 285 mg, 90%. **UPLC-MS** (**ESI-**): found *m/z* 255.1 [M - H + FA]$^-$; calculated [$C_9H_9N_2O_4$ + FA]$^-$ 255.1.

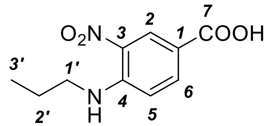

3-Nitro-4-(propylamino)benzoic acid **24b**, yellow solid, 365 мг, 95%. **UPLC-MS** (**ESI-**): found *m/z* 269.0 [M - H + FA]$^-$; calculated [$C_{10}H_{11}N_2O_4$ + FA]$^-$ 269.1.

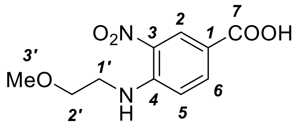

4-((2-Methoxyethyl)amino)-3-nitrobenzoic acid **24c***,* yellow solid, 360 mg, 95%. **UPLC-MS** (**ESI-**): found *m/z* 285.0 [M - H + FA]$^-$; calculated [$C_{10}H_{11}N_2O_5$ + FA]$^-$ 285.1.

General procedure to synthesis of O-acylamidoximes **25a-c**. To the suspension of the corresponding benzoic acid **24a-c** (1 eq) in DCM (15 ml) *N,N'*-carbonyldiimidazole (CDI, 1.2 eq) was added, and the formation of a yellow clear solution was observed. The reaction mixture was stirred at rt for 1.5 h, after which a weighted portion of acetoamidoxime (1.2 eq) was added. The reaction mixture was stirred at rt for 4-6 h, the reaction was controlled by TLC (DCM:MeOH = 9:1). After the reaction was completed, the solvent was evaporated on a rotary evaporator under reduced pressure, and the residue was purified by column chromatography on silica gel (DCM:MeOH (gradient from 100:0 to 9:1)). Fractions containing the target product were collected, the solvent was evaporated on a rotary evaporator under reduced pressure, and the residue was dried to form the corresponding *O*-acylamidoxime **25a-c** in good yield.

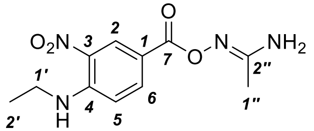

N-((4-(Ethylamino)-3-nitrobenzoyl)oxy)acetimidamide **25a**, yellow solid, 285 mg, 80%. **^{1}H NMR** (**400 MHz, DMSO-*d₆*, *δ ppm***): 8.69 (1H, *d*, *J* = 2.1 Hz, 2-CH), 8.44 (1H, *br.s.*, NH), 8.18-8.09 (1H, *m*, 6-CH), 7.09 (1H, *d*, *J* = 9.2 Hz, 5-CH), 6.51 (1H, *br.s.*, NH_2), 3.47 (2H, *qd*, *J* = 7.1, 5.6 Hz, 1'-CH_2), 1.81 (3H, *s*, 1''-CH_3), 1.24 (3H, *t*, *J* = 7.1 Hz, 2'-CH_3). **^{13}C NMR** (**101 MHz, DMSO-*d₆*, *δ ppm***): 162.8 (C-7), 156.7 (C-2''), 147.5 (C-3), 136.6 (C-6), 131.0 (C-1), 129.1 (C-2), 116.3 (C-4), 114.7 (C-5), 37.7 (C-1'), 16.9 (C-1''), 14.4 (C-2'). **UPLC-MS** (**ESI+**): found *m/z* 267.1 [M + H]$^+$; calculated $C_{11}H_{15}N_4O_4^+$ 267.1.

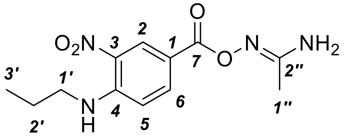

N-((3-Nitro-4-(propylamino)benzoyl)oxy)acetimidamide **25b**, yellow solid, 385 mg, 85%. **^{1}H NMR** (**400 MHz, $CDCl_3$, *δ ppm***): 8.76 (1H, *d*, *J* = 2.1 Hz, 2-CH), 8.31 (1H, *br.s.*, NH), 8.01 (1H, *ddd*, *J* = 9.0, 2.1, 0.7 Hz, 6-CH), 6.82 (1H, *d*, *J* = 9.1 Hz, 5-CH), 4.83 (1H, *br.s.*, NH_2), 3.27 (2H, *td*, *J* = 7.1, 5.2 Hz, 1'-CH_2), 1.99 (3H, *s*, 1''-CH_3), 1.72 (2H, *h*, *J* = 7.3 Hz, 2'-CH_2), 1.00 (3H, *t*, *J* = 7.4 Hz, 3'-CH_3). **^{13}C NMR** (**101 MHz, $CDCl_3$, *δ ppm***): 162.6 (C-7), 155.8 (C-2''), 147.9 (C-3), 136.5 (C-6), 131.0 (C-1), 129.0 (C-2), 116.2 (C-4), 113.8 (C-5), 45.0 (C-1'), 22.1 (C-2'), 17.2 (C-1''), 11.5 (C-3'). **UPLC-MS** (**ESI+**): found *m/z* 281.1 [M + H]$^+$; calculated $C_{12}H_{17}N_4O_4^+$ 281.1.

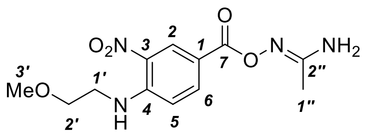

N-((4-((2-Methoxyethyl)amino)-3-nitrobenzoyl)oxy)acetimidamide **25c**, yellow solid, 375 mg, 85%. **^{1}H NMR (400 MHz, $CDCl_3$, *δ ppm*)**: 8.78 (1H, *d*, *J* = 2.1 Hz, 2-CH), 8.49 (1H, *br.s.*, NH), 8.01 (1H, *ddd*, *J* = 9.1, 2.1, 0.7 Hz, 6-CH), 6.84 (1H, *d*, *J* = 9.1 Hz, 5-CH), 5.06 (1H, *br.s.*, NH_2), 3.63 (2H, *dd*, *J* = 5.7, 4.8 Hz, 2'-CH_2), 3.50-3.46 (2H, *m*, 1'-CH_2), 3.37 (3H, *s*, 3'-CH_3), 1.97 (3H, *s*, 1"-CH_3). **^{13}C NMR (101 MHz, $CDCl_3$, *δ ppm*)**: 162.9 (C-7), 156.0 (C-2"), 147.8 (C-3), 136.4 (C-6), 131.3 (C-1), 129.0 (C-2), 116.3 (C-4), 113.9 (C-5), 70.1 (C-2'), 59.1 (C-3'), 43.0 (C-1'), 17.1 (C-1"). **UPLC-MS (ESI+)**: found *m/z* 297.1 $[M + H]^+$; calculated $C_{12}H_{17}N_4O_5^+$ 297.1.

General procedure to synthesis of 1,2,4-oxadiazole derivatives **26a-c**. The corresponding *O*-acylamidoxime **25a-c** (1 eq) was dissolved in THF (10 ml) under vigorous stirring. A TBAF solution in THF (1M, 0.5 eq) was added to the resulting solution. The reaction mixture was refluxed under vigorous stirring for 30 min, the reaction was controlled by TLC (DCM:MeOH = 9:1). After the reaction was completed, the solvent was evaporated on a rotary evaporator under reduced pressure; the residue was dissolved in EtOAc (20 ml), sequentially washed with deionized water (2×10 ml) and brine solution (1×10 ml). The organic layer was dried with anhydrous Na_2SO_4 under vigorous stirring for 1 h, after which the precipitate was filtered off, the solvent was evaporated to dryness, and the residue was dissolved in DCM (10 ml) and purified by column chromatography on silica gel (DCM:MeOH (gradient from 100:0 to 9:1)). Fractions containing the target product were collected, the solvent was evaporated on a rotary evaporator under reduced pressure, and the residue was dried to form the corresponding 1,2,4-oxadiazole derivative **26a-c** in good yield.

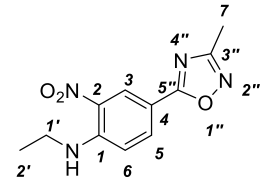

N-Ethyl-4-(3-methyl-1,2,4-oxadiazol-5-yl)-2-nitroaniline **26a**, orange solid, 240 mg, 90%. **^{1}H NMR (400 MHz, $CDCl_3$, *δ ppm*)**: 8.80 (1H, *d*, *J* = 2.1 Hz, 3-CH), 8.16 (1H, *br.s.*, NH), 7.94 (1H, *ddd*, *J* = 9.0, 2.1, 0.7 Hz, 5-CH), 6.80 (1H, *d*, *J* = 9.1 Hz, 6-CH), 3.28 (2H, *qd*, *J* = 7.2, 5.1 Hz, 1'-CH_2), 2.29 (3H, *s*, 7-CH_3), 1.26 (3H, *t*, *J* = 7.2 Hz, 2'-CH_3). **^{13}C NMR (101 MHz, $CDCl_3$, *δ ppm*)**: 174.0 (C-5"), 167.7 (C-3"), 147.3 (C-2), 134.5 (C-5), 131.6 (C-4), 127.6 (C-3), 114.4 (C-6), 111.3 (C-1), 38.0 (C-1'), 14.2 (C-2'), 11.7 (C-7). **UPLC-MS (ESI+)**: found *m/z* 249.1 $[M + H]^+$; calculated $C_{11}H_{13}N_4O_3^+$ 249.1

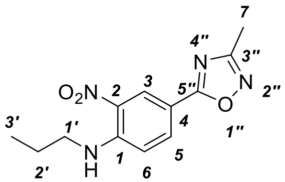

4-(3-Methyl-1,2,4-oxadiazol-5-yl)-2-nitro-N-propylaniline **26b**, orange solid, 340 mg, 95%. **^{1}H NMR (400 MHz, $CDCl_3$, *δ ppm*)**: 8.89 (1H, *d*, *J* = 2.1 Hz, 3-CH), 8.34 (1H, *br.s.*, NH), 8.03 (1H, *ddd*, *J* = 9.1, 2.1, 0.7 Hz, 5-CH), 6.90 (1H, *d*, *J* = 9.1 Hz, 6-CH), 3.29 (2H, *td*, *J* = 7.1, 5.2 Hz, 1'-CH_2), 2.38 (3H, *s*, 7-CH_3), 1.74 (2H, *h*, *J* = 7.3 Hz, 2'-CH_2), 1.02 (3H, *t*, *J* = 7.4 Hz, 3'-CH_3). **^{13}C NMR (101 MHz, $CDCl_3$, *δ ppm*)**: 174.0 (C-5"), 167.7 (C-3"), 147.4 (C-2), 134.5 (C-3), 131.6 (C-4), 127.7 (C-5), 114.5 (C-6), 111.2 (C-1), 45.0 (C-1'), 22.2 (C-2'), 11.7 (C-7), 11.5 (C-3'). **UPLC-MS (ESI+)**: found *m/z* 263.1 $[M + H]^+$; calculated $C_{12}H_{15}N_4O_3^+$ 263.1.

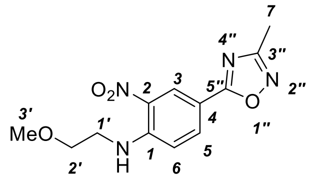

N-(2-Methoxyethyl)-4-(3-methyl-1,2,4-oxadiazol-5-yl)-2-nitroaniline **26c**, orange solid, 315 mg, 90%. **^{1}H NMR (400 MHz, $CDCl_3$, *δ ppm*)**: 8.89 (1H, *d*, *J* = 2.1 Hz, 3-CH), 8.51 (1H, *br.s.*, NH), 8.03 (1H, *ddd*, *J* = 9.0, 2.1, 0.7 Hz, 5-CH), 6.92 (1H, *d*, *J* = 9.1 Hz, 6-CH), 3.64 (2H, *dd*, *J* = 5.7, 4.8 Hz, 2'-CH_2), 3.50 (2H, *q*, *J* = 5.2 Hz, 1'-CH_2), 3.38 (3H, *s*, 3'-CH_3), 2.38 (3H, *s*, 7-CH_3). **^{13}C NMR (101 MHz, $CDCl_3$, *δ ppm*)**: 174.0 (C-5"), 167.7 (C-3"), 147.3 (C-2), 134.5 (C-5), 131.9 (C-4), 127.6 (C-3), 114.5 (C-6), 111.5 (C-1), 70.1 (C-2'), 59.2 (C-3'), 43.0 (C-1'), 11.7 (C-1"). **UPLC-MS (ESI+)**: found *m/z* 279.1 $[M + H]^+$; calculated $C_{12}H_{15}N_4O_4^+$ 279.1.

General procedure to synthesis of 1,2,4-oxadiazolyl-substituted benzene-1,2-diamines **27a-c**. Compounds **27a-c** were obtained from the corresponding 1,2,4-oxadiazole derivative **22a-c**, respectively, as described above for compounds **17f-l**.

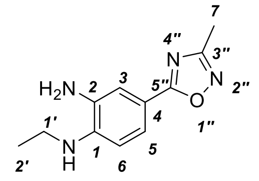

N-ethyl-4-(3-methyl-1,2,4-oxadiazol-5-yl)benzene-1,2-diamine **27a**, orange solid, 185 mg, 90%. **^{1}H NMR (400 MHz, $CDCl_3$, *δ ppm*)**: 7.56 (1H, *dd*, *J* = 8.3, 2.0 Hz, 5-CH), 7.37 (1H, *d*, *J* = 2.0 Hz, 3-CH), 6.59 (1H, *d*, *J* = 8.4 Hz, 6-CH), 3.17 (2H, *q*, *J* = 7.1 Hz, 1'-CH_2), 2.35 (3H, *s*, 7-CH_3), 1.26 (3H, *t*, *J* = 7.1 Hz, 2'-CH_3). **^{13}C NMR (101 MHz, $CDCl_3$, *δ ppm*)**: 176.1 (C-5"), 167.3 (C-3"), 142.9 (C-2), 132.8 (C-4), 122.6 (C-5), 116.1 (C-3), 112.8 (C-1), 110.0 (C-6), 38.2 (C-1'), 14.7 (C-2'), 11.7 (C-7). **UPLC-MS (ESI+)**: found *m/z* 219.1 $[M + H]^+$; calculated $C_{11}H_{15}N_4O^+$ 219.1.

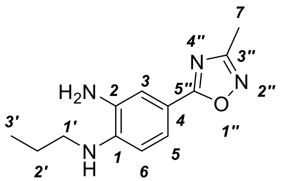

4-(3-methyl-1,2,4-oxadiazol-5-yl)-N-propylbenzene-1,2-diamine **27b**, orange solid, 270 mg, 90%. **^{1}H NMR (400 MHz, CDCl$_3$, *δ ppm*)**: 7.56 (1H, *dd*, *J* = 8.3, 2.0 Hz, 5-CH), 7.38 (1H, *d*, *J* = 2.0 Hz, 3-CH), 6.59 (1H, *d*, *J* = 8.3 Hz, 6-CH), 3.10 (2H, *t*, *J* = 7.1 Hz, 1'-CH$_2$), 2.35 (3H, *s*, 7-CH$_3$), 1.65 (2H, *h*, *J* = 7.3 Hz, 2'-CH$_2$), 0.98 (3H, *t*, *J* = 7.4 Hz, 3'-CH$_3$). **^{13}C NMR (101 MHz, CDCl$_3$, *δ ppm*)**: 176.1 (C-5''), 167.3 (C-3''), 143.0 (C-2), 132.7 (C-4), 122.7 (C-5), 116.2 (C-3), 112.7 (C-1), 110.0 (C-6), 45.5 (C-1'), 22.6 (C-2'), 11.7 (C-7), 11.7 (C-3'). **UPLC-MS (ESI**+): found *m/z* 233.1 [M + H]$^+$; calculated $C_{12}H_{17}N_4O^+$ 233.1.

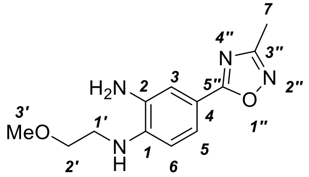

N-(2-Methoxyethyl)-4-(3-methyl-1,2,4-oxadiazol-5-yl)benzene-1,2-diamine **27c**, orange solid, 235 mg, 85%. **^{1}H NMR (400 MHz, CDCl$_3$, *δ ppm*)**: 7.55 (1H, *dd*, *J* = 8.3, 2.0 Hz, 5-CH), 7.37 (1H, *d*, *J* = 2.0 Hz, 3-CH), 6.60 (1H, *d*, *J* = 8.3 Hz, 6-CH), 3.68-3.58 (4H, *m*, 2'-CH$_2$, 1'-CH$_2$), 3.34 (3H, *s*, 3'-CH$_3$), 2.35 (3H, *s*, 7-CH$_3$). **^{13}C NMR (101 MHz, CDCl$_3$, *δ ppm*)**: 173.5 (C-5''), 167.3 (C-3''), 143.1 (C-2), 133.4 (C-4), 122.3 (C-5), 116.3 (C-3), 113.3 (C-1), 109.6 (C-6), 70.7 (C-2'), 58.8 (C-3'), 43.2 (C-1'), 11.7 (C-7). **UPLC-MS (ESI**+): found *m/z* 249.1 [M + H]$^+$; calculated $C_{12}H_{17}N_4O_2^+$ 249.1.

General procedure to synthesis of 5-(1H-benzo[d]imidazol-5-yl)-3-methyl-1,2,4-oxadiazole derivatives **28a-c**. Compounds **28a-c** were obtained from the corresponding 1,2,4-oxadiazolyl-substituted benzene-1,2-diamine **27a-c**, respectively, as described above for compounds **18a-f**, **18i-j**.

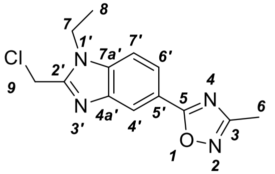

5-(2-(Chloromethyl)-1-ethyl-1H-benzo[d]imidazol-5-yl)-3-methyl-1,2,4-oxadiazole **28a**, white solid, 110 mg, 50%. **^{1}H NMR (400 MHz, CDCl$_3$, *δ ppm*)**: 8.46 (1H, *dd*, *J* = 1.6, 0.6 Hz, 4'-CH), 8.04 (1H, *dd*, *J* = 8.5, 1.6 Hz, 6'-CH), 7.43 (1H, *dd*, *J* = 8.5, 0.7 Hz, 7'-CH), 4.79 (2H, *s*, 9-CH$_2$), 4.30 (2H, *q*, *J* = 7.3 Hz, 7-CH$_2$), 2.42 (3H, *s*, 6-CH$_3$), 1.49 (3H, *t*, *J* = 7.3 Hz, 8-CH$_3$). **^{13}C NMR (101 MHz, CDCl$_3$, *δ ppm*)**: 175.9 (C-5), 167.8 (C-3), 150.8 (C-2'), 142.4 (C-4a'), 138.0 (C-7a'), 123.5 (C-6'), 121.0 (C-4'), 118.9 (C-5'), 110.5 (C-7'), 39.5 (C-7), 36.5 (C-9), 15.1 (C-8), 11.8 (C-6). **HRMS (ESI+)**: found *m/z* 277.0902 [M + H]$^+$; calculated $C_{13}H_{14}{}^{35}ClN_4O^+$ 277.0778

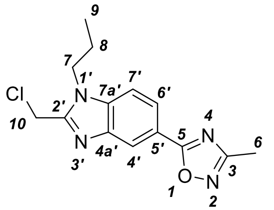

5-(2-(Chloromethyl)-1-propyl-1H-benzo[d]imidazol-5-yl)-3-methyl-1,2,4-oxadiazole **28b**, white solid, 135 mg, 40%. **^{1}H NMR (400 MHz, CDCl$_3$, *δ ppm*)**: 8.46 (1H, *d*, *J* = 1.4 Hz, 4'-CH), 8.02 (1H, *dd*, *J* = 8.6, 1.6 Hz, 6'-CH), 7.42 (1H, *dd*, *J* = 8.6, 0.6 Hz, 7'-CH), 4.79 (2H, *s*, 10-CH$_2$), 4.23-4.13 (2H, *m*, 7-CH$_2$), 2.41 (3H, *s*, 6-CH$_3$), 1.90 (2H, *h*, *J* = 7.4 Hz, 8-CH$_2$), 0.98 (3H, *t*, *J* = 7.4 Hz, 9-CH$_3$). **^{13}C NMR (101 MHz, CDCl$_3$, *δ ppm*)**: 175.9 (C-5), 167.8 (C-3), 151.0 (C-2'), 142.3 (C-4a'), 138.4 (C-7a'), 123.4 (C-6'), 121.0 (C-4'), 118.9 (C-5'), 110.7 (C-7'), 46.2 (C-7), 36.5 (C-10), 23.2 (C-8), 11.8 (C-6), 11.4 (C-9). **HRMS (ESI+)**: found *m/z* 291.1056 [M + H]$^+$; calculated $C_{14}H_{16}{}^{35}ClN_4O^+$ 291.0934.

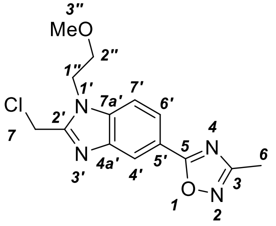

5-(2-(Chloromethyl)-1-(2-methoxyethyl)-1H-benzo[d]imidazol-5-yl)-3-methyl-1,2,4-oxadiazole **28c**, white solid, 145 mg, 50%. **^{1}H NMR (400 MHz, CDCl$_3$, *δ ppm*)**: 8.46 (1H, *d*, *J* = 1.5 Hz, 4'-CH), 8.02 (1H, *dd*, *J* = 8.6, 1.4 Hz, 6'-CH), 7.43 (1H, *d*, *J* = 8.6 Hz, 7'-CH), 4.89 (2H, *s*, 7-CH$_2$), 4.44 (2H, *t*, *J* = 5.1 Hz, 1''-CH$_2$), 4.16-4.05 (2H, *m*, 2''-CH$_2$), 3.22 (3H, *s*, 3''-CH$_3$), 2.42 (3H, *s*, 6-CH$_3$). **^{13}C NMR (101 MHz, CDCl$_3$, *δ ppm*)**: 175.8 (C-5), 167.7 (C-3), 152.1 (C-2'), 142.2 (C-4a'), 138.3 (C-7a'), 123.4 (C-6'), 120.8 (C-4'), 118.8 (C-5'), 110.5 (C-7'), 70.7 (C-2''), 59.1 (C-3''), 44.6 (C-1''), 36.7 (C-7), 11.7 (C-6). **UPLC-MS (ESI**+): found *m/z* 307.0 [M + H]$^+$; calculated $C_{14}H_{16}{}^{35}ClN_4O_2^+$ 307.1.

^{1}H NMR spectrum of compound **25a**

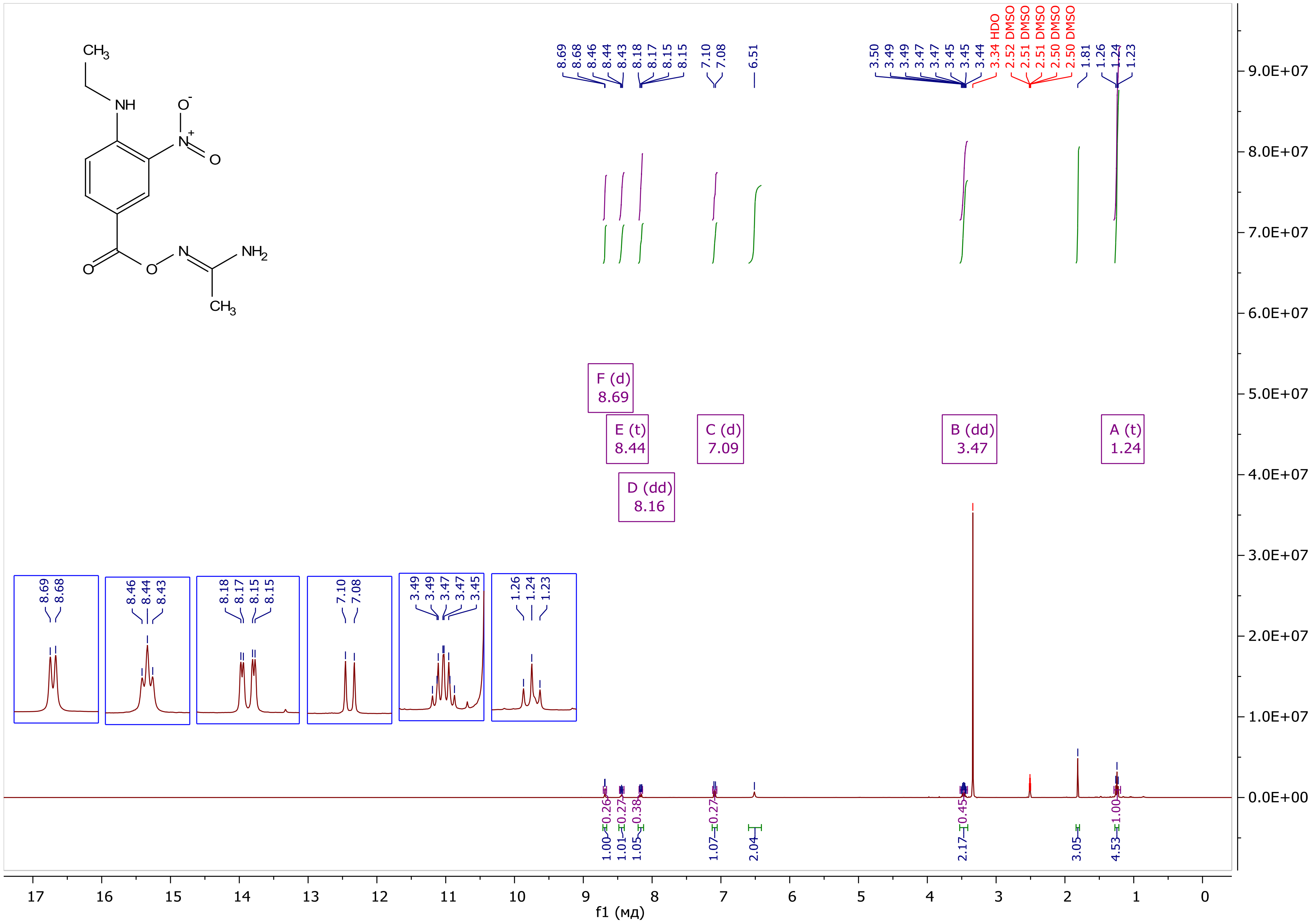

^{13}C NMR spectrum of compound **25a**

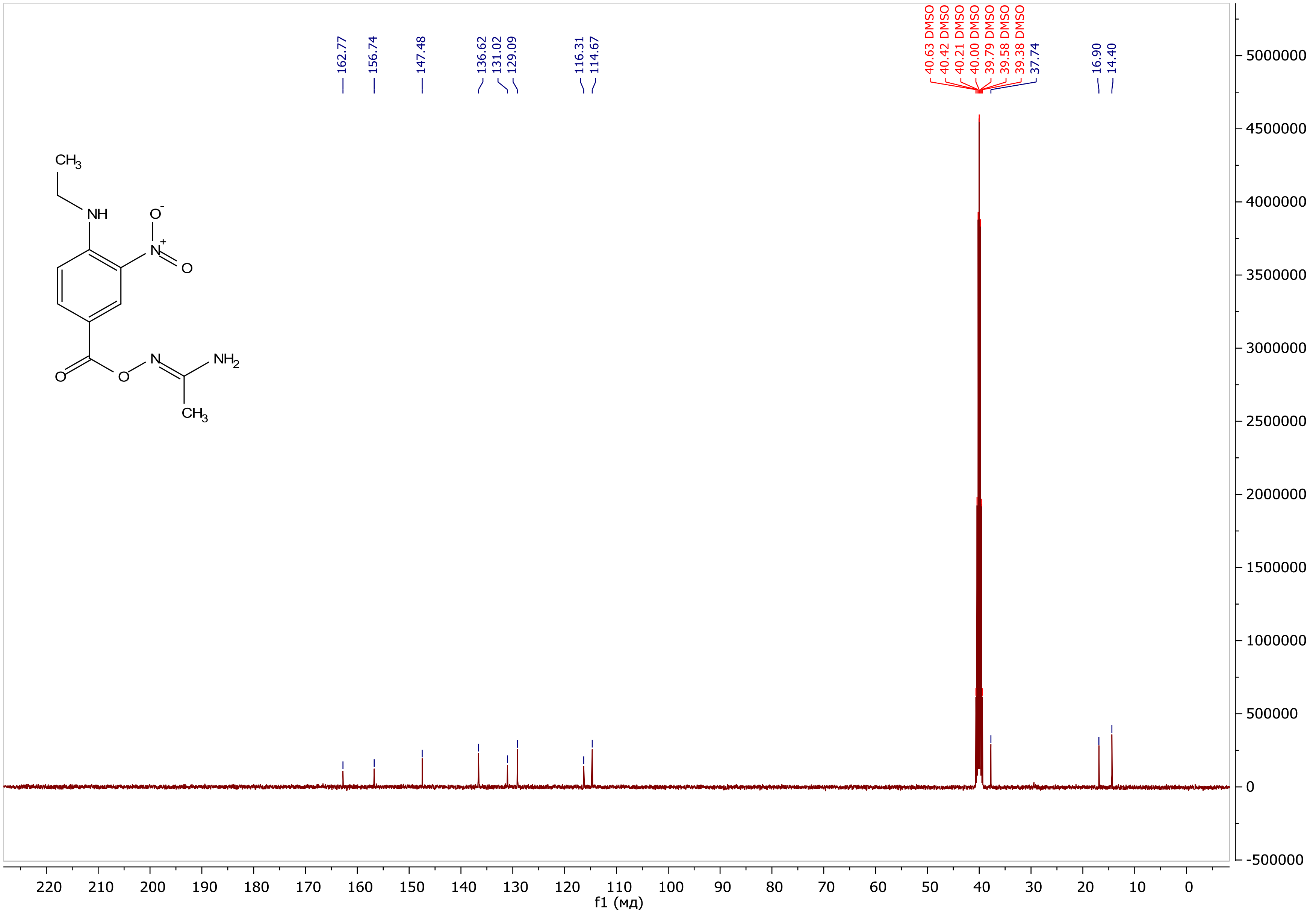

^{1}H NMR spectrum of compound **25b**

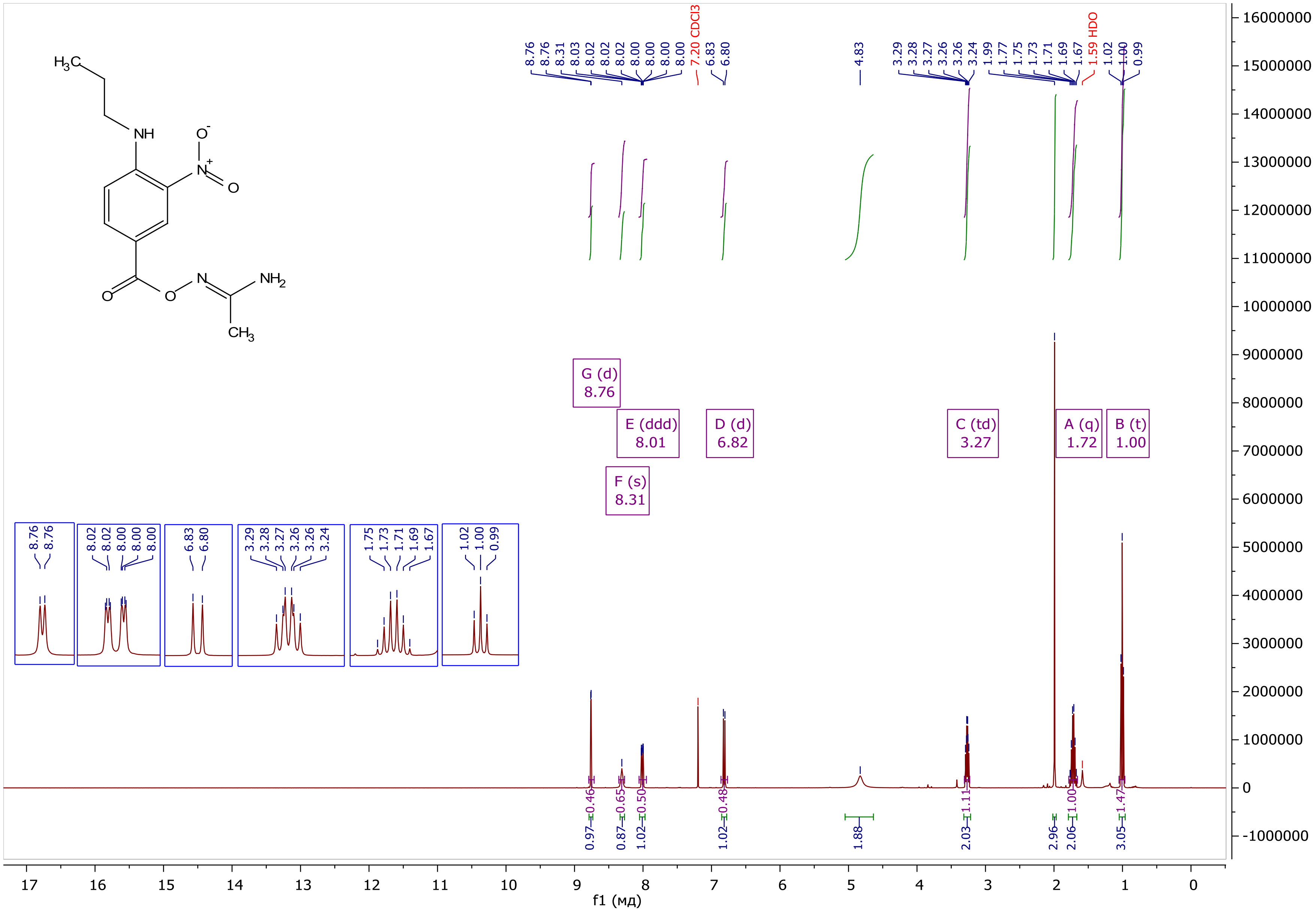

^{13}C NMR spectrum of compound **25b**

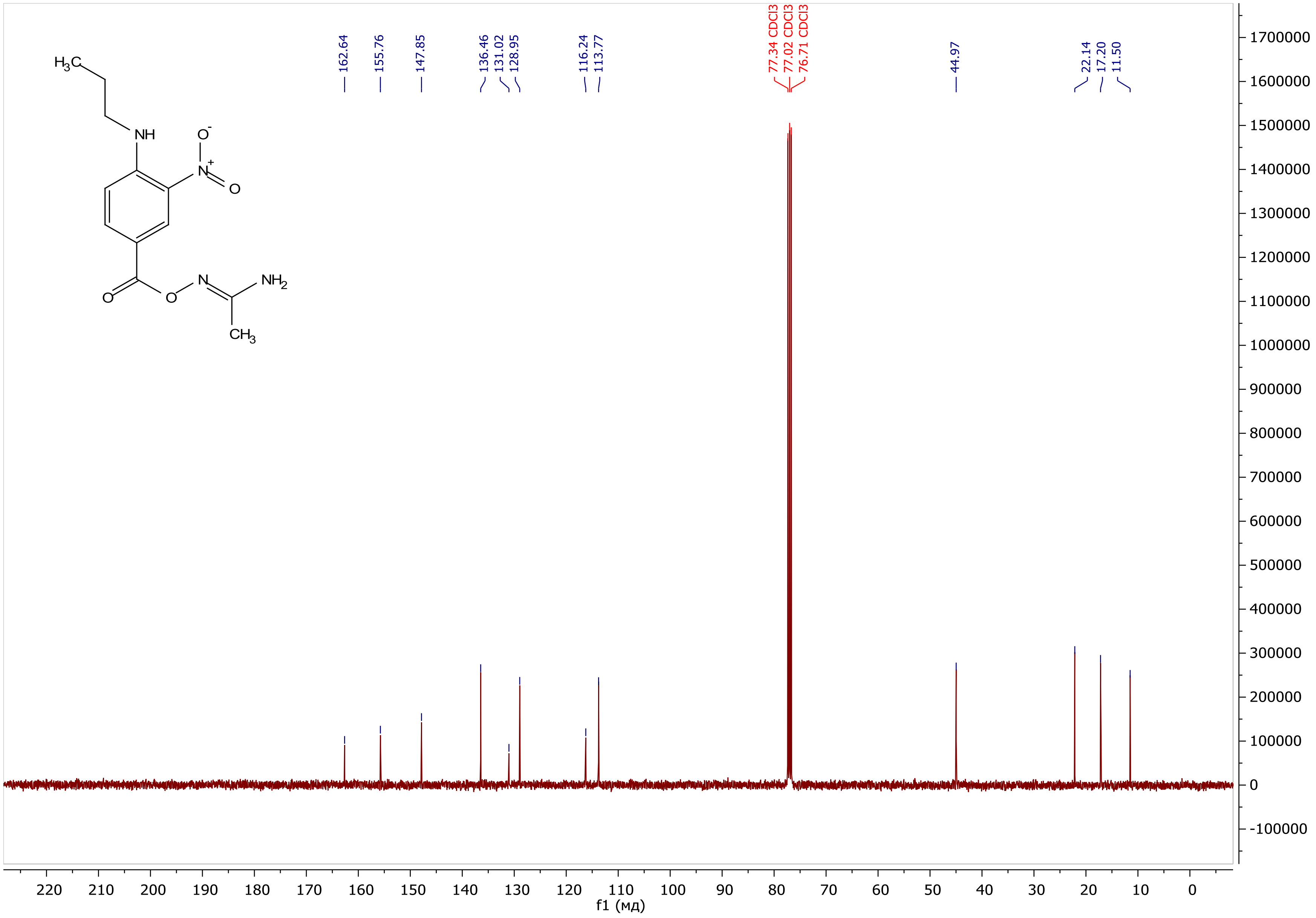

^{1}H NMR spectrum of compound **25c**

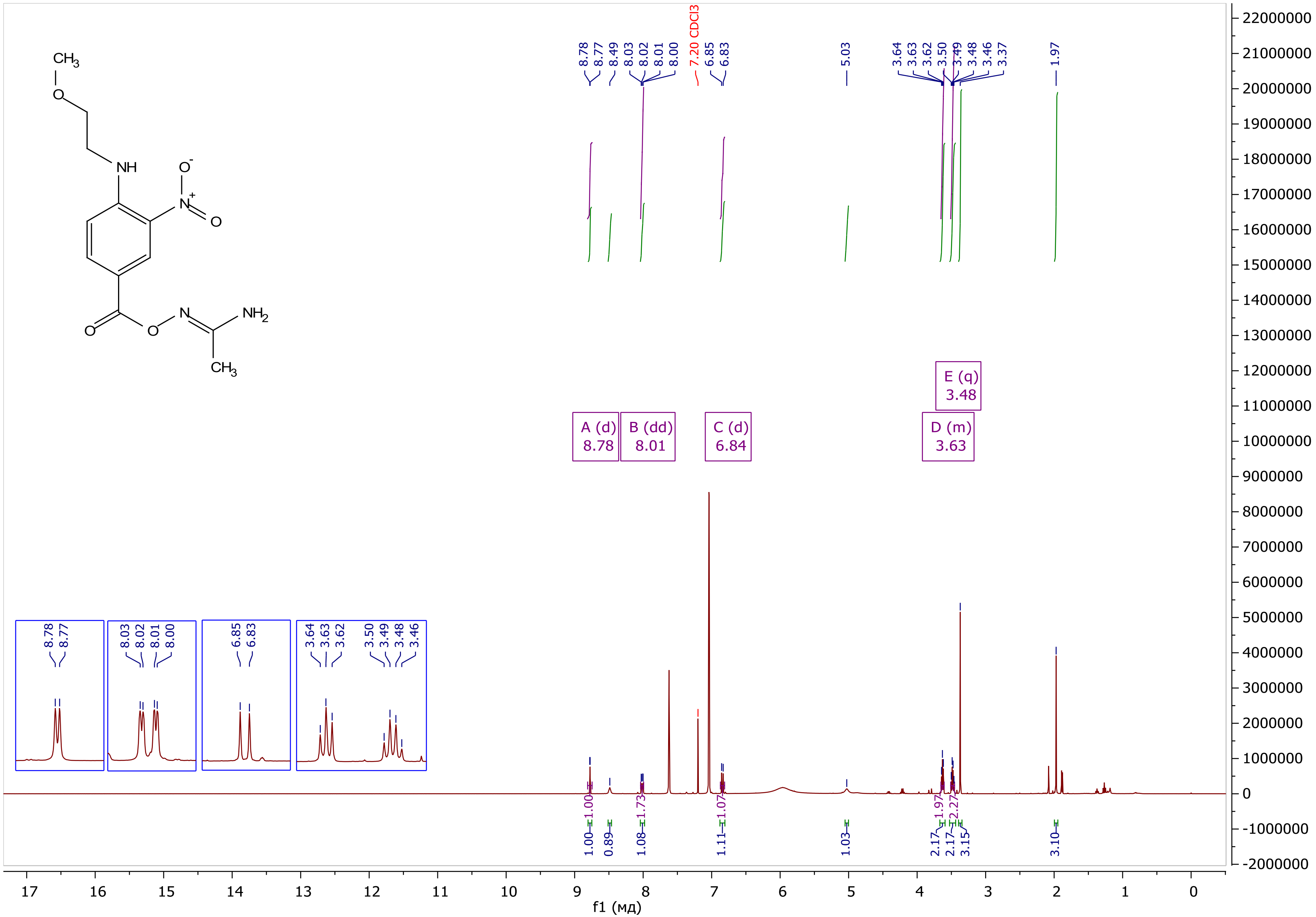

^{13}C NMR spectrum of compound **25c**

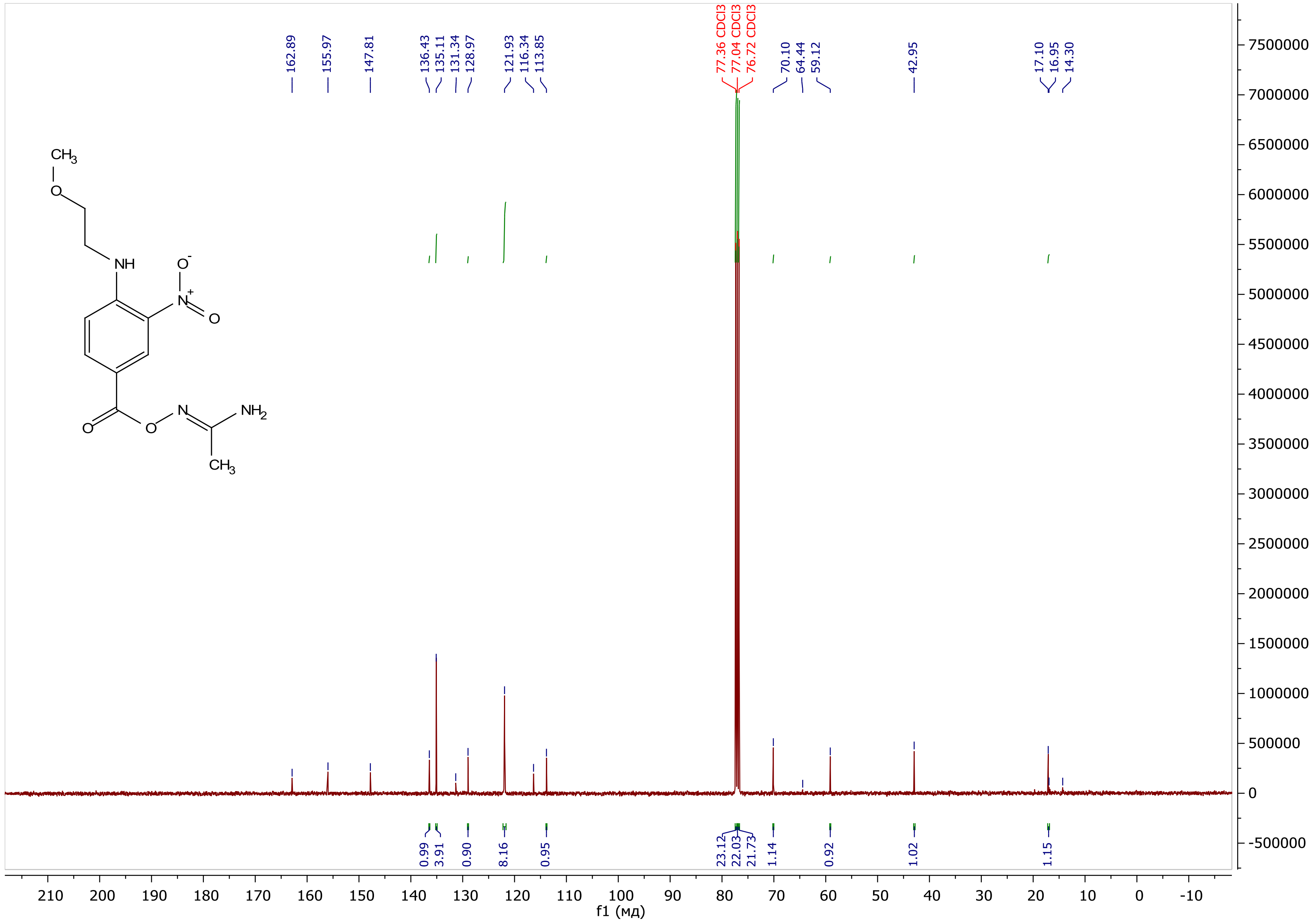

^{1}H NMR spectrum of compound **26a**

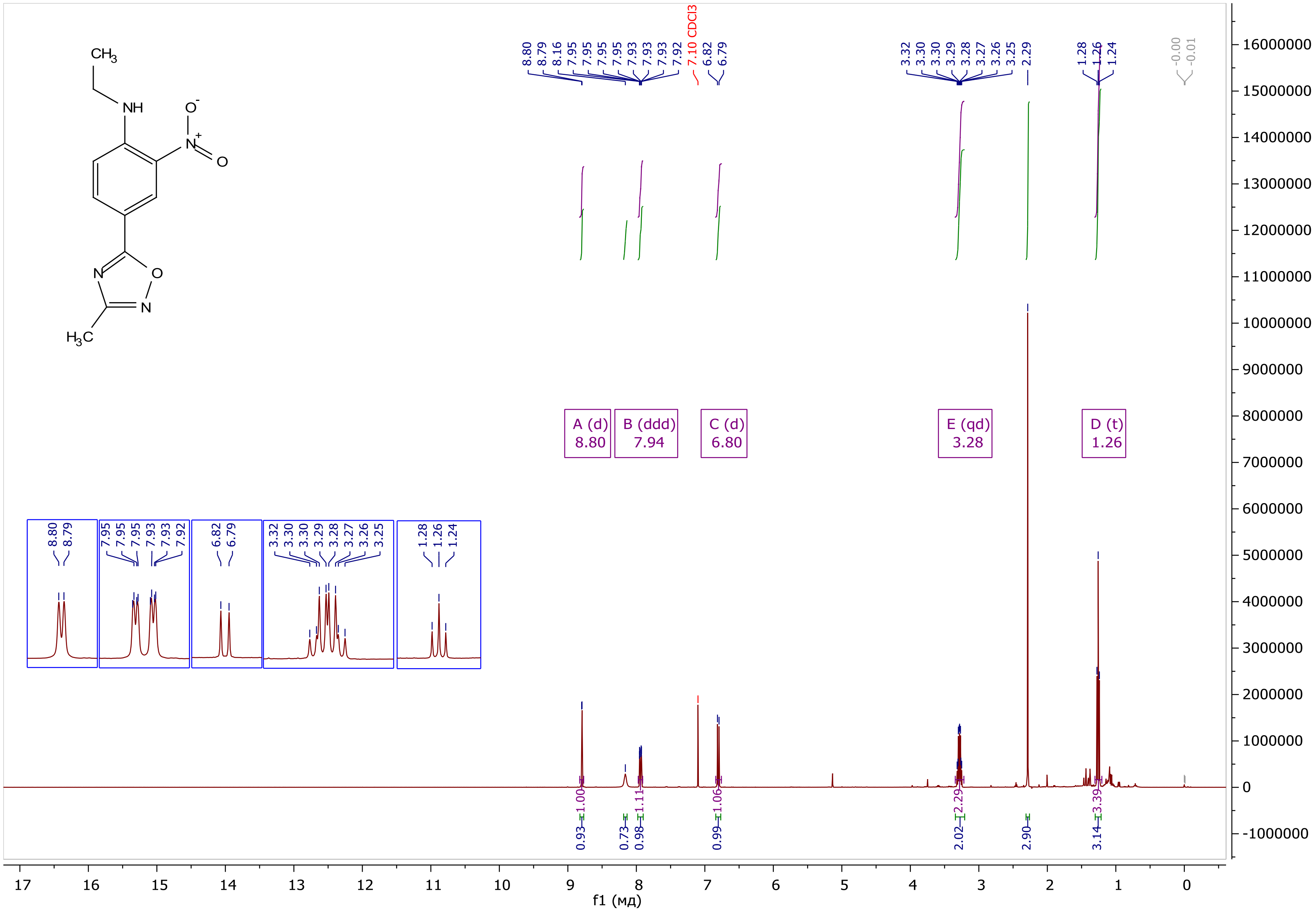

^{13}C NMR spectrum of compound **26a**

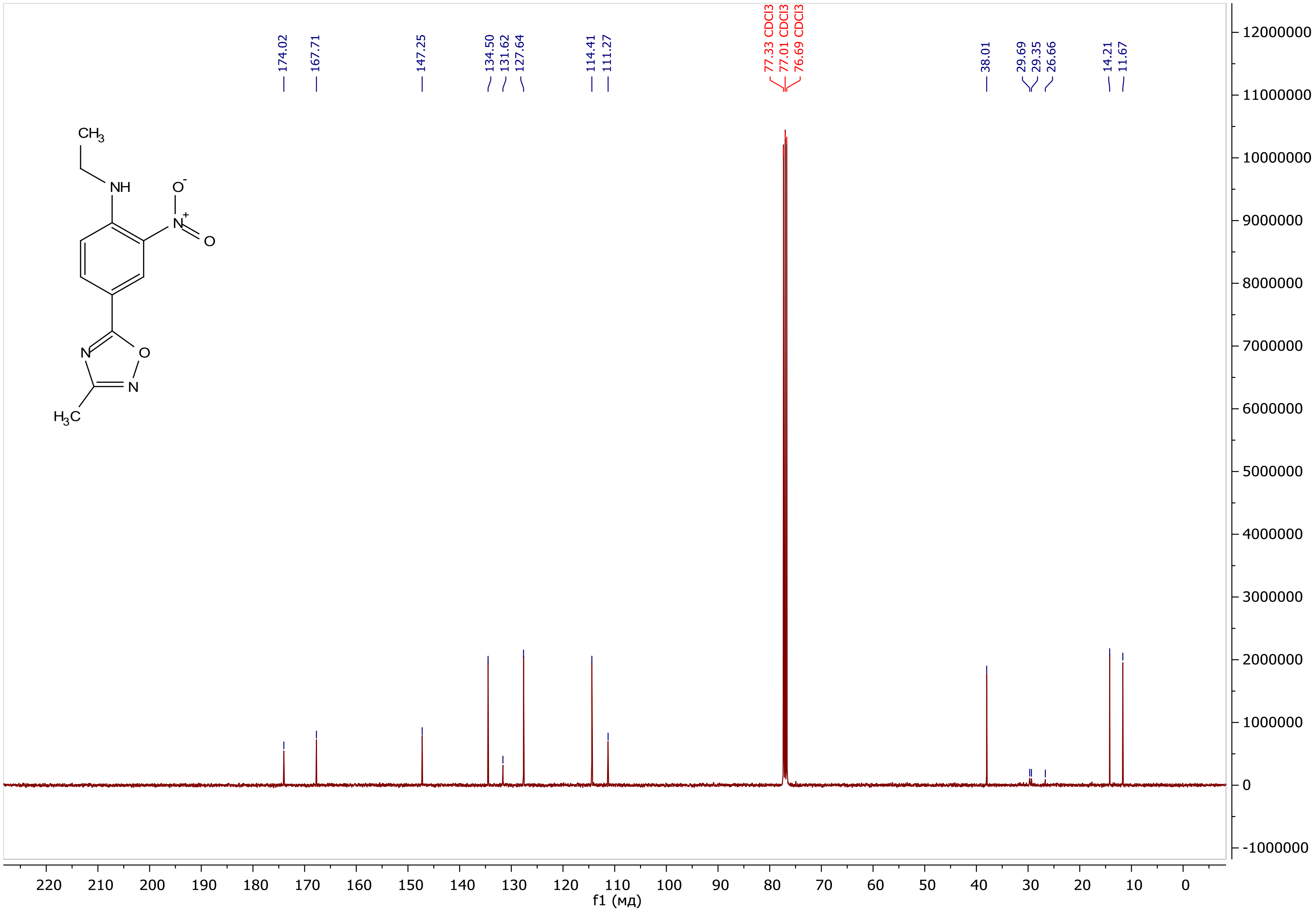

^{1}H NMR spectrum of compound **26b**

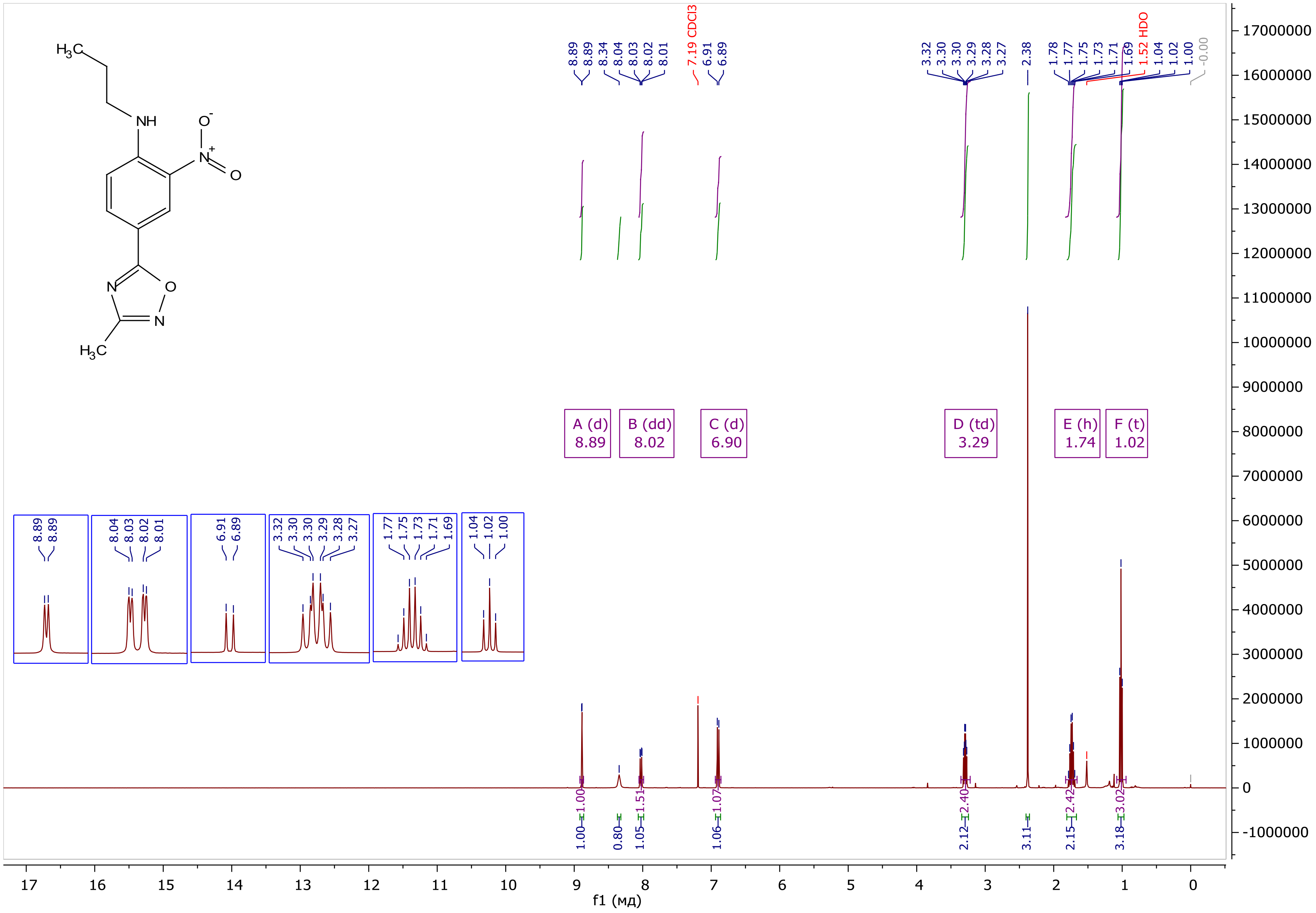

^{13}C NMR spectrum of compound **26b**

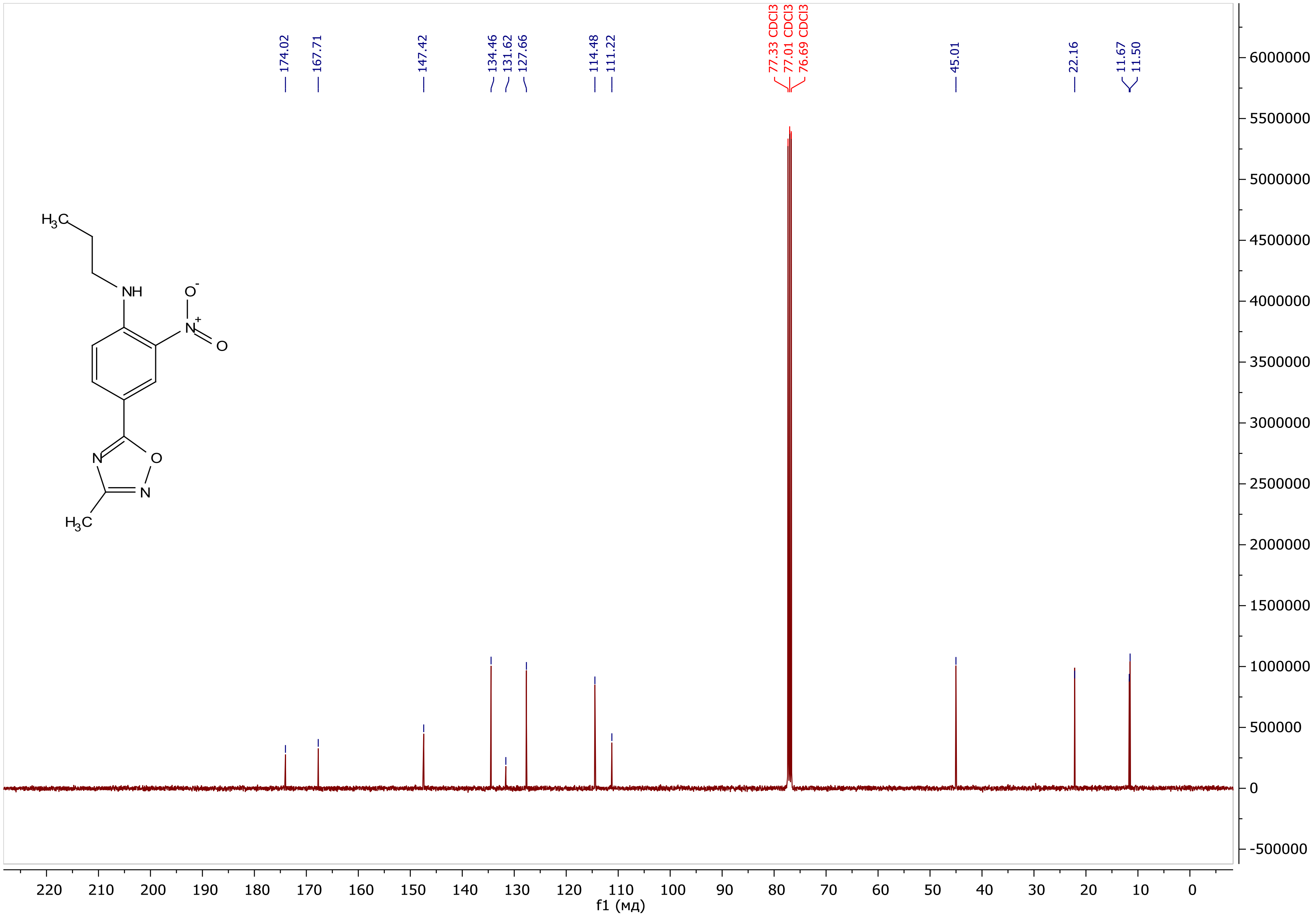

^{1}H NMR spectrum of compound **26c**

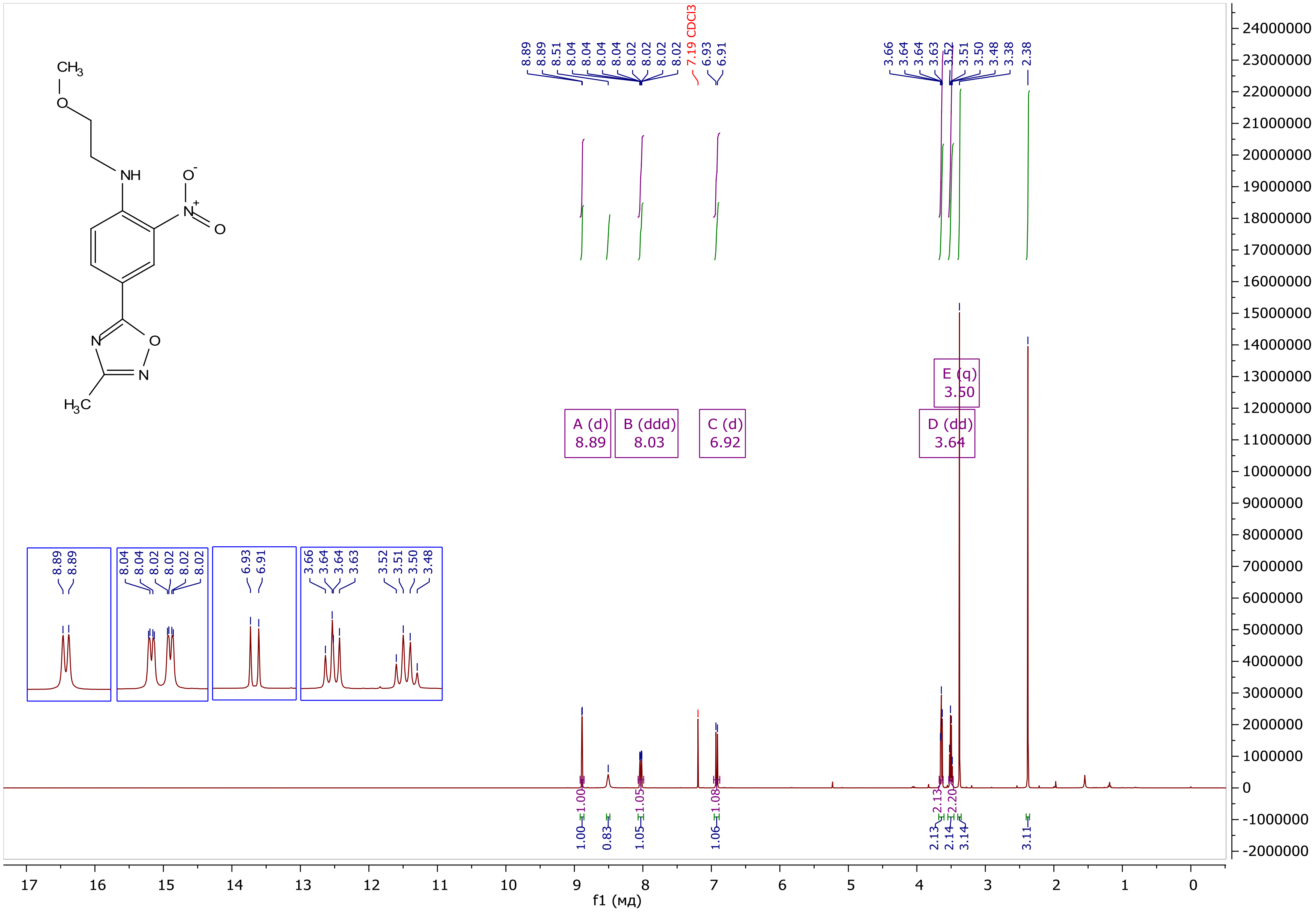

^{13}C NMR spectrum of compound **26c**

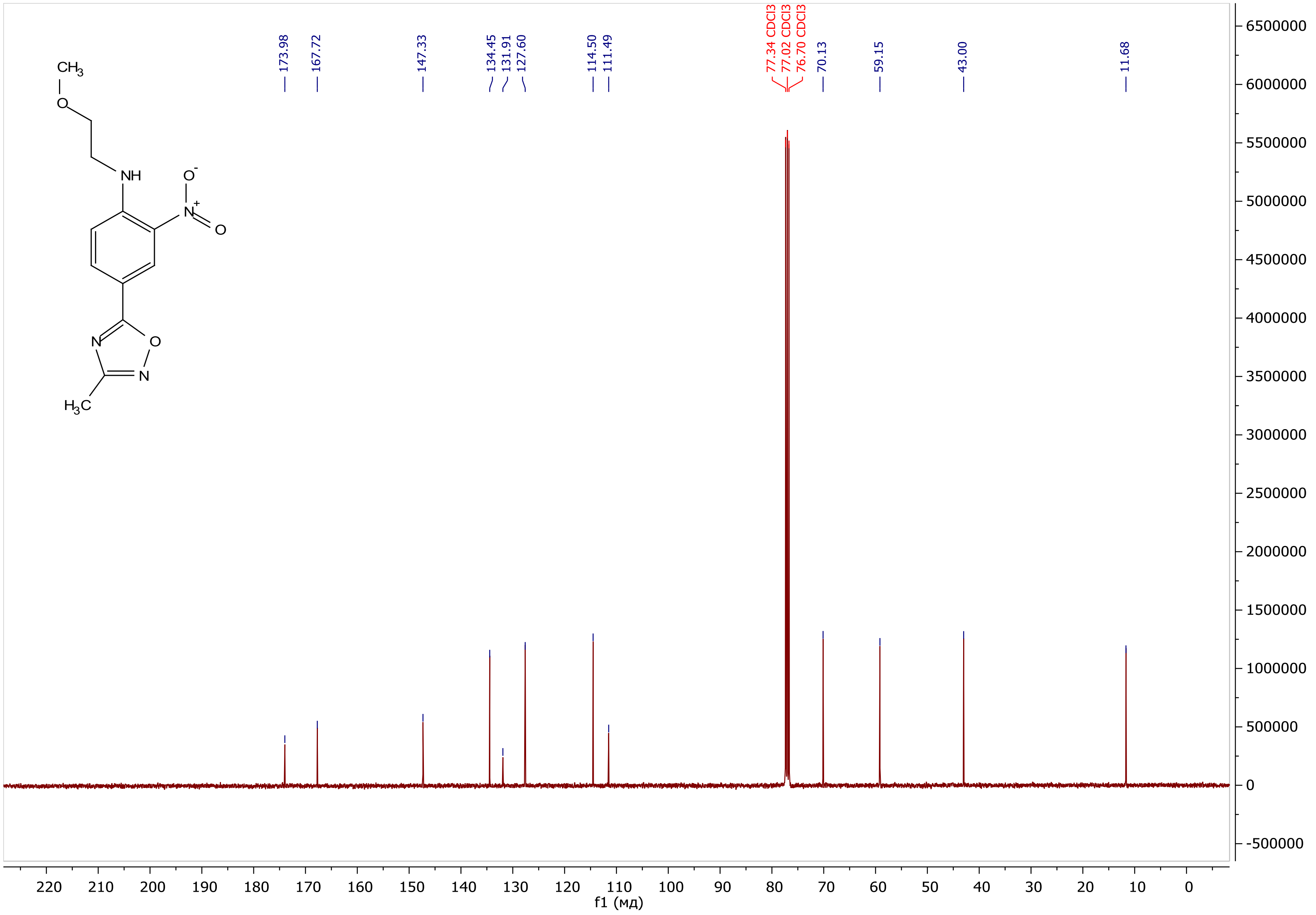

^{1}H NMR spectrum of compound **27a**

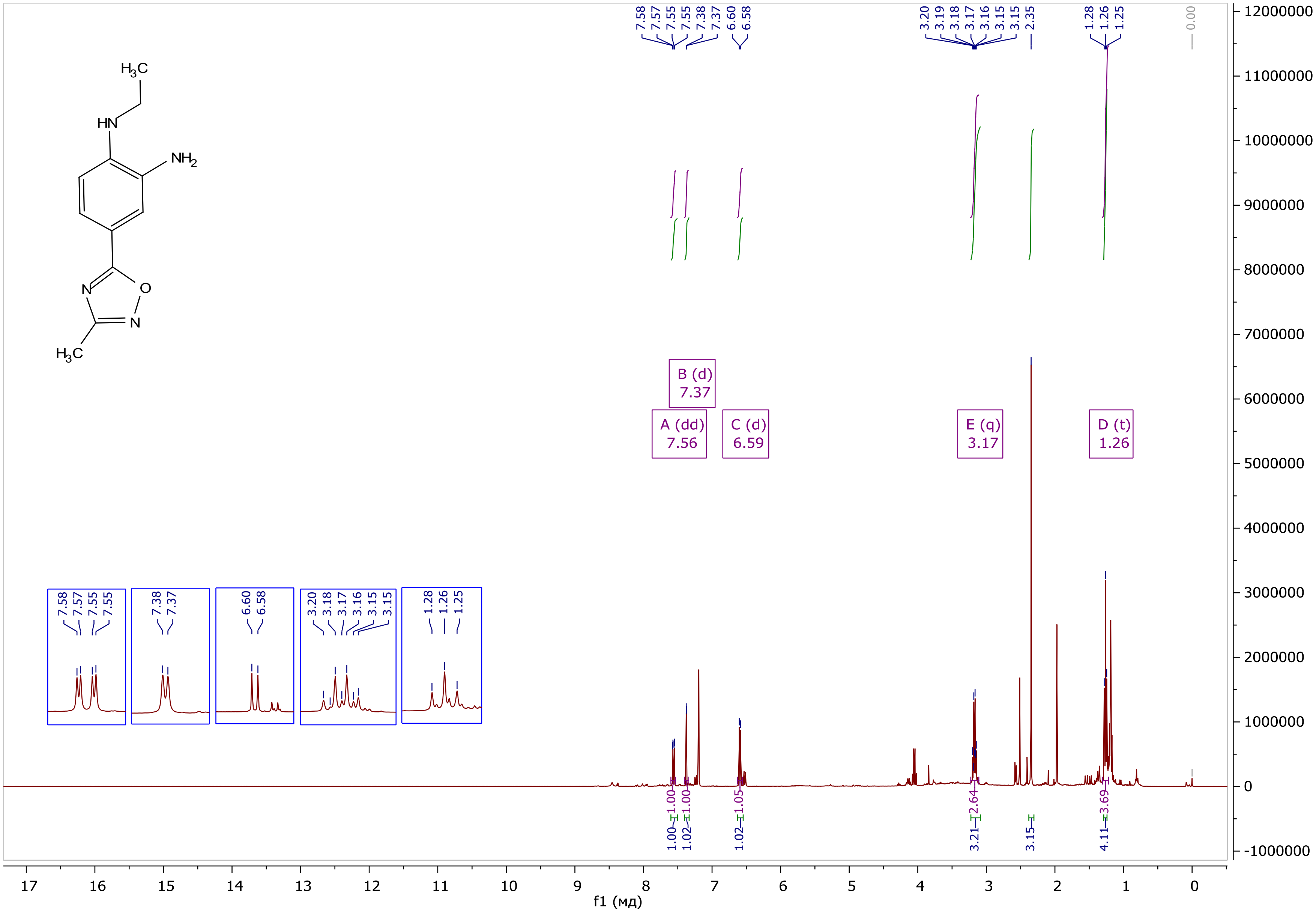

^{13}C NMR spectrum of compound **27a**

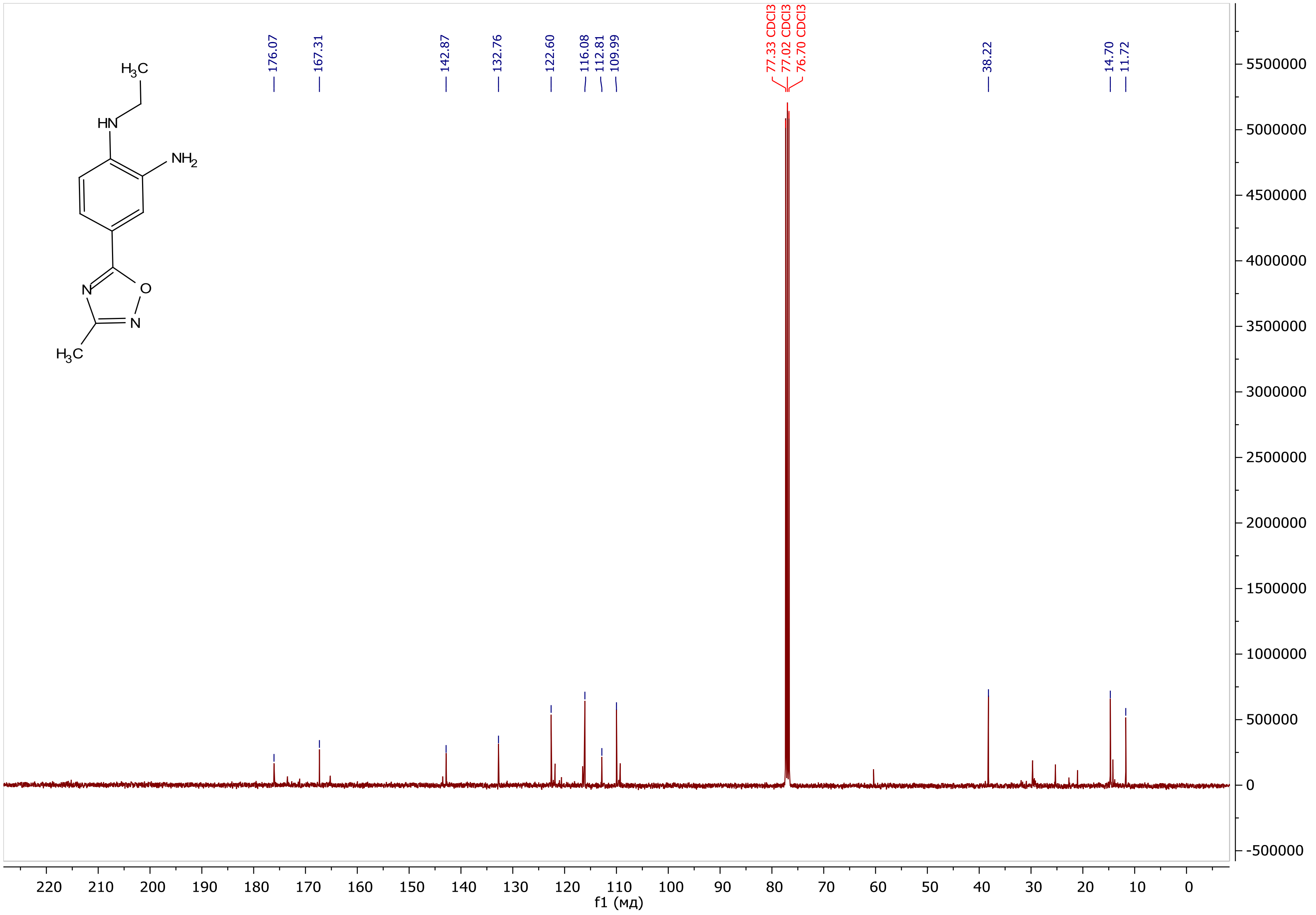

^{1}H NMR spectrum of compound **27b**

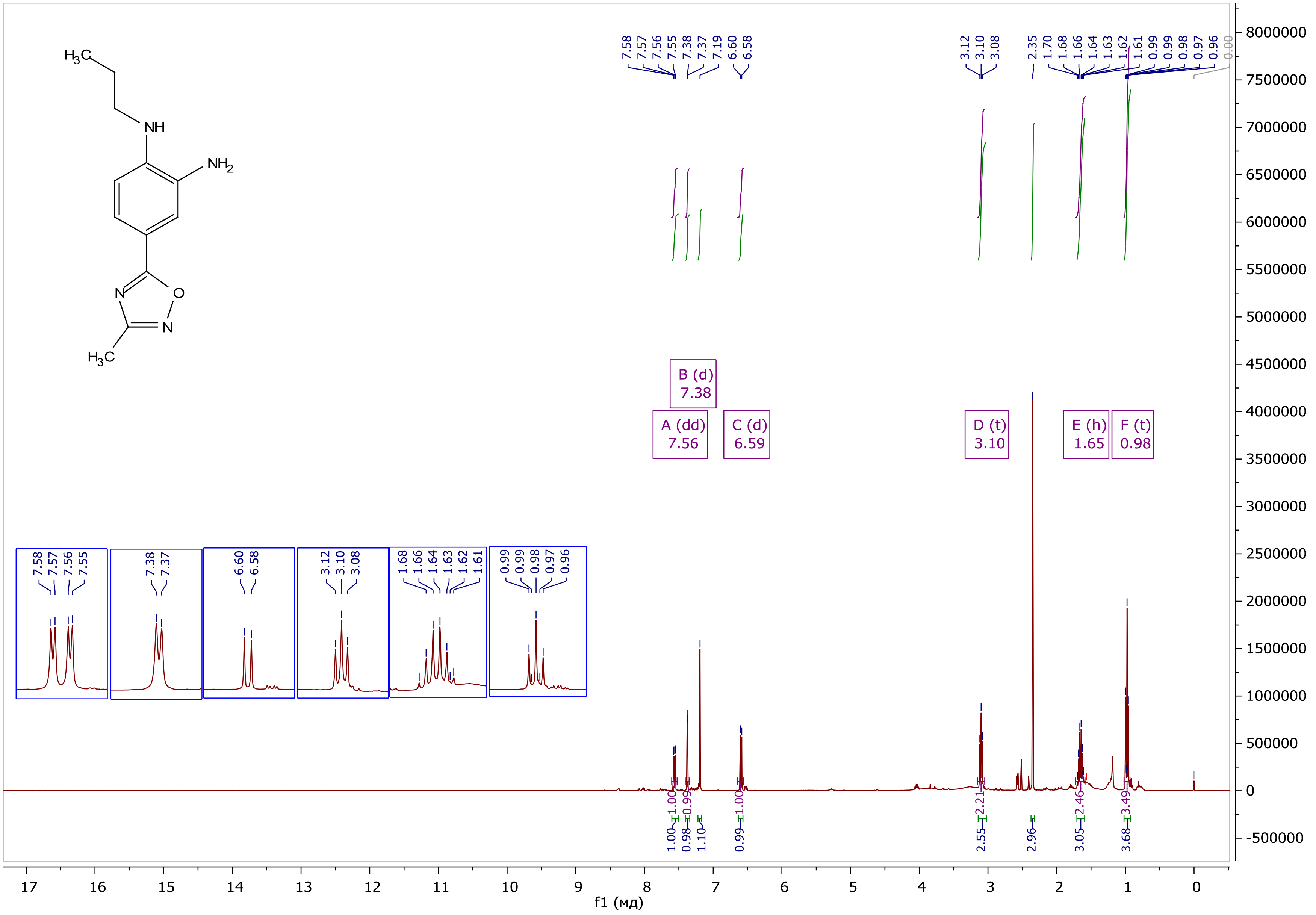

^{13}C NMR spectrum of compound **27b**

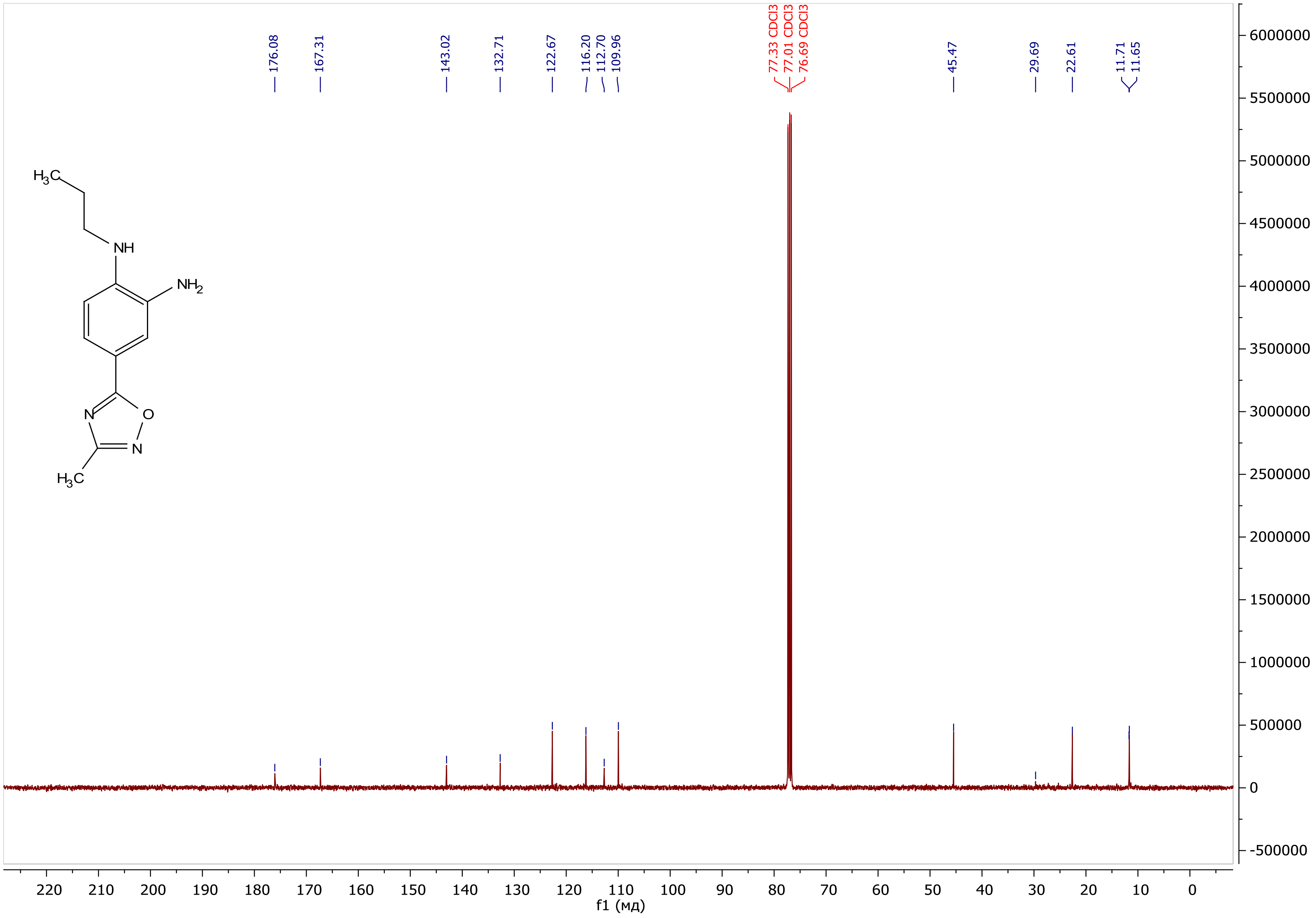

^{1}H NMR spectrum of compound **27c**

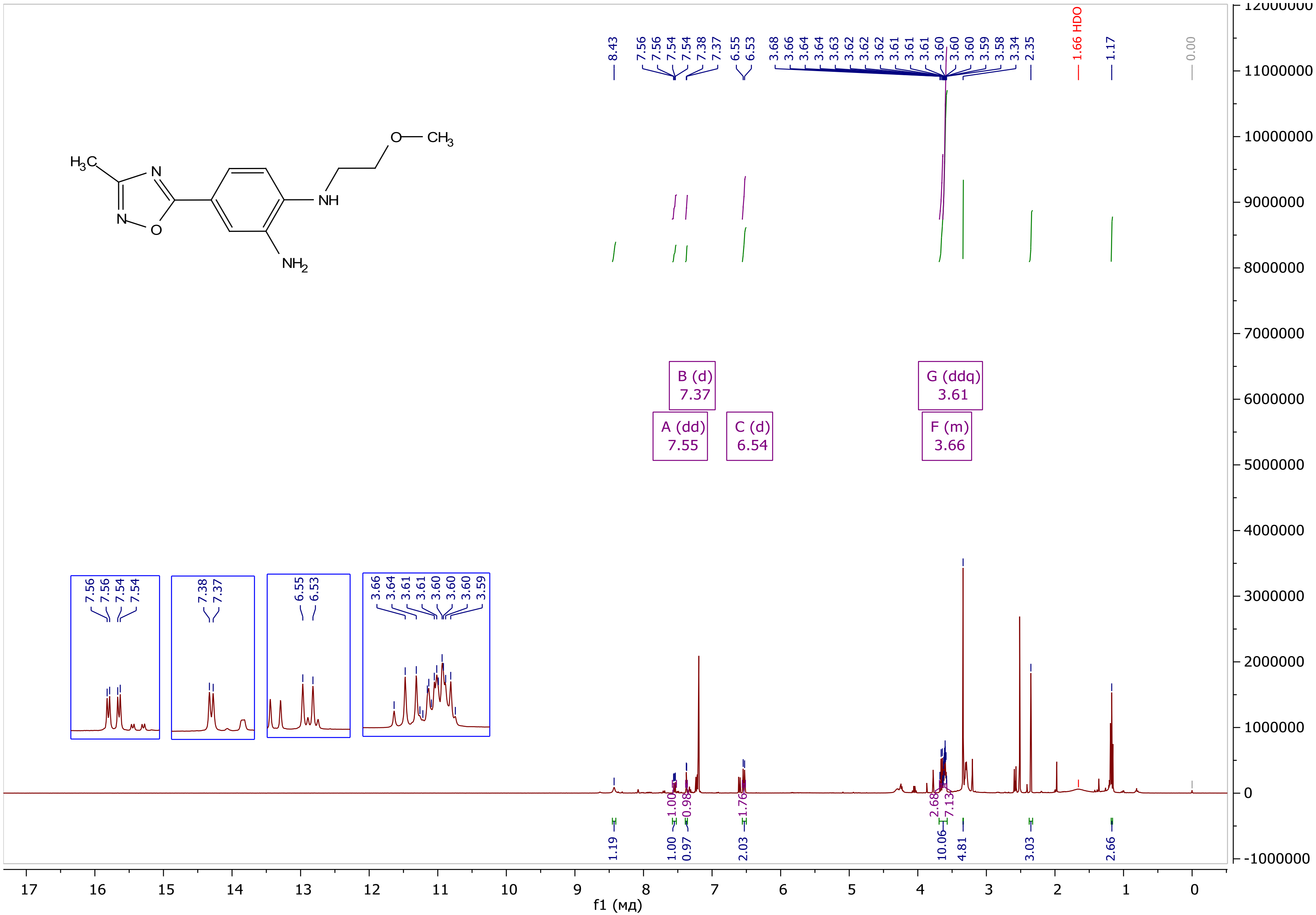

^{13}C NMR spectrum of compound **27c**

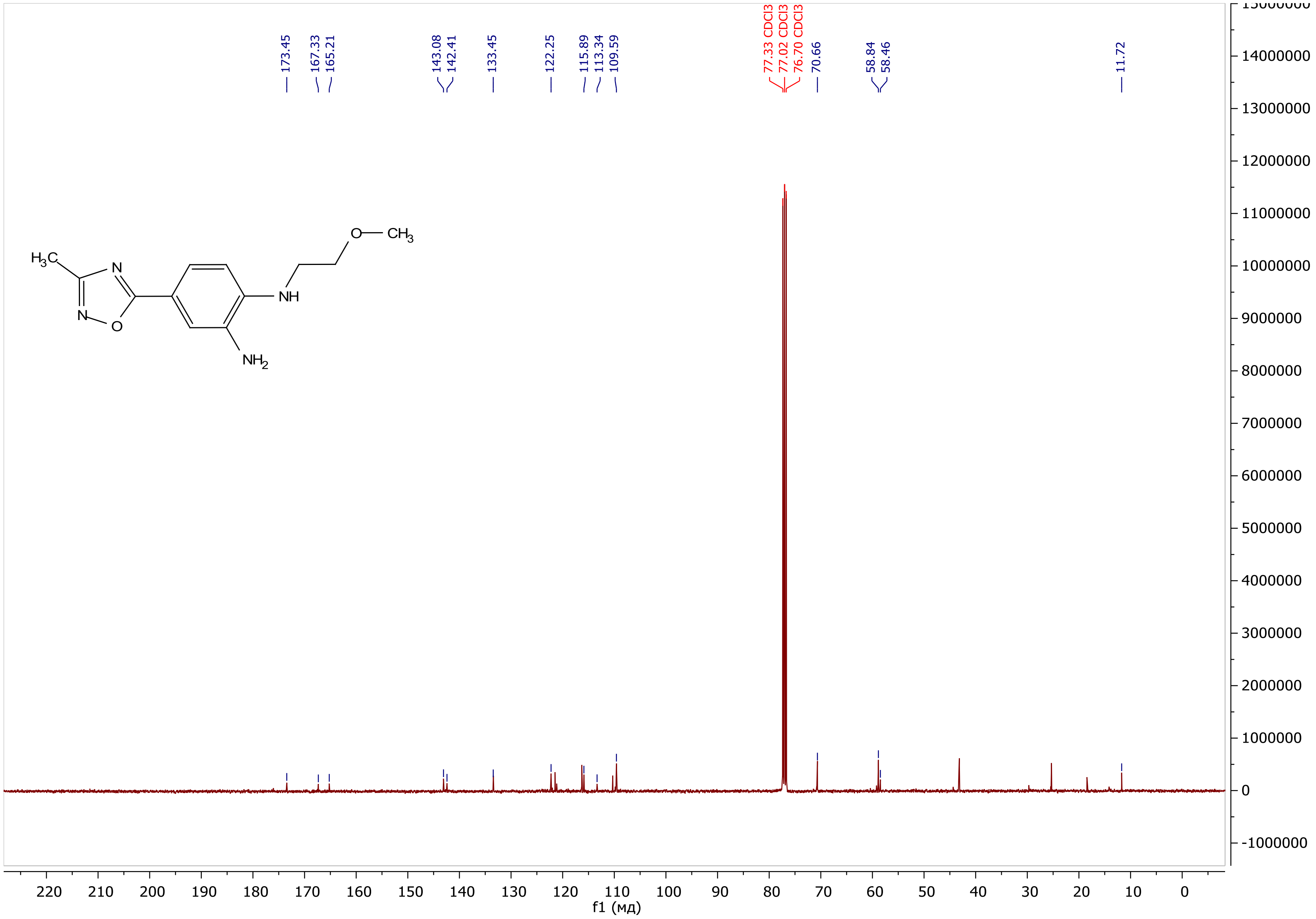

^{1}H NMR spectrum of compound **28a**

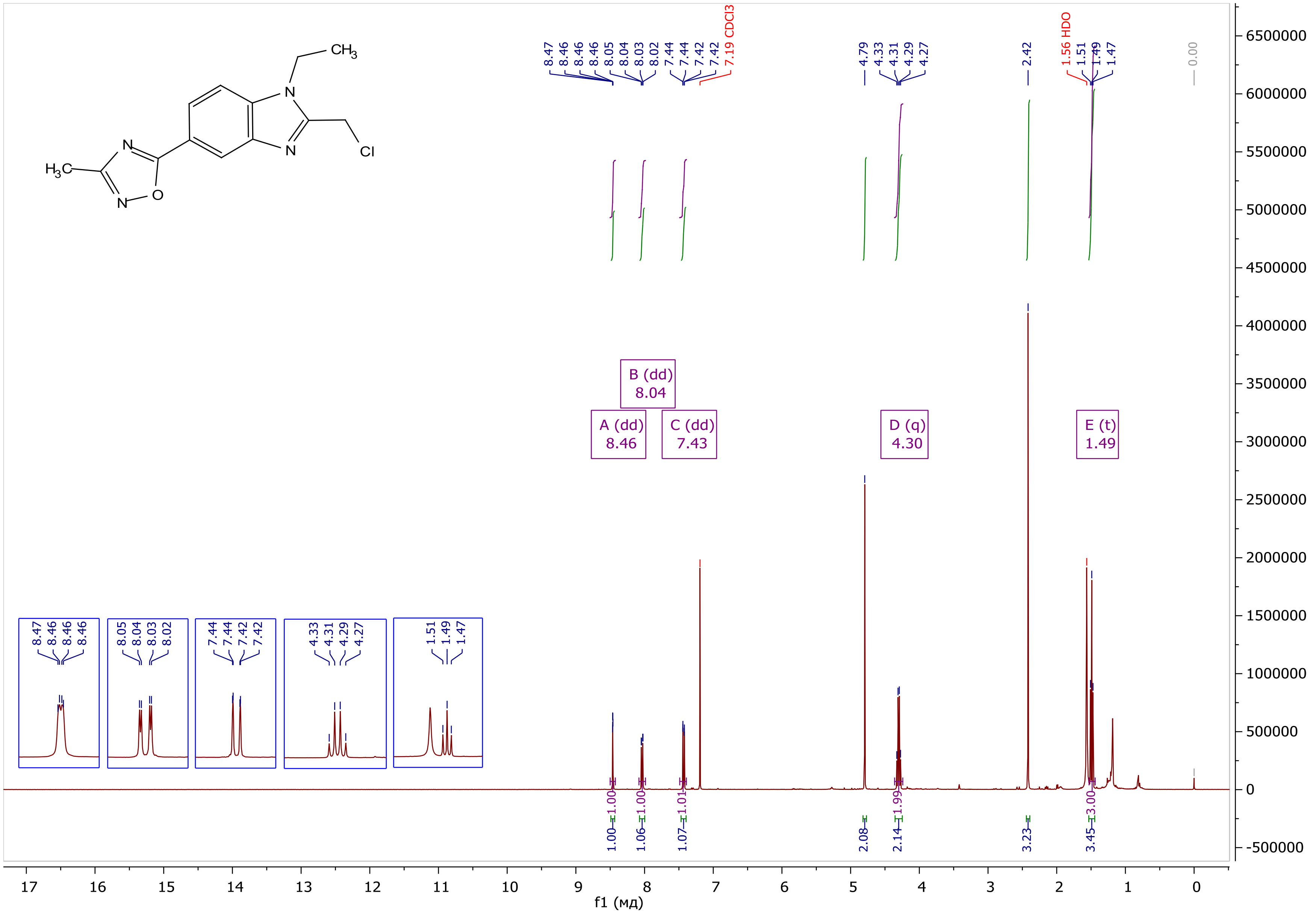

^{13}C NMR spectrum of compound **28a**

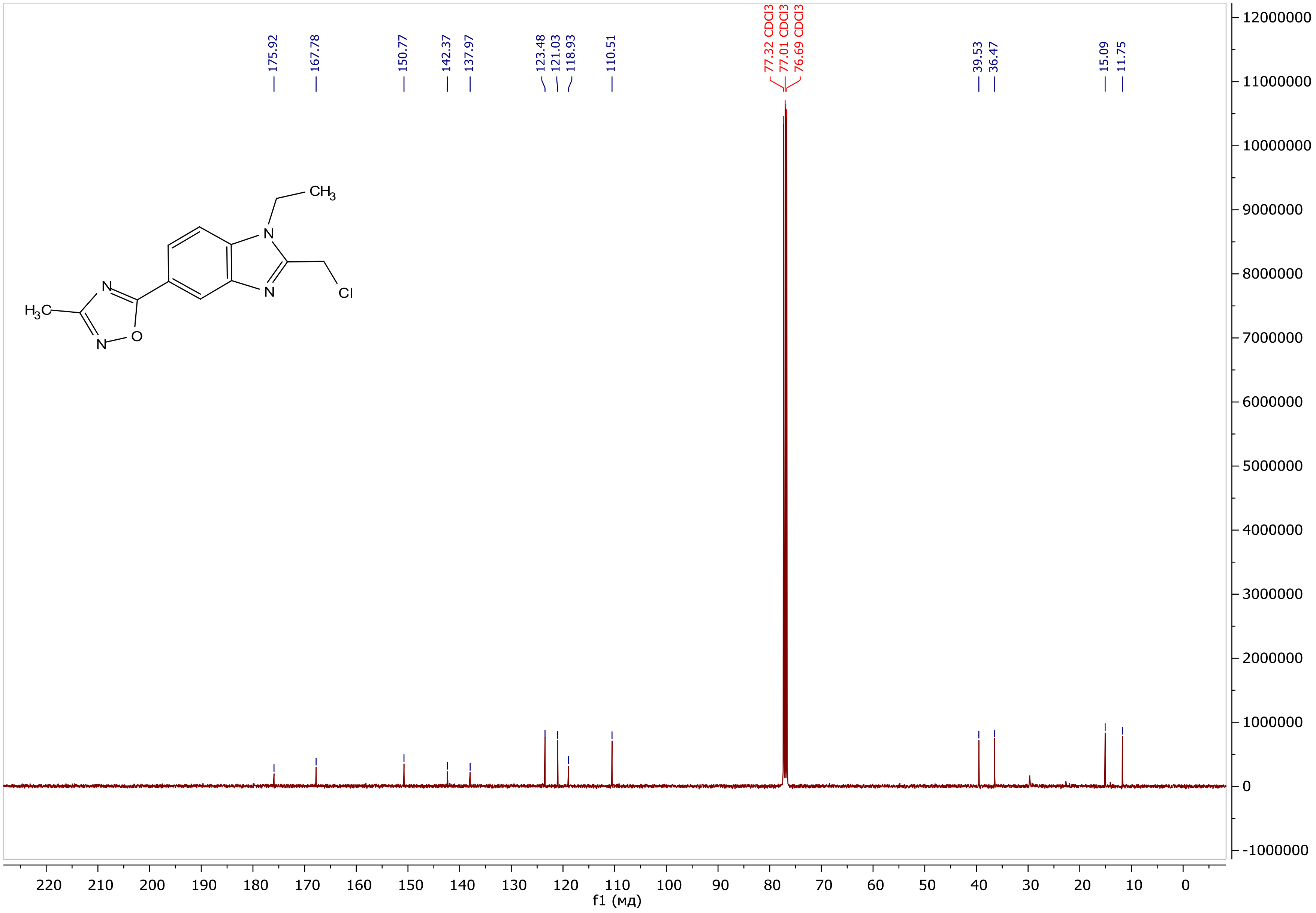

^{1}H NMR spectrum of compound **28b**

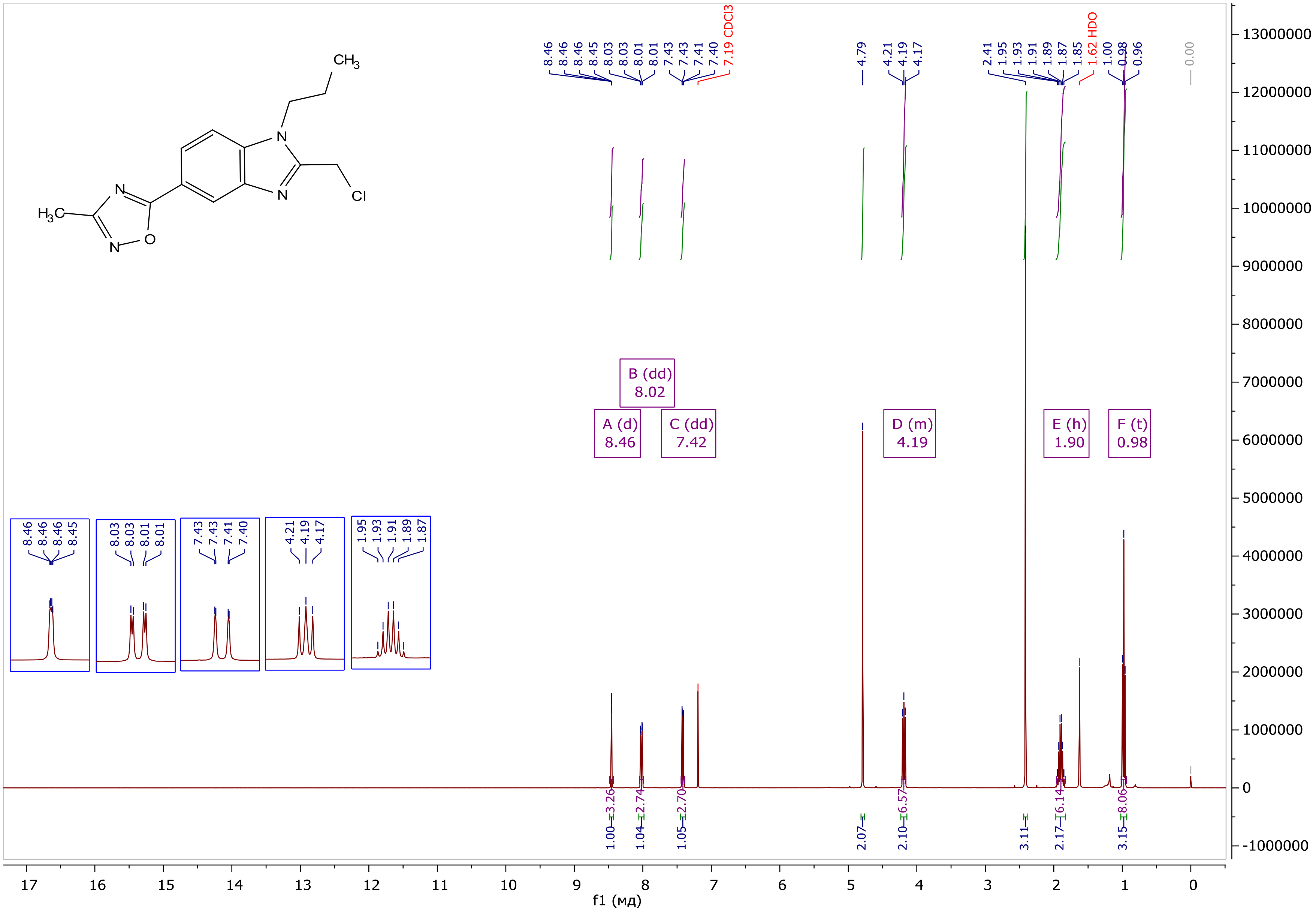

^{13}C NMR spectrum of compound **28b**

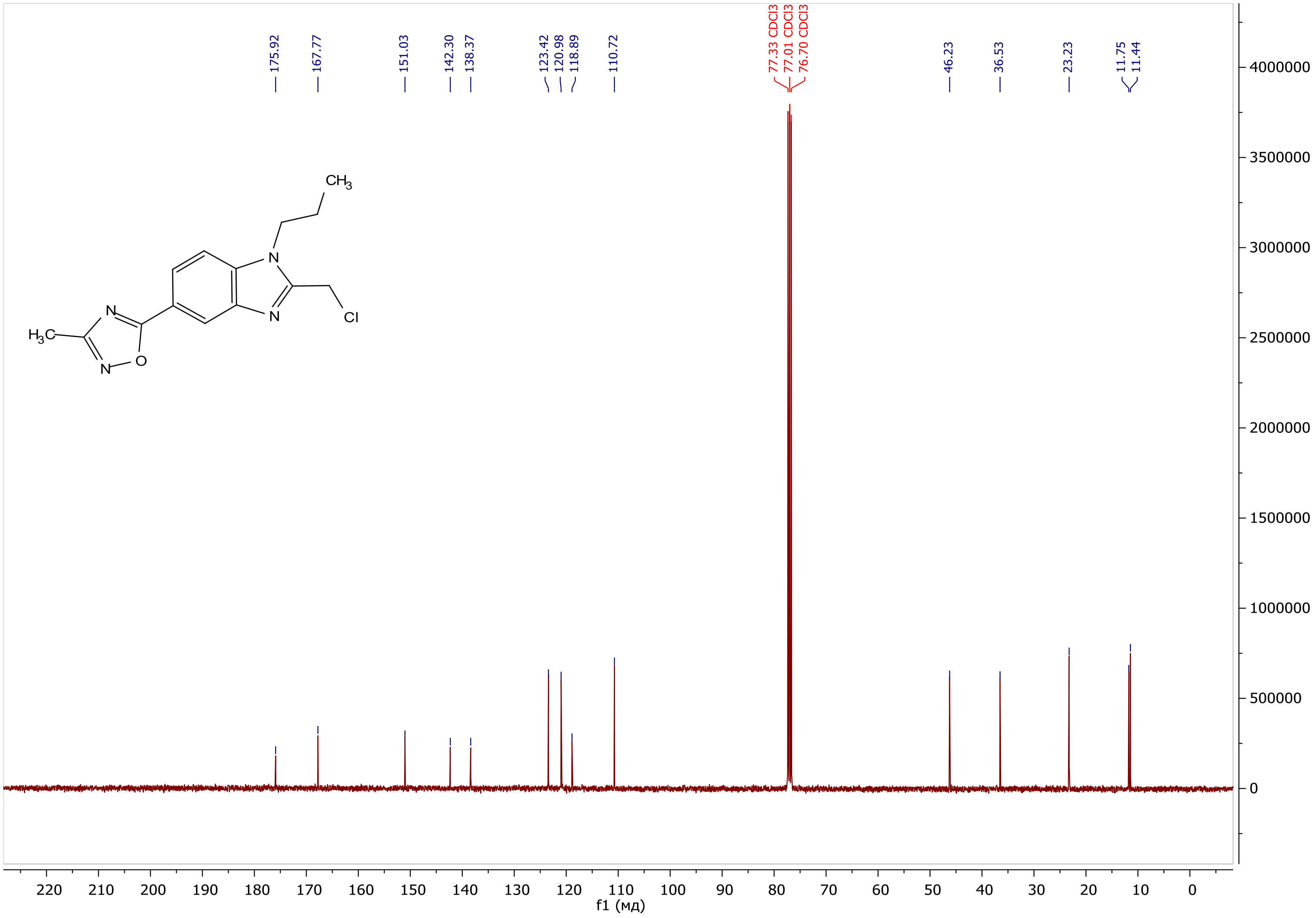

^{1}H NMR spectrum of compound **28c**

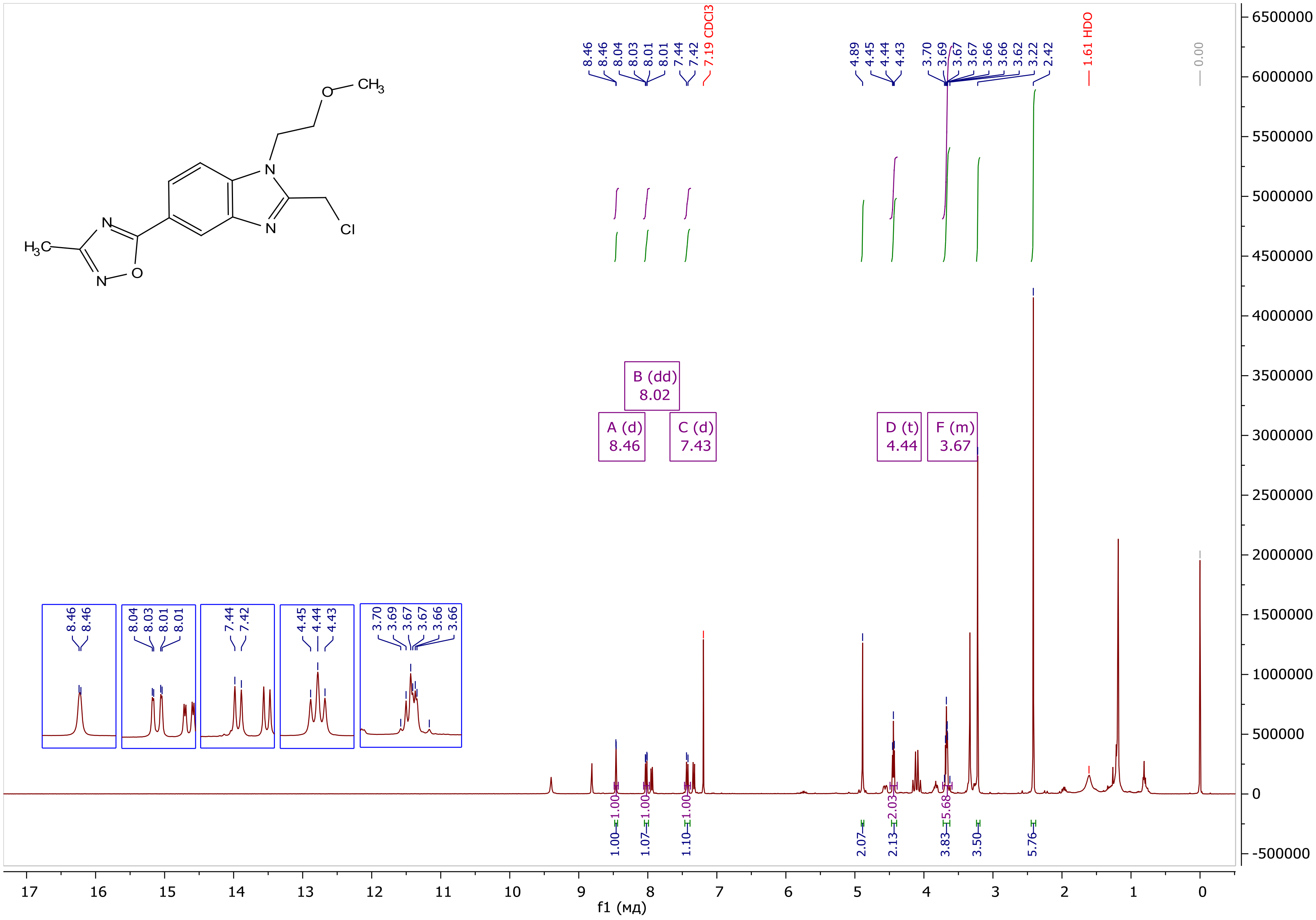

^{13}C NMR spectrum of compound **28c**

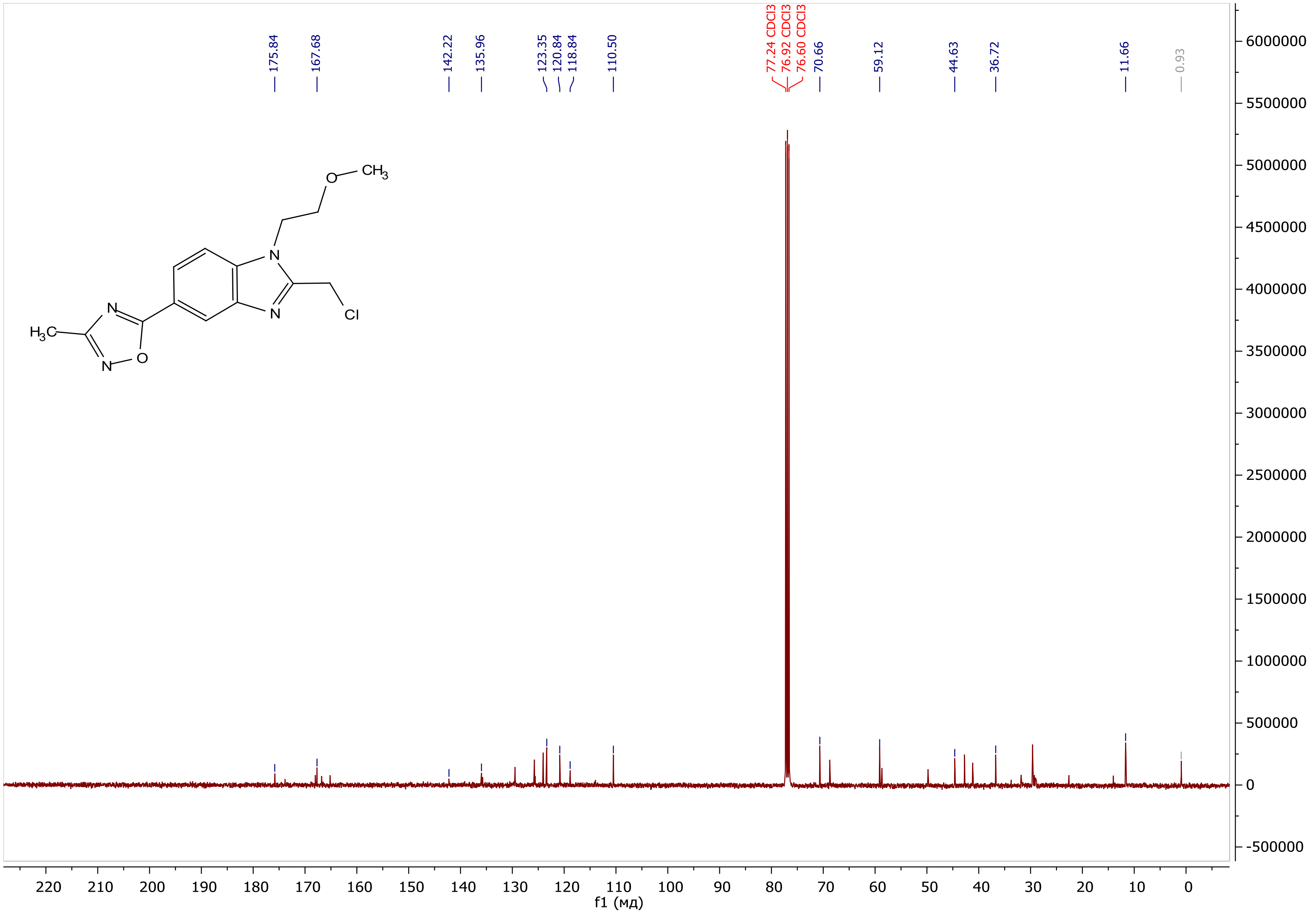

2,4-Disubstituted pyrimidine derivatives 22a-e, 22c', 22e' and intermediates of their synthesis 20a-e, 21a-e, 21c', 21e'

General procedure to synthesis of 4-(benzyloxy)-2-chloropyrimidines **20a-e**. Weighted portions of *t*-BuOK (1 eq) and the corresponding benzyl alcohol (2 eq) were added to anhydrous THF (50 ml) in a round bottom flask. The resulting suspension was stirred at rt (for compounds **20c**,**d**,**e**) or refluxed (for compounds **20a**,**b**) for 30 min. The resulting solution was cooled to 0°C, and slowly added drop by drop to a solution of 2,4-dichloropyrimidine **19** (1 eq) in anhydrous DMF (30 ml), which was preliminarily cooled to -50°C, under vigorous stirring, maintaining a temperature of -50°C constant. After the end of the addition, the resulting solution was stirred at -50°C for 1 h, then for another 2-3 h, gradually raising the temperature to 0°C, the reaction was controlled by TLC (*n*-hexane:EtOAc = 10:1). After the reaction was completed, the solution was transferred to deionized water (100 ml) and the target product was extracted with EtOAc. The organic layer was additionally washed sequentially with deionized water (2×50 ml) and brine solution (1×50 ml), dried with Na_2SO_4 under vigorous stirring for 1 h. The precipitate was filtered off, the solvent was evaporated on a rotary evaporator under reduced pressure to obtain the corresponding 4-(benzyloxy)-2-chloropyrimidine **20a-e** (the content of the second regioisomer, which was separated at the next stage of synthesis, was not more than 15% according to ^{1}H NMR).

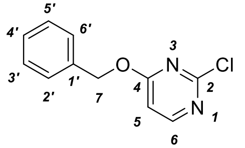

4-(Benzyloxy)-2-chloropyrimidine **20a**, white solid, 3 g, 85%. **^{1}H NMR (400 MHz, CDCl₃, *δ ppm*)**: 8.23 (1H, *d*, *J* = 5.7 Hz, 6-CH), 7.39-7.27 (5H, *m*, 2'-CH, 3'-CH, 4'-CH, 5'-CH, 6'-CH), 6.63 (1H, *d*, *J* = 5.7 Hz, 5-CH), 5.37 (2H, *s*, 7-CH_2). **^{13}C NMR (101 MHz, $CDCl_3$, *δ ppm*)**: 170.1 (C-4), 160.2 (C-2), 158.9 (C-6), 135.2 (C-1'), 128.7 (C-2', C-6' or C-3', C-5'), 128.6 (C-4'), 128.5 (C-3', C-5' or C-2', C-6'), 107.3 (C-5), 69.2 (C-7). **UPLC-MS (ESI**+): found *m/z* 220.9 $[M + H]^+$; calculated $C_{11}H_{10}{}^{35}ClN_2O^+$ 221.0.

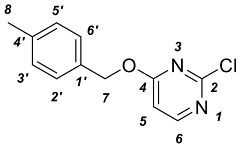

2-Chloro-4-((4-methylbenzyl)oxy)pyrimidine **20b**, white solid, 1 g, 85%. **^{1}H NMR (400 MHz, $CDCl_3$, *δ ppm*)**: 8.22 (1H, *d*, *J* = 5.7 Hz, 6-CH), 7.31-7.26 (2H, *m*, 2'-CH, 6'-CH), 7.14-7.09 (2H, *m*, 3'-CH, 5'-CH), 6.60 (1H, *d*, *J* = 5.7 Hz, 5-CH), 5.32 (2H, *s*, 7-CH_2), 2.29 (3H, *s*, 8-CH_3). **^{13}C NMR (101 MHz, $CDCl_3$, *δ ppm*)**: 170.2 (C-4), 160.2 (C-2), 158.8 (C-6), 138.6 (C-4'), 132.2 (C-1'), 129.3 (C-3', C-5'), 128.7 (C-2', C-6'), 107.4 (C-5), 69.2 (C-7), 21.2 (C-8). **UPLC-MS (ESI**+): found *m/z* 235.1 $[M + H]^+$; calculated $C_{12}H_{12}{}^{35}ClN_2O^+$ 235.1.

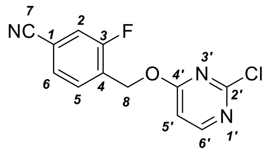

4-(((2-Chloropyrimidin-4-yl)oxy)methyl)-3-fluorobenzonitrile **20c**, white solid, 10 g, 80%. **^{1}H NMR (400 MHz, $CDCl_3$, *δ ppm*)**: 8.29 (1H, *d*, *J* = 5.7 Hz, 6'-CH), 7.56 (1H, *t*, *J* = 7.4 Hz, 6-CH), 7.43 (1H, *dd*, *J* = 7.9, 1.5 Hz, 5-CH), 7.35 (1H, *dd*, *J* = 9.2, 1.5 Hz, 2-CH), 6.68 (1H, *d*, *J* = 5.7 Hz, 5'-CH), 5.48 (2H, *s*, 8-CH_2). **^{13}C NMR (101 MHz, $CDCl_3$, *δ ppm*)**: 169.5 (C-4'), 160.3 (C-3, *d*, $J_{(C-F)}$ = 252.8 Hz), 160.1 (C-2'), 159.4 (C-6'), 131.3 (C-6, *d*, $J_{(C-F)}$ = 4.4 Hz), 128.5 (C-4, *d*, $J_{(C-F)}$ = 14.5 Hz), 128.3 (C-5, *d*, $J_{(C-F)}$= 4.4 Hz), 119.3 (C-2, *d*, $J_{(C-F)}$ = 24.6 Hz), 117.2 (C-7, *d*, $J_{(C-F)}$ = 3.0 Hz), 114.0 (C-1, *d*, $J_{(C-F)}$ = 9.4 Hz), 107.2 (C-5'), 62.1 (C-8, *d*, $J_{(C-F)}$ = 4.3 Hz). **UPLC-MS (ESI**+): found *m/z* 264.0 $[M + H]^+$; calculated $C_{12}H_8{}^{35}ClFN_3O^+$ 264.0.

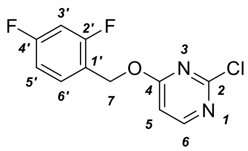

2-Chloro-4-((2,4-difluorobenzyl)oxy)pyrimidine **20d**, white solid, 10 g, 80%. **^{1}H NMR (400 MHz, $CDCl_3$, *δ ppm*)**: 8.24 (1H, *d*, *J* = 5.7 Hz, 6-CH), 7.43-7.39 (1H, *m*, 6'-CH), 6.86-6.76 (2H, *m*, 5'-CH, 3'-CH), 6.62 (1H, *d*, *J* = 5.7 Hz, 5-CH), 5.38 (2H, *s*, 7-CH_2). **^{13}C NMR (101 MHz, $CDCl_3$, *δ ppm*)**: 169.9 (C-4), 163.6 (C-2', *dd*, $J_{(C-F)}$ = 250.6, 11.9 Hz), 161.5 (C-4', *dd*, $J_{(C-F)}$ = 251.9, 12.2 Hz), 160.1 (C-2), 159.1 (C-6), 132.3 (C-6', *dd*, $J_{(C-F)}$ = 9.9, 5.2 Hz), 118.5 (C-1', *dd*, $J_{(C-F)}$ = 14.8, 3.8 Hz), 111.5 (C-5', *dd*, $J_{(C-F)}$ = 21.3, 3.8 Hz), 107.2 (C-5), 104.2 (C-3', *t*, $J_{(C-F)}$ = 25.3 Hz), 62.5 (C-7, *d*, $J_{(C-F)}$ = 3.6 Hz). **UPLC-MS (ESI**+): found *m/z* 257.1 $[M + H]^+$; calculated $C_{11}H_8{}^{35}ClF_2N_2O^+$ 257.0.

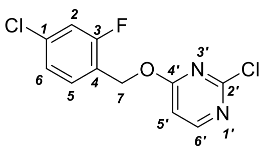

2-Chloro-4-((4-chloro-2-fluorobenzyl)oxy)pyrimidine **20e**, white solid, 6 g, 90%. **^{1}H NMR (400 MHz, $CDCl_3$, *δ ppm*)**: 8.25 (1H, *d*, *J* = 5.7 Hz, 6'-CH), 7.37 (1H, *t*, *J* = 8.0 Hz, 5-CH), 7.12-7.07 (2H, *m*, 2-CH, 6-CH), 6.63 (1H, *d*, *J* = 5.6 Hz, 5'-CH), 5.39 (2H, *s*, 7-CH_2). **^{13}C NMR (101 MHz, $CDCl_3$, *δ ppm*)**: 169.80 (C-4'), 161.01 (C-3, *d*, $J_{(C-F)}$ = 252.8 Hz), 161.14 (C-2'), 159.10 (C-6'), 135.72 (C-1, $J_{(C-F)}$ = 10.2 Hz), 131.81 (C-5, *d*, $J_{(C-F)}$ = 4.5 Hz), 124.70 (C-6, *d*, $J_{(C-F)}$ = 3.7 Hz), 121.17 (C-4, *d*, $J_{(C-F)}$ = 15.0 Hz), 116.52 (C-2, *d*, $J_{(C-F)}$ = 24.7 Hz), 107.25 (C-5'), 62.47 (C-7, *d*, $J_{(C-F)}$ = 4.0 Hz). **UPLC-MS (ESI**+): found *m/z* 273.0 $[M + H]^+$; calculated $C_{11}H_8{}^{35}Cl_2FN_2O^+$ 273.0.

General procedure to synthesis of Boc-protected compounds **21a-e**, **21c'**, **21e'**. A mixture of the corresponding 4-(benzyloxy)-2-chloropyrimidine **20a-e** (1 eq) and N-Boc-piperazine (3 eq) was dissolved in anhydrous THF (50 ml) under vigorous stirring. The mixture was refluxed for 8 h, the reaction was controlled by TLC (*n*-hexane:EtOAc = 10:1). After the reaction was completed, the solvent was evaporated on a rotary evaporator under reduced pressure, the residue was dissolved in EtOAc (50 ml), washed sequentially with deionized water (3×30 ml) and brine solution

(1×50 ml). The organic layer was dried with Na_2SO_4 under vigorous stirring for 1 h, after which the precipitate was filtered off, the solvent was evaporated on a rotary evaporator under reduced pressure. The target product was purified by column chromatography on silica gel (*n*-hexane:EtOAc = 8:1). The fractions containing the target product were combined, the solvent was evaporated on a rotary evaporator under reduced pressure to dryness to form the corresponding Boc-protected compound **21a-e**, **21c'**, **21e'**.

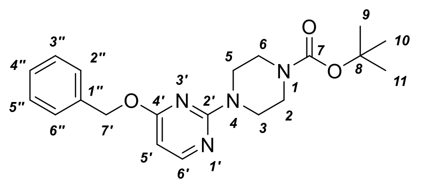

tert-Butyl 4-(4-(benzyloxy)pyrimidin-2-yl)piperazine-1-carboxylate **21a**, light yellow solid, 3.8 g, 75%. **^{1}H NMR (400 MHz, $CDCl_3$, *δ ppm*)**: 8.00 (1H, *d*, *J* = 5.6 Hz, 6'-CH), 7.37-7.22 (5H, *m*, 2"-CH, 3"-CH, 4"-CH, 5"-CH, 6"-CH), 5.98 (1H, *d*, *J* = 5.6 Hz, 5'-CH), 5.27 (2H, *s*, 7'-CH_2), 3.72-3.69 (4H, *m*, 3-CH_2, 5-CH_2), 3.41-3.39 (4H, *m*, 2-CH_2, 6-CH_2), 1.42 (9H, *s*, 9-CH_3, 10-CH_3, 11-CH_3). **^{13}C NMR (101 MHz, $CDCl_3$, *δ ppm*)**: 169.4 (C-4'), 161.5 (C-2'), 158.1 (C-6'), 154.9 (C-7), 136.8 (C-1"), 128.5 (C-3", C-5"), 128.0 (C-4"), 127.9 (C-2", C-6"), 97.2 (C-5'), 79.9 (C-8), 67.4 (C-7'), 43.7 (C-2, C-3, C-5, C-6, *br.s.*), 28.4 (C-9, C-10, C-11). **UPLC-MS (ESI+)**: found *m/z* 371.1 $[M + H]^+$; calculated $C_{20}H_{27}N_4O_3^+$ 371.2.

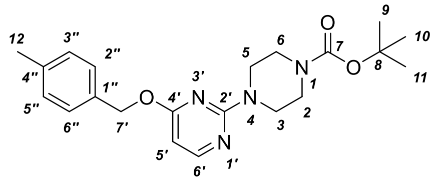

tert-Butyl 4-(4-((4-methylbenzyl)oxy)pyrimidin-2-yl)piperazine-1-carboxylate **21b**, light yellow solid, 1.3 g, 80%. **^{1}H NMR (400 MHz, $CDCl_3$, *δ ppm*)**: 7.98 (1H, *d*, *J* = 5.6 Hz, 6'-CH), 7.24-7.22 (2H, *m*, 2"-CH, 6"-CH), 7.11-7.09 (2H, *m*, 3"-CH, 5"-CH), 5.96 (1H, *d*, *J* = 5.6 Hz, 5'-CH), 5.23 (2H, *s*, 7'-CH_2), 3.73-3.70 (4H, *m*, 3-CH_2, 5-CH_2), 3.42-3.39 (4H, *m*, 2-CH_2, 6-CH_2), 2.28 (3H, *s*, 12-CH_3), 1.42 (9H, *s*, 9-CH_3, 10-CH_3, 11-CH_3). **^{13}C NMR (101 MHz, $CDCl_3$, *δ ppm*)**: 169.4 (C-4'), 161.6 (C-2'), 158.0 (C-6'), 154.9 (C-7), 137.8 (C-4"), 133.7 (C-1"), 129.2 (C-3", C-5"), 128.1 (C-2", C-6"), 97.3 (C-5'), 79.9 (C-8), 67.3 (C-7'), 43.7 (C-2, C-3, C-5, C-6, *br.s.*), 28.5 (C-9, C-10, C-11), 21.2 (C-12). **UPLC-MS (ESI+)**: found *m/z* 385.1 $[M + H]^+$; calculated $C_{21}H_{29}N_4O_3^+$ 385.2

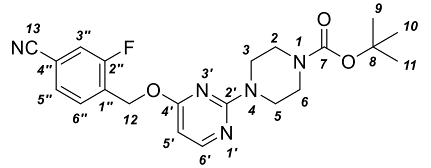

tert-Butyl 4-(4-((4-cyano-2-fluorobenzyl)oxy)pyrimidin-2-yl)piperazine-1-carboxylate **21c**, white solid, 11.8 g, 75%. **^{1}H NMR (400 MHz, $CDCl_3$, *δ ppm*)**: 8.03 (1H, *d*, *J* = 5.6 Hz, 6'-CH), 7.51 (1H, *t*, *J* = 7.5 Hz, 6"-CH), 7.40 (1H, *dd*, *J* = 8.0, 1.5 Hz, 5"-CH), 7.32 (1H, *dd*, *J* = 9.3, 1.5 Hz, 3"-CH), 6.01 (1H, *d*, *J* = 5.6 Hz, 5'-CH), 5.39 (2H, *s*, 12-CH_2), 3.70-3.67 (4H, *m*, 3-CH_2, 5-CH_2), 3.41-3.38 (4H, *m*, 2-CH_2, 6-CH_2), 1.42 (9H, *s*, 9-CH_3, 10-CH_3, 11-CH_3). **^{13}C NMR (101 MHz, $CDCl_3$, *δ ppm*)**: 168.7 (C-4'), 161.4 (C-2'), 159.7 (C-2", *d*, $J_{(C\text{-}F)}$ = 251.1 Hz), 158.6 (C-6'), 154.8 (C-7), 130.4 (C-6", *d*, $J_{(C\text{-}F)}$ = 4.9 Hz), 130.3 (C-1", *d*, $J_{(C\text{-}F)}$ = 14.4 Hz), 128.3 (C-5", *d*, $J_{(C\text{-}F)}$ = 4.2 Hz), 119.0 (C-3", *d*, $J_{(C\text{-}F)}$ = 24.7 Hz), 117.4 (C-13, *d*, $J_{(C\text{-}F)}$ = 2.9 Hz), 113.1 (C-4", *d*, $J_{(C\text{-}F)}$ = 9.5 Hz), 96.8 (C-5'), 80.0 (C-8), 60.0 (C-12, *d*, $J_{(C\text{-}F)}$ = 4.8 Hz), 43.7 (C-2, C-3, C-5, C-6, *br.s.*), 28.4 (C-9, C-10, C-11). **HRMS (ESI+)**: found *m/z* 413.8634 $[M + H]^+$; calculated $C_{21}H_{25}FN_5O_3^+$ 414.1863.

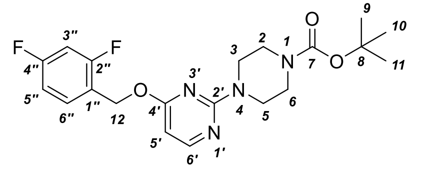

tert-Butyl 4-(4-((2,4-difluorobenzyl)oxy)pyrimidin-2-yl)piperazine-1-carboxylate **21d**, white solid, 12.7 g, 80%. **^{1}H NMR (400 MHz, $CDCl_3$, *δ ppm*)**: 8.10 (1H, *d*, *J* = 5.6 Hz, 6'-CH), 7.44 (1H, *td*, *J* = 8.3, 6.3 Hz, 6"-CH), 6.93-6.83 (2H, *m*, 5"-CH, 3"-CH), 6.06 (1H, *d*, *J* = 5.6 Hz, 5'-CH), 5.39 (2H, *s*, 12-CH_2), 3.82-3.79 (4H, *m*, 3-CH_2, 5-CH_2), 3.52-3.49 (4H, *m*, 2-CH_2, 6-CH_2), 1.51 (9H, *s*, 9-CH_3, 10-CH_3, 11-CH_3). **^{13}C NMR (101 MHz, $CDCl_3$, *δ ppm*)**: 169.0 (C-4'), 162.9 (C-2", *dd*, $J_{(C\text{-}F)}$ = 249.4, 11.7 Hz), 161.5 (C-2'), 160.9 (C-4", *dd*, $J_{(C\text{-}F)}$ = 250.4, 12.1 Hz), 158.3 (C-6'), 154.9 (C-7), 131.3 (C-6", *dd*, $J_{(C\text{-}F)}$ = 9.9, 5.5 Hz), 120.0 (C-1", *dd*, $J_{(C\text{-}F)}$ = 14.9, 4.0 Hz), 111.4 (C-5", *dd*, $J_{(C\text{-}F)}$ = 21.1, 3.7 Hz), 103.9 (C-3", *t*, $J_{(C\text{-}F)}$ = 25.2 Hz), 97.1 (C-5'), 80.0 (C-8), 60.4 (C-12, *d*, $J_{(C\text{-}F)}$ = 4.0 Hz), 43.7 (C-2, C-3, C-5, C-6, *br.s.*), 28.4 (C-9, C-10, C-11). **HRMS (ESI+)**: found *m/z* 406.9962 $[M + H]^+$; calculated $C_{20}H_{25}F_2N_4O_3^+$ 407.1816.

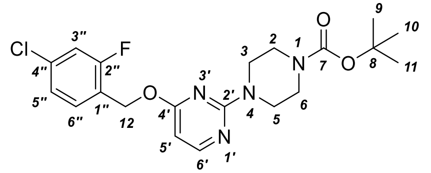

tert-Butyl 4-(4-((4-cyano-2-fluorobenzyl)oxy)pyrimidin-2-yl)piperazine-1-carboxylate **21e**, white solid, 7.9 g, 85%. **^{1}H NMR (400 MHz, $CDCl_3$, *δ ppm*)**: 8.01 (1H, *d*, *J* = 5.6 Hz, 6'-CH), 7.31 (1H, *t*, *J* = 8.2 Hz, 6"-CH), 7.12-7.05 (2H, *m*, 3"-CH, 5"-CH), 5.97 (1H, *d*, *J* = 5.6 Hz, 5'-CH), 5.30 (2H, *s*, 12-CH_2), 3.76-3.65 (4H, *m*, 3-CH_2, 5-CH_2), 3.47-3.35 (4H, *m*, 2-CH_2, 6-CH_2), 1.42 (9H, *s*, 9-CH_3, 10-CH_3, 11-CH_3). **^{13}C NMR (101 MHz, $CDCl_3$, *δ ppm*)**: 168.96 (C-4'), 161.44 (C-2'), 160.46 (C-2", *d*, $J_{(C\text{-}F)}$ = 250.8 Hz), 158.32 (C-6'), 154.84 (C-7), 134.77 (C-4", *d*, $J_{(C\text{-}F)}$ = 10.2 Hz), 130.87 (C-6", *d*, $J_{(C\text{-}F)}$ = 4.9 Hz), 124.63 (C-5", *d*, $J_{(C\text{-}F)}$ = 3.7 Hz), 122.77 (C-1", *d*, $J_{(C\text{-}F)}$ = 14.6 Hz), 116.24 (C-3", *d*, $J_{(C\text{-}F)}$ = 24.8 Hz), 97.01 (C-5'), 79.99 (C-8), 60.33 (C-12, *d*, $J_{(C\text{-}F)}$ = 4.1 Hz), 43.69 (C-2, C-3, C-5, C-6, *br.s.*), 28.44 (C-9, C-10, C-11). **HRMS (ESI+)**: found *m/z* 423.1590 $[M + H]^+$; calculated $C_{20}H_{25}{}^{35}ClFN_4O_3^+$ 423.1521.

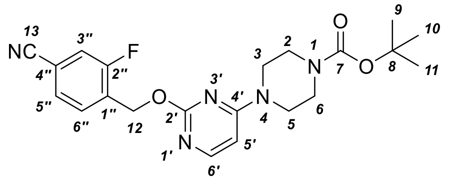

tert-Butyl 4-(2'-((4''-cyano-2''-fluorobenzyl)oxy)pyrimidin-4'-yl)piperazine-1-carboxylate **21c'**, white solid, 550 mg. **^{1}H NMR (400 MHz, CDCl$_3$, *δ ppm*)**: 7.98 (1H, *d*, *J* = 6.1 Hz, 6'-CH), 7.60 (1H, *t*, *J* = 7.5 Hz, 6''-CH), 7.38 (1H, *dd*, *J* = 7.9, 1.5 Hz, 5''-CH), 7.29 (1H, *dd*, *J* = 9.3, 1.5 Hz, 3''-CH), 6.13 (1H, *d*, *J* = 6.1 Hz, 5'-CH), 5.41 (2H, *s*, 12-CH$_2$), 3.56 (4H, *dd*, *J* = 6.9, 3.9 Hz, 3-CH$_2$, 5-CH$_2$), 3.44 (4H, *dd*, *J* = 6.7, 3.9 Hz, 2-CH$_2$, 6-CH$_2$), 1.42 (9H, *s*, 9-CH$_3$, 10-CH$_3$, 11-CH$_3$). **^{13}C NMR (101 MHz, CDCl$_3$, *δ ppm*)**: 164.2 (C-2'), 163.5 (C-4'), 159.7 (C-2'', *d*, $J_{(C\text{-}F)}$ = 250.7 Hz), 157.7 (C-6'), 154.6 (C-7), 130.8 (C-1'', *d*, $J_{(C\text{-}F)}$ = 14.5 Hz), 130.4 (C-6'', *d*, $J_{(C\text{-}F)}$ = 4.5 Hz), 128.2 (C-5'', *d*, $J_{(C\text{-}F)}$ = 4.1 Hz), 118.8 (C-3'', *d*, $J_{(C\text{-}F)}$ = 24.7 Hz), 117.5 (C-13, *d*, $J_{(C\text{-}F)}$ = 2.9 Hz), 112.8 (C-4'', *d*, $J_{(C\text{-}F)}$ = 9.4 Hz), 97.8 (C-5'), 80.4 (C-8), 60.3 (C-12, *d*, $J_{(C\text{-}F)}$ = 4.9 Hz), 43.7 (C-2, C-3, C-5, C-6, *br.s.*), 28.4 (C-9, C-10, C-11). **HRMS (ESI+)**: found *m/z* 414.1925 [M + H]$^+$; calculated $C_{21}H_{25}FN_5O_3^+$ 414.1863.

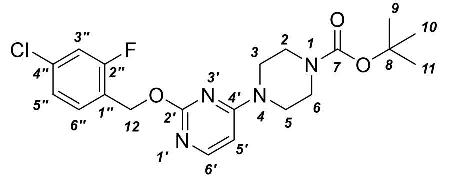

tert-Butyl 4-(2'-((4''-cyano-2''-fluorobenzyl)oxy)pyrimidin-4'-yl)piperazine-1-carboxylate **21e'**, white solid, 620 mg. **^{1}H NMR (400 MHz, CDCl$_3$, *δ ppm*)**: 7.98 (1H, *d*, *J* = 6.0 Hz, 6'-CH), 7.40 (1H, *t*, *J* = 8.0 Hz, 6''-CH), 7.09-6.96 (2H, *m*, 3''-CH, 5''-CH), 6.11 (1H, *d*, *J* = 6.1 Hz, 5'-CH), 5.32 (2H, *s*, 12-CH$_2$), 3.56 (4H, *dd*, *J* = 6.7, 3.9 Hz, 3-CH$_2$, 5-CH$_2$), 3.43 (4H, *dd*, *J* = 6.7, 3.8 Hz, 2-CH$_2$, 6-CH$_2$), 1.42 (9H, *s*, 9-CH$_3$, 10-CH$_3$, 11-CH$_3$). **^{13}C NMR (101 MHz, CDCl$_3$, *δ ppm*)**: 164.5 (C-2'), 163.5 (C-4'), 160.4 (C-2'', *d*, $J_{(C\text{-}F)}$ = 250.8 Hz), 157.7 (C-6'), 154.6 (C-7), 134.4 (C-4'', *d*, $J_{(C\text{-}F)}$ = 10.2 Hz), 130.9 (C-6'', *d*, $J_{(C\text{-}F)}$ = 4.8 Hz), 124.5 (C-5'', *d*, $J_{(C\text{-}F)}$ = 3.6 Hz), 123.2 (C-1'', *d*, $J_{(C\text{-}F)}$ = 14.5 Hz), 116.0 (C-3', *d*, $J_{(C\text{-}F)}$ = 24.8 Hz), 97.5 (C-5'), 80.3 (C-8), 61.5 (C-12, *d*, $J_{(C\text{-}F)}$ = 4.3 Hz), 43.7 (C-2, C-3, C-5, C-6, *br.s.*), 28.4 (C-9, C-10, C-11). **HRMS (ESI+)**: found *m/z* 423.1593 [M + H]$^+$; calculated $C_{20}H_{25}{}^{35}ClFN_4O_3^+$ 423.1521.

General procedure to synthesis of target 2,4-disubstituted pyrimidine derivatives **22a-e**, **22c'**, **22e'**. A weighted portion of corresponding compound **21a-e**, **21c'**, **21e'** (1 eq) was dissolved in DCM (40 ml) in a round bottom flask under vigorous stirring, TFA (30 eq) was added to the resulting solution. The reaction mixture was refluxed for 2 h, the reaction was controlled by TLC (DCM (sat. NH_3)). After the reaction was completed, the solution was diluted with DCM (20 ml), washed sequentially with saturated aqueous Na_2CO_3 solution (1×20 ml), deionized water (2×20 ml), brine solution (1×20 ml). The combined organic layer was dried with Na_2SO_4 under vigorous stirring for 1 h, after which the precipitate was filtered off, the solvent was evaporated on a rotary evaporator under reduced pressure to form the corresponding 2,4-disubstituted pyrimidine derivative **22a-e**, **22c'**, **22e'**.

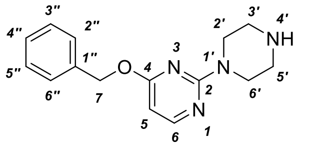

4-(Benzyloxy)-2-(piperazin-1-yl)pyrimidine **22a**, yellow solid, 2.5 g, 90%. **^{1}H NMR (400 MHz, CDCl$_3$, *δ ppm*)**: 8.00 (1H, *d*, *J* = 5.6 Hz, 6-CH), 7.37-7.22 (5H, *m*, 2''-CH, 3''-CH, 4''-CH, 5''-CH, 6''-CH), 5.96 (1H, *d*, *J* = 5.6 Hz, 5-CH), 5.27 (2H, *s*, 7-CH$_2$), 3.74-3.71 (4H, *m*, 2'-CH$_2$, 6'-CH$_2$), 2.87-2.84 (4H, *m*, 3'-CH$_2$, 5'-CH$_2$). **^{13}C NMR (101 MHz, CDCl$_3$, *δ ppm*)**: 168.3 (C-4), 160.6 (C-2), 157.1 (C-6), 135.8 (C-1''), 127.5 (C-2'', C-6'' or C-3'', C-5''), 127.0 (C-4''), 126.9 (C-3'', C-5'' or C-2'', C-6''), 95.8 (C-5), 66.2 (C-7), 44.8 (C-2', C-6'), 43.8 (C-3', C-5'). **UPLC-MS (ESI+)**: found *m/z* 271.1 [M + H]$^+$; calculated $C_{15}H_{19}N_4O^+$ 271.3.

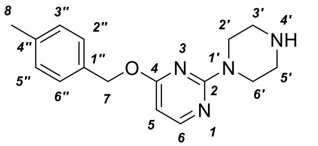

4-((4-Methylbenzyl)oxy)-2-(piperazin-1-yl)pyrimidine **22b**, yellow solid, 860 mg, 90%. **^{1}H NMR (400 MHz, CDCl$_3$, *δ ppm*)**: 8.01 (1H, *d*, *J* = 5.7 Hz, 6-CH), 7.23-7.20 (2H, *m*, 2''-CH, 6''-CH), 7.18-7.10 (2H, *m*, 3''-CH, 5''-CH), 6.06 (1H, *d*, *J* = 5.6 Hz, 5-CH), 5.22 (2H, *s*, 7-CH$_2$), 4.05-4.02 (4H, *m*, 2'-CH$_2$, 6'-CH$_2$), 3.14-3.11 (4H, *m*, 3'-CH$_2$, 5'-CH$_2$), 2.28 (3H, *s*, 8-CH$_3$). **^{13}C NMR (101 MHz, CDCl$_3$, *δ ppm*)**: 168.7 (C-4), 159.7 (C-2), 157.0 (C-6), 137.0 (C-4''), 132.3 (C-1''), 128.2 (C-3'', C-5''), 126.9 (C-2'', C-6''), 97.7 (C-5), 66.7 (C-7), 42.1 (C-2', C-6'), 39.9 (C-3', C-5'), 20.2 (C-8). **UPLC-MS (ESI+)**: found *m/z* 285.1 [M + H]$^+$; calculated $C_{16}H_{21}N_4O^+$ 285.2.

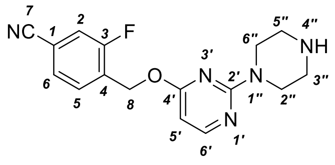

3-Fluoro-4-(((2-(piperazin-1-yl)pyrimidin-4-yl)oxy)methyl)benzonitrile **22c**, white solid, 7.6 g, 85%. **^{1}H NMR (400 MHz, CDCl$_3$, *δ ppm*)**: 8.03 (1H, *d*, *J* = 5.6 Hz, 6'-CH), 7.51 (1H, *t*, *J* = 7.5 Hz, 6-CH), 7.39 (1H, *dd*, *J* = 8.0, 1.5 Hz, 5-CH), 7.31 (1H, *dd*, *J* = 9.3, 1.5 Hz, 2-CH), 5.98 (1H, *d*, *J* = 5.6 Hz, 5'-CH), 5.39 (2H, *s*, 8-CH$_2$), 3.75-3.67 (4H, *m*, 2''-CH$_2$, 6''-CH$_2$), 2.85-2.82 (4H, *m*, 3''-CH$_2$, 5''-CH$_2$). **^{13}C NMR (101 MHz, CDCl$_3$, *δ ppm*)**: 168.6 (C-4'), 161.5 (C-2'), 159.7 (C-3, *d*, $J_{(C\text{-}F)}$ = 250.8 Hz), 158.6 (C-6'), 130.4 (C-6, *d*, $J_{(C\text{-}F)}$ = 4.4 Hz), 129.1 (C-4, *d*, $J_{(C\text{-}F)}$ = 14.4 Hz), 128.3 (C-5, *d*, $J_{(C\text{-}F)}$ = 3.9 Hz), 119.0 (C-2, *d*, $J_{(C\text{-}F)}$ = 24.8 Hz), 117.4 (C-7, *d*, $J_{(C\text{-}F)}$ = 2.9 Hz), 113.0 (C-1, *d*, $J_{(C\text{-}F)}$ = 9.5 Hz), 96.4 (C-5'), 59.9 (C-8, *d*, $J_{(C\text{-}F)}$ = 4.6 Hz), 45.8 (C-3'', C-5''), 44.8 (C-2'', C-6''). **HRMS (ESI+)**: found *m/z* 313.8694 [M + H]$^+$; calculated $C_{16}H_{17}FN_5O^+$ 314.1339.

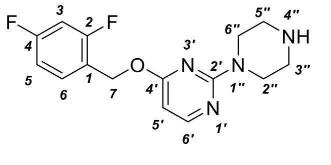

4-((2,4-Difluorobenzyl)oxy)-2-(piperazin-1-yl)pyrimidine **22d**, white solid, 8.1 g, 85%. **^{1}H NMR (400 MHz, $CDCl_3$, *δ ppm*)**: 8.00 (1H, *d*, *J* = 5.6 Hz, 6'-CH), 7.35 (1H, *td*, *J* = 8.4, 6.3 Hz, 6-CH), 7.00-6.64 (2H, *m*, 5-CH, 3-CH), 5.94 (1H, *d*, *J* = 5.6 Hz, 5'-CH), 5.29 (2H, *s*, 7-CH_2), 3.81-3.71 (4H, *m*, 2"-CH_2, 6"-CH_2), 2.87-2.84 (4H, *m*, 2"-CH_2, 6"-CH_2). **^{13}C NMR (101 MHz, $CDCl_3$, *δ ppm*)**: 169.0 (C-4'), 162.8 (C-2, *dd*, $J_{(C-F)}$ = 249.2, 12.2 Hz), 161.6 (C-2'), 160.9 (C-4, *dd*, $J_{(C-F)}$ = 250.4, 12.0 Hz), 158.2 (C-6'), 131.3 (C-6, *dd*, $J_{(C-F)}$ = 10.1, 5.7 Hz), 120.1 (C-1, *dd*, $J_{(C-F)}$ = 15.0, 3.8 Hz), 111.3 (C-5, *dd*, $J_{(C-F)}$ = 21.2, 3.7 Hz), 103.9 (C-3, *t*, $J_{(C-F)}$ = 25.4 Hz), 96.7 (C-5'), 60.3 (C-7, *d*, $J_{(C-F)}$ = 3.7 Hz), 45.9 (C-3", C-5"), 44.9 (C-2", C-6"). **HRMS (ESI+)**: found *m/z* 307.1402 $[M + H]^+$; calculated $C_{15}H_{17}F_2N_4O^+$ 307.1292.

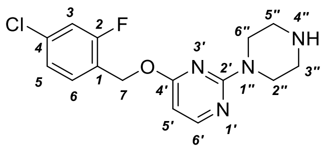

4-((4-Chloro-2-fluorobenzyl)oxy)-2-(piperazin-1-yl)pyrimidine **22e**, white solid, 5.1 g, 85%. **^{1}H NMR (400 MHz, DMSO-d_6, *δ ppm*)**: 8.18 (1H, *d*, *J* = 5.6 Hz, 6'-CH), 7.58 (1H, *t*, *J* = 8.2 Hz, 6-CH), 7.49 (1H, *dd*, *J* = 10.1, 2.1 Hz, 3-CH), 7.33 (1H, *dd*, 5-CH), 6.23 (1H, *d*, *J* = 5.6 Hz, 5'-CH), 5.41 (2H, *s*, 7-CH_2), 3.95 (4H, *t*, *J* = 5.3 Hz, 3"-CH_2, 5"-CH_2), 3.16 (4H, *br. s.*, 2"-CH_2, 6"-CH_2). **^{13}C NMR (101 MHz, DMSO-d_6, *δ ppm*)**: 169.13 (C-4'), 161.00 (C-2'), 160.84 (C-2, *d*, $J_{(C-F)}$ = 250.1 Hz), 159.31 (C-6'), 134.39 (C-4, *d*, $J_{(C-F)}$ = 10.7 Hz), 132.66 (C-6, *d*, $J_{(C-F)}$ = 5.0 Hz), 125.29 (C-5, *d*, $J_{(C-F)}$ = 3.6 Hz), 123.25 (C-1, *d*, $J_{(C-F)}$ = 14.9 Hz), 116.59 (C-3, *d*, $J_{(C-F)}$ = 24.8 Hz), 97.82 (C-5'), 61.03 (C-7, *d*, $J_{(C-F)}$ = 3.2 Hz), 42.85 (C-3", C-5"), 40.88 (C-2", C-6"). **UPLC-MS (ESI+)**: found *m/z* 323.2 $[M + H]^+$; calculated $C_{15}H_{17}{}^{35}ClFN_4O^+$ 323.1.

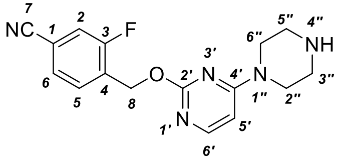

3-Fluoro-4-(((4-(piperazin-1-yl)pyrimidin-2-yl)oxy)methyl)benzonitrile **22c'**, white solid, 350 mg, 85%. **^{1}H NMR (400 MHz, $CDCl_3$, *δ ppm*)**: 8.11 (1H, *d*, *J* = 6.1 Hz, 6'-CH), 7.90 (1H, *dd*, *J* = 10.0, 1.4 Hz, 2-CH), 7.76-7.62 (2H, *m*, 5-CH, 6-CH), 6.60 (1H, *d*, *J* = 6.1 Hz, 5'-CH), 5.43 (2H, *s*, 8-CH_2), 3.79 (4H, *t*, *J* = 5.1 Hz, 2"-CH_2, 6"-CH_2), 3.14 (4H, *t*, *J* = 5.2 Hz, 3"-CH_2, 5"-CH_2). **^{13}C NMR (101 MHz, $CDCl_3$, *δ ppm*)**: 164.2 (C-2'), 163.4 (C-4'), 160.0 (C-3, *d*, $J_{(C-F)}$ = 248.6 Hz), 158.3 (C-6'), 131.5 (C-5, *d*, $J_{(C-F)}$ = 5.0 Hz), 131.0 (C-6, *d*, $J_{(C-F)}$ = 14.6 Hz), 129.3 (C-6, *d*, $J_{(C-F)}$ = 3.9 Hz), 119.8 (C-2, *d*, $J_{(C-F)}$ = 25.4 Hz), 118.0 (C-7, *d*, $J_{(C-F)}$ = 3.3 Hz), 112.7 (C-1, *d*, $J_{(C-F)}$ = 10.1 Hz), 98.9 (C-5'), 61.7 (C-8, *d*, $J_{(C-F)}$ = 3.6 Hz), 43.0 (C-3", C-5"), 41.3 (C-2", C-6"). **UPLC-MS (ESI+)**: found *m/z* 314.0 $[M + H]^+$; calculated $C_{16}H_{17}FN_5O^+$ 314.1.

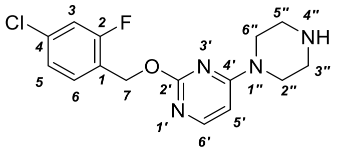

2-((4-Chloro-2-fluorobenzyl)oxy)-4-(piperazin-1-yl)pyrimidine **22e'**, white solid, 400 mg, 85%. **^{1}H NMR (400 MHz, DMSO-d_6, *δ ppm*)**: 8.13 (1H, *d*, *J* = 6.2 Hz, 6'-CH), 7.56 (1H, *t*, *J* = 8.2 Hz, 6-CH), 7.49 (1H, *dd*, *J* = 10.0, 2.1 Hz, 3-CH), 7.33 (1H, *dd*, *J* = 8.2, 2.1 Hz, 5-CH), 6.64 (1H, *d*, *J* = 6.2 Hz, 5'-CH), 5.37 (2H, *s*, 7-CH_2), 3.85 (4H, *t*, *J* = 5.3 Hz, 3"-CH_2, 5"-CH_2), 3.20-3.16 (4H, *m*, 2"-CH_2, 6"-CH_2). **^{13}C NMR (101 MHz, DMSO-d_6, *δ ppm*)**: 163.6 (C-4'), 163.3 (C-2'), 160.8 (C-2, *d*, $J_{(C-F)}$ = 250.0 Hz), 157.0 (C-6'), 134.2 (C-4, *d*, $J_{(C-F)}$ = 10.7 Hz), 132.5 (C-6, *d*, $J_{(C-F)}$ = 5.1 Hz), 125.2 (C-5, *d*, $J_{(C-F)}$ = 3.5 Hz), 123.5 (C-1, *d*, $J_{(C-F)}$ = 15.2 Hz), 116.6 (C-3, *d*, $J_{(C-F)}$ = 25.2 Hz), 98.8 (C-5'), 62.0 (C-7, *d*, $J_{(C-F)}$ = 3.5 Hz), 42.8 (C-3", C-5"), 41.2 (C-2", C-6"). **UPLC-MS (ESI+)**: found *m/z* 323.2 $[M + H]^+$; calculated $C_{15}H_{17}{}^{35}ClFN_4O^+$ 323.1.

^{1}H NMR spectrum of compound **20a**

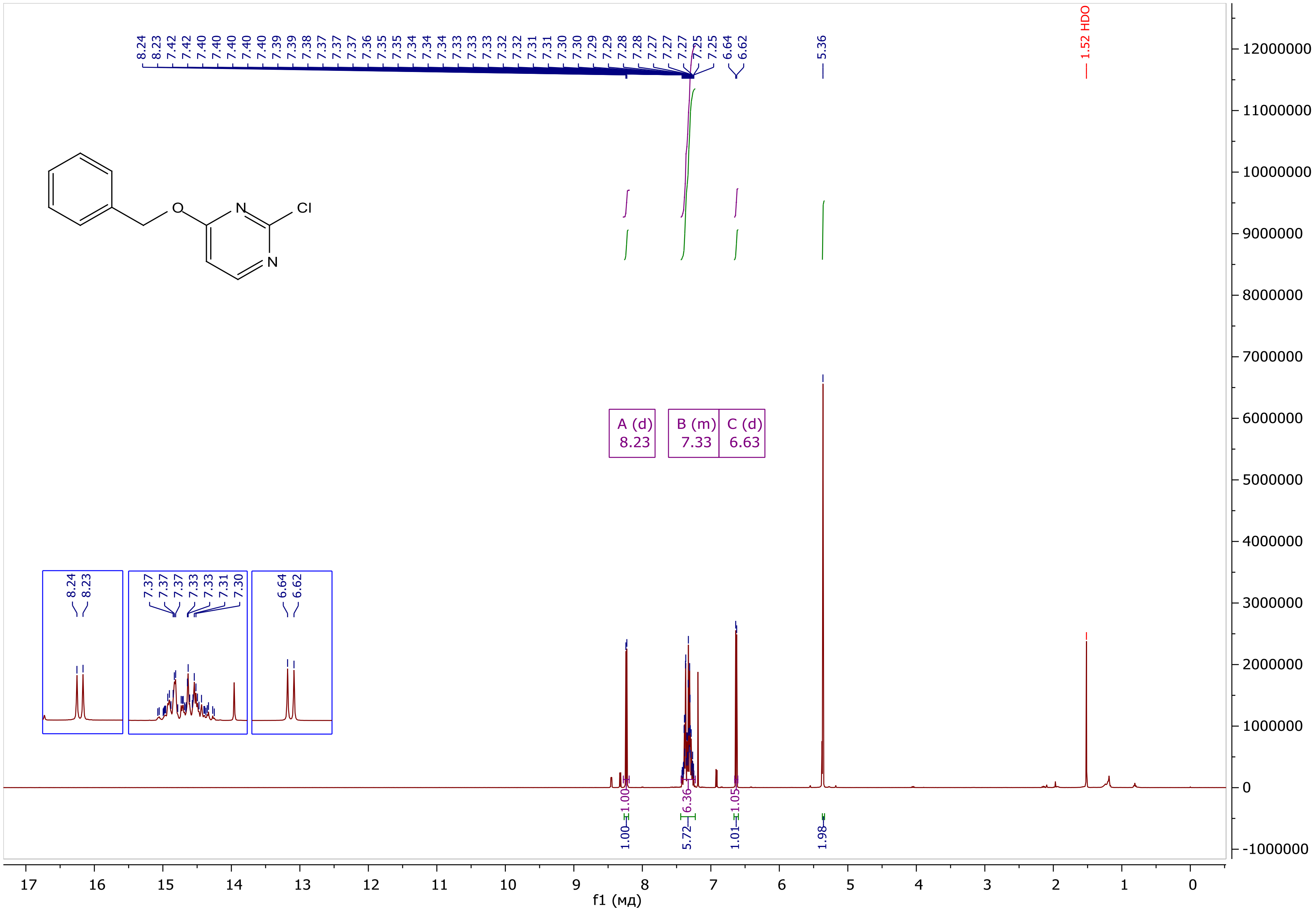

^{13}C NMR spectrum of compound **20a**

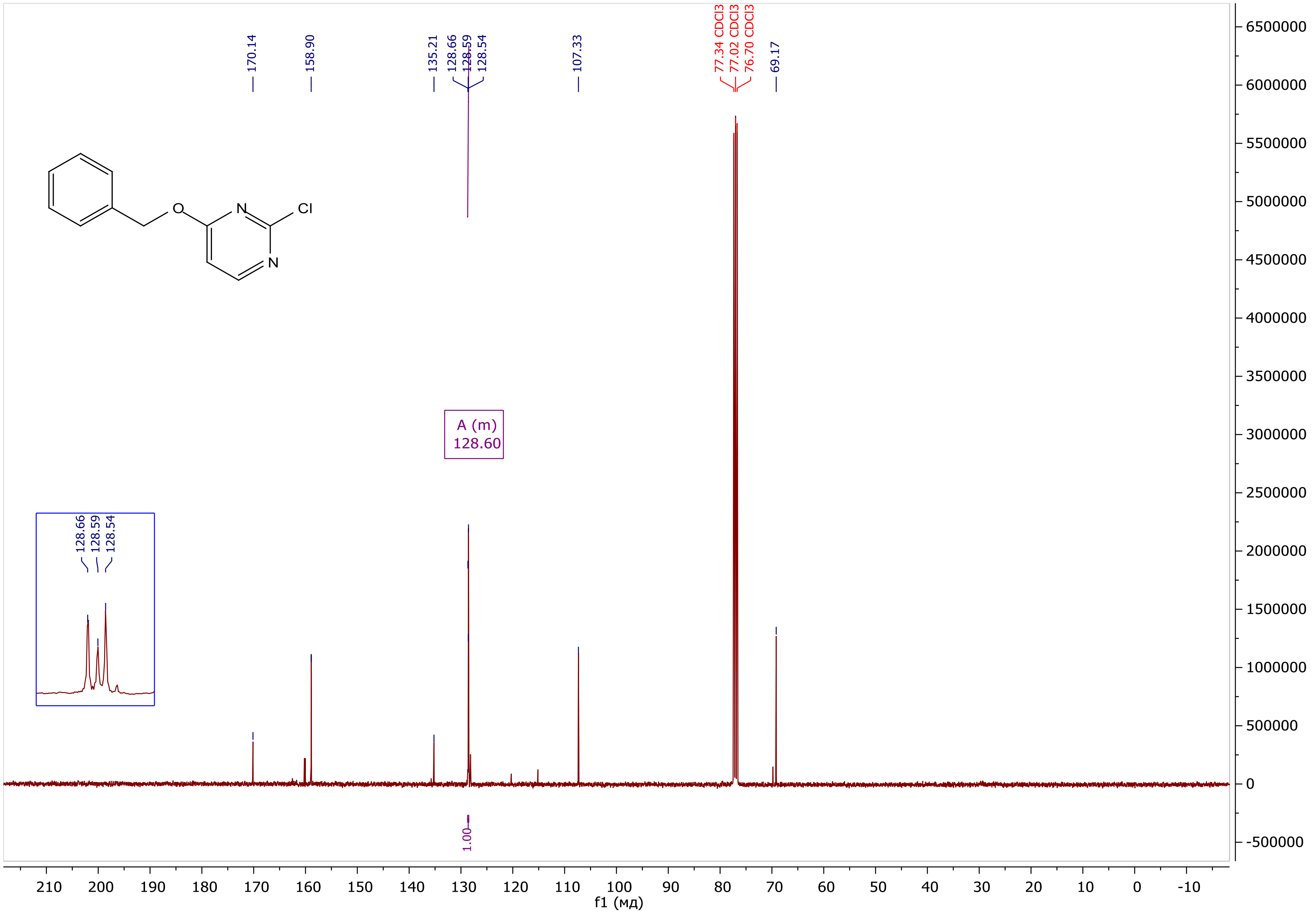

^{1}H NMR spectrum of compound **20b**

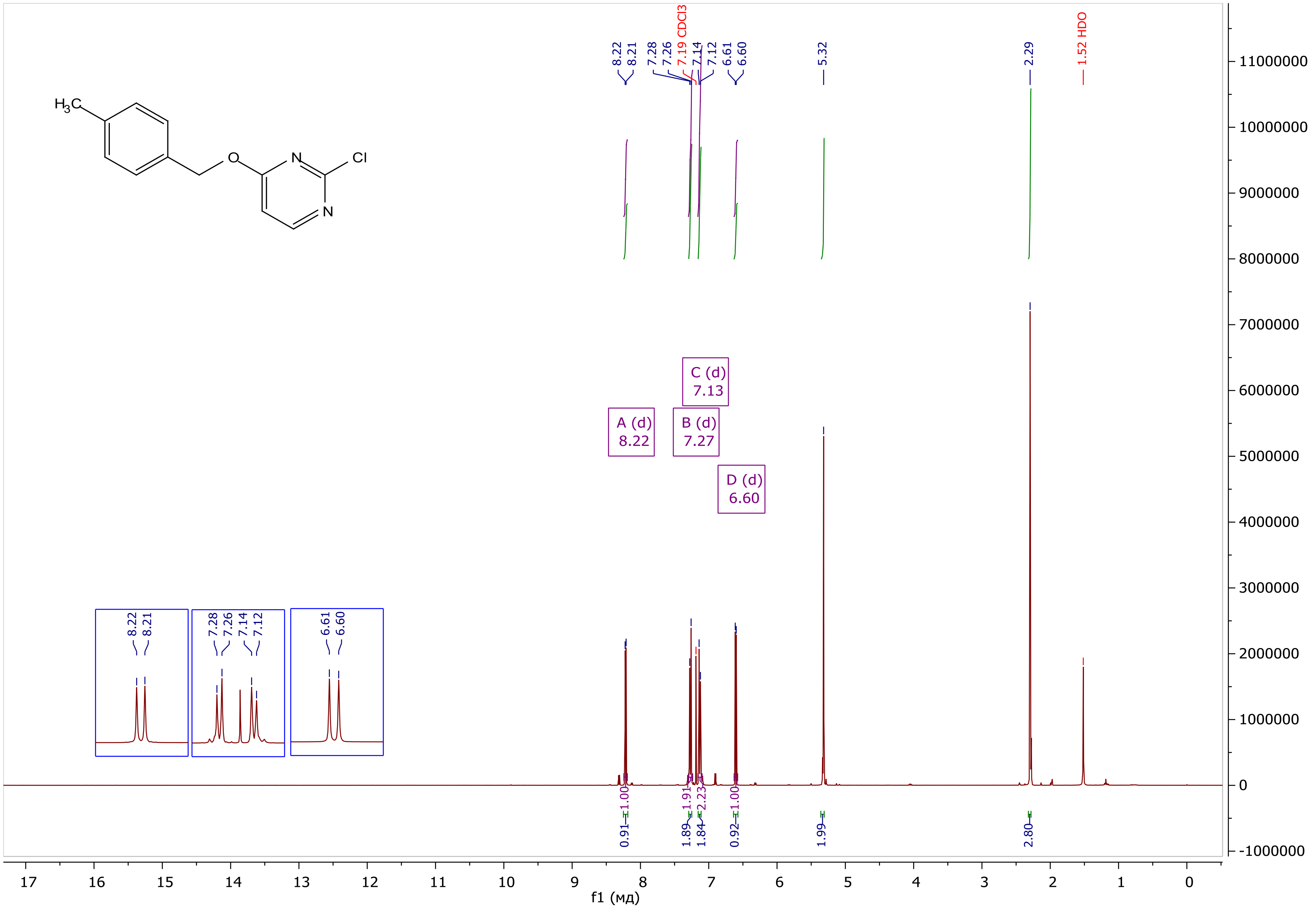

^{13}C NMR spectrum of compound **20b**

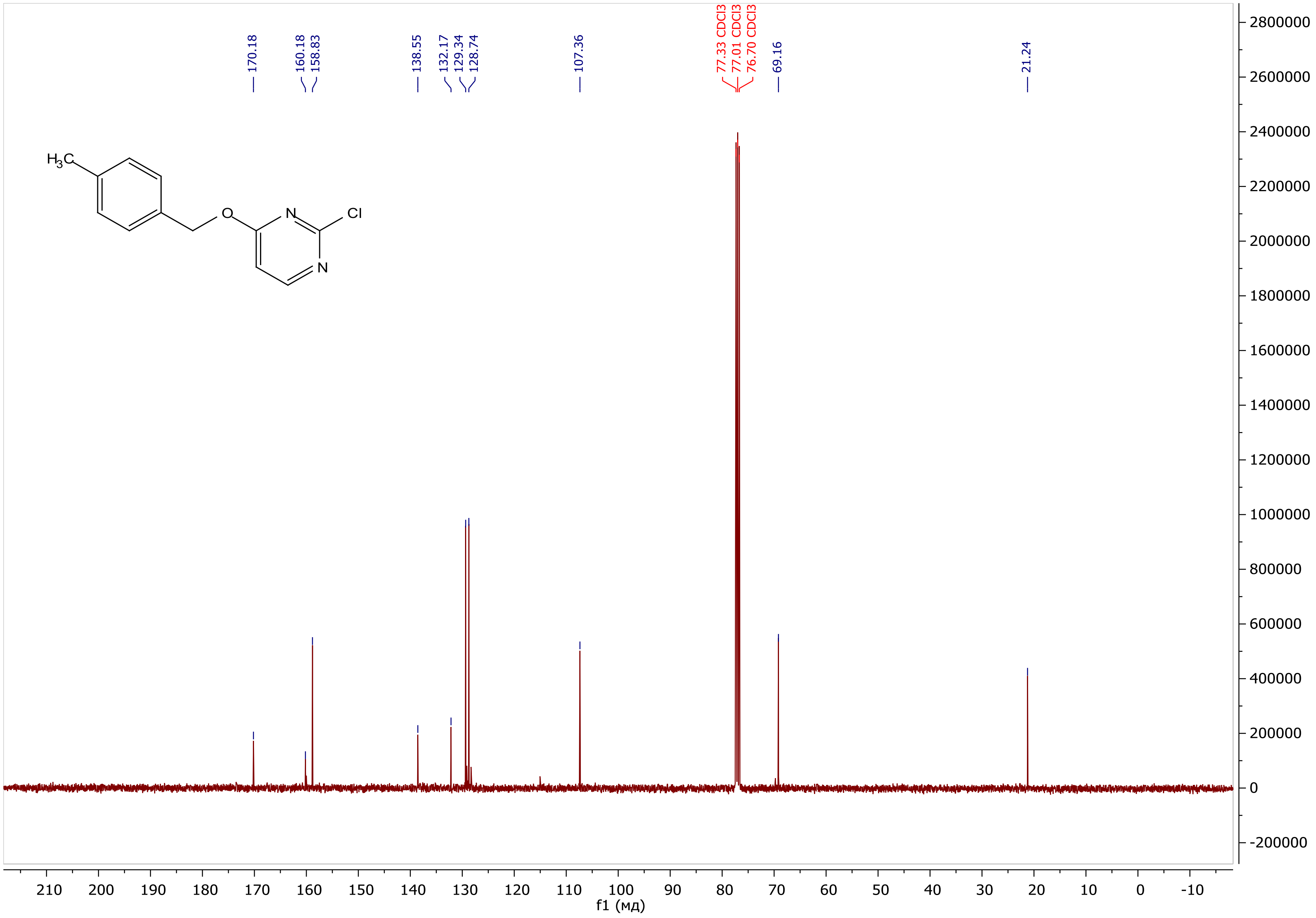

^{1}H NMR spectrum of compound **20c**

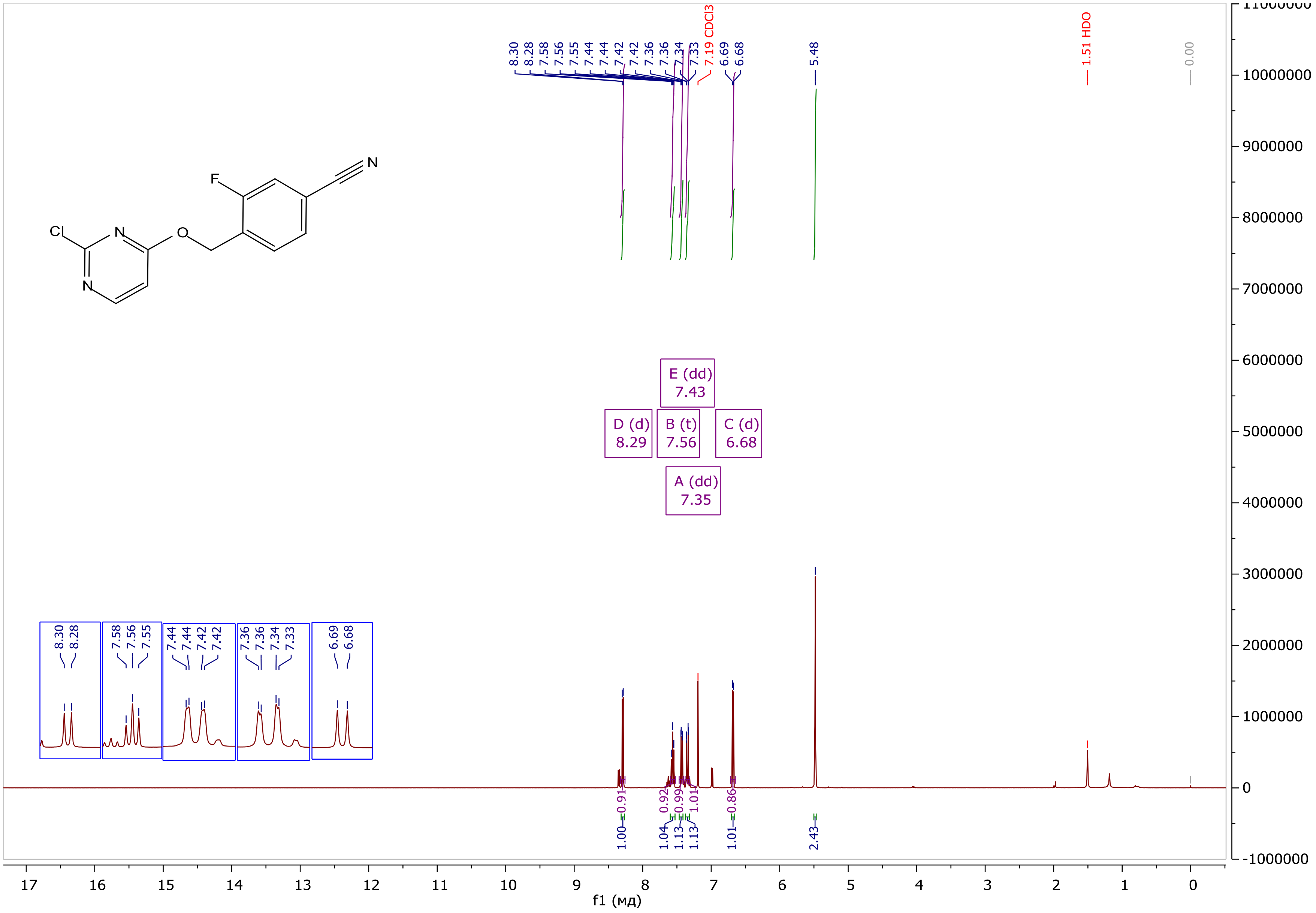

^{13}C NMR spectrum of compound **20c**

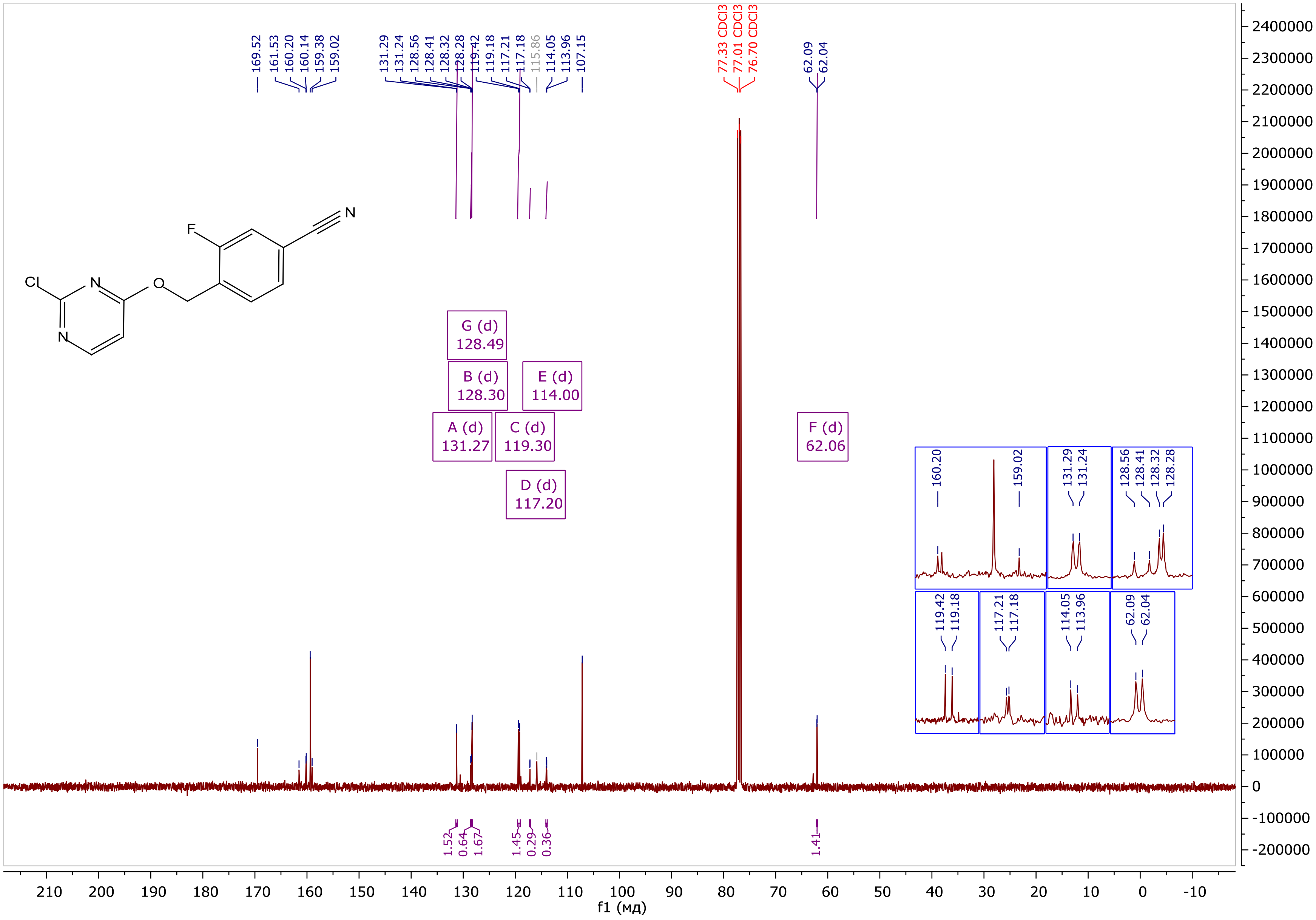

^{1}H NMR spectrum of compound **20d**

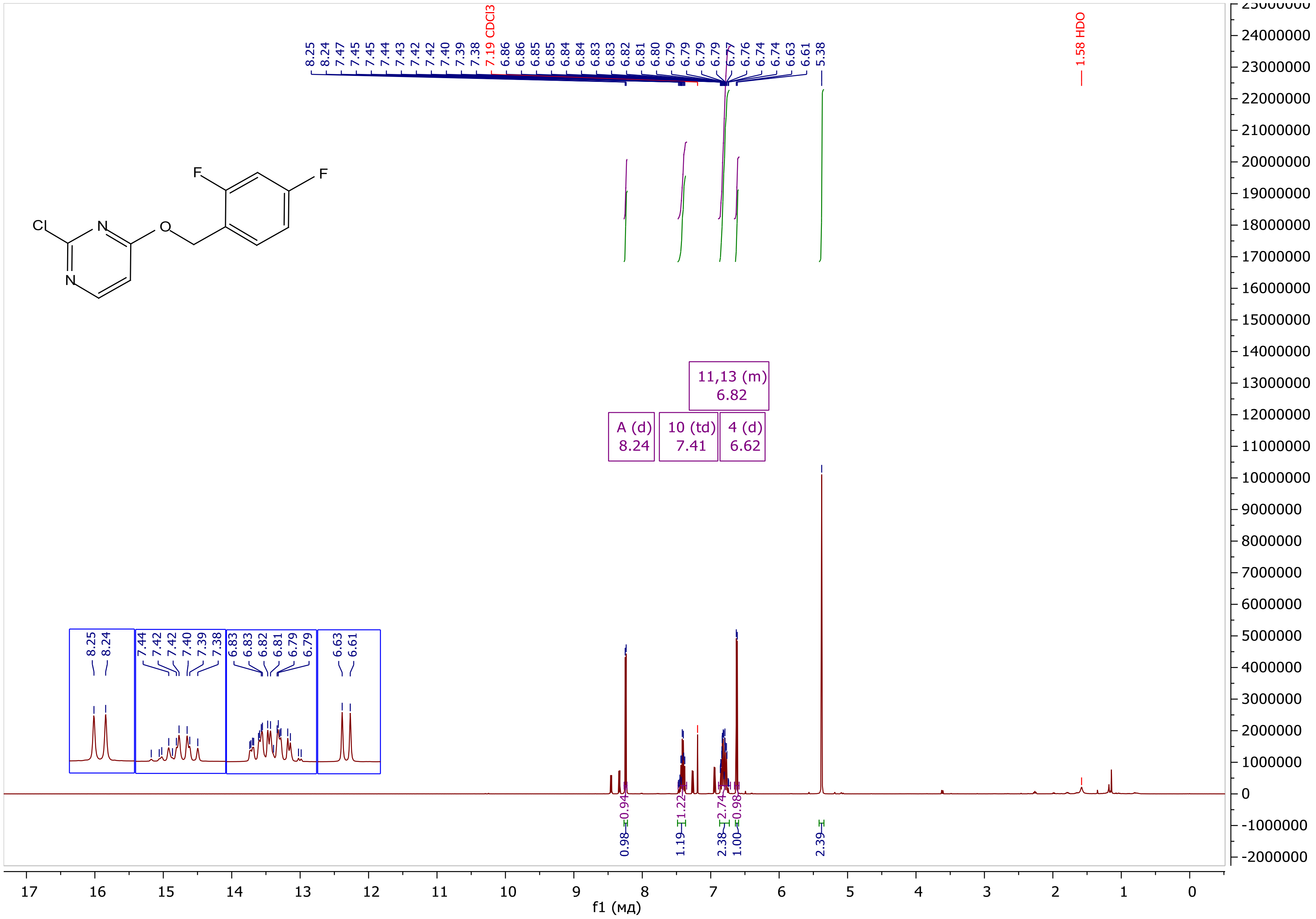

^{13}C NMR spectrum of compound **20d**

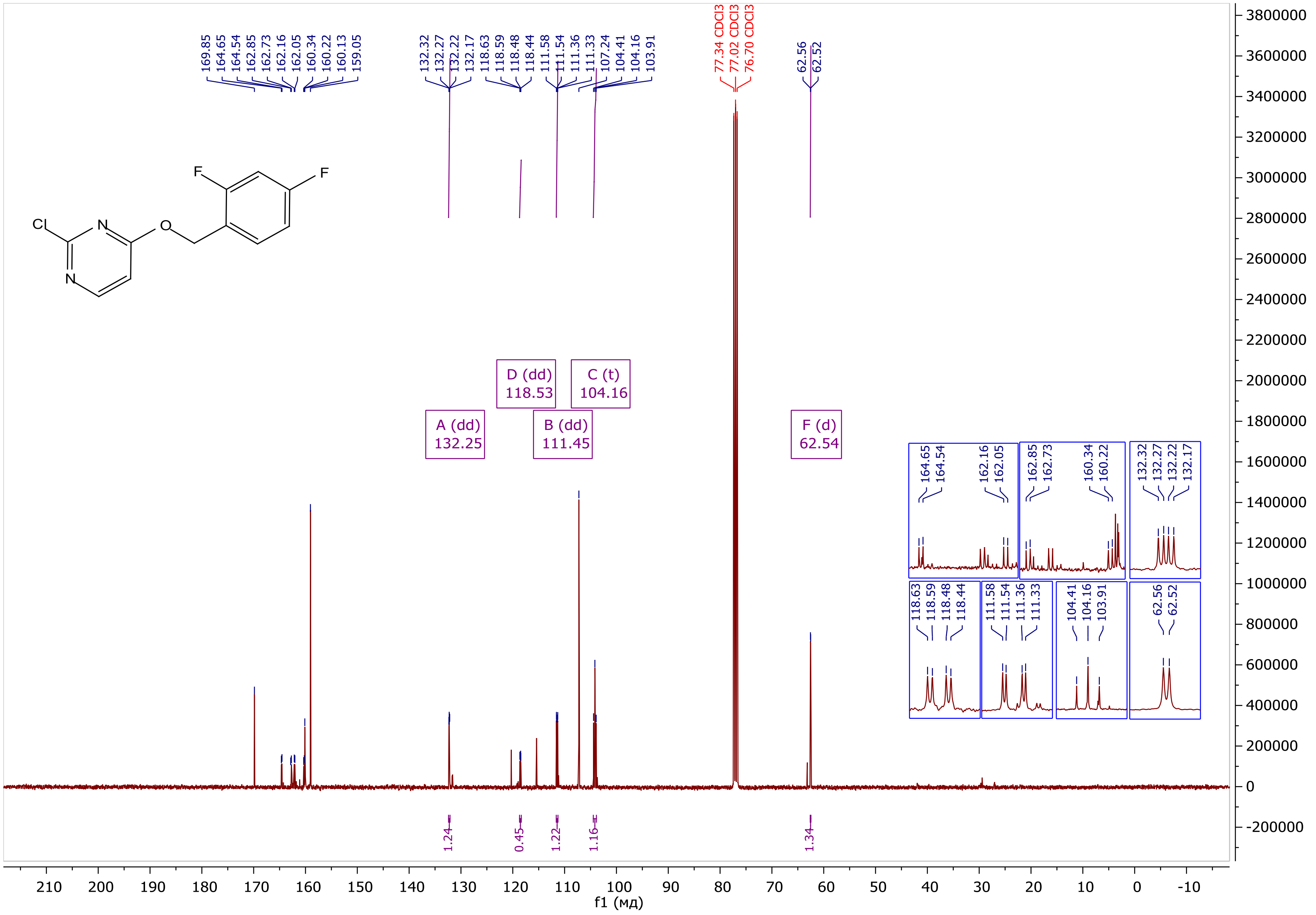

^{1}H NMR spectrum of compound **20e**

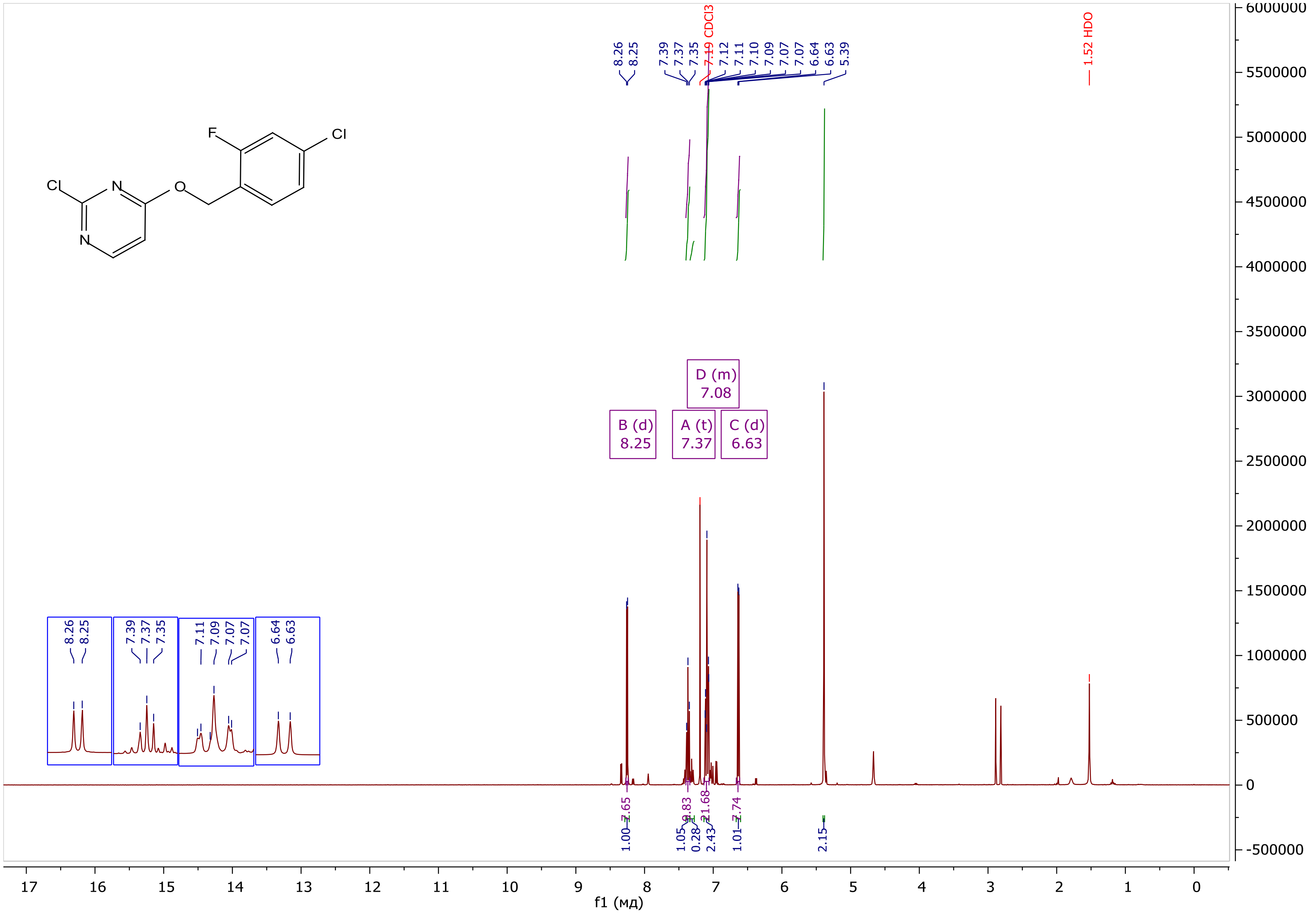

^{13}C NMR spectrum of compound **20e**

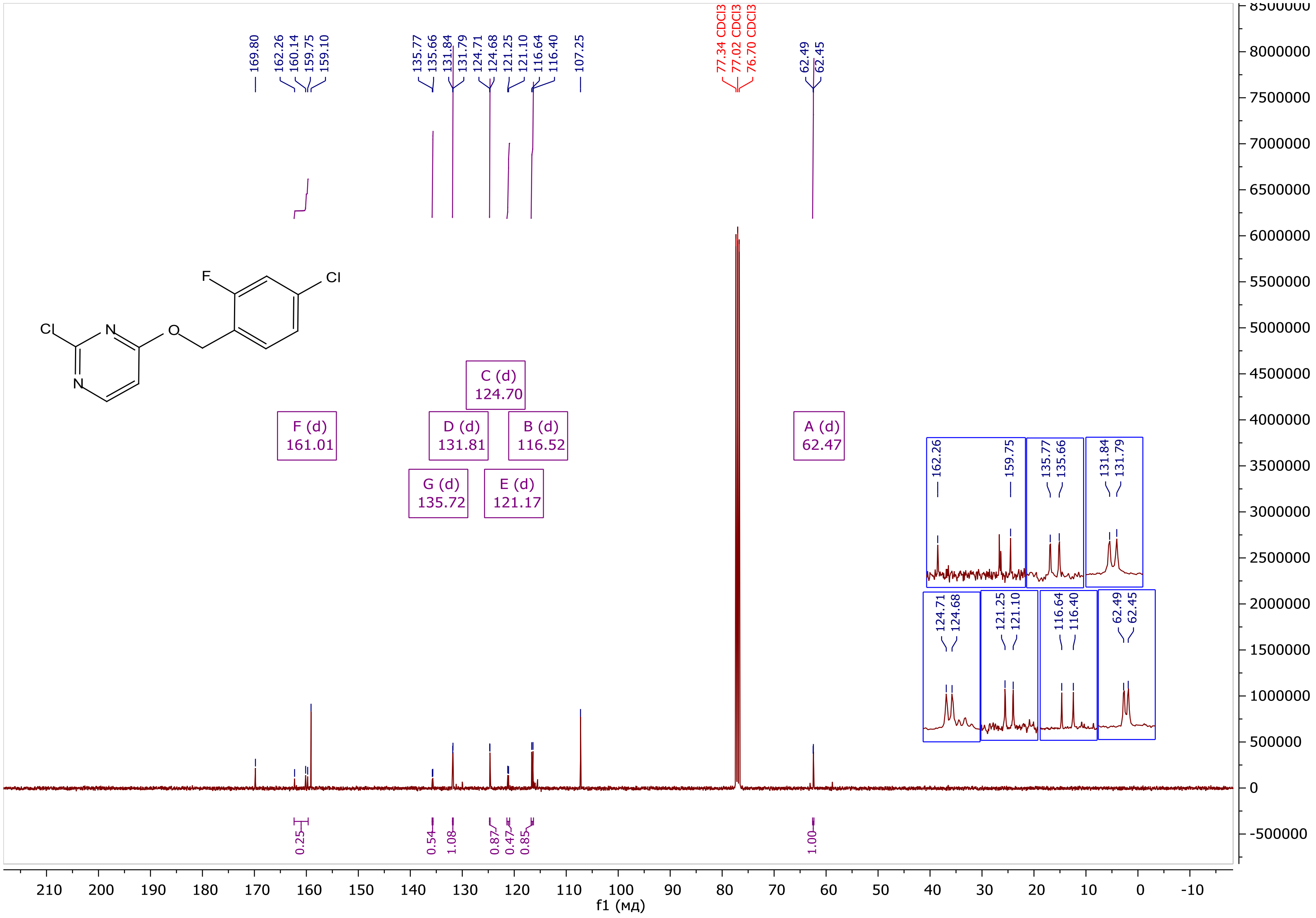

^{1}H NMR spectrum of compound **21a**

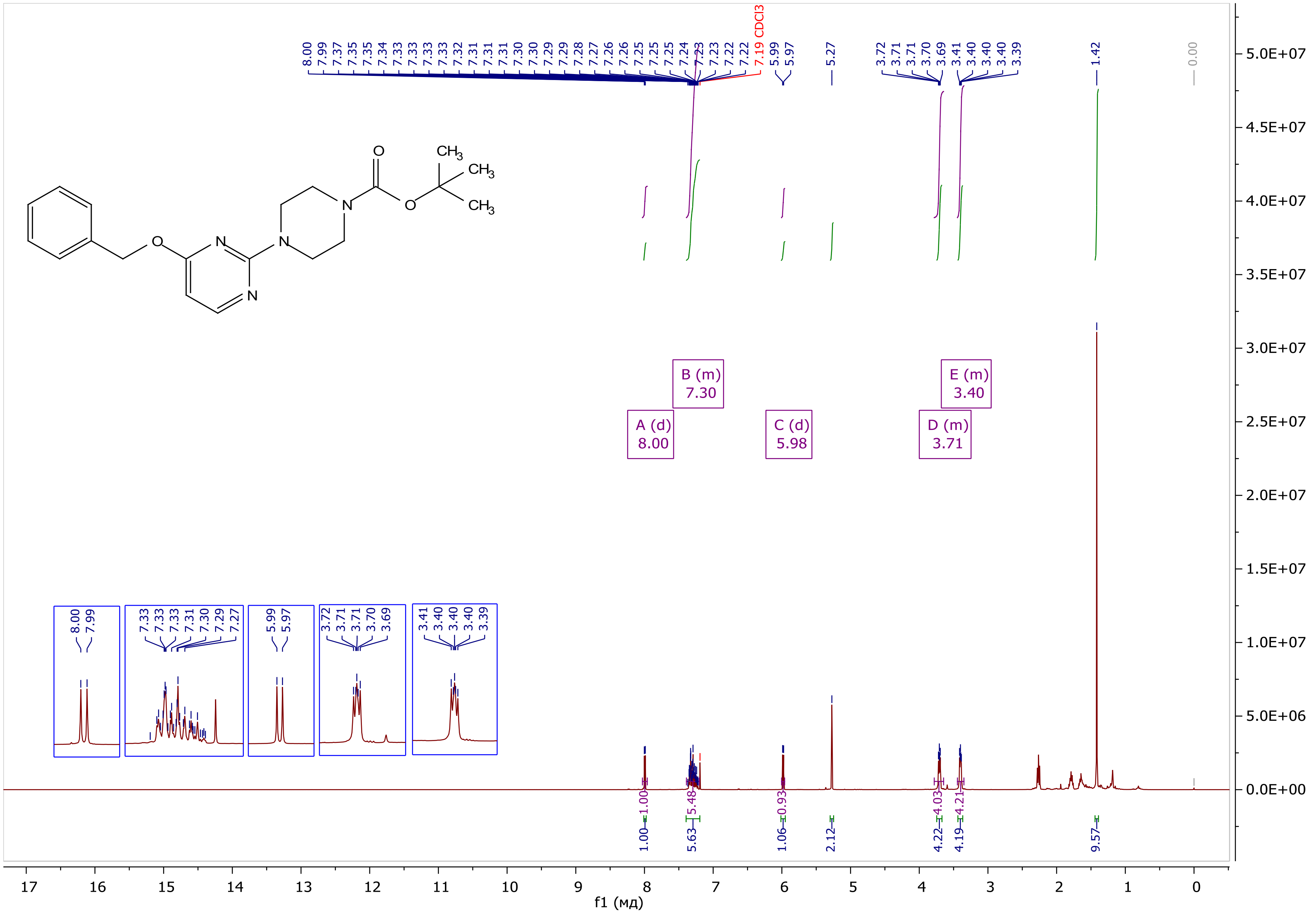

^{13}C NMR spectrum of compound **21a**

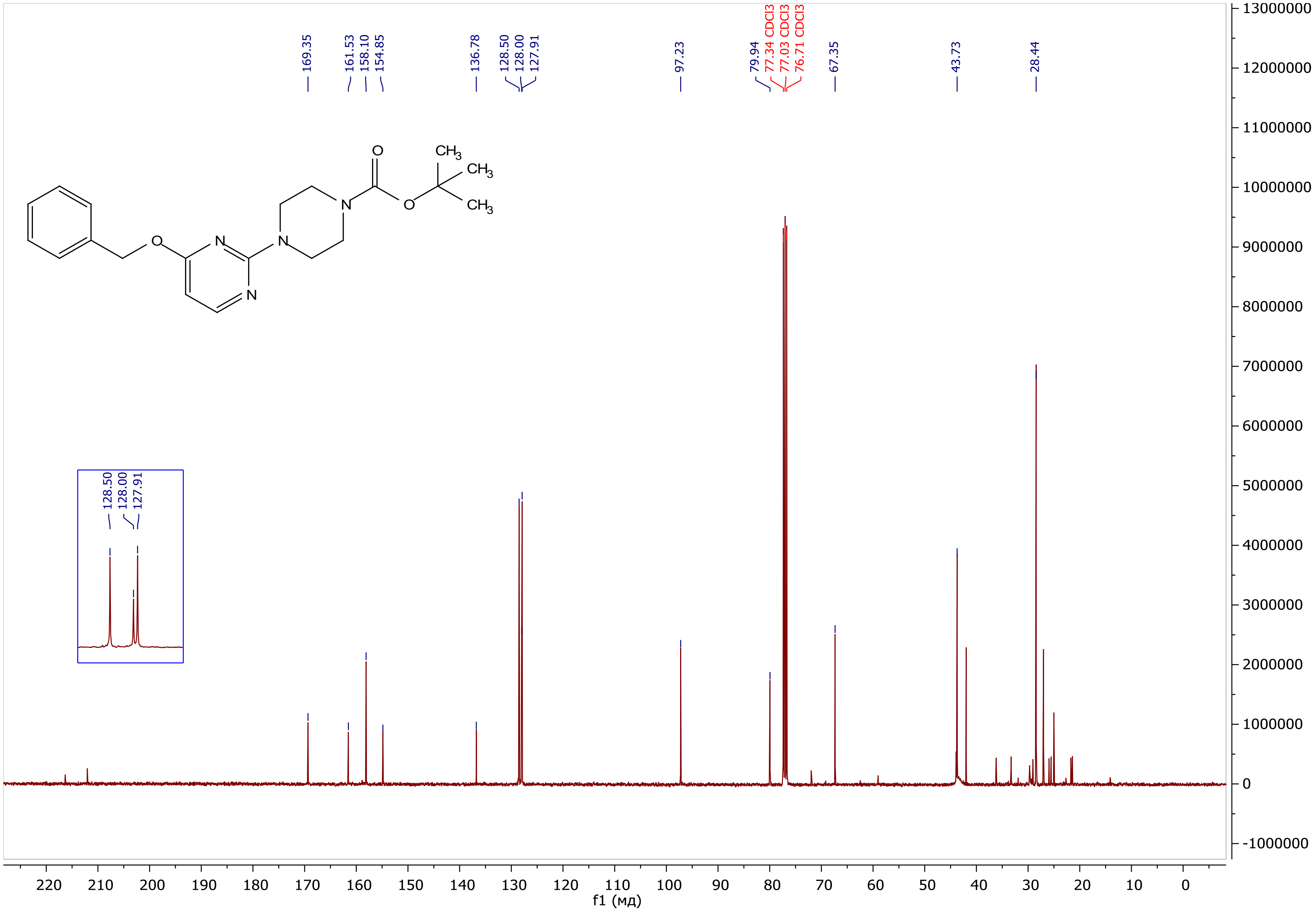

^{1}H NMR spectrum of compound **21b**

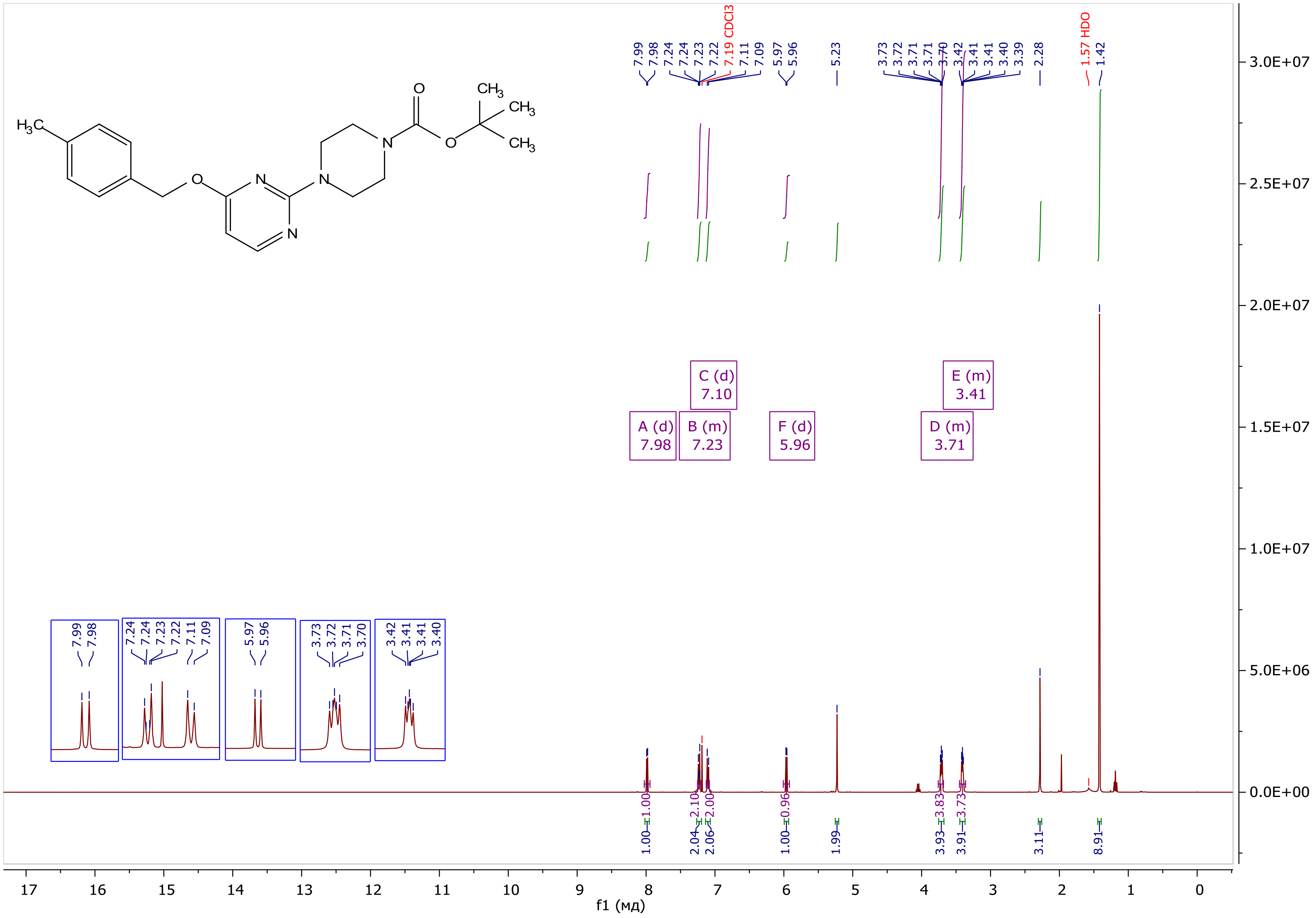

^{13}C NMR spectrum of compound **21b**

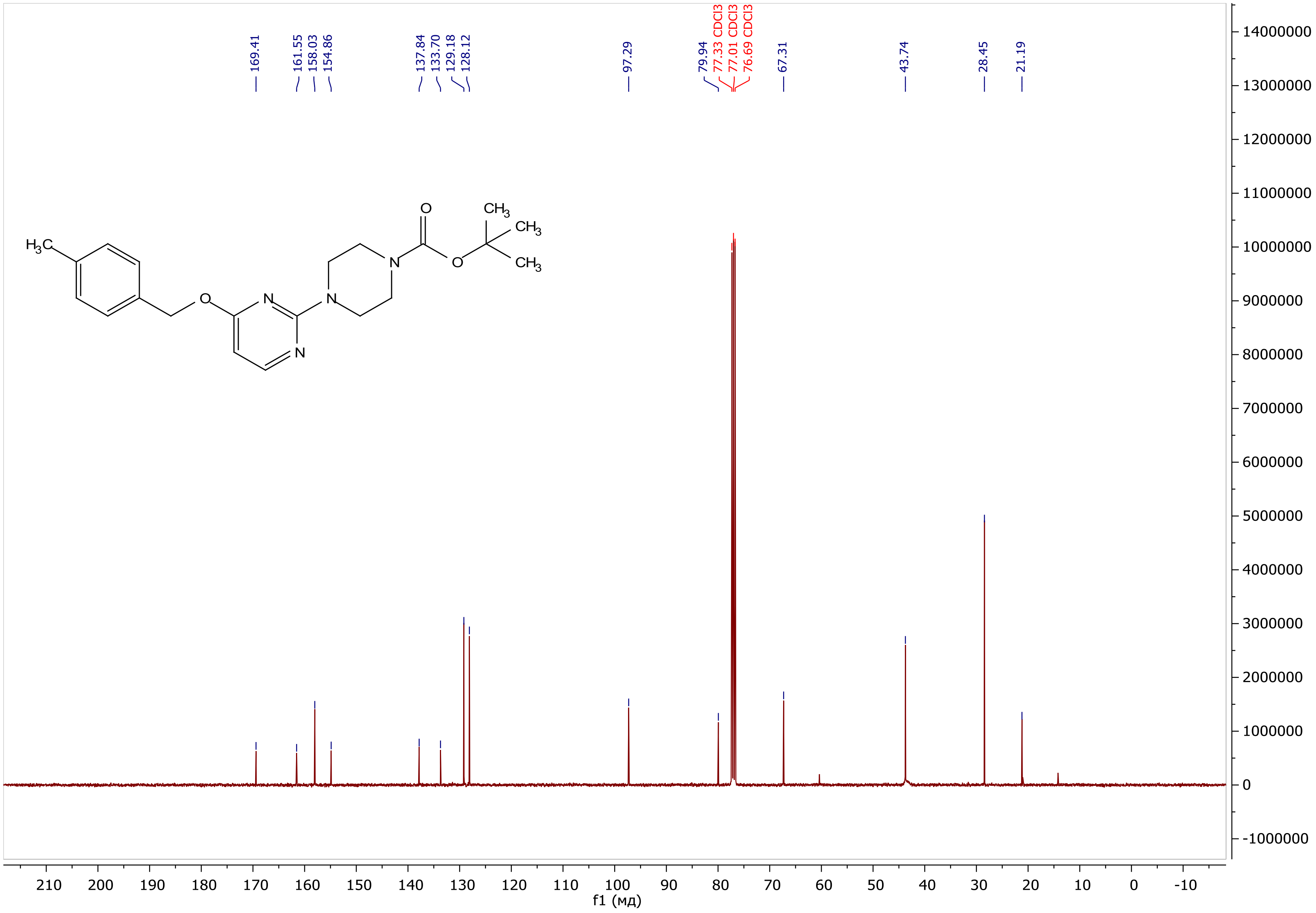

^{1}H NMR spectrum of compound **21c**

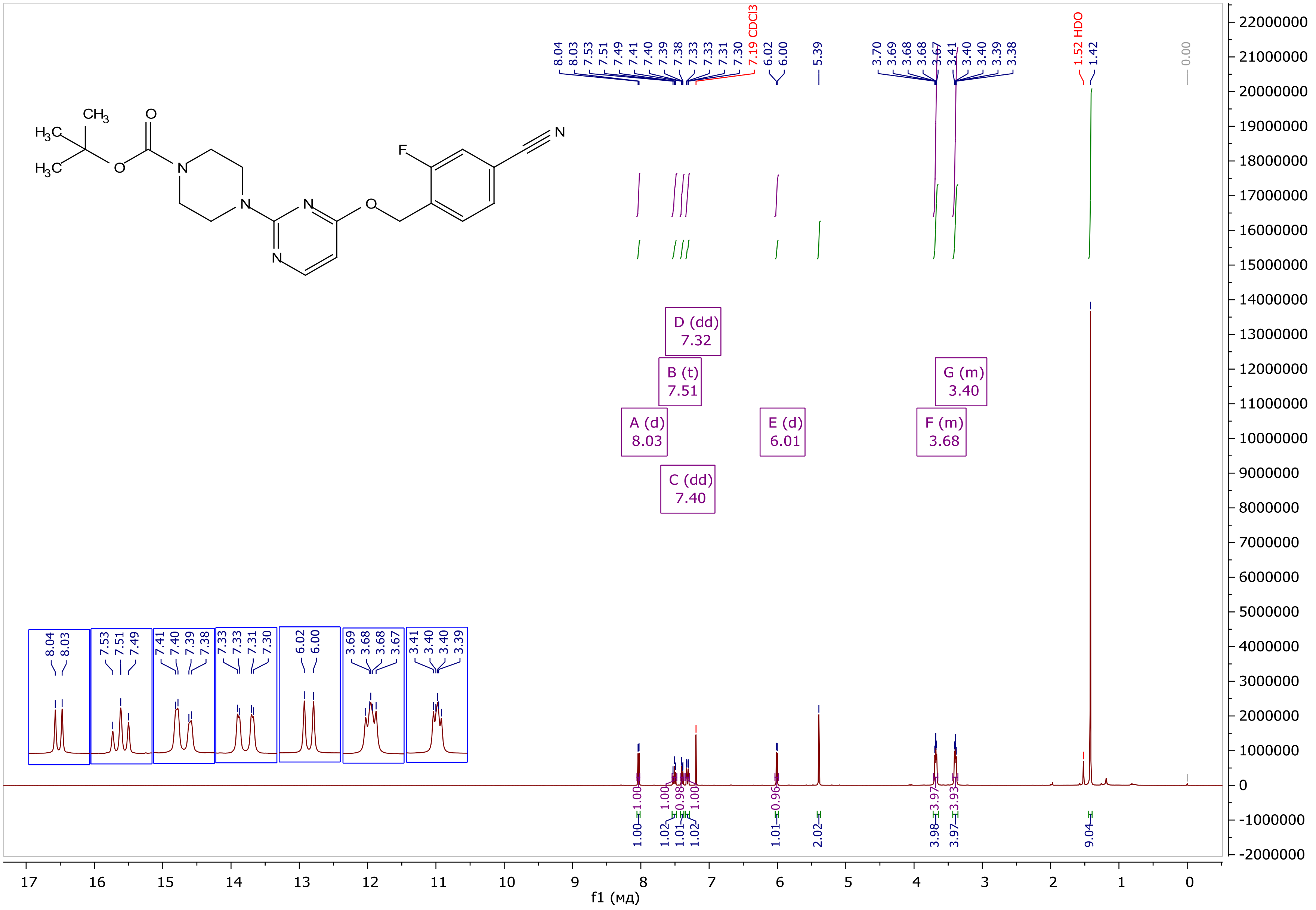

^{13}C NMR spectrum of compound **21c**

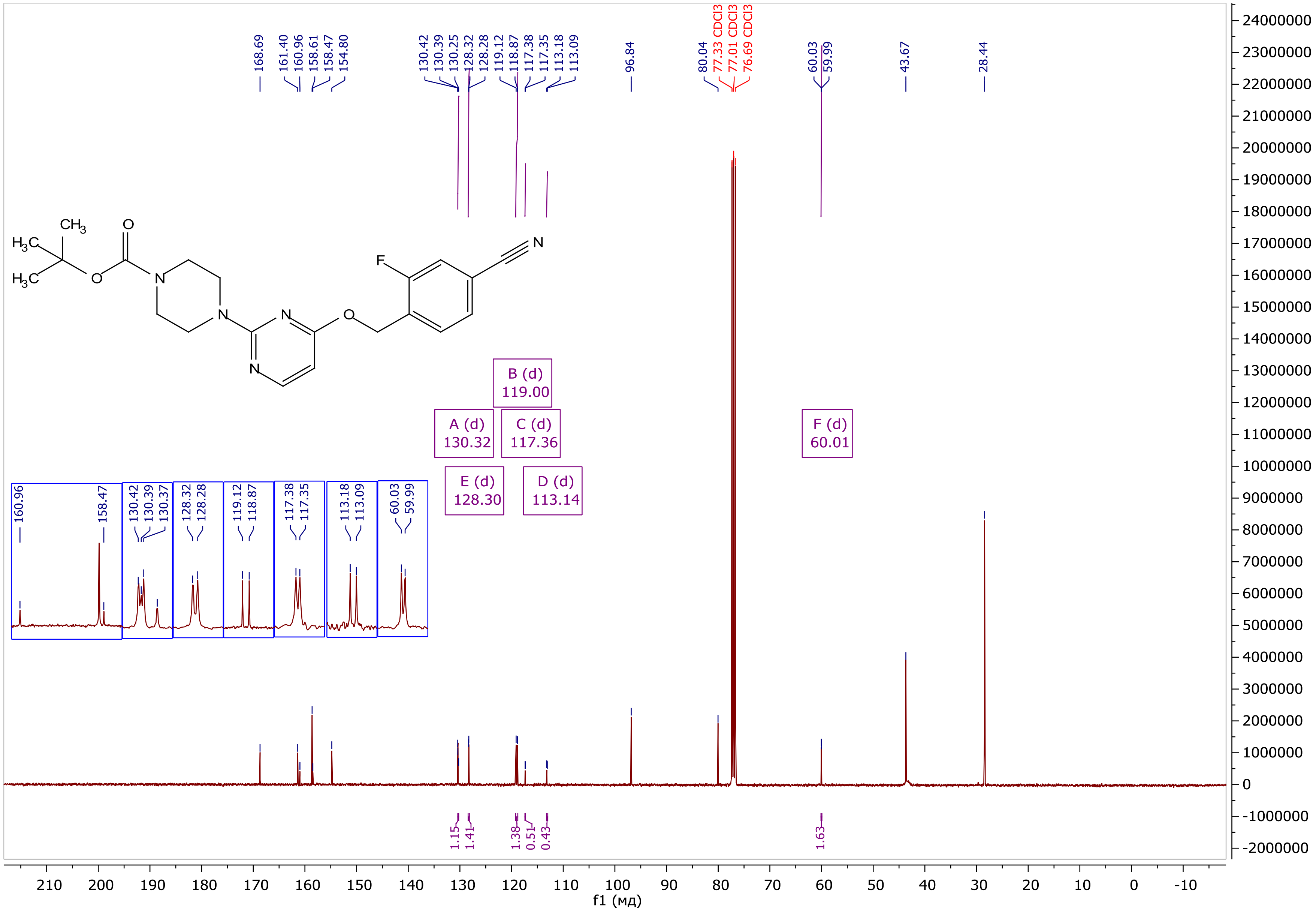

^{1}H NMR spectrum of compound **21d**

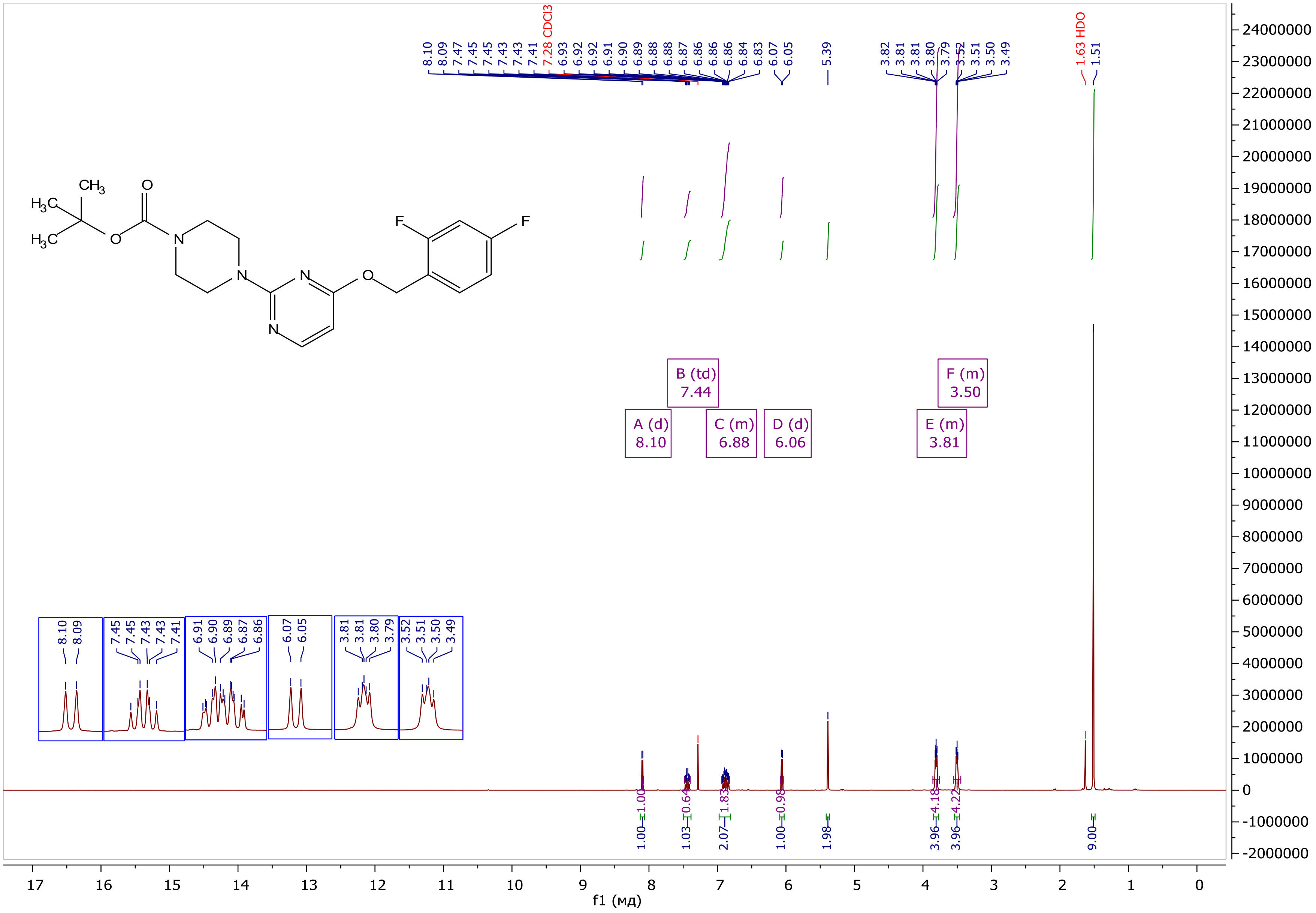

^{13}C NMR spectrum of compound **21d**

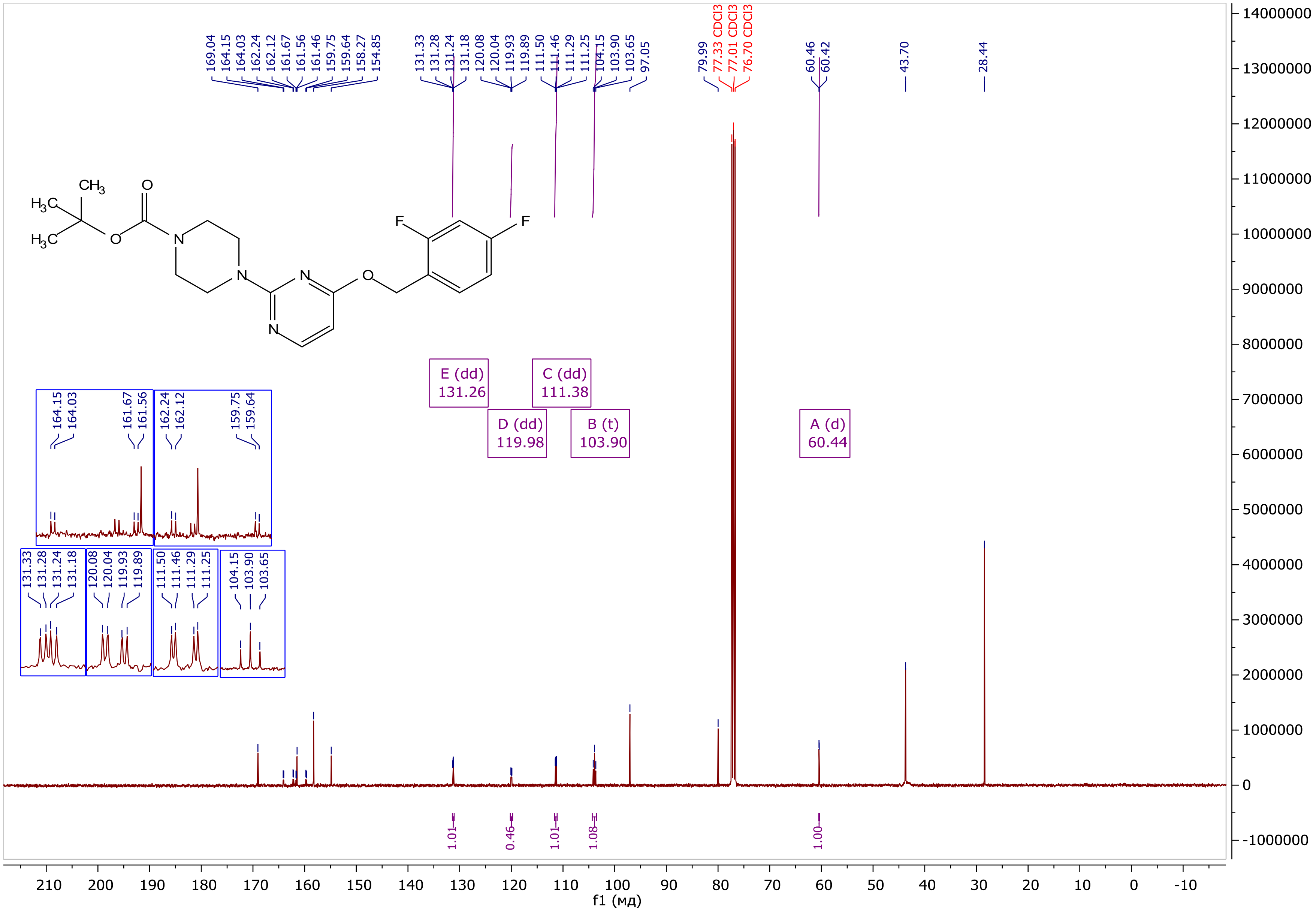

^{1}H NMR spectrum of compound **21e**

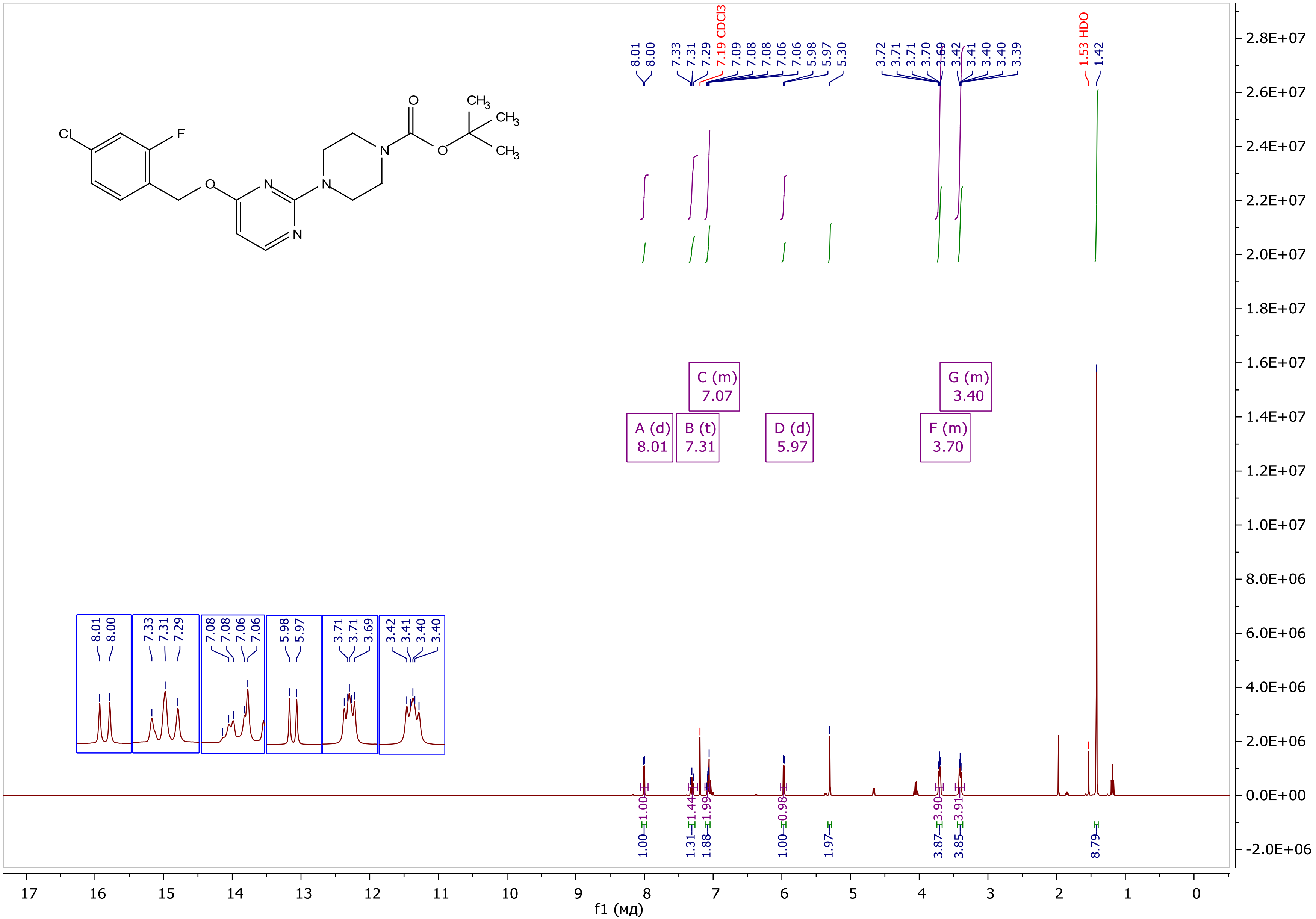

^{13}C NMR spectrum of compound **21e**

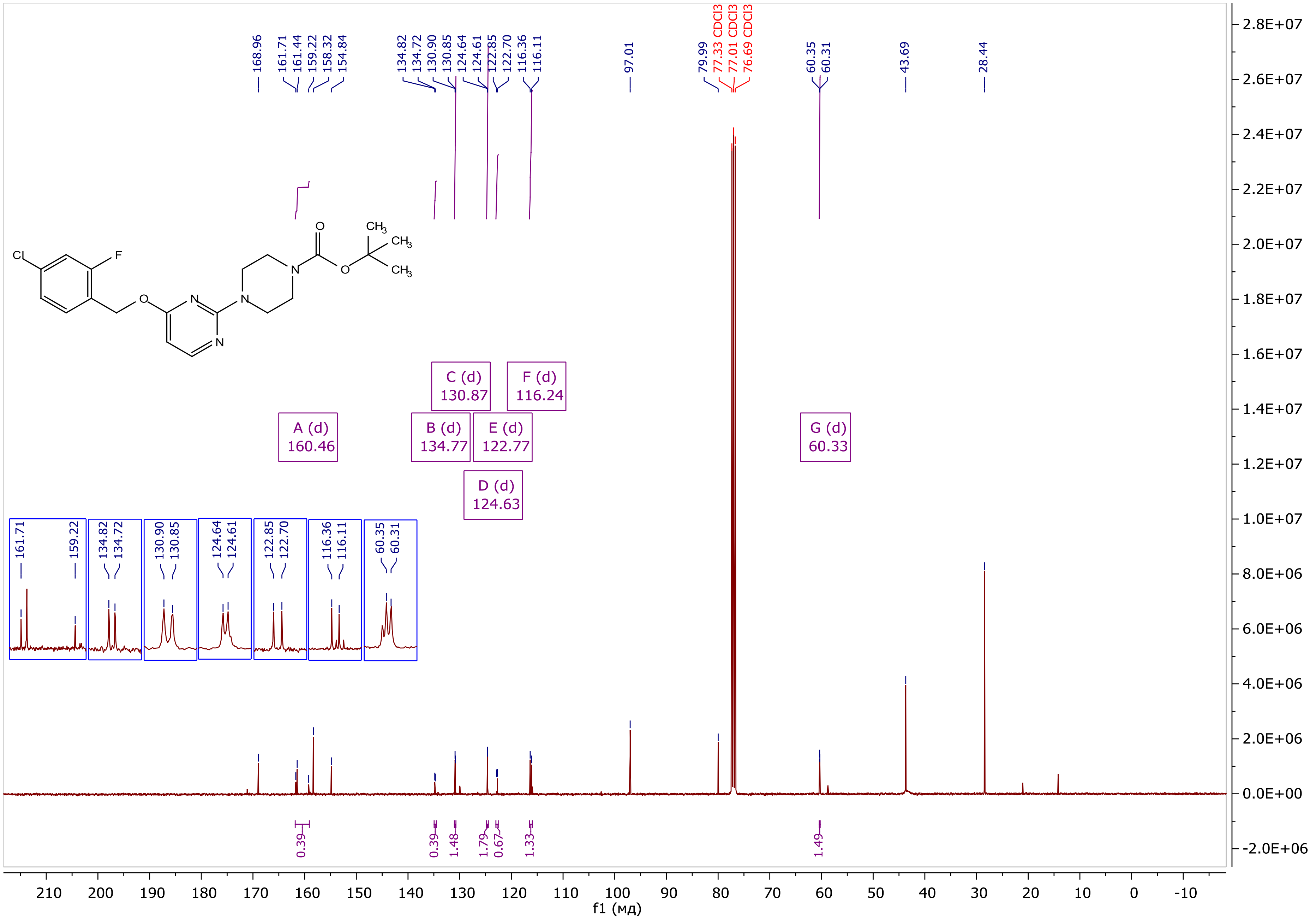

^{1}H NMR spectrum of compound **21c'**

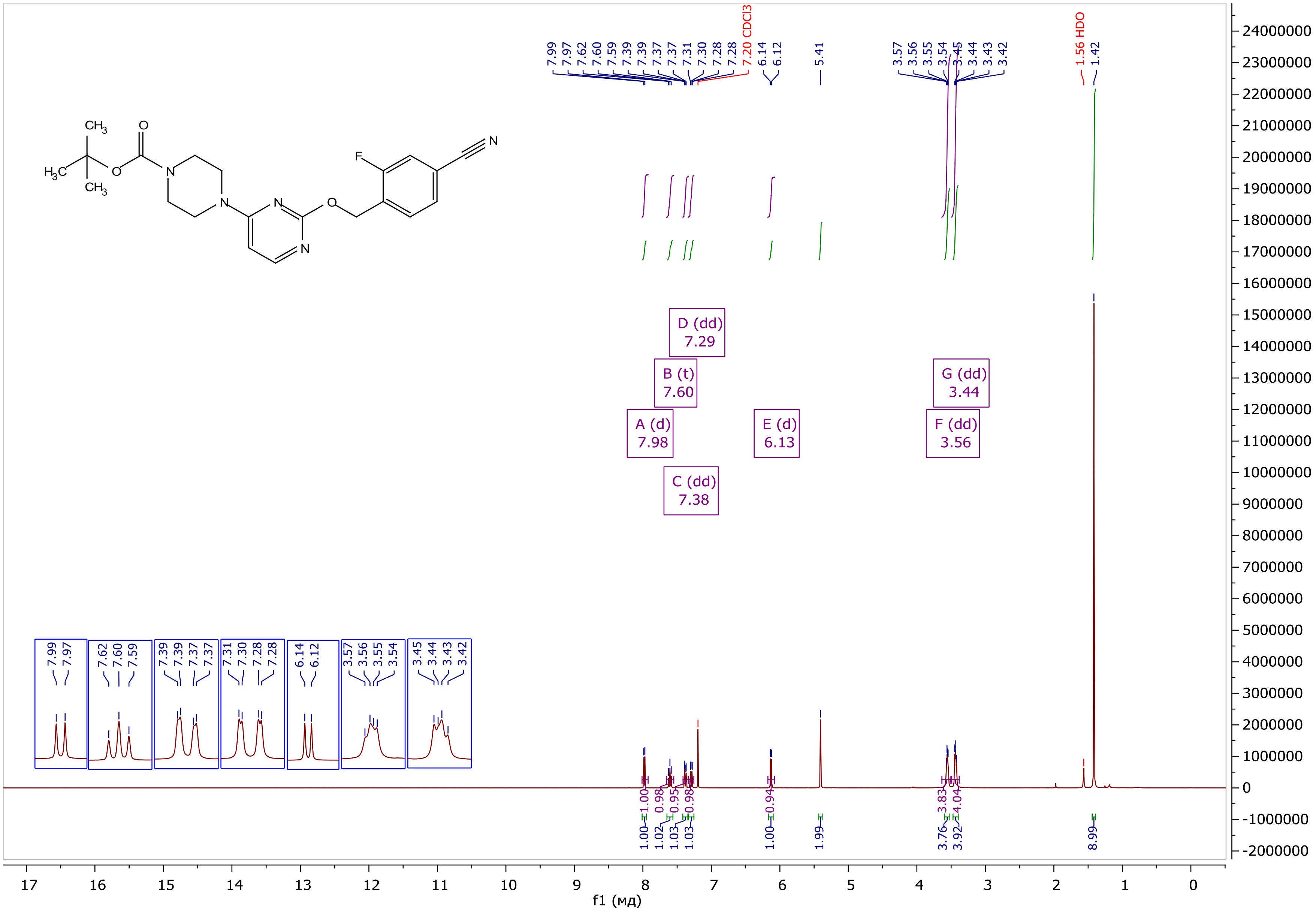

^{13}C NMR spectrum of compound **21c'**

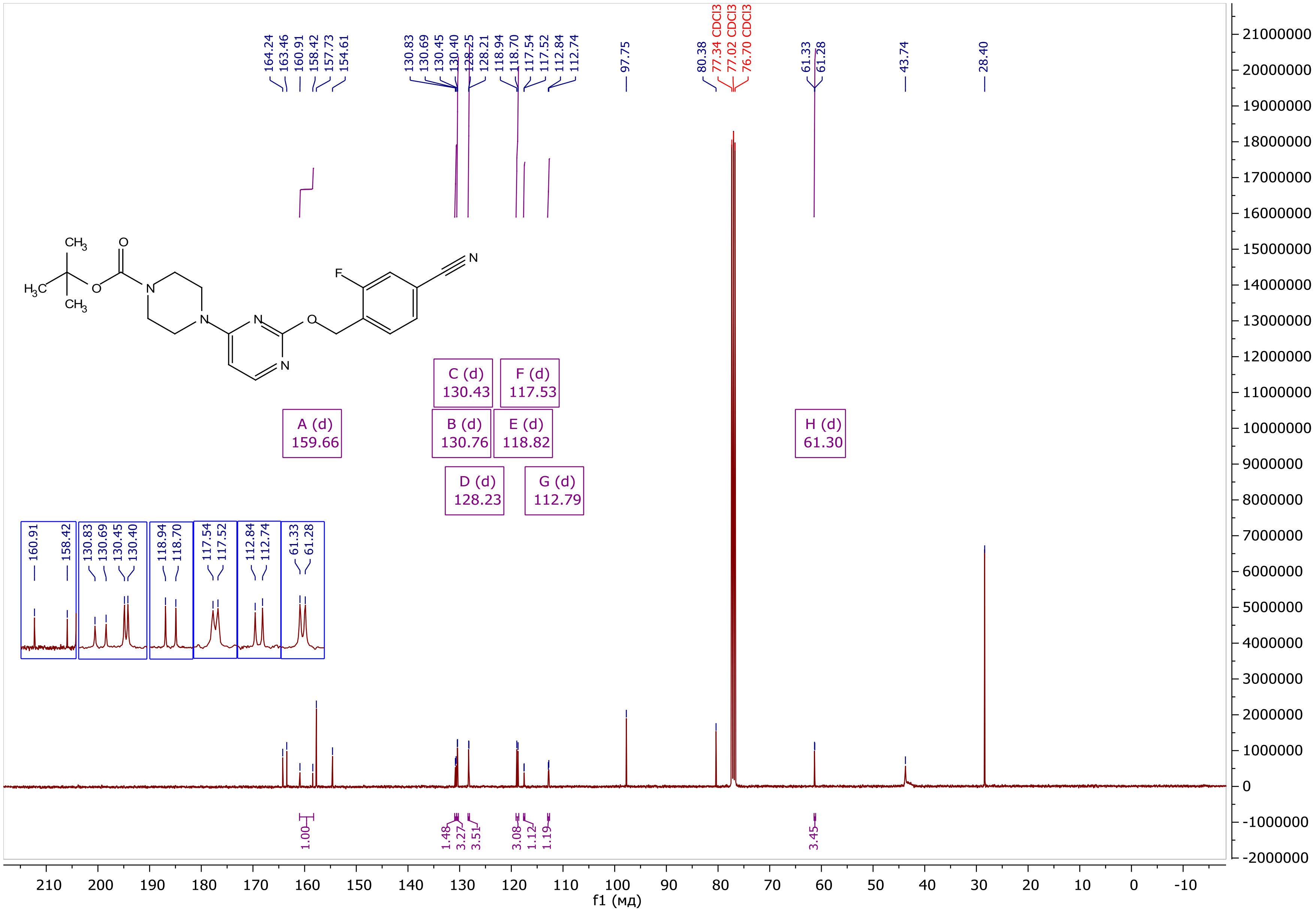

^{1}H NMR spectrum of compound **21e'**

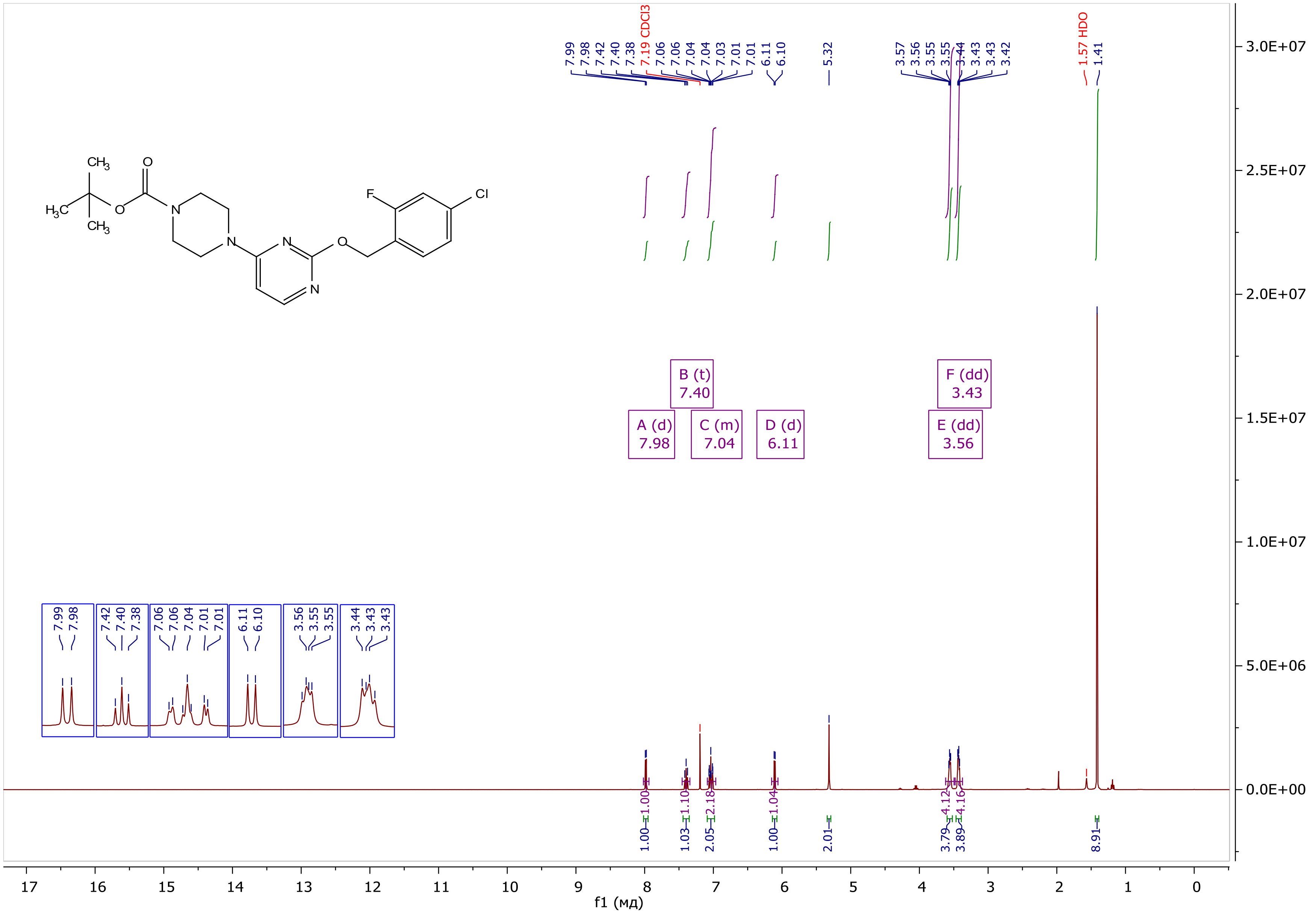

^{13}C NMR spectrum of compound **21e'**

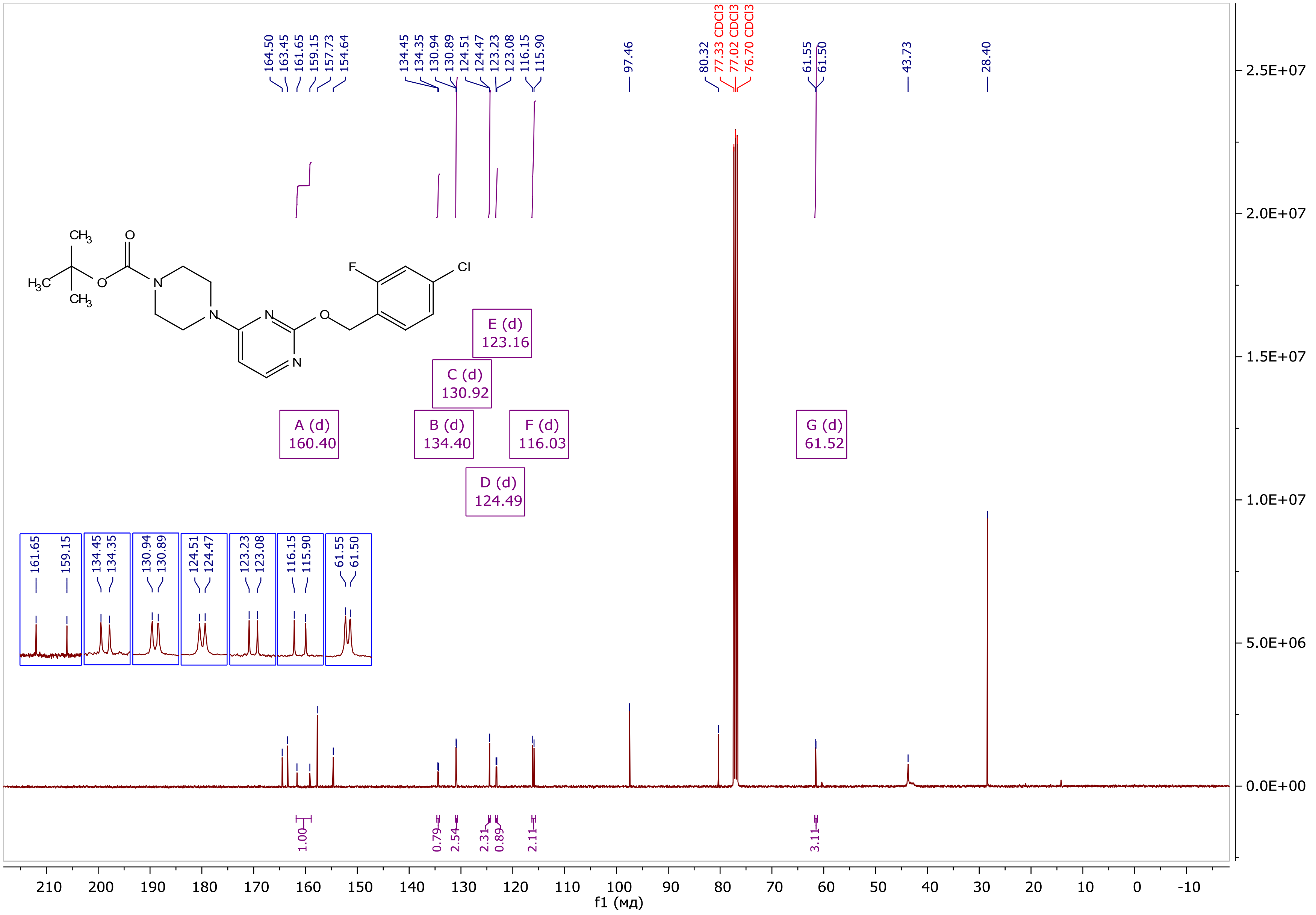

^{1}H NMR spectrum of compound **22a**

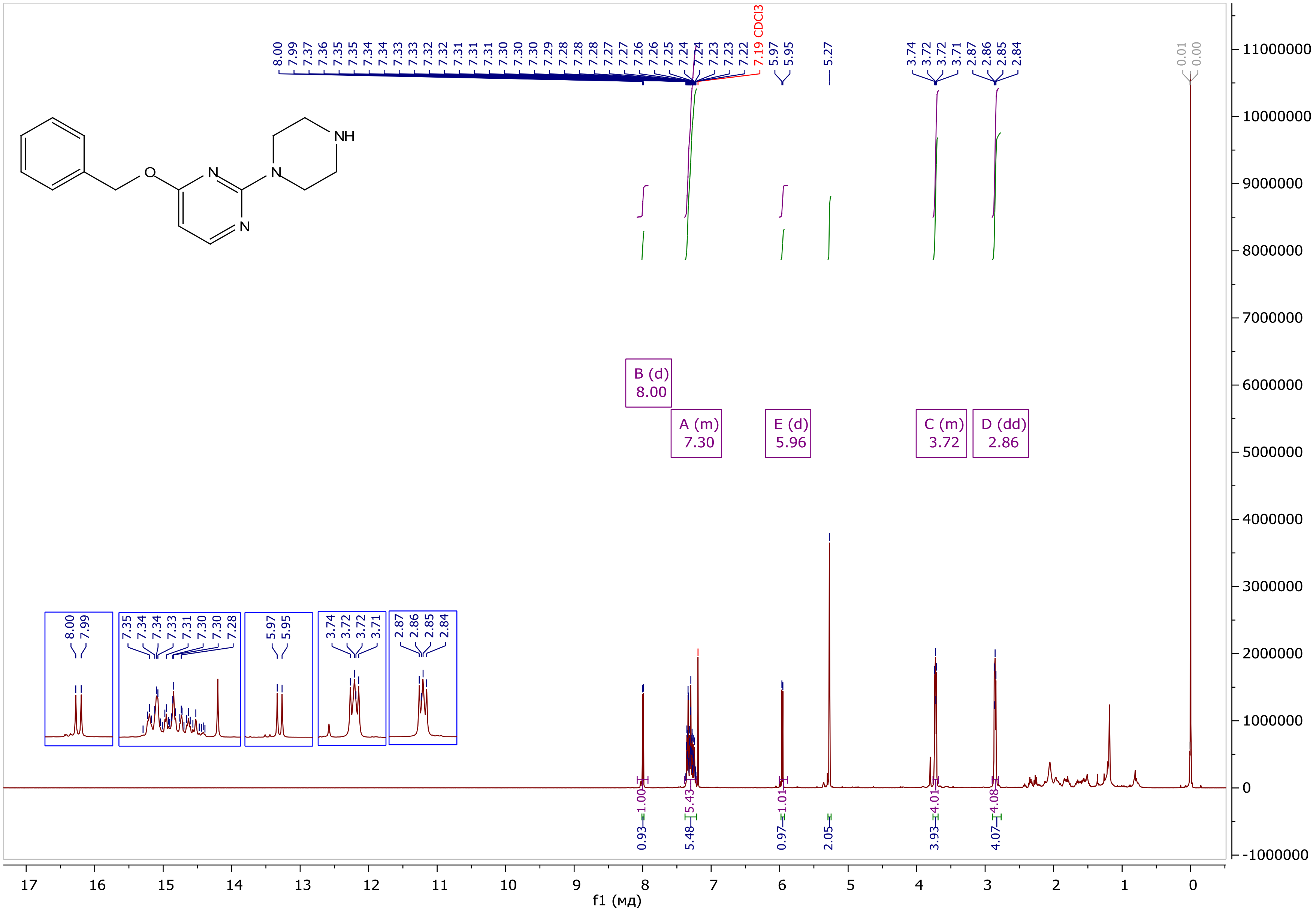

^{13}C NMR spectrum of compound **22a**

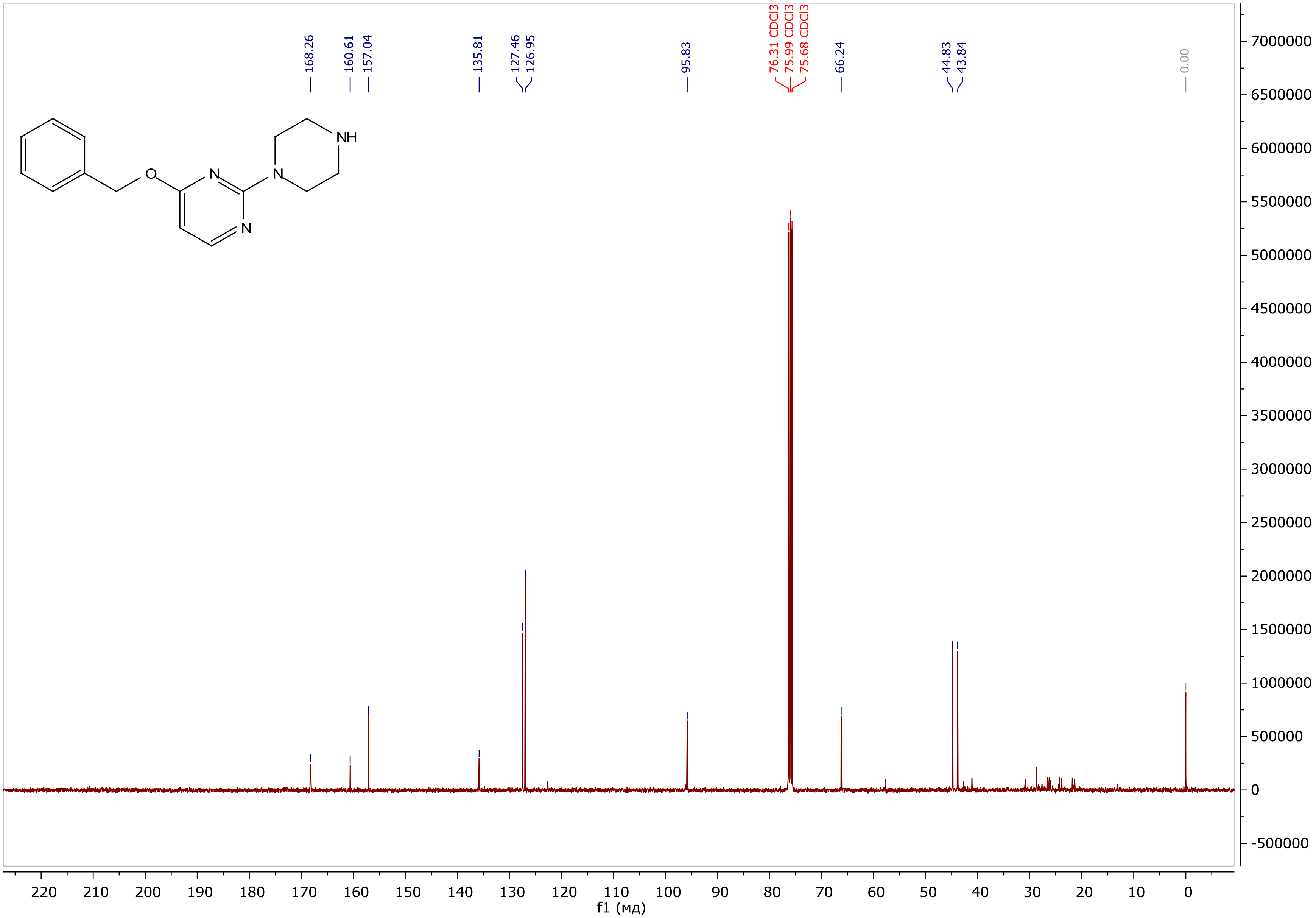

^{1}H NMR spectrum of compound **22b**

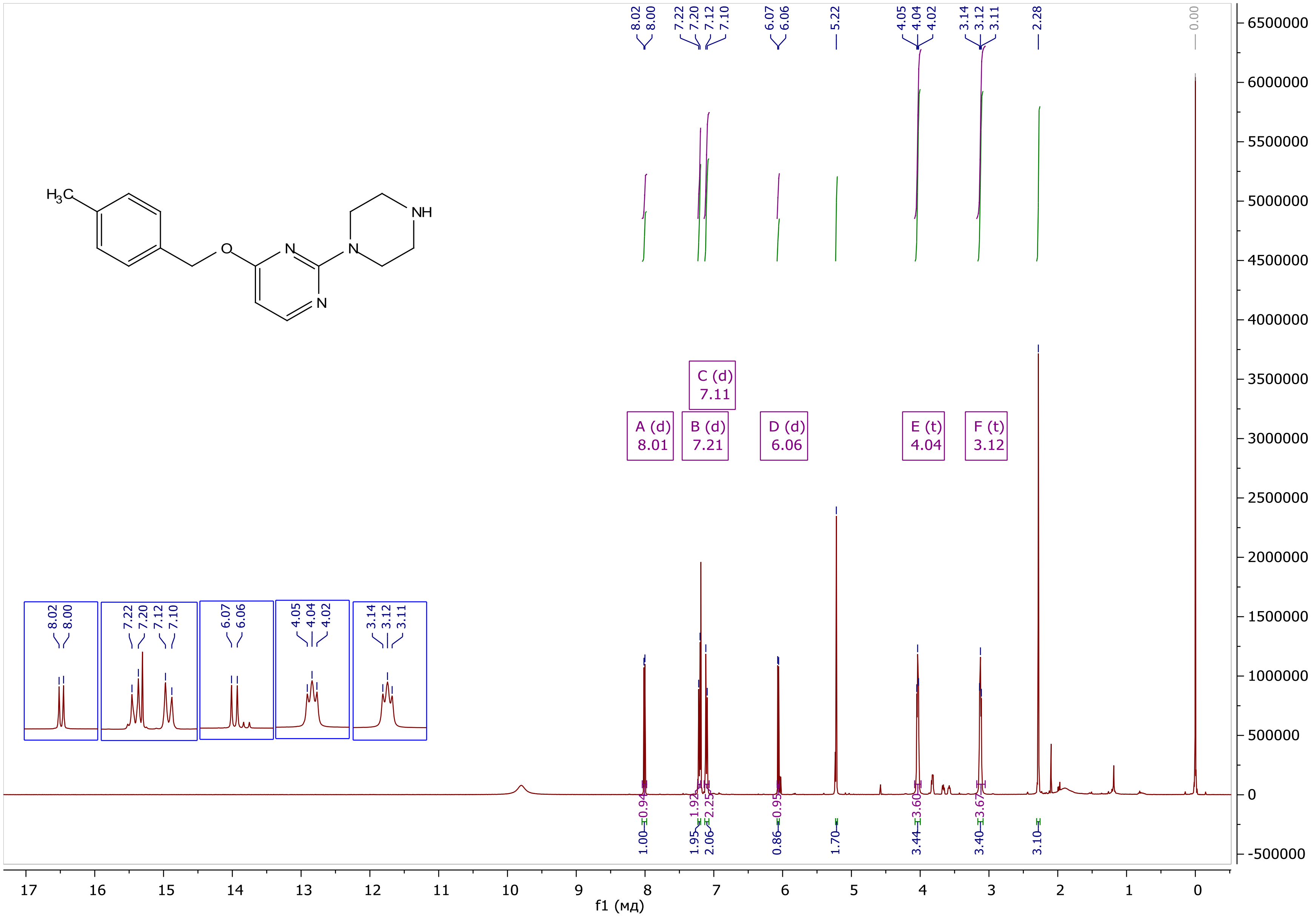

^{13}C NMR spectrum of compound **22b**

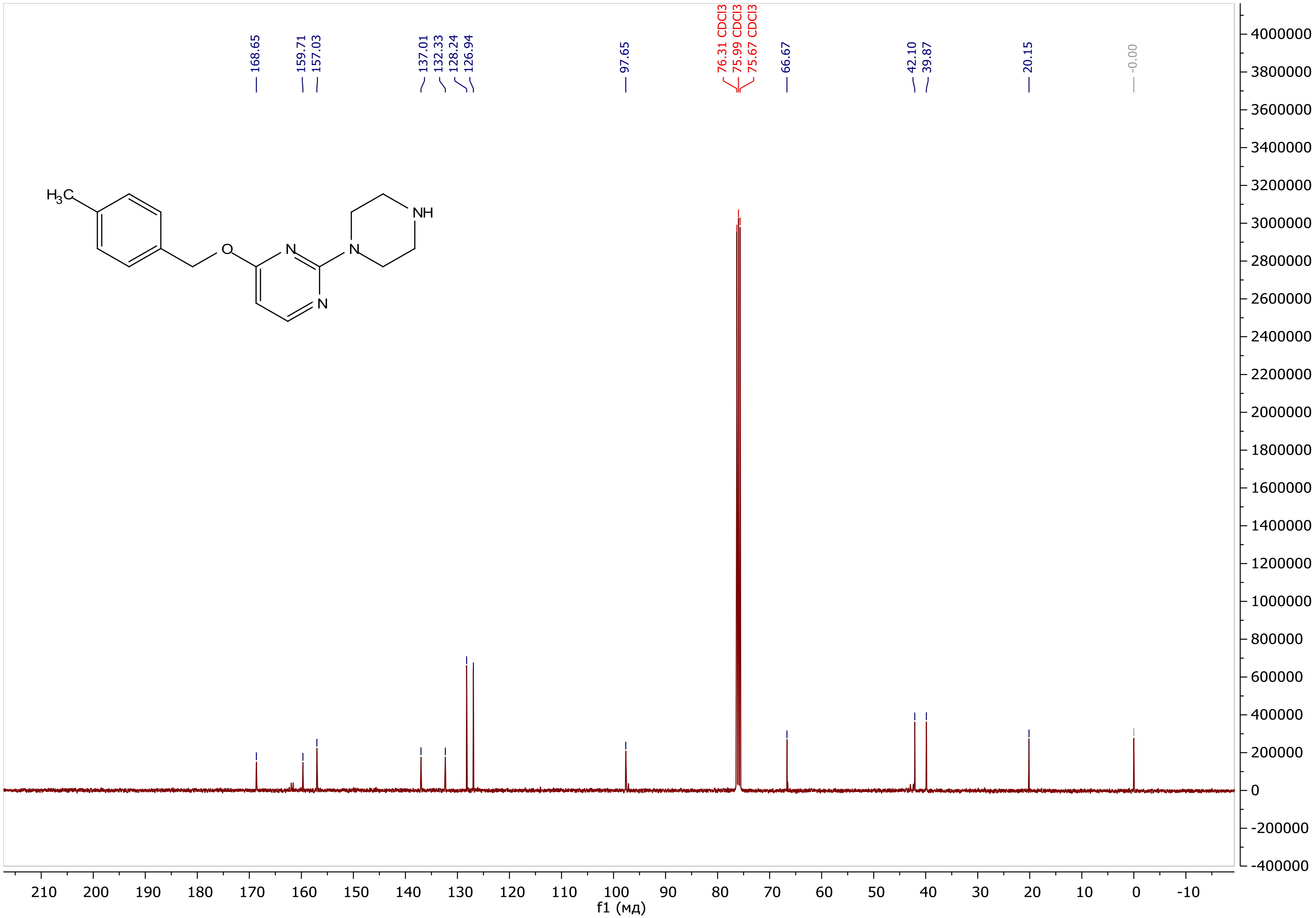

^{1}H NMR spectrum of compound **22c**

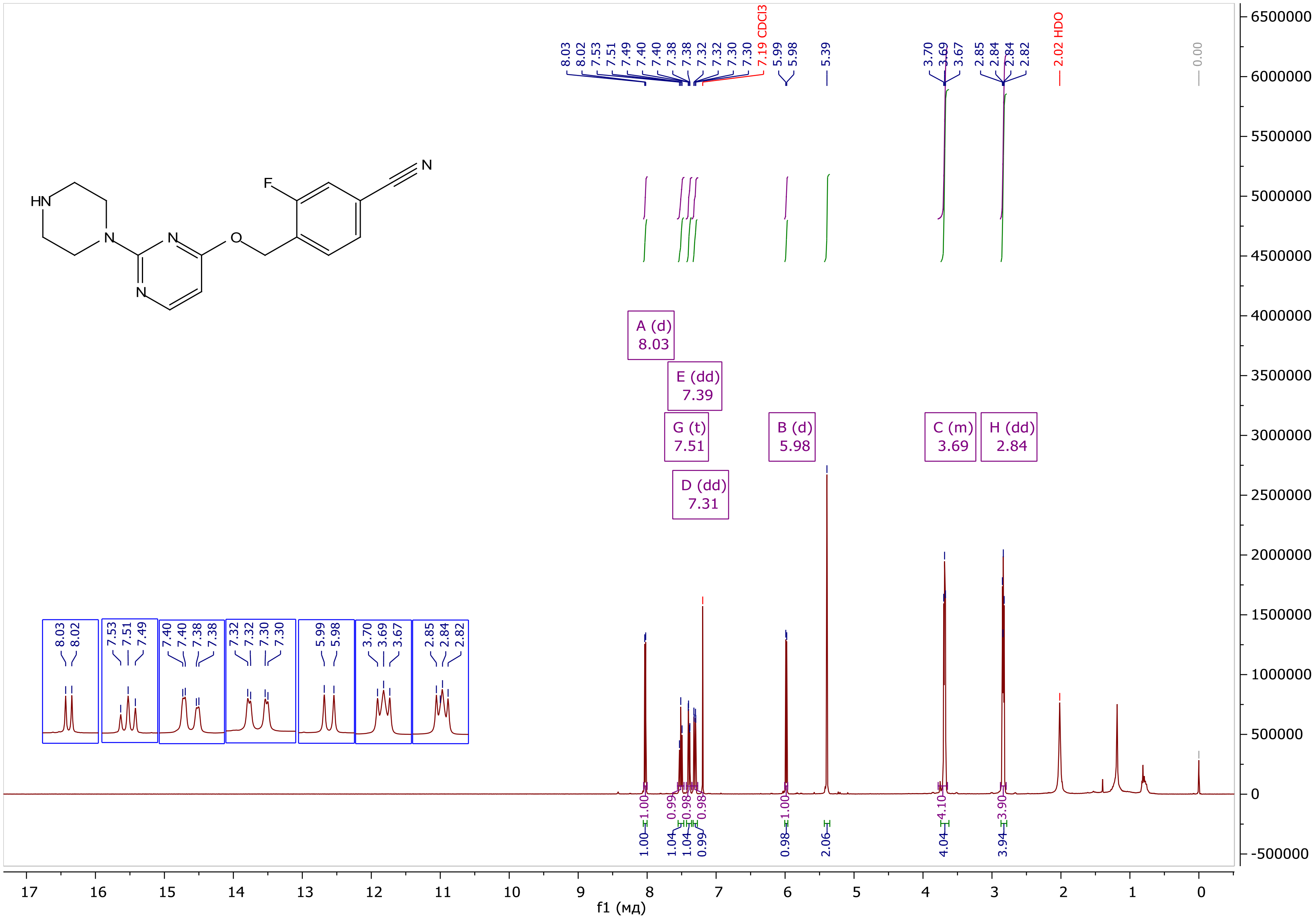

^{13}C NMR spectrum of compound **22c**

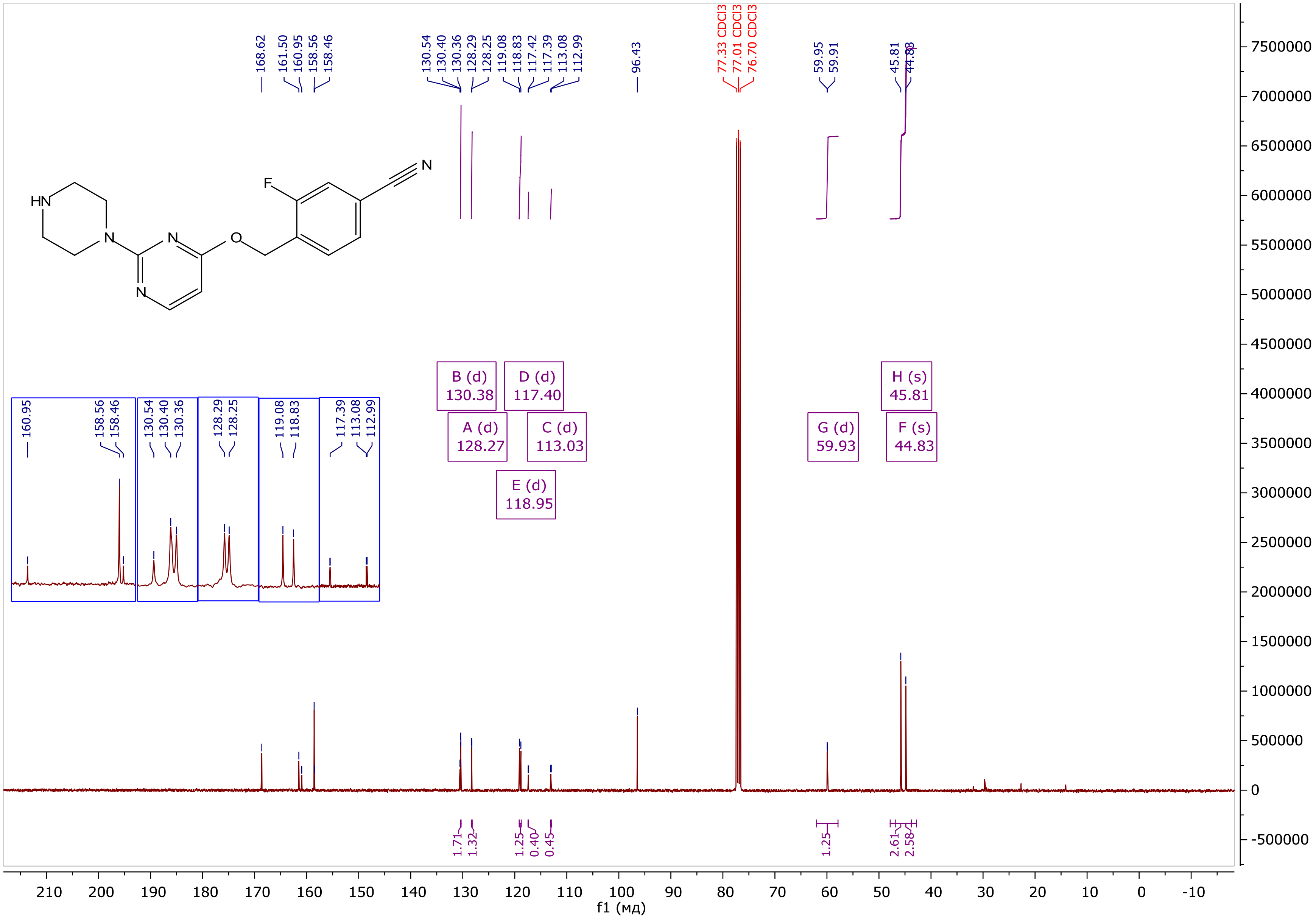

^{1}H NMR spectrum of compound **22d**

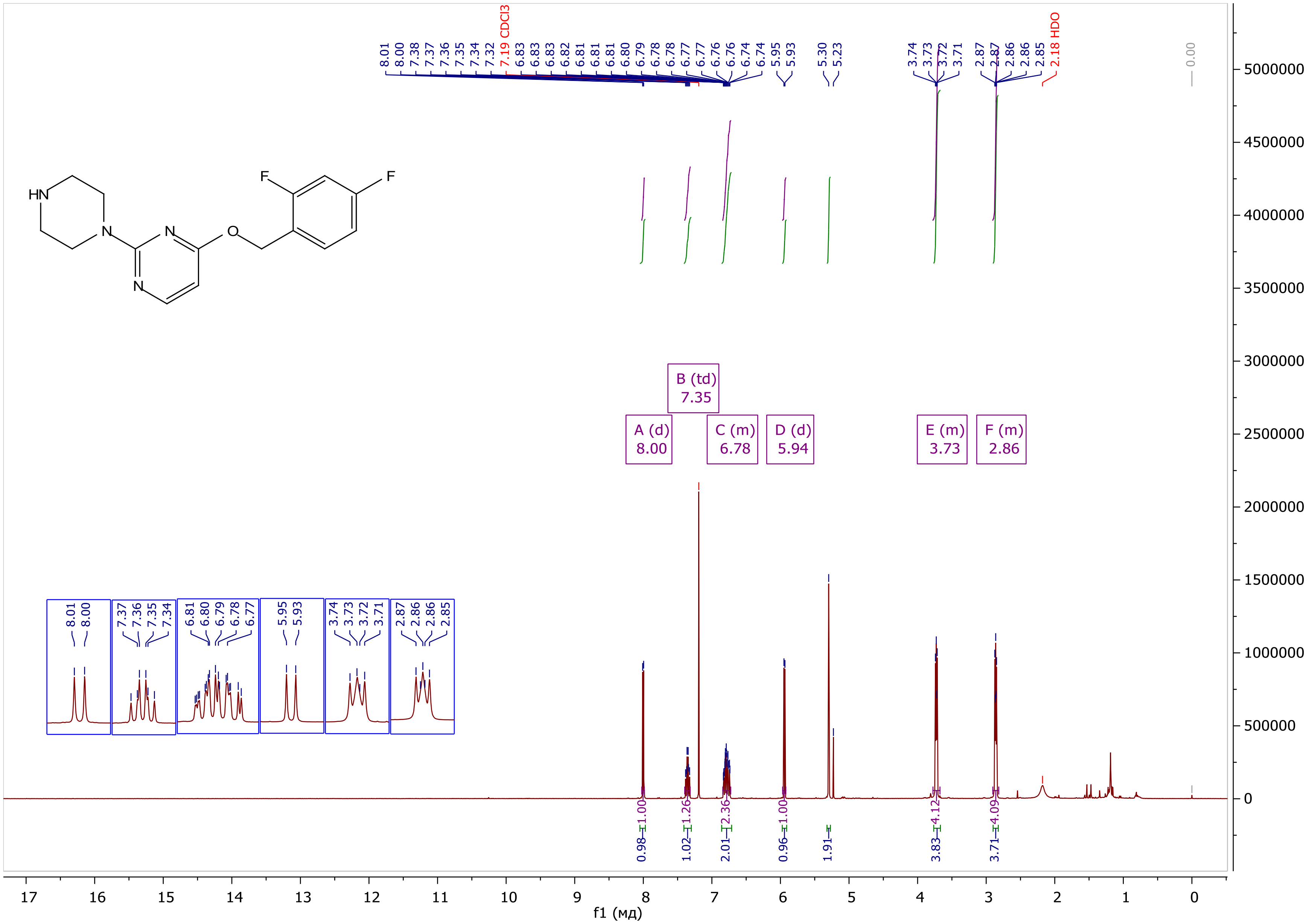

^{13}C NMR spectrum of compound **22d**

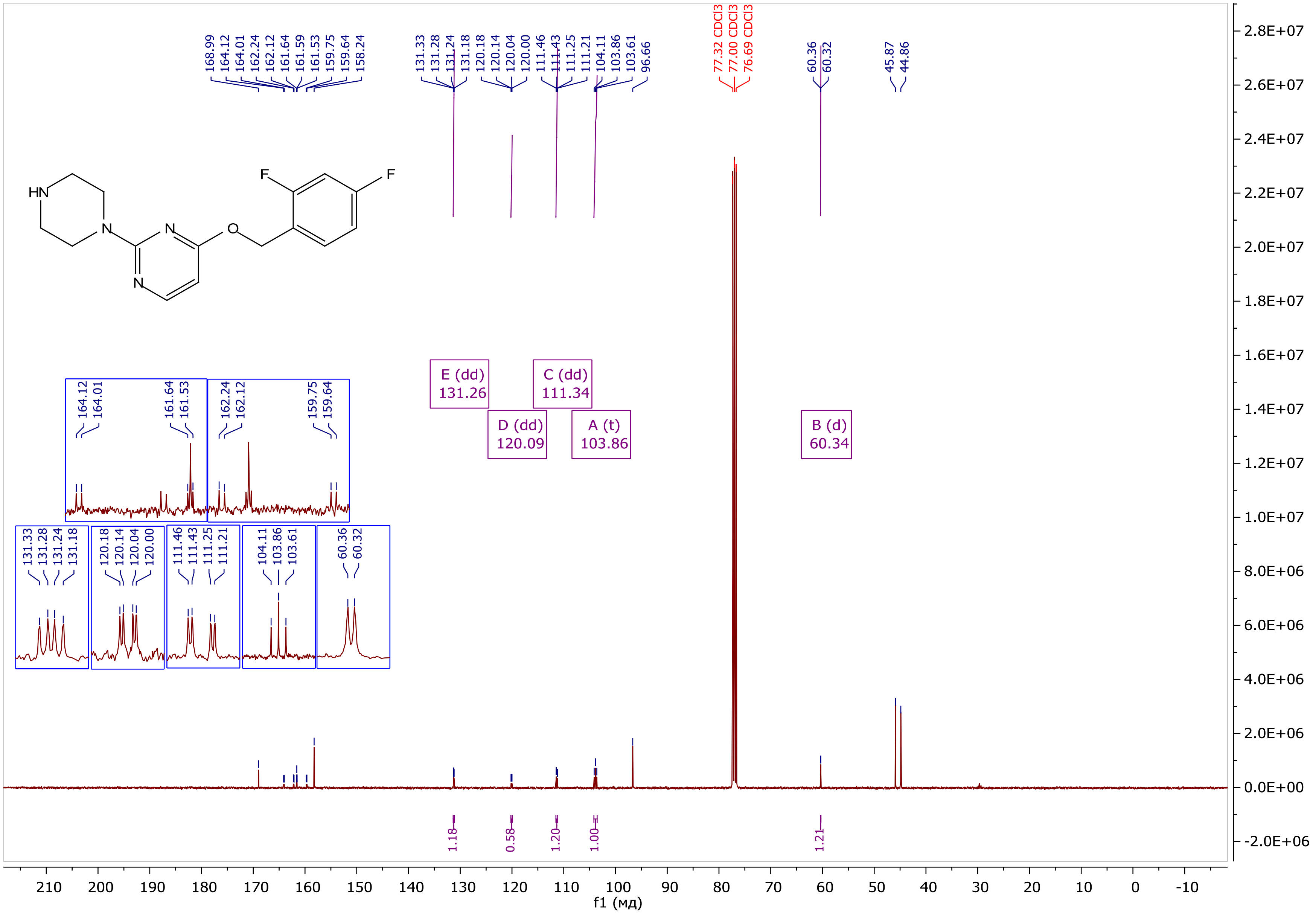

^{1}H NMR spectrum of compound **22e**

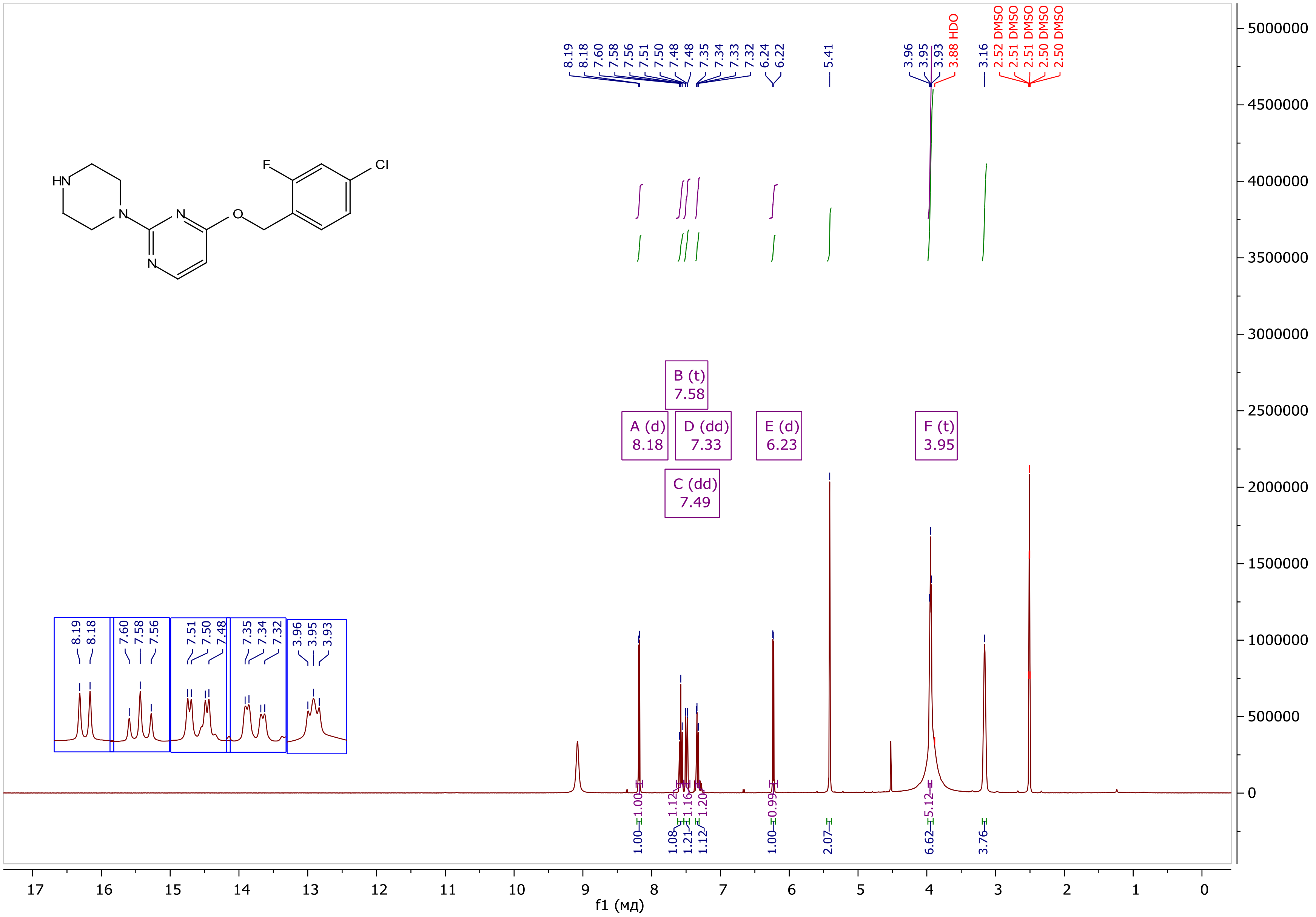

^{13}C NMR spectrum of compound **22e**

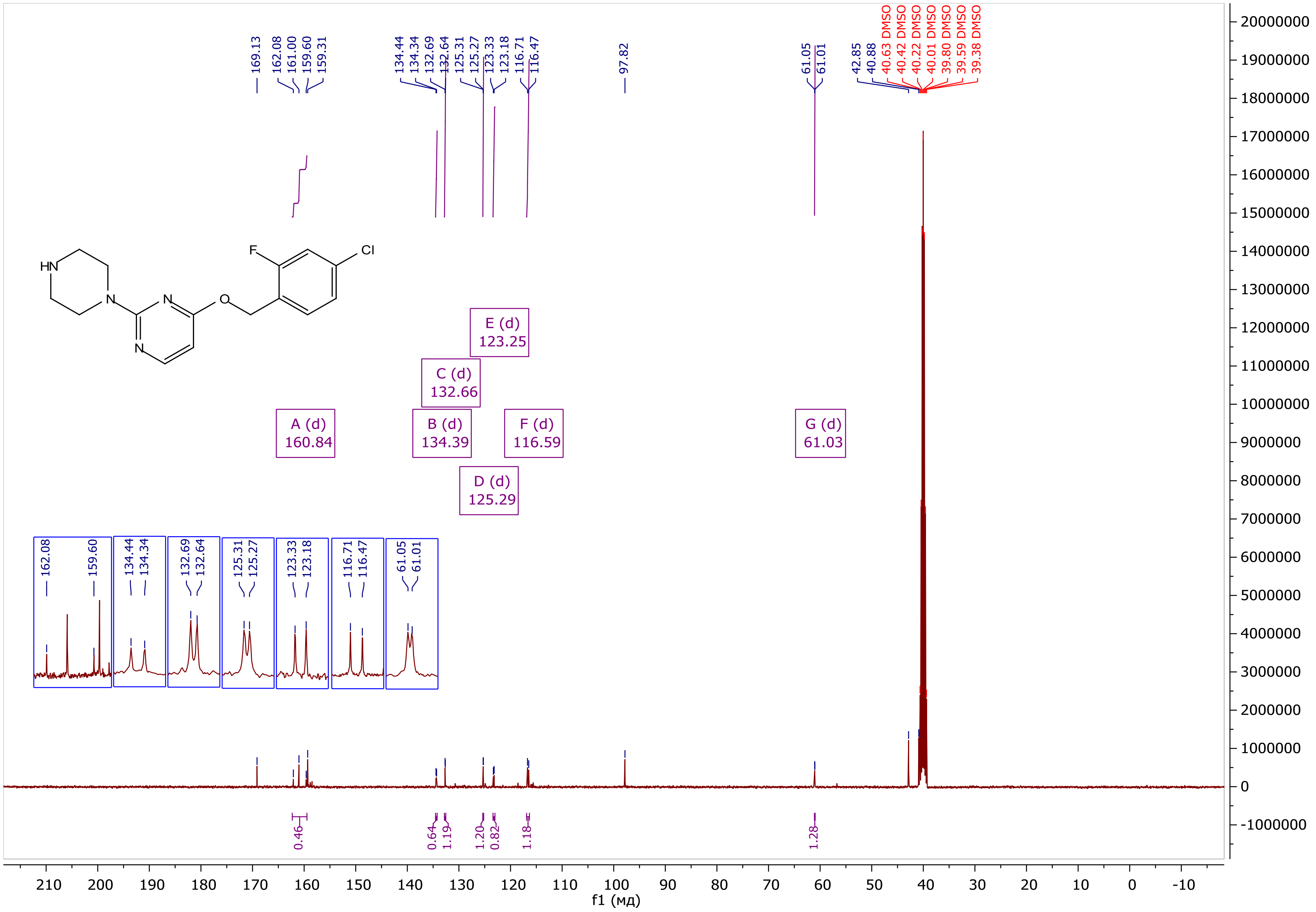

^{1}H NMR spectrum of compound **22c'**

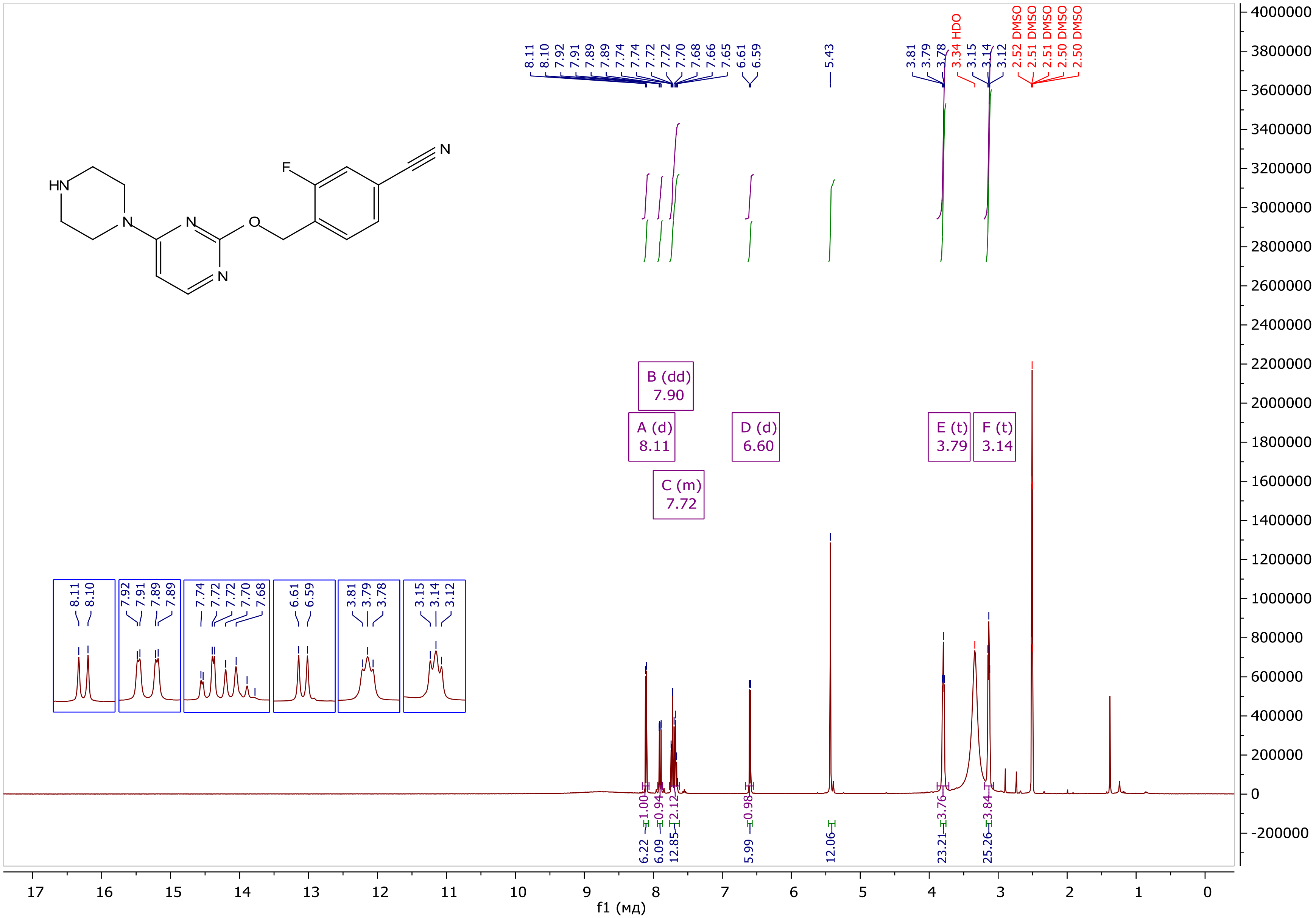

^{13}C NMR spectrum of compound **22c'**

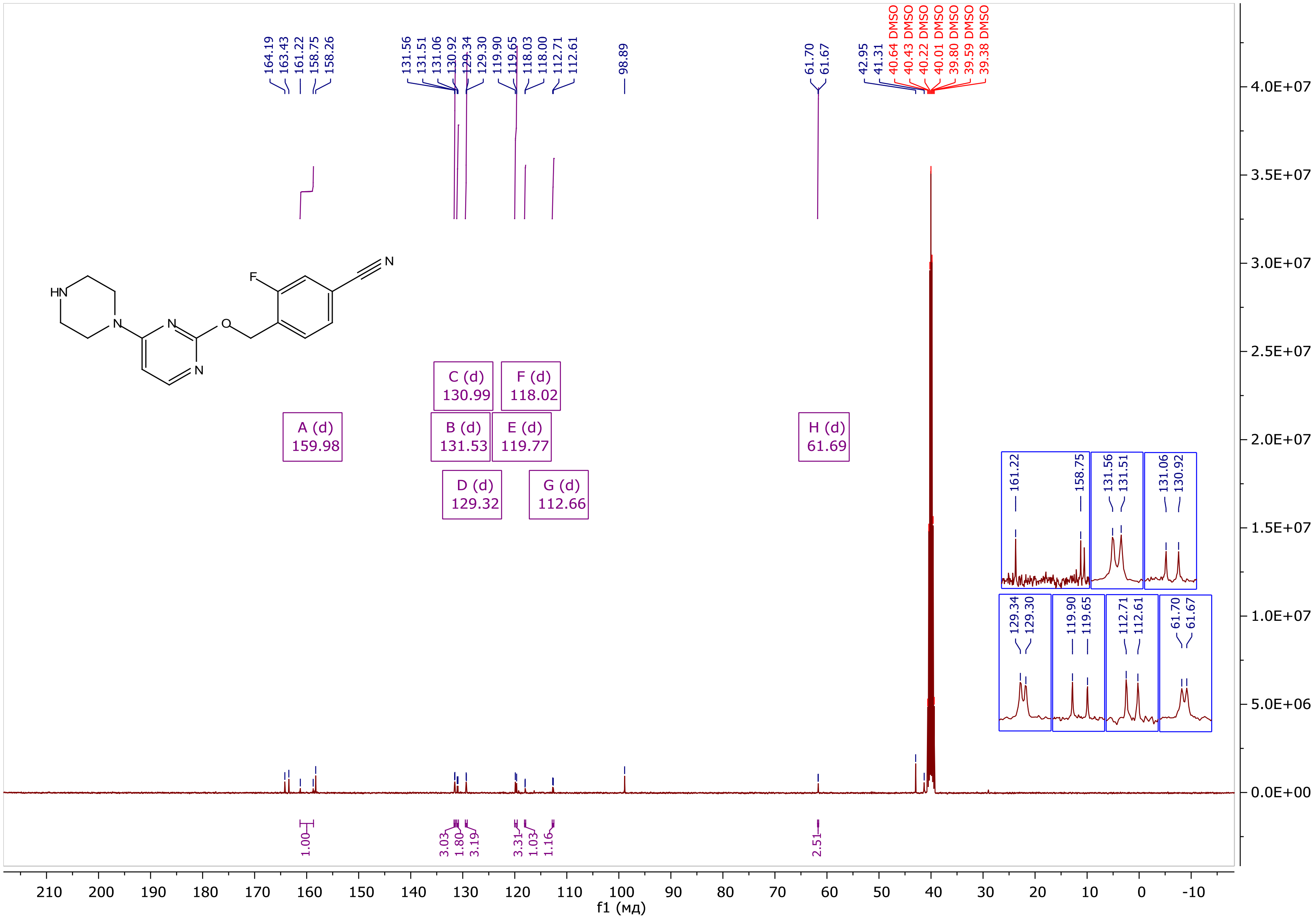

^{1}H NMR spectrum of compound **22e'**

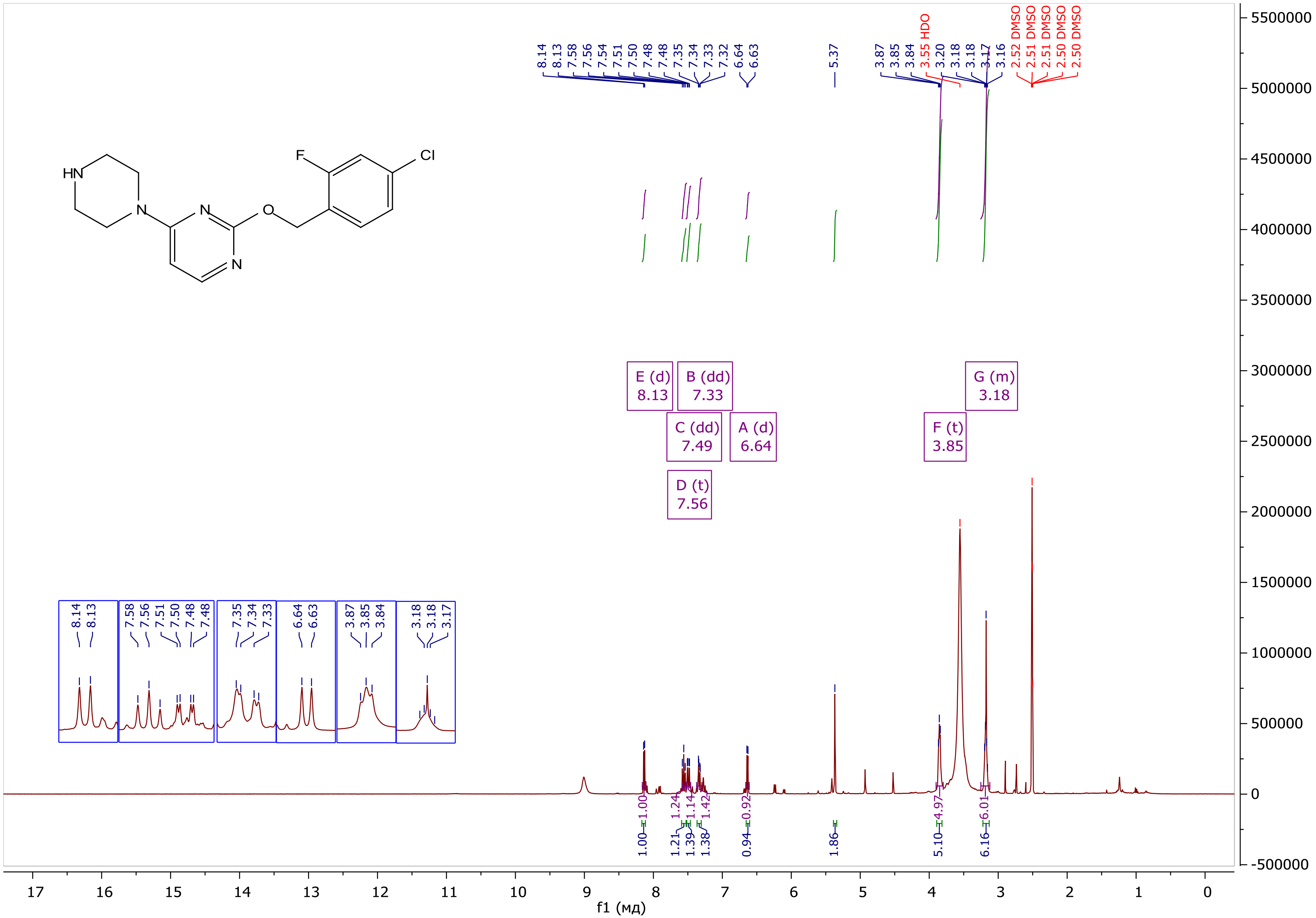

^{13}C NMR spectrum of compound **22e'**

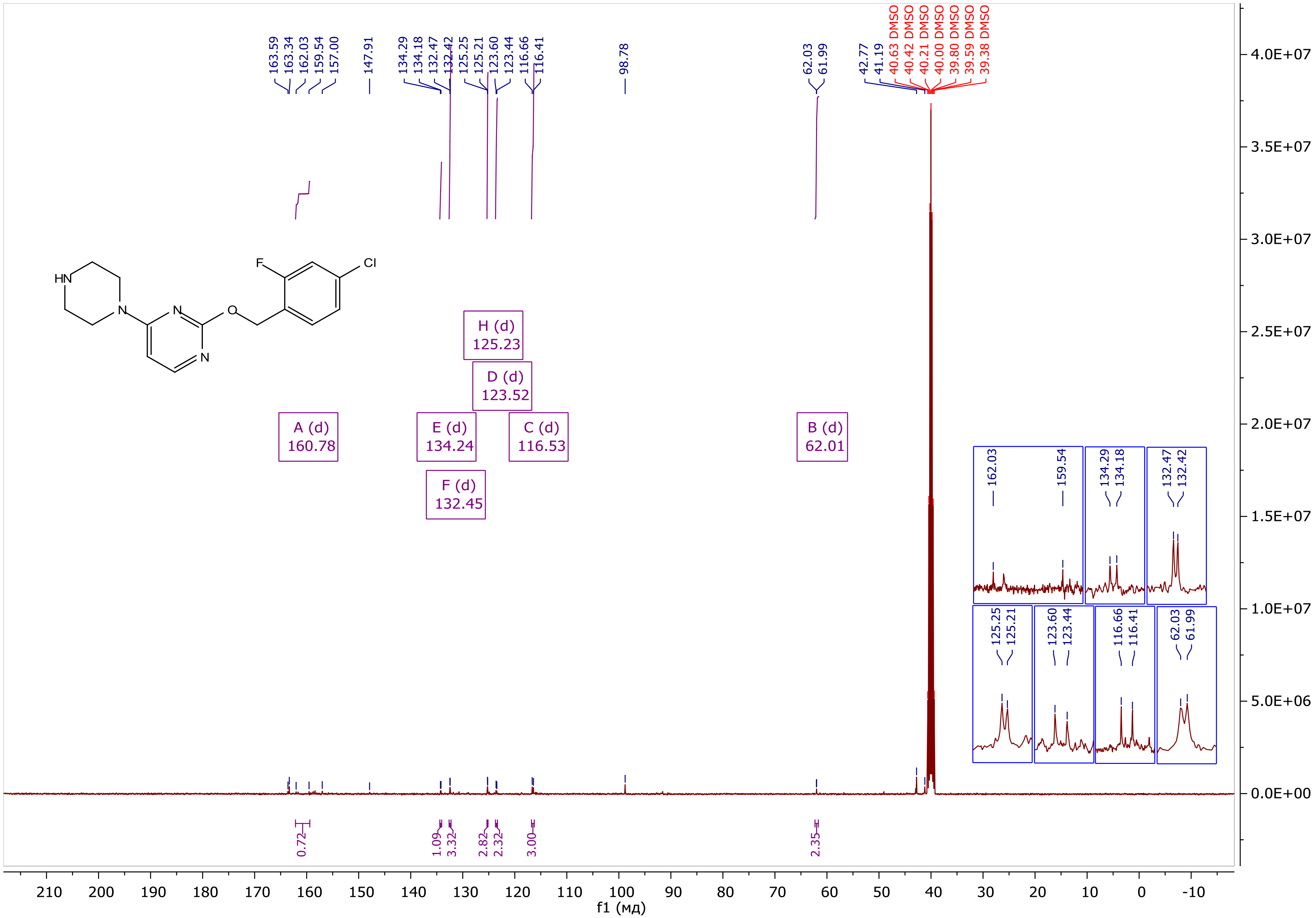

Target methyl esters 23a-23aa, 23r', 23s', 23z', 23aa'

General procedure to synthesis of methyl esters **23a-23aa**, **23r'**, **23s'**, **23z'**, **23aa'**. A corresponding 1*H*-benzo[*d*]imidazole derivative **18a-l** (1 eq) was dissolved in AcN (10 ml) at rt under vigorous stirring in a round bottom flask. A weighted portion of KI (1 eq) was added to the resulting solution and the mixture was stirred for 15 min until the salt was completely dissolved. Then K_2CO_3 (3 eq) and corresponding pyrimidine derivative **22a-e**, **22c'**, **22e'** (1 eq) were sequentially added to the resulting solution. The mixture was stirred at rt for 12 h, the reaction was monitored by TLC (DCM:MeOH = 95:5). After the reaction was completed, the precipitate was filtered off and washed with AcN (10 ml), the filtrate was evaporated to dryness. The resulting residue was dissolved in EtOAc and washed sequentially with deionized water (2×20 ml) brine solution (1×15 ml). The combined organic layer was dried with Na_2SO_4 under vigorous stirring for 1 h, after which the precipitate was filtered off, the solvent was evaporated to dryness. The resulting residue was purified by column chromatography on silica gel (DCM:MeOH = 95:5). Fractions containing the target product was collected, the solvent was evaporated to dryness to form the corresponding methyl ester **23a-23aa**, **23r'**, **23s'**, **23z'**, **23aa'**.

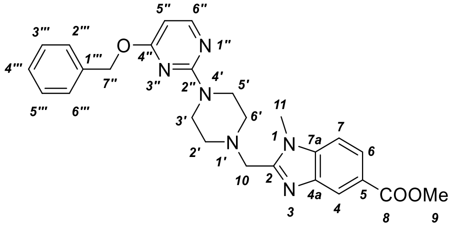

Methyl 2-((4-(4-(benzyloxy)pyrimidin-2-yl)piperazin-1-yl)methyl)-1-methyl-1H-benzo[d]imidazole-5-carboxylate **23a**, white solid, 75 mg, 90%. **^{1}H NMR (400 MHz, CDCl$_3$, *δ ppm*)**: 8.38 (1H, *s*, 4-CH), 8.02-7.96 (2H, *m*, 6"-CH, 6-CH), 7.33-7.21 (6H, *m*, 7-CH, 2'''-CH, 3'''-CH, 4'''-CH, 5'''-CH, 6'''-CH), 5.96 (1H, *d*, *J* = 5.6 Hz, 5"-CH), 5.25 (2H, *s*, 7"-CH$_2$), 3.88 (6H, *s*, 9-CH$_3$, 11-CH$_3$), 3.80 (2H, *s*, 10-CH$_2$), 3.74-3.72 (4H, *m*, 3'-CH$_2$, 5'-CH$_2$), 2.53-2.50 (4H, *m*, 2'-CH$_2$, 6'-CH$_2$). **^{13}C NMR (101 MHz, CDCl$_3$, *δ ppm*)**: 169.3 (C-4"), 167.6 (C-8), 161.5 (C-2"), 157.8 (C-6"), 152.9 (C-2), 141.8 (C-4a), 139.6 (C-7a), 136.7 (C-1'''), 127.9 (C-4'''), 127.8 (C-2''', C-6'''), 128.4 (C-3''', C-5'''), 124.3 (C-6), 124.2 (C-5), 122.0 (C-4), 108.9 (C-7), 97.1 (C-5"), 67.3 (C-7"), 55.7 (C-10), 53.1 (C-2', C-6'), 52.1 (C-9), 43.8 (C-3', C-5'), 30.5 (C-11). **HRMS (ESI+)**: found *m/z* 473.2471 [M + H]$^+$; calculated $C_{26}H_{29}N_6O_3^+$ 473.2223.

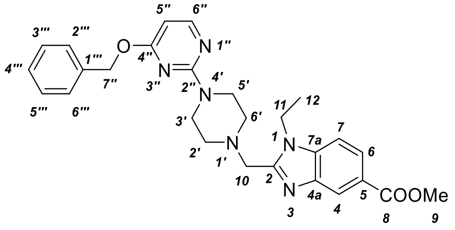

Methyl 2-((4-(4-(benzyloxy)pyrimidin-2-yl)piperazin-1-yl)methyl)-1-ethyl-1H-benzo[d]imidazole-5-carboxylate **23b**, light yellow solid, 90 mg, 95%. **^{1}H NMR (400 MHz, CDCl$_3$, *δ ppm*)**: 8.39-8.38 (1H, *m*, 4-CH), 7.99 (1H, *d*, *J* = 5.6 Hz, 6"-CH), 7.95 (1H, *dd*, *J* = 8.5, 1.6 Hz, 6-CH), 7.33-7.21 (6H, *m*, 7-CH, 2'''-CH, 3'''-CH, 4'''-CH, 5'''-CH, 6'''-CH), 5.97 (1H, *d*, *J* = 5.6 Hz, 5"-CH), 5.25 (2H, *s*, 7"-CH$_2$), 4.33 (2H, *q*, *J* = 7.2 Hz, 11-CH$_2$), 3.87 (3H, *s*, 9-CH$_3$), 3.79 (2H, *s*, 10-CH$_2$), 3.74-3.71 (4H, *m*, 3'-CH$_2$, 5'-CH$_2$), 2.53-2.51 (4H, *m*, 2'-CH$_2$, 6'-CH$_2$), 1.43 (3H, *t*, *J* = 7.2 Hz, 12-CH$_3$). **^{13}C NMR (101 MHz, CDCl$_3$, *δ ppm*)**: 169.3 (C-4"), 167.6 (C-8), 161.5 (C-2"), 158.1 (C-6"), 152.4 (C-2), 142.0 (C-4a), 138.5 (C-7a), 136.8 (C-1'''), 128.5 (C-3''', C-5'''), 128.0 (C-4'''), 127.9 (C-2''', C-6'''), 124.3 (C-6), 124.3 (C-5), 122.2 (C-4), 109.1 (C-7), 97.1 (C-5"), 67.3 (C-7"), 55.8 (C-10), 53.1 (C-2', C-6'), 52.1 (C-9), 43.8 (C-3', C-5'), 39.3 (C-11), 15.1 (C-12). **HRMS (ESI+)**: found *m/z* 487.2626 [M + H]$^+$; calculated $C_{27}H_{31}N_6O_3^+$ 487.2379.

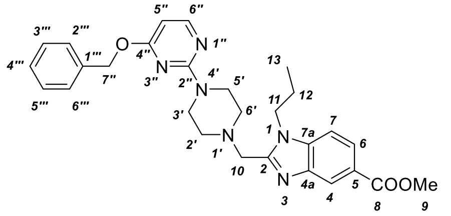

Methyl 2-((4-(4-(benzyloxy)pyrimidin-2-yl)piperazin-1-yl)methyl)-1-propyl-1H-benzo[d]imidazole-5-carboxylate **23c**, white amorphous solid, 110 mg, 95%. **^{1}H NMR (400 MHz, CDCl$_3$, *δ ppm*)**: 8.38 (1H, *d*, *J* = 1.5 Hz, 4-CH), 7.99 (1H, *d*, *J* = 5.6 Hz, 6"-CH), 7.94 (1H, *dd*, *J* = 8.5, 1.6 Hz, 6-CH), 7.39-7.21 (6H, *m*, 7-CH, 2'''-CH, 3'''-CH, 4'''-CH, 5'''-CH, 6'''-CH), 5.97 (1H, *d*, *J* = 5.6 Hz, 5"-CH), 5.25 (2H, *s*, 7"-CH$_2$), 4.22 (2H, *dd*, *J* = 8.7, 6.5 Hz, 11-CH$_2$), 3.88 (3H, *s*, 9-CH$_3$), 3.80 (2H, *s*, 10-CH$_2$), 3.74-3.71 (4H, *m*, 3'-CH$_2$, 5'-CH$_2$), 2.54-2.51 (4H, *m*, 2'-CH$_2$, 6'-CH$_2$), 1.87 (2H, *h*, *J* = 7.4 Hz, 12-CH$_2$), 0.95 (3H, *t*, *J* = 7.4 Hz, 13-CH$_3$). **^{13}C NMR (101 MHz, CDCl$_3$, *δ ppm*)**: 169.3 (C-4"), 167.6 (C-8), 161.5 (C-2"), 158.1 (C-6"), 152.6 (C-2), 142.0 (C-4a), 139.0 (C-7a), 136.8 (C-1'''), 128.5 (C-3''', C-5'''), 128.0 (C-4'''), 127.9 (C-2''', C-6'''), 124.2 (C-6), 124.2 (C-5), 122.2 (C-4), 109.3 (C-7), 97.1 (C-5"), 67.3 (C-7"), 55.9 (C-10), 53.2 (C-2', C-6'), 52.1 (C-9), 46.0 (C-11), 43.8 (C-3', C-5'), 23.2 (C-12), 11.5 (C-13). **HRMS (ESI+)**: found *m/z* 501.2782 [M + H]$^+$; calculated $C_{28}H_{33}N_6O_3^+$ 501.2536.

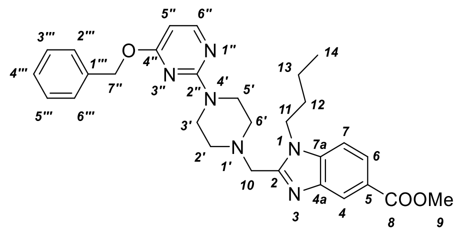

Methyl 2-((4-(4-(benzyloxy)pyrimidin-2-yl)piperazin-1-yl)methyl)-1-butyl-1H-benzo[d]imidazole-5-carboxylate **23d**, grey amorphous solid, 180 mg, 95%. **^{1}H NMR (400 MHz, $CDCl_3$, *δ ppm*)**: 8.38 (1H, *d*, *J* = 1.4 Hz, 4-CH), 7.99 (1H, *d*, *J* = 5.6 Hz, 6"-CH), 7.94 (1H, *dd*, *J* = 8.6, 1.6 Hz, 6-CH), 7.33-7.21 (6H, *m*, 7-CH, 2'''-CH, 3'''-CH, 4'''-CH, 5'''-CH, 6'''-CH), 5.97 (1H, *d*, *J* = 5.6 Hz, 5"-CH), 5.25 (2H, *s*, 7"-CH_2), 4.28-4.24 (2H, *m*, 11-CH_2), 3.87 (3H, *s*, 9-CH_3), 3.79 (2H, *s*, 10-CH_2), 3.74-3.71 (4H, *m*, 3'-CH_2, 5'-CH_2), 2.53-2.51 (4H, *m*, 2'-CH_2, 6'-CH_2), 1.81 (2H, *ddt*, *J* = 9.2, 7.6, 3.7 Hz, 13-CH_2), 1.38 (2H, *dt*, *J* = 15.2, 7.6 Hz, 12-CH_2), 0.93 (3H, *t*, *J* = 7.4 Hz, 14-CH_3). **^{13}C NMR (101 MHz, $CDCl_3$, *δ ppm*)**: 169.3 (C-4"), 167.6 (C-8), 161.5 (C-2"), 158.1 (C-6"), 152.5 (C-2), 142.0 (C-4a), 139.0 (C-7a), 136.8 (C-1'''), 128.5 (C-3''', C-5'''), 128.0 (C-4'''), 127.9 (C-2''', C-6'''), 124.2 (C-6), 124.2 (C-5), 122.2 (C-4), 109.3 (C-7), 97.1 (C-5"), 67.3 (C-7"), 55.9 (C-10), 53.2 (C-2', C-6'), 52.1 (C-9), 44.3 (C-11), 43.8 (C-3', C-5'), 31.9 (C-13), 20.3 (C-12), 13.8 (C-14). **HRMS** (**ESI+**): found *m/z* 515.2934 $[M + H]^+$; calculated $C_{29}H_{35}N_6O_3^+$ 515.2692.

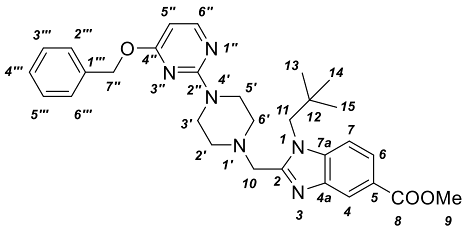

Methyl 2-((4-(4-(benzyloxy)pyrimidin-2-yl)piperazin-1-yl)methyl)-1-neopentyl-1H-benzo[d]imidazole-5-carboxylate **23e**, white solid, 90 mg, 95%. **^{1}H NMR (400 MHz, $CDCl_3$, *δ ppm*)**: 8.38 (1H, *s*, 4-CH), 7.98 (1H, *d*, *J* = 5.6 Hz, 6"-CH), 7.92 (1H, *d*, *J* = 8.6 Hz, 6-CH), 7.36-7.20 (6H, *m*, 7-CH, 2'''-CH, 3'''-CH, 4'''-CH, 5'''-CH, 6'''-CH), 5.96 (1H, *d*, *J* = 5.6 Hz, 5"-CH), 5.24 (2H, *s*, 7"-CH_2), 4.24 (2H, *s*, 11-CH_2), 3.88 (3H, *s*, 9-CH_3), 3.87 (2H, *s*, 10-CH_2), 3.74-3.72 (4H, *m*, 3'-CH_2, 5'-CH_2), 2.49-2.46 (4H, *m*, 2'-CH_2, 6'-CH_2), 0.99 (9H, *s*, 13-CH_3, 14-CH_3, 15-CH_3). **^{13}C NMR (101 MHz, $CDCl_3$, *δ ppm*)**: 169.3 (C-4"), 167.6 (C-8), 161.5 (C-2"), 158.1 (C-6"), 153.3 (C-2), 141.9 (C-4a), 140.3 (C-7a), 136.8 (C-1'''), 128.5 (C-3''', C-5'''), 128.0 (C-4'''), 127.9 (C-2''', C-6'''), 124.1 (C-5), 124.0 (C-6), 122.0 (C-4), 110.8 (C-7), 97.1 (C-5"), 67.3 (C-7"), 56.5 (C-10), 54.9 (C-11), 53.2 (C-2', C-6'), 52.1 (C-9), 43.7 (C-3', C-5'), 35.1 (C-12), 28.9 (C-13, C-14, C-15). **HRMS** (**ESI+**): found *m/z* 529.3086 $[M + H]^+$; calculated $C_{30}H_{37}N_6O_3^+$ 529.2849.

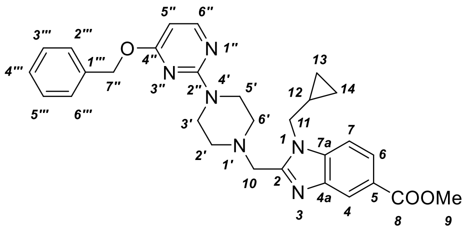

Methyl 2-((4-(4-(benzyloxy)pyrimidin-2-yl)piperazin-1-yl)methyl)-1-(cyclopropylmethyl)-1H-benzo[d]imidazole-5-carboxylate **23f**, white amorphous solid, 100 mg, 95%. **^{1}H NMR (400 MHz, $CDCl_3$, *δ ppm*)**: 8.39 (1H, *s*, 4-CH), 7.98 (1H, *d*, *J* = 5.7 Hz, 6"-CH), 7.95 (1H, *d*, *J* = 8.5 Hz, 6-CH), 7.37-7.19 (7H, *m*, 7-CH, 2'''-CH, 3'''-CH, 4'''-CH, 5'''-CH, 6'''-CH), 5.96 (1H, *d*, *J* = 5.8 Hz, 5"-CH), 5.25 (2H, *s*, 7"-CH_2), 4.20 (2H, *d*, *J* = 6.8 Hz, 11-CH_2), 3.87 (3H, *s*, 9-CH_3), 3.81 (2H, *s*, 10-CH_2), 3.73-3.71 (4H, *m*, 3'-CH_2, 5'-CH_2), 2.53-2.51 (4H, *m*, 2'-CH_2, 6'-CH_2), 1.32-1.26 (1H, *m*, 12-CH), 0.57-0.54 (2H, *m*, 13-CH_2, 14-CH_2), 0.44-0.39 (2H, *m*, 13-CH_2, 14-CH_2). **^{13}C NMR (101 MHz, $CDCl_3$, *δ ppm*)**: 169.3 (C-4"), 167.6 (C-8), 161.5 (C-2"), 158.1 (C-6"), 152.4 (C-2), 142.0 (C-4a), 139.2 (C-7a), 136.8 (C-1'''), 128.5 (C-3''', C-5'''), 128.0 (C-4'''), 127.9 (C-2''', C-6'''), 124.3 (C-6), 124.2 (C-5), 122.1 (C-4), 109.6 (C-7), 97.1 (C-5"), 67.3 (C-7"), 55.9 (C-10), 53.2 (C-2', C-6'), 52.1 (C-9), 48.6 (C-11), 43.7 (C-3', C-5'), 11.2 (C-12), 4.4 (C-13, C-14). **HRMS** (**ESI+**): found *m/z* 513.2782 $[M + H]^+$; calculated $C_{29}H_{33}N_6O_3^+$ 513.2536.

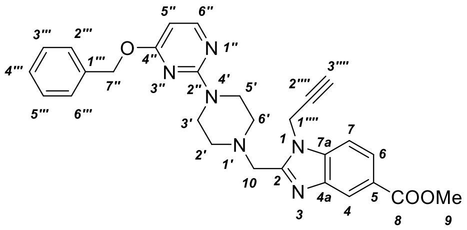

Methyl 2-((4-(4-(benzyloxy)pyrimidin-2-yl)piperazin-1-yl)methyl)-1-(prop-2-yn-1-yl)-1H-benzo[d]imidazole-5-carboxylate **23g**, white amorphous solid, 110 mg, 95%. **^{1}H NMR (400 MHz, $CDCl_3$, *δ ppm*)**: 8.38 (1H, *s*, 4-CH), 8.01-7.98 (2H, *m*, 6-CH, 6"-CH), 7.44 (1H, *d*, *J* = 8.5 Hz, 7-CH), 7.33-7.22 (5H, *m*, 2'''-CH, 3'''-CH, 4'''-CH, 5'''-CH, 6'''-CH), 5.97 (1H, *d*, *J* = 5.6 Hz, 5"-CH), 5.25 (2H, *s*, 7"-CH_2), 5.20 (2H, *d*, *J* = 2.6 Hz, 1''''-CH_2), 3.89 (2H, *s*, 10-CH_2), 3.88 (3H, *s*, 9-CH_3), 3.77-3.74 (4H, *m*, 3'-CH_2, 5'-CH_2), 2.53-2.50 (4H, *m*, 2'-CH_2, 6'-CH_2), 2.31-2.30 (1H, *m*, 3''''-CH). **^{13}C NMR (101 MHz, $CDCl_3$, *δ ppm*)**: 169.2 (C-4"), 167.4 (C-8), 161.4 (C-2"), 158.0 (C-6"), 151.9 (C-2), 141.8 (C-4a), 138.4 (C-7a), 136.7 (C-1'''), 128.4 (C-3''', C-5'''), 127.9 (C-4'''), 127.8 (C-2''', C-6'''), 124.7 (C-6), 124.7 (C-5), 122.1 (C-4), 109.2 (C-7), 97.0 (C-5"), 76.8 (C-2''''), 73.5 (C-3''''), 67.2 (C-7"), 55.9 (C-10), 53.0 (C-2', C-6'), 52.0 (C-9), 43.6 (C-3', C-5'), 33.6 (C-1''''). **HRMS** (**ESI+**): found *m/z* 497.2475 $[M + H]^+$; calculated $C_{28}H_{29}N_6O_3^+$ 497.2223

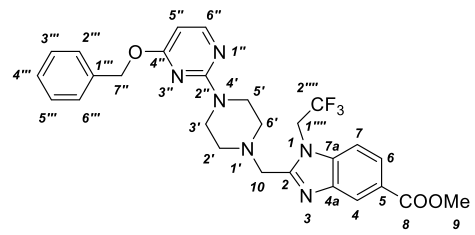

Methyl 2-((4-(4-(benzyloxy)pyrimidin-2-yl)piperazin-1-yl)methyl)-1-(2,2,2-trifluoroethyl)-1H-benzo[d]imidazole-5-carboxylate **23h**, white amorphous solid, 100 mg, 95%. **^{1}H NMR (400 MHz, $CDCl_3$, *δ ppm*)**: 8.40 (1H, *d*, *J* = 1.5 Hz, 4-CH), 8.01 (1H, *dd*, *J* = 8.6, 1.5 Hz, 6-CH), 7.99 (1H, *d*, *J* = 5.6 Hz, 6"-CH), 7.36 (1H, *d*, *J* = 8.6 Hz, 7-CH), 7.33-7.21 (5H, *m*, 2'''-CH, 3'''-CH, 4'''-CH, 5'''-CH, 6'''-CH), 5.98 (1H, *d*, *J* = 5.6 Hz, 5"-CH), 5.25 (2H, *s*, 7"-CH_2), 5.18 (2H, *q*, *J* = 8.7 Hz, 1''''-CH_2), 3.89 (5H, *s*, 10-CH_2, 9-CH_3), 3.75-3.72 (4H, *m*, 3'-CH_2, 5'-CH_2), 2.51-2.49 (4H, *m*, 2'-CH_2, 6'-CH_2). **^{13}C NMR (101 MHz, $CDCl_3$, *δ ppm*)**: 169.3 (C-4"), 167.2 (C-8), 161.4 (C-2"), 158.1 (C-6"), 152.2 (C-2), 141.9 (C-4a), 139.1 (C-7a), 136.8 (C-1'''), 128.5 (C-3''', C-5'''), 128.0 (C-4'''), 127.9 (C-2''', C-6'''), 125.4 (C-6), 125.4 (C-5), 123.2 (C-2'''', *q*, $J_{(C-F)}$ = 280.2 Hz), 122.4 (C-4), 109.2 (C-7), 97.3 (C-5"), 67.3 (C-7"), 56.0 (C-10), 53.2 (C-2', C-6'), 52.2 (C-9), 45.2 (C-1'''', *q*, $J_{(C-F)}$ = 35.9 Hz), 43.7 (C-3', C-5'). **HRMS (ESI+)**: found *m/z* 541.2341 $[M + H]^+$; calculated $C_{27}H_{28}F_3N_6O_3^+$ 541.2097.

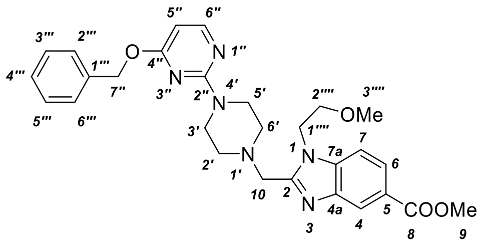

Methyl 2-((4-(4-(benzyloxy)pyrimidin-2-yl)piperazin-1-yl)methyl)-1-(2-methoxyethyl)-1H-benzo[d]imidazole-5-carboxylate **23i**, white amorphous solid, 85 mg, 92%. **^{1}H NMR (400 MHz, $CDCl_3$, *δ ppm*)**: 8.38 (1H, *s*, 4-CH), 7.99 (1H, *d*, *J* = 5.6 Hz, 6"-CH), 7.95 (1H, *dd*, *J* = 8.5, 1.6 Hz, 6-CH), 7.37-7.21 (6H, *m*, 7-CH, 2'''-CH, 3'''-CH, 4'''-CH, 5'''-CH, 6'''-CH), 5.97 (1H, *d*, *J* = 5.6 Hz, 5"-CH), 5.25 (2H, *s*, 7"-CH_2), 4.51 (2H, *t*, *J* = 5.4 Hz, 1''''-CH_2), 3.88 (3H, *s*, 9-CH_3), 3.85 (2H, *s*, 10-CH_2), 3.74-3.71 (4H, *m*, 3'-CH_2, 5'-CH_2), 3.69 (2H, *t*, *J* = 5.4 Hz, 2''''-CH_2), 3.22 (3H, *s*, 3''''-CH_3), 2.53-2.50 (4H, *m*, 2'-CH_2, 6'-CH_2). **^{13}C NMR (101 MHz, $CDCl_3$, *δ ppm*)**: 169.3 (C-4"), 167.6 (C-8), 161.5 (C-2"), 158.1 (C-6"), 153.2 (C-2), 141.9 (C-4a), 139.2 (C-7a), 136.8 (C-1'''), 128.5 (C-3''', C-5'''), 128.0 (C-4'''), 127.9 (C-2''', C-6'''), 124.3 (C-6), 124.3 (C-5), 122.1 (C-4), 109.5 (C-7), 97.1 (C-5"), 71.3 (C-2''''), 67.3 (C-7"), 59.2 (C-3''''), 55.8 (C-10), 53.1 (C-2', C-6'), 52.1 (C-9), 44.3 (C-1''''), 43.8 (C-3', C-5'). **HRMS (ESI+)**: found *m/z* 517.2728 $[M + H]^+$; calculated $C_{28}H_{33}N_6O_4^+$ 517.2485.

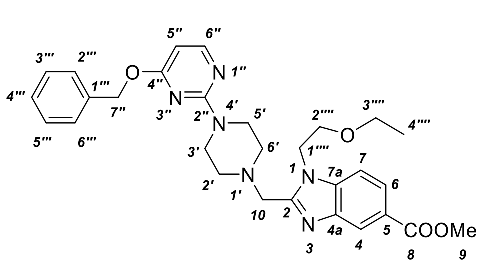

Methyl 2-((4-(4-(benzyloxy)pyrimidin-2-yl)piperazin-1-yl)methyl)-1-(2-ethoxyethyl)-1H-benzo[d]imidazole-5-carboxylate **23j**, white amorphous solid, 95 mg, 92%. **^{1}H NMR (400 MHz, $CDCl_3$, *δ ppm*)**: 8.38 (1H, *d*, *J* = 1.6 Hz, 4-CH), 7.99 (1H, *d*, *J* = 5.6 Hz, 6"-CH), 7.94 (1H, *dd*, *J* = 8.5, 1.6 Hz, 6-CH), 7.37 (1H, *d*, *J* = 8.6 Hz, 7-CH), 7.33-7.21 (5H, *m*, 2'''-CH, 3'''-CH, 4'''-CH, 5'''-CH, 6'''-CH), 5.97 (1H, *d*, *J* = 5.6 Hz, 5"-CH), 5.25 (2H, *s*, 7"-CH_2), 4.51 (2H, *t*, *J* = 5.4 Hz, 1''''-CH_2), 3.87 (3H, *s*, 9-CH_3), 3.86 (2H, *s*, 10-CH_2), 3.74-3.71 (6H, *m*, 2''''-CH_2, 3'-CH_2, 5'-CH_2), 3.34 (2H, *q*, *J* = 7.0 Hz, 3''''-CH_2), 2.53-2.51 (4H, *m*, 2'-CH_2, 6'-CH_2), 1.04 (3H, *t*, *J* = 7.0 Hz, 4''''-CH_3). **^{13}C NMR (101 MHz, $CDCl_3$, *δ ppm*)**: 169.3 (C-4"), 167.7 (C-8), 161.5 (C-2"), 158.1 (C-6"), 153.3 (C-2), 141.9 (C-4a), 139.2 (C-7a), 136.8 (C-1'''), 128.5 (C-3''', C-5'''), 128.0 (C-4'''), 127.9 (C-2''', C-6'''), 124.3 (C-6, C-5), 122.1 (C-4), 109.6 (C-7), 97.1 (C-5"), 69.2 (C-2''''), 67.3 (C-7"), 66.9 (C-3''''), 55.8 (C-10), 53.2 (C-2', C-6'), 52.1 (C-9), 44.5 (C-1''''), 43.8 (C-3', C-5'), 15.1 (C-4''''). **HRMS (ESI+)**: found *m/z* 531.2882 $[M + H]^+$; calculated $C_{29}H_{35}N_6O_4^+$ 531.2642

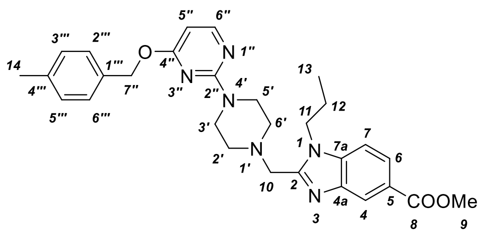

Methyl 2-((4-(4-((4-methylbenzyl)oxy)pyrimidin-2-yl)piperazin-1-yl)methyl)-1-propyl-1H-benzo[d]imidazole-5-carboxylate **23k**, white amorphous solid, 70 mg, 92%. **^{1}H NMR (400 MHz, $CDCl_3$, *δ ppm*)**: 8.38 (1H, *d*, *J* = 1.5 Hz, 4-CH), 7.98 (1H, *d*, *J* = 5.6 Hz, 6"-CH), 7.94 (1H, *dd*, *J* = 8.6, 1.6 Hz, 6-CH), 7.32 (1H, *d*, *J* = 8.6 Hz, 7-CH), 7.22-7.20 (2H, *m*, 2'''-CH, 6'''-CH), 7.10-7.08 (2H, *m*, 2'''-CH, 6'''-CH), 5.95 (1H, *d*, *J* = 5.6 Hz, 5"-CH), 5.20 (2H, *s*, 7"-CH_2), 4.25-4.21 (2H, *m*, 11-CH_2), 3.87 (3H, *s*, 9-CH_3), 3.80 (2H, *s*, 10-CH_2), 3.74-3.72 (4H, *m*, 3'-CH_2, 5'-CH_2), 2.54-2.52 (4H, *m*, 2'-CH_2, 6'-CH_2), 2.27 (3H, *s*, 14-CH_3), 1.87 (2H, *h*, *J* = 7.5 Hz, 12-CH_2), 0.96 (3H, *t*, *J* = 7.4 Hz, 13-CH_3). **^{13}C NMR (101 MHz, $CDCl_3$, *δ ppm*)**: 169.4 (C-4"), 167.6 (C-8), 161.5 (C-2"), 158.0 (C-6"), 152.6 (C-2), 141.9 (C-4a), 139.0 (C-7a), 137.8 (C-1'''), 133.7 (C-4'''), 129.2 (C-3''', C-5'''), 128.1 (C-2''', C-6'''), 124.3 (C-6), 124.2 (C-5), 122.1 (C-4), 109.3 (C-7), 97.2 (C-5"), 67.2 (C-7"), 55.9 (C-10), 53.2 (C-2', C-6'), 52.1 (C-9), 46.0 (C-11), 43.8 (C-3', C-5'), 23.2 (C-12), 21.2 (C-14), 11.5 (C-13). **HRMS (ESI+)**: found *m/z* 515.2754 $[M + H]^+$; calculated $C_{29}H_{35}N_6O_3^+$ 515.2692.

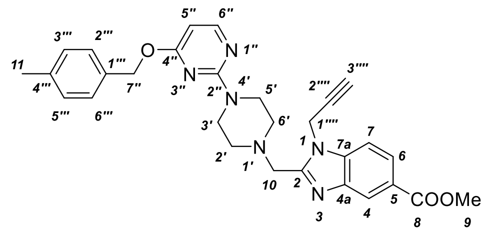

Methyl 2-((4-(4-((4-methylbenzyl)oxy)pyrimidin-2-yl)piperazin-1-yl)methyl)-1-(prop-2-yn-1-yl)-1H-benzo[d]imidazole-5-carboxylate **23l**, white amorphous solid, 65 mg, 90%. **^{1}H NMR (400 MHz, $CDCl_3$, *δ ppm*)**: 8.39 (1H, *d*, *J* = 1.5 Hz, 4-CH), 8.01-7.97 (2H, *m*, 6-CH, 6''-CH), 7.45 (1H, *d*, *J* = 8.5 Hz, 7-CH), 7.22-7.20 (2H, *m*, 2'''-CH, 6'''-CH), 7.10-7.08 (2H, *m*, 3'''-CH, 5'''-CH), 5.95 (1H, *d*, *J* = 5.6 Hz, 5''-CH), 5.20 (2H, *s*, 7''-CH_2), 5.20 (2H, *d*, *J* = 2.6 Hz, 1''''-CH_2), 3.90 (2H, *s*, 10-CH_2), 3.88 (3H, *s*, 9-CH_3), 3.77-3.75 (4H, *m*, 3'-CH_2, 5'-CH_2), 2.53-2.51 (4H, *m*, 2'-CH_2, 6'-CH_2), 2.31 (1H, *t*, *J* = 2.5 Hz, 3''''-CH), 2.27 (11-CH_3). **^{13}C NMR (101 MHz, $CDCl_3$, *δ ppm*)**: 169.4 (C-4''), 167.5 (C-8), 161.5 (C-2''), 158.0 (C-6''), 152.0 (C-2), 141.8 (C-4a), 138.5 (C-7a), 137.8 (C-1'''), 133.7 (C-4'''), 129.2 (C-3''', C-5'''), 128.1 (C-2''', C-6'''), 124.8 (C-6), 124.8 (C-5), 122.2 (C-4), 109.3 (C-7), 97.2 (C-5''), 76.9 (C-2''''), 73.6 (C-3''''), 67.3 (C-7''), 56.0 (C-10), 53.1 (C-2', C-6'), 52.1 (C-9), 42.7 (C-3', C-5'), 33.7 (C-1''''), 21.2 (C-11). **HRMS (ESI+)**: found *m/z* 511.2440 $[M + H]^+$; calculated $C_{29}H_{31}N_6O_3^+$ 511.2379.

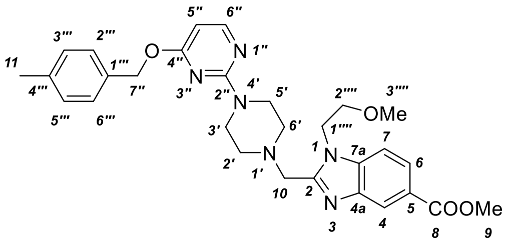

Methyl 1-(2-methoxyethyl)-2-((4-(4-((4-methylbenzyl)oxy)pyrimidin-2-yl)piperazin-1-yl)methyl)-1H-benzo[d]imidazole-5-carboxylate **23m**, white amorphous solid, 80 mg, 90%. **^{1}H NMR (400 MHz, $CDCl_3$, *δ ppm*)**: 8.39 (1H, *d*, *J* = 1.4 Hz, 4-CH), 7.98 (1H, *d*, *J* = 5.7 Hz, 6''-CH), 7.95 (1H, *dd*, *J* = 8.5, 1.6 Hz, 6-CH), 7.36 (1H, *d*, *J* = 8.6 Hz, 7-CH), 7.23-7.20 (2H, *m*, 2'''-CH, 6'''-CH), 7.12-7.08 (2H, *m*, 3'''-CH, 5'''-CH), 5.95 (1H, *d*, *J* = 5.6 Hz, 5''-CH), 5.20 (2H, *s*, 7''-CH_2), 4.52 (2H, *t*, *J* = 5.4 Hz, 1''''-CH_2), 3.88 (3H, *s*, 9-CH_3), 3.87 (2H, *s*, 10-CH_2), 3.74-3.72 (4H, *m*, 3'-CH_2, 5'-CH_2), 3.69 (2H, *t*, *J* = 5.3 Hz, 2''''-CH_2), 3.22 (3H, *s*, 3''''-CH_3), 2.54-2.51 (4H, *m*, 2'-CH_2, 6'-CH_2), 2.27 (3H, *s*, 11-CH_3). **^{13}C NMR (101 MHz, $CDCl_3$, *δ ppm*)**: 169.4 (C-4''), 167.6 (C-8), 161.5 (C-2''), 157.9 (C-6''), 153.2 (C-2), 141.7 (C-4a), 139.0 (C-7a), 137.8 (C-1'''), 133.7 (C-4'''), 129.2 (C-3''', C-5'''), 128.1 (C-2''', C-6'''), 124.4 (C-6), 124.4 (C-5), 122.0 (C-4), 109.5 (C-7), 97.2 (C-5''), 71.3 (C-2''''), 67.3 (C-7''), 59.2 (C-3''''), 55.6 (C-10), 53.1 (C-2', C-6'), 52.1 (C-9), 44.4 (C-1''''), 43.8 (C-3', C-5'), 21.2 (C-11). **HRMS (ESI+)**: found *m/z* 531.2703 $[M + H]^+$; calculated $C_{29}H_{35}N_6O_4^+$ 531.2642.

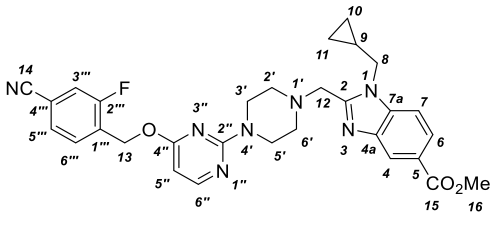

Methyl 2-((4-(4-((4-cyano-2-fluorobenzyl)oxy)pyrimidin-2-yl)piperazin-1-yl)methyl)-1-cyclopropylmethyl-1H-benzo[d]imidazole-5-carboxylate **23n**, white amorphous solid, 90 mg, 90%. **^{1}H NMR (400 MHz, $CDCl_3$, *δ ppm*)**: 8.39-8.38 (1H, *m*, 4-CH), 8.02 (1H, *d*, *J* = 5.6 Hz, 6''-CH), 7.95 (1H, *dd*, *J* = 8.5, 1.6 Hz, 6-CH), 7.49 (1H, *t*, *J* = 7.5 Hz, 6'''-CH), 7.42-7.34 (2H, *m*, 5'''-CH, 7-CH), 7.29 (1H, *dd*, *J* = 9.3, 1.5 Hz, 3'''-CH), 5.99 (1H, *d*, *J* = 5.6 Hz, 5''-CH), 5.37 (2H, *s*, 13-CH_2), 4.20 (2H, *d*, *J* = 6.8 Hz, 8-CH_2), 3.88 (3H, *s*, 16-CH_3), 3.80 (2H, *s*, 12-CH_2), 3.69 (4H, *t*, *J* = 5.0 Hz, 3'-CH_2, 5'-CH_2), 2.51 (4H, *t*, *J* = 5.1 Hz, 2'-CH_2, 6'-CH_2), 1.34-1.23 (1H, *m*, 9-CH), 0.64-0.52 (2H, *m*, 10-CH_2, 11-CH_2), 0.42-0.38 (2H, *m*, 10-CH_2, 11-CH_2). **^{13}C NMR (101 MHz, $CDCl_3$, *δ ppm*)**: 168.6 (C-4''), 167.6 (C-15), 161.4 (C-2''), 159.7 (C-2''', *d*, $J_{(C-F)}$ = 251.0 Hz), 158.6 (C-6''), 152.3 (C-2), 141.9 (C-4a), 139.1 (C-7a), 130.4 (C-1''', *d*, $J_{(C-F)}$ = 14.4 Hz), 130.3 (C-6''', *d*, $J_{(C-F)}$ = 4.6 Hz), 128.3 (C-5''', *d*, $J_{(C-F)}$ = 3.9 Hz), 124.3 (C-6), 124.2 (C-5), 122.1 (C-4), 119.0 (C-3''', *d*, $J_{(C-F)}$ = 24.9 Hz), 117.4 (C-14, *d*, $J_{(C-F)}$ = 2.9 Hz), 113.0 (C-4''', *d*, $J_{(C-F)}$ = 9.5 Hz), 109.7 (C-7), 96.7 (C-5''), 60.0 (C-13, *d*, $J_{(C-F)}$ = 4.7 Hz), 55.8 (C-12), 53.1 (C-2', C-6'), 52.1 (C-16), 48.6 (C-8), 43.7 (C-3', C-5'), 11.2 (C-9), 4.4 (C-10, C-11). **HRMS (ESI+)**: found *m/z* 556.2488 $[M + H]^+$; calculated $C_{30}H_{31}FN_7O_3^+$ 556.2394.

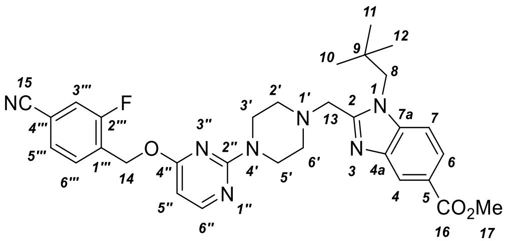

Methyl 2-((4-(4-((4-cyano-2-fluorobenzyl)oxy)pyrimidin-2-yl)piperazin-1-yl)methyl)-1-neopentyl-1H-benzo[d]imidazole-5-carboxylate **23o**, white amorphous solid, 80 mg, 95%. **^{1}H NMR (400 MHz, $CDCl_3$, *δ ppm*)**: 8.38 (1H, *d*, *J* = 1.5 Hz, 4-CH), 8.02 (1H, *d*, *J* = 5.6 Hz, 6''-CH), 7.92 (1H, *dd*, *J* = 8.6, 1.6 Hz, 6-CH), 7.49 (1H, *t*, *J* = 7.5 Hz, 6'''-CH), 7.38 (1H, *dd*, *J* = 7.9, 1.5 Hz, 5'''-CH), 7.35 (1H, *d*, *J* = 8.6 Hz, 7-CH), 7.29 (1H, *dd*, *J* = 9.3, 1.5 Hz, 3'''-CH), 5.99 (1H, *d*, *J* = 5.6 Hz, 5''-CH), 5.36 (2H, *s*, 14-CH_2), 4.23 (2H, *s*, 8-CH_2), 3.87 (5H, *s*, 13-CH_2, 17-CH_3), 3.71-3.69 (4H, *m*, 3'-CH_2, 5'-CH_2), 2.48-2.45 (4H, *m*, 2'-CH_2, 6'-CH_2), 0.99 (9H, *s*, 10-CH_3, 11-CH_3, 12-CH_3). **^{13}C NMR (101 MHz, $CDCl_3$, *δ ppm*)**: 168.6 (C-4''), 167.6 (C-16), 161.4 (C-2''), 159.7 (C-2''', *d*, $J_{(C-F)}$ = 251.4 Hz), 158.6 (C-6''), 153.2 (C-2), 141.9 (C-4a), 140.3 (C-7a), 130.4 (C-1''', *d*, $J_{(C-F)}$ = 14.4 Hz), 130.3 (C-6''', *d*, $J_{(C-F)}$ = 4.7 Hz), 128.3 (C-5''', *d*, $J_{(C-F)}$ = 3.8 Hz), 124.2 (C-5), 124.0 (C-6), 122.0 (C-4), 119.0 (C-3''', *d*, $J_{(C-F)}$ = 24.7 Hz), 117.4 (C-15, *d*, $J_{(C-F)}$ = 2.9 Hz), 113.0 (C-4''', *d*, $J_{(C-F)}$ = 9.5 Hz), 110.8 (C-7), 96.9 (C-5''), 59.9 (C-14, *d*, $J_{(C-F)}$ = 4.8 Hz), 56.4 (C-13), 55.0 (C-8), 53.1 (C-2', C-6'), 52.1 (C-17), 43.7 (C-3', C-5'), 35.1 (C-9), 28.9 (C-10, C-11, C-12). **HRMS (ESI+)**: found *m/z* 572.2805 $[M + H]^+$; calculated $C_{31}H_{35}FN_7O_3^+$ 572.2707.

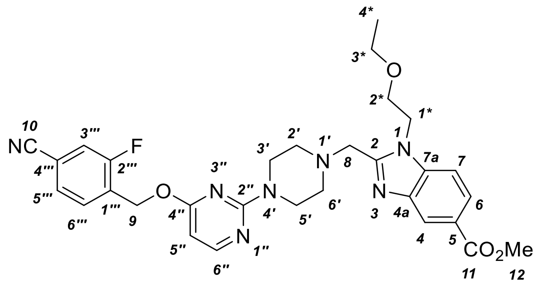

Methyl 2-((4-(4-((4-cyano-2-fluorobenzyl)oxy)pyrimidin-2-yl)piperazin-1-yl)methyl)-1-(2-ethoxyethyl)-1H-benzo[d]imidazole-5-carboxylate **23p**, white amorphous solid, 95 mg, 90%. **^{1}H NMR (400 MHz, $CDCl_3$, *δ ppm*)**: 8.38 (1H, *dd*, *J* = 1.5, 0.6 Hz, 4-CH), 8.02 (1H, *d*, *J* = 5.6 Hz, 6"-CH), 7.94 (1H, *dd*, *J* = 8.5, 1.6 Hz, 6-CH), 7.49 (1H, *t*, *J* = 7.5 Hz, 5'''-CH), 7.39-7.35 (2H, *m*, 6'''-CH, 7-CH), 7.29 (1H, *dd*, *J* = 9.3, 1.5 Hz, 3'''-CH), 5.99 (1H, *d*, *J* = 5.6 Hz, 5"-CH), 5.37 (2H, *s*, 9-CH_2), 4.50 (2H, *t*, *J* = 5.4 Hz, 1*-CH_2), 3.88 (3H, *s*, 12-CH_3) 3.86 (2H, *s*, 8-CH_2), 3.73-3.68 (6H, *m*, 2*-CH_2, 3'-CH_2, 5'-CH_2), 3.34 (2H, *q*, *J* = 7.0 Hz, 3*-CH_2), 2.52-2.50 (4H, *m*, 2'-CH_2, 6'-CH_2), 1.04 (3H, *t*, *J* = 7.0 Hz, 4*-CH_3). **^{13}C NMR (101 MHz, $CDCl_3$, *δ ppm*)**: 168.6 (C-4"), 167.6 (C-11), 161.4 (C-2"), 159.7 (C-2''', *d*, $J_{(C-F)}$ = 250.9 Hz), 158.6 (C-6"), 153.2 (C-2), 142.0 (C-4a), 139.2 (C-7a), 130.4 (C-1''', *d*, $J_{(C-F)}$ = 14.8 Hz), 130.4 (C-6''', *d*, $J_{(C-F)}$ = 4.4 Hz), 128.3 (C-5''', *d*, $J_{(C-F)}$ = 4.1 Hz), 124.3 (C-5, C-6), 122.1 (C-4), 119.0 (C-3''', *d*, $J_{(C-F)}$ = 24.7 Hz), 117.4 (C-10, *d*, $J_{(C-F)}$ = 3.3 Hz), 113.1 (C-4''', *d*, $J_{(C-F)}$ = 9.5 Hz), 109.5 (C-7), 96.7 (C-5"), 69.1 (C-2*), 66.9 (C-3*), 60.0 (C-9, *d*, $J_{(C-F)}$ = 4.5 Hz), 55.8 (C-8), 53.1 (C-2', C-6'), 52.1 (C-12), 44.5 (C-1*), 43.7 (C-3', C-5'), 15.1 (C-4*). **HRMS (ESI+)**: found *m/z* 574.1516 $[M + H]^+$; calculated $C_{30}H_{33}FN_7O_4^+$ 574.2500.

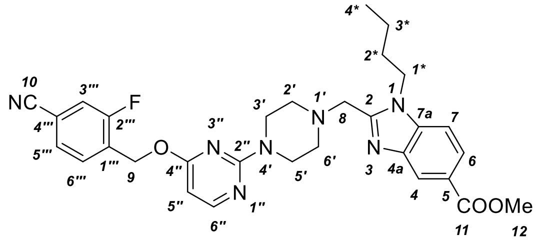

Methyl 2-((4-(4-((4-cyano-2-fluorobenzyl)oxy)pyrimidin-2-yl)piperazin-1-yl)methyl)-1-butyl-1H-benzo[d]imidazole-5-carboxylate **23q**, white amorphous solid, 100 mg, 90%. **^{1}H NMR (400 MHz, $CDCl_3$, *δ ppm*)**: 8.38 (1H, *d*, *J* = 1.5 Hz, 4-CH), 8.02 (1H, *d*, *J* = 5.6 Hz, 6"-CH), 7.95 (1H, *dd*, *J* = 8.5, 1.6 Hz, 6-CH), 7.49 (1H, *t*, *J* = 7.5 Hz, 5'''-CH), 7.38 (1H, *dd*, *J* = 8.0, 1.5 Hz, 5'''-CH), 7.32-7.28 (2H, *m*, 7-CH, 3'''-CH), 5.99 (1H, *d*, *J* = 5.6 Hz, 5"-CH), 5.37 (2H, *s*, 9-CH_2), 4.27-4.23 (2H, *m*, 1*-CH_2), 3.88 (3H, *s*, 12-CH_3), 3.79 (2H, *s*, 8-CH_2), 3.71-3.68 (4H, *m*, 3'-CH_2, 5'-CH_2), 2.52-2.50 (4H, *m*, 2'-CH_2, 6'-CH_2), 1.81 (2H, *ddt*, *J* = 9.2, 7.6, 3.7 Hz, 2*-CH_2), 1.38 (2H, *h*, *J* = 7.4 Hz, 3*-CH_2), 0.93 (3H, *t*, *J* = 7.4 Hz, 4*-CH_3). **^{13}C NMR (101 MHz, $CDCl_3$, *δ ppm*)**: 168.6 (C-4"), 167.6 (C-11), 161.4 (C-2"), 159.7 (C-2''', *d*, $J_{(C-F)}$ = 251.0 Hz), 158.6 (C-6"), 152.4 (C-2), 142.0 (C-4a), 139.0 (C-7a), 130.4 (C-1''', *d*, $J_{(C-F)}$ = 13.4 Hz), 130.3 (C-5''', *d*, $J_{(C-F)}$ = 4.8 Hz), 128.3 (C-6''', *d*, $J_{(C-F)}$ = 4.1 Hz), 124.3 (C-6), 124.2 (C-5), 122.2 (C-4), 119.0 (C-3''', *d*, $J_{(C-F)}$ = 24.9 Hz), 117.4 (C-10, *d*, $J_{(C-F)}$ = 2.9 Hz), 113.1 (C-4''', *d*, $J_{(C-F)}$ = 9.5 Hz), 109.3 (C-7), 96.7 (C-5"), 60.0 (C-9, *d*, $J_{(C-F)}$ = 4.5 Hz), 55.9 (C-8), 53.1 (C-2', C-6'), 52.1 (C-12), 44.2 (C-1*), 43.7 (C-3', C-5'), 31.9 (C-2*), 20.3 (C-3*), 13.8 (C-4*). **HRMS (ESI+)**: found *m/z* 558.2621 $[M + H]^+$; calculated $C_{30}H_{33}FN_7O_3^+$ 558.2551.

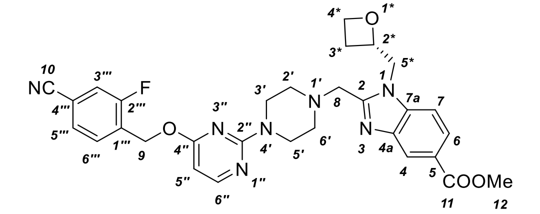

Methyl (S)-2-((4-(4-((4-cyano-2-fluorobenzyl)oxy)pyrimidin-2-yl)piperazin-1-yl)methyl)-1-(oxetan-2-ylmethyl)-1H-benzo[d]imidazole-5-carboxylate **23r**, white amorphous solid, 115 mg, 95%. **^{1}H NMR (400 MHz, $CDCl_3$, *δ ppm*)**: 8.39 (1H, *d*, *J* = 1.4 Hz, 4-CH), 8.02 (1H, *d*, *J* = 5.6 Hz, 6"-CH), 7.95 (1H, *dd*, *J* = 8.5, 1.6 Hz, 7-CH), 7.49 (1H, *t*, *J* = 7.5 Hz, 6'''-CH), 7.41-7.37 (2H, *m*, 6'''-CH, 6-CH) 7.29 (1H, *dd*, *J* = 9.3, 1.5 Hz, 3'''-CH), 5.99 (1H, *d*, *J* = 5.6 Hz, 5"-CH), 5.37 (2H, *s*, 9-CH_2), 5.19-5.13 (1H, *m*, 2*-CH), 4.60 (2H, *d*, *J* = 4.4 Hz, 5*-CH_2), 4.55 (1H, *td*, *J* = 8.0, 6.0 Hz, 4*-CH_2), 4.29 (1H, *dt*, *J* = 9.2, 5.9 Hz, 4*-CH_2), 3.90 (2H, *q*, *J* = 13.5 Hz, 8-CH_2), 3.88 (3H, *s*, 12-CH_3), 3.70-3.68 (4H, *m*, 3'-CH_2, 5'-CH_2), 2.65 (1H, *dtd*, *J* = 11.3, 8.0, 5.9 Hz, 3*-CH_2), 2.50 (4H, *t*, *J* = 5.1 Hz, 2'-CH_2, 6'-CH_2), 2.37 (1H, *ddt*, *J* = 11.3, 9.1, 7.3 Hz, 3*-CH_2). **^{13}C NMR (101 MHz, $CDCl_3$, *δ ppm*)**: 168.6 (C-4"), 167.6 (C-11), 161.4 (C-2"), 159.7 (C-2''', *d*, $J_{(C-F)}$ = 250.8 Hz), 158.6 (C-6"), 153.2 (C-2), 141.9 (C-4a), 139.6 (C-7a), 130.4 (C-1''', *d*, $J_{(C-F)}$ = 14.4 Hz), 130.3 (C-6''', *d*, $J_{(C-F)}$ = 4.6 Hz), 128.3 (C-5''', *d*, $J_{(C-F)}$ = 3.7 Hz), 124.5 (C-6), 124.5 (C-5), 122.2 (C-4), 119.0 (C-3''', *d*, $J_{(C-F)}$ = 24.8 Hz), 117.4 (C-10, *d*, $J_{(C-F)}$ = 3.0 Hz), 113.1 (C-4''', *d*, $J_{(C-F)}$ = 9.4 Hz), 109.7 (C-7), 96.7 (C-5"), 81.0 (C-2*), 68.3 (C-4*), 60.0 (C-9, *d*, $J_{(C-F)}$ = 4.6 Hz), 55.8 (C-8), 53.1 (C-2', C-6'), 52.1 (C-12), 49.4 (C-5*), 43.7 (C-3', C-5'), 24.8 (C-3*). **HRMS (ESI+)**: found *m/z* 572.2421 $[M + H]^+$; calculated $C_{30}H_{31}FN_7O_4^+$ 572.2343.

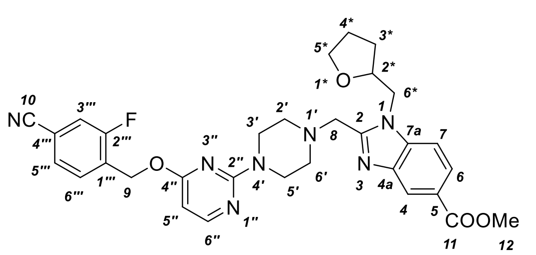

Methyl 2-((4-(4-((4-cyano-2-fluorobenzyl)oxy)pyrimidin-2-yl)piperazin-1-yl)methyl)-1-((tetrahydrofuran-2-yl)methyl)-1H-benzo[d]imidazole-5-carboxylate **23s**, white amorphous solid, 120 mg, 90%.**^{1}H NMR (400 MHz, CDCl$_3$, *δ ppm*)**: 8.37 (1H, *d*, *J* = 1.4 Hz, 4-CH), 8.02 (1H, *d*, *J* = 5.6 Hz, 6"-CH), 7.94 (1H, *dd*, *J* = 8.5, 1.6 Hz, 6-CH), 7.49 (1H, *t*, *J* = 7.5 Hz, 6'''-CH), 7.39-7.37 (2H, *m*, 5'''-CH, 7-CH) 7.29 (1H, *dd*, *J* = 9.3, 1.5 Hz, 3'''-CH), 5.99 (1H, *d*, *J* = 5.6 Hz, 5"-CH), 5.37 (2H, *s*, 9-CH_2), 4.48-4.37 (2H, *m*, 6*-CH_2), 4.26-4.19 (1H, *m*, 2*-CH), 3.93-3.81 (2H, *m*, 8-CH_2), 3.87 (3H, *s*, 12-CH_3), 3.81-3.76 (1H, *m*, 5*-CH_2), 3.71-3.65 (5H, *m*, 3'-CH_2, 5'-CH_2, 5*-CH_2), 2.54-2.49 (4H, *m*, 2'-CH_2, 6'-CH_2), 2.04-1.96 (1H, *m*, 3*-CH_2), 1.87-1.76 (2H, *m*, 4*-CH_2), 1.61-1.52 (1H, *m*, 3*-CH_2). **^{13}C NMR (101 MHz, CDCl$_3$, *δ ppm*)**: 168.6 (C-4"), 167.6 (C-11), 161.4 (C-2"), 159.7 (C-2''', *d*, $J_{(C-F)}$ = 250.8 Hz), 158.6 (C-6"), 153.1 (C-2), 141.9 (C-4a), 139.5 (C-7a), 130.4 (C-1''', *d*, $J_{(C-F)}$ = 14.7 Hz), 130.3 (C-6''', *d*, $J_{(C-F)}$ = 4.5 Hz), 128.3 (C-5''', *d*, $J_{(C-F)}$ = 4.0 Hz), 124.3 (C-6), 124.3 (C-5), 122.1 (C-4), 119.0 (C-3''', *d*, $J_{(C-F)}$ = 24.8 Hz), 117.4 (C-10, *d*, $J_{(C-F)}$ = 2.9 Hz), 113.1 (C-4''', *d*, $J_{(C-F)}$ = 9.5 Hz), 109.8 (C-7), 96.7 (C-5"), 78.6 (C-2*), 68.2 (C-5*), 60.0 (C-9, *d*, $J_{(C-F)}$ = 4.9 Hz), 55.9 (C-8), 53.1 (C-2', C-6'), 52.1 (C-12), 48.1 (C-6*), 43.7 (C-3', C-5'), 29.3 (C-3*), 25.7 (C-4*). **HRMS (ESI+)**: found *m/z* 586.2580 [M + H]$^+$; calculated $C_{31}H_{33}FN_7O_4^+$ 586.2500.

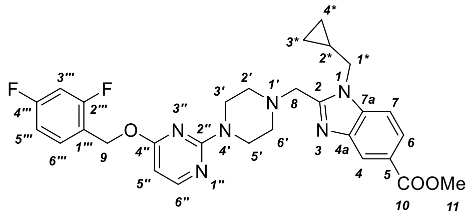

Methyl 1-(cyclopropylmethyl)-2-((4-(4-((2,4-difluorobenzyl)oxy)pyrimidin-2-yl)piperazin-1-yl)methyl)-1H-benzo[d]imidazole-5-carboxylate **23t**, white amorphous solid, 90 mg, 90%. **^{1}H NMR (400 MHz, CDCl$_3$, *δ ppm*)**: 8.39 (1H, *d*, *J* = 1.5 Hz, 4-CH), 7.99 (1H, *d*, *J* = 5.6 Hz, 6"-CH), 7.95 (1H, *dd*, *J* = 8.5, 1.5 Hz, 6-CH), 7.37-7.30 (2H, *m*, 6'''-CH, 7-CH), 6.82-6.72 (2H, *m*, 5'''-CH, 3'''-CH), 5.95 (1H, *d*, *J* = 5.6 Hz, 5"-CH), 5.27 (2H, *s*, 9-CH_2), 4.21 (2H, *d*, *J* = 6.7 Hz, 1*-CH_2), 3.88 (3H, *s*, 11-CH_3), 3.81 (2H, *s*, 8-CH_2), 3.73 (4H, *t*, *J* = 5.0 Hz, 3'-CH_2, 5'-CH_2), 2.53 (4H, *t*, *J* = 5.1 Hz, 2'-CH_2, 6'-CH_2), 1.32-1.25 (1H, *m*, 2*-CH), 0.59-0.54 (2H, *m*, 3*-CH_2, 4*-CH_2), 0.42-0.38 (2H, *m*, 3*-CH_2, 4*-CH_2). **^{13}C NMR (101 MHz, CDCl$_3$, *δ ppm*)**: 169.0 (C-4"), 167.6 (C-10), 162.8 (C-4''', *dd*, $J_{(C-F)}$ = 249.3, 12.0 Hz), 161.5 (C-2"), 160.9 (C-2''', *dd*, $J_{(C-F)}$ = 250.4, 11.9 Hz), 158.2 (C-6"), 152.4 (C-2), 142.0 (C-4a), 139.2 (C-7a), 131.2 (C-6''', *dd*, $J_{(C-F)}$ = 9.7, 5.6 Hz), 124.3 (C-6), 124.2 (C-5), 122.2 (C-4), 120.0 (C-1''', *dd*, $J_{(C-F)}$ = 14.8, 4.0 Hz), 111.3 (C-5''', *dd*, $J_{(C-F)}$ = 21.2, 3.8 Hz), 109.6 (C-7), 103.9 (C-3''', *t*, $J_{(C-F)}$ = 25.4 Hz), 96.9 (C-5"), 60.4 (C-9, *d*, $J_{(C-F)}$ = 3.8 Hz), 55.9 (C-8), 53.2 (C-2', C-6'), 52.1 (C-11), 48.6 (C-1*), 43.7 (C-3', C-5'), 11.2 (C-2*), 4.4 (C-3*, C-4*). **HRMS (ESI+)**: found *m/z* 549.2423 [M + H]$^+$; calculated $C_{29}H_{31}F_2N_6O_3^+$ 549.2347.

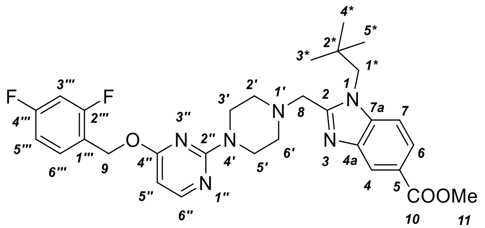

Methyl 2-((4-(4-((2,4-difluorobenzyl)oxy)pyrimidin-2-yl)piperazin-1-yl)methyl)-1-neopentyl-1H-benzo[d]imidazole-5-carboxylate **23u**, white amorphous solid, 110 mg, 95%. **^{1}H NMR (400 MHz, CDCl$_3$, *δ ppm*)**: 8.38 (1H, *d*, *J* = 1.5 Hz, 4-CH), 7.99 (1H, *d*, *J* = 5.6 Hz, 6"-CH), 7.92 (1H, *dd*, *J* = 8.6, 1.6 Hz, 6-CH), 7.36-7.30 (2H, *m*, 6'''-CH, 7-CH), 6.82-6.72 (2H, *m*, 5'''-CH, 3'''-CH), 5.94 (1H, *d*, *J* = 5.6 Hz, 5"-CH), 5.27 (2H, *s*, 9-CH_2), 4.24 (2H, *s*, 1*-CH_2), 3.88 (2H, *s*, 8-CH_2), 3.87 (3H, *s*, 11-CH_3), 3.74 (4H, *t*, *J* = 5.0 Hz, 3'-CH_2, 5'-CH_2), 2.48 (4H, *t*, *J* = 5.1 Hz, 2'-CH_2, 6'-CH_2), 1.00 (9H, *s*, 3*-CH_3, 4*-CH_3, 5*-CH_3). **^{13}C NMR (101 MHz, CDCl$_3$, *δ ppm*)**: 169.0 (C-4"), 167.6 (C-10), 162.8 (C-4''', *dd*, $J_{(C-F)}$ = 249.2, 12.2 Hz), 161.4 (C-2"), 160.9 (C-2''', *dd*, $J_{(C-F)}$ = 250.4, 12.0 Hz), 158.2 (C-6"), 153.3 (C-2), 141.9 (C-4a), 140.3 (C-7a), 131.2 (C-6''', *dd*, $J_{(C-F)}$ = 9.8, 5.5 Hz), 124.1 (C-5), 123.9 (C-6), 122.0 (C-4), 120.0 (C-1''', *dd*, $J_{(C-F)}$ = 14.9, 3.8 Hz), 111.3 (C-5''', *dd*, $J_{(C-F)}$ = 21.1, 3.7 Hz), 110.8 (C-7), 103.9 (C-3''', *t*, $J_{(C-F)}$ = 25.4 Hz), 96.9 (C-5"), 60.3 (C-9, *d*, $J_{(C-F)}$ = 3.8 Hz), 56.5 (C-8), 54.9 (C-1*), 53.2 (C-2', C-6'), 52.0 (C-11), 43.7 (C-3', C-5'), 35.1 (C-2*), 28.9 (C-3*, C-4*, C-5*). **HRMS (ESI+)**: found *m/z* 565.2730 [M + H]$^+$; calculated $C_{30}H_{35}F_2N_6O_3^+$ 565.2660.

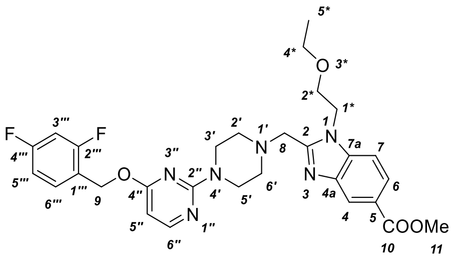

Methyl 2-((4-(4-((2,4-difluorobenzyl)oxy)pyrimidin-2-yl)piperazin-1-yl)methyl)-1-(2-ethoxyethyl)-1H-benzo[d]imidazole-5-carboxylate **23v**, white amorphous solid, 60 mg, 90%. **^{1}H NMR (400 MHz, CDCl$_3$, *δ ppm*)**: 8.38 (1H, *d*, *J* = 1.5 Hz, 4-CH), 7.99 (1H, *d*, *J* = 5.6 Hz, 6"-CH), 7.95 (1H, *dd*, *J* = 8.6, 1.6 Hz, 6-CH), 7.35-7.30 (2H, *m*, 6'''-CH, 7-CH), 6.81-6.70 (2H, *m*, 5'''-CH, 3'''-CH), 5.95 (1H, *d*, *J* = 5.6 Hz, 5"-CH), 5.27 (2H, *s*, 9-CH_2), 4.56 (2H, *t*, *J* = 5.4 Hz, 1*-CH_2), 3.89 (2H, *s*, 8-CH_2), 3.87 (3H, *s*, 11-CH_3), 3.75-3.70 (6H, *m*, 2*-CH_2, 3'-CH_2, 5'-CH_2), 3.39 (2H, *q*, *J* = 7.0 Hz, 4*-CH_2), 2.52-2.50 (4H, *m*, 2'-CH_2, 6'-CH_2), 1.06 (3H, *t*, *J* = 7.0 Hz, 5*-CH_3). **^{13}C NMR (101 MHz, CDCl$_3$, *δ ppm*)**: 169.0 (C-4"), 167.7 (C-10), 162.7 (C-4''', *dd*, $J_{(C-F)}$ = 249.3, 12.2 Hz), 161.5 (C-2"), 160.8 (C-2''', *dd*, $J_{(C-F)}$ = 250.2, 12.2 Hz), 158.4 (C-6"), 153.2 (C-2), 141.8 (C-4a), 140.1 (C-7a), 131.1 (C-6''', *dd*, $J_{(C-F)}$ = 9.7, 5.6 Hz), 124.0 (C-5), 123.8 (C-6), 122.1 (C-4), 120.1 (C-1''', *dd*, $J_{(C-F)}$ = 14.7, 3.7 Hz), 111.2 (C-5''', *dd*, $J_{(C-F)}$ = 21.0, 3.7 Hz), 110.9 (C-7), 103.6 (C-3''', *t*, $J_{(C-F)}$ = 25.2 Hz), 96.8

(C-5''), 69.3 (C-2*), 66.7 (C-4*), 60.4 (C-9, *d*, $J_{(C-F)}$ = 3.8 Hz), 56.6 (C-8), 53.2 (C-2', C-6'), 52.1 (C-11), 44.9 (C-1*), 43.4 (C-3', C-5'), 15.7 (C-5*). **HRMS** (**ESI+**): found *m/z* 567.2549 [M + H]$^+$; calculated $C_{29}H_{33}F_2N_6O_4^+$ 567.2453.

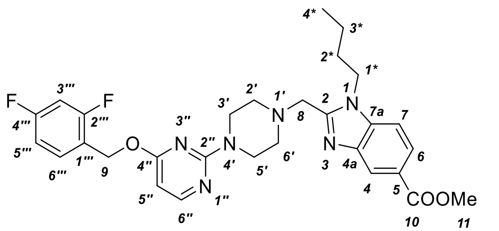

Methyl 1-butyl-2-((4-(4-((2,4-difluorobenzyl)oxy)pyrimidin-2-yl)piperazin-1-yl)methyl)-1H-benzo[d]imidazole-5-carboxylate **23w**, white amorphous solid, 90 mg, 95%. **^{1}H NMR** (**400 MHz, CDCl$_3$, *δ ppm***): 8.38 (1H, *d*, *J* = 1.5 Hz, 4-CH), 7.99 (1H, *d*, *J* = 5.6 Hz, 6''-CH), 7.95 (1H, *dd*, *J* = 8.5, 1.6 Hz, 6-CH), 7.36-7.30 (2H, *m*, 6'''-CH, 7-CH), 6.82-6.72 (2H, *m*, 5'''-CH, 3'''-CH), 5.95 (1H, *d*, *J* = 5.6 Hz, 5''-CH), 5.27 (2H, *s*, 9-CH$_2$), 4.28-4.24 (2H, *m*, 1*-CH$_2$), 3.88 (3H, *s*, 11-CH$_3$), 3.80 (2H, *s*, 8-CH$_2$), 3.73 (4H, *t*, *J* = 5.0 Hz, 3'-CH$_2$, 5'-CH$_2$), 2.53 (4H, *t*, *J* = 5.1 Hz, 2'-CH$_2$, 6'-CH$_2$), 1.81 (2H, *ddt*, *J* = 9.2, 7.6, 3.6 Hz, 2*-CH$_2$), 1.39 (2H, *h*, *J* = 7.4 Hz, 3*-CH$_2$), 0.93 (3H, *t*, *J* = 7.4 Hz, 4*-CH$_3$). **^{13}C NMR** (**101 MHz, CDCl$_3$, *δ ppm***): 169.0 (C-4''), 167.6 (C-10), 162.8 (C-4''', *dd*, $J_{(C-F)}$ = 249.3, 11.7 Hz), 161.5 (C-2''), 160.9 (C-2''', *dd*, $J_{(C-F)}$ = 250.4, 12.0 Hz), 158.2 (C-6''), 152.5 (C-2), 142.0 (C-4a), 139.0 (C-7a), 131.2 (C-6''', *dd*, $J_{(C-F)}$ = 9.8, 5.5 Hz), 124.2 (C-6), 124.2 (C-5), 122.2 (C-4), 120.0 (C-1''', *dd*, $J_{(C-F)}$ = 14.6, 3.7 Hz), 111.3 (C-5''', *dd*, $J_{(C-F)}$ = 21.3, 3.7 Hz), 109.3 (C-7), 103.9 (C-3''', *t*, $J_{(C-F)}$ = 25.2 Hz), 96.9 (C-5''), 60.4 (C-9, *d*, $J_{(C-F)}$ = 3.9 Hz), 55.9 (C-8), 53.2 (C-2', C-6'), 52.1 (C-11), 44.3 (C-1*), 43.7 (C-3', C-5'), 31.9 (C-2*), 20.4 (C-3*), 13.8 (C-4*). **HRMS** (**ESI+**): found *m/z* 551.2585 [M + H]$^+$; calculated $C_{29}H_{33}F_2N_6O_3^+$ 551.2504.

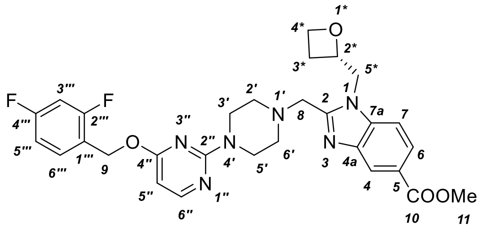

Methyl (S)-2-((4-(4-((2,4-difluorobenzyl)oxy)pyrimidin-2-yl)piperazin-1-yl)methyl)-1-(oxetan-2-ylmethyl)-1H-benzo[d]imidazole-5-carboxylate **23x**, white amorphous solid, 80 mg, 90%. **^{1}H NMR** (**400 MHz, CDCl$_3$, *δ ppm***): 8.39 (1H, *dd*, *J* = 1.6, 0.6 Hz, 4-CH), 7.99 (1H, *d*, *J* = 5.6 Hz, 6''-CH), 7.95 (1H, *dd*, *J* = 8.6, 1.6 Hz, 6-CH), 7.40 (1H, *dd*, *J* = 8.6, 0.6 Hz, 7-CH), 7.33 (1H, *td*, *J* = 8.4, 6.3 Hz, 6'''-CH), 6.82-6.72 (2H, *m*, 5'''-CH, 3'''-CH), 5.95 (1H, *d*, *J* = 5.6 Hz, 5''-CH), 5.27 (2H, *s*, 9-CH$_2$), 5.17 (1H, *tt*, *J* = 7.3, 4.4 Hz, 2*-CH), 4.61 (2H, *d*, *J* = 4.5 Hz, 5*-CH$_2$), 4.56 (1H, *ddd*, *J* = 8.3, 7.5, 5.9 Hz, 4*-CH$_2$), 4.29 (1H, *dt*, *J* = 9.2, 5.9 Hz, 4*-CH$_2$), 3.95-3.86 (2H, *m*, 8-CH$_2$), 3.88 (3H, *s*, 11-CH$_3$), 3.74-3.72 (4H, *m*, 3'-CH$_2$, 5'-CH$_2$), 2.66 (1H, *dtd*, *J* = 11.3, 8.0, 5.9 Hz, 3*-CH$_2$), 2.54-2.51 (4H, *m*, 2'-CH$_2$, 6'-CH$_2$), 2.38 (1H, *ddt*, *J* = 11.3, 9.2, 7.3 Hz, 3*-CH$_2$). **^{13}C NMR** (**101 MHz, CDCl$_3$, *δ ppm***): 169.0 (C-4''), 167.6 (C-10), 162.8 (C-4''', *dd*, $J_{(C-F)}$ = 249.4, 11.9 Hz), 161.5 (C-2''), 160.9 (C-2''', *dd*, $J_{(C-F)}$ = 250.2, 11.9 Hz), 158.2 (C-6''), 153.3 (C-2), 141.9 (C-4a), 139.6 (C-7a), 131.2 (C-6''', *dd*, $J_{(C-F)}$ = 9.8, 5.5 Hz), 124.5 (C-6), 124.4 (C-5), 122.1 (C-4), 120.0 (C-1''', *dd*, $J_{(C-F)}$ = 14.6, 3.6 Hz), 111.3 (C-5''', *dd*, $J_{(C-F)}$ = 21.1, 3.7 Hz), 109.7 (C-7), 103.9 (C-3''', *t*, $J_{(C-F)}$ = 25.4 Hz), 96.9 (C-5''), 81.1 (C-2*), 68.3 (C-4*), 60.4 (C-9, *d*, $J_{(C-F)}$ = 3.7 Hz), 55.9 (C-8), 53.2 (C-2', C-6'), 52.1 (C-11), 49.4 (C-5*), 43.7 (C-3', C-5'), 24.8 (C-3*). **HRMS** (**ESI+**): found *m/z* 565.2368 [M + H]$^+$; calculated $C_{29}H_{31}F_2N_6O_4^+$ 565.2297.

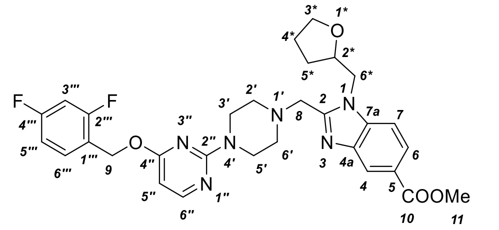

Methyl 2-((4-(4-((2,4-difluorobenzyl)oxy)pyrimidin-2-yl)piperazin-1-yl)methyl)-1-((tetrahydrofuran-2-yl)methyl)-1H-benzo[d]imidazole-5-carboxylate **23y**, white amorphous solid, 75 mg, 95%. **^{1}H NMR** (**400 MHz, CDCl$_3$, *δ ppm***): 8.37 (1H, *dd*, *J* = 1.6, 0.6 Hz, 4-CH), 7.98 (1H, *d*, *J* = 5.6 Hz, 6''-CH), 7.94 (1H, *dd*, *J* = 8.6, 1.6 Hz, 6-CH), 7.39 (1H, *dd*, *J* = 8.6, 0.6 Hz, 7-CH), 7.31 (1H, *td*, *J* = 8.4, 6.3 Hz, 6'''-CH), 6.80-6.69 (2H, *m*, 5'''-CH, 3'''-CH), 5.92 (1H, *d*, *J* = 5.6 Hz, 5''-CH), 5.38 (2H, *s*, 9-CH$_2$), 4.45-4.38 (2H, *m*, 6*-CH$_2$), 4.24-4.17 (1H, *m*, 2*-CH), 3.92-3.81 (2H, *m*, 8-CH$_2$), 3.88 (3H, *s*, 11-CH$_3$), 3.84-3.80 (1H, *m*, 5*-CH$_2$), 3.70-3.62 (5H, *m*, 3'-CH$_2$, 5'-CH$_2$, 5*-CH$_2$), 2.52-2.48 (4H, *m*, 2'-CH$_2$, 6'-CH$_2$), 2.03-1.94 (1H, *m*, 3*-CH$_2$), 1.88-1.78 (2H, *m*, 4*-CH$_2$), 1.61-1.54 (1H, *m*, 3*-CH$_2$). **^{13}C NMR** (**101 MHz, CDCl$_3$, *δ ppm***): 169.0 (C-4''), 167.5 (C-10), 162.7 (C-4''', *dd*, $J_{(C-F)}$ = 249.6, 11.8 Hz), 161.4 (C-2''), 160.5 (C-2''', *dd*, $J_{(C-F)}$ = 250.4, 11.7 Hz), 158.1 (C-6''), 153.1 (C-2), 141.8 (C-4a), 139.9 (C-7a), 131.3 (C-6''', *dd*, $J_{(C-F)}$ = 9.9, 5.7 Hz), 124.4 (C-6), 124.2 (C-5), 122.0 (C-4), 120.1 (C-1''', *dd*, $J_{(C-F)}$ = 14.7, 3.8 Hz), 111.1 (C-5''', *dd*, $J_{(C-F)}$ = 21.0, 3.8 Hz), 109.5 (C-7), 103.7 (C-3''', *t*, $J_{(C-F)}$ = 25.1 Hz), 96.9 (C-5''), 78.9 (C-2*), 69.0 (C-5*), 60.3 (C-9, *d*, $J_{(C-F)}$ = 4.8 Hz), 55.8 (C-8), 53.2 (C-2', C-6'), 52.2 (C-11), 48.4 (C-6*), 43.1 (C-3', C-5'), 29.1 (C-3*), 25.1 (C-4*).**HRMS** (**ESI+**): found *m/z* 579.2557 [M + H]$^+$; calculated $C_{30}H_{33}F_2N_6O_4^+$ 579.2453.

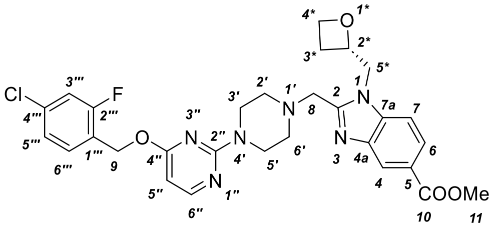

Methyl (S)-2-((4-(4-((4-chloro-2-fluorobenzyl)oxy)pyrimidin-2-yl)piperazin-1-yl)methyl)-1-(oxetan-2-ylmethyl)-1H-benzo[d]imidazole-5-carboxylate **23z**, white solid, 115 mg, 90%. **^{1}H NMR (400 MHz, CDCl$_3$, *δ ppm*)**: 8.39 (1H, *d*, *J* = 1.5 Hz, 4-CH), 8.00 (1H, *d*, *J* = 5.6 Hz, 6''-CH), 7.95 (1H, *dd*, *J* = 8.6, 1.6 Hz, 6-CH), 7.40 (1H, *d*, *J* = 8.6 Hz, 7-CH), 7.29 (1H, *t*, *J* = 8.0 Hz, 6'''-CH), 7.09-6.99 (2H, *m*, 3'''-CH, 5'''-CH), 5.96 (1H, *d*, *J* = 5.6 Hz, 5''-CH), 5.28 (2H, *s*, 9-CH$_2$), 5.16 (1H, *td*, *J* = 7.4, 3.8 Hz, 2*-CH), 4.61 (2H, *d*, *J* = 4.5 Hz, 5*-CH$_2$), 4.56 (1H, *td*, *J* = 8.0, 6.0 Hz, 4*-CH$_2$), 4.29 (1H, *dt*, *J* = 9.2, 5.9 Hz, 4*-CH$_2$), 3.97-3.83 (2H, *m*, 8-CH$_2$), 3.88 (3H, *s*, 11-CH$_3$), 3.72 (4H, *t*, *J* = 4.9 Hz, 3'-CH$_2$, 5'-CH$_2$), 2.64 (1H, *d*, *J* = 5.7 Hz, 3*-CH$_2$), 2.52 (4H, *t*, *J* = 5.1 Hz, 2'-CH$_2$, 6'-CH$_2$), 2.43-2.32 (1H, *m*, 3*-CH$_2$). **^{13}C NMR (101 MHz, CDCl$_3$, *δ ppm*)**: 168.9 (C-4''), 167.6 (C-10), 161.4 (C-2''), 160.4 (C-2''', *d*, $J_{(C-F)}$ = 251.3 Hz), 158.3 (C-6''), 153.2 (C-2), 141.9 (C-4a), 139.6 (C-7a), 134.7 (C-4''', *d*, $J_{(C-F)}$ = 10.2 Hz), 130.9 (C-6''', *d*, $J_{(C-F)}$ = 4.9 Hz), 124.6 (C-5''', *d*, $J_{(C-F)}$ = 3.8 Hz), 124.5 (C-6), 124.4 (C-5), 122.8 (C-1''', *d*, $J_{(C-F)}$ = 14.5 Hz), 122.1 (C-4), 116.2 (C-3''', *d*, $J_{(C-F)}$ = 24.8 Hz), 109.7 (C-7), 96.9 (C-5''), 81.1 (C-2*), 68.3 (C-4*), 60.3 (C-9, *d*, $J_{(C-F)}$ = 4.6 Hz), 55.9 (C-8), 53.1 (C-2', C-6'), 52.1 (C-11), 49.4 (C-5*), 43.7 (C-3', C-5'), 24.8 (C-3*). **HRMS (ESI+)**: found *m/z* 581.2049 [M + H]$^+$; calculated $C_{29}H_{31}{}^{35}ClFN_6O_4{}^+$ 581.2001.

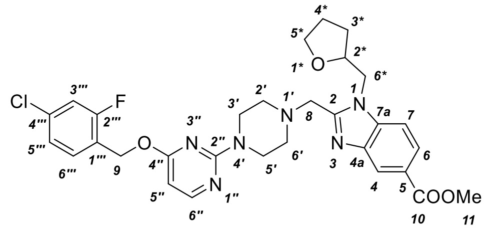

Methyl 2-((4-(4-((4-chloro-2-fluorobenzyl)oxy)pyrimidin-2-yl)piperazin-1-yl)methyl)-1-((tetrahydrofuran-2-yl)methyl)-1H-benzo[d]imidazole-5-carboxylate **23aa**, white solid, 105 mg, 95%. **^{1}H NMR (400 MHz, CDCl$_3$, *δ ppm*)**: 8.37 (1H, *d*, *J* = 1.4 Hz, 4-CH), 7.99 (1H, *d*, *J* = 5.6 Hz, 6''-CH), 7.98-7.90 (1H, *m*, 6-CH), 7.39 (1H, *d*, *J* = 8.7 Hz, 7-CH), 7.29 (1H, *t*, *J* = 8.0 Hz, 6'''-CH), 7.14-6.99 (2H, *m*, *J* = 9.3, 1.5 Hz, 3'''-CH, 5'''-CH), 5.95 (1H, *d*, *J* = 5.6 Hz, 5''-CH), 5.28 (2H, *s*, 9-CH$_2$), 4.49-4.37 (2H, *m*, 6*-CH$_2$), 4.23 (1H, *qd*, *J* = 6.7, 3.8 Hz, 2*-CH), 3.96-3.82 (2H, *m*, 8-CH$_2$), 3.87 (3H, *s*, 12-CH$_3$), 3.83-3.76 (1H, *m*, 5*-CH$_2$), 3.70 (5H, *m*, 3'-CH$_2$, 5'-CH$_2$, 5*-CH$_2$), 2.52 (4H, *t*, *J* = 5.0 Hz,2'-CH$_2$, 6'-CH$_2$), 2.04-1.93 (1H, *m*, 3*-CH$_2$), 1.89-1.76 (2H, *m*, 4*-CH$_2$), 1.57 (1H, *dq*, *J* = 12.1, 8.0 Hz, 3*-CH$_2$). **^{13}C NMR (101 MHz, CDCl$_3$, *δ ppm*)**: 168.9 (C-4''), 167.6 (C-11), 161.4 (C-2''), 160.4 (C-2''', *d*, $J_{(C-F)}$ = 251.3 Hz), 158.3 (C-6''), 153.2 (C-2), 141.9 (C-4a), 139.5 (C-7a), 134.7 (C-4''', *d*, $J_{(C-F)}$ = 10.2 Hz), 130.9 (C-6''', *d*, $J_{(C-F)}$ = 5.0 Hz), 124.6 (C-5''', *d*, $J_{(C-F)}$ = 4.5 Hz), 124.3 (C-6), 124.2 (C-5), 122.8 (C-1''', *d*, $J_{(C-F)}$ = 14.6 Hz), 122.1 (C-4), 116.2 (C-3''', *d*, $J_{(C-F)}$ = 24.7 Hz), 109.8 (C-7), 96.9 (C-5''), 78.3 (C-2*), 68.2 (C-5*), 60.3 (C-9, *d*, $J_{(C-F)}$ = 3.8 Hz), 55.9 (C-8), 53.2 (C-2', C-6'), 52.1 (C-12), 48.1 (C-6*), 43.7 (C-3', C-5'), 29.3 (C-3*), 25.7 (C-4*). **HRMS (ESI+)**: found *m/z* 595.2204 [M + H]$^+$; calculated $C_{30}H_{33}{}^{35}ClFN_6O_4{}^+$ 595.2158.

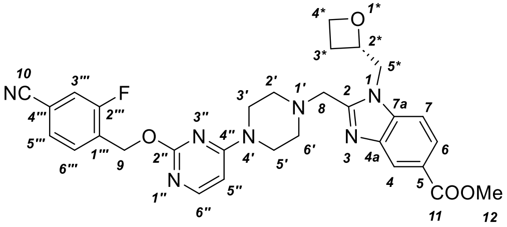

Methyl (S)-2-((4-(2-((4-cyano-2-fluorobenzyl)oxy)pyrimidin-4-yl)piperazin-1-yl)methyl)-1-(oxetan-2-ylmethyl)-1H-benzo[d]imidazole-5-carboxylate **23r'**, white solid, 50 mg, 90%. **^{1}H NMR (400 MHz, CDCl$_3$, *δ ppm*)**: 8.39 (1H, *d*, *J* = 1.5 Hz, 4-CH), 7.99-7.91 (2H, *d*, 6-CH, 6''-CH), 7.59 (1H, *t*, *J* = 7.5, 1.6 Hz, 6'''-CH), 7.42-7.33 (2H, *m*, 5'''-CH, 7-CH) 7.28 (1H, *dd*, *J* = 9.3, 1.5 Hz, 3'''-CH), 6.13 (1H, *d*, *J* = 5.6 Hz, 5''-CH), 5.40 (2H, *s*, 9-CH$_2$), 5.21-5.10 (1H, *m*, 2*-CH), 4.57 (2H, *d J* = 4.7 Hz, 5*-CH$_2$), 4.54 (1H, *dd*, *J* = 7.8, 5.8 Hz, 4*-CH$_2$), 4.27 (1H, *dt*, *J* = 9.2, 5.9 Hz, 4*-CH$_2$), 4.04-3.83 (2H, *m*, 8-CH$_2$), 3.88 (3H, *s*, 12-CH$_3$), 3.56 (4H, *br.s.*, 3'-CH$_2$, 5'-CH$_2$), 2.74-2.59 (1H, *m*, 3*-CH$_2$), 2.55 (4H, *t*, *J* = 5.1 Hz, 2'-CH$_2$, 6'-CH$_2$), 2.376 (1H, *ddt*, *J* = 11.3, 9.1, 7.3 Hz, 3*-CH$_2$). **^{13}C NMR (101 MHz, CDCl$_3$, *δ ppm*)**: 167.5 (C-11), 164.1 (C-2''), 163.4 (C-4''), 159.6 (C-2''', *d*, $J_{(C-F)}$ = 250.5 Hz), 157.4 (C-6''), 152.9 (C-2), 141.8 (C-4a), 139.5 (C-7a), 130.8 (C-1''', *d*, $J_{(C-F)}$ = 14.4 Hz), 130.4 (C-6''', *d*, $J_{(C-F)}$ = 4.6 Hz), 128.2 (C-5''', *d*, $J_{(C-F)}$ = 3.8 Hz), 124.6 (C-6), 124.6 (C-5), 122.2 (C-4), 118.8 (C-3''', *d*, $J_{(C-F)}$ = 24.8 Hz), 117.5 (C-10, *d*, $J_{(C-F)}$ = 2.9 Hz), 112.8 (C-4''', *d*, $J_{(C-F)}$ = 9.5 Hz), 109.7 (C-7), 96.7 (C-5''), 80.9 (C-2*), 68.3 (C-4*), 61.3 (C-9, *d*, $J_{(C-F)}$ = 4.8 Hz), 55.5 (C-8), 52.8 (C-2', C-6'), 52.1 (C-12), 49.3 (C-5*), 43.9 (C-3', C-5'), 24.7 (C-3*). **HRMS (ESI+)**: found *m/z* 572.2392 [M + H]$^+$; calculated $C_{30}H_{31}FN_7O_4{}^+$ 572.2343.

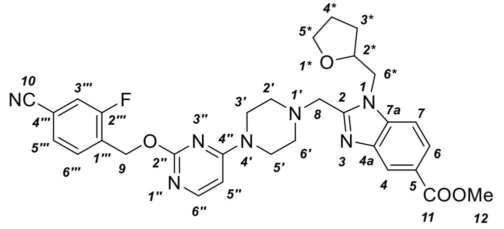

Methyl 2-((4-(2-((4-cyano-2-fluorobenzyl)oxy)pyrimidin-4-yl)piperazin-1-yl)methyl)-1-((tetrahydrofuran-2-yl)methyl)-1H-benzo[d]imidazole-5-carboxylate **23s'**, white solid, 95 mg, 90%. **^{1}H NMR (400 MHz, CDCl$_3$, *δ ppm*)**: 8.37 (1H, *d*, *J* = 1.4 Hz, 4-CH), 7.98-7.92 (2H, *m*, 6-CH, 6''-CH), 7.60 (1H, *t*, *J* = 7.5 Hz, 6'''-CH), 7.40-7.36 (2H, *m*, 5'''-CH, 7-CH), 7.28 (1H, *dd*, *J* = 9.3, 1.5 Hz, 3'''-CH), 6.11 (1H, *d*, *J* = 6.1 Hz, 5''-CH), 5.39 (2H, *s*, 9-CH$_2$), 4.41 (2H, *qd*, *J* = 14.9, 5.1 Hz, 6*-CH$_2$), 4.24-4.18 (1H, *m*, 2*-CH), 3.93-3.81 (2H, *m*, 8-CH$_2$), 3.88 (3H, *s*, 12-CH$_3$), 3.78 (1H, *dt*, *J* = 8.4, 6.9 Hz, 5*-CH$_2$), 3.72-3.63 (1H, *m*, 5*-CH$_2$), 3.59-3.48 (4H, *m*, 3'-CH$_2$, 5'-CH$_2$), 2.54 (4H, *t*, *J* = 5.1 Hz, 2'-CH$_2$, 6'-CH$_2$), 2.00 (1H, *dtd*, *J* = 12.2, 7.3, 5.5 Hz, 3*-CH$_2$), 1.90-1.73 (2H, *m*, 4*-CH$_2$), 1.55 (1H, *dq*, *J* = 12.2, 8.1 Hz, 3*-CH$_2$). **^{13}C NMR (101 MHz, CDCl$_3$, *δ ppm*)**: 167.6 (C-11), 164.2 (C-2''), 163.4 (C-4''), 159.6 (C-2''', *d*, $J_{(C-F)}$ = 250.7 Hz), 157.6 (C-6''), 152.8 (C-2), 141.8 (C-4a), 139.4 (C-7a), 130.8 (C-1''', *d*, $J_{(C-F)}$ = 14.5 Hz), 130.4 (C-6''', *d*, $J_{(C-F)}$ = 5.0 Hz), 128.2 (C-5''', *d*, $J_{(C-F)}$ = 3.7 Hz), 124.4 (C-6), 124.4 (C-5), 122.1 (C-4), 118.8 (C-3''', *d*, $J_{(C-F)}$ = 25.1 Hz), 117.6 (C-10, *d*, $J_{(C-F)}$ = 2.9 Hz), 112.7 (C-4''', *d*, $J_{(C-F)}$ = 9.5 Hz), 109.8 (C-7), 97.7 (C-5''), 78.2 (C-2*), 68.2 (C-5*), 61.3 (C-9, *d*, $J_{(C-F)}$ = 4.6 Hz), 55.6 (C-8), 52.8 (C-2', C-6'), 52.1 (C-12), 48.0 (C-6*), 43.9 (C-3', C-5'), 29.3 (C-3*), 25.7 (C-4*). **HRMS (ESI+)**: found *m/z* 586.2543 [M + H]$^+$; calculated $C_{31}H_{33}FN_7O_4^+$ 586.2500.

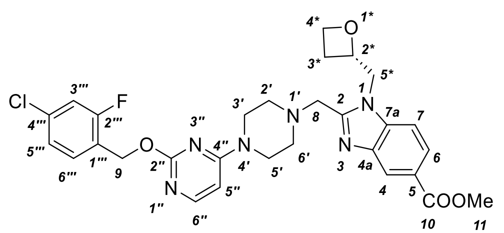

Methyl (S)-2-((4-(2-((4-chloro-2-fluorobenzyl)oxy)pyrimidin-4-yl)piperazin-1-yl)methyl)-1-(oxetan-2-ylmethyl)-1H-benzo[d]imidazole-5-carboxylate **23z'**, white solid, 105 mg, 85%. **^{1}H NMR (400 MHz, CDCl$_3$, *δ ppm*)**: 8.38 (1H, *d*, *J* = 1.4 Hz, 4-CH), 7.94 (1H, *dd*, *J* = 8.6, 1.6 Hz, 6-CH), 7.42 (1H, *t*, *J* = 8.0 Hz, 6'''-CH), 7.37 (1H, *d*, *J* = 8.5 Hz, 7-CH), 7.28 (1H, *dd*, *J* = 7.6, 1.3 Hz, 6''-CH), 7.08-6.97 (2H, *m*, 3'''-CH, 5'''-CH) 5.73 (1H, *d*, *J* = 7.6 Hz, 5''-CH), 5.14 (1H, *tt*, *J* = 7.5, 4.3 Hz, 2*-CH), 4.89 (2H, *s*, 9-CH$_2$), 4.54 (2H, *d*, *J* = 4.4 Hz, 5*-CH$_2$), 4.59-4.49 (1H, *m*, 4*-CH$_2$), 4.26 (1H, *dt*, *J* = 9.2, 5.9 Hz, 4*-CH$_2$), 3.99-3.81 (2H, *m*, 8-CH$_2$), 3.87 (3H, *s*, 11-CH$_3$), 3.56 (2H, *dd*, *J* = 11.0, 3.2 Hz, 3'-CH$_2$ or 5'-CH$_2$), 3.33 (2H, *dd*, *J* = 11.0, 7.8 Hz, 3'-CH$_2$ or 5'-CH$_2$), 2.65 (1H, *dtd*, *J* = 11.3, 8.1, 5.9 Hz, 3*-CH$_2$), 2.51 (4H, *t*, *J* = 5.1 Hz, 2'-CH$_2$, 6'-CH$_2$), 2.35 (1H, *ddt*, *J* = 11.4, 9.1, 7.3 Hz, 3*-CH$_2$). **^{13}C NMR (101 MHz, CDCl$_3$, *δ ppm*)**: 167.5 (C-10), 162.8 (C-4''), 160.7 (C-2''', *d*, $J_{(C-F)}$ = 251.3 Hz), 156.2 (C-2''), 152.9 (C-2), 145.3 (C-6'', *d*, $J_{(C-F)}$ = 2.9 Hz), 141.8 (C-4a), 139.5 (C-7a), 135.0 (C-4''', *d*, $J_{(C-F)}$ = 10.3 Hz), 132.6 (C-6''', *d*, $J_{(C-F)}$ = 4.8 Hz), 125.0 (C-5''', *d*, $J_{(C-F)}$ = 4.0 Hz), 124.6 (C-6), 124.5 (C-5), 122.2 (C-4), 122.0 (C-1''', *d*, $J_{(C-F)}$ = 15.0 Hz), 116.1 (C-3''', *d*, $J_{(C-F)}$ = 24.9 Hz), 109.7 (C-7), 91.5 (C-5''), 80.9 (C-2*), 68.3 (C-4*), 68.1 (C-3', C-5'), 55.4 (C-8), 52.9 (C-2', C-6'), 52.1 (C-11), 49.3 (C-5*), 46.0 (C-9, *d*, $J_{(C-F)}$ = 3.1 Hz), 24.7 (C-3*). **HRMS (ESI+)**: found *m/z* 581.2044 [M + H]$^+$; calculated $C_{29}H_{31}{}^{35}ClFN_6O_4^+$ 581.2001.

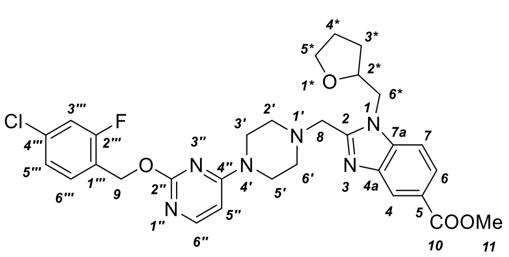

Methyl 2-((4-(2-((4-chloro-2-fluorobenzyl)oxy)pyrimidin-4-yl)piperazin-1-yl)methyl)-1-((tetrahydrofuran-2-yl)methyl)-1H-benzo[d]imidazole-5-carboxylate **23aa'**, white solid, 110 mg, 85%. **^{1}H NMR (400 MHz, CDCl$_3$, *δ ppm*)**: 8.37 (1H, *d*, *J* = 1.5 Hz, 4-CH), 7.96-7.93 (2H, *m*, 6-CH, 6''-CH), 7.44-7.33 (2H, *m*, 5-CH, 6'''-CH), 7.03 (2H, *ddd*, *J* = 14.0, 8.0, 2.1 Hz, 5'''-CH, 3'''-CH), 6.09 (1H, *d*, *J* = 6.1 Hz, 5''-CH), 5.30 (2H, *s*, 9-CH$_2$), 4.49-4.32 (2H, *m*, 6*-CH$_2$), 4.21 (1H, *qd*, *J* = 6.8, 3.5 Hz, 2*-CH), 3.96-3.80 (2H, *m*, 8-CH$_2$), 3.87 (3H, *s*, 11-CH$_3$), 3.78 (1H, *td*, *J* = 8.3, 6.9 Hz, 5*-CH$_2$), 3.67 (1H, *td*, *J* = 7.9, 6.1 Hz, 5*-CH$_2$), 3.59-3.52 (4H, *m*, 3'-CH$_2$, 5'-CH$_2$), 2.53 (4H, *t*, *J* = 5.1 Hz, 2'-CH$_2$, 6'-CH$_2$), 2.06-1.94 (1H, *m*, 3*-CH$_2$), 1.89-1.73 (2H, *m*, 4*-CH$_2$), 1.56 (1H, *dq*, *J* = 12.1, 8.1 Hz, 3*-CH$_2$). **^{13}C NMR (101 MHz, CDCl$_3$, *δ ppm*)**: 167.6 (C-10), 164.5 (C-2''), 163.4 (C-4''), 160.4 (C-2''', *d*, $J_{(C-F)}$ = 250.9 Hz), 157.6 (C-6''), 152.9 (C-2), 141.9 (C-4a), 139.4 (C-7a), 134.3 (C-4''', *d*, $J_{(C-F)}$ = 10.2 Hz), 130.9 (C-6''', *d*, $J_{(C-F)}$ = 5.0 Hz), 124.5 (C-5''', *d*, $J_{(C-F)}$ = 4.5 Hz), 124.4 (C-6), 124.3 (C-5), 123.2 (C-1''', *d*, $J_{(C-F)}$ = 14.5 Hz), 122.1 (C-4), 116.0 (C-3''', *d*, $J_{(C-F)}$ = 25.1 Hz), 109.8 (C-7), 97.5 (C-5''), 78.2 (C-2*), 68.2 (C-5*), 61.5 (C-9, *d*, $J_{(C-F)}$ = 4.2 Hz), 55.6 (C-8), 52.8 (C-2', C-6'), 52.1 (C-11), 48.0 (C-6*), 43.8 (C-3', C-5'), 29.3 (C-3*), 25.7 (C-4*). **HRMS (ESI+)**: found *m/z* 595.2197 [M + H]$^+$; calculated $C_{30}H_{33}{}^{35}ClFN_6O_4^+$ 595.2158.

^{1}H NMR spectrum of compound **23a**

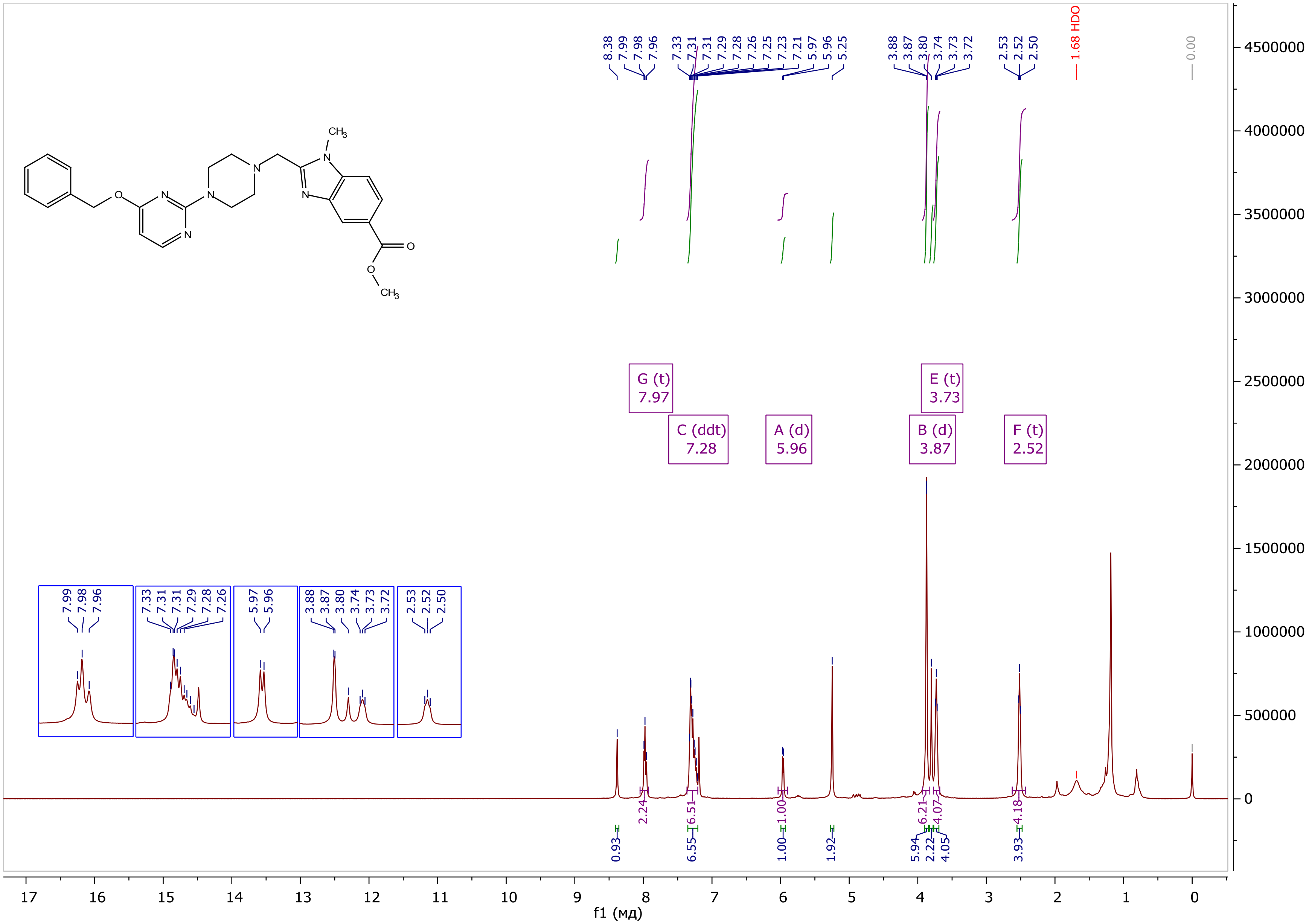

^{13}C NMR spectrum of compound **23a**

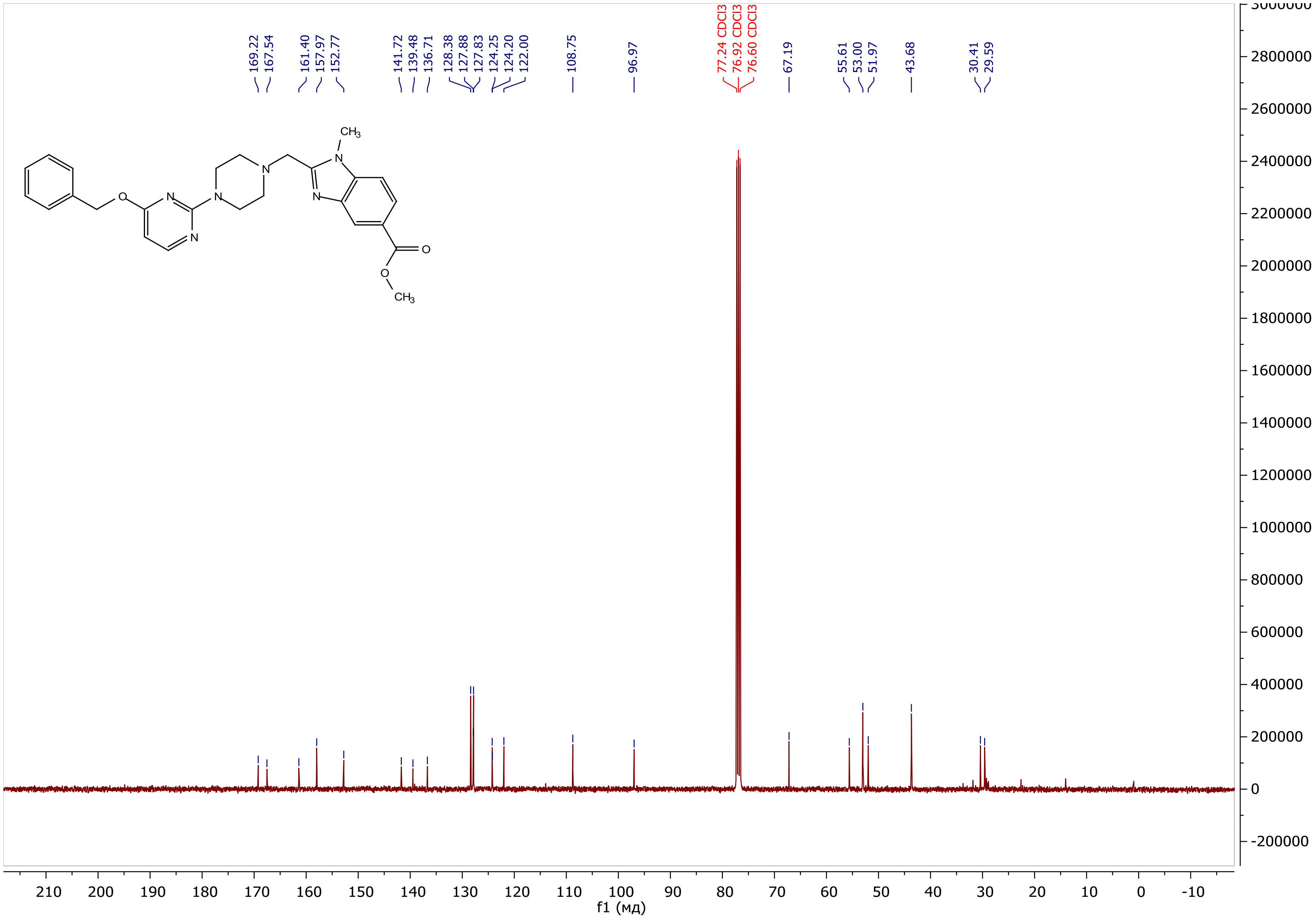

^{1}H NMR spectrum of compound **23b**

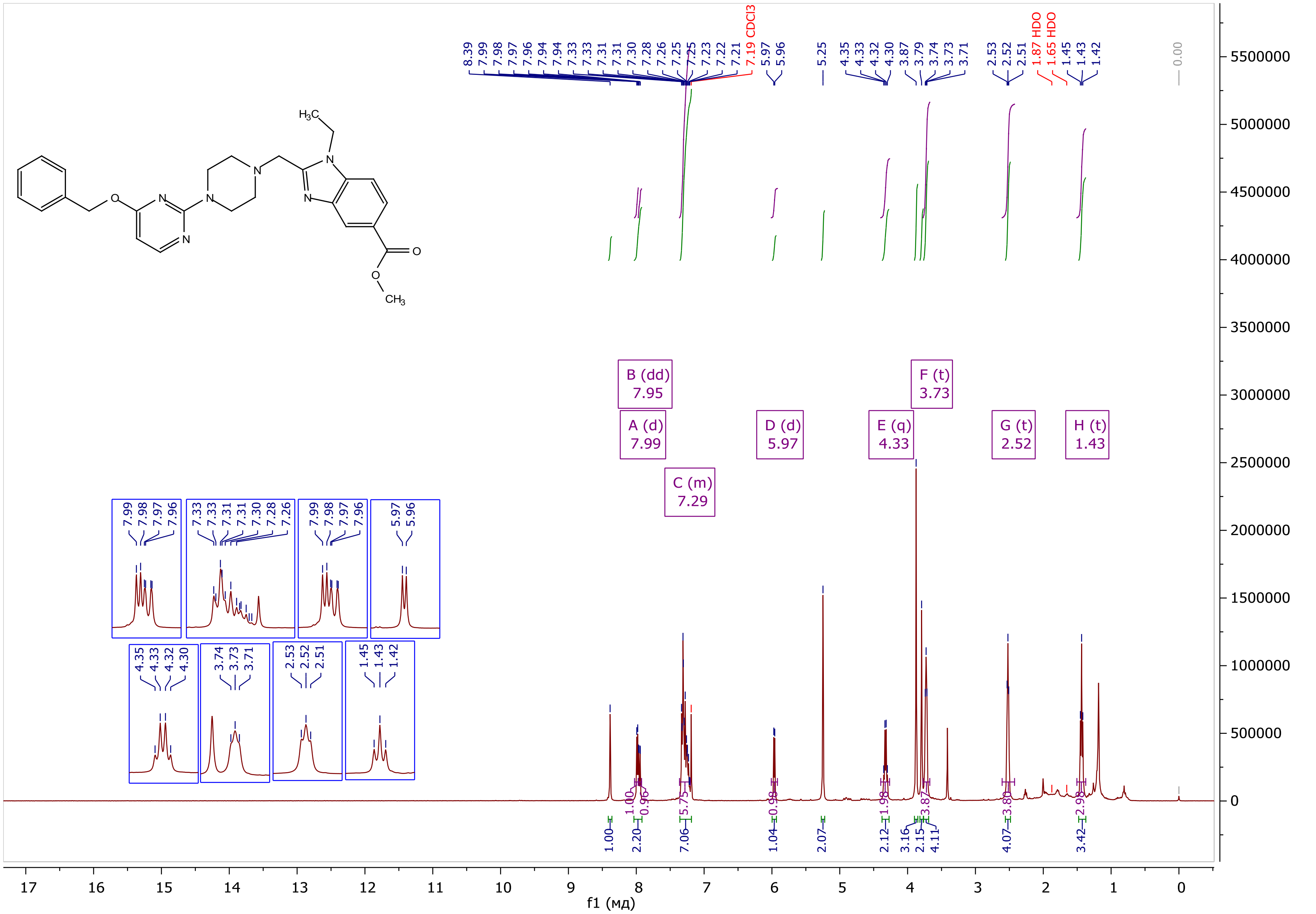

^{13}C NMR spectrum of compound **23b**

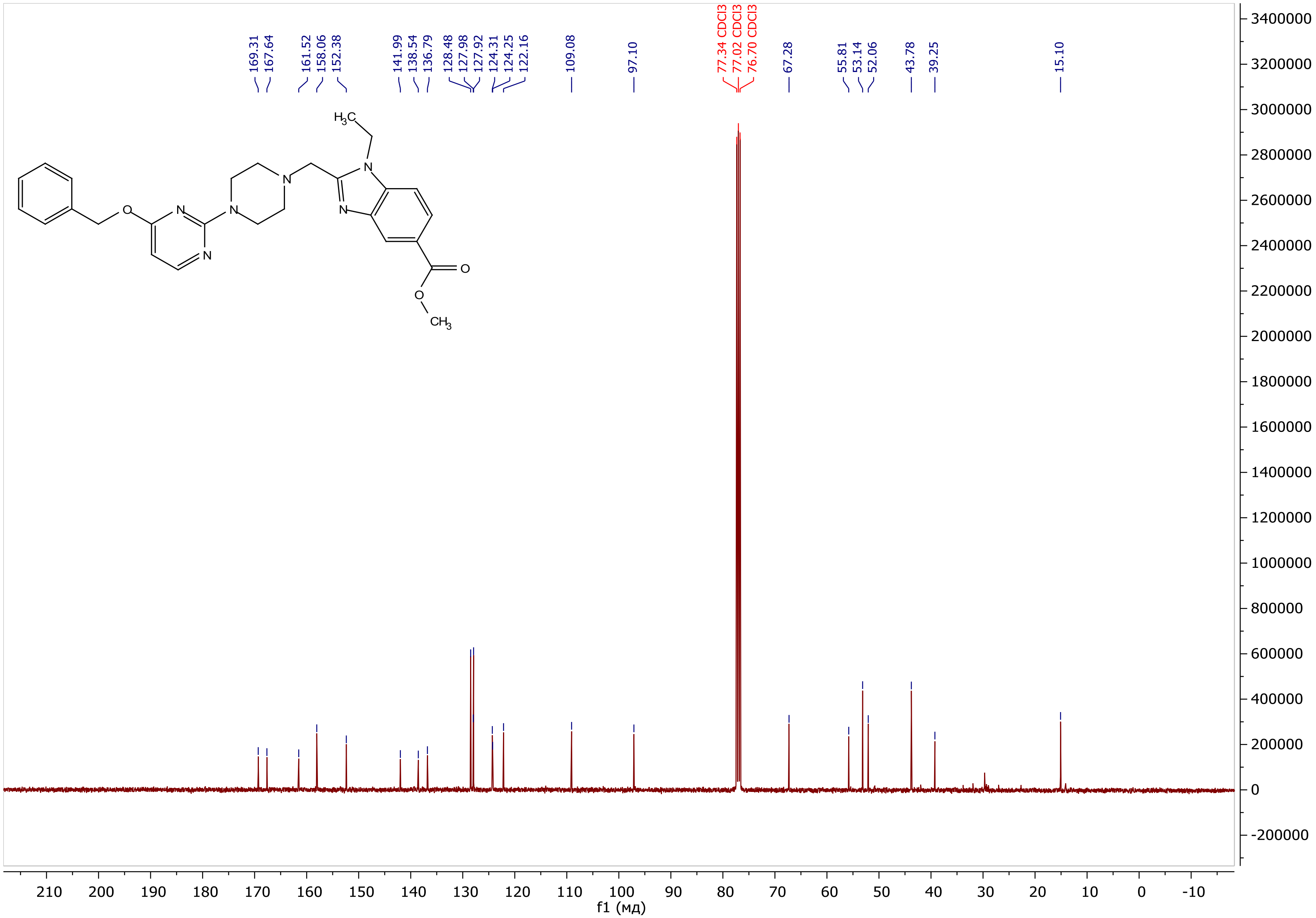

^{1}H NMR spectrum of compound **23c**

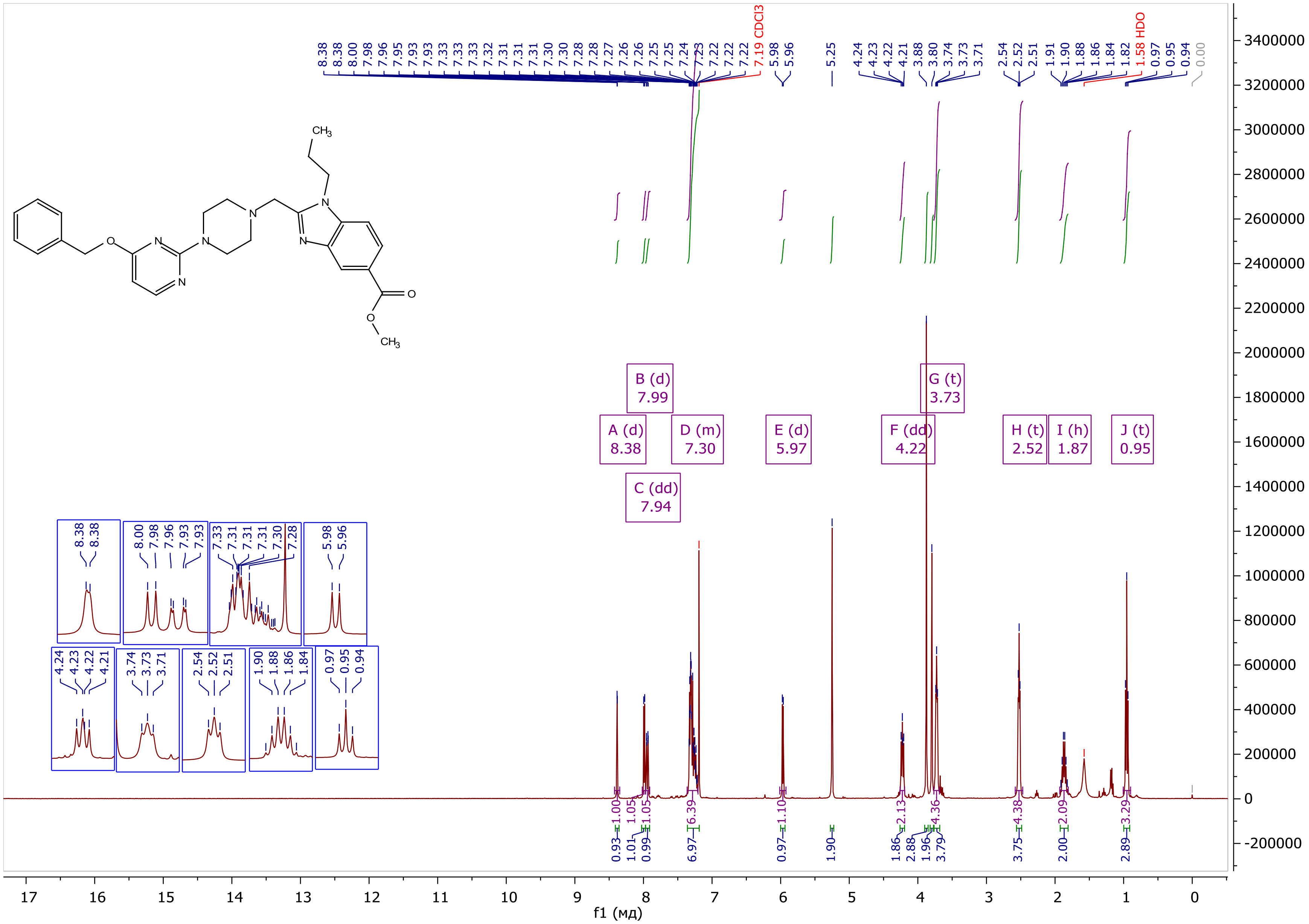

^{13}C NMR spectrum of compound **23c**

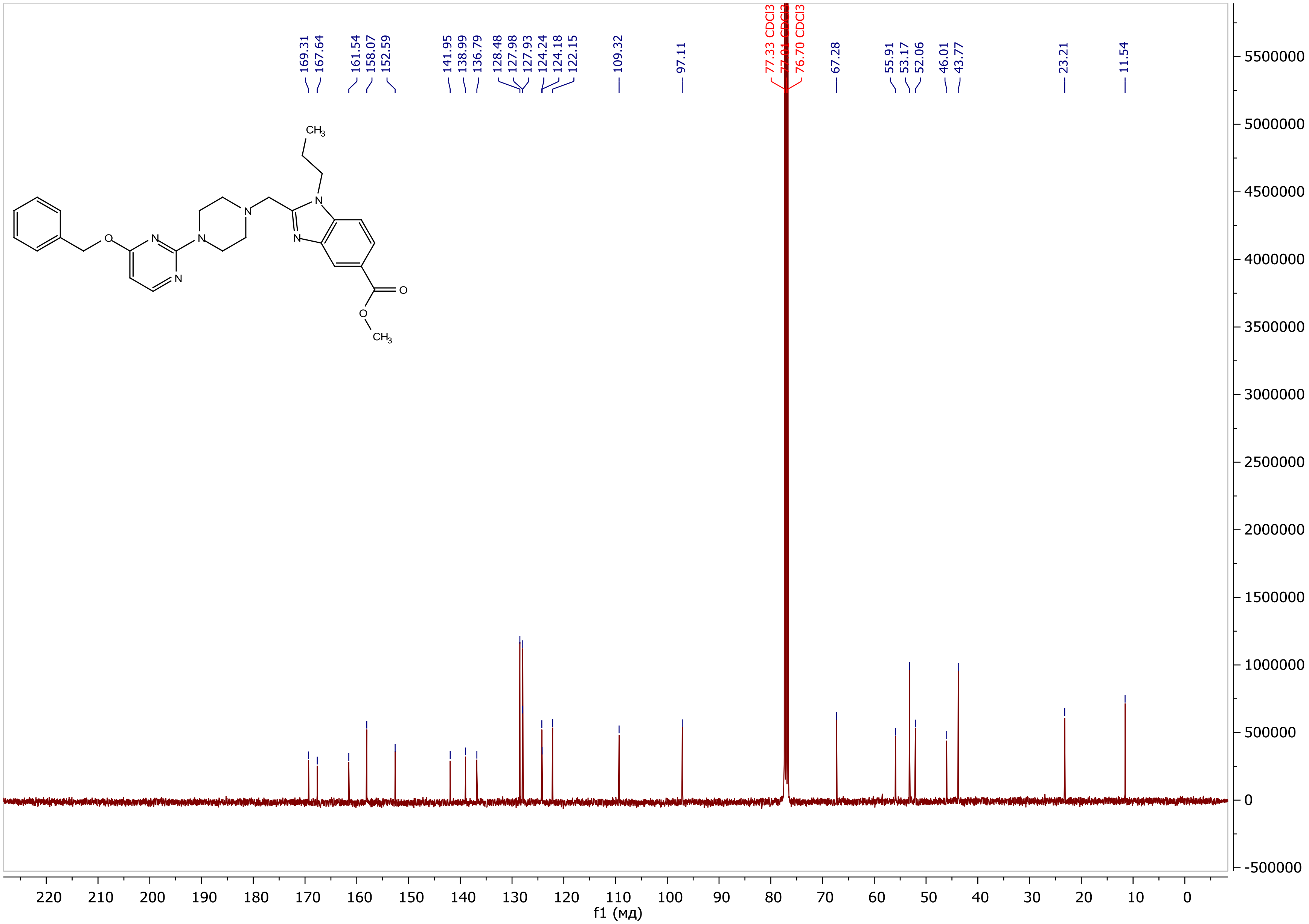

^{1}H NMR spectrum of compound **23d**

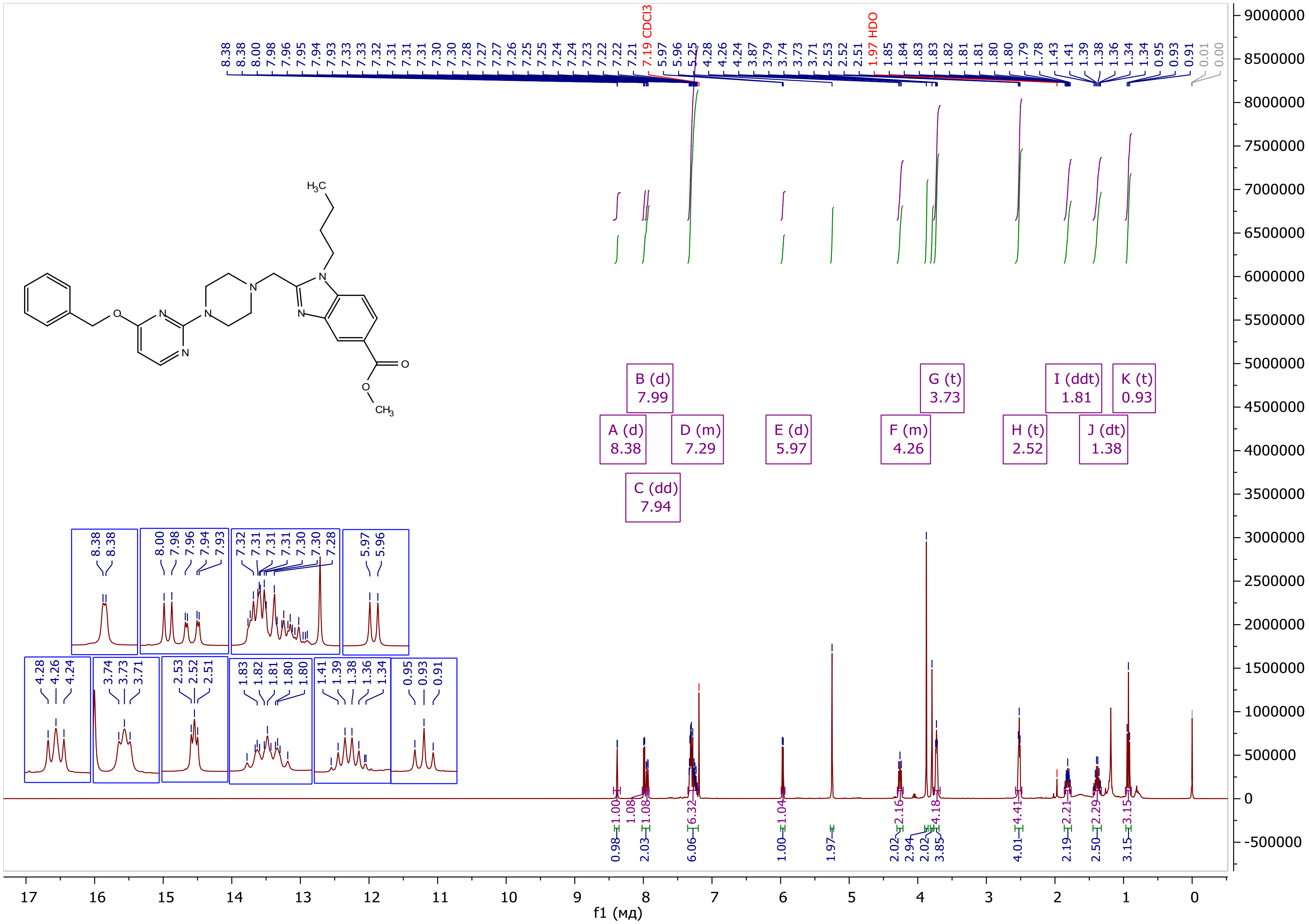

^{13}C NMR spectrum of compound **23d**

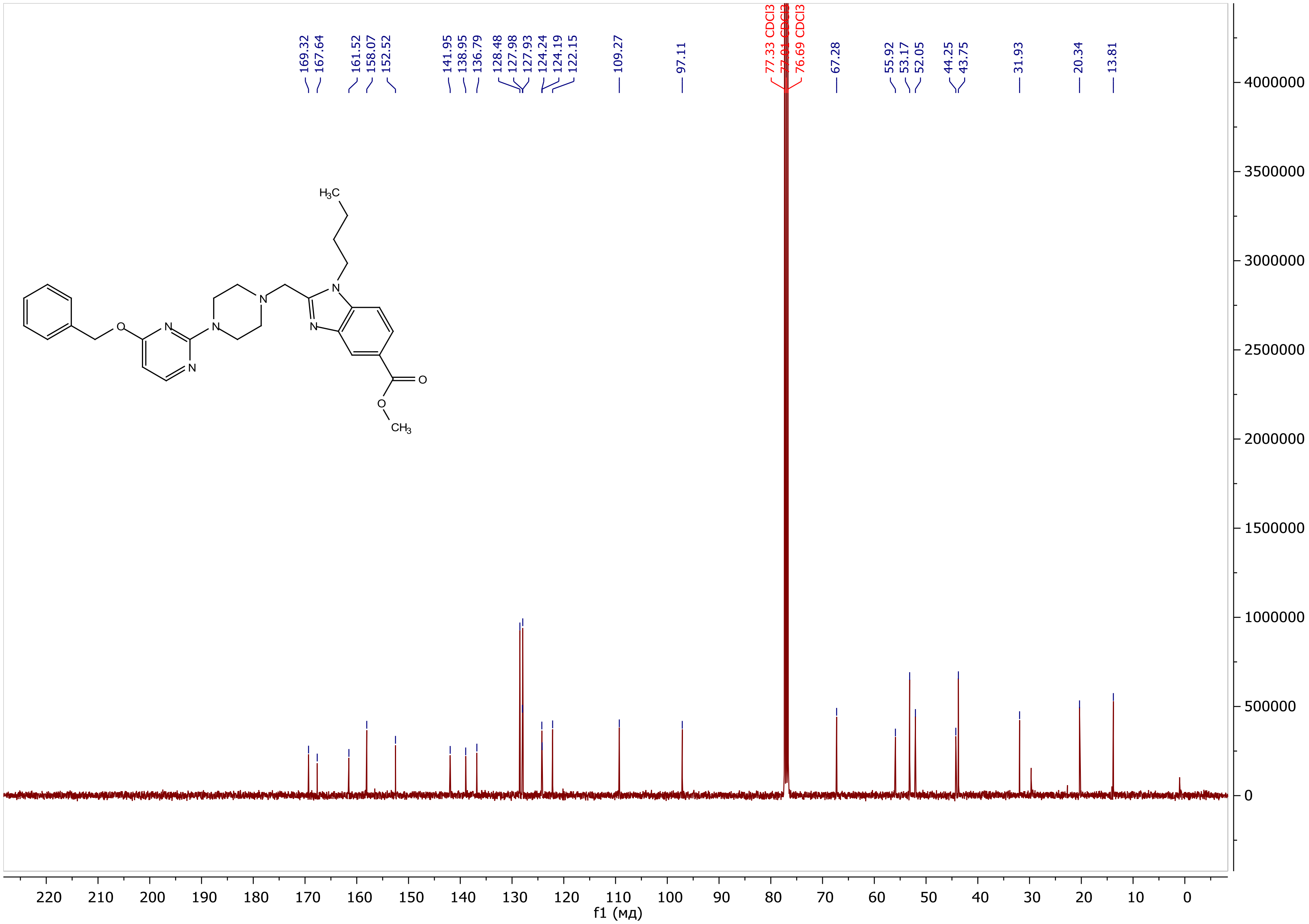

^{1}H NMR spectrum of compound **23e**

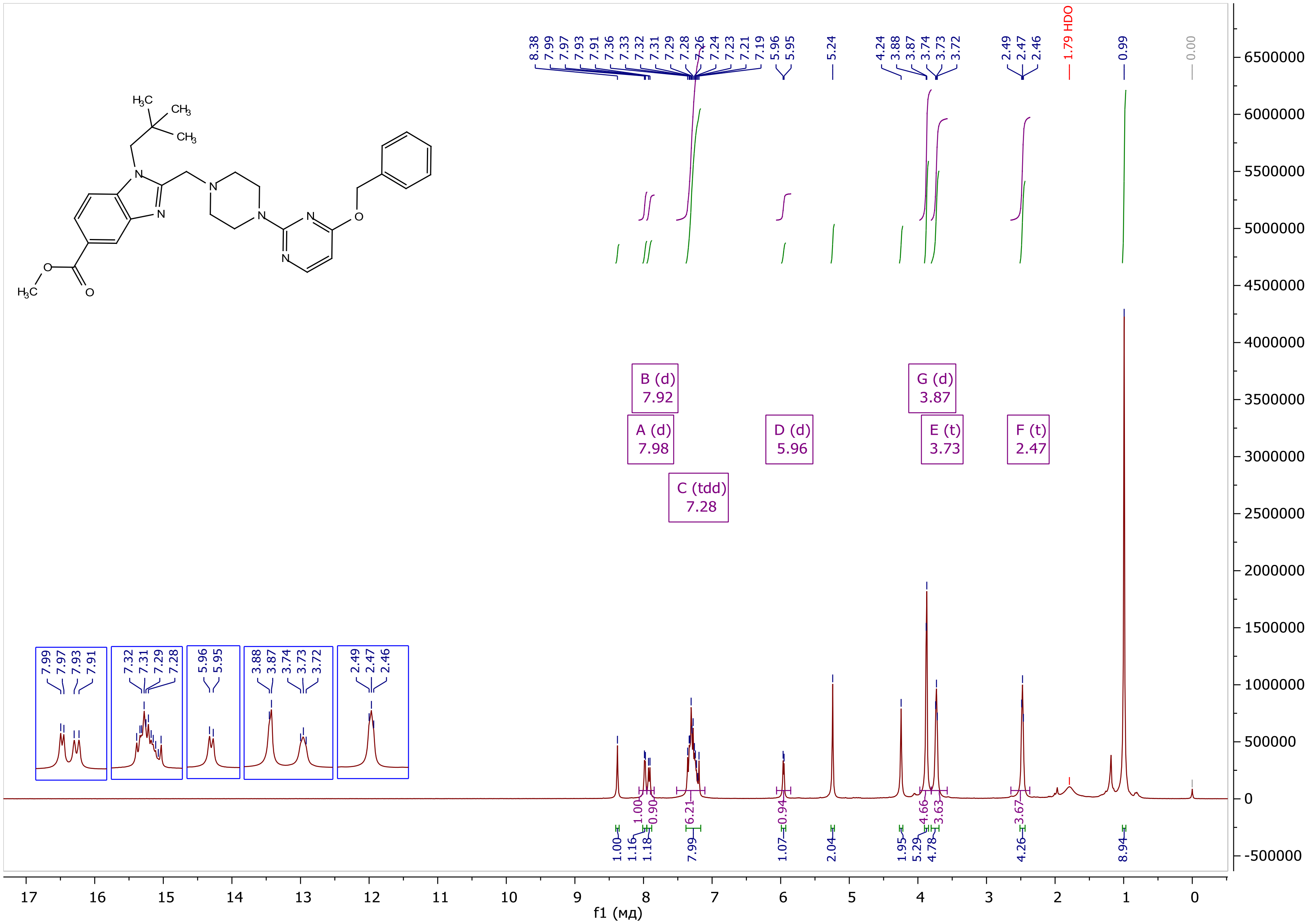

^{13}C NMR spectrum of compound **23e**

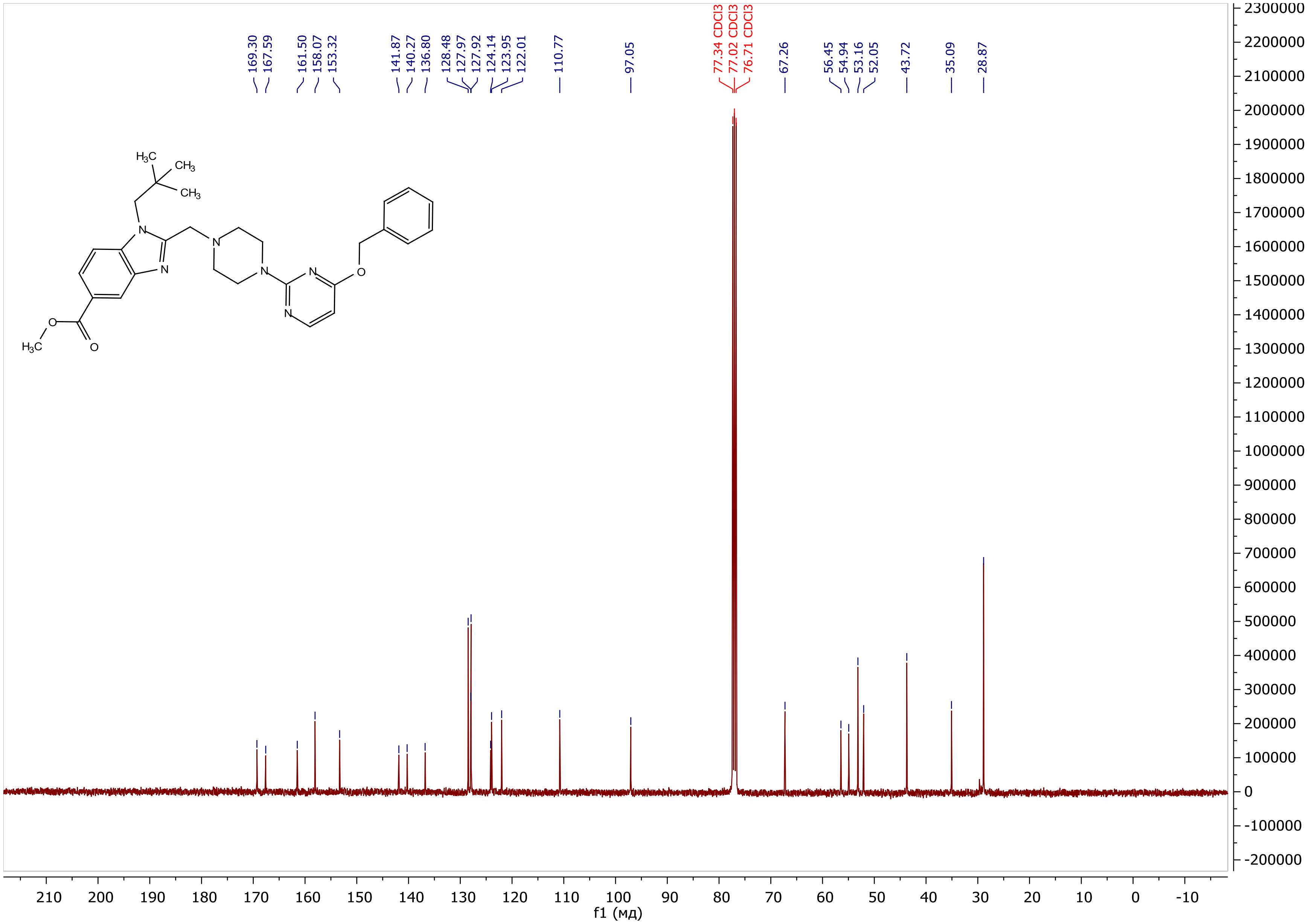

^{1}H NMR spectrum of compound **23f**

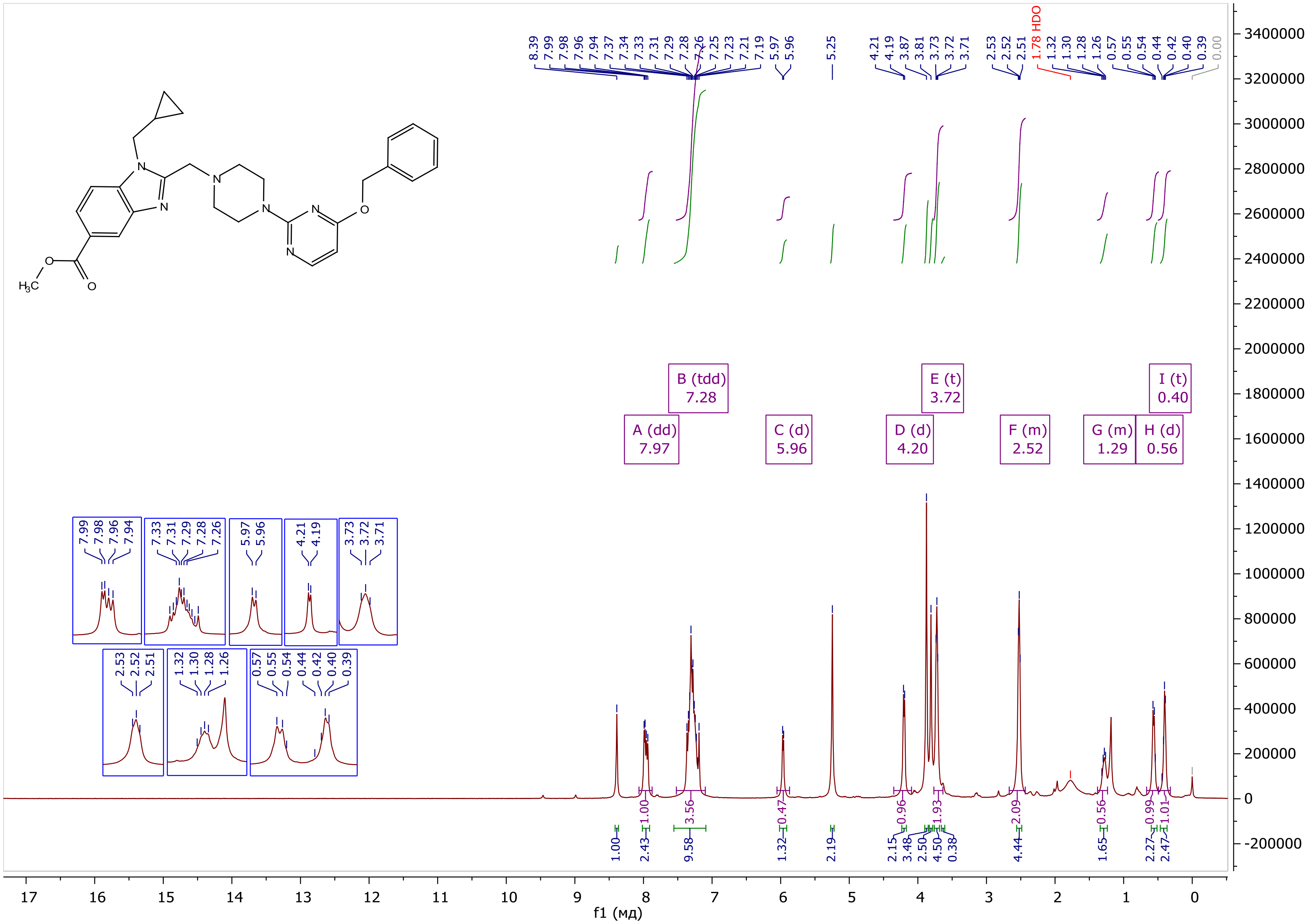

^{13}C NMR spectrum of compound **23f**

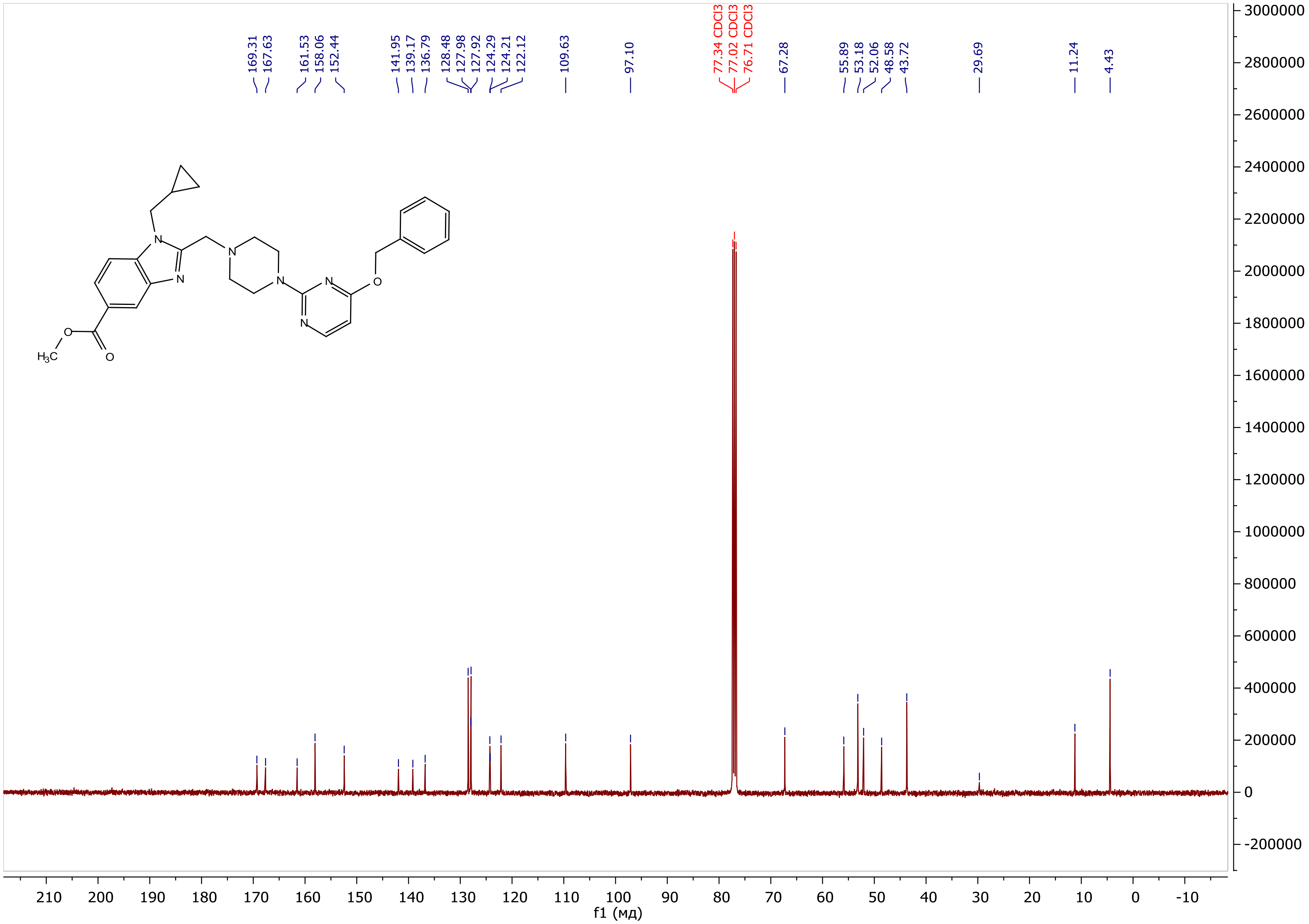

^{1}H NMR spectrum of compound **23g**

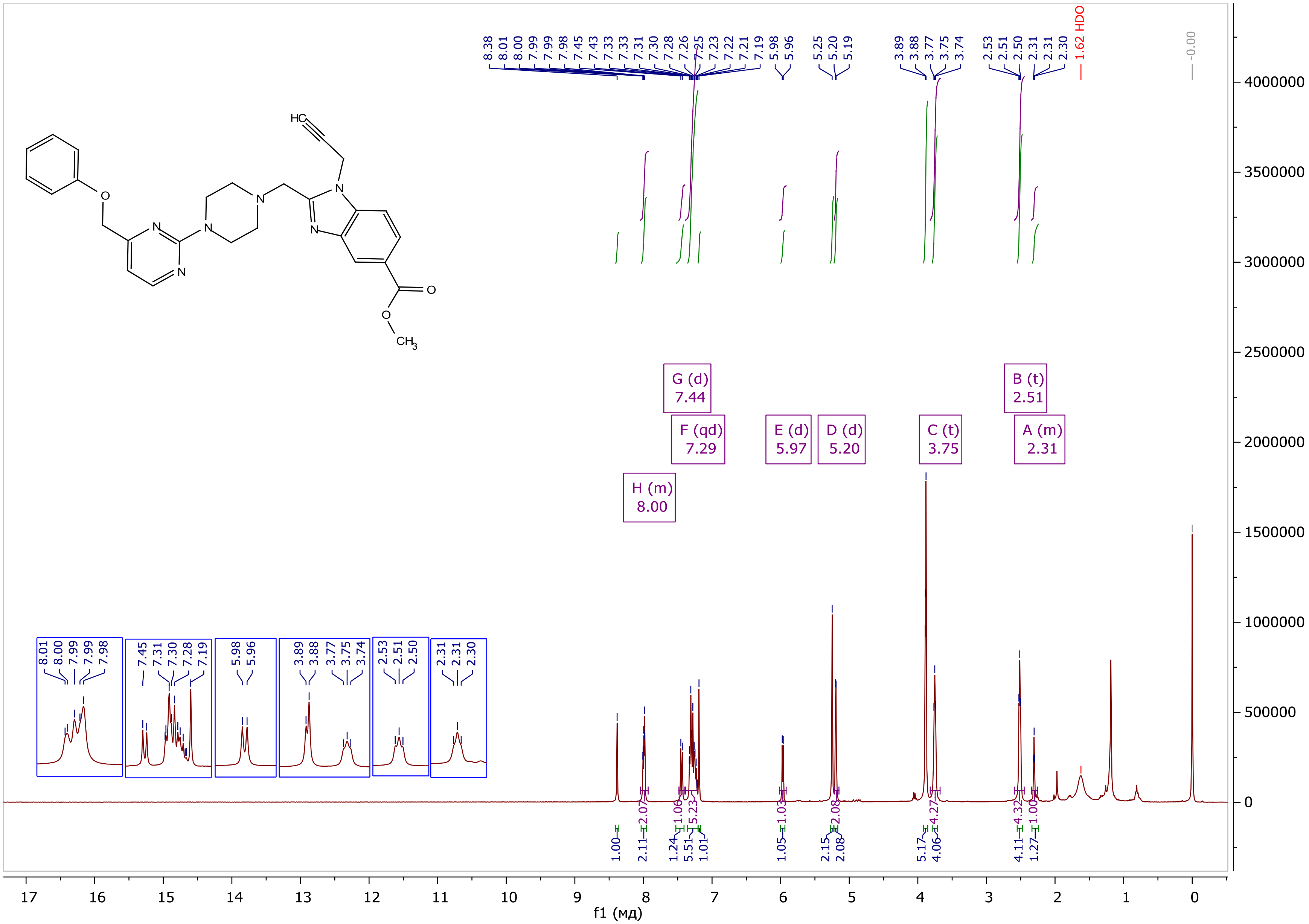

^{13}C NMR spectrum of compound **23g**

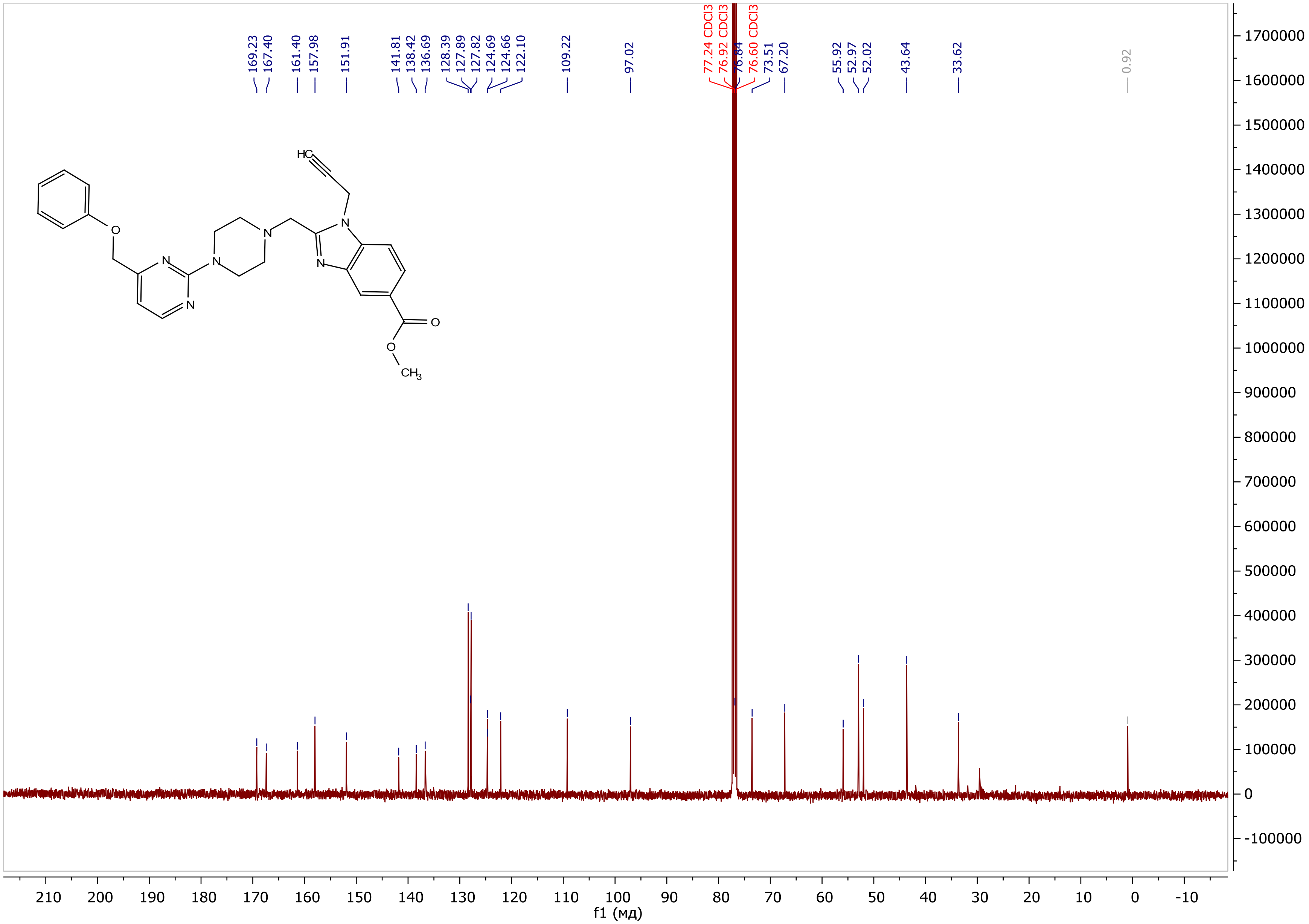

^{1}H NMR spectrum of compound **23h**

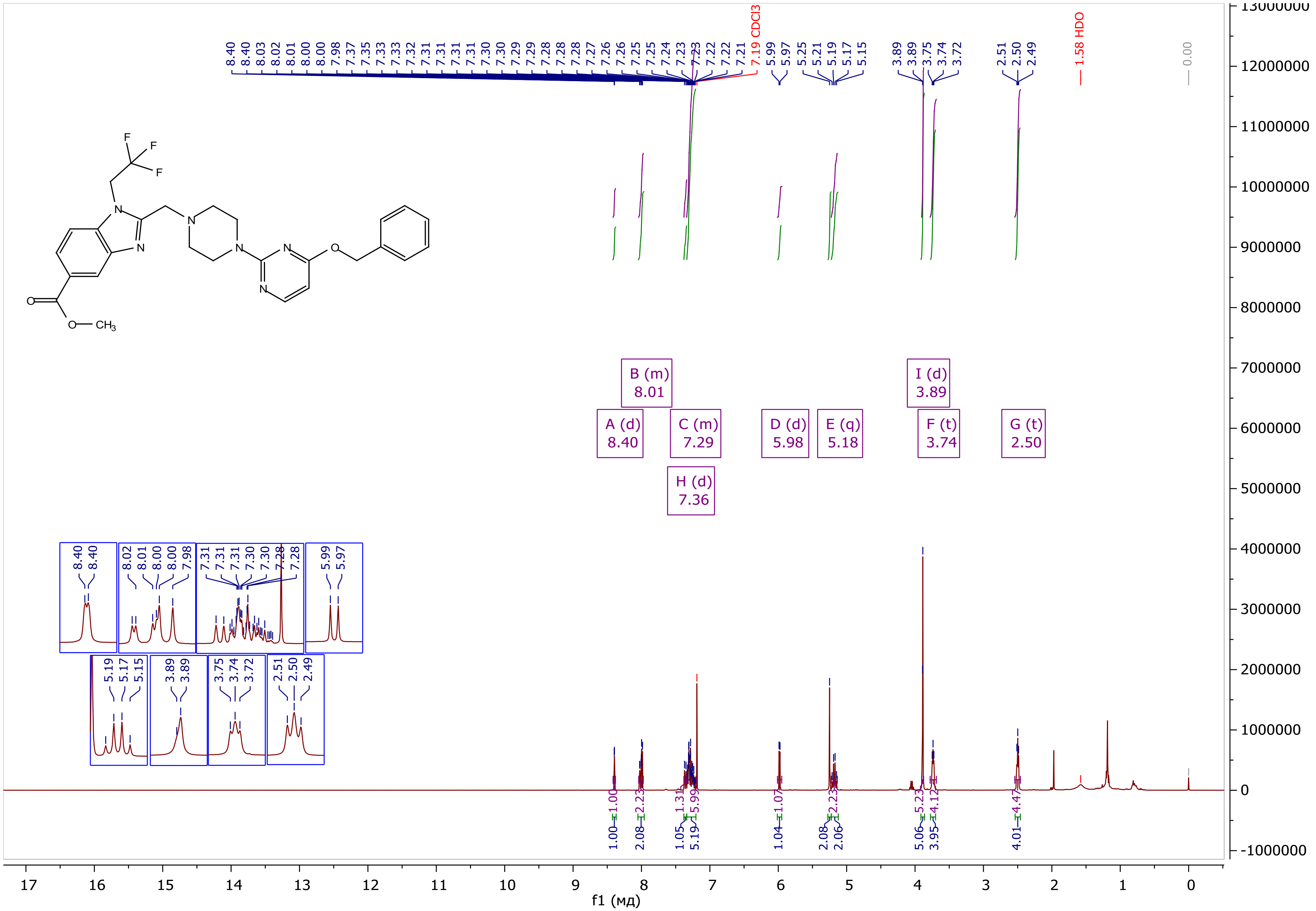

^{13}C NMR spectrum of compound **23h**

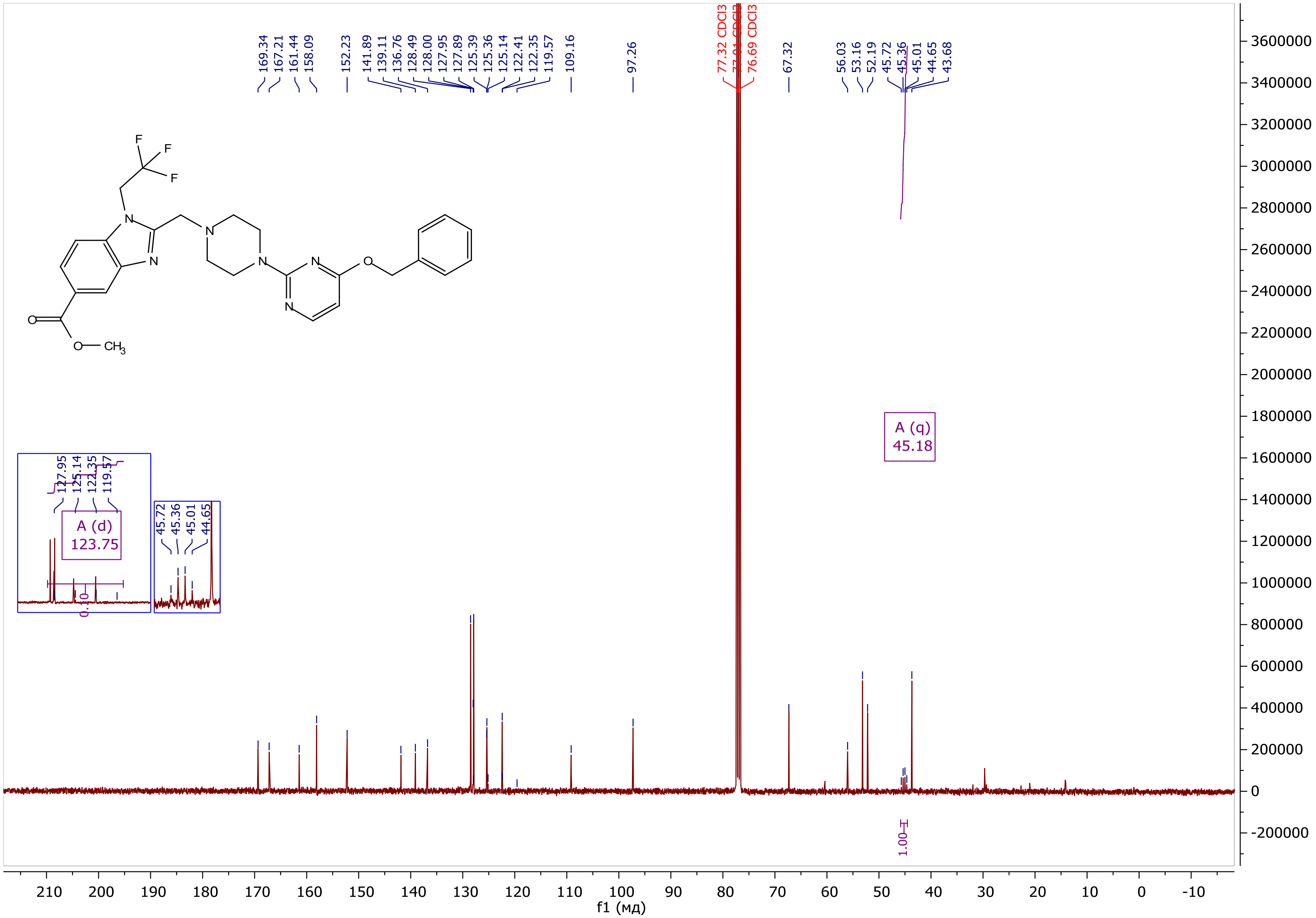

^{1}H NMR spectrum of compound **23i**

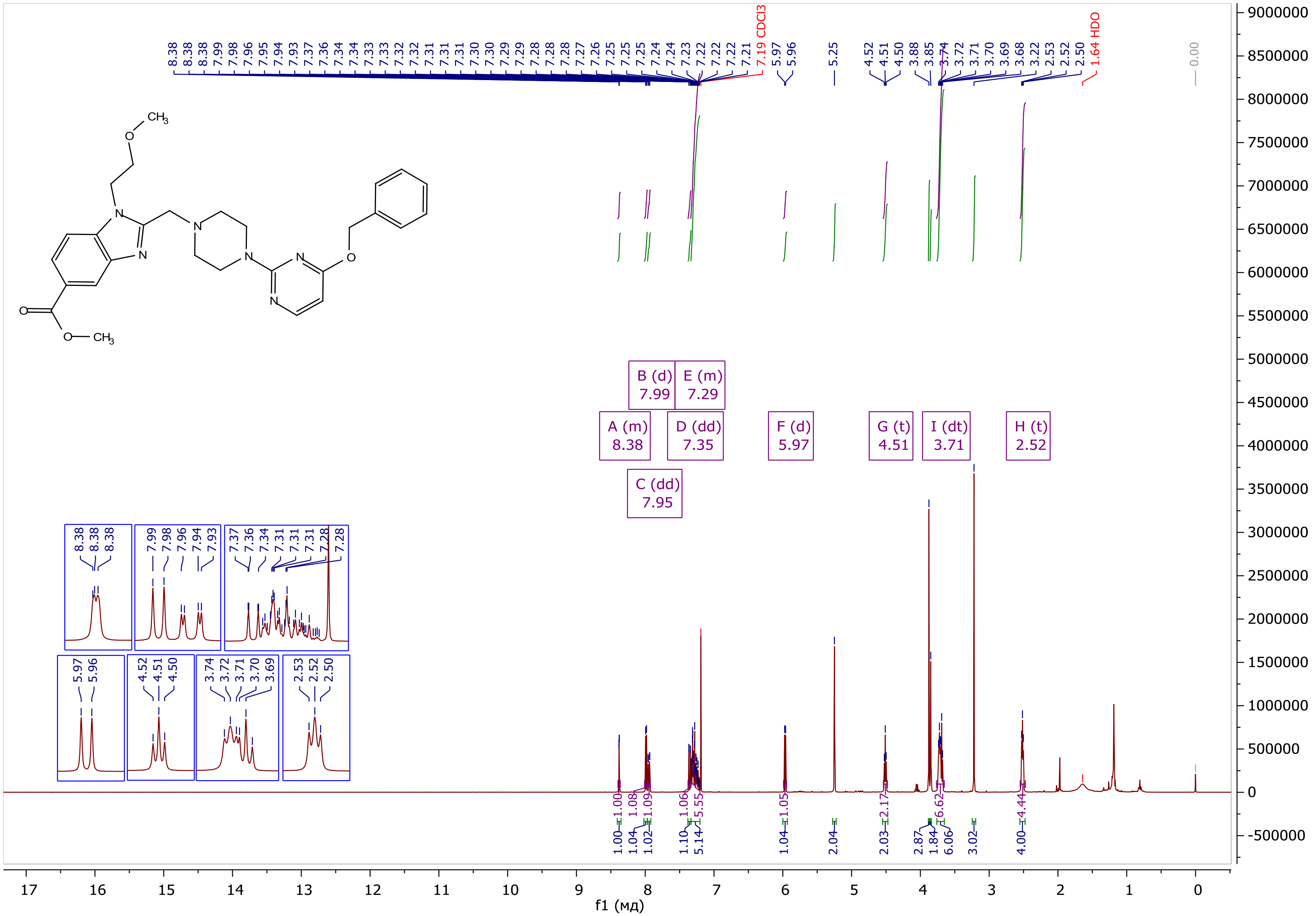

^{13}C NMR spectrum of compound **23i**

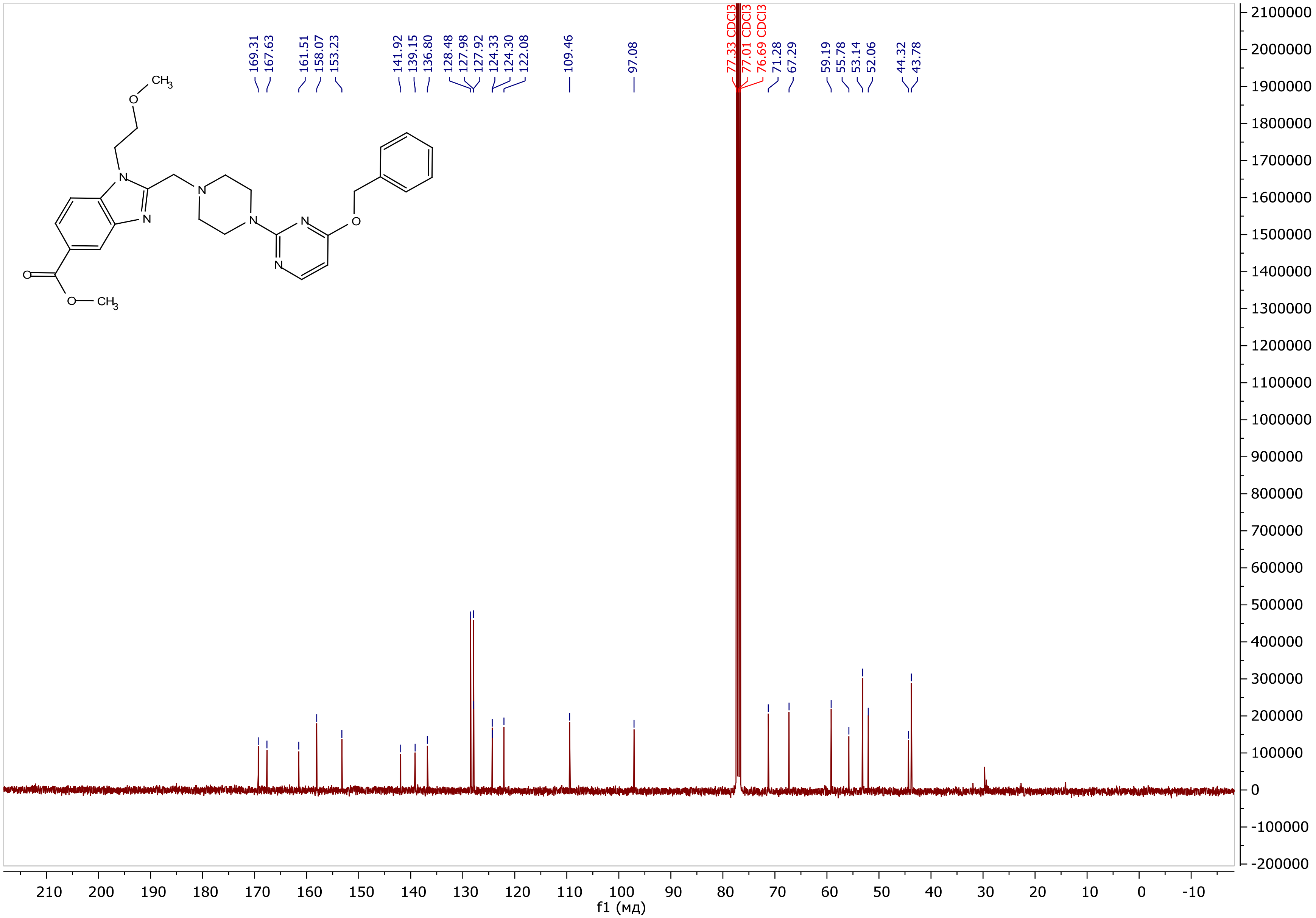

^{1}H NMR spectrum of compound **23j**

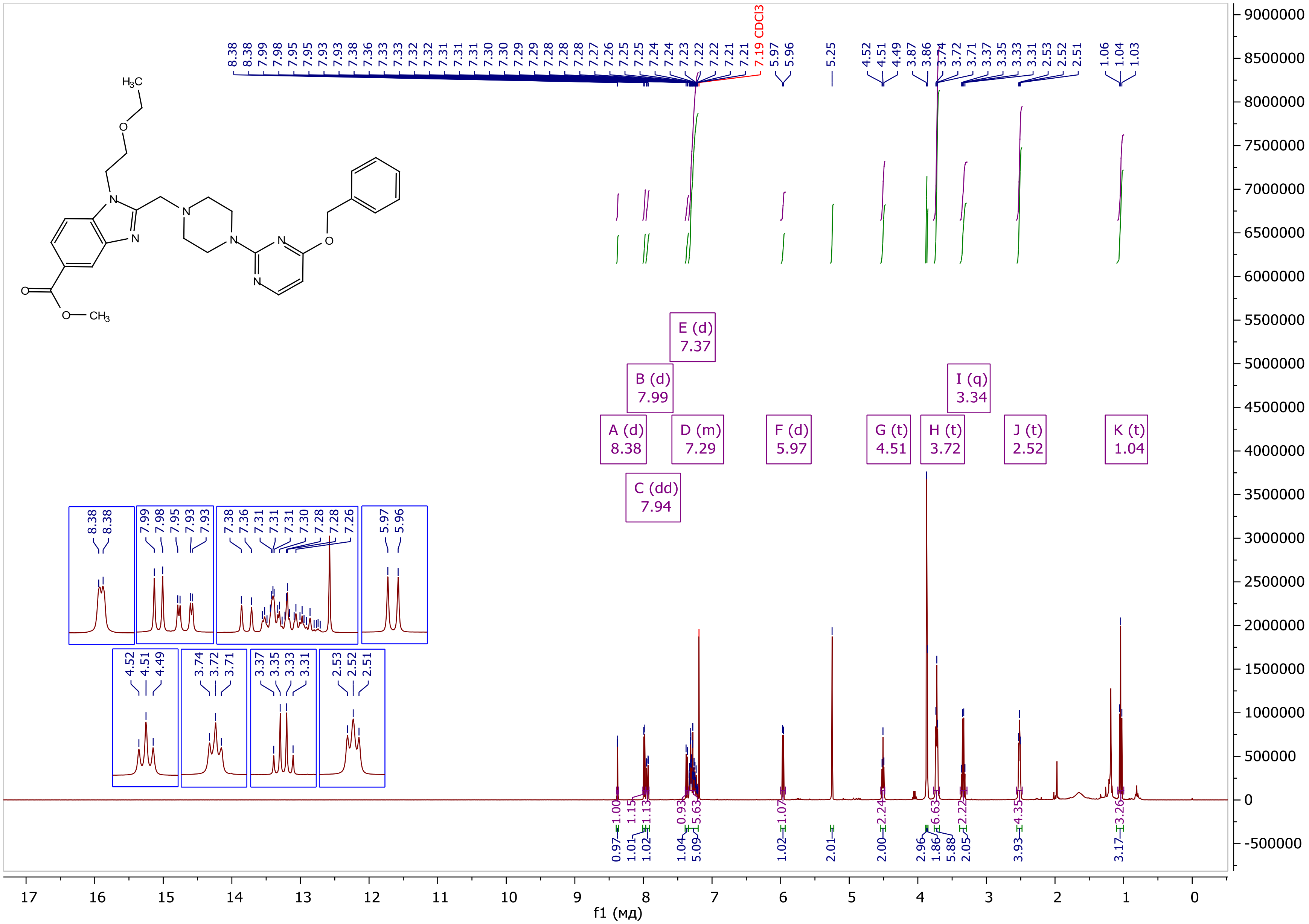

^{13}C NMR spectrum of compound **23j**

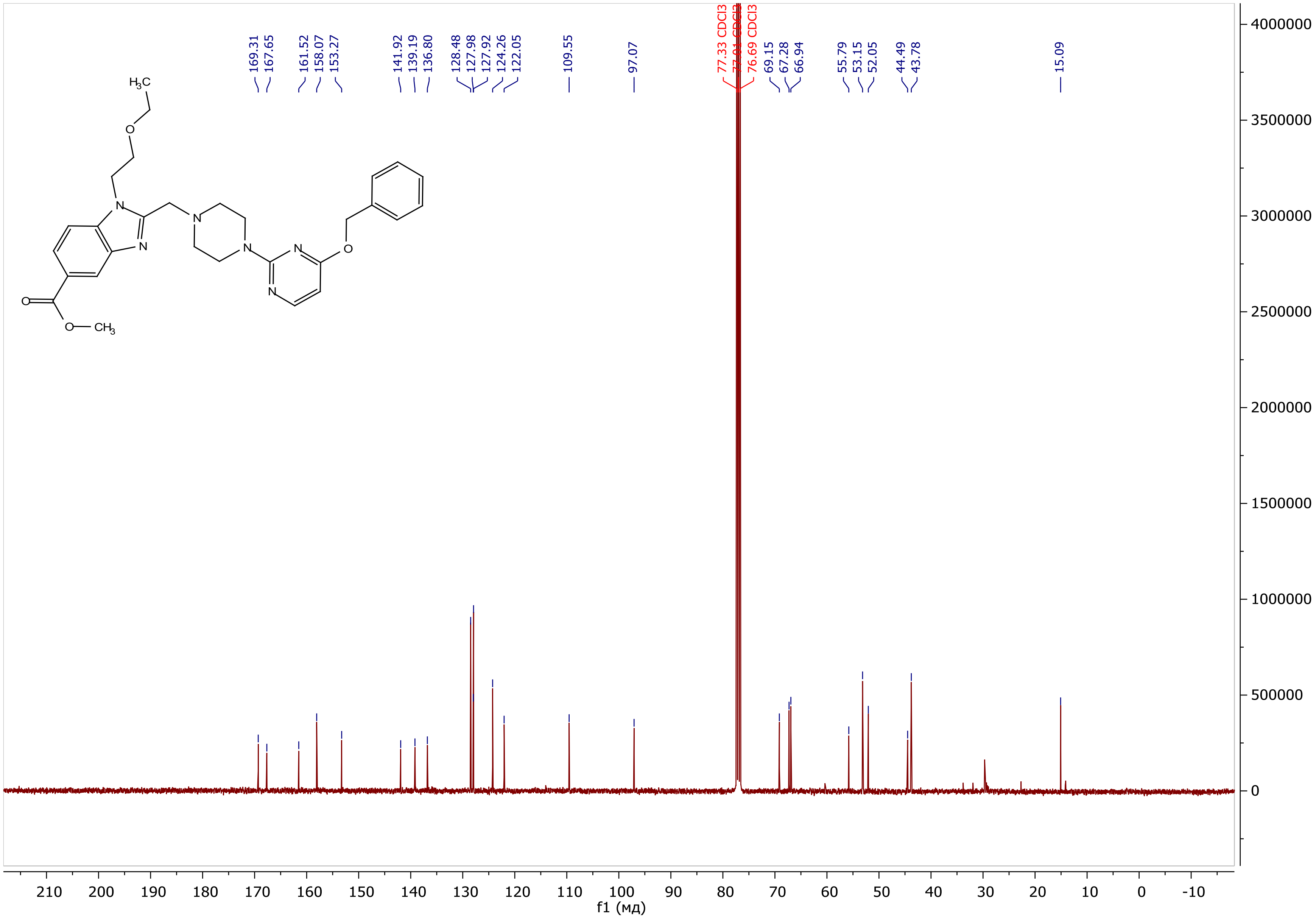

^{1}H NMR spectrum of compound **23k**

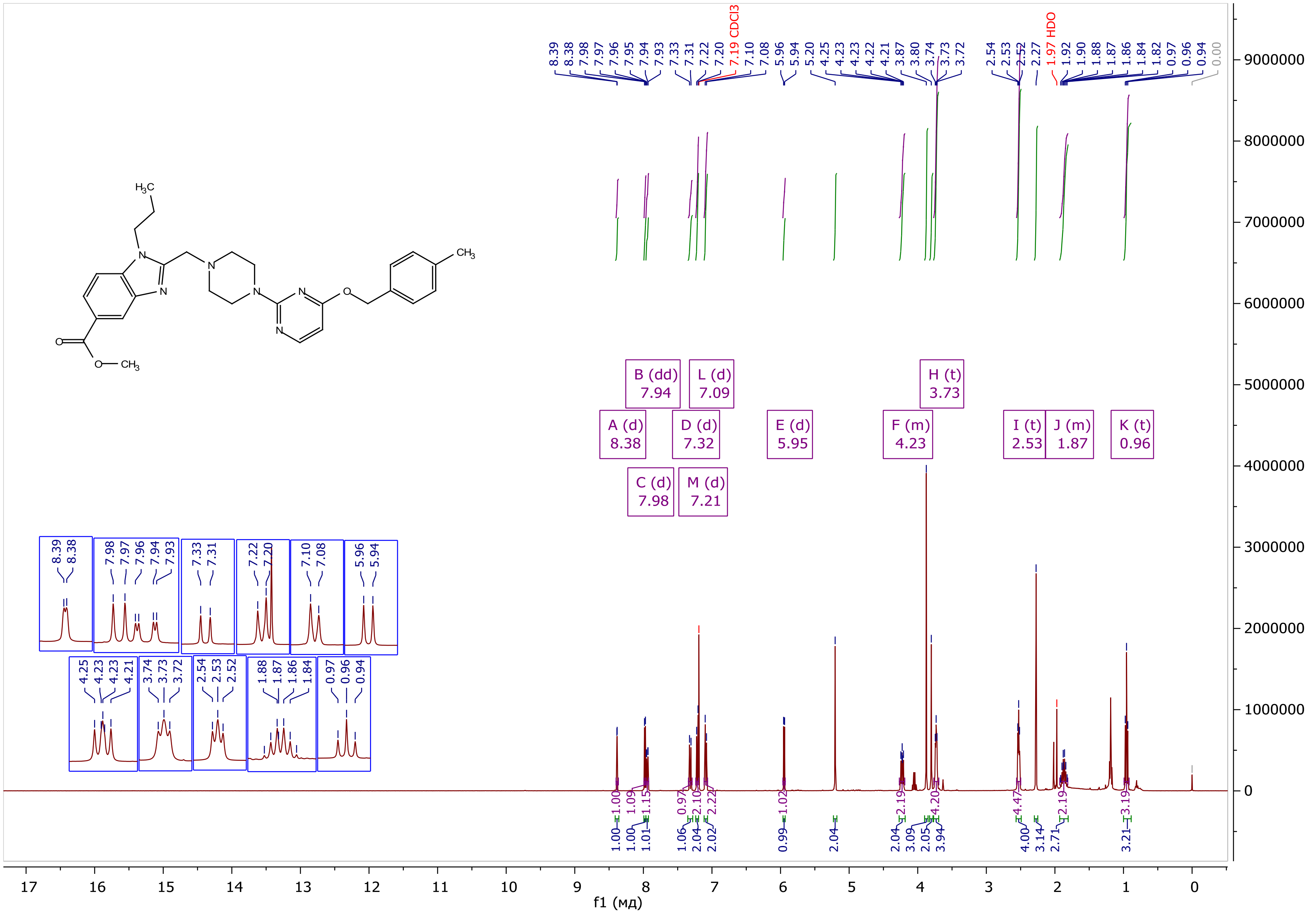

^{13}C NMR spectrum of compound **23k**

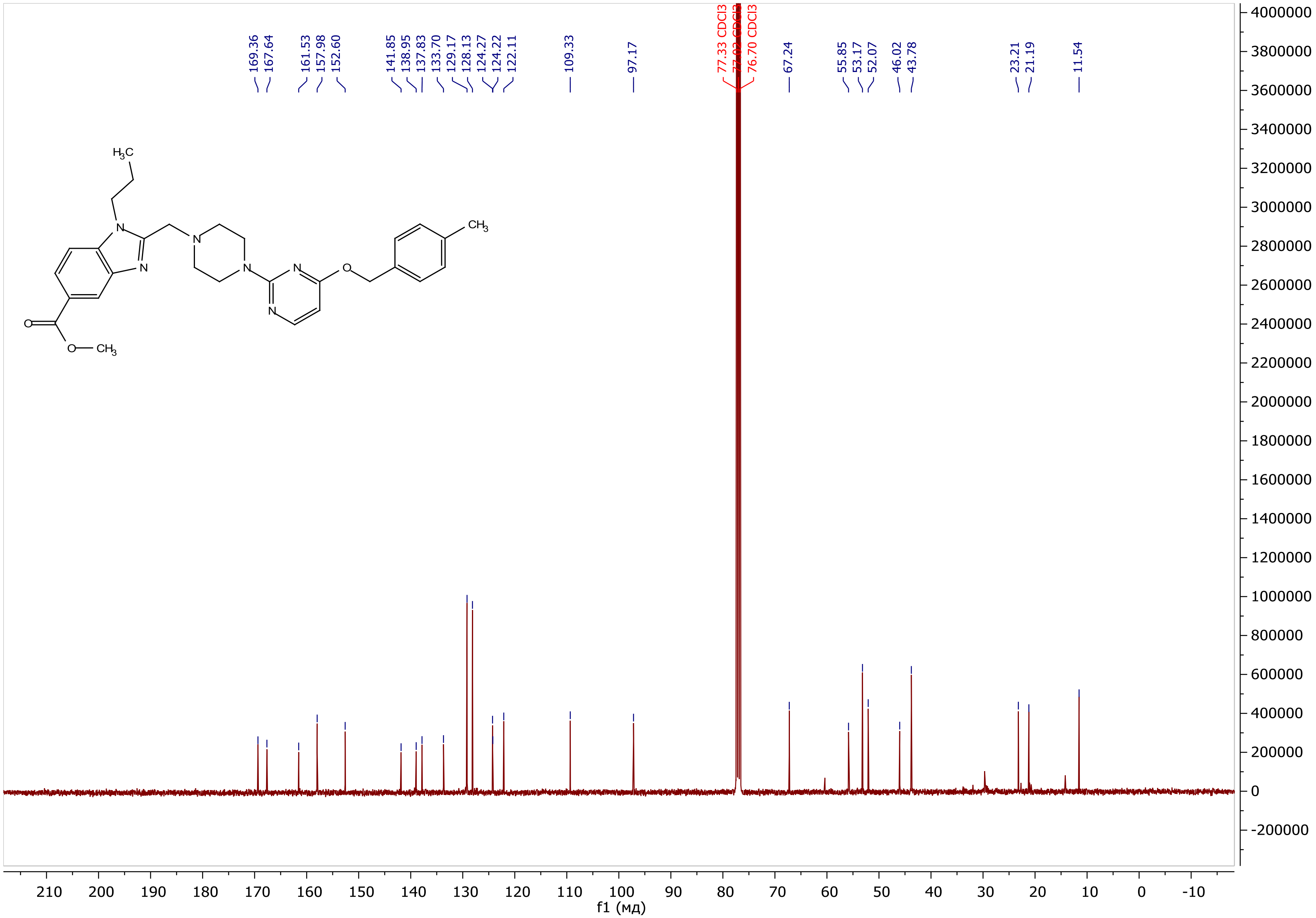

^{1}H NMR spectrum of compound **23l**

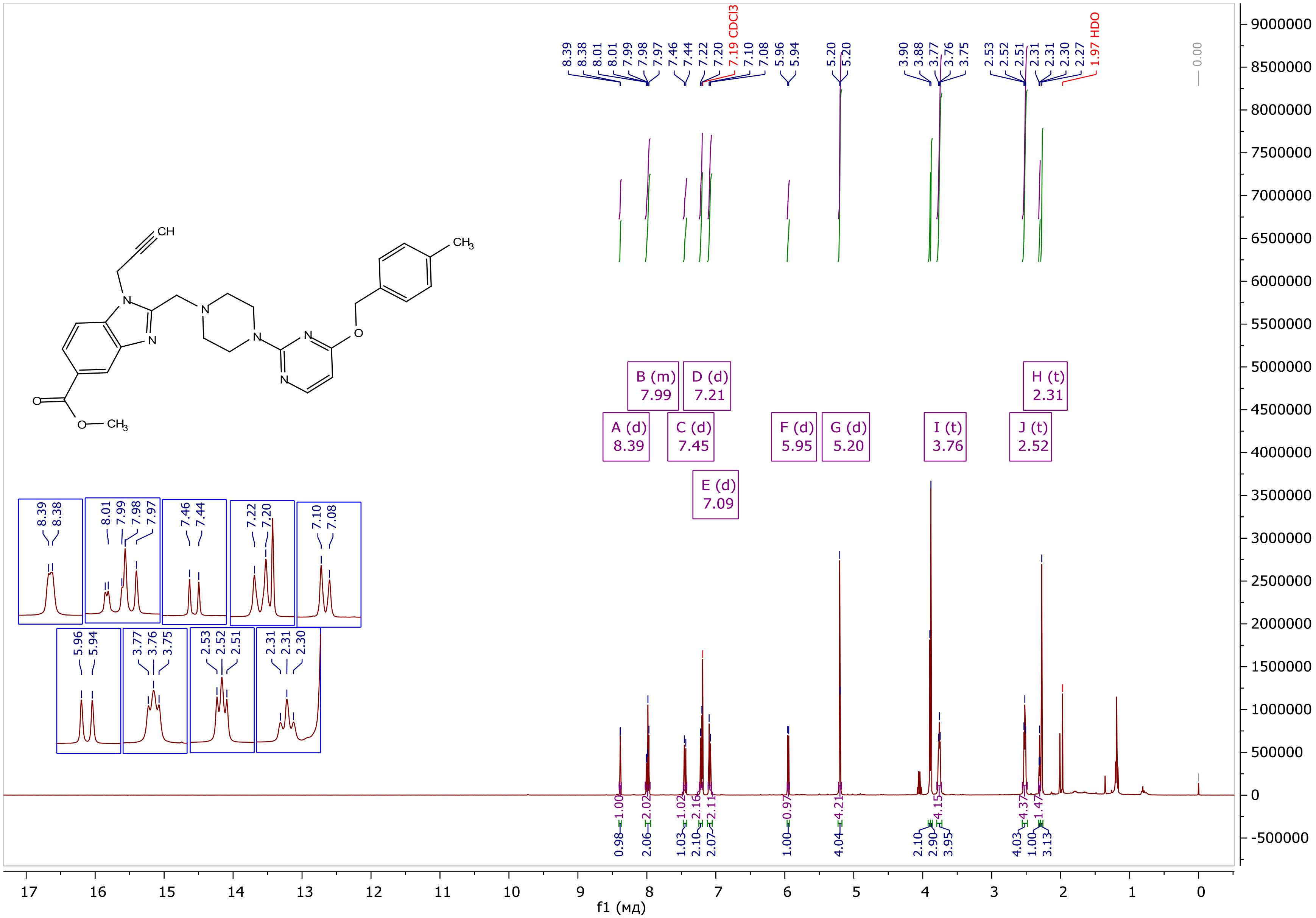

^{13}C NMR spectrum of compound **23l**

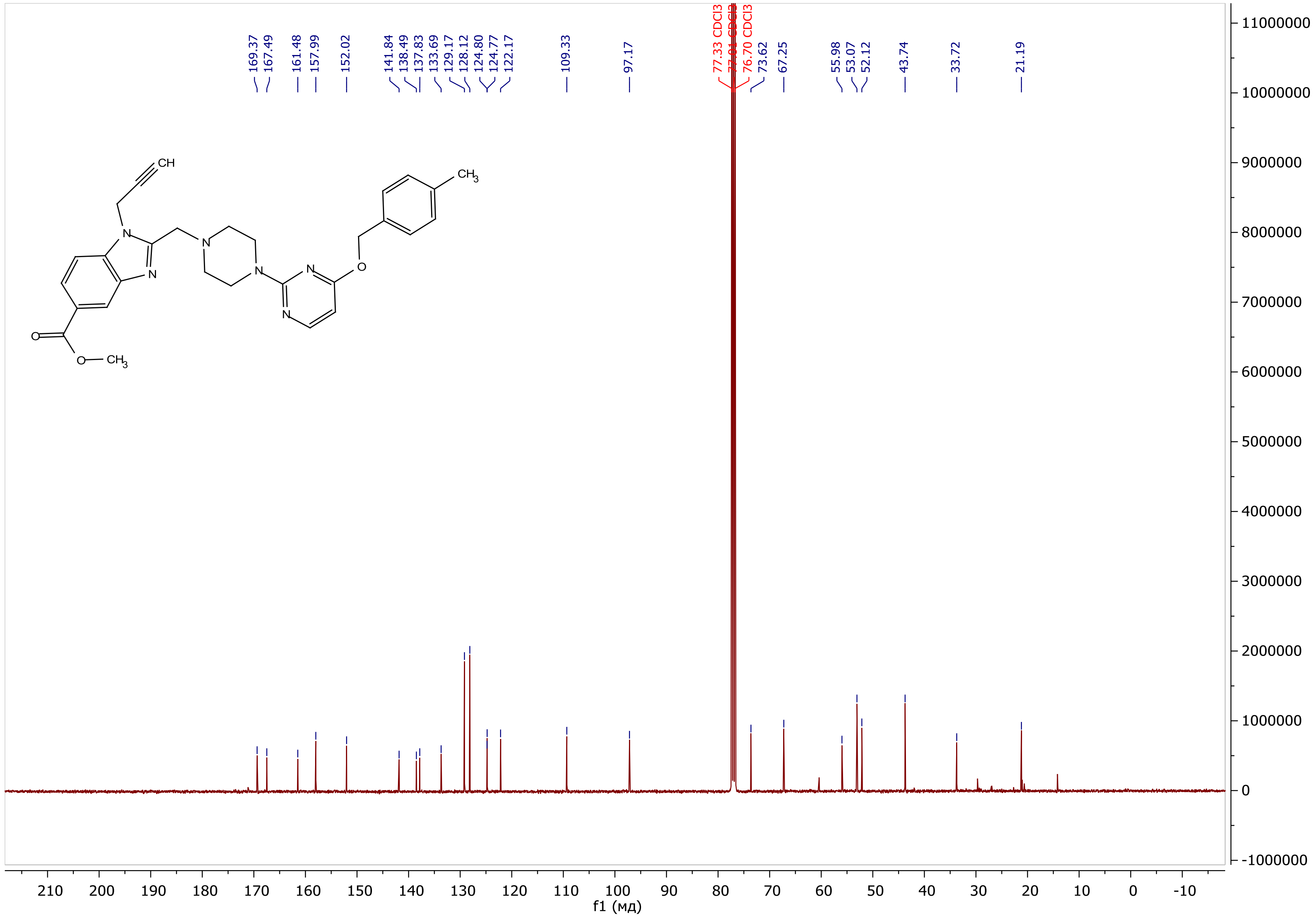

^{1}H NMR spectrum of compound **23m**

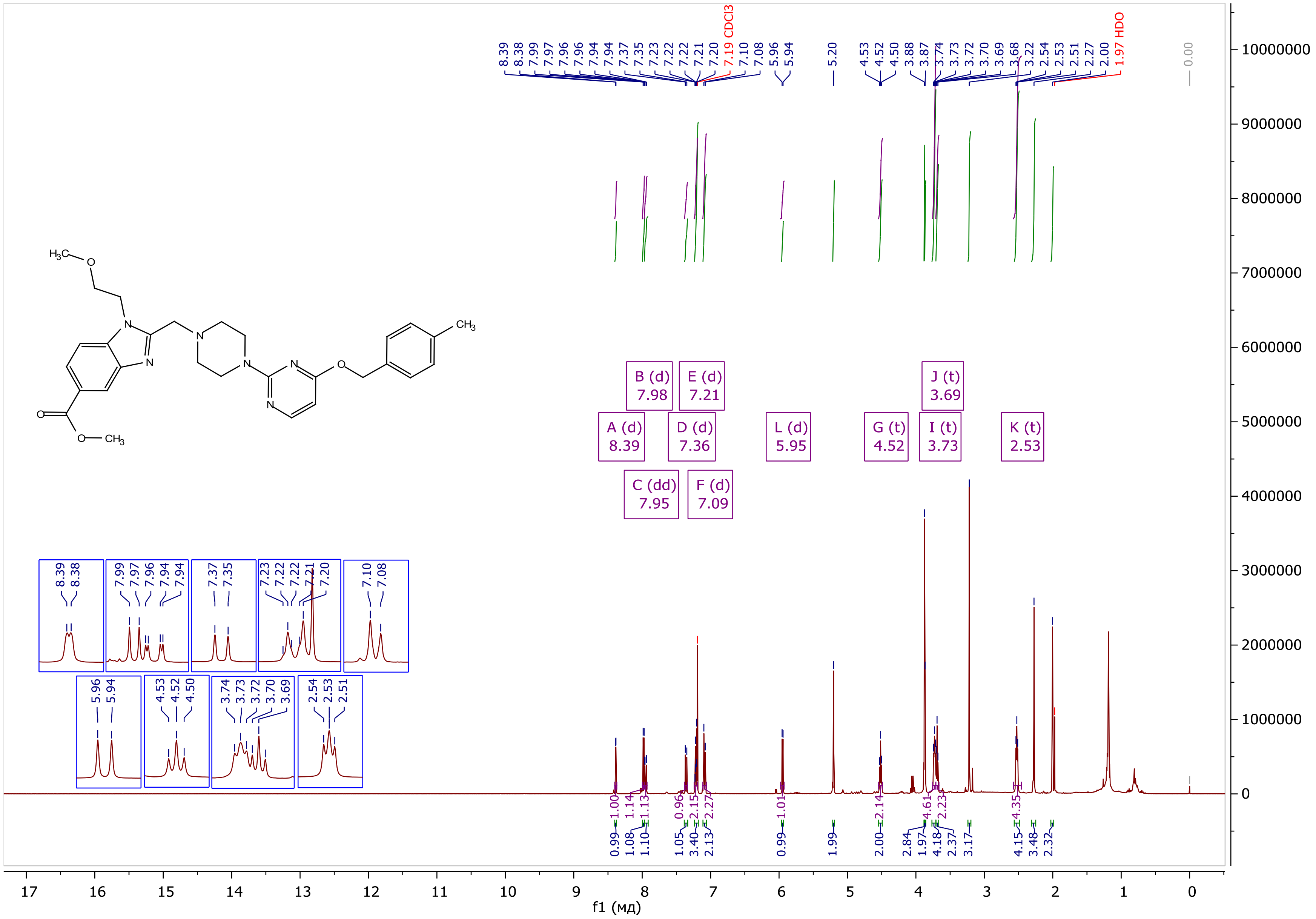

^{13}C NMR spectrum of compound **23m**

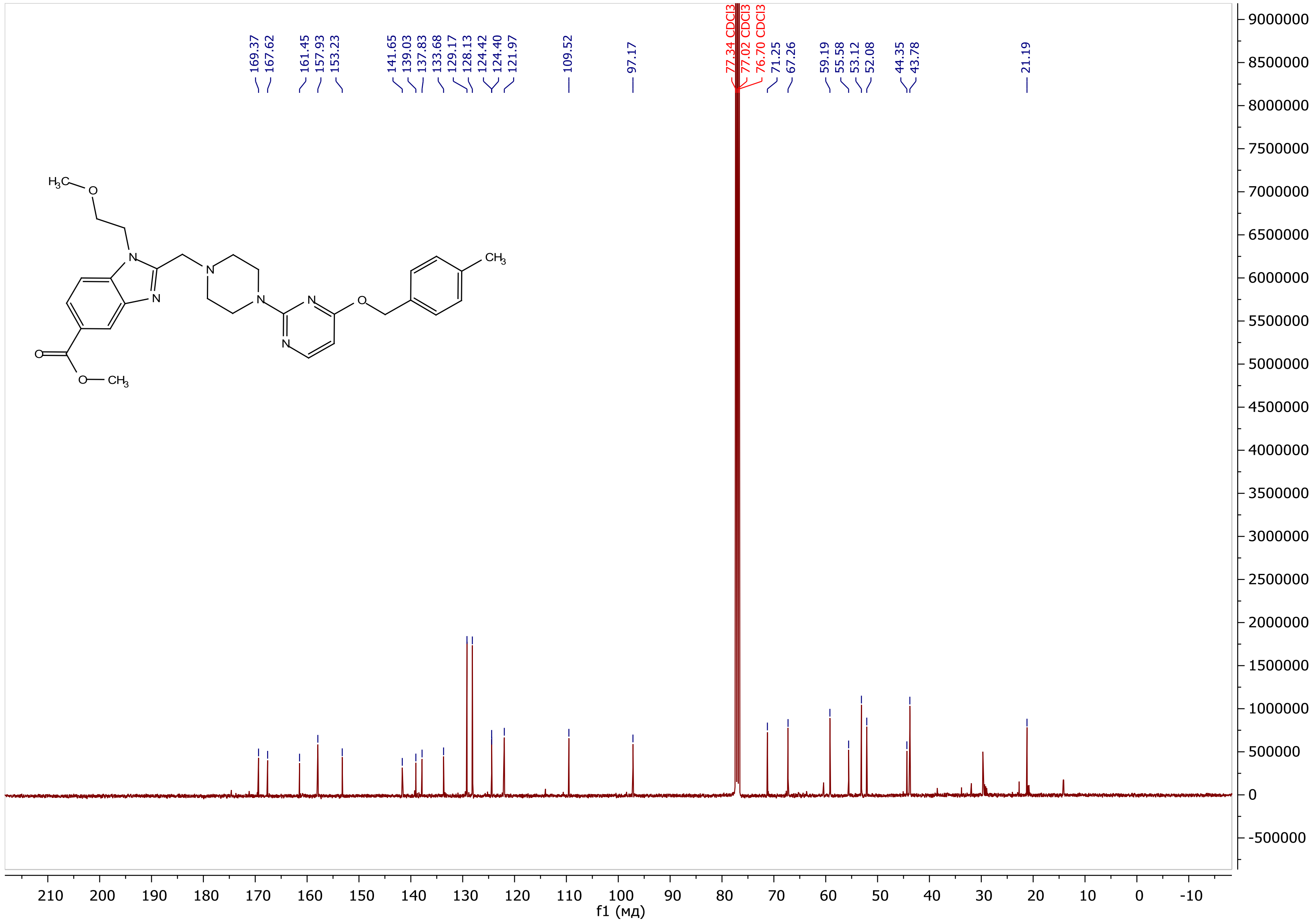

^{1}H NMR spectrum of compound **23n**

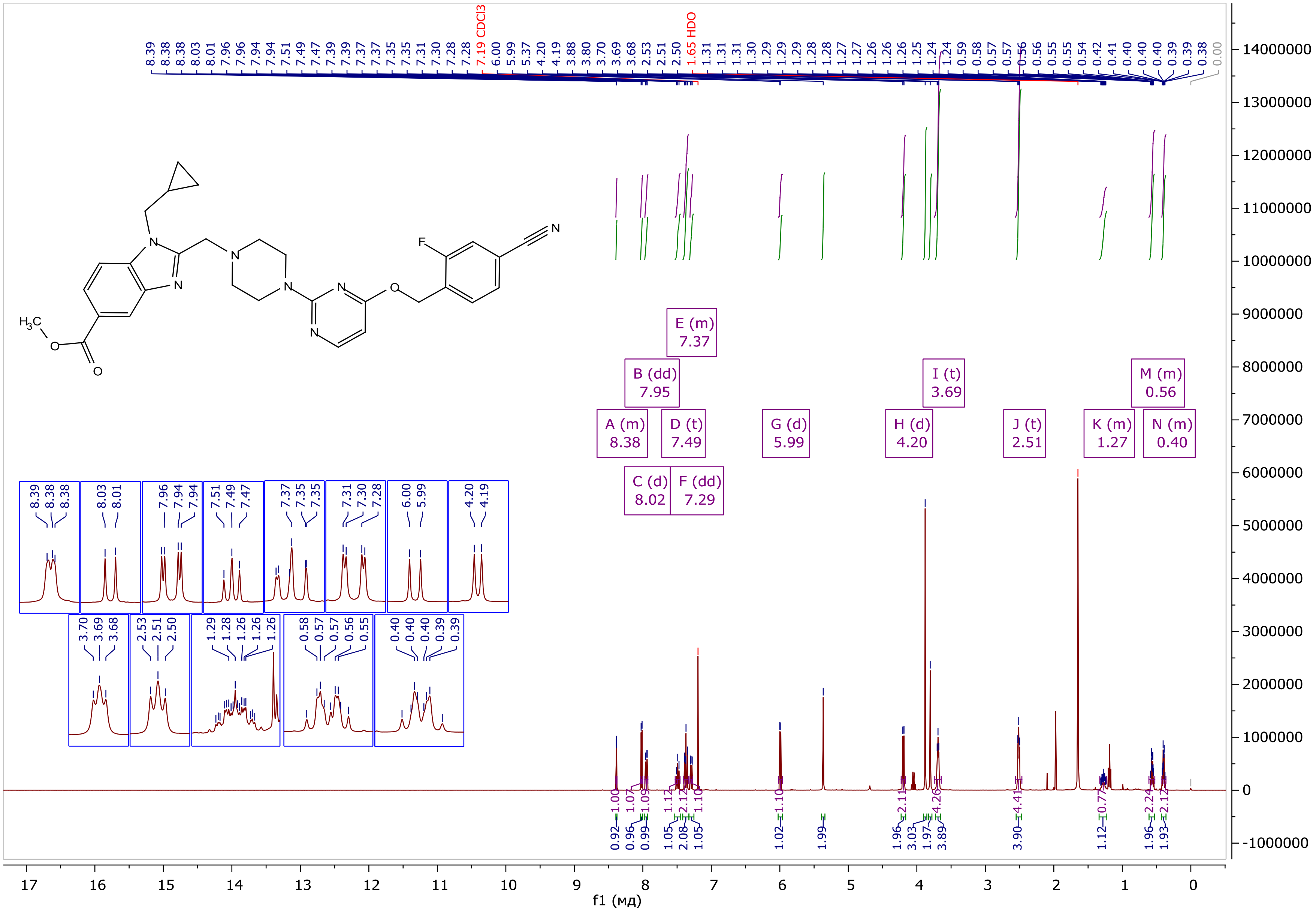

^{13}C NMR spectrum of compound **23n**

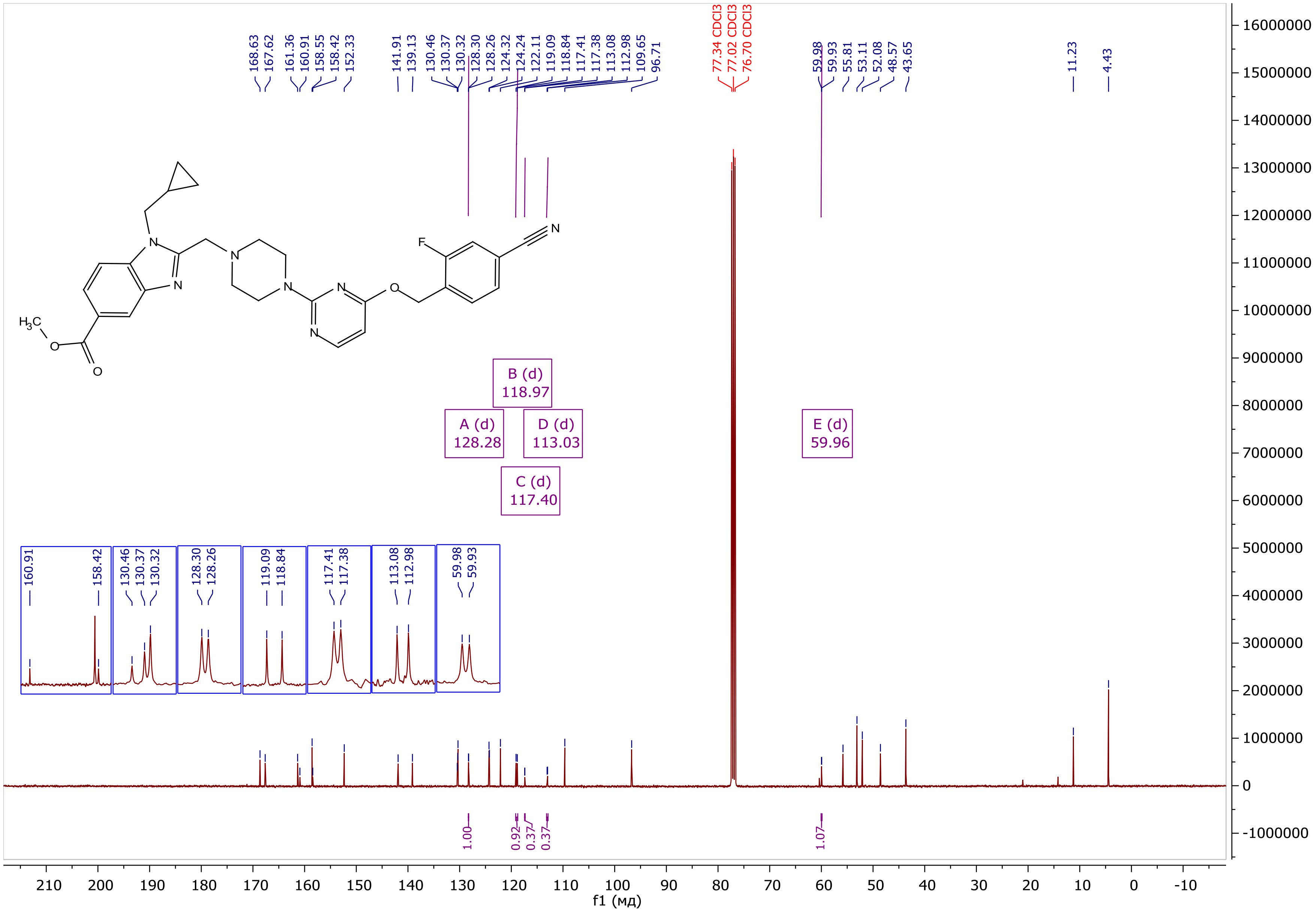

^{1}H NMR spectrum of compound **23o**

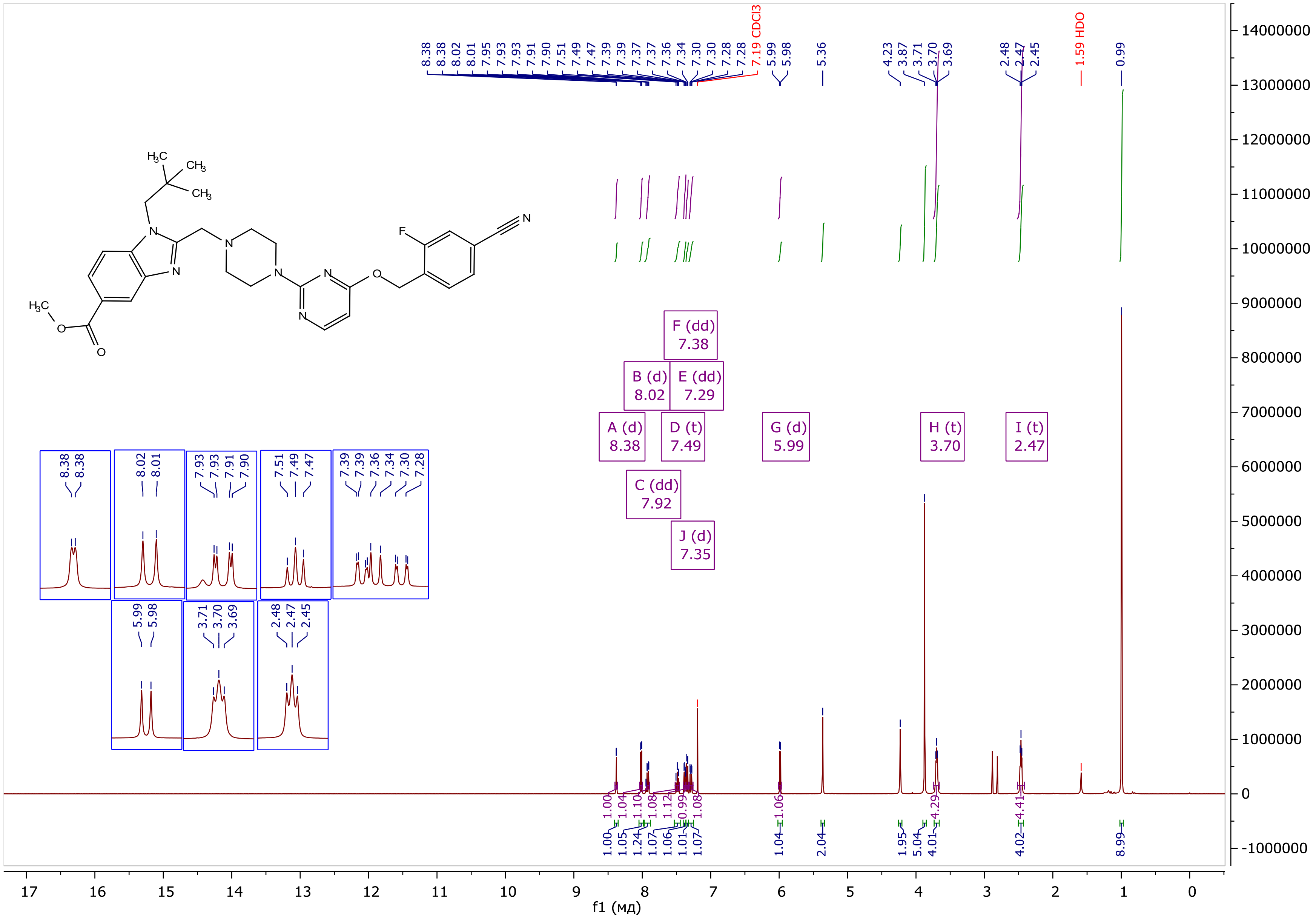

^{13}C NMR spectrum of compound **23o**

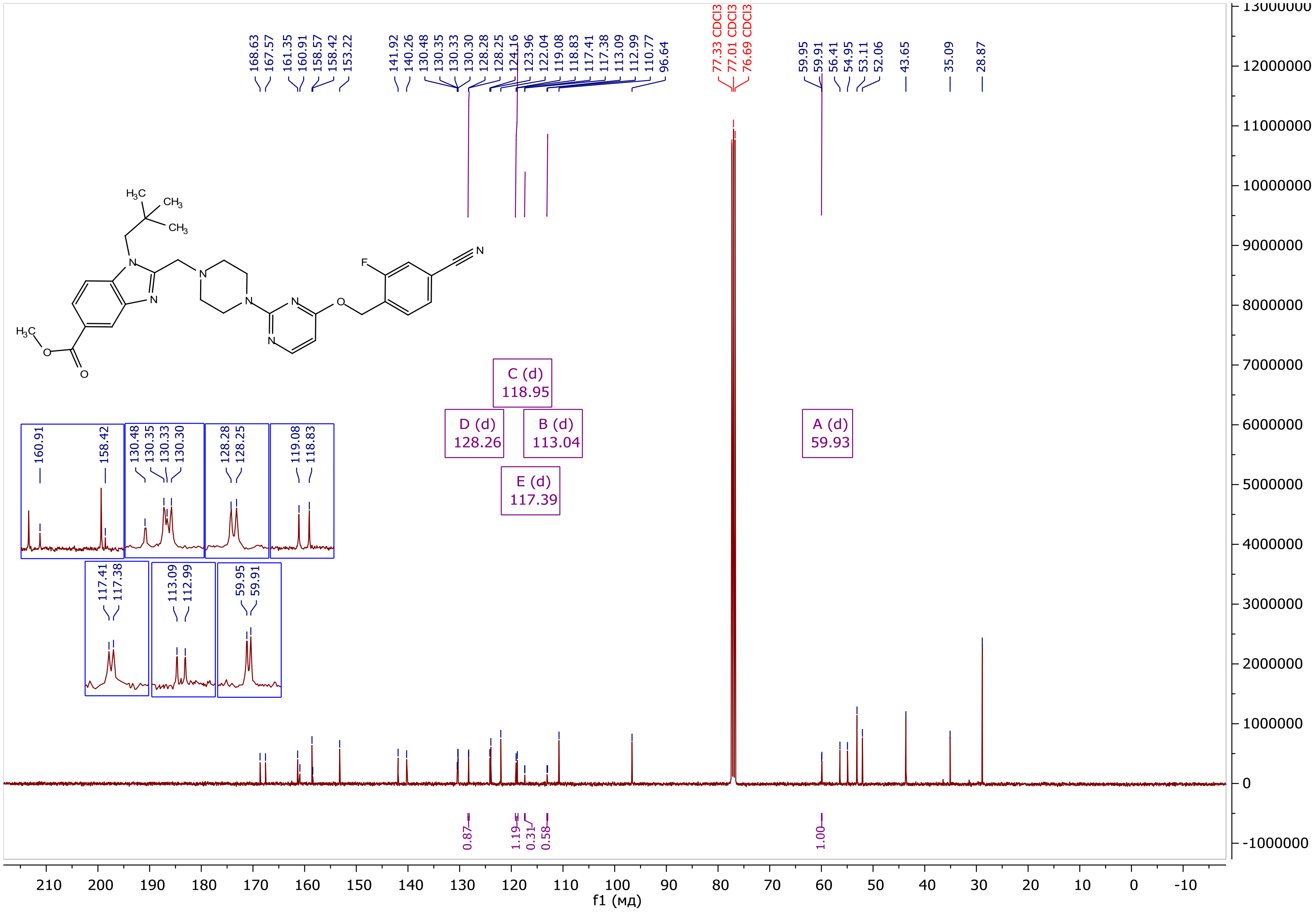

^{1}H NMR spectrum of compound **23p**

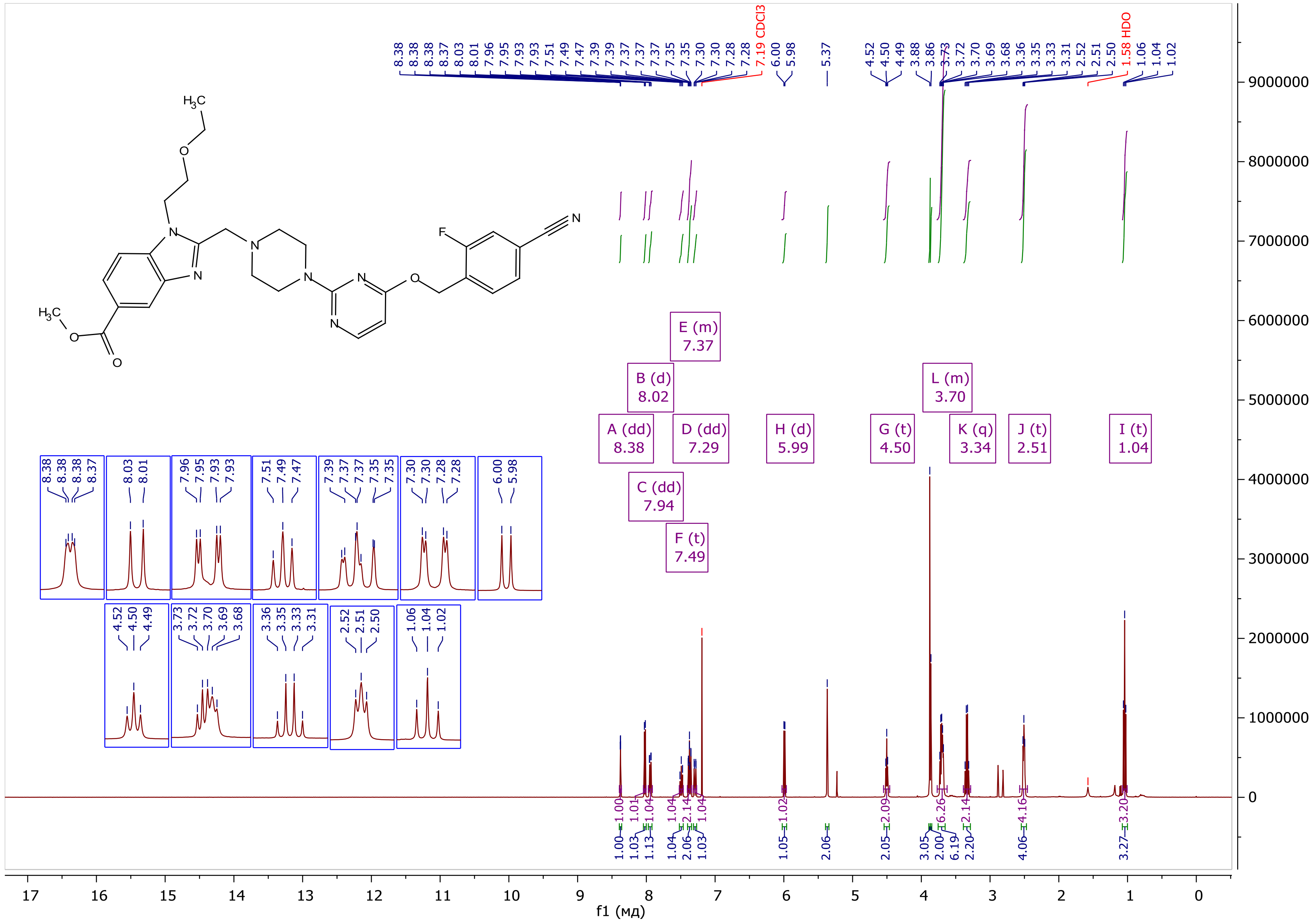

^{13}C NMR spectrum of compound **23p**

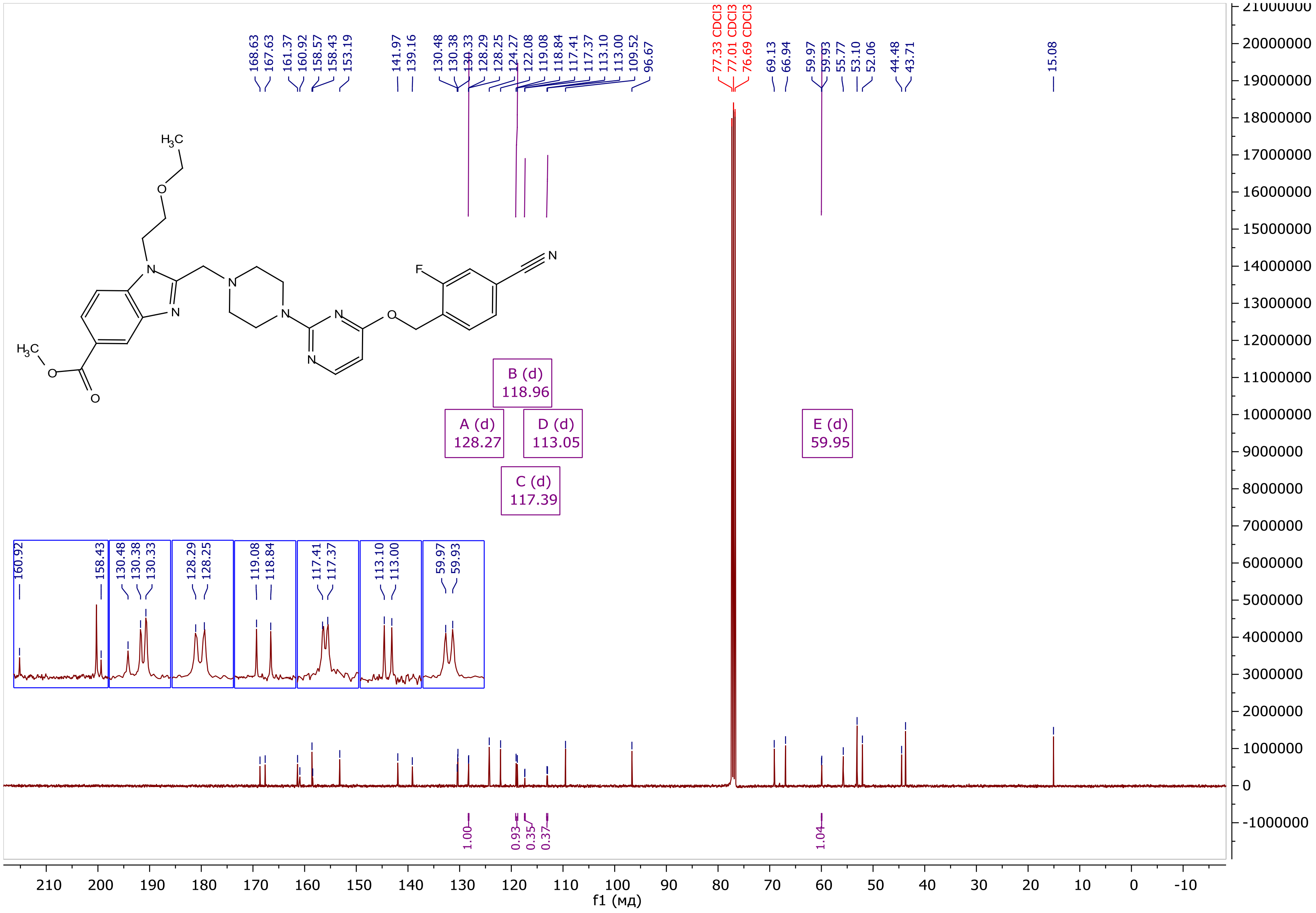

^{1}H NMR spectrum of compound **23q**

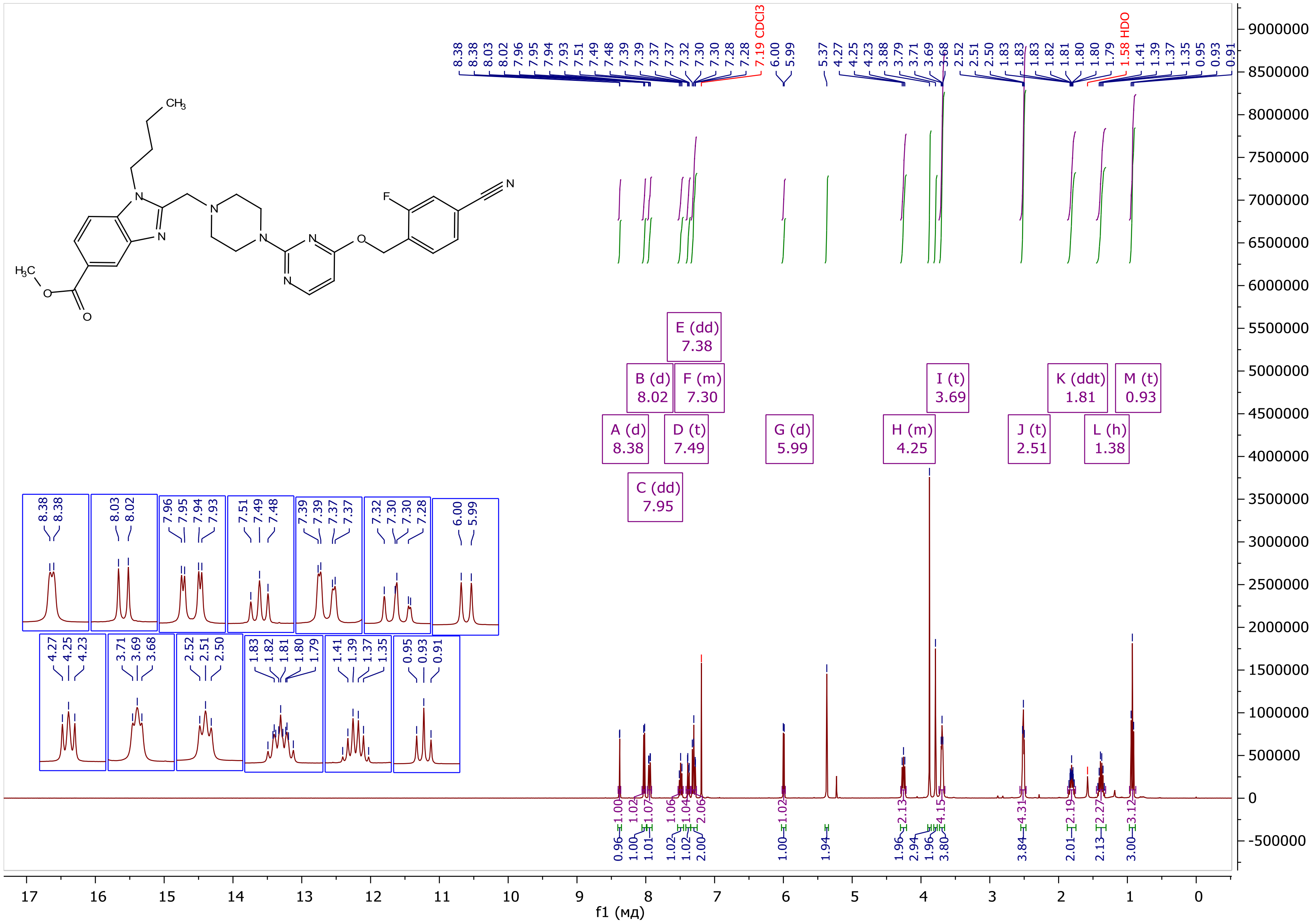

^{13}C NMR spectrum of compound **23q**

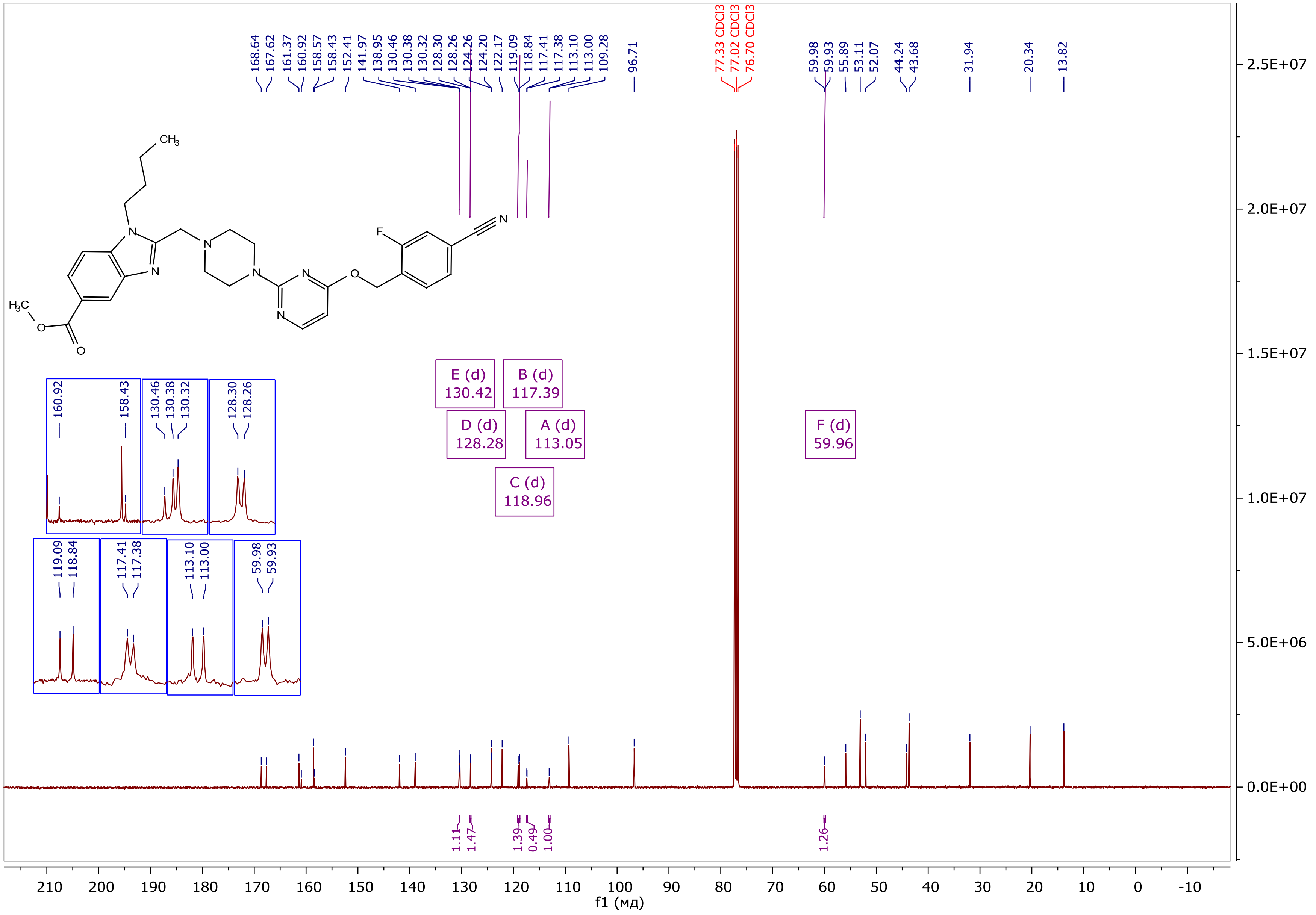

^{1}H NMR spectrum of compound **23r**

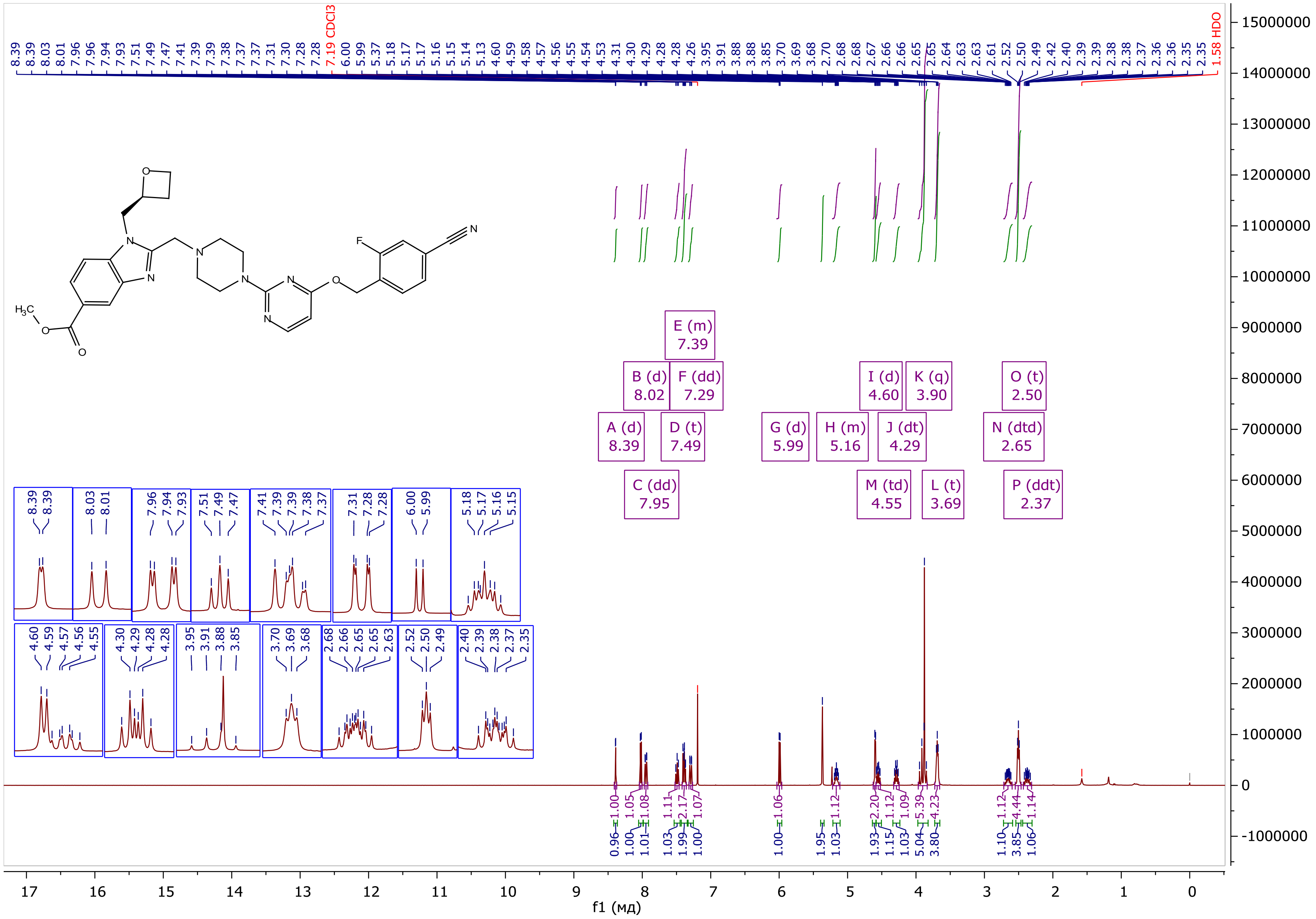

^{13}C NMR spectrum of compound **23r**

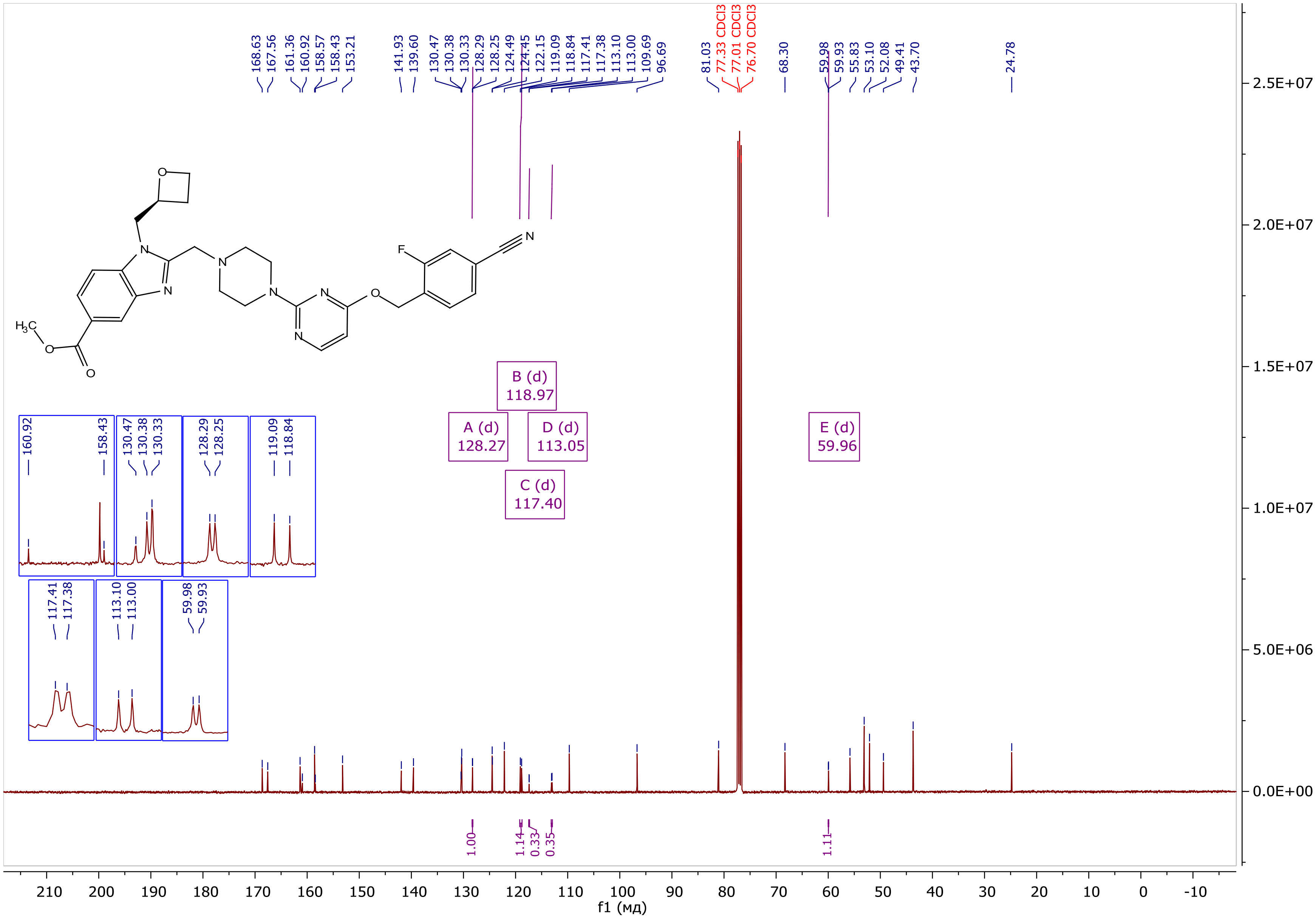

^{1}H NMR spectrum of compound **23s**

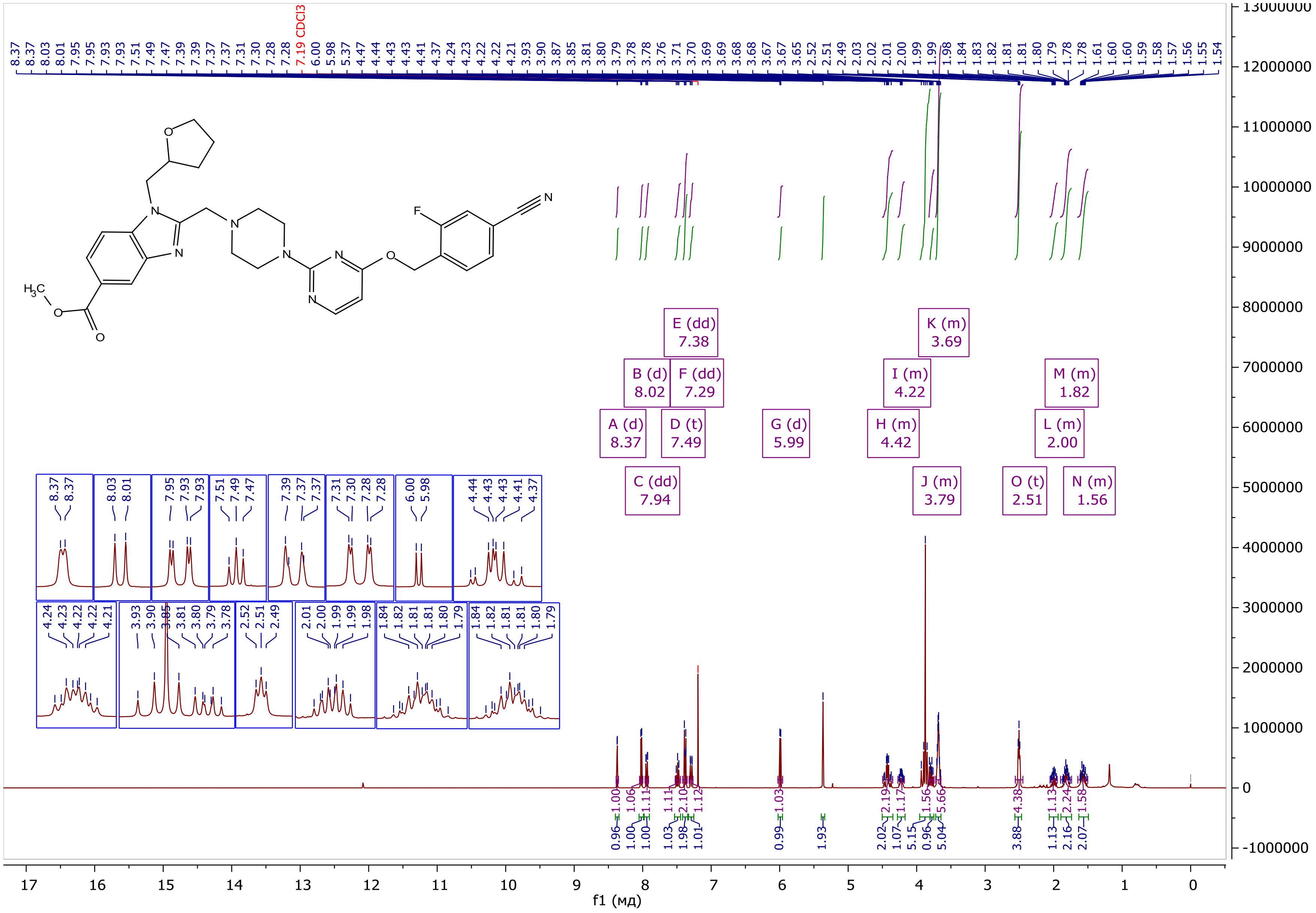

^{13}C NMR spectrum of compound **23s**

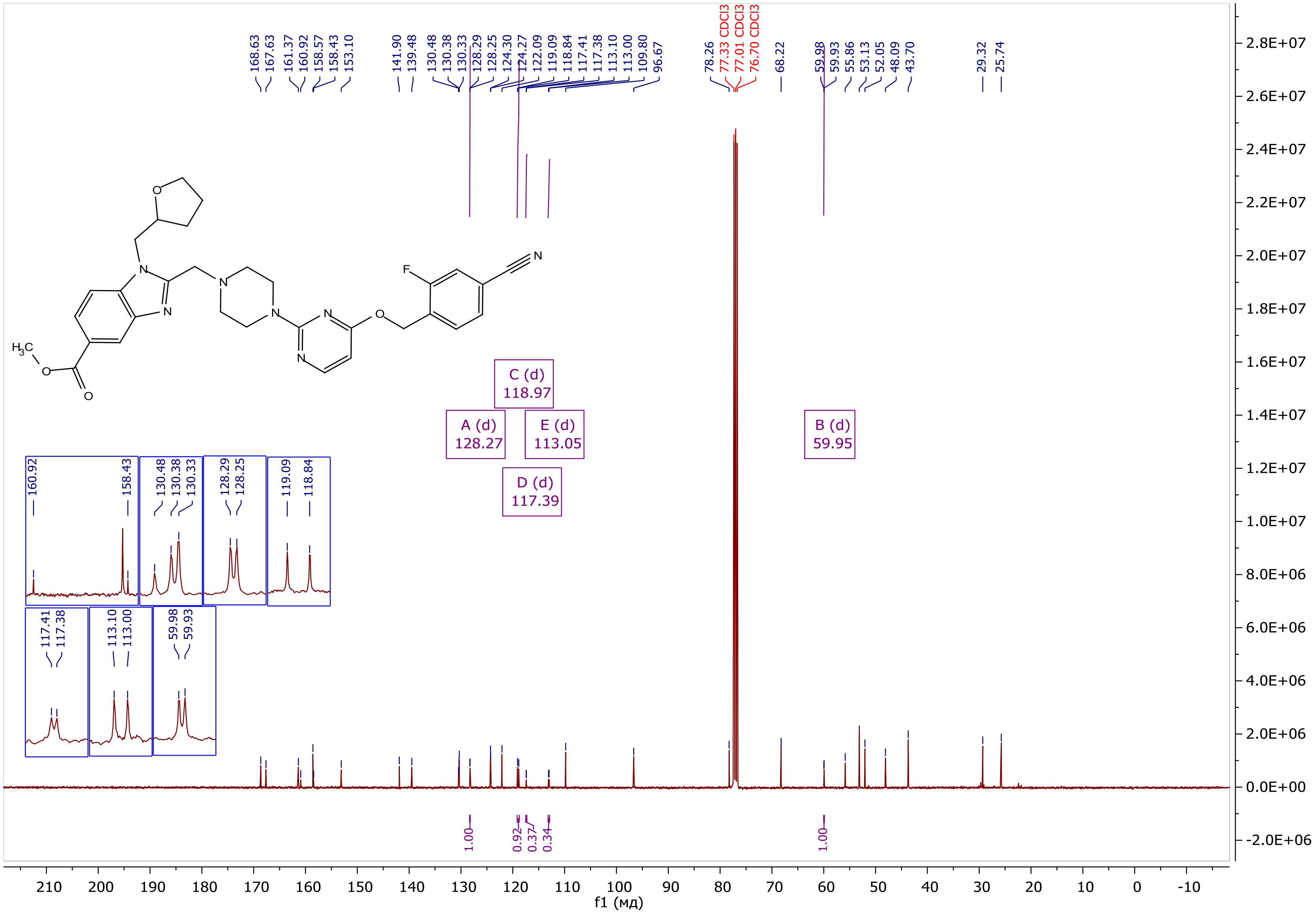

^{1}H NMR spectrum of compound **23t**

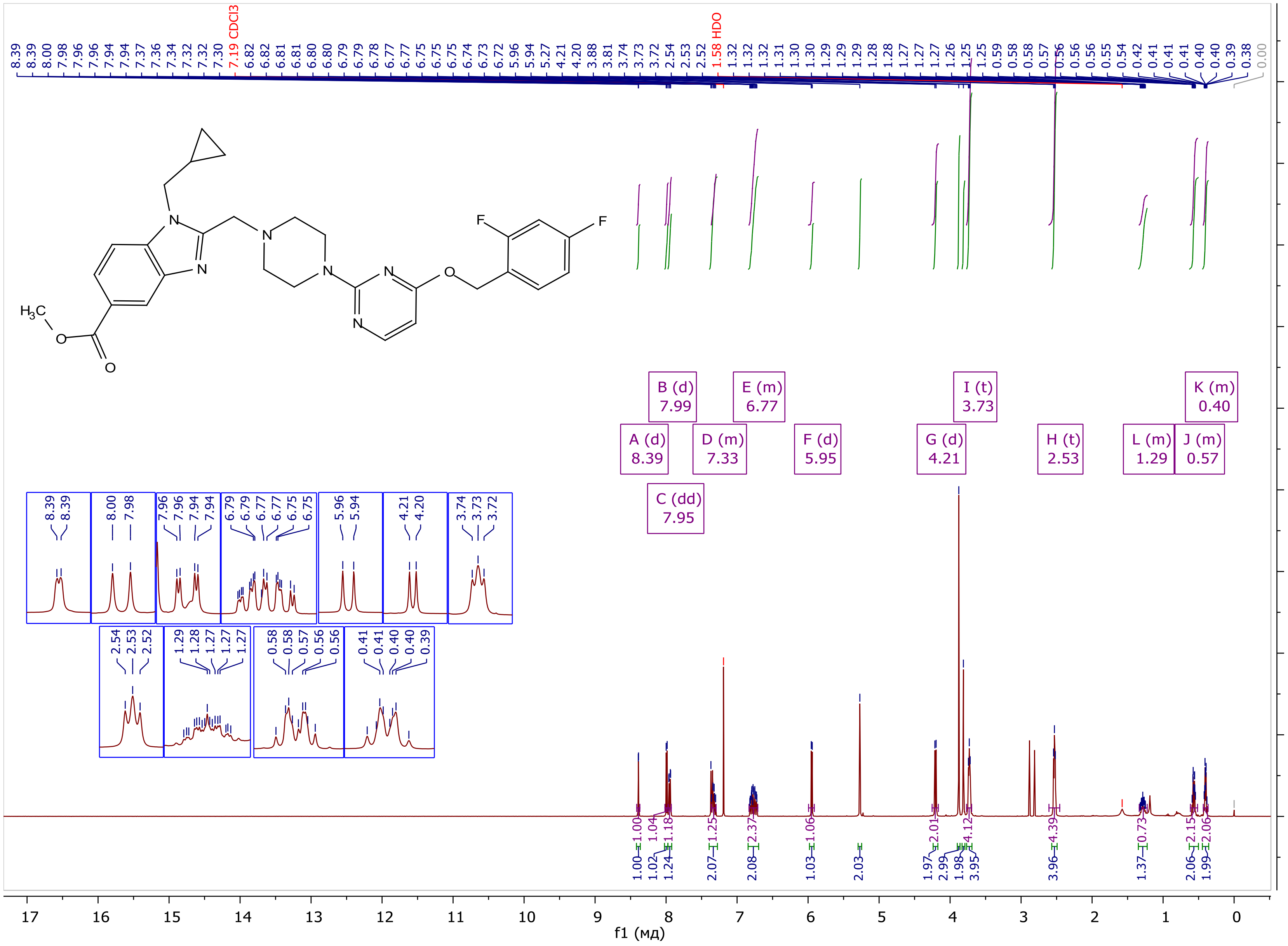

^{13}C NMR spectrum of compound **23t**

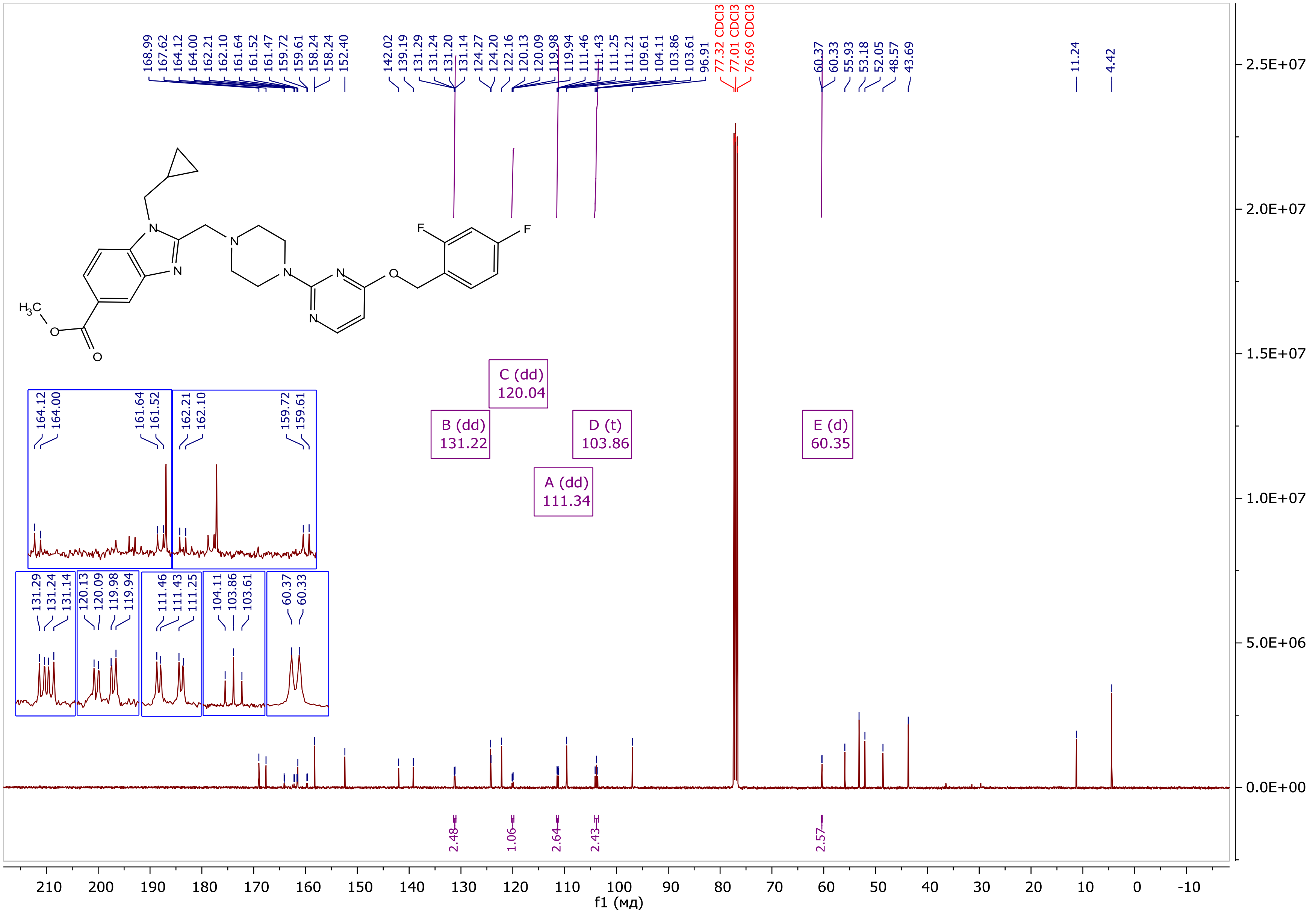

^{1}H NMR spectrum of compound **23u**

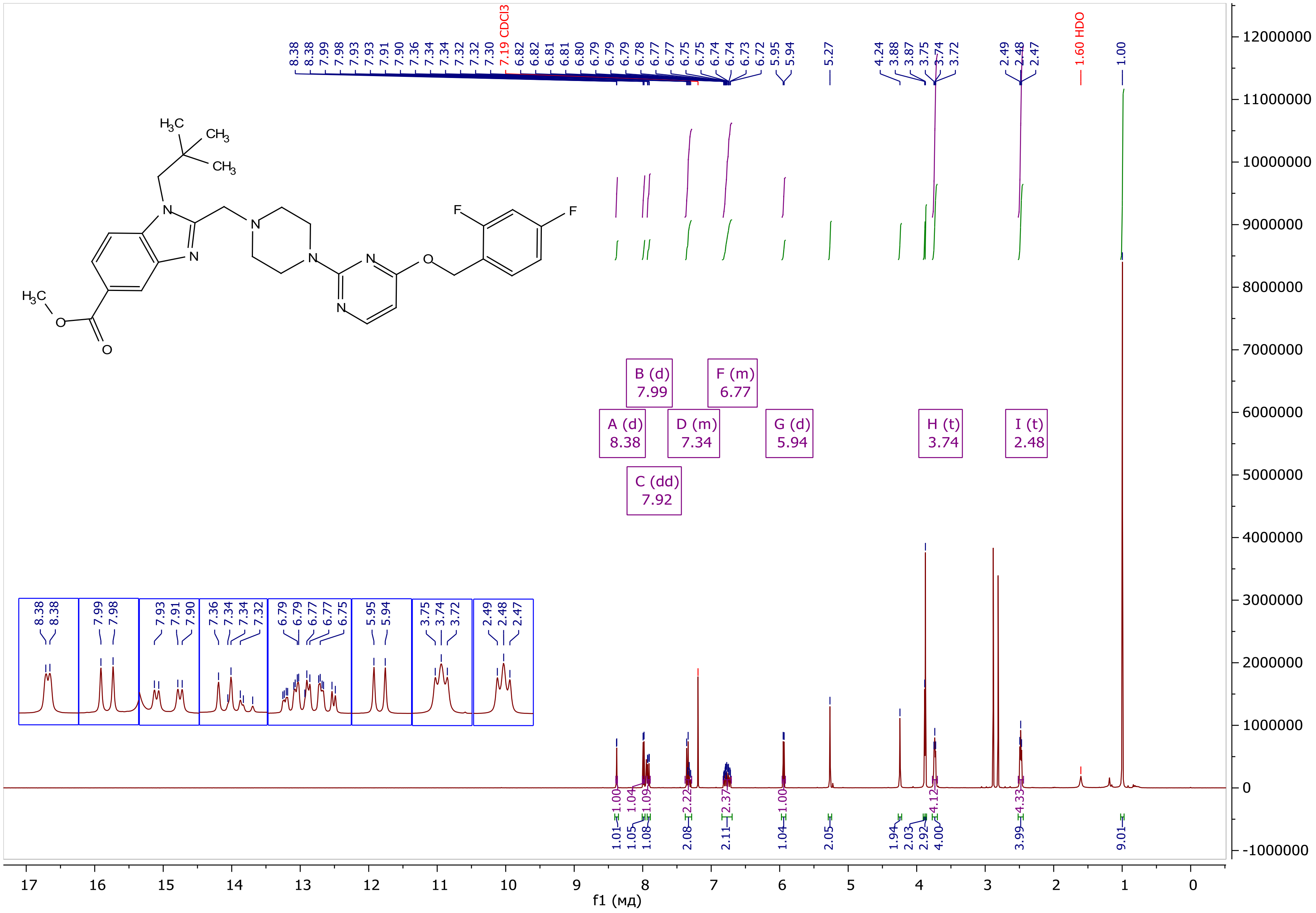

^{13}C NMR spectrum of compound **23u**

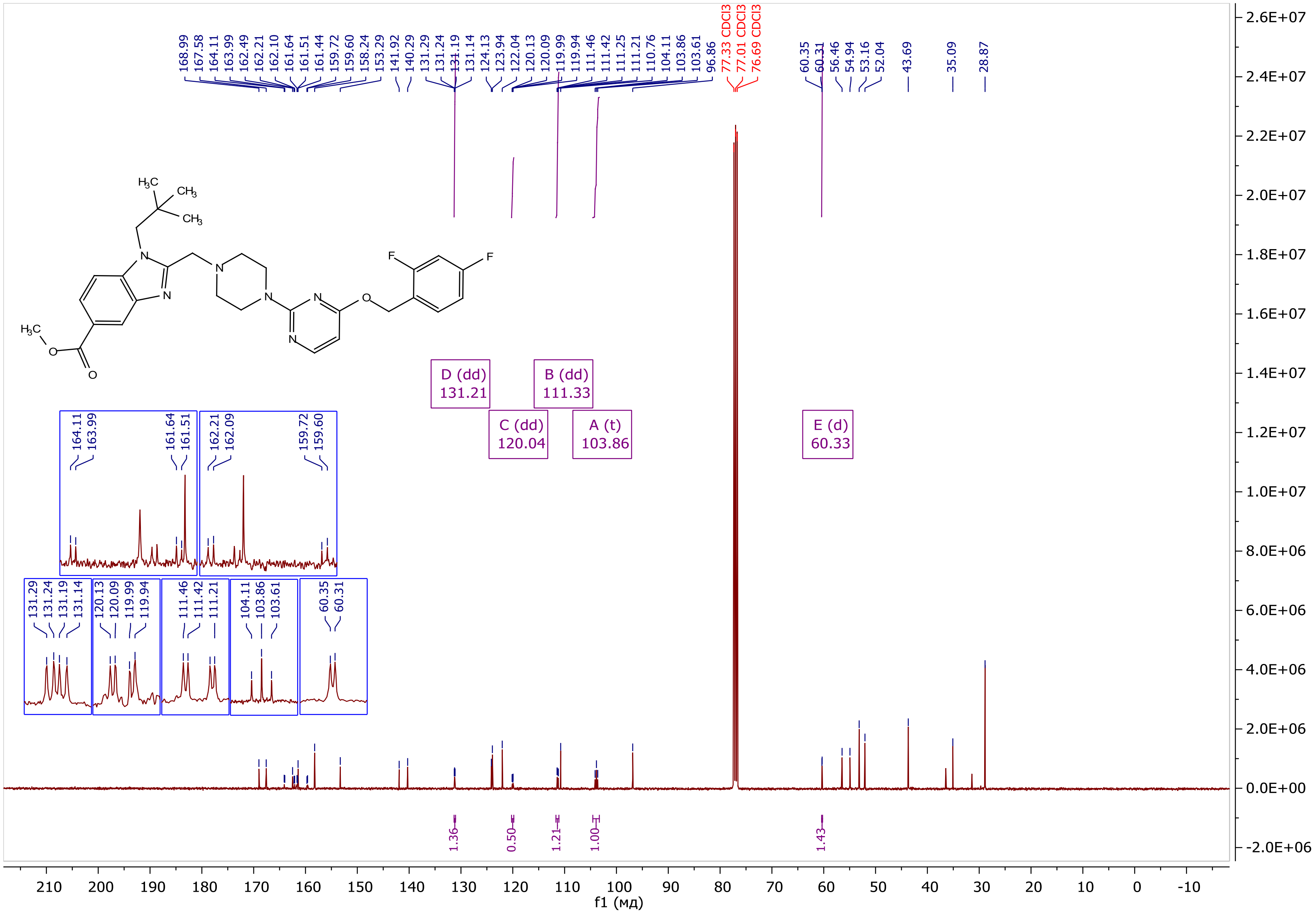

^{1}H NMR spectrum of compound **23w**

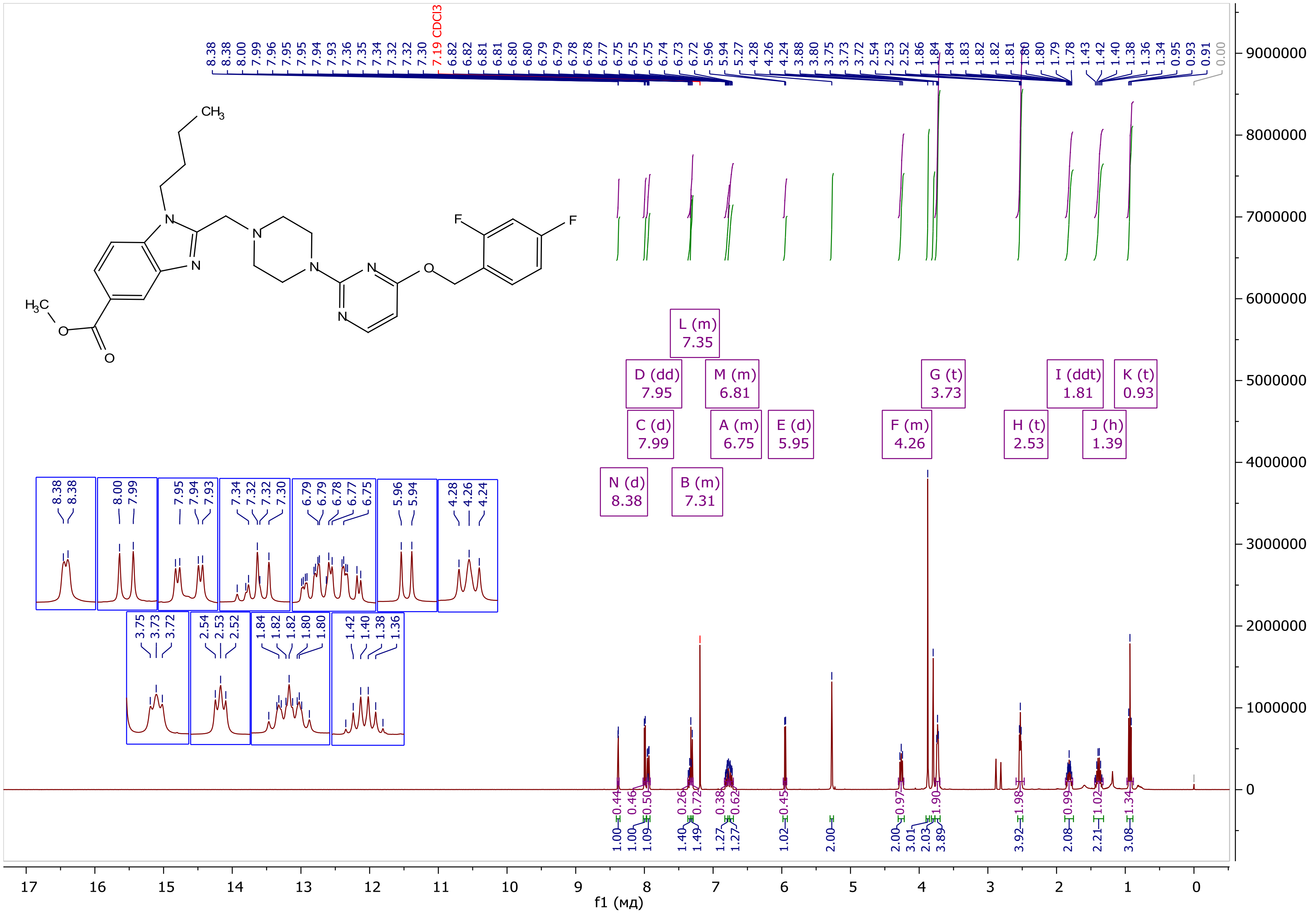

^{13}C NMR spectrum of compound **23w**

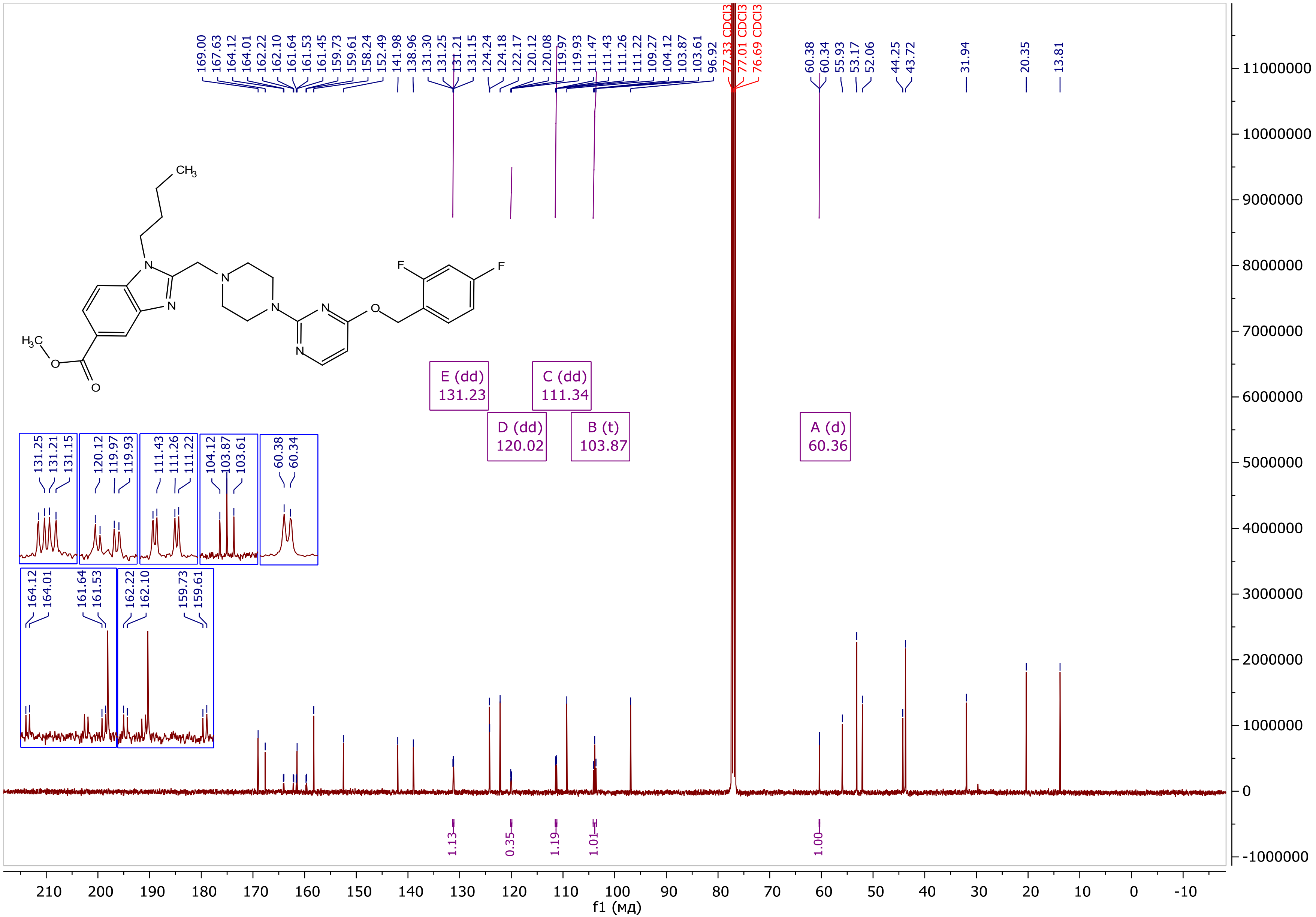

^{1}H NMR spectrum of compound **23x**

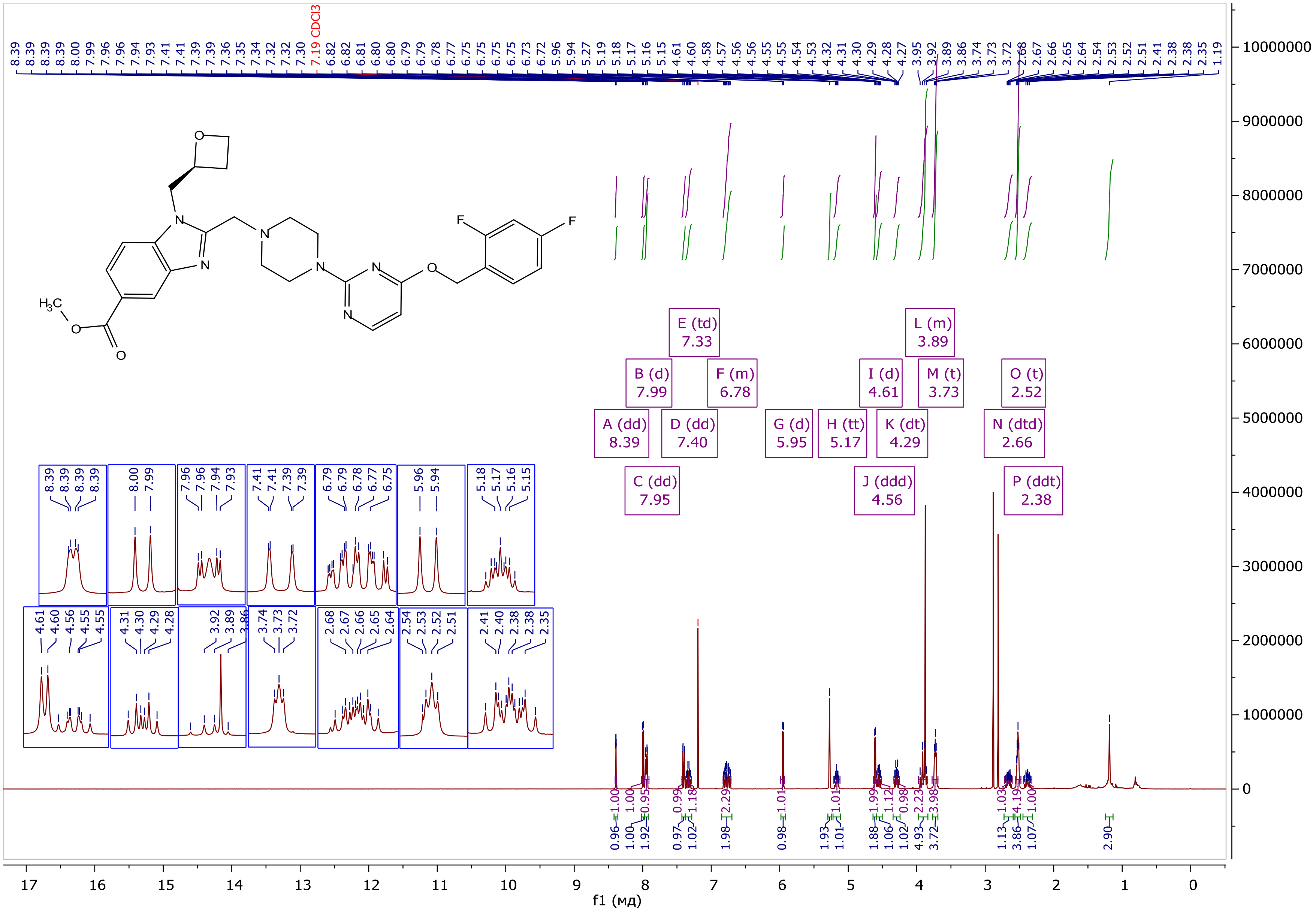

^{13}C NMR spectrum of compound **23x**

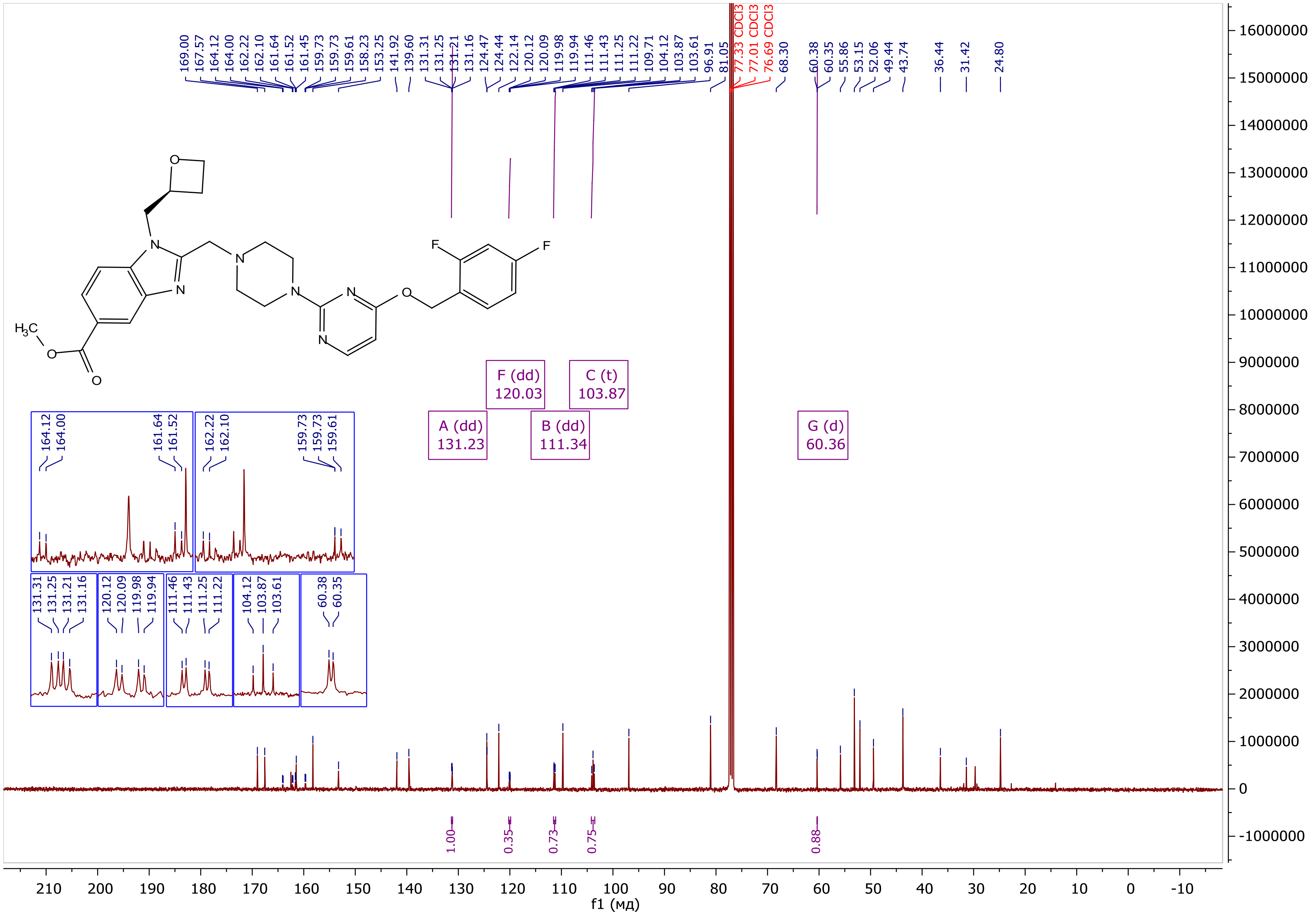

^{1}H NMR spectrum of compound **23z**

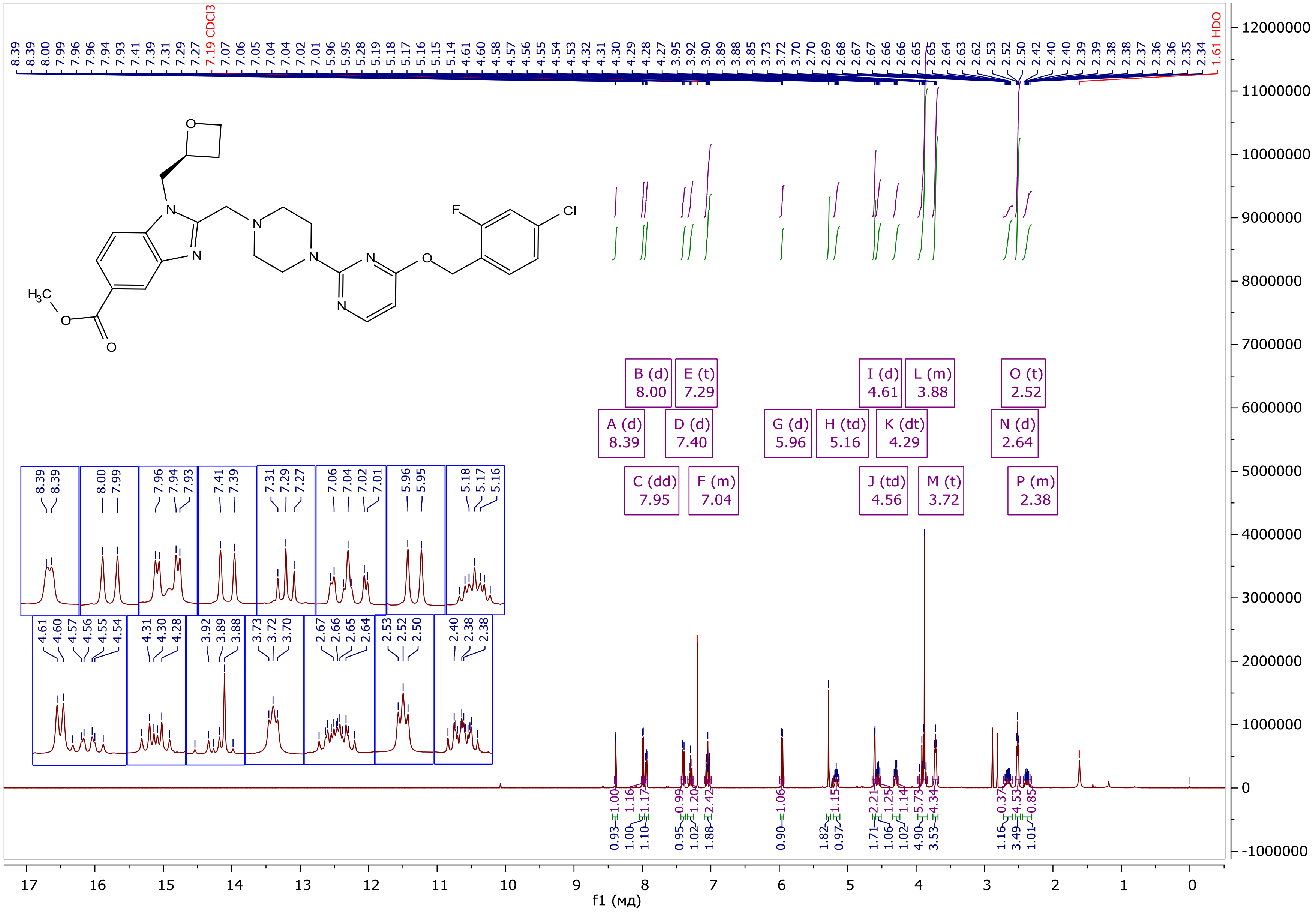

^{13}C NMR spectrum of compound **23z**

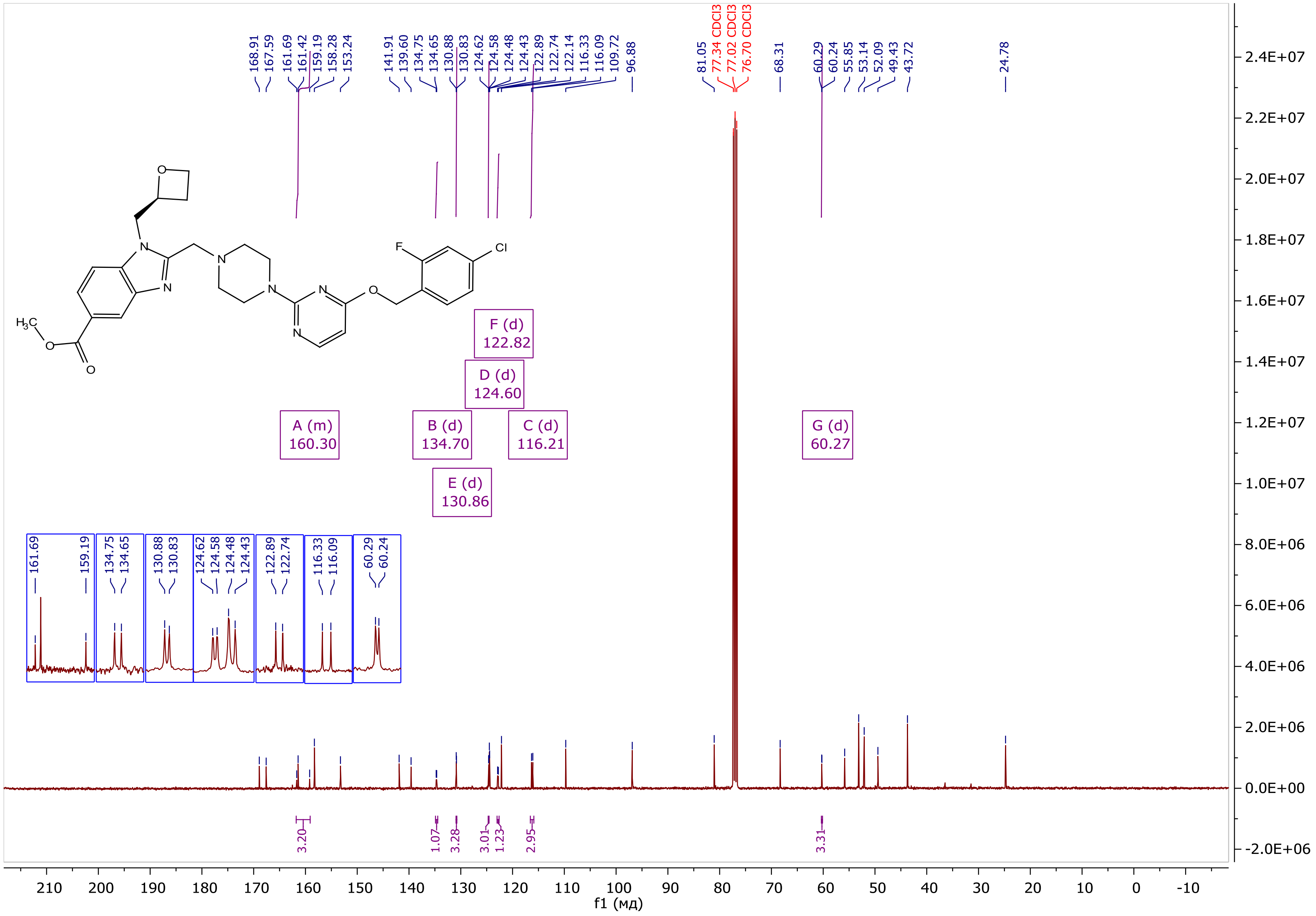

^{1}H NMR spectrum of compound **23aa**

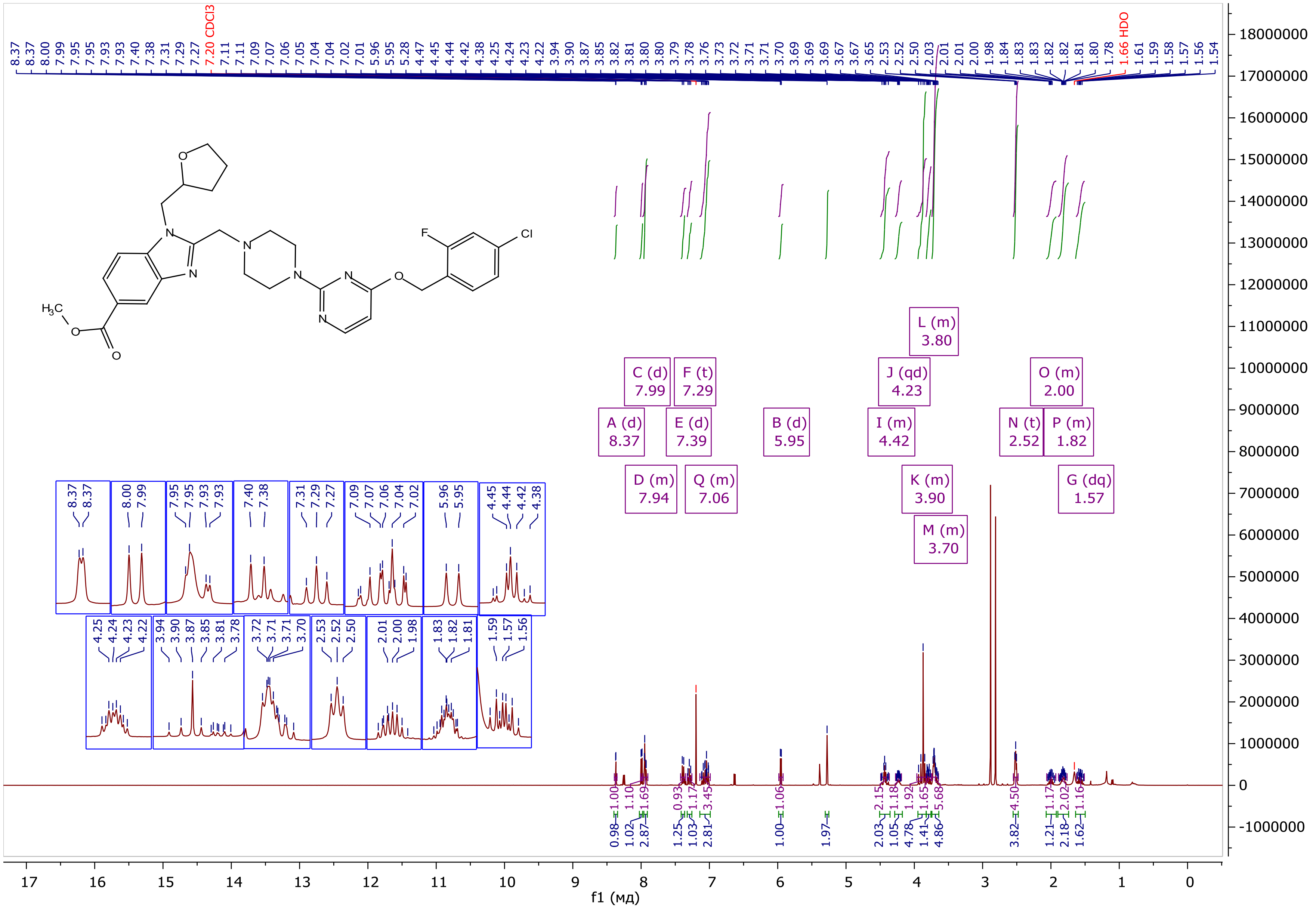

^{13}C NMR spectrum of compound **23aa**

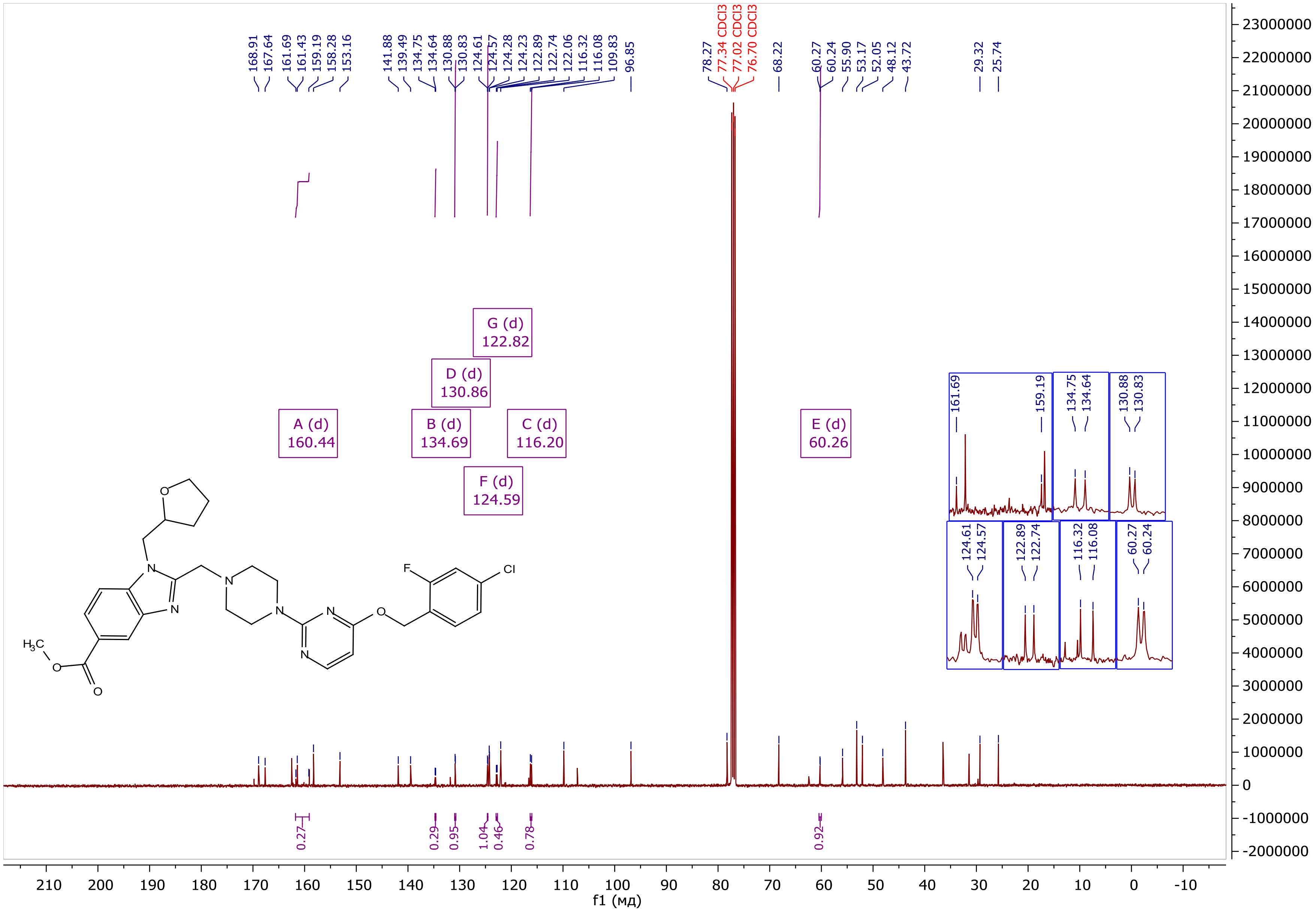

^{1}H NMR spectrum of compound **23r'**

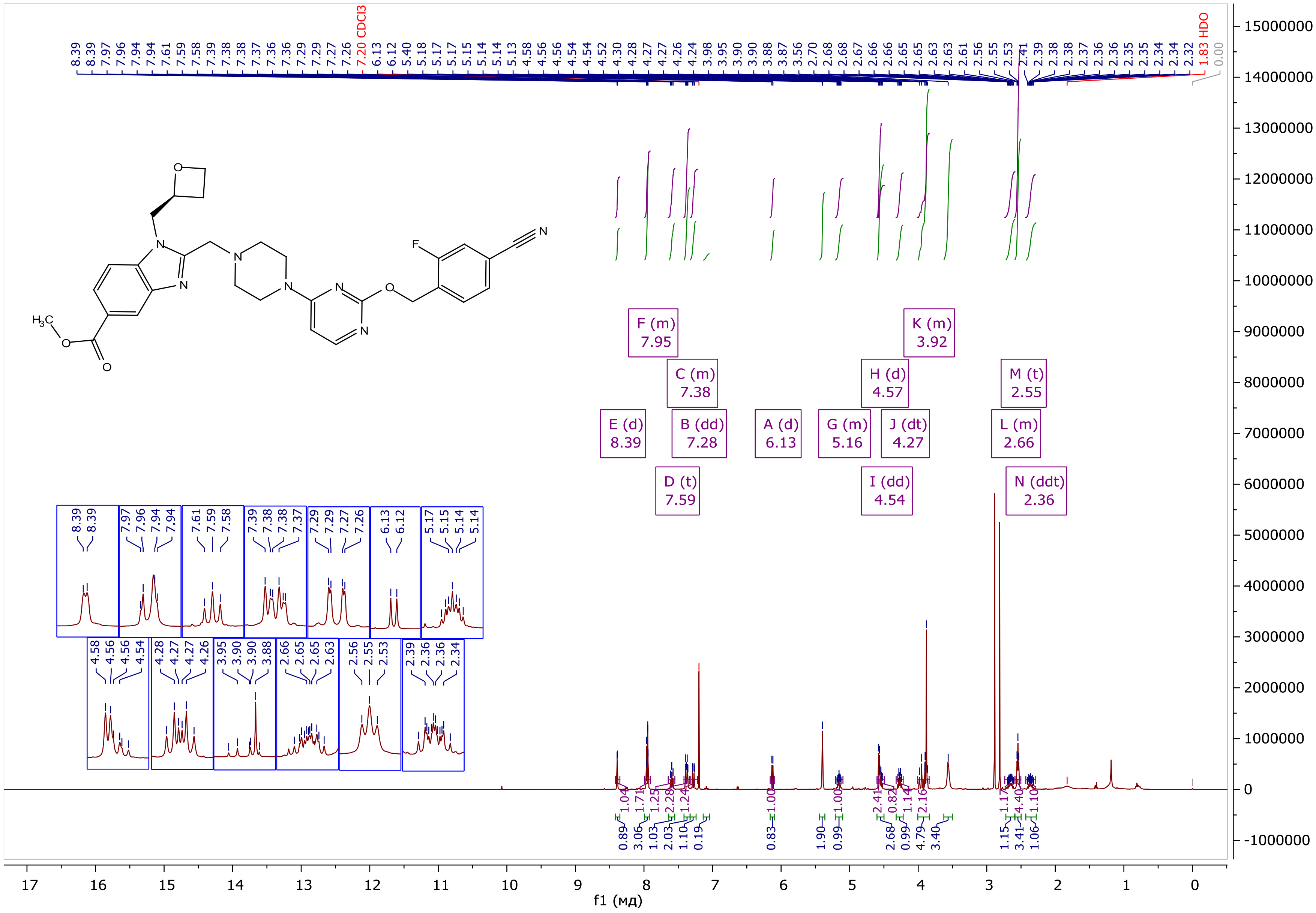

^{13}C NMR spectrum of compound **23r'**

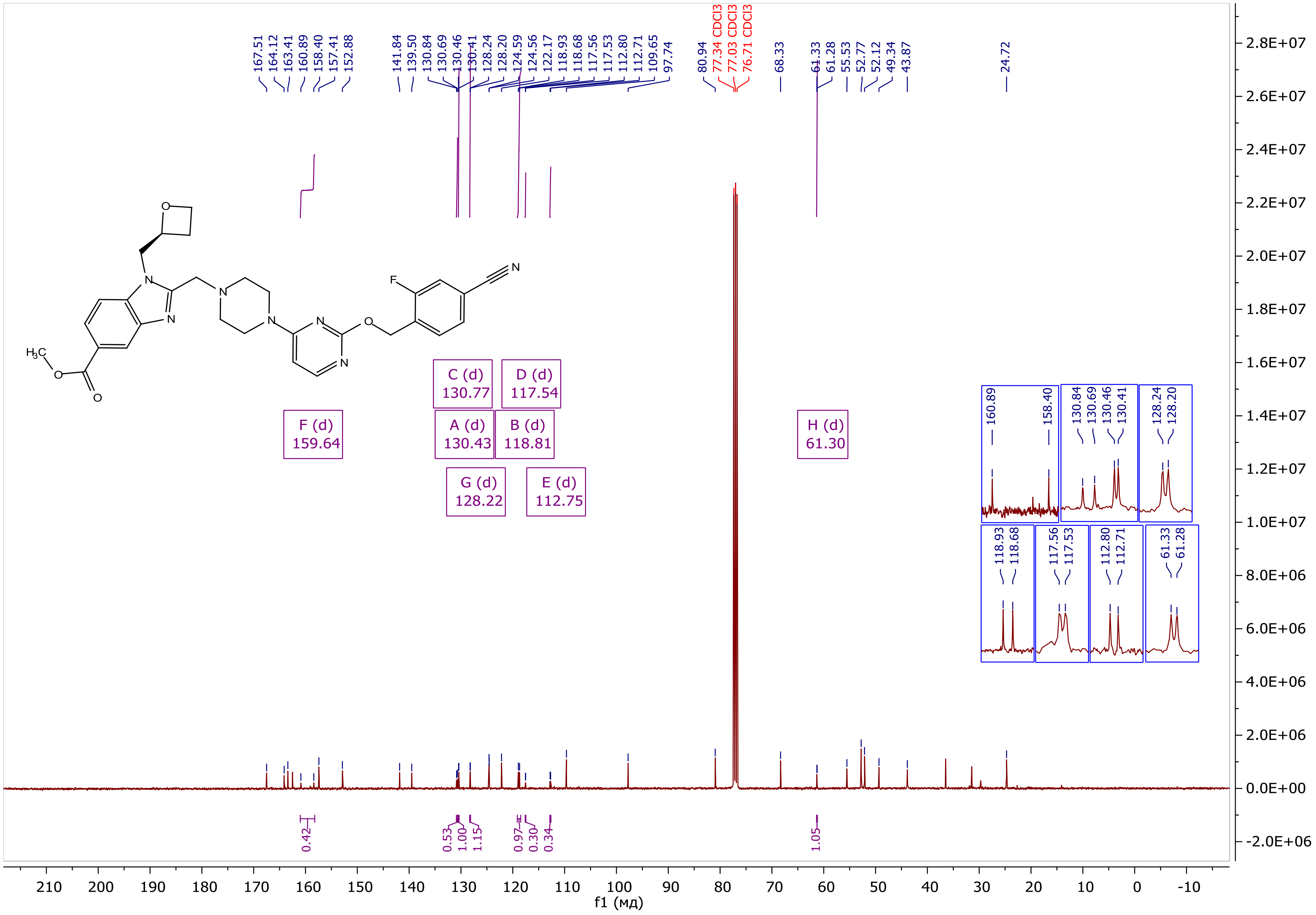

^{1}H NMR spectrum of compound **23s'**

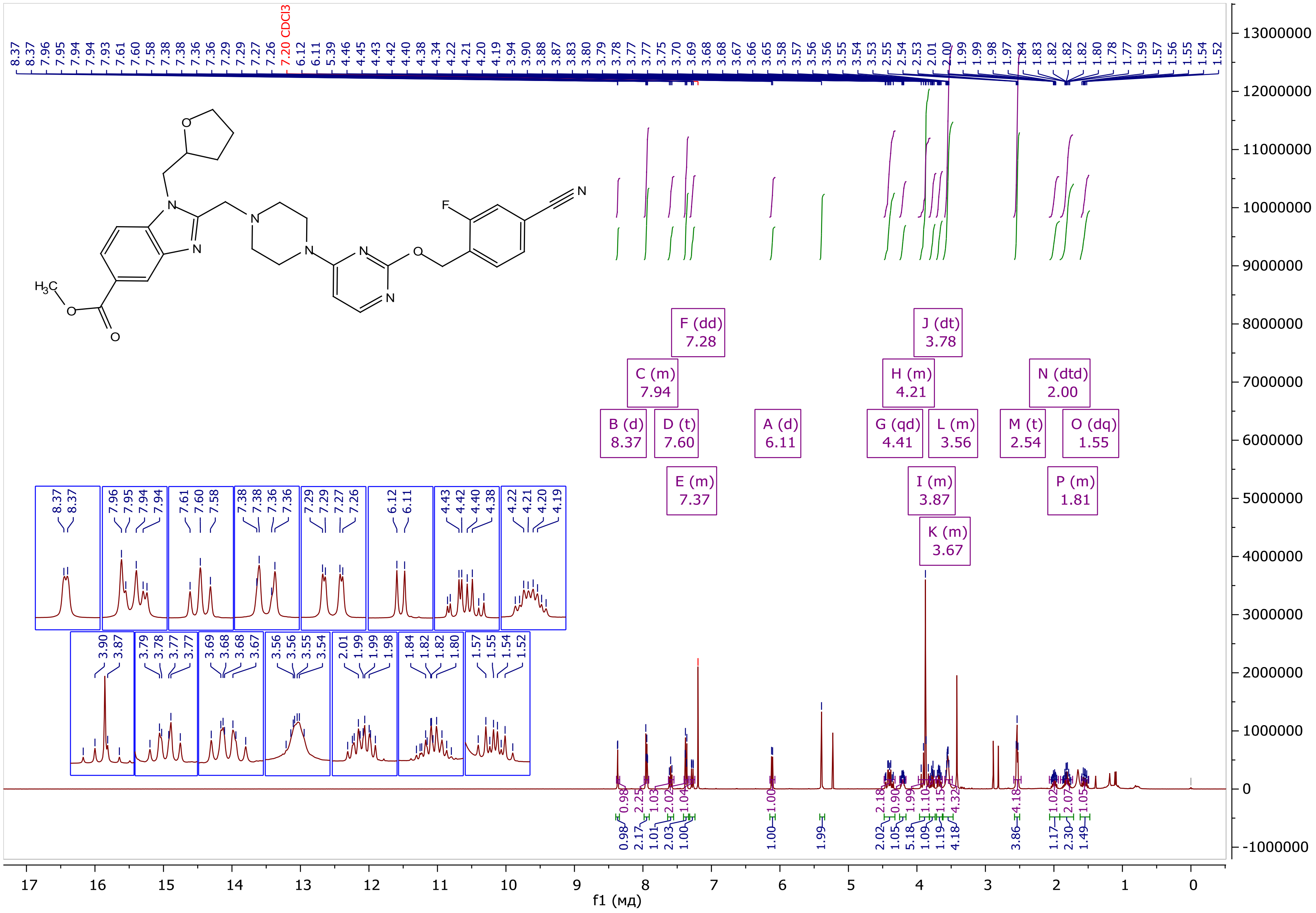

^{13}C NMR spectrum of compound **23s'**

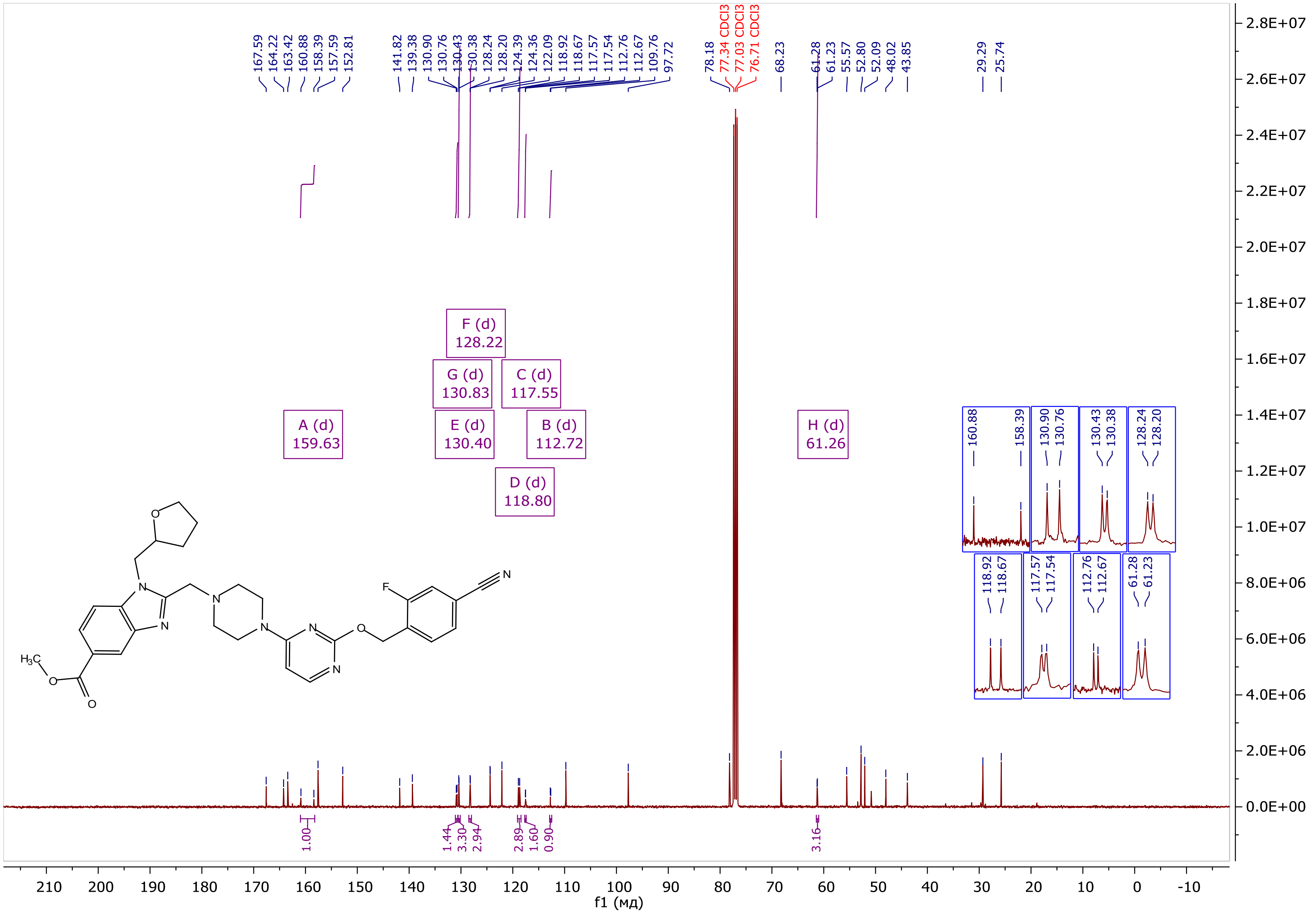

^{1}H NMR spectrum of compound **23z'**

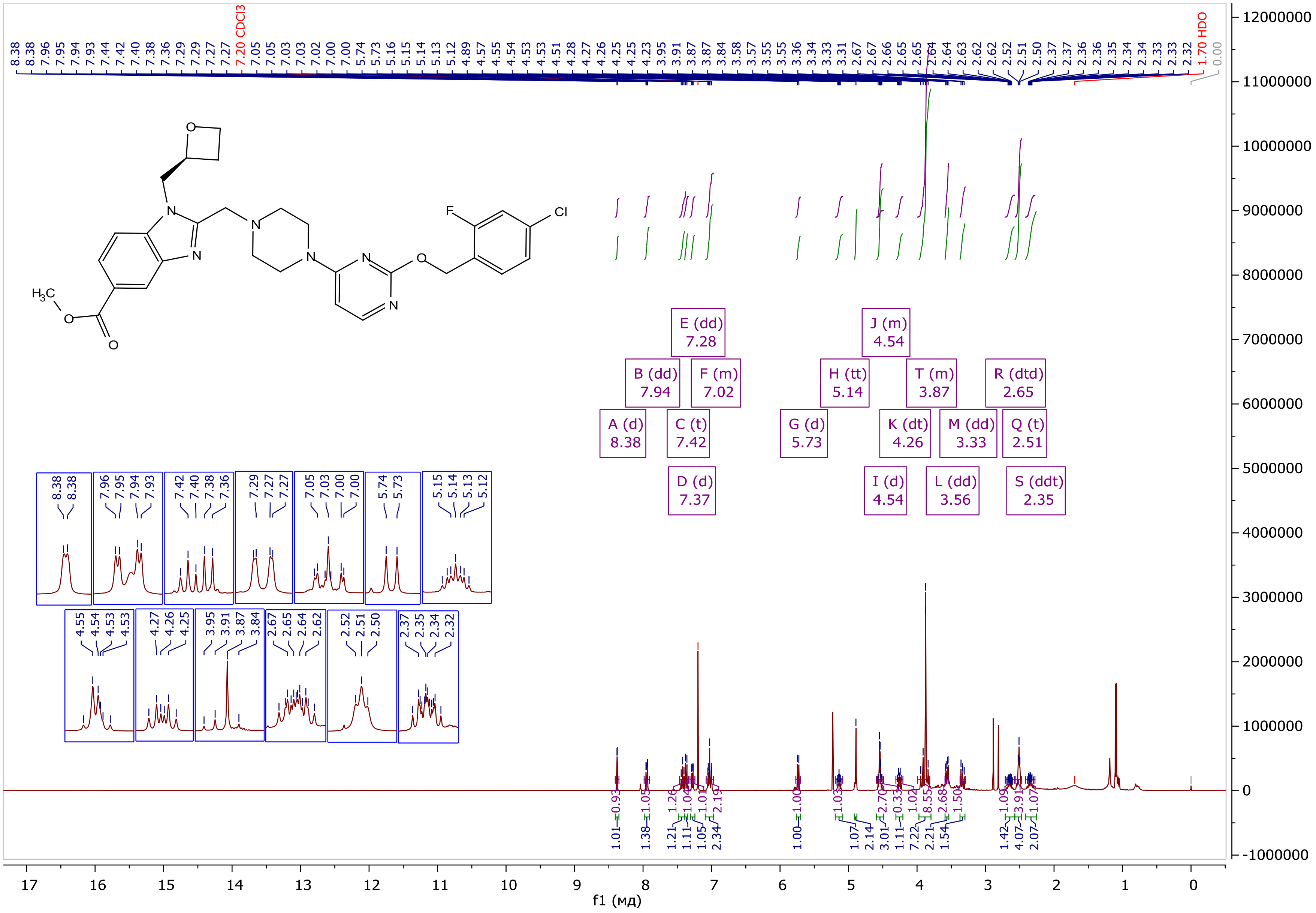

^{13}C NMR spectrum of compound **23z'**

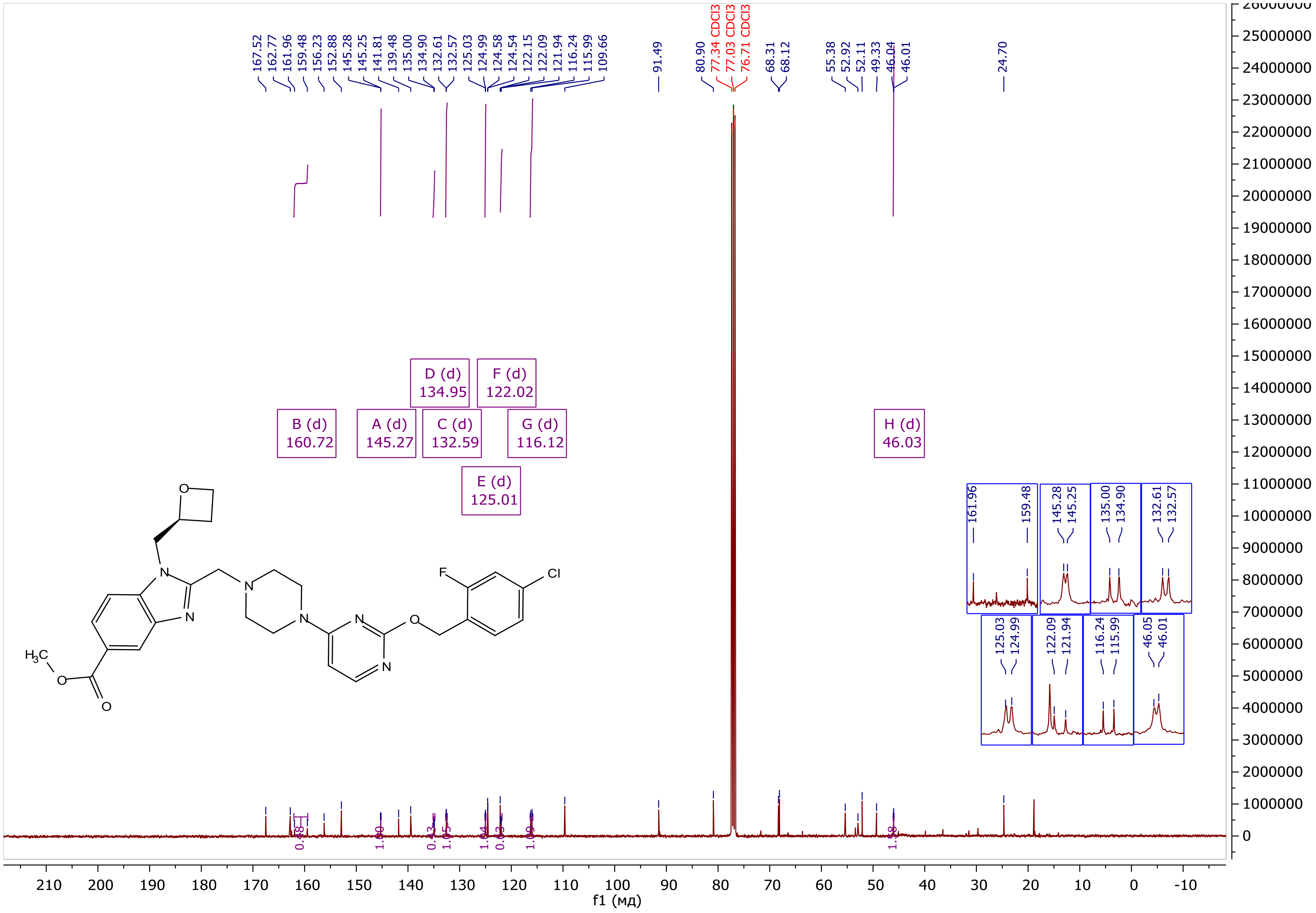

^{1}H NMR spectrum of compound **23aa'**

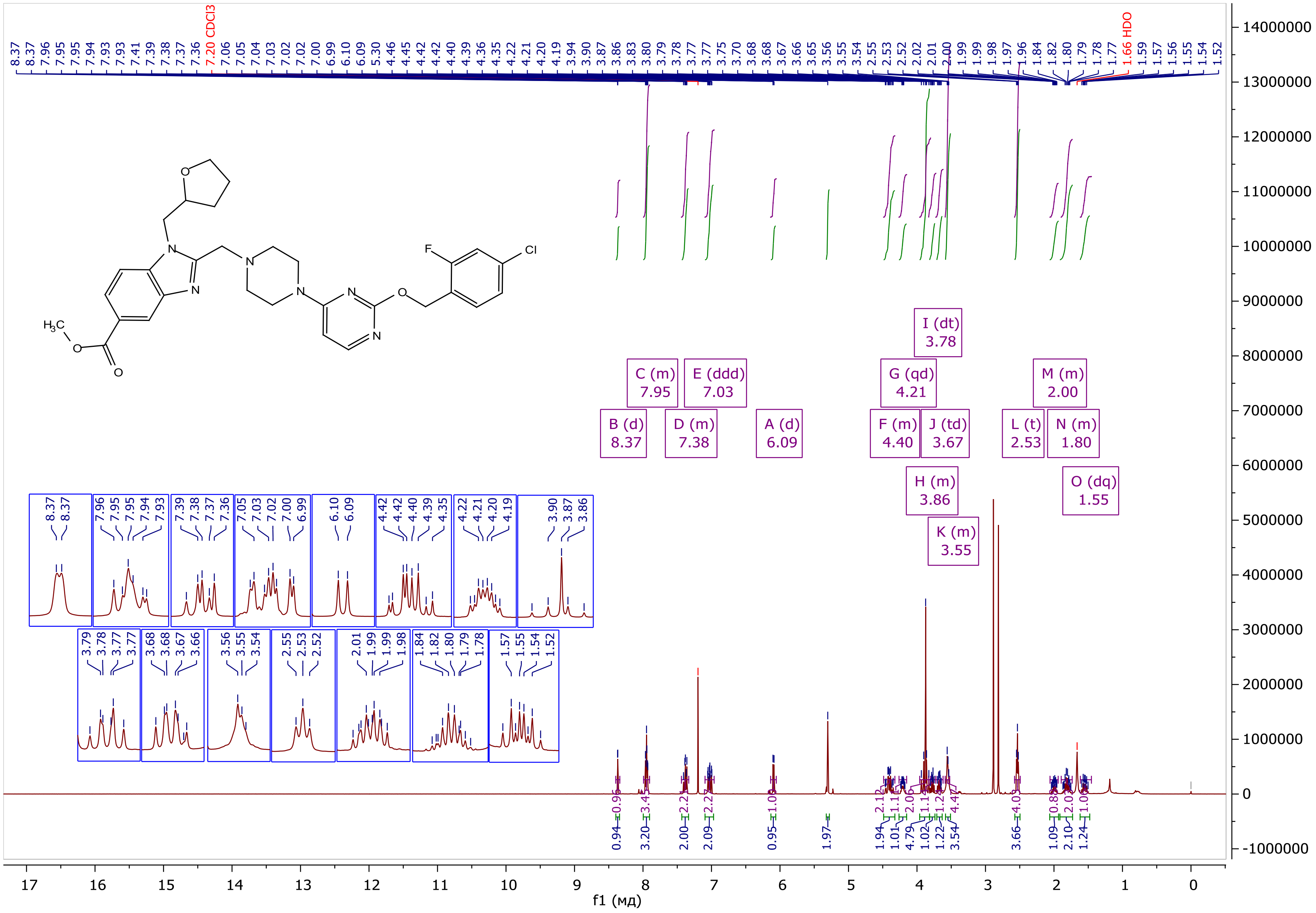

^{13}C NMR spectrum of compound **23aa'**

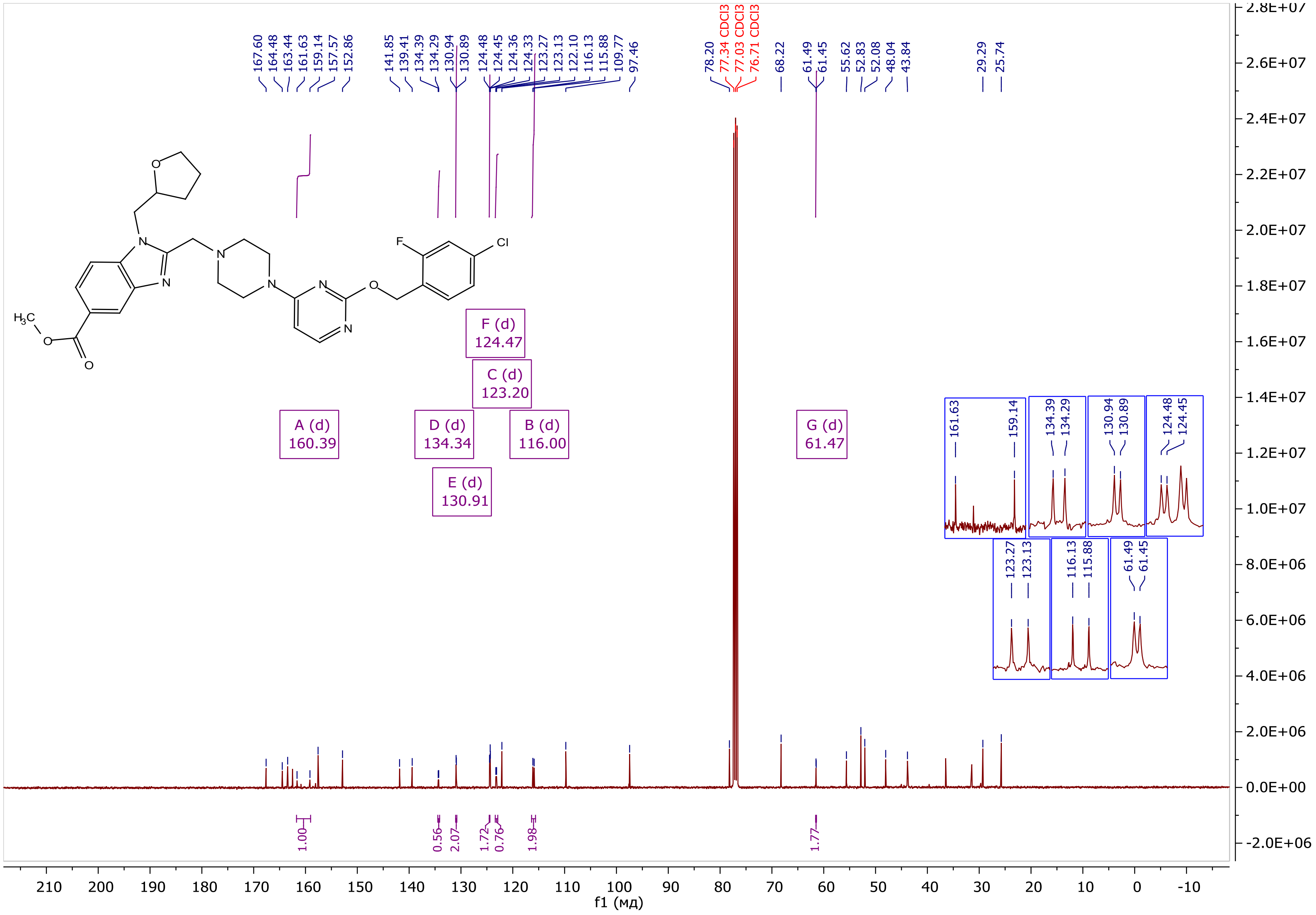

Target 1*H*-benzo[*d*]imidazole-5-carboxylic acid derivatives 12a-12aa, 12r', 12s', 12z', 12aa'

General procedures to synthesis of target 1H-benzo[d]imidazole-5-carboxylic acid derivatives **12a-12aa, 12r', 12s', 12z', 12aa'**.

Method A. A weighted portion of the corresponding methyl ester **23a-f**, **23i-k**, **23m** (1 eq) was dissolved in MeOH (5 ml) under vigorous stirring. An aqueous NaOH solution (1M, 10 ml) was added to the resulting ester solution, the reaction mixture was stirred at rt for 24 h, the reaction was monitored by TLC (DCM:MeOH:HCOOH = 95:5:0.1). After the reaction was completed, the mixture was treated with 1N aqueous citric acid solution to pH = 5, the target acids were extracted with DCM (3×20 ml). The combined organic layer was dried with anhydrous Na_2SO_4 under vigorous stirring for 1 h, the precipitate was filtered off, the solvent was evaporated to dryness, and the residue was purified by column chromatography on silica gel (DCM:MeOH = 9:1). Fractions containing the target product was collected, the solvent was evaporated to dryness to form the corresponding acid **12a-f**, **12i-k**, **12m**, which was lyophilized immediately before biological studies.

Method B. A weighted portion of corresponding methyl ester **23g-h**, **23l**, **23n-23aa, 23r', 23s', 23z', 23aa'** (1 eq) was dissolved in AcN (10 ml) in a round bottom flask under vigorous stirring. A 0.97M aqueous solution of 1,5,7-triazabicyclo[4.4.0]dec-5-ene (TBD, 4 eq) in deionized water was added to the resulting solution. The reaction mixture was stirred at rt for 24 h, the reaction was monitored by TLC (DCM:MeOH:HCOOH = 95:5:0.1). After the reaction was completed, the mixture was treated with 1N aqueous citric acid solution to pH = 5, the target acids were extracted with DCM (3×20 ml). The combined organic layer was dried with anhydrous Na_2SO_4 under vigorous stirring for 1 h, the precipitate was filtered off, the solvent was evaporated to dryness, and the residue was purified by column chromatography on silica gel (DCM:MeOH = 9:1). Fractions containing the target product was collected, the solvent was evaporated to dryness to form the corresponding acid **12g-h**, **12l**, **12n-12aa, 12r', 12s', 12z', 12aa'**, which was lyophilized immediately before biological studies.

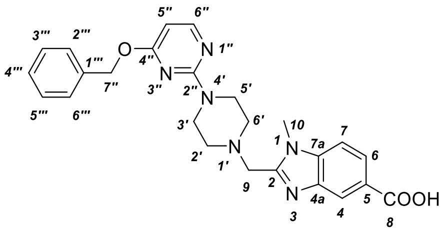

2-((4-(4-(Benzyloxy)pyrimidin-2-yl)piperazin-1-yl)methyl)-1-methyl-1H-benzo[d]imidazole-5-carboxylic acid **12a**, obtained according to **Method A**, white solid, 60 mg, 85%, mp 195-196°C. **^{1}H NMR (400 MHz, DMSO-*d$_6$*, *δ ppm*)**: 8.20 (1H, *s*, 4-CH), 8.09 (1H, *d*, *J* = 5.6 Hz, 6"-CH), 7.93-7.91 (1H, *d*, 6-CH), 7.54 (1H, *d*, *J* = 8.5 Hz, 7-CH), 7.44-7.30 (5H, *m*, 2'''-CH, 3'''-CH, 4'''-CH, 5'''-CH, 6'''-CH), 6.10 (1H, *d*, *J* = 5.6 Hz, 5"-CH), 5.34 (2H, *s*, 7"-CH_2), 3.90 (3H, *s*, 10-CH_3), 3.84 (2H, *s*, 9-CH_2), 3.73-3.71 (4H, *m*, 3'-CH_2, 5'-CH_2), 2.54-2.51 (4H, *m*, 2'-CH_2, 6'-CH_2). **^{13}C NMR (101 MHz, DMSO-*d$_6$*, *δ ppm*)**: 169.2 (C-4"), 161.5 (C-2"), 159.3 (C-8), 159.1 (C-6"), 153.1 (C-2), 141.8 (C-4a), 139.7 (C-7a), 137.3 (C-1'''), 128.8 (C-3''', C-5'''), 128.6 (C-2''', C-6'''), 128.4 (C-4'''), 128.0 (C-5), 124.9 (C-7), 120.9 (C-4), 109.6 (C-6), 96.8 (C-5"), 67.2 (C-7"), 54.9 (C-9), 52.9 (C-2', C-6'), 43.9 (C-3', C-5'), 30.7 (C-10). **HRMS (ESI+)**: found *m/z* 459.2132 $[M + H]^+$; calculated $C_{25}H_{27}N_6O_3^+$ 459.2066.

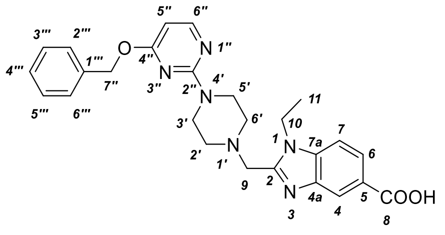

2-((4-(4-(Benzyloxy)pyrimidin-2-yl)piperazin-1-yl)methyl)-1-ethyl-1H-benzo[d]imidazole-5-carboxylic acid **12b**, obtained according to **Method A**, white solid, 70 mg, 90%, mp 204-205°C. **^{1}H NMR (400 MHz, DMSO-*d$_6$*, *δ ppm*)**: 8.22 (1H, *s*, 4-CH), 8.09 (1H, *d*, *J* = 5.6 Hz, 6"-CH), 7.93-7.91 (1H, *m*, 6-CH), 7.58 (1H, *d*, *J* = 8.6 Hz, 7-CH), 7.44-7.30 (5H, *m*, 2'''-CH, 3'''-CH, 4'''-CH, 5'''-CH, 6'''-CH), 6.11 (1H, *d*, *J* = 5.6 Hz, 5"-CH), 5.34 (2H, *s*, 7"-CH_2), 4.39 (2H, *q*, *J* = 7.0 Hz, 10-CH_2), 3.84 (2H, *s*, 9-CH_2), 3.72-3.70 (4H, *m*, 3'-CH_2, 5'-CH_2), 2.54-2.51 (4H, *m*, 2'-CH_2, 6'-CH_2), 1.41 (3H, *t*, *J* = 7.0 Hz, 11-CH_3). **^{13}C NMR (101 MHz, DMSO-*d$_6$*, *δ ppm*)**: 169.2 (C-4"), 161.5 (C-2"), 159.6 (C-8), 159.1 (C-6"), 153.1 (C-2), 142.1 (C-4a), 140.7 (C-7a), 137.3 (C-1'''), 128.8 (C-3''', C-5'''), 128.5 (C-2''', C-6'''), 128.4 (C-4'''), 128.3 (C-5), 124.2 (C-4), 121.1 (C-6), 109.8 (C-7), 96.9 (C-5"), 67.2 (C-7"), 39.1 (C-10), 55.1 (C-9), 53.0 (C-2', C-6'), 43.9 (C-3', C-5'), 15.4 (C-11). **HRMS (ESI+)**: found *m/z* 473.2286 $[M + H]^+$; calculated $C_{26}H_{29}N_6O_3^+$ 473.2223.

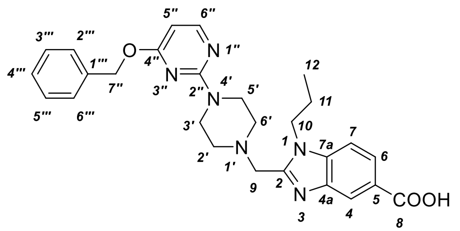

2-((4-(4-(Benzyloxy)pyrimidin-2-yl)piperazin-1-yl)methyl)-1-propyl-1H-benzo[d]imidazole-5-carboxylic acid **12c**, obtained according to **Method A**, white solid, 90 mg, 85%, mp 159-160°C. **^{1}H NMR** (**400 MHz, DMSO-*d₆*, *δ ppm***): 8.19 (1H, *s*, 4-CH), 8.10 (1H, *d*, *J* = 5.6 Hz, 6''-CH), 7.87 (1H, *dd*, *J* = 8.5, 1.5 Hz, 6-CH), 7.66 (1H, *d*, *J* = 8.5 Hz, 7-CH), 7.67-7.30 (5H, *m*, 2'''-CH, 3'''-CH, 4'''-CH, 5'''-CH, 6'''-CH), 6.11 (1H, *d*, *J* = 5.6 Hz, 5''-CH), 5.34 (2H, *s*, 7''-CH_2), 4.32 (2H, *t*, *J* = 7.5 Hz, 10-CH_2), 3.85 (2H, *s*, 9-CH_2), 3.74-3.70 (4H, *m*, 3'-CH_2, 5'-CH_2), 2.54-2.51 (4H, *m*, 2'-CH_2, 6'-CH_2), 1.86 (2H, *h*, *J* = 7.6 Hz, 11-CH_2), 0.95 (3H, *t*, *J* = 7.3 Hz, 12-CH_3). **^{13}C NMR** (**101 MHz, DMSO-*d₆*, *δ ppm***): 169.2 (C-4''), 161.5 (C-2''), 159.7 (C-8), 159.1 (C-6''), 153.4 (C-2), 142.0 (C-4a), 139.1 (C-7a), 137.3 (C-1'''), 128.8 (C-3''', C-5'''), 128.6 (C-2''', C-6'''), 128.4 (C-4'''), 128.2 (C-5), 124.0 (C-4), 121.2 (C-6), 110.6 (C-7), 96.9 (C-5''), 67.2 (C-7''), 55.1 (C-9), 52.9 (C-2', C-6'), 45.7 (C-10), 43.9 (C-3', C-5'), 23.2 (C-11), 11.7 (C-12). **HRMS** (**ESI+**): found *m/z* 487.2448 $[M + H]^+$; calculated $C_{27}H_{31}N_6O_3^+$ 487.2379.

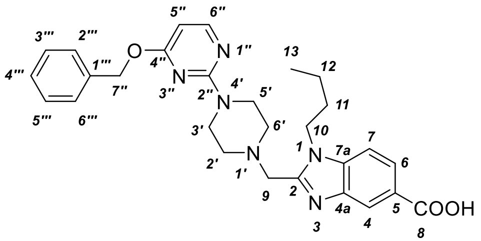

2-((4-(4-(Benzyloxy)pyrimidin-2-yl)piperazin-1-yl)methyl)-1-butyl-1H-benzo[d]imidazole-5-carboxylic acid **12d**, obtained according to **Method A**, white solid, 85 mg, 90%, mp 115-116°C. **^{1}H NMR** (**400 MHz, DMSO-*d₆*, *δ ppm***): 8.19 (1H, *s*, 4-CH), 8.10 (1H, *d*, *J* = 5.6 Hz, 6''-CH), 7.88 (1H, *d*, *J* = 8.4 Hz, 6-CH), 7.64 (1H, *d*, *J* = 8.5 Hz, 7-CH), 7.43-7.30 (5H, *m*, 2'''-CH, 3'''-CH, 4'''-CH, 5'''-CH, 6'''-CH), 6.11 (1H, *d*, *J* = 5.6 Hz, 5''-CH), 5.34 (2H, *s*, 7''-CH_2), 4.35 (2H, *t*, 10-CH_2), 3.85 (2H, *s*, 9-CH_2), 3.70 (4H, *d*, 3'-CH_2, 5'-CH_2), 2.54-2.51 (4H, *m*, 2'-CH_2, 6'-CH_2), 1.86-1.79 (2H, *m*, 11-CH_2), 1.45-1.37 (2H, *m*, 12-CH_2), 0.94 (3H, *t*, *J* = 7.4 Hz, 13-CH_3). **^{13}C NMR** (**101 MHz, DMSO-*d₆*, *δ ppm***): 169.2 (C-4''), 161.5 (C-2''), 159.6 (C-8), 159.1 (C-6''), 153.3 (C-2), 142.0 (C-4a), 139.0 (C-7a), 137.3 (C-1'''), 128.8 (C-3''', C-5'''), 128.6 (C-2''', C-6'''), 128.4 (C-4'''), 128.0 (C-5), 124.0 (C-4), 121.1 (C-6), 110.5 (C-7), 96.9 (C-5''), 67.2 (C-7''), 55.1 (C-9), 53.0 (C-2', C-6'), 44.1 (C-10), 43.9 (C-3', C-5'), 31.9 (C-11), 20.1 (C-12), 14.2 (C-13). **HRMS** (**ESI+**): found *m/z* 501.2600 $[M + H]^+$; calculated $C_{28}H_{33}N_6O_3^+$ 501.2536.

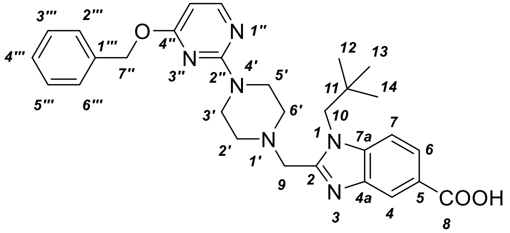

2-((4-(4-(Benzyloxy)pyrimidin-2-yl)piperazin-1-yl)methyl)-1-neopentyl-1H-benzo[d]imidazole-5-carboxylic acid **12e**, obtained according to **Method A**, white solid, 70 mg, 85%, mp 154-155°C. **^{1}H NMR** (**400 MHz, DMSO-*d₆*, *δ ppm***): 8.24 (1H, *d*, *J* = 1.6 Hz, 4-CH), 8.14 (1H, *d*, *J* = 5.6 Hz, 6''-CH), 7.90 (1H, *dd*, *J* = 8.6, 1.6 Hz, 6-CH), 7.74 (1H, *d*, *J* = 8.6 Hz, 7-CH), 7.49-7.34 (5H, *m*, 2'''-CH, 3'''-CH, 4'''-CH, 5'''-CH, 6'''-CH), 6.15 (1H, *d*, *J* = 5.6 Hz, 5''-CH), 5.39 (2H, *s*, 7''-CH_2), 4.39 (2H, *s*, 10-CH_2), 3.95 (2H, *s*, 9-CH_2), 3.78-3.76 (4H, *m*, 3'-CH_2, 5'-CH_2), 2.54-2.51 (4H, *m*, 2'-CH_2, 6'-CH_2), 1.05 (9H, *s*, 12-CH_3, 13-CH_3, 14-CH_3). **^{13}C NMR** (**101 MHz, DMSO-*d₆*, *δ ppm***): 169.2 (C-4''), 161.5 (C-2''), 159.2 (C-8), 159.1 (C-6''), 153.8 (C-2), 141.9 (C-4a), 140.2 (C-7a), 137.3 (C-1'''), 128.8 (C-3''', C-5'''), 128.5 (C-2''', C-6'''), 128.3 (C-4'''), 126.9 (C-5), 123.7 (C-4), 121.0 (C-6), 112.0 (C-7), 96.8 (C-5''), 67.2 (C-7''), 55.5 (C-9), 54.3 (C-10), 52.9 (C-2', C-6'), 43.9 (C-3', C-5'), 35.1 (C-11), 28.7 (C-12, C-13, C-14). **HRMS** (**ESI+**): found *m/z* 515.2759 $[M + H]^+$; calculated $C_{29}H_{35}N_6O_3^+$ 515.2692.

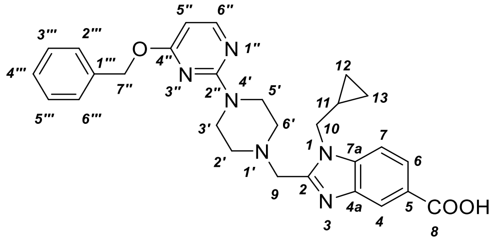

2-((4-(4-(Benzyloxy)pyrimidin-2-yl)piperazin-1-yl)methyl)-1-(cyclopropylmethyl)-1H-benzo[d]imidazole-5-carboxylic acid **12f**, obtained according to **Method A**, white solid, 85 mg, 90%, mp 172-173°C. **^{1}H NMR** (**400 MHz, DMSO-*d₆*, *δ ppm***): 8.14 (1H, *s*, 4-CH), 8.10 (1H, *d*, *J* = 5.6 Hz, 6''-CH), 7.86 (1H, *d*, *J* = 8.5 Hz, 6-CH), 7.56 (1H, *d*, *J* = 8.4 Hz, 7-CH), 7.45-7.28 (5H, *m*, 2'''-CH, 3'''-CH, 4'''-CH, 5'''-CH, 6'''-CH), 6.11 (1H, *d*, *J* = 5.6 Hz, 5''-CH), 5.34 (2H, *s*, 7''-CH_2), 4.26 (2H, *d*, *J* = 7.0 Hz, 10-CH_2), 3.83 (2H, *s*, 9-CH_2), 3.73-3.71 (4H, *m*, 3'-CH_2, 5'-CH_2), 2.53-2.51 (4H, *m*, 2'-CH_2, 6'-CH_2), 1.43 (1H, *p*, 11-CH), 0.57-0.54 (2H, *m*, 12-CH_2, 13-CH_2), 0.44-0.39 (2H, *m*, 12-CH_2, 13-CH_2). **^{13}C NMR** (**101 MHz, DMSO-*d₆*, *δ ppm***): 169.3 (C-4''), 167.6 (C-8), 161.5 (C-2''), 158.1 (C-6''), 152.4 (C-2), 142.0 (C-4a), 139.2 (C-7a), 136.8 (C-1'''), 128.5 (C-3''', C-5'''), 128.0 (C-4'''), 127.9 (C-2''', C-6'''), 124.3 (C-6), 124.2 (C-5), 122.1 (C-4), 109.6 (C-7), 97.1 (C-5''), 67.3 (C-7''), 55.9 (C-10), 53.2 (C-2', C-6'), 52.1 (C-9), 43.7 (C-3', C-5'), 11.2 (C-11), 4.4 (C-12, C-13). **HRMS** (**ESI+**): found *m/z* 499.2446 $[M + H]^+$; calculated $C_{28}H_{31}N_6O_3^+$ 499.2379

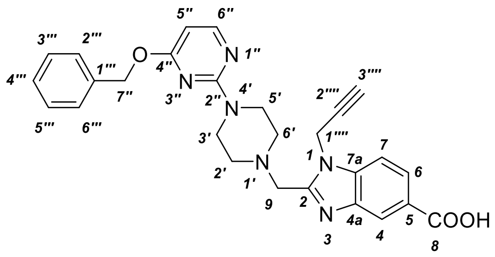

2-((4-(4-(Benzyloxy)pyrimidin-2-yl)piperazin-1-yl)methyl)-1-(prop-2-yn-1-yl)-1H-benzo[d]imidazole-5-carboxylic acid **12g**, obtained according to **Method B**, white solid, 80 mg, 75%, mp 183-184°C. **^{1}H NMR (400 MHz, DMSO-*d*$_6$, *δ ppm*)**: 8.12 (1H, *s*, 4-CH), 8.11 (1H, *d*, *J* = 5.6 Hz, 6''-CH), 7.88 (1H, *d*, *J* = 8.6 Hz, 6-CH), 7.55 (1H, *d*, *J* = 8.5 Hz, 7-CH), 7.45-7.28 (5H, *m*, 2'''-CH, 3'''-CH, 4'''-CH, 5'''-CH, 6'''-CH), 6.11 (1H, *d*, *J* = 5.6 Hz, 5''-CH), 5.34 (2H, *s*, 7''-CH_2), 5.25 (2H, *d*, *J* = 2.8 Hz, 1''''-CH_2), 3.83 (2H, *s*, 9-CH_2), 3.73-3.71 (4H, *m*, 3'-CH_2, 5'-CH_2), 2.53-2.51 (4H, *m*, 2'-CH_2, 6'-CH_2), 2.30 (1H, *t*, *J* = 2.4 Hz, 3''''-CH). **^{13}C NMR (101 MHz, DMSO-*d*$_6$, *δ ppm*)**: 169.6 (C-4''), 167.3 (C-8), 161.6 (C-2''), 158.2 (C-6''), 152.4 (C-2), 142.1 (C-4a), 139.4 (C-7a), 136.6 (C-1'''), 128.5 (C-3''', C-5'''), 128.1 (C-4'''), 127.8 (C-2''', C-6'''), 124.2 (C-6), 124.0 (C-5), 122.1 (C-4), 109.2 (C-7), 97.0 (C-5''), 76.5 (C-2''''), 73.1 (C-3''''), 67.2 (C-7''), 53.2 (C-2', C-6'), 52.1 (C-9), 43.7 (C-3', C-5'), 34.6 (C-1''''). **HRMS** (**ESI+**): found *m/z* 483.2132 $[M + H]^+$; calculated $C_{27}H_{27}N_6O_3^+$ 483.2066

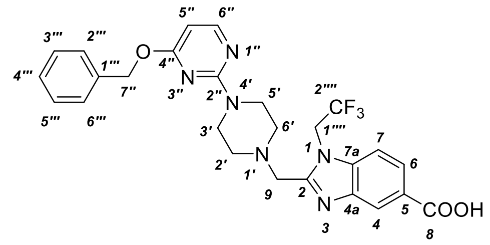

2-((4-(4-(Benzyloxy)pyrimidin-2-yl)piperazin-1-yl)methyl)-1-(2,2,2-trifluoroethyl)-1H-benzo[d]imidazole-5-carboxylic acid **12h**, obtained according to **Method B**, white solid, 75 mg, 80%, mp 184-185°C. **^{1}H NMR (400 MHz, DMSO-*d*$_6$, *δ ppm*)**: 8.15 (1H, *s*, 4-CH), 8.12 (1H, *d*, *J* = 5.6 Hz, 6''-CH), 7.90 (1H, *d*, *J* = 8.6 Hz, 6-CH), 7.53 (1H, *d*, *J* = 8.5 Hz, 7-CH), 7.44-7.27 (5H, *m*, 2'''-CH, 3'''-CH, 4'''-CH, 5'''-CH, 6'''-CH), 6.10 (1H, *d*, *J* = 5.6 Hz, 5''-CH), 5.30 (2H, *s*, 7''-CH_2), 5.22 (2H, *q*, *J* = 8.4 Hz, 1''''-CH_2), 3.86 (2H, *s*, 9-CH_2), 3.72-3.69 (4H, *m*, 3'-CH_2, 5'-CH_2), 2.54-2.52 (4H, *m*, 2'-CH_2, 6'-CH_2). **^{13}C NMR (101 MHz, DMSO-*d*$_6$, *δ ppm*)**: 169.8 (C-4''), 167.1 (C-8), 161.5 (C-2''), 158.1 (C-6''), 152.2 (C-2), 142.0 (C-4a), 139.0 (C-7a), 136.5 (C-1'''), 128.5 (C-3''', C-5'''), 128.0 (C-4'''), 127.6 (C-2''', C-6'''), 124.1 (C-6), 124.0 (C-5), 123.8 (C-2'''', *q*, $J_{(C-F)}$ = 281.6 Hz), 122.0 (C-4), 109.6 (C-7), 97.1 (C-5''), 67.1 (C-7''), 53.2 (C-2', C-6'), 52.3 (C-9), 46.8 (C-1'''', *q*, $J_{(C-F)}$ = 36.6 Hz), 43.6 (C-3', C-5'). **HRMS** (**ESI+**): found *m/z* 526.9820 $[M + H]^+$; calculated $C_{26}H_{26}F_3N_6O_3^+$ 527.1940.

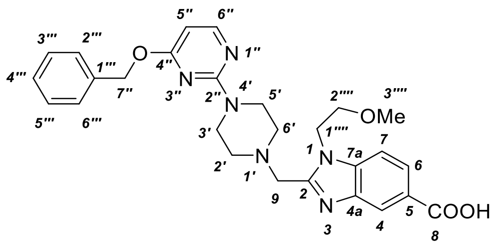

2-((4-(4-(Benzyloxy)pyrimidin-2-yl)piperazin-1-yl)methyl)-1-(2-methoxyethyl)-1H-benzo[d]imidazole-5-carboxylic acid **12i**, obtained according to **Method A**, white solid, 70 mg, 90%, mp 173-174°C. **^{1}H NMR (400 MHz, DMSO-*d*$_6$, *δ ppm*)**: 8.19 (1H, *d*, *J* = 1.4 Hz, 4-CH), 8.10 (1H, *d*, *J* = 5.6 Hz, 6''-CH), 7.87 (1H, *dd*, *J* = 8.5, 1.6 Hz, 6-CH), 7.66 (1H, *d*, *J* = 8.5 Hz, 7-CH), 7.44-7.30 (5H, *m*, 2'''-CH, 3'''-CH, 4'''-CH, 5'''-CH, 6'''-CH), 6.12 (1H, *d*, *J* = 5.6 Hz, 5''-CH), 5.34 (2H, *s*, 7''-CH_2), 4.60 (2H, *t*, *J* = 5.3 Hz, 1''''-CH_2), 3.87 (2H, *s*, 9-CH_2), 3.78-3.68 (6H, *m*, *J* = 5.3 Hz, 2''''-CH_2, 4H, *m*, 3'-CH_2, 5'-CH_2), 3.23 (3H, *s*, 3''''-CH_3), 2.53-2.50 (4H, *m*, 2'-CH_2, 6'-CH_2). **^{13}C NMR (101 MHz, DMSO-*d*$_6$, *δ ppm*)**: 169.2 (C-4''), 168.4 (C-8), 161.5 (C-2''), 159.1 (C-6''), 153.8 (C-2), 141.9 (C-4a), 139.5 (C-7a), 137.3 (C-1'''), 128.8 (C-3''', C-5'''), 128.6 (C-2''', C-6'''), 128.4 (C-4'''), 124.9 (C-5), 123.9 (C-4), 121.1 (C-6), 111.0 (C-7), 96.9 (C-5''), 71.4 (C-2''''), 67.2 (C-7''), 58.9 (C-3''''), 55.1 (C-9), 53.0 (C-2', C-6'), 44.1 (C-1''''), 43.9 (C-3', C-5'). **HRMS** (**ESI+**): found *m/z* 503.2388 $[M + H]^+$; calculated $C_{27}H_{31}N_6O_4^+$ 503.2329

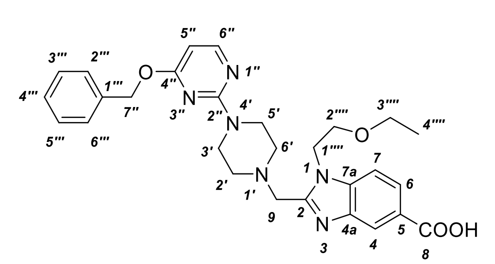

2-((4-(4-(Benzyloxy)pyrimidin-2-yl)piperazin-1-yl)methyl)-1-(2-ethoxyethyl)-1H-benzo[d]imidazole-5-carboxylic acid **12j**, obtained according to **Method A**, white solid, 75 mg, 85%, mp 152-153°C. **^{1}H NMR (400 MHz, DMSO-*d*$_6$, *δ ppm*)**: 8.24 (1H, *d*, *J* = 1.5 Hz, 4-CH), 8.15 (1H, *d*, *J* = 5.6 Hz, 6''-CH), 7.92 (1H, *dd*, *J* = 8.5, 1.6 Hz, 6-CH), 7.70 (1H, *d*, *J* = 8.5 Hz, 7-CH), 7.49-7.35 (5H, *m*, 2'''-CH, 3'''-CH, 4'''-CH, 5'''-CH, 6'''-CH), 6.17 (1H, *d*, *J* = 5.6 Hz, 5''-CH), 5.39 (2H, *s*, 7''-CH_2), 4.64 (2H, *t*, *J* = 5.4 Hz, 1''''-CH_2), 3.93 (2H, *s*, 9-CH_2), 3.83 (2H, *t*, *J* = 5.4 Hz, 2''''-CH_2), 3.78-3.76 (4H, *m*, 3'-CH_2, 5'-CH_2), 3.45 (2H, *q*, *J* = 7.0 Hz, 3''''-CH_2), 2.53-2.51 (4H, *m*, 2'-CH_2, 6'-CH_2), 1.08 (3H, *t*, *J* = 7.0 Hz, 4''''-CH_3). **^{13}C NMR (101 MHz, DMSO-*d*$_6$, *δ ppm*)**: 169.2 (C-4''), 161.5 (C-2''), 160.1 (C-8), 159.1 (C-6''), 153.7 (C-2), 141.9 (C-4a), 139.3 (C-7a), 137.3 (C-1'''), 128.8 (C-3''', C-5'''), 128.6 (C-2''', C-6'''), 128.4 (C-4'''), 125.5 (C-5), 123.9 (C-4), 121.0 (C-6), 110.9 (C-7), 96.9 (C-5''), 69.2 (C-2''''), 67.2 (C-7''), 66.2 (C-3''''), 55.1 (C-9), 53.0 (C-2', C-6'), 44.3 (C-1''''), 43.9 (C-3', C-5'), 15.5 (C-4''''). **HRMS** (**ESI+**): found *m/z* 517.2539 $[M + H]^+$; calculated $C_{28}H_{33}N_6O_4^+$ 517.2485.

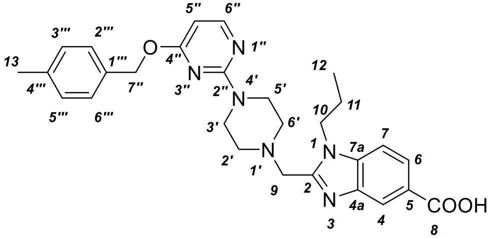

2-((4-(4-((4-Methylbenzyl)oxy)pyrimidin-2-yl)piperazin-1-yl)methyl)-1-propyl-1H-benzo[d]imidazole-5-carboxylic acid **12k**, obtained according to **Method A**, white solid, 55 mg, 85%, mp 120-121°C. **^{1}H NMR** (**400 MHz, DMSO-*d_6*, *δ ppm***): 12.72 (1H, *br.s.*, 8-COOH), 8.24 (1H, *d*, *J* = 1.4 Hz, 4-CH), 8.14 (1H, *d*, *J* = 5.6 Hz, 6"-CH), 7.93 (1H, *dd*, *J* = 8.5, 1.6 Hz, 6-CH), 7.72 (1H, *d*, *J* = 8.5 Hz, 7-CH), 7.37-7.35 (2H, *m*, 2'''-CH, 6'''-CH), 7.22 (2H, *d*, 3'''-CH, 5'''-CH), 6.14 (1H, *d*, *J* = 5.6 Hz, 5"-CH), 5.34 (2H, *s*, 7"-CH_2),4.38 (2H, *t*, *J* = 7.6 Hz,10-CH), 3.91 (3H, *s*, 9-CH_3), 3.76 (4H, *t*, 3'-CH_2, 5'-CH_2), 2.59-2.55 (4H, *m*, 2'-CH_2, 6'-CH_2), 2.35 (3H, *s*, 13-CH_3), 1.96-1.87 (2H, *h*, *J* = 7.5 Hz, 11-CH_2), 1.00 (3H, *t*, *J* = 7.4 Hz, 12-CH_3). **^{13}C NMR** (**101 MHz, DMSO-*d_6*, *δ ppm***): 169.3 (C-4"), 168.3 (C-8), 161.5 (C-2"), 159.0 (C-6"), 153.5 (C-2), 142.0 (C-4a), 139.2 (C-7a), 137.6 (C-1'''), 134.2 (C-4'''), 129.4 (C-3''', C-5'''), 128.7 (C-2''', C-6'''), 124.7 (C-5), 123.9 (C-4), 121.2 (C-6), 110.7 (C-7), 96.9 (C-5"), 67.1 (C-7"), 55.1 (C-9), 52.9 (C-2', C-6'), 45.7 (C-10), 44.0 (C-3', C-5'), 23.2 (C-11), 21.2 (C-13), 11.6 (C-12). **HRMS** (**ESI+**): found *m/z* 501.2589 [M + H]$^+$; calculated $C_{28}H_{33}N_6O_3^+$ 501.2536.

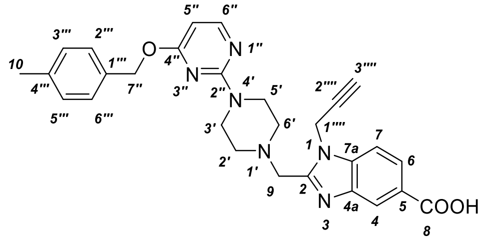

2-((4-(4-((4-Methylbenzyl)oxy)pyrimidin-2-yl)piperazin-1-yl)methyl)-1-(prop-2-yn-1-yl)-1H-benzo[d]imidazole-5-carboxylic acid **12l**, obtained according to **Method B**, white solid, 45 mg, 75%, mp 149-150°C. **^{1}H NMR** (**400 MHz, DMSO-*d_6*, *δ ppm***): 8.12 (1H, *s*, 4-CH), 8.10 (1H, *d*, *J* = 5.6 Hz, 6"-CH), 7.86 (1H, *d*, *J* = 8.6 Hz, 6-CH), 7.53 (1H, *d*, *J* = 8.5 Hz, 7-CH), 7.40-7.28 (5H, *m*, 2'''-CH, 3'''-CH, 4'''-CH, 5'''-CH, 6'''-CH), 6.10 (1H, *d*, *J* = 5.6 Hz, 5"-CH), 5.32 (2H, *s*, 7"-CH_2), 5.22 (2H, *d*, *J* = 3.2 Hz, 1''''-CH_2), 3.80 (2H, *s*, 9-CH_2), 3.71-3.68 (4H, *m*, 3'-CH_2, 5'-CH_2), 2.52-2.48 (4H, *m*, 2'-CH_2, 6'-CH_2), 2.32 (3H, *s*, 10-CH_3), 2.24 (1H, *t*, *J* = 2.8 Hz, 3''''-CH). **^{13}C NMR** (**101 MHz, DMSO-*d_6*, *δ ppm***): 169.9 (C-4"), 167.0 (C-8), 161.1 (C-2"), 158.4 (C-6"), 152.2 (C-2), 142.0 (C-4a), 139.2 (C-7a), 136.9 (C-1'''), 132.1 (C-4'''), 128.4 (C-3''', C-5'''), 127.9 (C-2''', C-6'''), 124.1 (C-6), 124.0 (C-5), 122.1 (C-4), 109.8 (C-7), 97.2 (C-5"), 76.7 (C-2''''), 73.9 (C-3''''), 67.0 (C-7"), 53.2 (C-2', C-6'), 52.1 (C-9), 43.7 (C-3', C-5'), 34.6 (C-1''''), 20.6 (C-10). **HRMS** (**ESI+**): found *m/z* 497.0893 [M + H]$^+$; calculated $C_{28}H_{29}N_6O_3^+$ 497.2223.

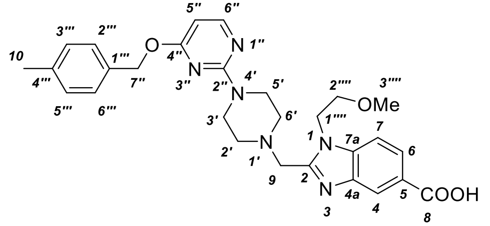

1-(2-Methoxyethyl)-2-((4-(4-((4-methylbenzyl)oxy)pyrimidin-2-yl)piperazin-1-yl)methyl)-1H-benzo[d]imidazole-5-carboxylic acid **12m**, obtained according to **Method A**, white solid, 70 mg, 90%, mp 98-99°C. **^{1}H NMR** (**400 MHz, DMSO-*d_6*, *δ ppm***): 8.19 (1H, *d*, *J* = 1.5 Hz, 4-CH), 8.09 (1H, *d*, *J* = 5.6 Hz, 6"-CH), 7.87 (1H, *dd*, *J* = 8.6, 1.6 Hz, 6-CH), 7.66 (1H, *d*, *J* = 8.6 Hz, 7-CH), 7.36-7.30 (2H, *m*, 2'''-CH, 6'''-CH), 7.21-7.16 (2H, *m*, 3'''-CH, 5'''-CH), 6.09 (1H, *d*, *J* = 5.6 Hz, 5"-CH), 5.29 (2H, *s*, 7"-CH_2), 4.60 (2H, *t*, *J* = 5.4 Hz, 1''''-CH_2), 3.88 (2H, *s*, 10-CH_2), 3.77-3.74 (6H, *m*, 3'-CH_2, 5'-CH_2, 2''''-CH_2), 3.23 (3H, *s*, 3''''-CH_3), 2.53-2.51 (4H, *m*, 2'-CH_2, 6'-CH_2), 2.30 (3H, *s*, 11-CH_3). **^{13}C NMR** (**101 MHz, DMSO-*d_6*, *δ ppm***): 169.3 (C-4"), 168.3 (C-8), 161.5 (C-2"), 159.0 (C-6"), 153.7 (C-2), 141.9 (C-4a), 139.5 (C-7a), 137.6 (C-1'''), 134.2 (C-4'''), 129.4 (C-3''', C-5'''), 128.7 (C-2''', C-6'''), 124.7 (C-4), 123.9 (C-5), 121.1 (C-6), 110.0 (C-7), 96.9 (C-5"), 71.4 (C-2''''), 67.1 (C-7"), 58.9 (C-3''''), 55.0 (C-9), 53.0 (C-2', C-6'), 44.1 (C-1''''), 43.9 (C-3', C-5'), 21.2 (C-10). **HRMS** (**ESI+**): found *m/z* 517.2550 [M + H]$^+$; calculated $C_{28}H_{33}N_6O_4^+$ 517.2485.

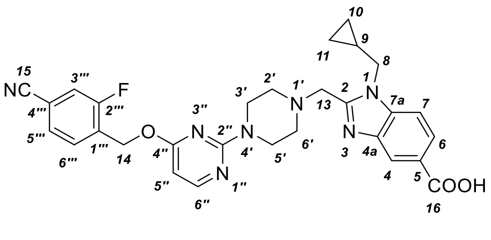

2-((4-(4-((4-Cyano-2-fluorobenzyl)oxy)pyrimidin-2-yl)piperazin-1-yl)methyl)-1-cyclopropylmethyl-1H-benzo[d]imidazole-5-carboxylic acid **12n**, obtained according to **Method B**, white solid, 70 mg, 80%, mp 111-112°C. **^{1}H NMR** (**400 MHz, DMSO-*d_6*, *δ ppm***): 12.69 (1H, *br.s.*, 16-COOH), 8.20 (1H, *d*, *J* = 1.4 Hz, 4-CH), 8.12 (1H, *d*, *J* = 5.6 Hz, 6"-CH), 7.91-7.86 (2H, *m*, 6-CH, 3'''-CH), 7.73-7.68 (3H, *m*, 6'''-CH, 7-CH, 5'''-CH), 6.15 (1H, *d*, *J* = 5.6 Hz, 5"-CH), 5.46 (2H, *s*, 14-CH_2), 4.29 (2H, *d*, *J* = 7.0 Hz, 8-CH_2), 3.86 (2H, *s*, 13-CH_2), 3.70-3.67 (4H, *m*, 3'-CH_2, 5'-CH_2), 2.53-2.50 (4H, *m*, 2'-CH_2, 6'-CH_2), 1.47-1.40 (1H, *m*, 9-CH), 0.53-0.51 (4H, *m*, 10-CH_2, 11-CH_2). **^{13}C NMR** (**101 MHz, DMSO-*d_6*, *δ ppm***): 168.7 (C-4"), 168.3 (C-16), 161.4 (C-2"), 160.1 (C-2''', *d*, $J_{(C-F)}$ = 248.7 Hz), 159.4 (C-6"), 153.3 (C-2), 142.0 (C-4a), 139.3 (C-7a), 131.9 (C-6''', *d*, $J_{(C-F)}$ = 4.5 Hz), 130.6 (C-1''', *d*, $J_{(C-F)}$ = 15.1 Hz), 129.3 (C-5''', *d*, $J_{(C-F)}$ = 3.6 Hz), 124.6 (C-5), 123.9 (C-6), 121.2 (C-4), 119.8 (C-3''', *d*, $J_{(C-F)}$ = 25.3 Hz), 118.0 (C-15, *d*, $J_{(C-F)}$ = 2.7 Hz), 112.8 (C-4''', *d*, $J_{(C-F)}$ = 9.8 Hz), 111.1 (C-7), 96.7 (C-5"), 60.7 (C-14, *d*, $J_{(C-F)}$ = 3.7 Hz), 55.0 (C-13), 52.9 (C-2', C-6'), 48.3 (C-8), 43.8 (C-3', C-5'), 11.7 (C-9), 4.4 (C-10, C-11). **HRMS** (**ESI+**): found *m/z* 542.2306 [M + H]$^+$; calculated $C_{29}H_{29}FN_7O_3^+$ 542.2238.

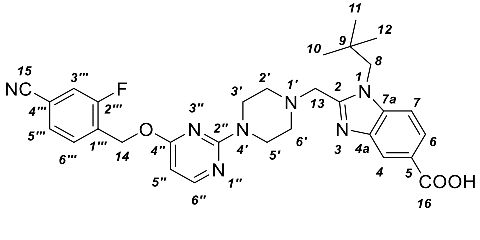

2-((4-(4-((4-Cyano-2-fluorobenzyl)oxy)pyrimidin-2-yl)piperazin-1-yl)methyl)-1-neopentyl-1H-benzo[d]imidazole-5-carboxylic acid **12o**, obtained according to **Method B**, white solid, 70 mg, 90%, mp 170-171°C. **^{1}H NMR (400 MHz, DMSO-d_6, *δ ppm*)**: 12.67 (1H, *br.s.*, 16-COOH), 8.19 (1H, *d*, *J* = 1.5 Hz, 4-CH), 8.12 (1H, *d*, *J* = 5.6 Hz, 6''-CH), 7.89 (1H, *dd*, *J* = 10.1, 1.4 Hz, 3'''-CH), 7.85 (1H, *dd*, *J* = 8.6, 1.6 Hz, 6-CH), 7.73-7.67 (3H, *m*, 6'''-CH, 7-CH, 5'''-CH), 6.14 (1H, *d*, *J* = 5.6 Hz, 5''-CH), 5.46 (2H, *s*, 14-CH_2), 4.33 (2H, *s*, 8-CH_2), 3.89 (2H, *s*, 13-CH_2), 3.70-3.68 (4H, *m*, 3'-CH_2, 5'-CH_2), 2.52-2.49 (4H, *m*, 2'-CH_2, 6'-CH_2), 1.00 (9H, *s*, 10-CH_3, 11-CH_3, 12-CH_3). **^{13}C NMR (101 MHz, DMSO-d_6, *δ ppm*)**: 168.7 (C-4''), 168.3 (C-16), 161.3 (C-2''), 160.0 (C-2''', *d*, $J_{(C-F)}$ = 249.0 Hz), 159.4 (C-6''), 153.9 (C-2), 141.9 (C-4a), 140.4 (C-7a), 131.8 (C-6''', *d*, $J_{(C-F)}$ = 4.4 Hz), 130.6 (C-1''', *d*, $J_{(C-F)}$ = 14.6 Hz), 129.3 (C-5''', *d*, $J_{(C-F)}$ = 3.7 Hz), 124.6 (C-5), 123.6 (C-6), 121.1 (C-4), 119.8 (C-3''', *d*, $J_{(C-F)}$ = 25.1 Hz), 118.0 (C-15, *d*, $J_{(C-F)}$ = 3.3 Hz), 112.8 (C-4''', *d*, $J_{(C-F)}$ = 9.9 Hz), 112.2 (C-7), 96.7 (C-5''), 60.7 (C-14, *d*, $J_{(C-F)}$ = 3.6 Hz), 55.4 (C-13), 54.3 (C-8), 52.8 (C-2', C-6'), 43.8 (C-3', C-5'), 35.1 (C-9), 28.7 (C-10, C-11, C-12). **HRMS (ESI+)**: found *m/z* 558.2627 $[M + H]^+$; calculated $C_{30}H_{33}FN_7O_3^+$ 558.2551.

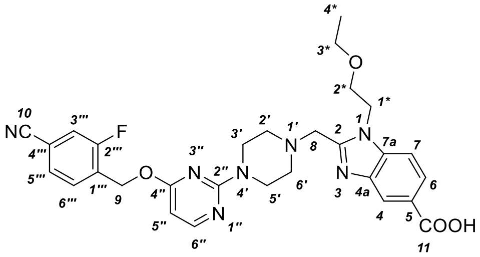

2-((4-(4-((4-Cyano-2-fluorobenzyl)oxy)pyrimidin-2-yl)piperazin-1-yl)methyl)-1-(2-ethoxyethyl)-1H-benzo[d]imidazole-5-carboxylic acid **12p**, obtained according to **Method B**, white solid, 75 mg, 85%, mp 225-226°C. **^{1}H NMR (400 MHz, DMSO-d_6, *δ ppm*)**: 12.68 (1H, *br.s.*, 11-COOH), 8.19 (1H, *d*, *J* = 1.5 Hz, 4-CH), 8.12 (1H, *d*, *J* = 5.6 Hz, 6''-CH), 7.90-7.85 (2H, *m*, 3'''-CH, 6-CH), 7.73-7.65 (3H, *m*, 6'''-CH, 5'''-CH, 7-CH), 6.15 (1H, *d*, *J* = 5.6 Hz, 5''-CH), 5.46 (2H, *s*, 9-CH_2), 4.58 (2H, *t*, *J* = 5.4 Hz, 1^*-CH_2), 3.88 (2H, *s*, 8-CH_2), 3.77 (2H, *t*, *J* = 5.4 Hz, 2^*-CH_2), 3.71-3.68 (4H, *m*, 3'-CH_2, 5'-CH_2), 3.39 (2H, *q*, *J* = 7.0 Hz, 3^*-CH_2), 2.52-2.49 (4H, *m*, 2'-CH_2, 6'-CH_2), 1.02 (3H, *t*, *J* = 7.0 Hz, 4^*-CH_3). **^{13}C NMR (101 MHz, DMSO-d_6, *δ ppm*)**: 168.8 (C-4''), 168.3 (C-11), 161.4 (C-2''), 160.1 (C-2''', *d*, $J_{(C-F)}$ = 249.0 Hz), 159.4 (C-6''), 153.8 (C-2), 141.9 (C-4a), 139.5 (C-7a), 131.9 (C-6''', *d*, $J_{(C-F)}$ = 4.8 Hz), 130.6 (C-1''', *d*, $J_{(C-F)}$ = 14.5 Hz), 129.3 (C-5''', *d*, $J_{(C-F)}$ = 3.6 Hz), 124.7 (C-5), 123.9 (C-6), 121.1 (C-4), 119.8 (C-3''', *d*, $J_{(C-F)}$ = 25.5 Hz), 118.0 (C-10, *d*, $J_{(C-F)}$ = 3.1 Hz), 112.8 (C-4''', *d*, $J_{(C-F)}$ = 9.8 Hz), 111.1 (C-7), 96.7 (C-5''), 69.2 (C-2^*), 66.2 (C-3^*), 60.7 (C-9, *d*, $J_{(C-F)}$ = 3.5 Hz), 55.0 (C-8), 52.9 (C-2', C-6'), 44.3 (C-1^*), 43.8 (C-3', C-5'), 15.5 (C-4^*). **HRMS (ESI+)**: found *m/z* 560.2424 $[M + H]^+$; calculated $C_{29}H_{31}FN_7O_4^+$ 560.2343.

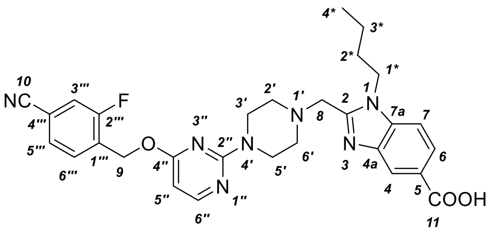

2-((4-(4-((4-Cyano-2-fluorobenzyl)oxy)pyrimidin-2-yl)piperazin-1-yl)methyl)-1-butyl-1H-benzo[d]imidazole-5-carboxylic acid **12q**, obtained according to **Method B**, white solid, 85 mg, 90%, mp 196-197°C. **^{1}H NMR (400 MHz, DMSO-d_6, *δ ppm*)**: 8.19 (1H, *br.s.*, 4-CH), 8.12 (1H, *d*, *J* = 5.6 Hz, 6''-CH), 7.89-7.86 (2H, *m*, 6-CH, 3'''-CH), 7.73-7.64 (3H, *m*, 6'''-CH, 5'''-CH, 7-CH), 6.15 (1H, *d*, *J* = 5.6 Hz, 5''-CH), 5.46 (2H, *s*, 9-CH_2), 4.35 (2H, *t*, *J* = 7.7 Hz, 1^*-CH_2), 3.84 (2H, *s*, 8-CH_2), 3.69-3.67 (4H, *m*, 3'-CH_2, 5'-CH_2), 2.54-2.49 (4H, *m*, 2'-CH_2, 6'-CH_2), 1.86 (2H, *ddd*, *J* = 15.1, 9.4, 5.0 Hz, 2^*-CH_2), 1.39 (2H, *h*, *J* = 7.5 Hz, 3^*-CH_2), 0.94 (3H, *t*, *J* = 7.4 Hz, 4^*-CH_3). **^{13}C NMR (101 MHz, DMSO-d_6, *δ ppm*)**: 168.8 (C-4''), 168.4 (C-11), 161.4 (C-2''), 160.1 (C-2''', *d*, $J_{(C-F)}$ = 249.0 Hz), 159.4 (C-6''), 153.3 (C-2), 142.0 (C-4a), 139.1 (C-7a), 131.9 (C-5''', *d*, $J_{(C-F)}$ = 4.5 Hz), 130.6 (C-1''', *d*, $J_{(C-F)}$ = 14.5 Hz), 128.3 (C-6''', *d*, $J_{(C-F)}$ = 3.6 Hz), 124.8 (C-7), 124.0 (C-5), 121.2 (C-4), 119.8 (C-3''', *d*, $J_{(C-F)}$ = 25.4 Hz), 118.0 (C-10, *d*, $J_{(C-F)}$ = 2.8 Hz), 112.8 (C-4''', *d*, $J_{(C-F)}$ = 10.1 Hz), 110.6 (C-6), 96.8 (C-5''), 60.8 (C-9, *d*, $J_{(C-F)}$ = 3.6 Hz), 55.1 (C-8), 52.9 (C-2', C-6'), 44.1 (C-1^*), 43.9 (C-3', C-5'), 31.9 (C-2^*), 20.1 (C-3^*), 14.1 (C-4^*). **HRMS (ESI+)**: found *m/z* 544.2472 $[M + H]^+$; calculated $C_{29}H_{31}FN_7O_3^+$ 544.2394.

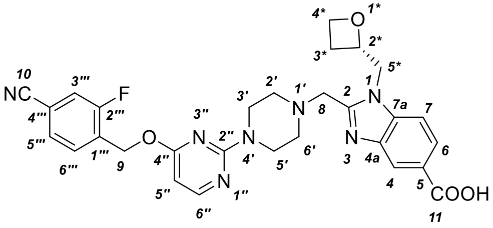

(S)-2-((4-(4-((4-Cyano-2-fluorobenzyl)oxy)pyrimidin-2-yl)piperazin-1-yl)methyl)-1-(oxetan-2-ylmethyl)-1H-benzo[d]imidazole-5-carboxylic acid **12r**, obtained according to **Method B**, white solid, 90 mg, 80%, mp 201-202°C. **^{1}H NMR (400 MHz, DMSO-d_6, *δ ppm*)**: 12.71 (1H, *br.s.*, 11-COOH), 8.19 (1H, *d*, *J* = 1.5 Hz, 4-CH), 8.12 (1H, *d*, *J* = 5.6 Hz, 6''-CH), 7.90-7.85 (2H, *m*, 6-CH, 5'''-CH), 7.73-7.67 (3H, *m*, 6'''-CH, 7-CH, 3'''-CH), 6.15 (1H, *d*, *J* = 5.6 Hz, 5''-CH), 5.47 (2H, *s*, 9-CH_2), 5.12 (1H, *qd*, *J* = 7.2, 2.9 Hz, 2^*-CH), 4.77 (1H, *dd*, *J* = 15.2, 7.1 Hz, 5^*-CH_2), 4.64 (1H, *dd*, *J* = 15.2, 3.0 Hz, 5^*-CH_2), 4.49 (1H, *td*, *J* = 8.0, 5.8 Hz, 4^*-CH_2), 4.39 (1H, *dt*, *J* = 9.0, 5.9 Hz, 4^*-CH_2), 3.98-3.81 (1H, *m*, 8-CH_2), 3.71-3.69 (4H, *m*, 3'-CH_2, 5'-CH_2), 2.72 (1H, *ddd*, *J* = 11.3, 8.5, 6.2 Hz, 3^*-CH_2), 2.53-2.49 (4H, *m*, 2'-CH_2, 6'-CH_2), 2.47-2.38 (1H, *m*, 3^*-CH_2). **^{13}C NMR (101 MHz, DMSO-d_6, *δ ppm*)**: 168.8 (C-4''), 168.3 (C-11), 161.4 (C-2''), 160.1 (C-2''', *d*, $J_{(C-F)}$ = 248.9 Hz), 159.4 (C-6''), 153.8 (C-2), 141.9 (C-4a), 139.8 (C-7a), 131.9 (C-5''', *d*, $J_{(C-F)}$ = 4.6 Hz), 130.6 (C-1''', *d*, $J_{(C-F)}$ = 14.5 Hz), 129.3 (C-6''', *d*, $J_{(C-F)}$ = 3.7 Hz), 124.8 (C-5), 123.9 (C-7), 121.1 (C-4), 119.8 (C-3''', *d*, $J_{(C-F)}$ = 25.3 Hz), 118.0 (C-10, *d*,

$J_{(C-F)}$ = 3.4 Hz), 112.8 (C-4''', *d*, $J_{(C-F)}$ = 10.2 Hz), 111.4 (C-6), 96.7 (C-5''), 80.9 (C-2*), 68.0 (C-4*), 60.7 (C-9, *d*, $J_{(C-F)}$ = 3.5 Hz), 55.0 (C-8), 52.9 (C-2', C-6'), 49.5 (C-5*), 43.8 (C-3', C-5'), 24.9 (C-3*). **HRMS** (**ESI+**): found *m/z* 558.2266 $[M + H]^+$; calculated $C_{29}H_{29}FN_7O_4^+$ 558.2187.

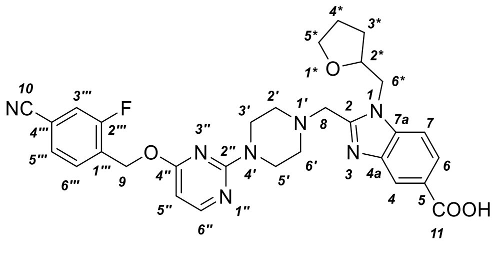

2-((4-(4-((4-Cyano-2-fluorobenzyl)oxy)pyrimidin-2-yl)piperazin-1-yl)methyl)-1-((tetrahydrofuran-2-yl)methyl)-1H-benzo[d]imidazole-5-carboxylic acid **12s**, obtained according to **Method B**, white solid, 100 mg, 85%, mp 222-223°C. **^{1}H NMR** (**400 MHz, DMSO-*d*$_6$, *δ ppm***): 8.18 (1H, *d*, *J* = 1.5 Hz, 4-CH), 8.12 (1H, *d*, *J* = 5.6 Hz, 6''-CH), 7.90-7.85 (2H, *m*, 6-CH, 3'''-CH), 7.73-7.67 (3H, *m*, 6'''-CH, 7-CH, 5'''-CH), 6.15 (1H, *d*, *J* = 5.6 Hz, 5''-CH), 5.46 (2H, *s*, 9-CH_2), 4.55-4.40 (2H, *m*, 6*-CH_2), 4.26 (1H, *qd*, *J* = 7.1, 3.2 Hz, 2*-CH), 3.98 (1H, *d*, *J* = 13.6 Hz, 8-CH), 3.84-3.61 (7H, *m*, 8-CH, 5*-CH_2, 3'-CH_2, 5'-CH_2), 2.53-2.46 (4H, *m*, 2'-CH_2, 6'-CH_2), 2.10-2.01 (1H, *m*, 3*-CH_2), 1.84 (2H, *dddd*, *J* = 19.1, 12.0, 9.4, 5.4 Hz, 4*-CH_2), 1.68-1.59 (1H, *m*, 3*-CH_2). **^{13}C NMR** (**101 MHz, DMSO-*d*$_6$, *δ ppm***): 168.7 (C-4''), 168.5 (C-11), 161.4 (C-2''), 160.1 (C-2''', *d*, $J_{(C-F)}$ = 249. Hz), 159.4 (C-6''), 153.7 (C-2), 141.9 (C-4a), 139.6 (C-7a), 131.9 (C-6''', *d*, $J_{(C-F)}$ = 4.8 Hz), 130.6 (C-1''', *d*, $J_{(C-F)}$ = 14.5 Hz), 129.3 (C-5''', *d*, $J_{(C-F)}$ = 3.7 Hz), 124.9 (C-5), 123.8 (C-6), 121.0 (C-4), 119.8 (C-3''', *d*, $J_{(C-F)}$ = 25.4 Hz), 118.0 (C-10, *d*, $J_{(C-F)}$ = 2.9 Hz), 112.8 (C-4''', *d*, $J_{(C-F)}$ = 10.1 Hz), 111.3 (C-7), 96.7 (C-5''), 78.2 (C-2*), 67.8 (C-5*), 60.7 (C-9, *d*, $J_{(C-F)}$ = 3.5 Hz), 55.1 (C-8), 52.9 (C-2', C-6'), 48.2 (C-6*), 43.8 (C-3', C-5'), 29.2 (C-3*), 25.7 (C-4*). **HRMS** (**ESI+**): found *m/z* 572.2422 $[M + H]^+$; calculated $C_{30}H_{31}FN_7O_4^+$ 572.2343.

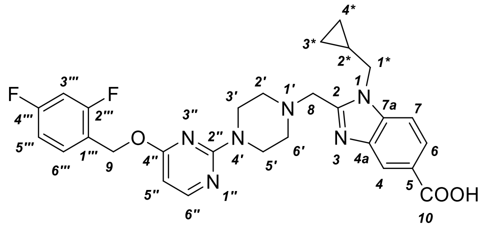

1-(Cyclopropylmethyl)-2-((4-(4-((2,4-difluorobenzyl)oxy)pyrimidin-2-yl)piperazin-1-yl)methyl)-1H-benzo[d]imidazole-5-carboxylic acid **12t**, obtained according to **Method B**, white solid, 75 mg, 85%, mp 128-129°C. **^{1}H NMR** (**400 MHz, $CDCl_3$, *δ ppm***): 8.71 (1H, *br.s.*, 10-COOH), 8.55 (1H, *d*, *J* = 1.5 Hz, 4-CH), 8.05-7.97 (2H, *m*, 6''-CH, 6-CH), 7.41 (1H, *d*, *J* = 8.6 Hz, 7-CH), 7.32 (1H, *td*, *J* = 8.4, 6.3 Hz, 6'''-CH), 6.81-6.71 (2H, *m*, 5'''-CH, 3'''-CH), 5.96 (1H, *d*, *J* = 5.7 Hz, 5''-CH), 5.27 (2H, *s*, 9-CH_2), 4.24 (2H, *d*, *J* = 6.8 Hz, 1*-CH_2), 3.95 (2H, *s*, 8-CH_2), 3.75(4H, *t*, , *J* = 5.0 Hz, 3'-CH_2, 5'-CH_2), 2.60 (4H, *t*, , *J* = 5.0 Hz, 2'-CH_2, 6'-CH_2), 1.35-1.25 (1H, *m*, 2*-CH), 0.61-0.56 (2H, *m*, 3*-CH_2, 4*-CH_2), 0.42 (2H, *dt*, , *J* = 6.3, 4.9 Hz, 3*-CH_2, 4*-CH_2). **^{13}C NMR** (**101 MHz, $CDCl_3$, *δ ppm***): 170.6 (C-10), 169.1 (C-4''), 162.8 (C-4''', *dd*, $J_{(C-F)}$ = 249.3, 12.0 Hz), 161.2 (C-2''), 160.9 (C-2''', *dd*, $J_{(C-F)}$ = 250.4, 12.0 Hz), 157.9 (C-6''), 152.2 (C-2), 140.9 (C-4a), 138.9 (C-7a), 131.3 (C-6''', *dd*, $J_{(C-F)}$ = 9.8, 5.5 Hz), 125.1 (C-6), 124.6 (C-5), 122.3 (C-4), 119.9 (C-1''', *dd*, $J_{(C-F)}$ = 14.9, 4.0 Hz), 111.4 (C-5''', *dd*, $J_{(C-F)}$ = 21.3, 3.8 Hz), 109.9 (C-7), 103.9 (C-3''', *t*, $J_{(C-F)}$ = 25.3 Hz), 97.1 (C-5''), 60.5 (C-9, *d*, $J_{(C-F)}$ = 3.8 Hz), 55.0 (C-8), 53.0 (C-2', C-6'), 48.8 (C-1*), 43.7 (C-3', C-5'), 11.2 (C-2*), 4.5 (C-3*, C-4*). **HRMS** (**ESI+**): found *m/z* 535.2287 $[M + H]^+$; calculated $C_{28}H_{29}F_2N_6O_3^+$ 535.2191.

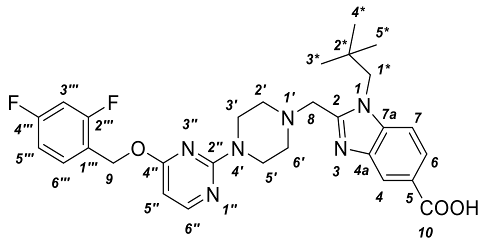

2-((4-(4-((2,4-Difluorobenzyl)oxy)pyrimidin-2-yl)piperazin-1-yl)methyl)-1-neopentyl-1H-benzo[d]imidazole-5-carboxylic acid **12u**, obtained according to **Method B**, white solid, 80 mg, 75%, mp 102-103°C. **^{1}H NMR** (**400 MHz, $CDCl_3$, *δ ppm***): 9.37 (1H, *br.s.*, 10-COOH), 8.56 (1H, *s*, 4-CH), 8.02 (1H, *m*, 6-CH, 6''-CH), 7.39 (1H, *d*, *J* = 8.6 Hz, 7-CH), 7.32 (1H, *q*, *J* = 7.9 Hz, 6'''-CH), 6.81-6.71 (2H, *m*, 5'''-CH, 3'''-CH), 5.94 (1H, *d*, *J* = 5.6 Hz, 5''-CH), 5.26 (2H, *s*, 9-CH_2), 4.29 (2H, *s*, 1*-CH_2), 4.02 (2H, *s*, 8-CH_2), 3.74 (4H, *t*, *J* = 4.9 Hz, 3'-CH_2, 5'-CH_2), 2.53 (4H, *m*, *J* = 5.0 Hz, 2'-CH_2, 6'-CH_2), 1.02 (9H, *s*, 3*-CH_3, 4*-CH_3, 5*-CH_3). **^{13}C NMR** (**101 MHz, $CDCl_3$, *δ ppm***): 170.8 (C-10'), 169.1 (C-4''), 162.8 (C-4''', *dd*, $J_{(C-F)}$ = 249.2, 12.2 Hz), 161.0 (C-2''), 160.9 (C-2''', *dd*, $J_{(C-F)}$ = 250.5, 12.1 Hz), 157.8 (C-6''), 152.9 (C-2), 140.8 (C-4a), 140.1 (C-7a), 131.3 (C-6''', *dd*, $J_{(C-F)}$ = 9.9, 5.6 Hz), 124.8 (C-6), 124.2 (C-5), 122.3 (C-4), 119.9 (C-1''', *dd*, $J_{(C-F)}$ = 14.6, 3.7 Hz), 111.4 (C-5''', *dd*, $J_{(C-F)}$ = 21.4, 3.9 Hz), 111.1 (C-7), 103.9 (C-3''', *t*, $J_{(C-F)}$ = 25.4 Hz), 97.2 (C-5''), 60.5 (C-9, *d*, $J_{(C-F)}$ = 3.9 Hz), 55.3 (C-8), 55.1 (C-1*), 52.9 (C-2', C-6'), 43.6 (C-3', C-5'), 35.1 (C-2*), 28.8 (C-3*, C-4*, C-5*). **HRMS** (**ESI+**): found *m/z* 551.2597 $[M + H]^+$; calculated $C_{29}H_{33}F_2N_6O_3^+$ 551.2504.

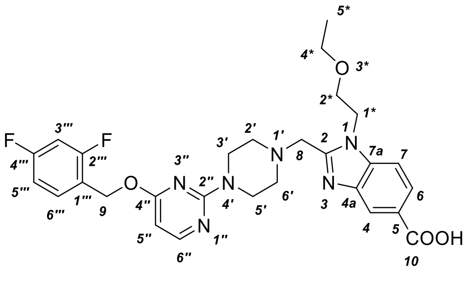

2-((4-(4-((2,4-Difluorobenzyl)oxy)pyrimidin-2-yl)piperazin-1-yl)methyl)-1-(2-ethoxyethyl)-1H-benzo[d]imidazole-5-carboxylic acid **12v**, obtained according to **Method B**, white solid, 50 mg, 90%, mp 85-86°C. **^{1}H NMR (400 MHz, $CDCl_3$, *δ ppm*)**: 8.56 (1H, *d*, *J* = 1.5 Hz, 4-CH), 8.03 (1H, *dd*, *J* = 8.5, 1.5 Hz, 6-CH), 8.00 (1H, *d*, *J* = 5.6 Hz, 6''-CH), 7.41 (1H, *d*, *J* = 8.6 Hz, 7-CH), 7.38-7.30 (1H, *m*, 6'''-CH), 6.82-6.72 (2H, *m*, 5'''-CH, 3'''-CH), 5.95 (1H, *d*, *J* = 5.6 Hz, 5''-CH), 5.27 (2H, *s*, 9-CH_2), 4.55 (2H, *t*, *J* = 5.3 Hz, 1*-CH_2), 4.00 (2H, *s*, 8-CH_2), 3.75 (6H, *t*, *J* = 5.3 Hz, 3'-CH_2, 5'-CH_2, 2*-CH_2), 3.35 (2H, *q*, *J* = 7.0 Hz, 4*-CH_2), 2.63-2.60 (4H, *m*, 2'-CH_2, 6'-CH_2), 1.05 (2H, *t*, *J* = 7.0 Hz, 5*-CH_3). **^{13}C NMR (101 MHz, $CDCl_3$, *δ ppm*)**: 170.9 (C-10), 169.1 (C-4''), 162.9 (C-4''', *dd*, $J_{(C-F)}$ = 249.3, 11.9 Hz), 161.1 (C-2''), 160.9 (C-2''', *dd*, $J_{(C-F)}$ = 250.6, 11.9 Hz), 157.8 (C-6''), 153.0 (C-2), 140.8 (C-4a), 139.0 (C-7a), 131.3 (C-6''', *dd*, $J_{(C-F)}$ = 9.7, 5.6 Hz), 125.1 (C-6), 124.3 (C-5), 122.4 (C-4), 119.9 (C-1''', *d*, $J_{(C-F)}$ = 13.5 Hz), 111.4 (C-5''', *dd*, $J_{(C-F)}$= 21.2, 3.8 Hz), 109.9 (C-7), 103.9 (C-3''', *t*, $J_{(C-F)}$ = 25.4 Hz), 97.1 (C-5''), 69.0 (C-2*), 67.0 (C-4*), 60.5 (C-9, *d*, $J_{(C-F)}$ = 3.6 Hz), 54.8 (C-8), 53.0 (C-2', C-6'), 44.7 (C-1*), 43.7 (C-3', C-5'), 15.1 (C-5*). **HRMS** (**ESI+**): found *m/z* 553.2393 $[M + H]^+$; calculated $C_{28}H_{31}F_2N_6O_4^+$ 553.2297.

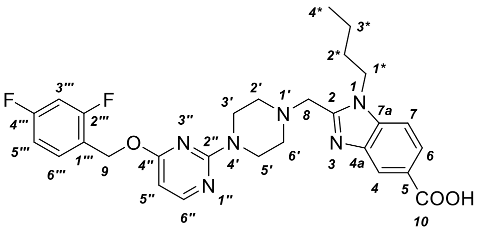

1-Butyl-2-((4-(4-((2,4-difluorobenzyl)oxy)pyrimidin-2-yl)piperazin-1-yl)methyl)-1H-benzo[d]imidazole-5-carboxylic acid **12w**, obtained according to **Method B**, white solid, 80 mg, 90%, mp 179-180°C. **^{1}H NMR (400 MHz, $CDCl_3$, *δ ppm*)**: 9.07 (1H, *br.s.*, 10-COOH), 8.52 (1H, *d*, *J* = 1.5 Hz, 4-CH), 8.06-7.97 (2H, *m*, 6''-CH, 6-CH), 7.38-7.30 (1H, *m*, 7-CH, 6'''-CH), 6.82-6.72 (2H, *m*, 5'''-CH, 3'''-CH), 5.97 (1H, *d*, *J* = 5.7 Hz, 5''-CH), 5.27 (2H, *s*, 9-CH_2), 4.32-4.28 (2H, *m*, 1*-CH_2), 3.91 (2H, *s*, 8-CH_2), 3.76-3.74 (4H, *m*, 3'-CH_2, 5'-CH_2), 2.51 (4H, *t*, *J* = 5.7 Hz, 2'-CH_2, 6'-CH_2), 1.83 (2H, *ddt*, *J* = 9.2, 7.7, 3.7 Hz, 2*-CH_2), 1.40 (2H, *h*, *J* = 7.4 Hz, 3*-CH_2), 0.94 (3H, *t*, *J* = 7.4 Hz, 4*-CH_3). **^{13}C NMR (101 MHz, $CDCl_3$, *δ ppm*)**: 170.9 (C-10), 169.1 (C-4''), 162.9 (C-4''', *dd*, $J_{(C-F)}$ = 249.3, 11.7 Hz), 161.1 (C-2''), 160.9 (C-2''', *dd*, $J_{(C-F)}$ = 250.5, 11.9 Hz), 157.8 (C-6''), 152.4 (C-2), 140.7 (C-4a), 138.7 (C-7a), 131.3 (C-6''', *dd*, $J_{(C-F)}$ = 10.0, 5.3 Hz), 125.1 (C-6), 124.3 (C-5), 122.4 (C-4), 119.9 (C-1''', *dd*, $J_{(C-F)}$ = 14.9, 3.8 Hz), 111.4 (C-5''', *dd*, $J_{(C-F)}$ = 21.1, 3.7 Hz), 109.6 (C-7), 103.9 (C-3''', *t*, $J_{(C-F)}$ = 25.4 Hz), 97.1 (C-5''), 60.5 (C-9, *d*, $J_{(C-F)}$ = 3.7 Hz), 54.9 (C-8), 53.0 (C-2', C-6'), 44.4 (C-1*), 43.7 (C-3', C-5'), 31.9 (C-2*), 20.3 (C-3*), 13.8 (C-4*). **HRMS** (**ESI+**): found *m/z* 537.2438 $[M + H]^+$; calculated $C_{28}H_{31}F_2N_6O_3^+$ 537.2347.

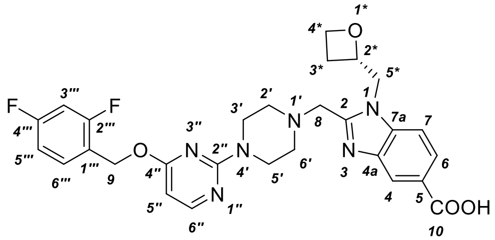

(S)-2-((4-(4-((2,4-Difluorobenzyl)oxy)pyrimidin-2-yl)piperazin-1-yl)methyl)-1-(oxetan-2-ylmethyl)-1H-benzo[d]imidazole-5-carboxylic acid **12x**, obtained according to **Method B**, white solid, 60 mg, 80%, mp 91-92°C. **^{1}H NMR (400 MHz, $CDCl_3$, *δ ppm*)**: 9.10 (1H, *br.s.*, 10-COOH), 8.59 (1H, *dd*, *J* = 1.6, 0.6 Hz, 4-CH), 8.04 (1H, *d*, *J* = 8.6 Hz, 6-CH), 8.01 (1H, *d*, *J* = 5.6 Hz, 6''-CH), 7.45 (1H, *d*, *J* = 8.6 Hz, 7-CH), 7.33 (1H, *q*, *J* = 7.8 Hz, 6'''-CH), 6.81-6.71 (2H, *m*, 5'''-CH, 3'''-CH), 5.95 (1H, *d*, *J* = 5.7 Hz, 5''-CH), 5.37 (2H, *s*, 9-CH_2), 5.20 (1H, *tt*, *J* = 7.7, 4.3 Hz, 2*-CH), 4.65 (2H, *d*, *J* = 4.4 Hz, 5*-CH_2), 4.57 (1H, *m*, 4*-CH_2), 4.31 (1H, *dt*, *J* = 9.1, 5.9 Hz, 4*-CH_2), 4.11-4.00 (2H, *m*, 8-CH_2), 3.74 (4H, *m*, 3'-CH_2, 5'-CH_2), 2.72-2.63 (1H, *m*, 3*-CH_2), 2.58 (2H, *m*, 2'-CH_2, 6'-CH_2), 2.44-2.36 (1H, *m*, 3*-CH_2). **^{13}C NMR (101 MHz, $CDCl_3$, *δ ppm*)**: 170.8 (C-10), 169.1 (C-4''), 162.8 (C-4''', *dd*, $J_{(C-F)}$ = 249.4, 11.9 Hz), 160.9 (C-2''), 160.8 (C-2''', *dd*, $J_{(C-F)}$ = 250.5, 12.0 Hz), 157.7 (C-6''), 152.7 (C-2), 140.7 (C-4a), 139.3 (C-7a), 131.3 (C-6''', *dd*, $J_{(C-F)}$ = 9.8, 5.4 Hz), 125.4 (C-6), 124.5 (C-5), 122.4 (C-4), 119.8 (C-1''', *dd*, $J_{(C-F)}$ = 14.6, 3.7 Hz), 111.4 (C-5''', *dd*, $J_{(C-F)}$ = 21.1, 3.7 Hz), 110.1 (C-7), 103.9 (C-3''', *t*, $J_{(C-F)}$ = 25.4 Hz), 97.3 (C-5''), 80.8 (C-2*), 68.4 (C-4*), 60.6 (C-9, *d*, $J_{(C-F)}$ = 3.7 Hz), 54.6 (C-8), 52.9 (C-2', C-6'), 49.6 (C-5*), 43.6 (C-3', C-5'), 24.7 (C-3*). **HRMS** (**ESI+**): found *m/z* 551.2233 $[M + H]^+$; calculated $C_{28}H_{29}F_2N_6O_4^+$ 551.2140.

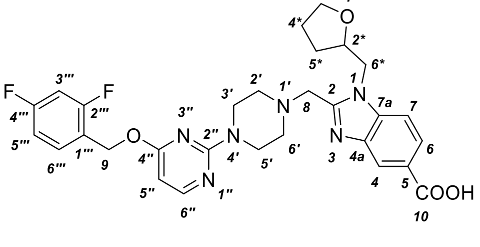

2-((4-(4-((2,4-Difluorobenzyl)oxy)pyrimidin-2-yl)piperazin-1-yl)methyl)-1-((tetrahydrofuran-2-yl)methyl)-1H-benzo[d]imidazole-5-carboxylic acid **12y**, obtained according to **Method B**, white solid, 55 mg, 75%, mp 94-95°C. **^{1}H NMR (400 MHz, $CDCl_3$, *δ ppm*)**: 8.57 (1H, *d*, *J* = 1.5 Hz, 4-CH), 8.02 (1H, *d*, *J* = 5.7 Hz, 6''-CH), 8.01 (1H, *dd*, *J* = 8.5, 1.5 Hz, 6-CH), 7.43 (1H, *d*, *J* = 8.6 Hz, 7-CH), 7.32 (1H, *td*, *J* = 8.4, 6.3 Hz, 6'''-CH), 6.82-6.72 (2H, *m*, 5'''-CH, 3'''-CH), 5.97 (1H, *d*, *J* = 5.7 Hz, 5''-CH), 5.27 (2H, *s*, 9-CH_2), 4.52-4.43 (2H, *m*, 6*-CH_2), 4.27-4.21 (1H, *m*, 2*-CH), 4.07-4.00 (2H, *m*, 8-CH_2), 3.84-3.78 (1H, *m*, 3*-CH_2), 3.74 (4H, *t*, 3'-CH_2, 5'-CH_2), 3.72-3.66 (1H, *m*, 3*-CH_2), 2.63-2.61 (4H, *m*, 2'-CH_2, 6'-CH_2), 2.07-1.99 (1H, *m*, 5*-CH_2), 1.88-1.80 (2H, *m*, 4*-CH_2), 1.63-1.54 (1H, *m*, 5*-CH_2). **^{13}C NMR (101 MHz, $CDCl_3$, *δ ppm*)**: 169.1 (C-4''), 162.9 (C-4''', *dd*, $J_{(C-F)}$ = 249.3, 11.9 Hz), 161.1 (C-2''), 160.9 (C-2''', *dd*, $J_{(C-F)}$ = 250.5, 12.2 Hz), 157.9 (C-6''), 153.0 (C-2), 140.8 (C-4a), 139.3 (C-7a), 131.3 (C-6''', *dd*, $J_{(C-F)}$ = 9.8, 5.5 Hz), 125.1 (C-6), 124.4 (C-5), 122.3 (C-4), 119.9

(C-1''', *dd*, $J_{(C-F)}$ = 14.6, 3.6 Hz), 111.4 (C-5''', *dd*, $J_{(C-F)}$ = 21.4, 3.8 Hz), 110.1 (C-7), 103.9 (C-3''', *t*, $J_{(C-F)}$ = 25.4 Hz), 97.1 (C-5''), 78.1 (C-2*), 68.3 (C-5*), 60.5 (C-9, *d*, $J_{(C-F)}$ = 4.0 Hz), 54.9 (C-8), 53.0 (C-2', C-6'), 48.3 (C-6*), 43.6 (C-3', C-5'), 29.3 (C-3*), 25.7 (C-4*). **HRMS** (**ESI+**): found *m/z* 565.2400 $[M + H]^+$; calculated $C_{29}H_{31}F_2N_6O_4^+$ 565.2297.

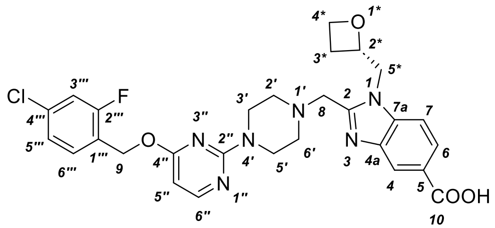

(S)-2-((4-(4-((4-chloro-2-fluorobenzyl)oxy)pyrimidin-2-yl)piperazin-1-yl)methyl)-1-(oxetan-2-ylmethyl)-1H-benzo[d]imidazole-5-carboxylic acid **12z**, obtained according to **Method B**, white solid, 95 mg, 85%, mp 114-115°C. **^{1}H NMR** (**400 MHz, DMSO-*d*$_6$, *δ ppm***): 12.80 (1H, *br.s.*, 11-COOH), 8.19 (1H, *br.s.*, 4-CH), 8.11 (1H, *d*, *J* = 5.6 Hz, 6''-CH), 7.86 (2H, *dd*, *J* = 8.5 Hz, 6-CH, 5'''-CH), 7.73 (1H, *d*, *J* = 8.6 Hz, 7-CH), 7.55 (1H, *t*, *J* = 8.1 Hz, 6'''-CH), 7.47 (1H, *d*, *J* = 10.0 Hz, 3'''-CH), 6.11 (1H, *d*, *J* = 5.5 Hz, 5''-CH), 5.37 (2H, *s*, 9-CH_2), 5.12 (1H, *q*, *J* = 7.4Hz, 2*-CH), 4.78 (1H, *dd*, *J* = 15.1, 7.2 Hz, 5*-CH_2), 4.64 (1H, *d*, *J* = 15.2 Hz, 5*-CH_2), 4.49 (1H, *q*, *J* = 7.2 Hz, 4*-CH_2), 4.39 (1H, *q*, *J* = 7.1 Hz, 4*-CH_2), 3.97 (1H, *d*, *J* = 13.5 Hz, 8-CH_2), 3.82 (1H, *d*, *J* = 13.6 Hz, 8-CH_2), 3.72 (4H, *br. s.*, 3'-CH_2, 5'-CH_2), 2.82-2.63 (1H, *m*, 3*-CH_2), 2.51 (4H, *br. s.*, 2'-CH_2, 6'-CH_2), 2.47-2.35 (1H, *m*, 3*-CH_2). **^{13}C NMR** (**101 MHz, DMSO-*d*$_6$, *δ ppm***): 168.9 (C-4''), 168.3 (C-11), 161.4 (C-2''), 160.8 (C-2''', *d*, $J_{(C-F)}$ = 248.9 Hz), 159.3 (C-6''), 153.9 (C-2), 141.9 (C-4a), 139.8 (C-7a), 134.3 (C-4''', *d*, $J_{(C-F)}$ = 10.8 Hz), 132.6 (C-6''', *d*, $J_{(C-F)}$ = 4.8 Hz), 125.2 (C-5''', *d*, $J_{(C-F)}$ = 3.5 Hz), 124.8 (C-5), 123.9 (C-6), 123.5 (C-1''', *d*, $J_{(C-F)}$ = 14.4 Hz), 121.1 (C-4), 116.5 (C-3''', *d*, $J_{(C-F)}$ = 25.3 Hz), 111.4 (C-7), 96.7 (C-5''), 80.9 (C-2*), 68.0 (C-4*), 60.7 (C-9, *d*, $J_{(C-F)}$ = 2.9 Hz), 55.0 (C-8), 52.9 (C-2', C-6'), 49.6 (C-5*), 43.9 (C-3', C-5'), 24.9 (C-3*). **HRMS** (**ESI+**): found *m/z* 567.1883 $[M + H]^+$; calculated $C_{28}H_{29}{}^{35}ClFN_6O_4^+$ 567.1845.

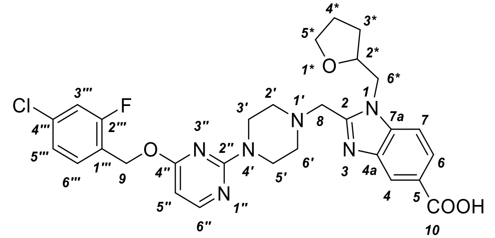

2-((4-(4-((4-chloro-2-fluorobenzyl)oxy)pyrimidin-2-yl)piperazin-1-yl)methyl)-1-((tetrahydrofuran-2-yl)methyl)-1H-benzo[d]imidazole-5-carboxylic acid, белые кристаллы **12aa**, 85 mg, 85%, mp 110-111°C. **^{1}H NMR** (**400 MHz, DMSO-*d*$_6$, *δ ppm***): 12.64 (1H, *br.s.*, 11-COOH), 8.18 (1H, *d*, *J* = 1.5 Hz, 4-CH), 8.11 (1H, *d*, *J* = 5.6 Hz, 6''-CH), 7.86 (2H, *dd*, *J* = 8.5, 1.5 Hz, 6-CH), 7.68 (1H, *d*, *J* = 8.6 Hz, 7-CH), 7.55 (1H, *t*, *J* = 8.2 Hz, 6'''-CH), 7.48 (1H, *dt*, *J* = 10.0, 2.6 Hz, 3'''-CH), 7.32 (1H, *dt*, *J* = 8.4, 2.8 Hz, 5'''-CH), 6.11 (1H, *d*, *J* = 5.6 Hz, 5''-CH), 5.37 (2H, *s*, 9-CH_2), 4.57-4.50 (1H, *m*, 8-CH_2), 4.44 (1H, *dd*, *J* = 14.8, 8.0 Hz, 6*-CH), 4.27 (1H, *qd*, *J* = 7.1, 3.1 Hz, 6*-CH), 4.27 (1H, *dq*, *J* = 7.1, 3.1 Hz, 6*-CH_2), 4.0 (1H, *d*, *J* = 13.6 Hz, 8-CH), 3.85-3.74 (1H, *m*, 8-CH, 5*-CH), 3.74-3.69 (4H, *m*, 3'-CH_2, 5'-CH_2), 3.69-3.60 (1H, *m*, 5*-CH_2), 2.59-2.48 (4H, *m*, 2'-CH_2, 4'-CH_2), 2.11-2.00 (1H, *m*, 3*-CH_2), 1.93-1.80 (1H, *m*, 4*-CH_2), 1.69-1.57 (1H, *m*, 3*-CH_2). **^{13}C NMR** (**101 MHz, DMSO-*d*$_6$, *δ ppm***): 168.9 (C-4''), 168.3 (C-10), 161.4 (C-2''), 160.8 (C-2''', *d*, $J_{(C-F)}$ = 250.5 Hz), 159.3 (C-6''), 153.8 (C-2), 141.9 (C-4a), 139.7 (C-7a), 134.2 (C-4''', *d*, $J_{(C-F)}$ = 10.5 Hz), 132.6 (C-6''', *d*, $J_{(C-F)}$ = 4.8 Hz), 125.2 (C-5''', *d*, $J_{(C-F)}$ = 3.6 Hz), 123.5 (C-1''', *d*, $J_{(C-F)}$ = 14.6 Hz), 124.6 (C-5), 123.8 (C-6), 121.0 (C-4), 116.5 (C-3''', *d*, $J_{(C-F)}$ = 26.0 Hz), 111.3 (C-7), 96.8 (C-5''), 78.2 (C-2*), 67.9 (C-5*), 60.7 (C-9, *m*), 55.1 (C-8), 53.0 (C-2', C-6'), 48.2 (C-6*), 43.8 (C-3', C-5'), 29.2 (C-3*), 25.7 (C-4*). **HRMS** (**ESI+**): found *m/z* 581.2031 $[M + H]^+$; calculated $C_{29}H_{31}{}^{35}ClFN_6O_4^+$ 581.2001.

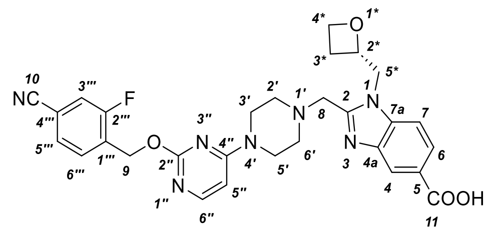

(S)-2-((4-(2-((4-cyano-2-fluorobenzyl)oxy)pyrimidin-4-yl)piperazin-1-yl)methyl)-1-(oxetan-2-ylmethyl)-1H-benzo[d]imidazole-5-carboxylic acid **12r'**, white solid, 40 mg, 85%, mp 184-185°C. **^{1}H NMR** (**400 MHz, DMSO-*d*$_6$, *δ ppm***): 12.70 (1H, *br.s.*, 11-COOH), 8.19 (1H, *d*, *J* = 1.5 Hz, 4-CH), 8.01 (1H, *d*, *J* = 5.6 Hz, 6''-CH), 7.92-7.83 (2H, *m*, 6-CH, 5'''-CH), 7.75-7.63 (3H, *m*, 6'''-CH, 7-CH, 3'''-CH), 6.51 (1H, *d*, *J* = 6.2 Hz, 5''-CH), 5.40 (2H, *s*, 9-CH_2), 5.11 (1H, *qd*, *J* = 7.1, 2.9 Hz, 2*-CH), 4.76 (1H, *dd*, *J* = 15.2, 7.2 Hz, 5*-CH_2), 4.63 (1H, *dd*, *J* = 15.2, 3.0 Hz, 5*-CH_2), 4.49 (1H, *td*, *J* = 8.0, 5.8 Hz, 4*-CH_2), 4.38 (1H, *dt*, *J* = 9.1, 5.9 Hz, 4*-CH_2), 3.96 (1H, *d*, *J* = 13.6 Hz, 8-CH_2), 3.82 (1H, *d*, *J* = 13.6 Hz, 8-CH_2), 3.59 (4H, *t*, *J* = 5.7 Hz,3'-CH_2, 5'-CH_2), 2.76-2.64 (1H, *m*, 3*-CH_2), 2.58-2.47 (4H, *m*, 2'-CH_2, 6'-CH_2), 2.47-2.34 (1H, *m*, 3*-CH_2). **^{13}C NMR** (**101 MHz, DMSO-*d*$_6$, *δ ppm***): 168.3 (C-11), 164.2 (C-4''), 163.5 (C-2''), 159.9 (C-2''', *d*, $J_{(C-F)}$ = 248.3 Hz), 158.7 (C-6''), 153.8 (C-2), 141.9 (C-4a), 139.7 (C-7a), 131.5 (C-5''', *d*, $J_{(C-F)}$ = 5.0 Hz), 131.2 (C-1''', *d*, $J_{(C-F)}$ = 14.6 Hz), 129.3 (C-6''', *d*, $J_{(C-F)}$ = 3.8 Hz), 124.8 (C-5), 124.0 (C-7), 121.1 (C-4), 119.7 (C-3''', *d*, $J_{(C-F)}$ = 25.3 Hz), 118.0 (C-10, *d*, $J_{(C-F)}$ = 2.3 Hz), 112.5 (C-4''', *d*, $J_{(C-F)}$ = 10.2 Hz), 111.4 (C-6), 98.6 (C-5''), 80.9 (C-2*), 68.0 (C-4*), 61.5 (C-9, *d*, $J_{(C-F)}$ = 3.1 Hz), 54.8 (C-8), 52.7 (C-2', C-6'), 49.5 (C-5*), 43.9 (C-3', C-5'), 24.8 (C-3*). **HRMS** (**ESI+**): found *m/z* 558.2221 $[M + H]^+$; calculated $C_{29}H_{29}FN_7O_4^+$ 558.2187.

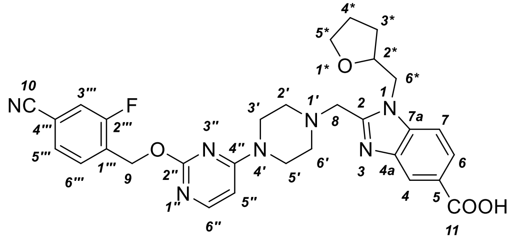

2-((4-(2-((4-cyano-2-fluorobenzyl)oxy)pyrimidin-4-yl)piperazin-1-yl)methyl)-1-((tetrahydrofuran-2-yl)methyl)-1H-benzo[d]imidazole-5-carboxylic acid **12s'**, white solid, 75 mg, 80%, mp 213-214°C. **^{1}H NMR (400 MHz, DMSO-d_6, *δ ppm*)**: 12.63 (1H, br.s., 11-COOH), 8.18 (1H, *br.s.*, 4-CH), 8.01 (1H, *d*, *J* = 6.1 Hz, 6''-CH), 7.87 (2H, *t*, *J* = 9.4 Hz, 6-CH, 3'''-CH), 7.72-7.65 (3H, *m*, 6'''-CH, 7-CH, 5'''-CH), 6.50 (1H, *d*, *J* = 5.6 Hz, 5''-CH), 5.40 (2H, *s*, 9-CH_2), 4.52 (2H, *dd*, *J* = 14.8, 3.2 Hz, 6^*-CH_2), 4.42 (1H, *dd*, *J* = 14.8, 7.9 Hz, 2^*-CH), 3.99 (1H, *d*, *J* = 13.6 Hz, 8-CH), 3.86-3.73 (2H, *dd*, *J* = 14.8, 3.2 Hz, 8-CH, 5^*-CH_2), 3.64 (1H, *dd*, *J* = 7.2 Hz, 8-CH, 5^*-CH_2), 3.60-3.57 (4H, *m*, 3'-CH_2, 5'-CH_2), 2.60-2.52 (4H, *m*, 2'-CH_2, 6'-CH_2), 2.05 (1H, *dd*, *J* = 12.2, 6.5 Hz,, 3^*-CH_2), 1.94-1.73 (2H, *m*, 4^*-CH_2), 1.67-1.59 (1H, *m*, 3^*-CH_2). **^{13}C NMR (101 MHz, DMSO-d_6, *δ ppm*)**: 168.3 (C-11), 164.2 (C-4''), 163.5 (C-2''), 159.9 (C-2''', *d*, $J_{(C\text{-}F)}$ = 248.6 Hz), 157.8 (C-6''), 153.7 (C-2), 141.8 (C-4a), 139.7 (C-7a), 131.5 (C-6''', *d*, $J_{(C\text{-}F)}$ = 4.9 Hz), 131.2 (C-1''', *d*, $J_{(C\text{-}F)}$ = 14.6 Hz), 129.3 (C-5''', *d*, $J_{(C\text{-}F)}$ = 3.7 Hz), 124.8 (C-5), 124.7 (C-6), 121.1 (C-4), 119.7 (C-3''', *d*, $J_{(C\text{-}F)}$ = 25.4 Hz), 118.0 (C-10, *d*, $J_{(C\text{-}F)}$ = 2.6 Hz), 112.5 (C-4''', *d*, $J_{(C\text{-}F)}$ = 10.1 Hz), 111.3 (C-7), 98.6 (C-5''), 78.2 (C-2^*), 67.8 (C-5^*), 61.5 (C-9, *d*, $J_{(C\text{-}F)}$ = 3.8 Hz), 54.9 (C-8), 52.7 (C-2', C-6'), 48.2 (C-6^*), 43.9 (C-3', C-5'), 29.1 (C-3^*), 25.7 (C-4^*). **HRMS (ESI+)**: found *m/z* 572.2365 $[M + H]^+$; calculated $C_{30}H_{31}FN_7O_4^+$ 572.2343.

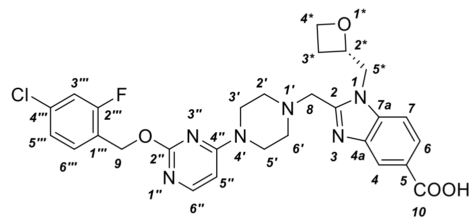

(S)-2-((4-(2-((4-chloro-2-fluorobenzyl)oxy)pyrimidin-4-yl)piperazin-1-yl)methyl)-1-(oxetan-2-ylmethyl)-1H-benzo[d]imidazole-5-carboxylic acid **12z'**, white solid, 80 mg, 80%, mp 56-57°C. **^{1}H NMR (400 MHz, DMSO-d_6, *δ ppm*)**: 8.18 (1H, *br.s.*, 4-CH), 7.86 (1H, *d*, *J* = 8.5 Hz, 6-CH), 7.81 (1H, *d*, *J* = 7.5 Hz, 6''-CH), 7.71 (1H, *d*, *J* = 8.6 Hz, 7-CH), 7.44 (1H, *dd*, *J* = 10.1, 2.0 Hz, 3'''-CH), 7.28 (1H, *dd*, *J* = 8.3, 2.0 Hz, 5'''-CH),7.22 (1H, *t*, *J* = 8.1 Hz, 6'''-CH), 6.18 (1H, *d*, *J* = 7.6 Hz, 5''-CH), 5.10 (1H, *qd*, *J* = 7.2, 2.8 Hz, 2^*-CH), 4.90 (2H, *s*, 9-CH_2), 4.75 (1H, *dd*, *J* = 15.3, 7.2 Hz, 5^*-CH_2), 4.62 (1H, *dd*, *J* = 15.2, 3.0 Hz, 5^*-CH_2), 4.55-4.43 (1H, *m*, 4^*-CH_2), 4.38 (1H, *dt*, *J* = 9.0, 5.9 Hz, 4^*-CH_2), 3.95 (1H, *d*, *J* = 13.6 Hz, 8-CH_2), 3.82 (1H, *d*, *J* = 13.6 Hz, 8-CH_2), 3.29-3.24 (4H, *m*, 3'-CH_2, 5'-CH_2), 2.72-2.66 (1H, *m*, 3^*-CH_2), 2.61-2.53 (4H, *m*, 2'-CH_2, 6'-CH_2), 2.44-2.37 (1H, *m*, 3^*-CH_2). **^{13}C NMR (101 MHz, DMSO-d_6, *δ ppm*)**: 163.3 (C-4''), 160.5 (C-2''', *d*, $J_{(C\text{-}F)}$ = 249.1 Hz), 155.3 (C-2''), 153.6 (C-2), 147.3 (C-6''), 141.9 (C-4a), 139.6 (C-7a), 133.3 (C-4''', *d*, $J_{(C\text{-}F)}$ = 10.3 Hz), 131.6 (C-6''', *d*, $J_{(C\text{-}F)}$ = 5.2 Hz), 125.1 (C-5''', *d*, $J_{(C\text{-}F)}$ = 3.4 Hz), 124.1 (C-1''', *d*, $J_{(C\text{-}F)}$ = 15.0 Hz), 124.0 (C-6), 121.0 (C-4), 116.4 (C-3''', *d*, $J_{(C\text{-}F)}$ = 24.8 Hz), 111.3 (C-7), 91.6 (C-5''), 80.9 (C-2^*), 68.0 (C-4^*), 54.7 (C-8), 52.9 (C-2', C-6'), 49.5 (C-5^*), 46.7 (C-3', C-5'), 46.2 (C-9, *m*), 24.9 (C-3^*). **HRMS (ESI+)**: found *m/z* 567.1884 $[M + H]^+$; calculated $C_{28}H_{29}{}^{35}ClFN_6O_4^+$ 567.1845.

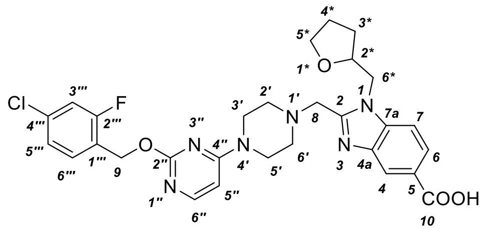

2-((4-(2-((4-chloro-2-fluorobenzyl)oxy)pyrimidin-4-yl)piperazin-1-yl)methyl)-1-((tetrahydrofuran-2-yl)methyl)-1H-benzo[d]imidazole-5-carboxylic acid **12aa'**, white solid, 95 mg, 90%, mp 204-205°C. **^{1}H NMR (400 MHz, DMSO-d_6, *δ ppm*)**: 12.70 (1H, *br.s.*, 10-COOH), 8.18 (1H, *d*, *J* = 1.5 Hz, 4-CH), 8.02 (1H, *d*, *J* = 5.6 Hz, 6''-CH), 7.86 (2H, *dd*, *J* = 8.5, 1.6 Hz, 6-CH), 7.68 (1H, *d*, *J* = 8.6 Hz, 7-CH), 7.53 (1H, *t*, *J* = 8.2 Hz, 6'''-CH), 7.47 (1H, *dt*, *J* = 10.0, 2.1 Hz, 3'''-CH), 7.31 (1H, *dd*, *J* = 8.2, 2.0 Hz, 5'''-CH), 6.50 (1H, *d*, *J* = 6.2 Hz, 5''-CH), 5.30 (2H, *s*, 9-CH_2), 4.52 (1H, *dd*, *J* = 14.9, 3.2 Hz, 6*-CH_2), 4.42 (1H, *dd*, *J* = 14.8, 8.0 Hz, 6*-CH), 4.25 (1H, *qd*, *J* = 7.2, 3.1 Hz, 2^*-CH), 3.99 (1H, *d*, *J* = 13.6 Hz, 8-CH_2), 3.85-3.74 (2H, *m*, 8-CH, 5*-CH), 3.62-3.59 (5H, *m*, 5*-CH, 3'-CH_2, 5'-CH_2), 2.60-2.46 (4H, *m*, 2'-CH_2, 4'-CH_2), 2.10-1.99 (1H, *m*, 3^*-CH_2), 1.95-1.74 (2H, *m*, 4^*-CH_2), 1.70-1.56 (1H, *m*, 3^*-CH_2). **^{13}C NMR (101 MHz, DMSO-d_6, *δ ppm*)**: 168.3 (C-10), 164.3 (C-2''), 163.5 (C-4''), 160.7 (C-2''', *d*, $J_{(C\text{-}F)}$ = 249.9 Hz), 157.8 (C-6''), 153.7 (C-2), 141.8 (C-4a), 139.7 (C-7a), 134.0 (C-4''', *d*, $J_{(C\text{-}F)}$ = 10.2 Hz), 132.3 (C-6''', *d*, $J_{(C\text{-}F)}$ = 5.1 Hz), 125.2 (C-5''', *d*, $J_{(C\text{-}F)}$ = 3.4 Hz), 124.6 (C-5), 123.9 (C-1''', *d*, $J_{(C\text{-}F)}$ = 15.3 Hz), 123.8 (C-6), 121.0 (C-4), 116.4 (C-3''', *d*, $J_{(C\text{-}F)}$ = 25.0 Hz), 111.4 (C-7), 98.5 (C-5''), 78.2 (C-2^*), 67.8 (C-5^*), 61.5 (C-9, *d*, $J_{(C\text{-}F)}$ = 3.4 Hz), 54.9 (C-8), 52.7 (C-2', C-6'), 48.2 (C-6^*), 43.9 (C-3', C-5'), 29.2 (C-3^*), 25.7 (C-4^*). **HRMS (ESI+)**: found *m/z* 581.2028 $[M + H]^+$; calculated $C_{29}H_{31}{}^{35}ClFN_6O_4^+$ 581.2001.

^{1}H NMR spectrum of compound **12a**

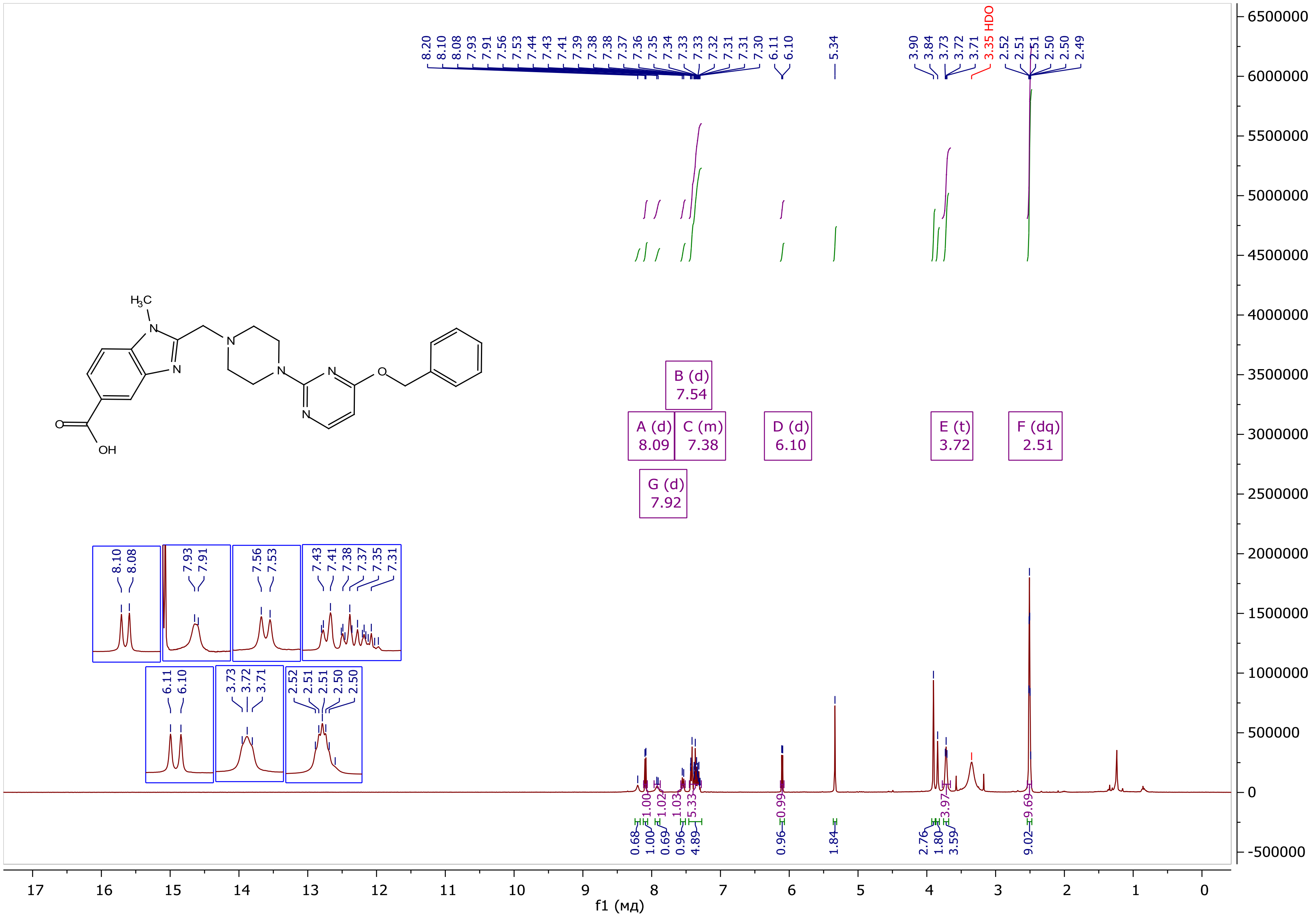

^{13}C NMR spectrum of compound **12a**

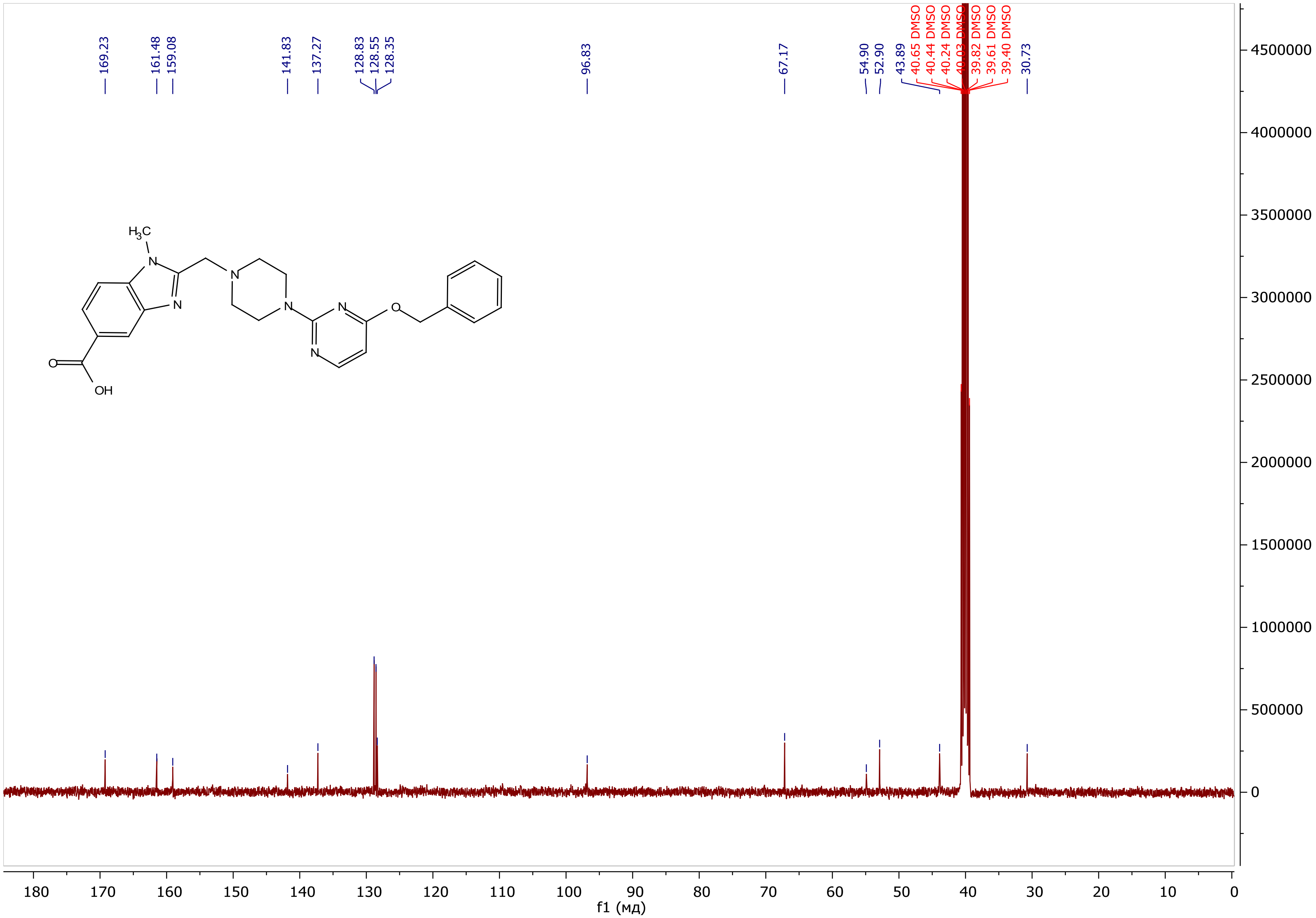

^{1}H NMR spectrum of compound **12b**

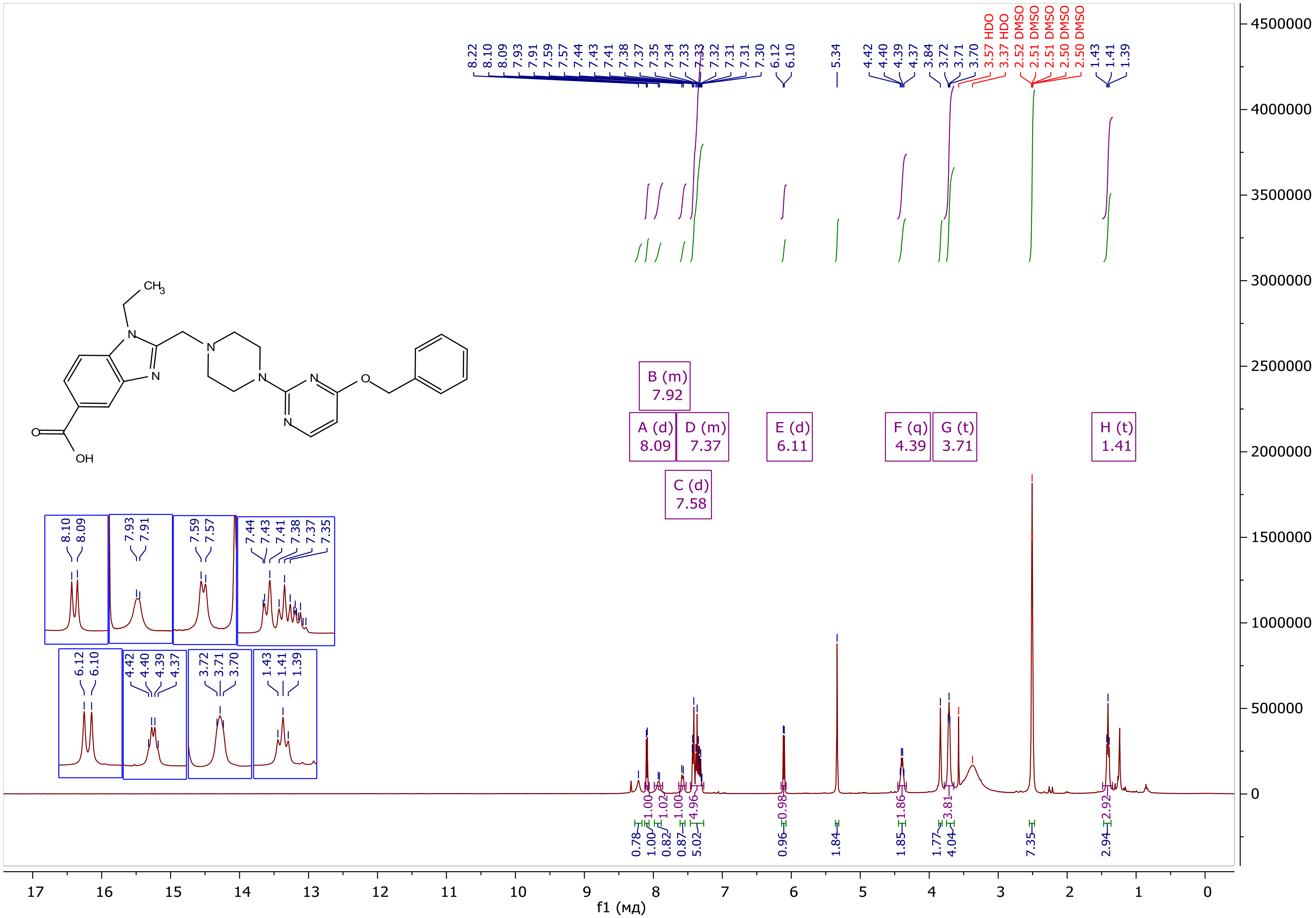

^{13}C NMR spectrum of compound **12b**

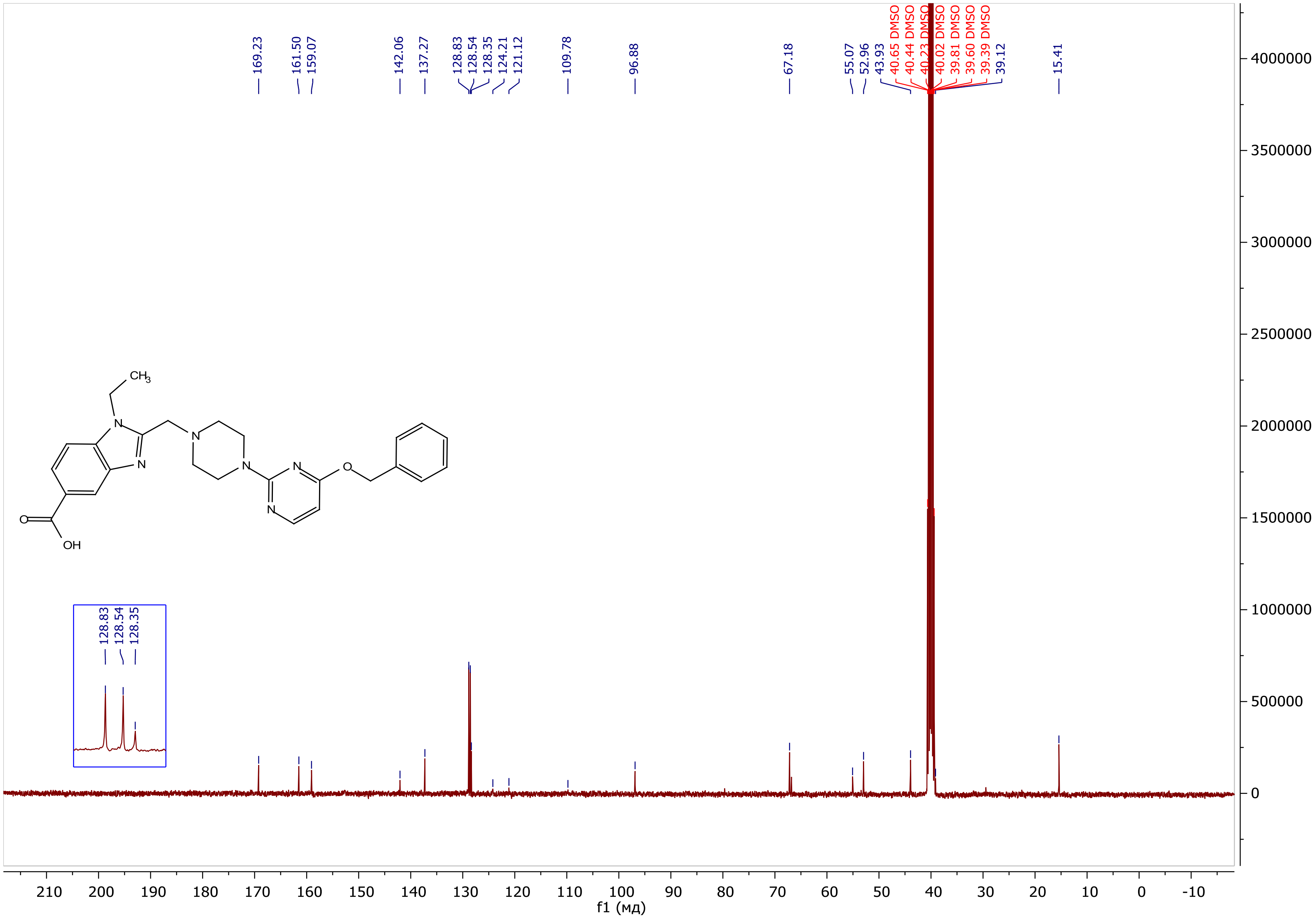

^{1}H NMR spectrum of compound **12c**

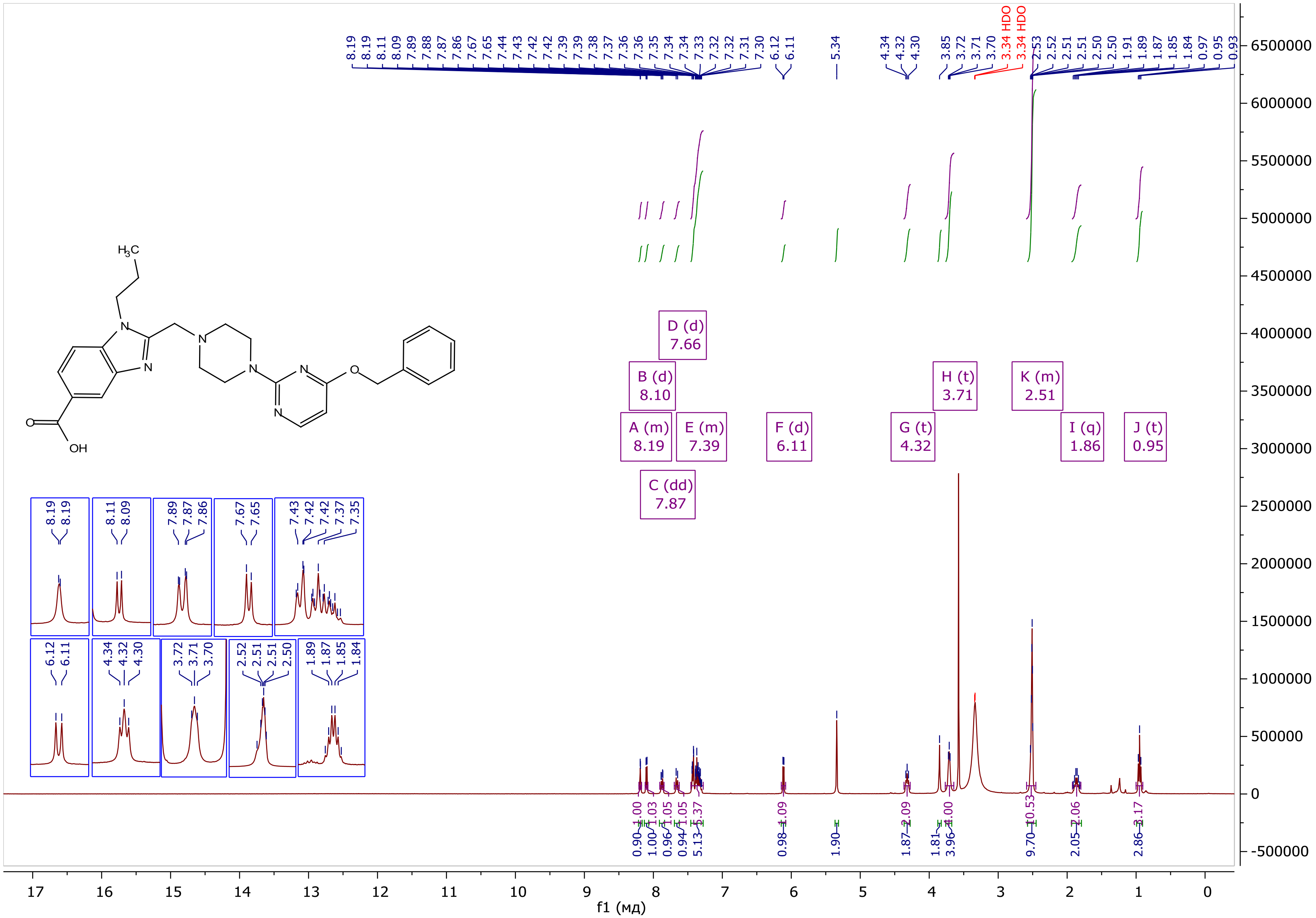

^{13}C NMR spectrum of compound **12c**

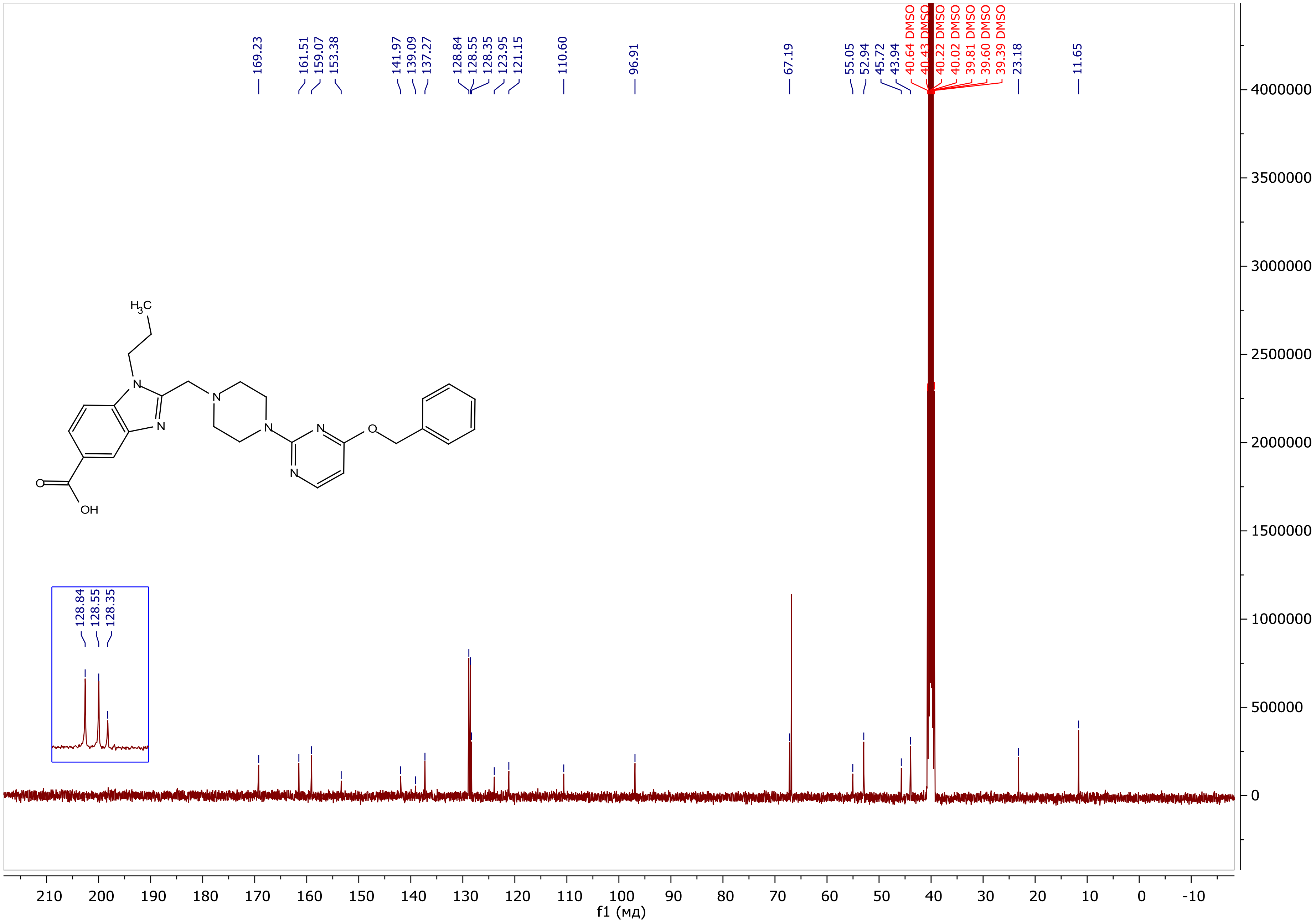

^{1}H NMR spectrum of compound **12d**

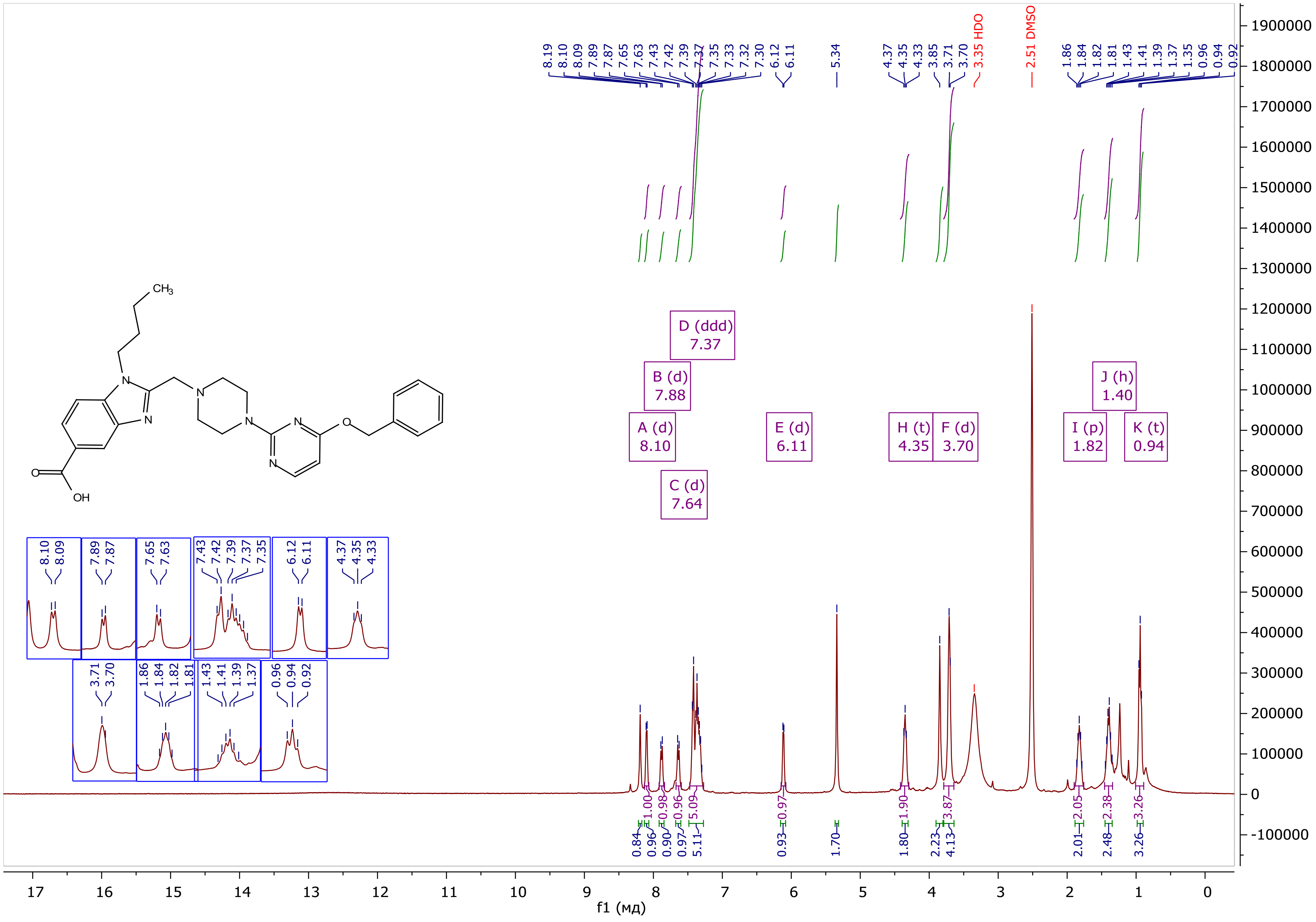

^{13}C NMR spectrum of compound **12d**

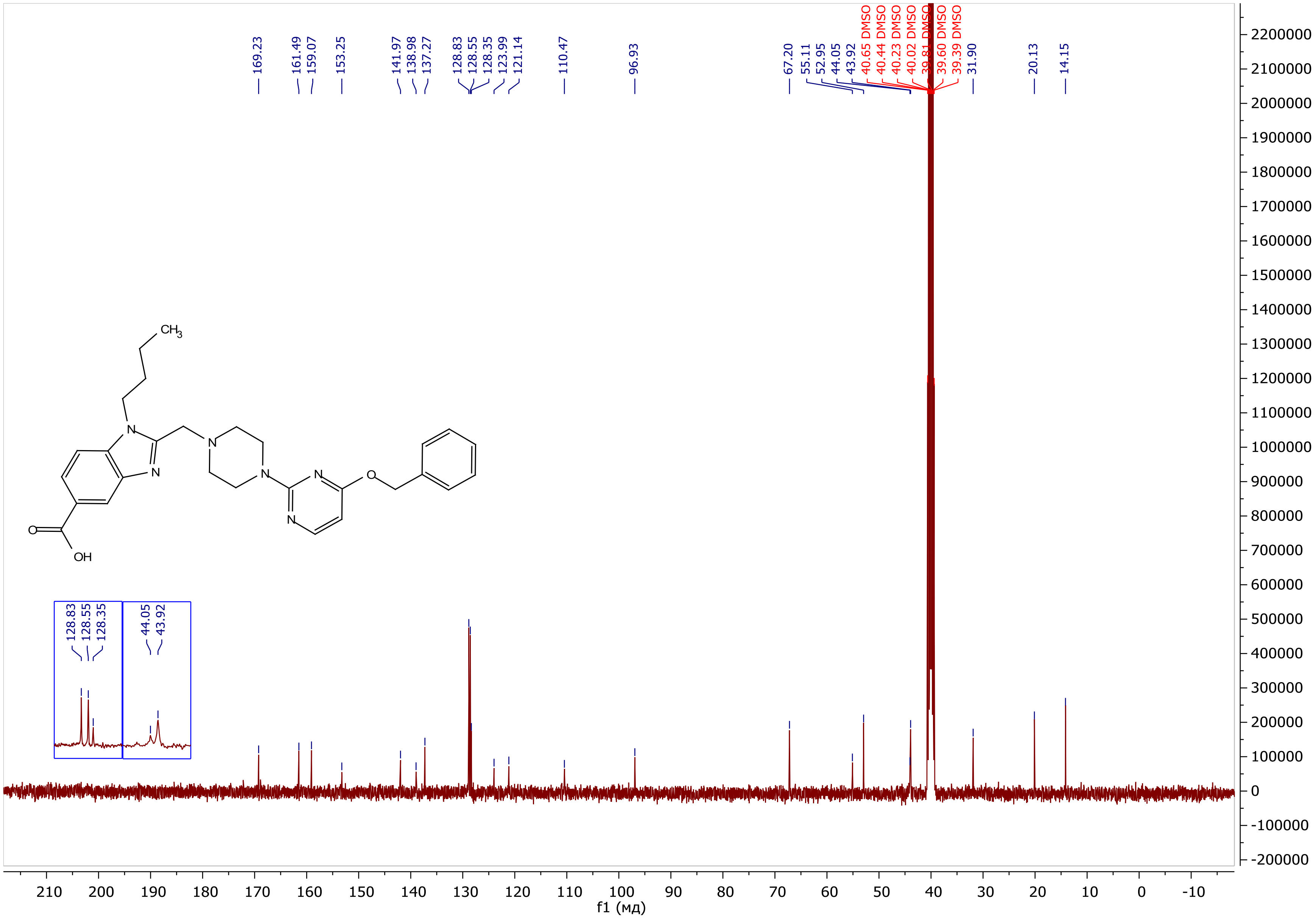

^{1}H NMR spectrum of compound **12e**

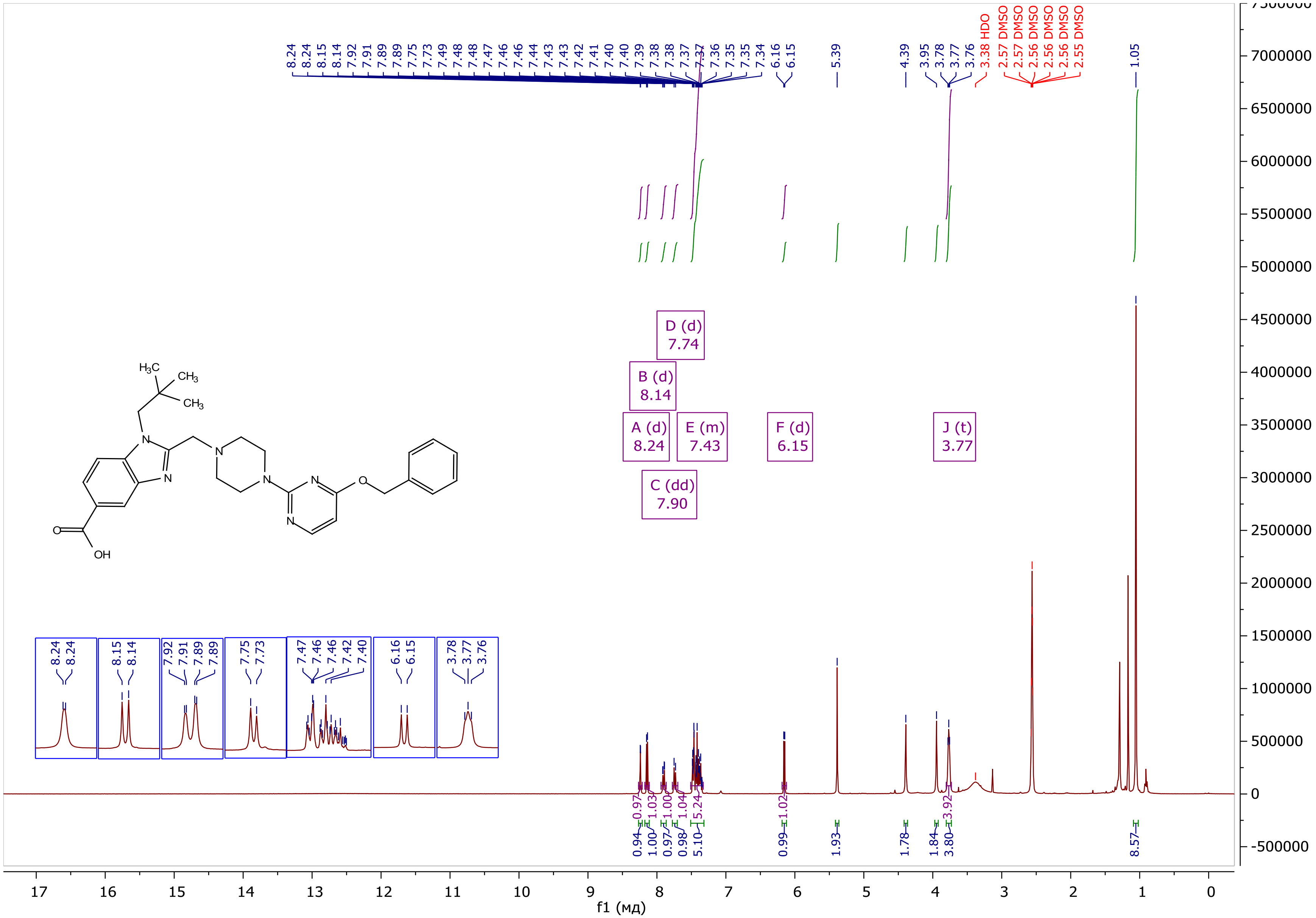

^{13}C NMR spectrum of compound **12e**

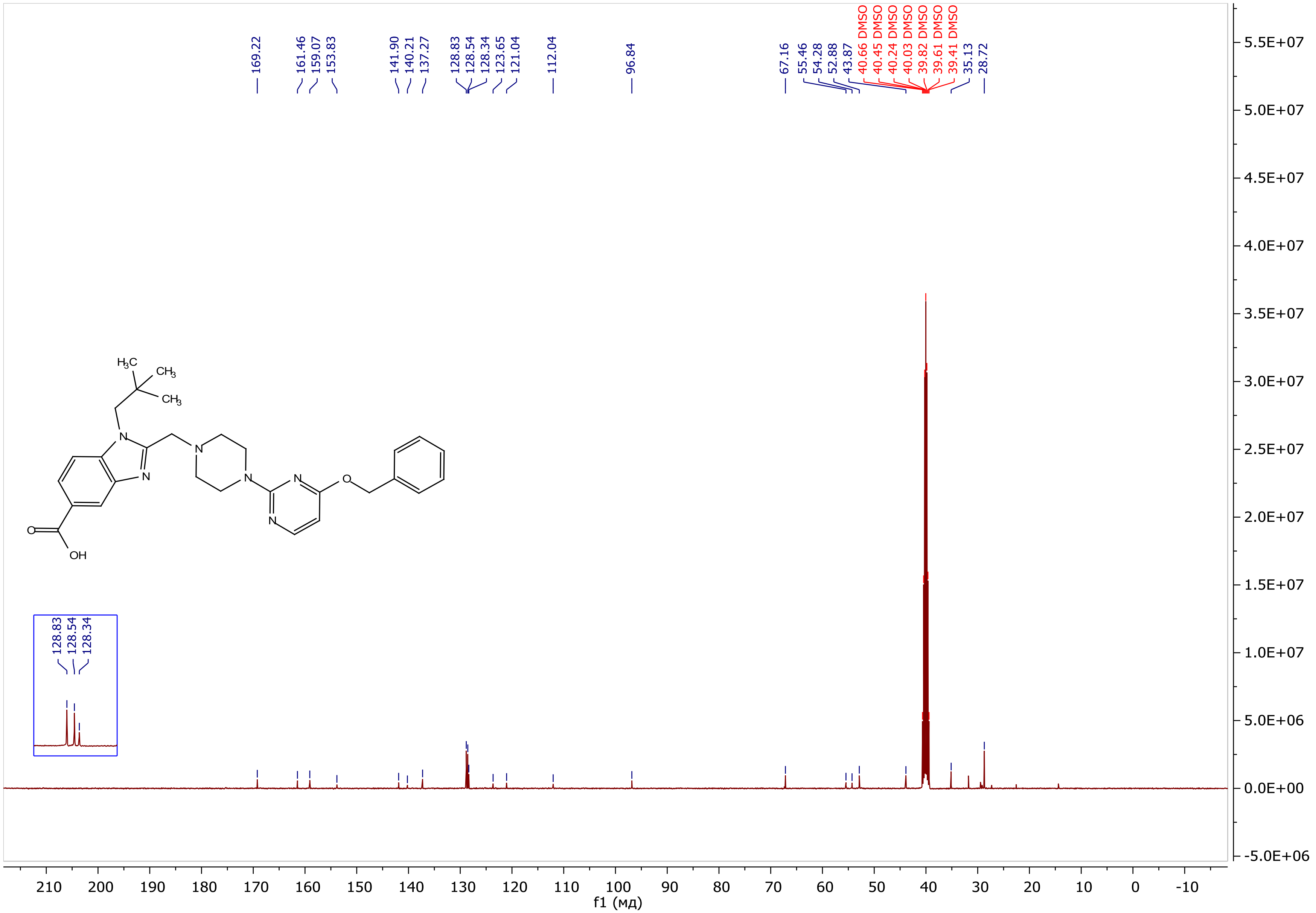

^{1}H NMR spectrum of compound **12f**

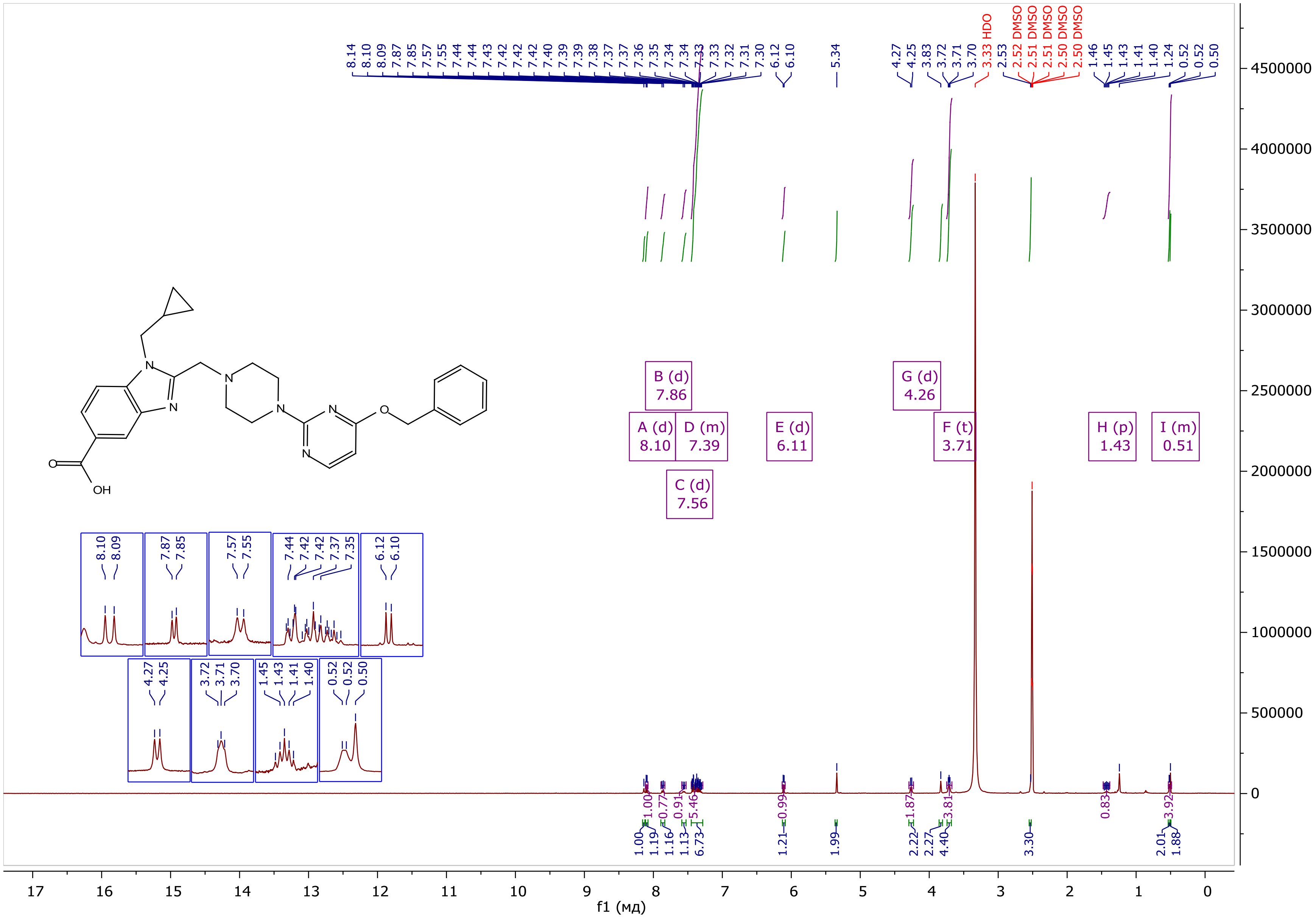

^{1}H NMR spectrum of compound **12i**

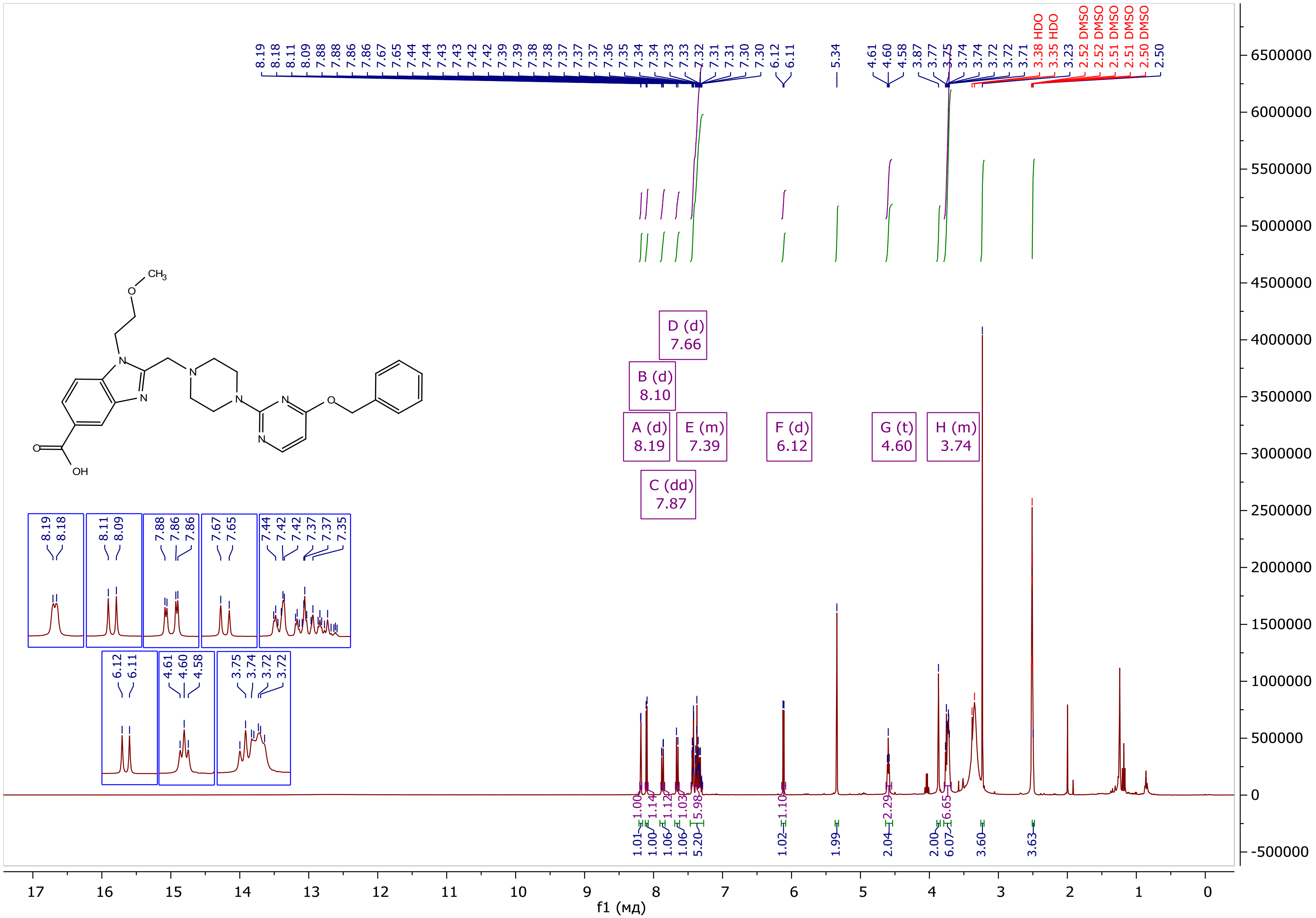

^{13}C NMR spectrum of compound **12i**

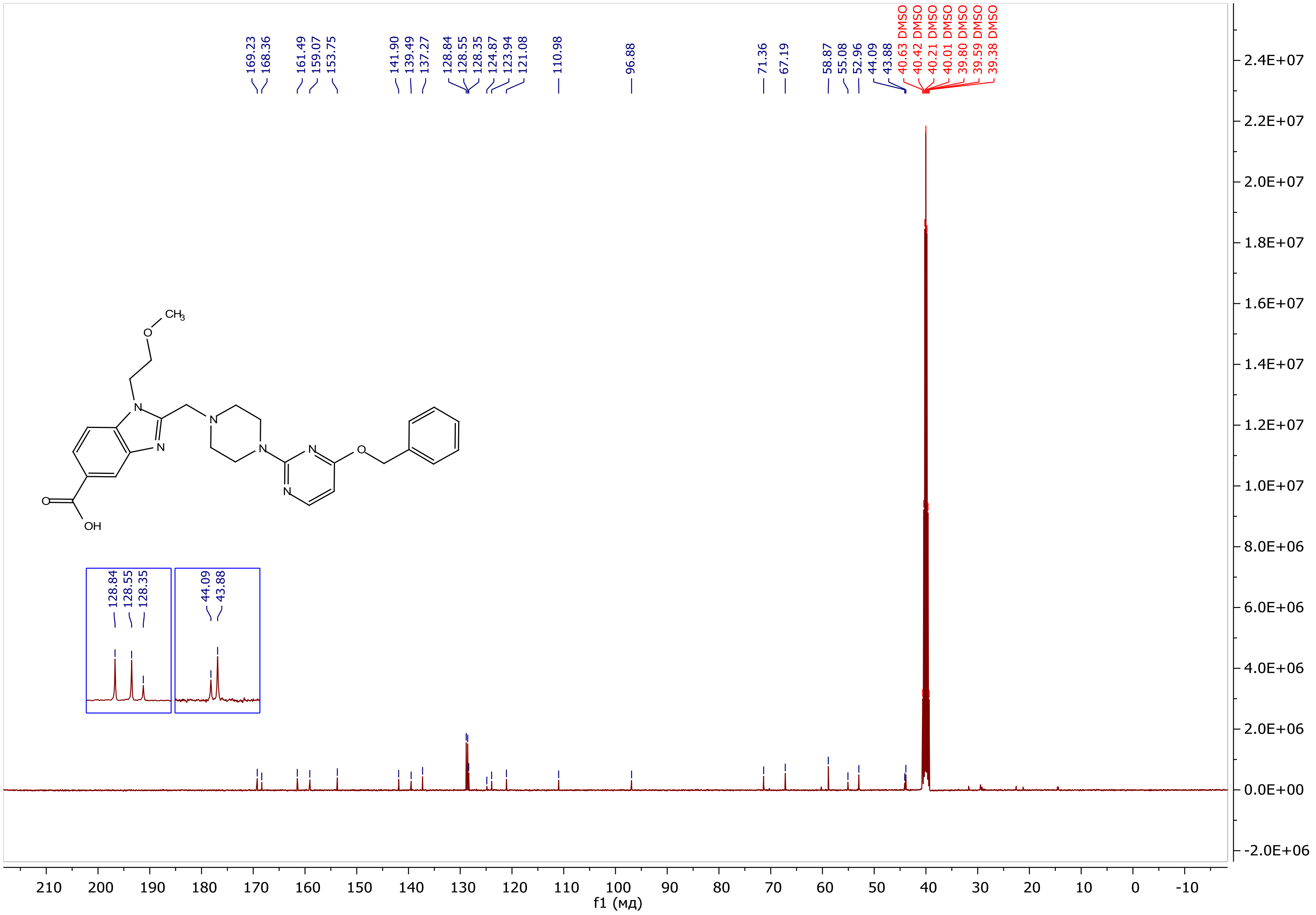

^{1}H NMR spectrum of compound **12j**

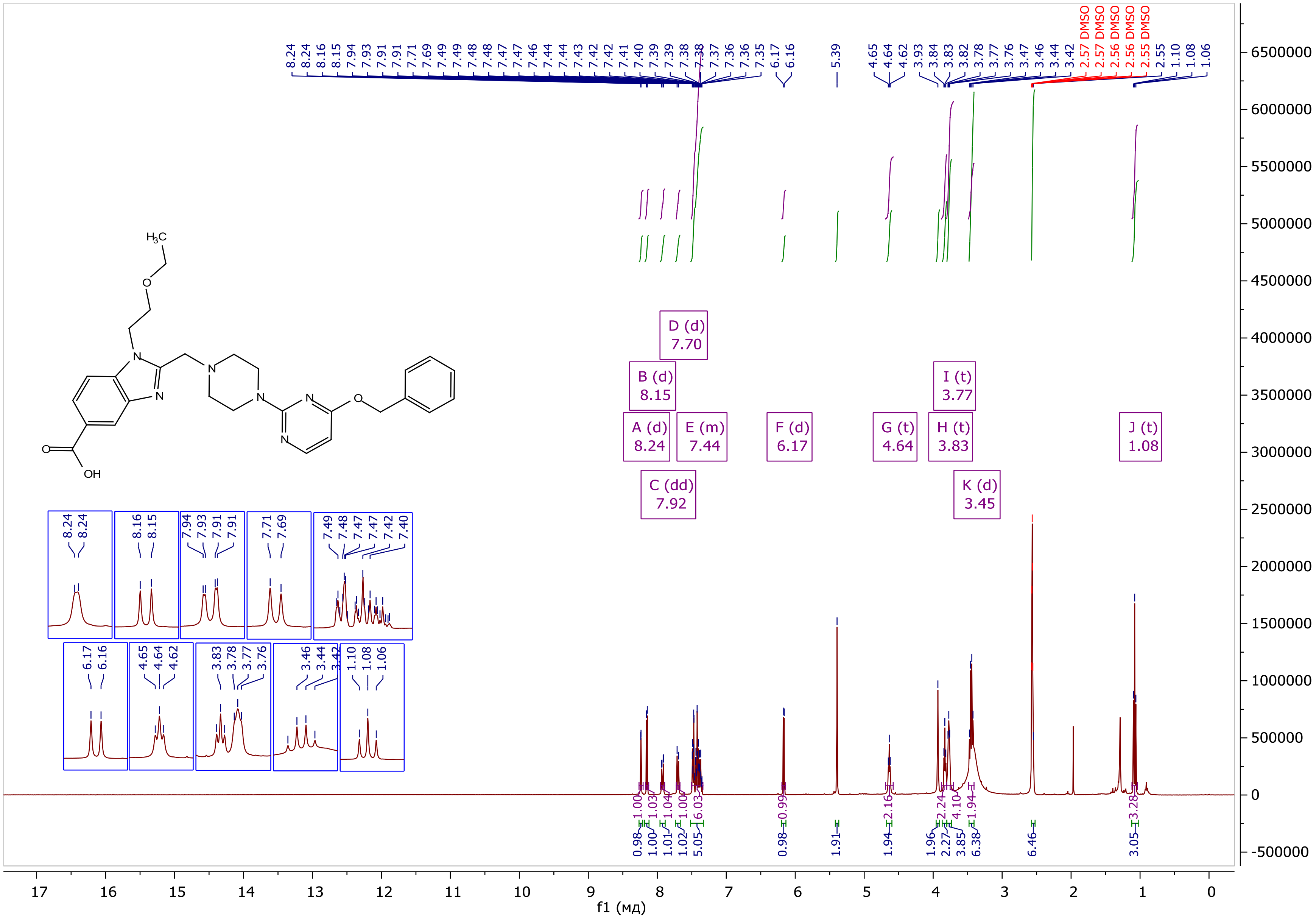

^{13}C NMR spectrum of compound **12j**

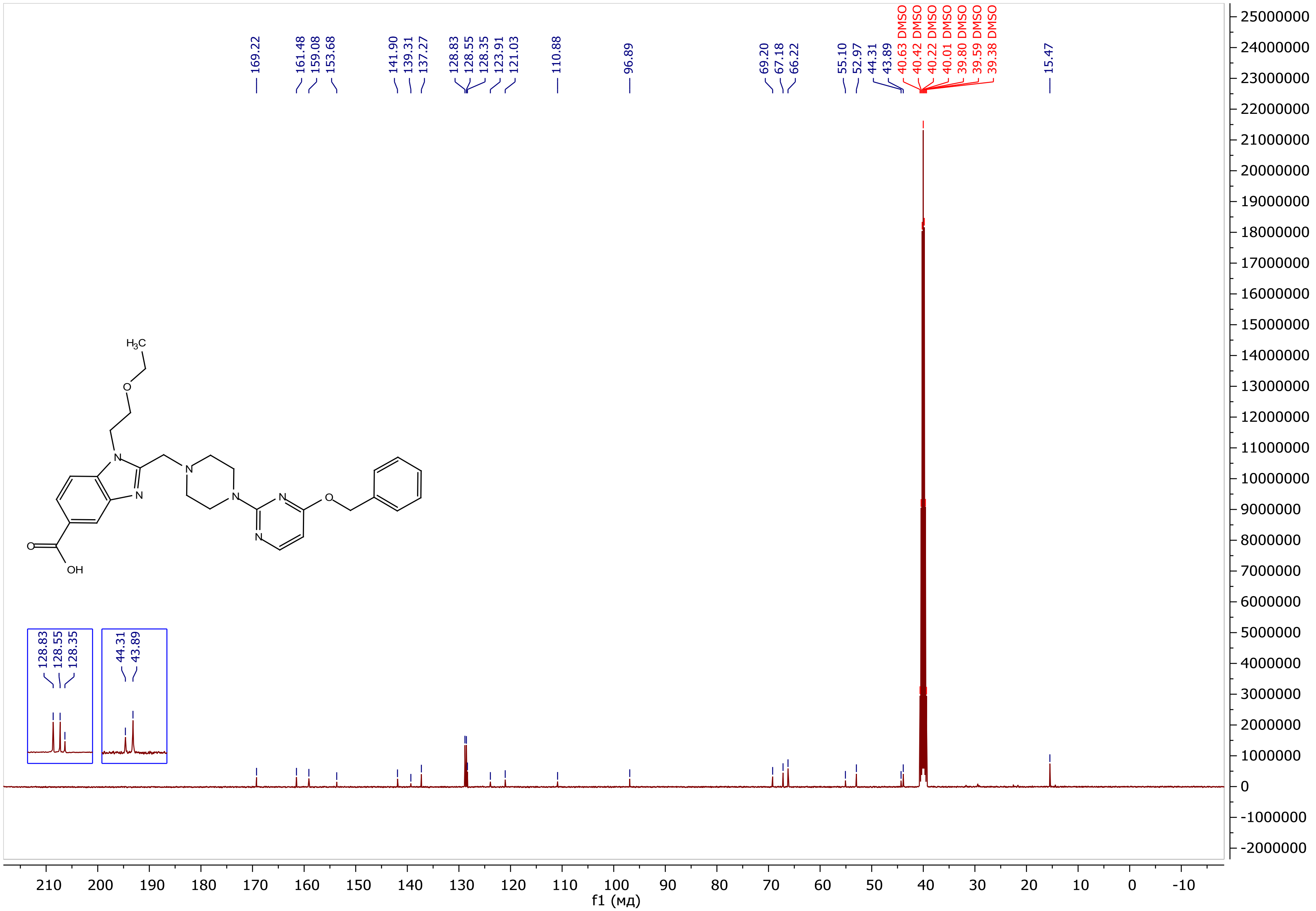

^{1}H NMR spectrum of compound **12k**

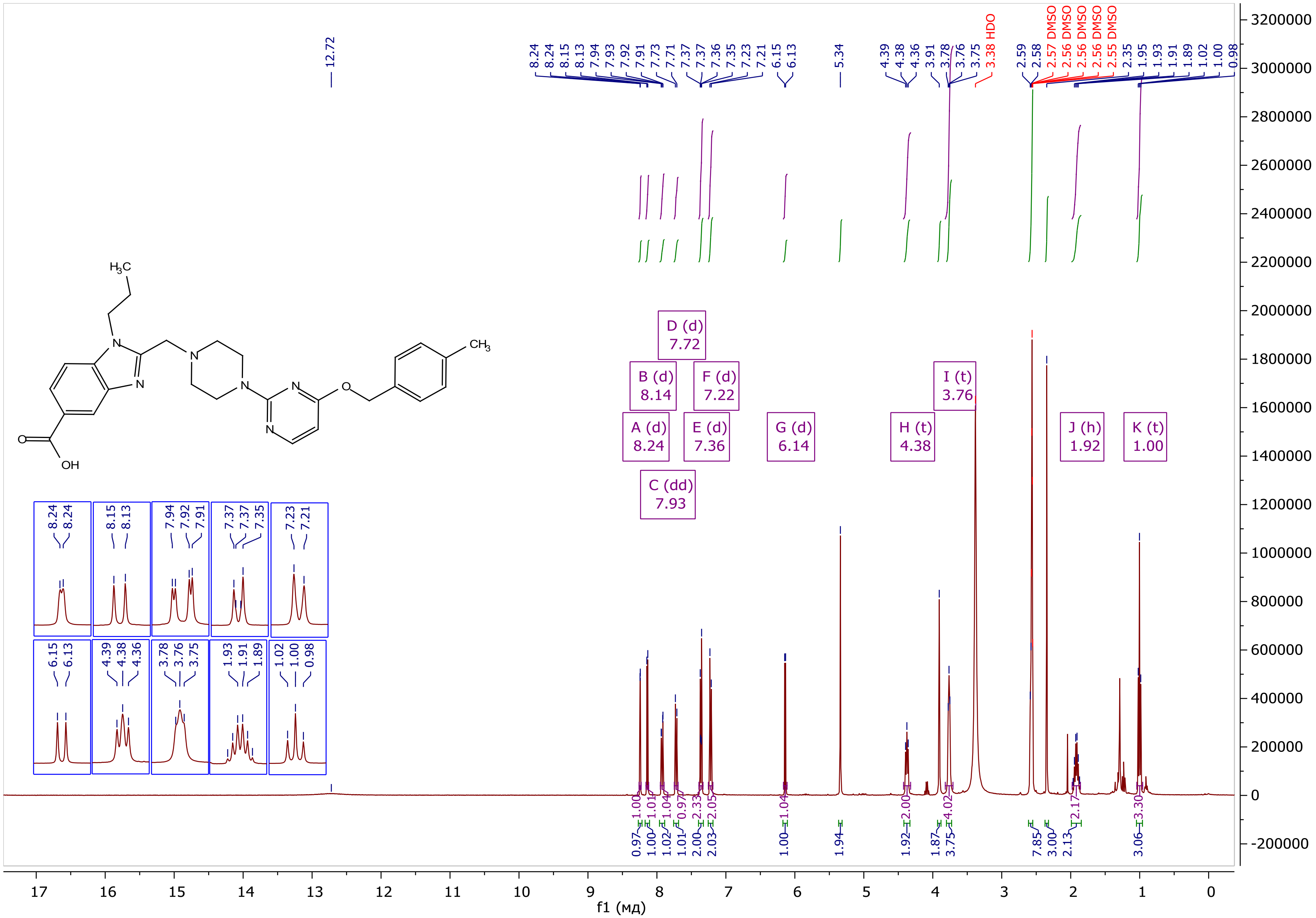

^{13}C NMR spectrum of compound **12k**

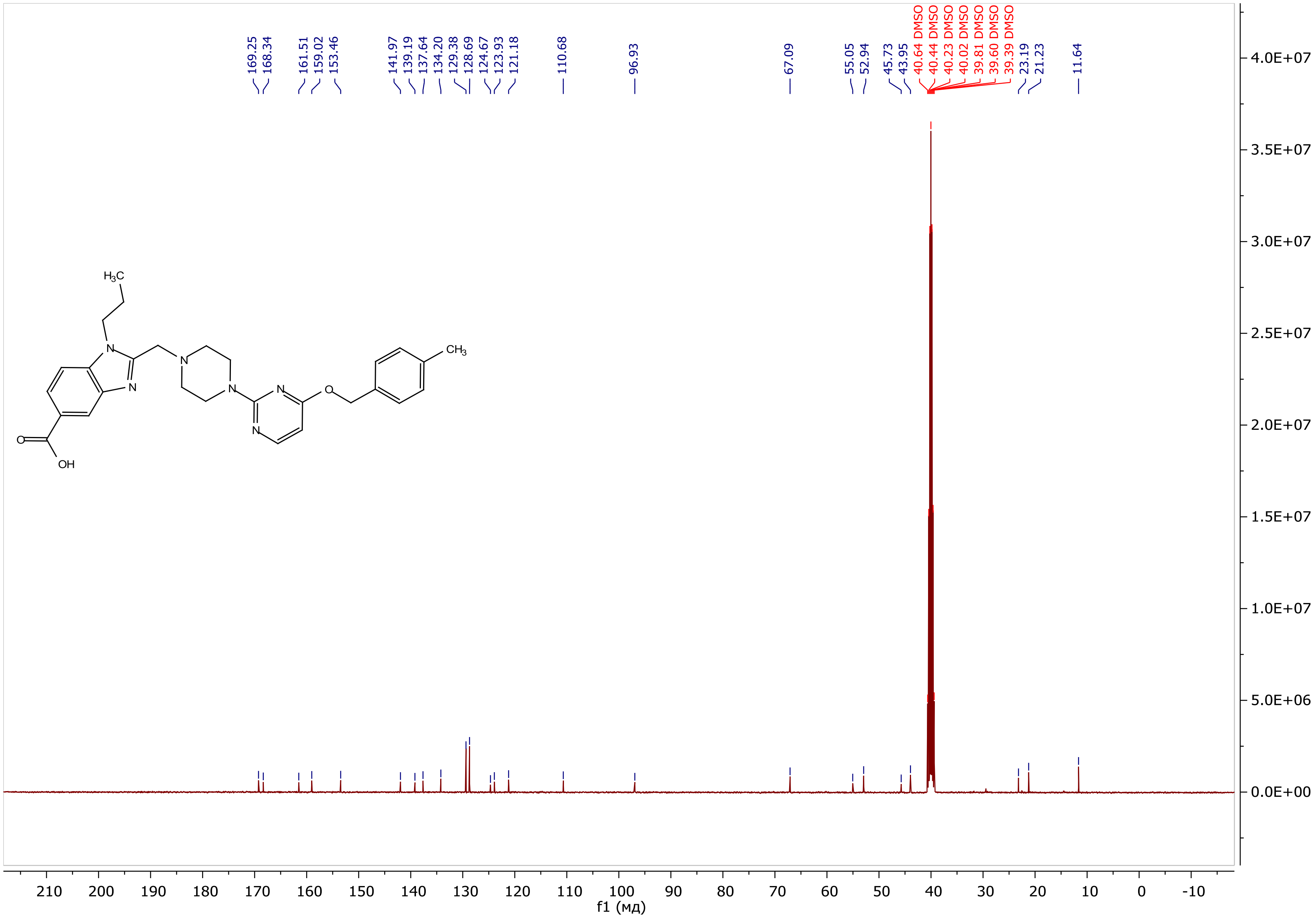

^{1}H NMR spectrum of compound **12m**

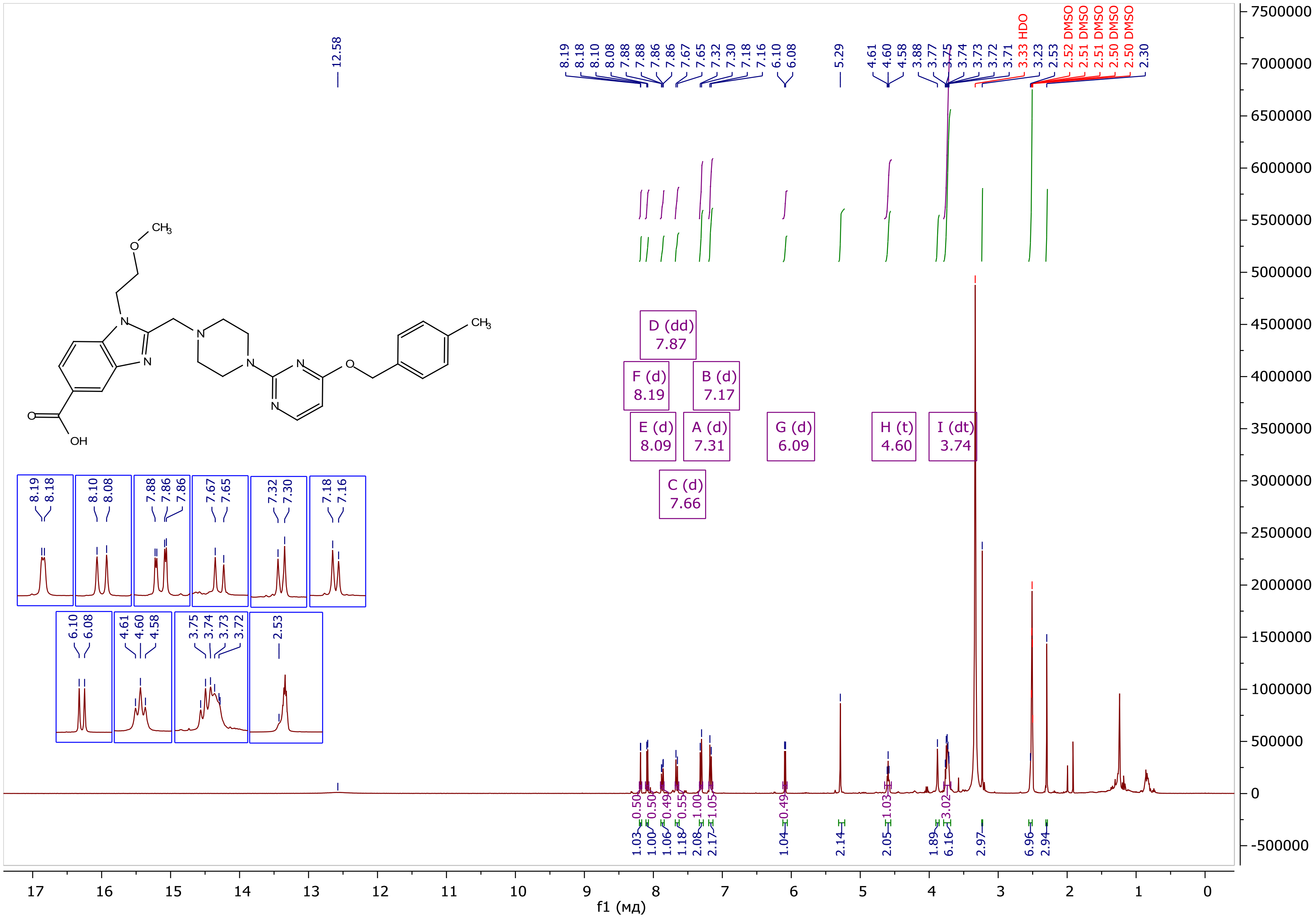

^{13}C NMR spectrum of compound **12m**

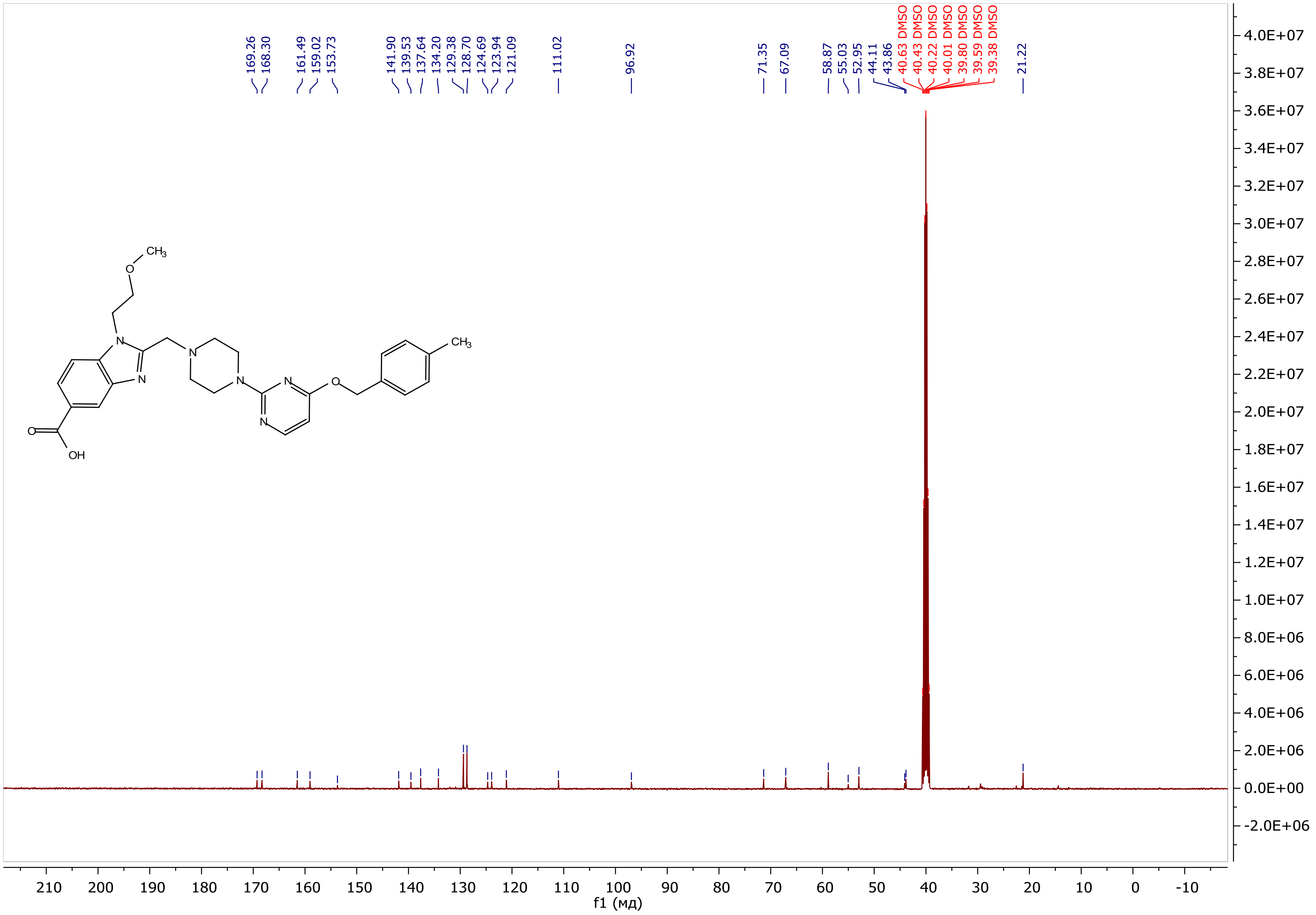

^{1}H NMR spectrum of compound **12n**

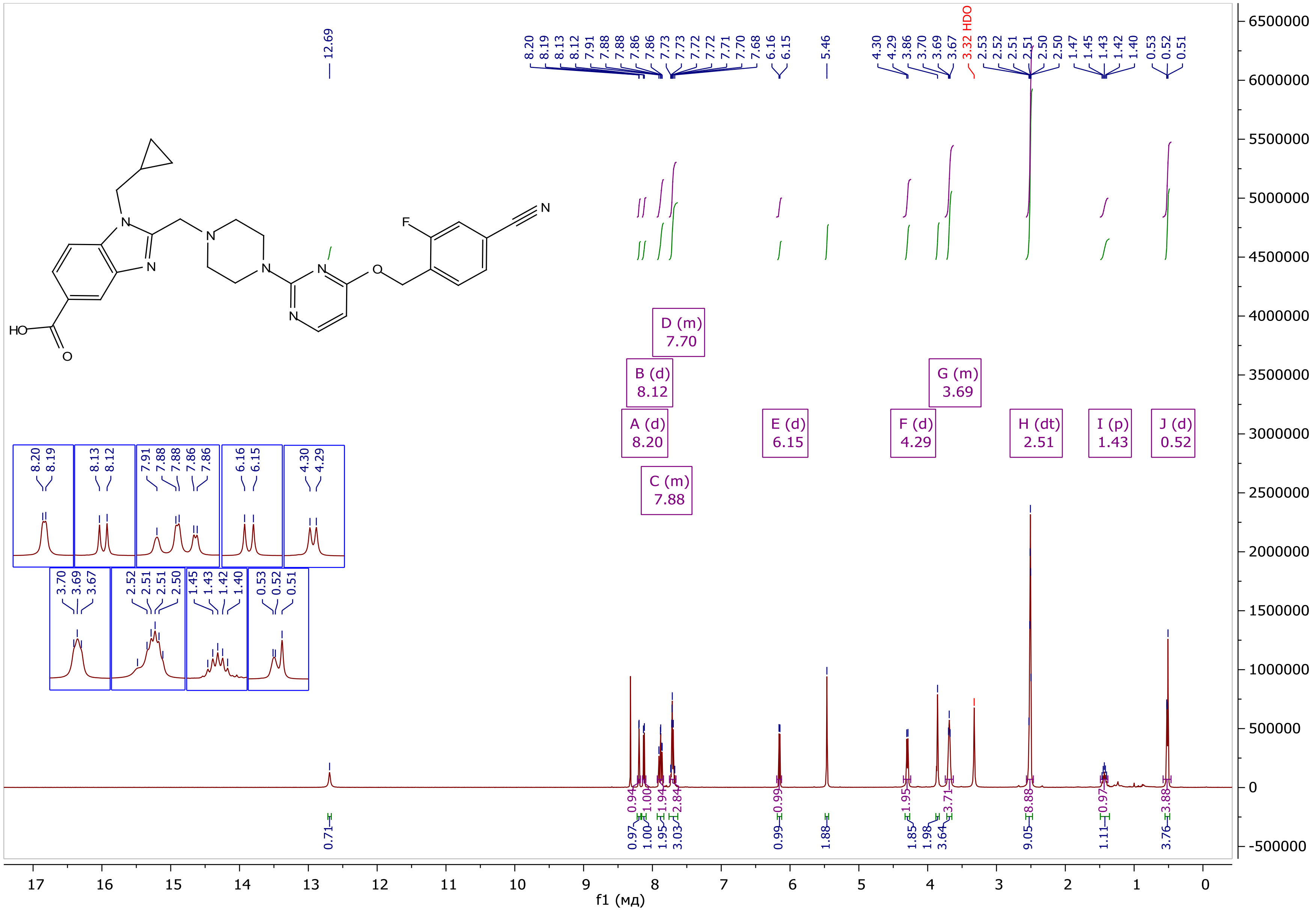

^{13}C NMR spectrum of compound **12n**

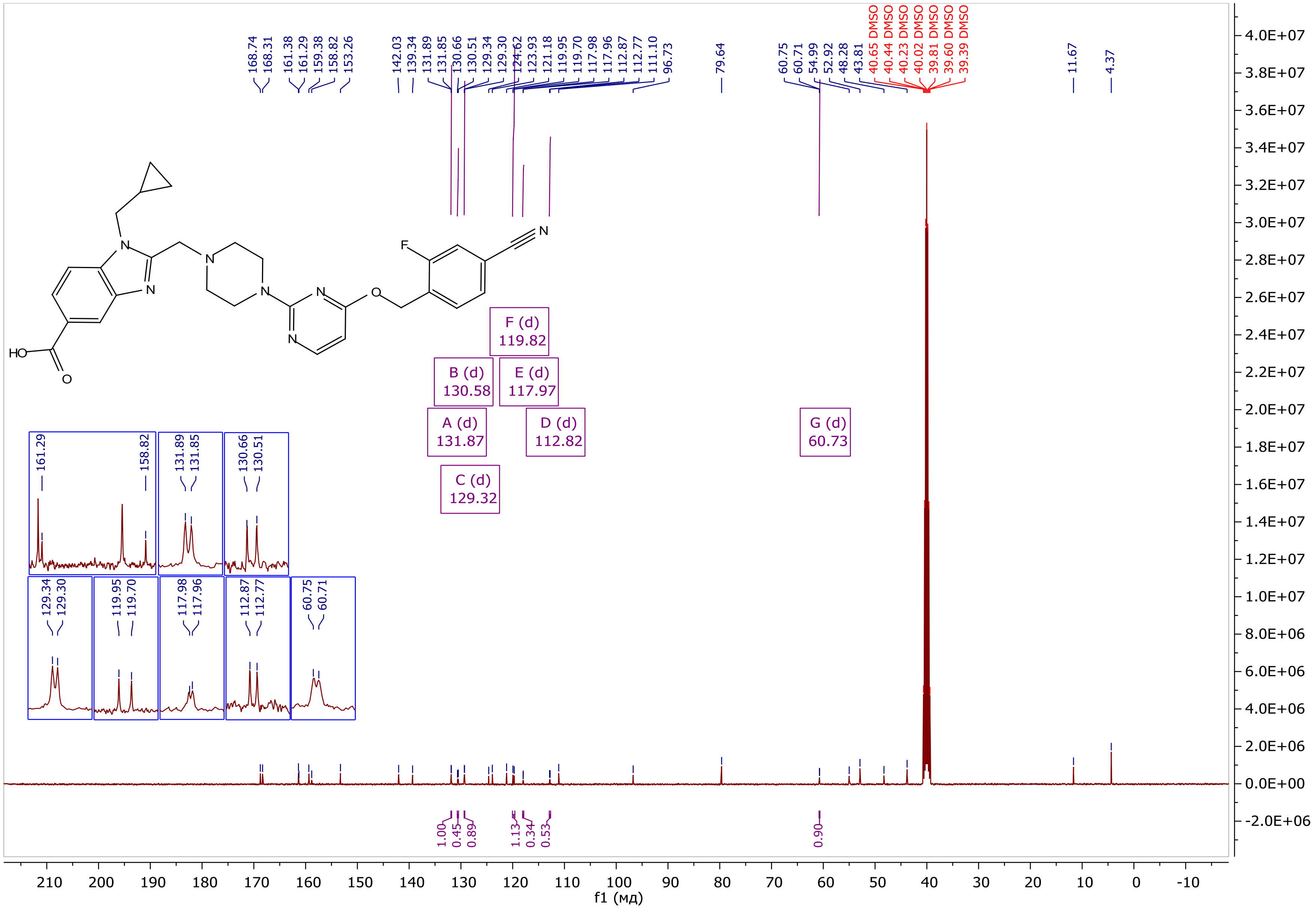

^{1}H NMR spectrum of compound **12o**

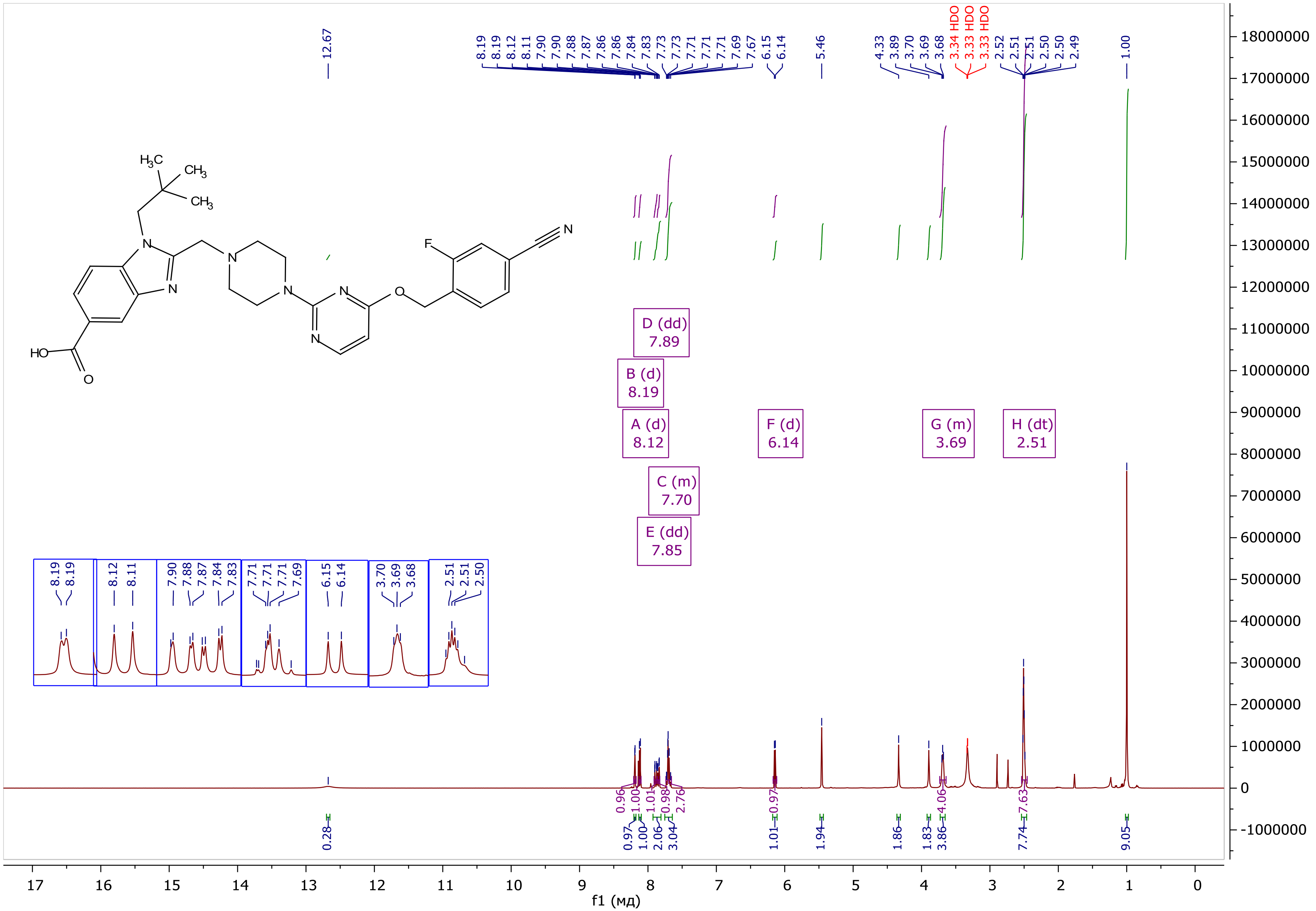

^{13}C NMR spectrum of compound **12o**

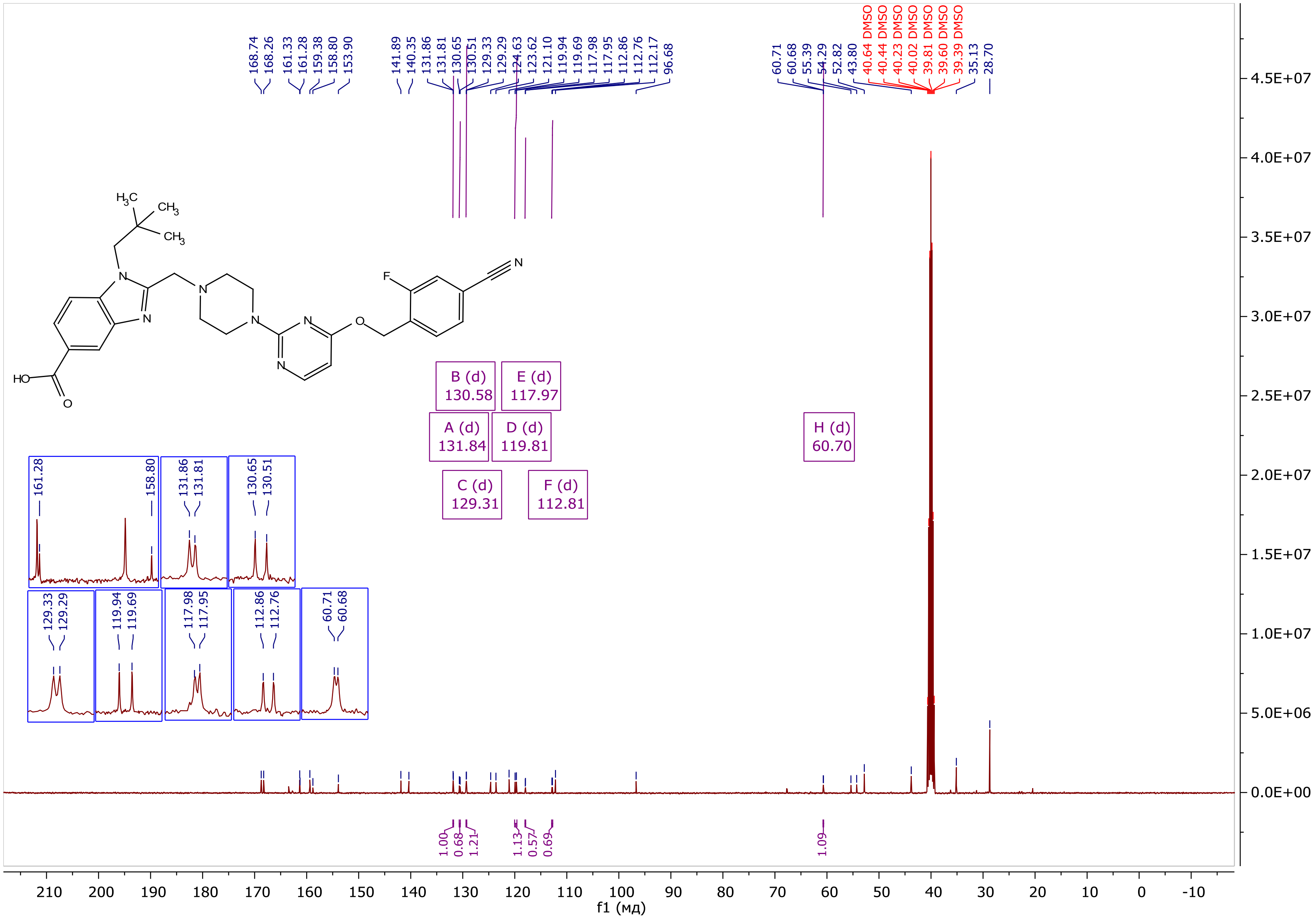

^{1}H NMR spectrum of compound **12p**

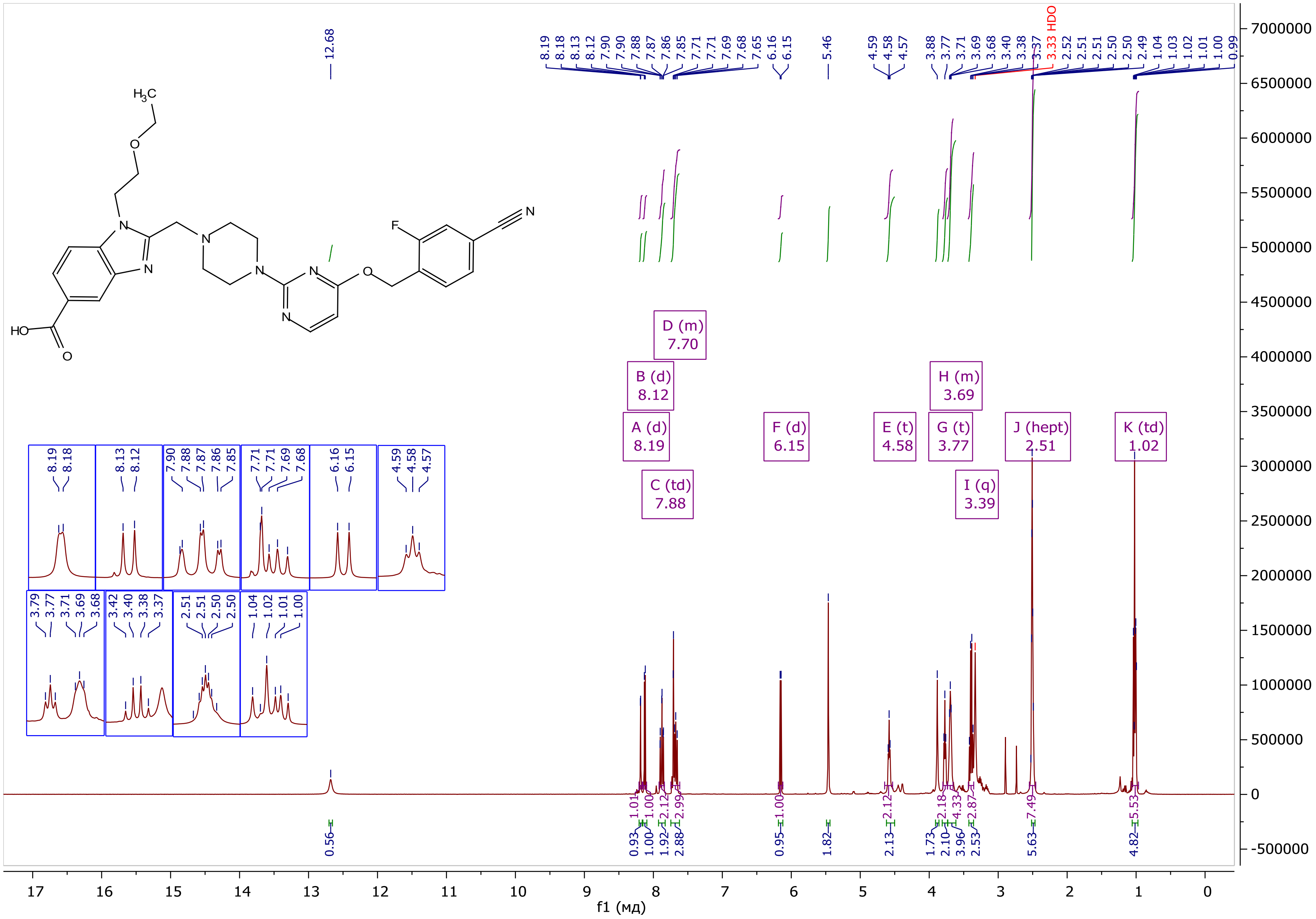

^{13}C NMR spectrum of compound **12p**

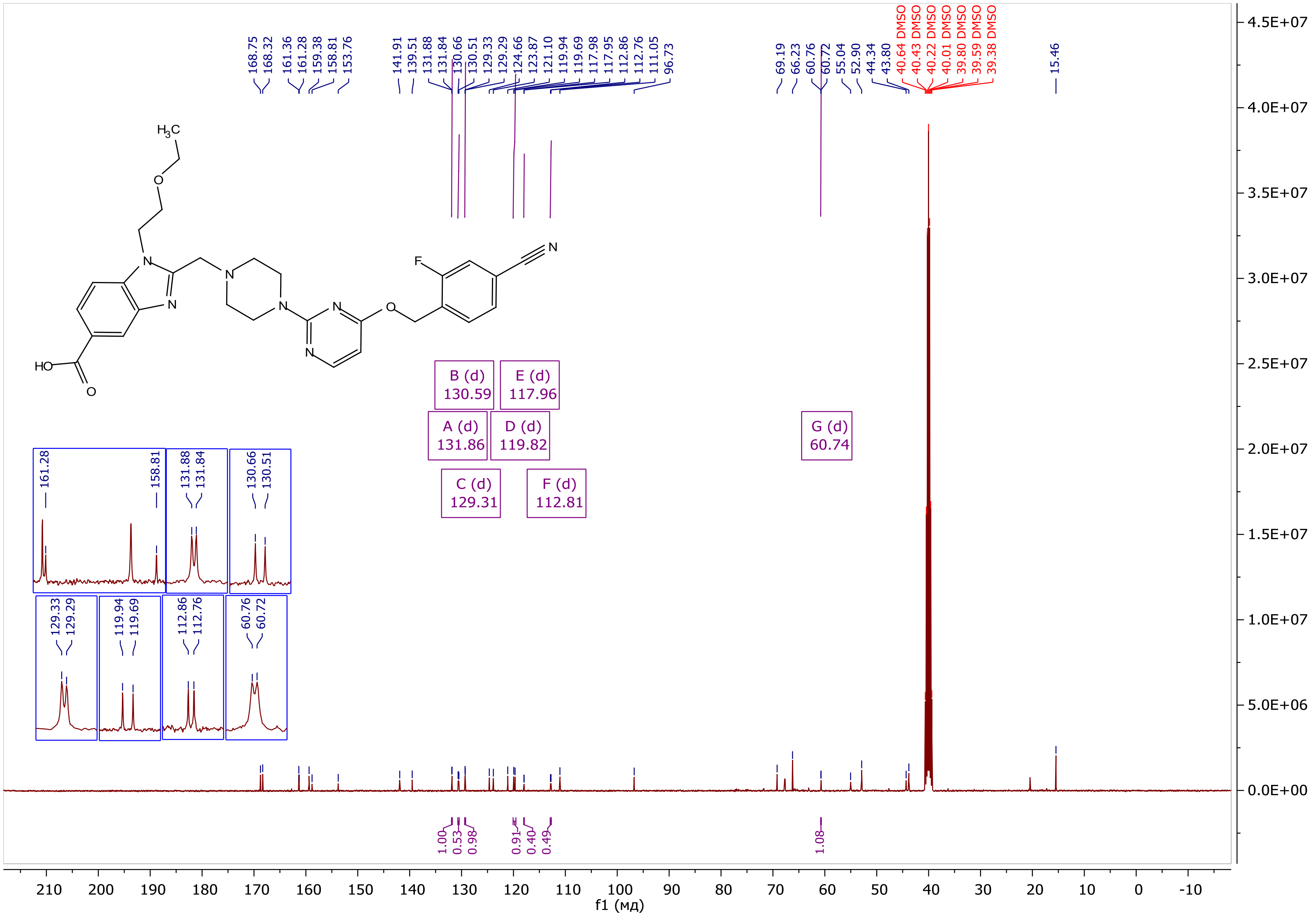

^{1}H NMR spectrum of compound **12q**

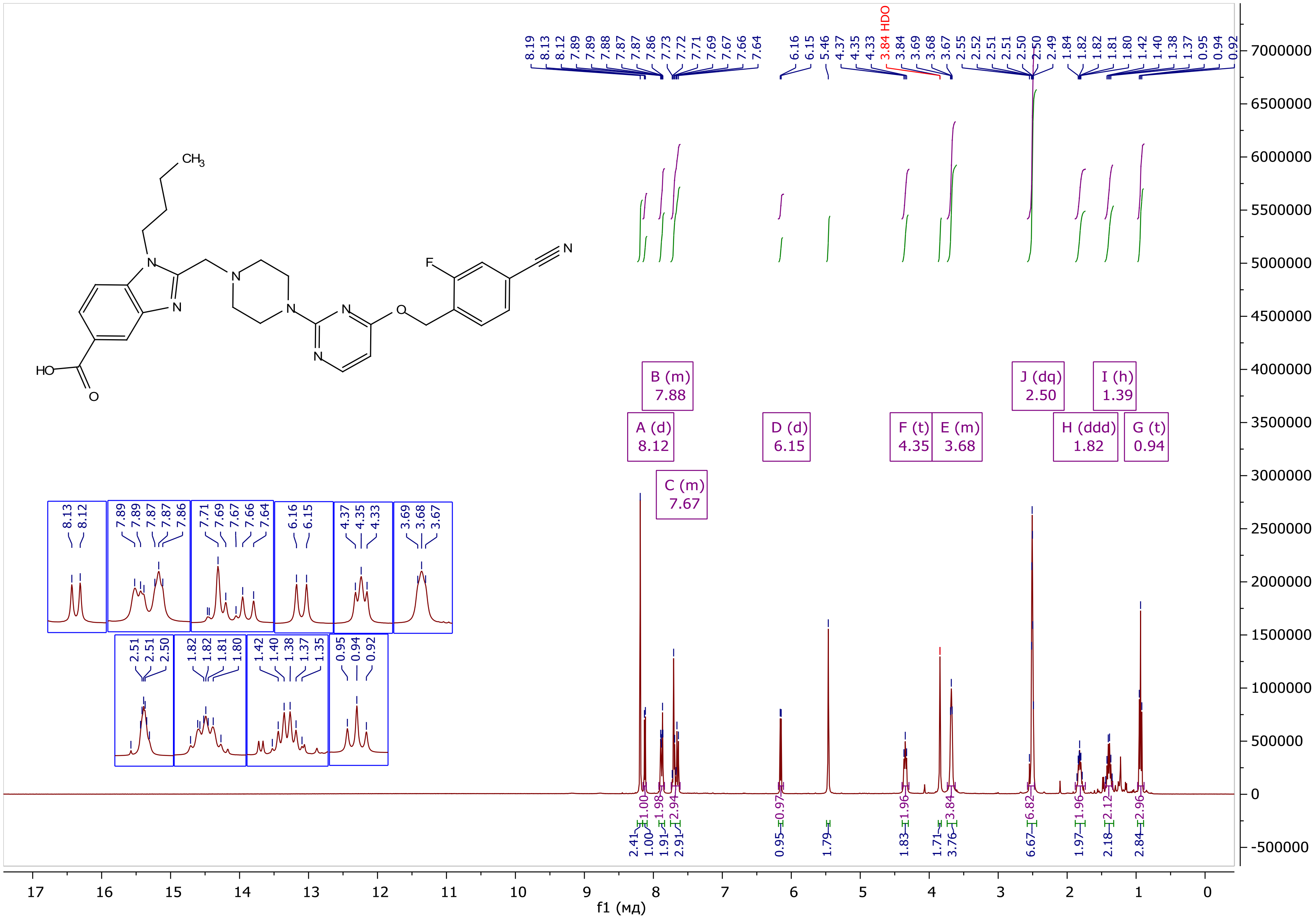

^{13}C NMR spectrum of compound **12q**

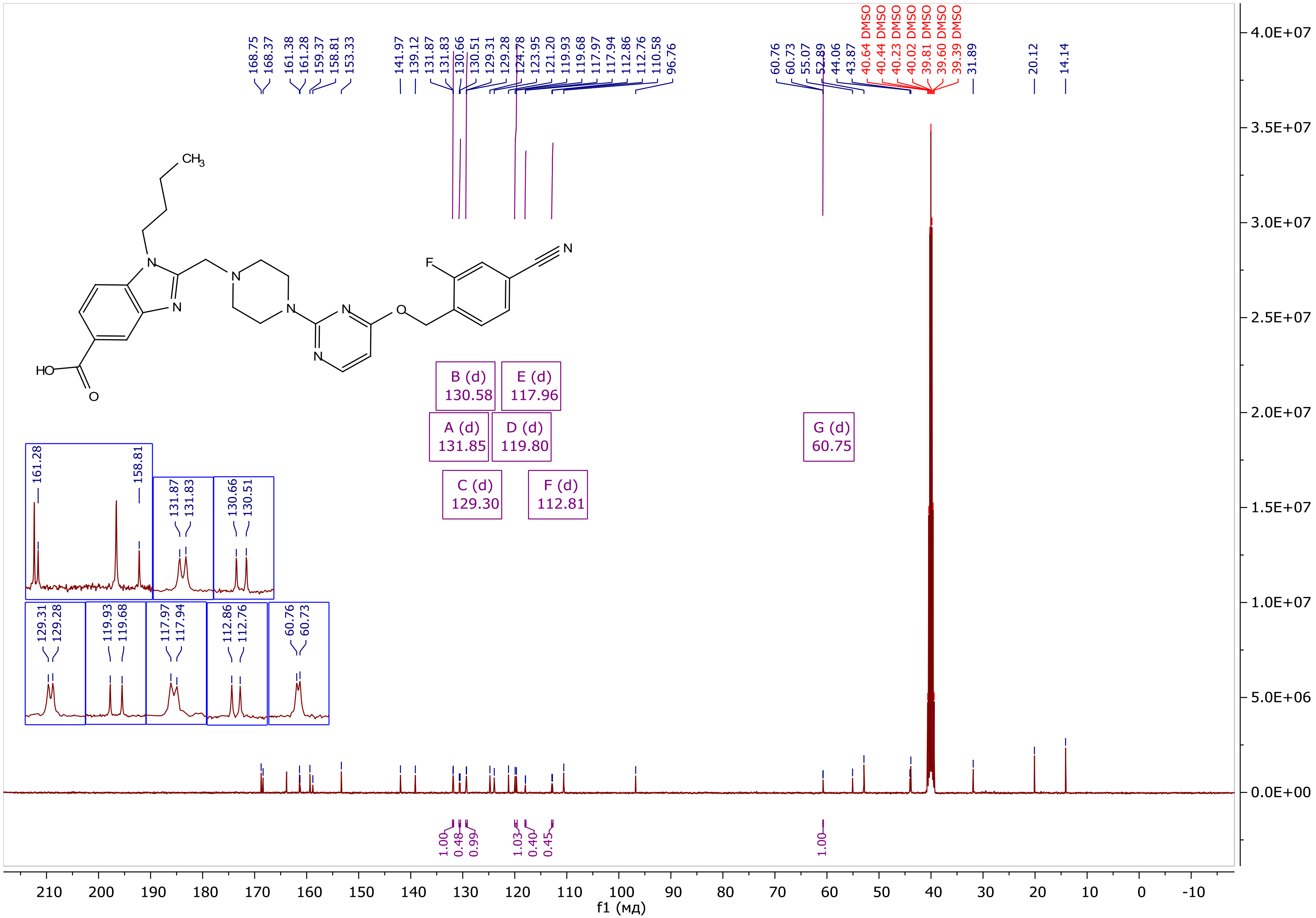

^{1}H NMR spectrum of compound **12r**

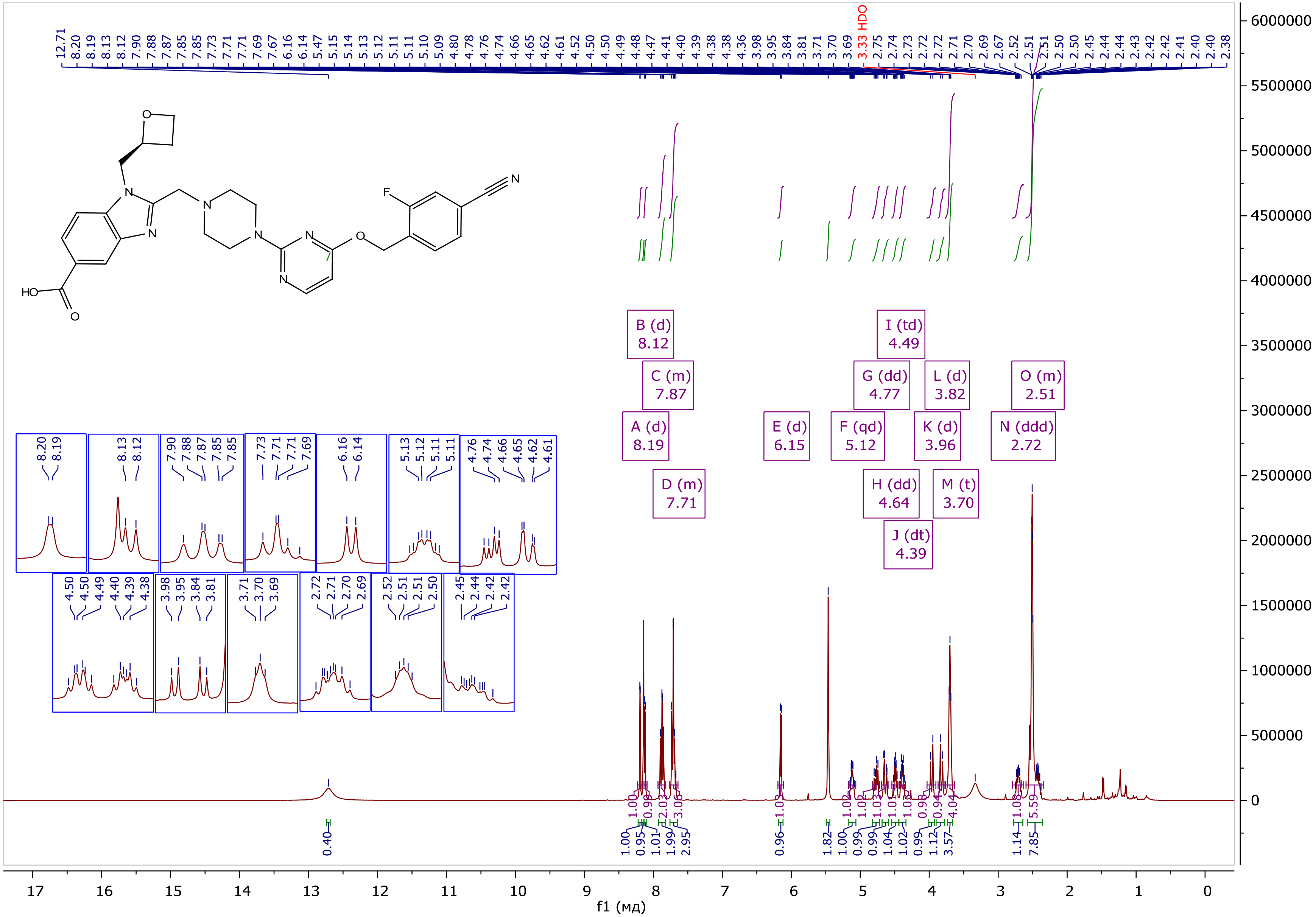

^{13}C NMR spectrum of compound **12r**

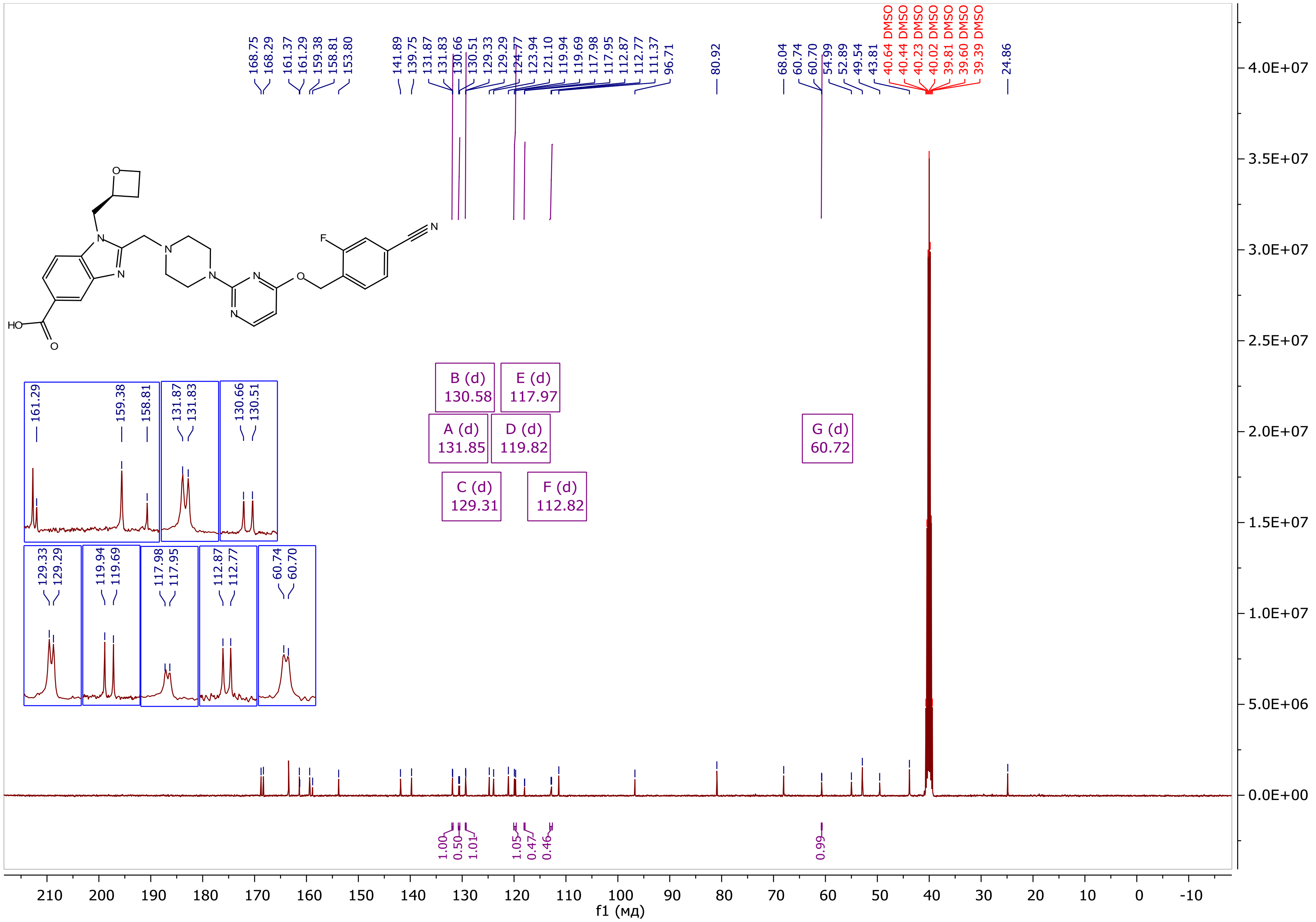

^{1}H NMR spectrum of compound **12s**

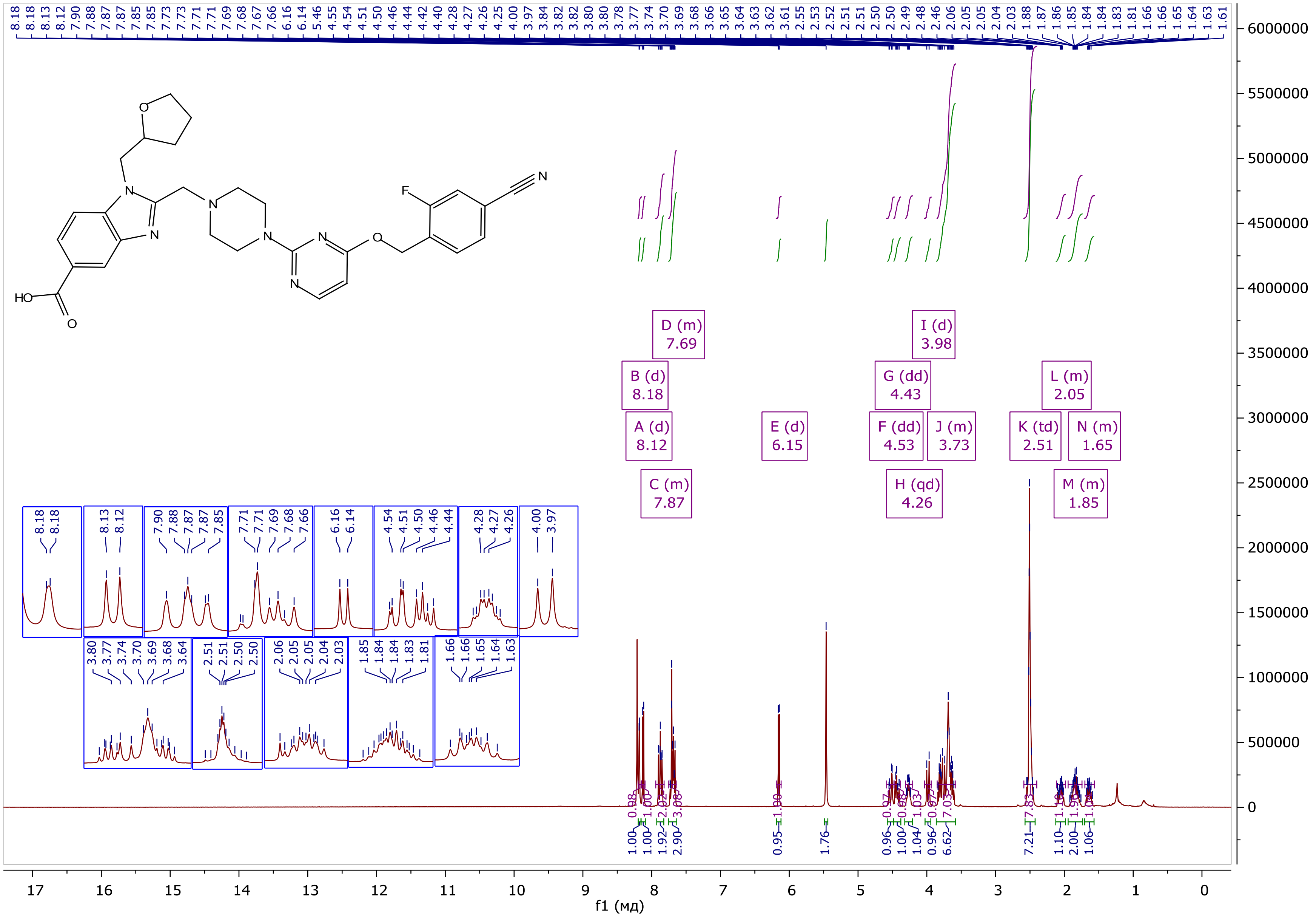

^{13}C NMR spectrum of compound **12s**

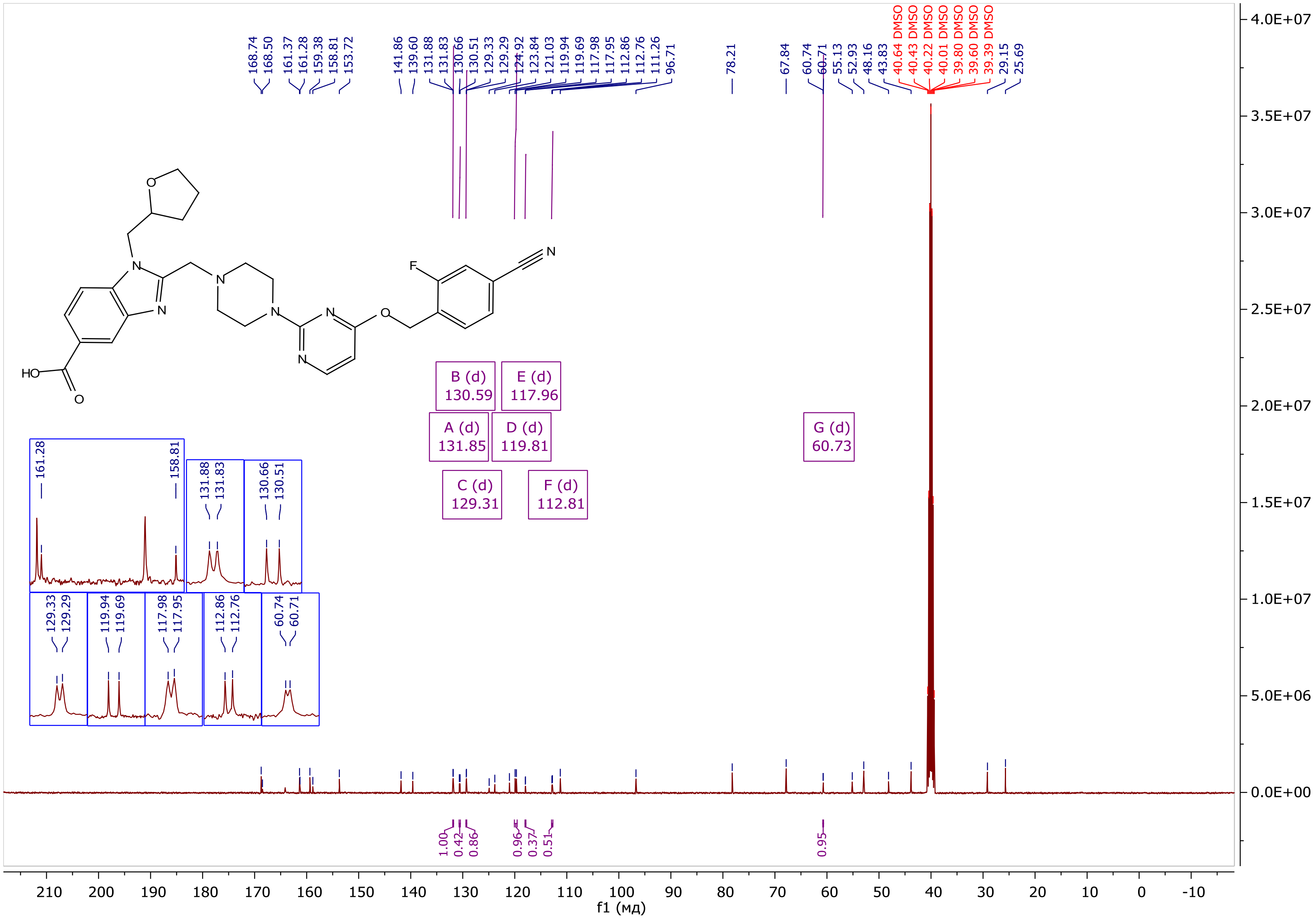

^{1}H NMR spectrum of compound **12t**

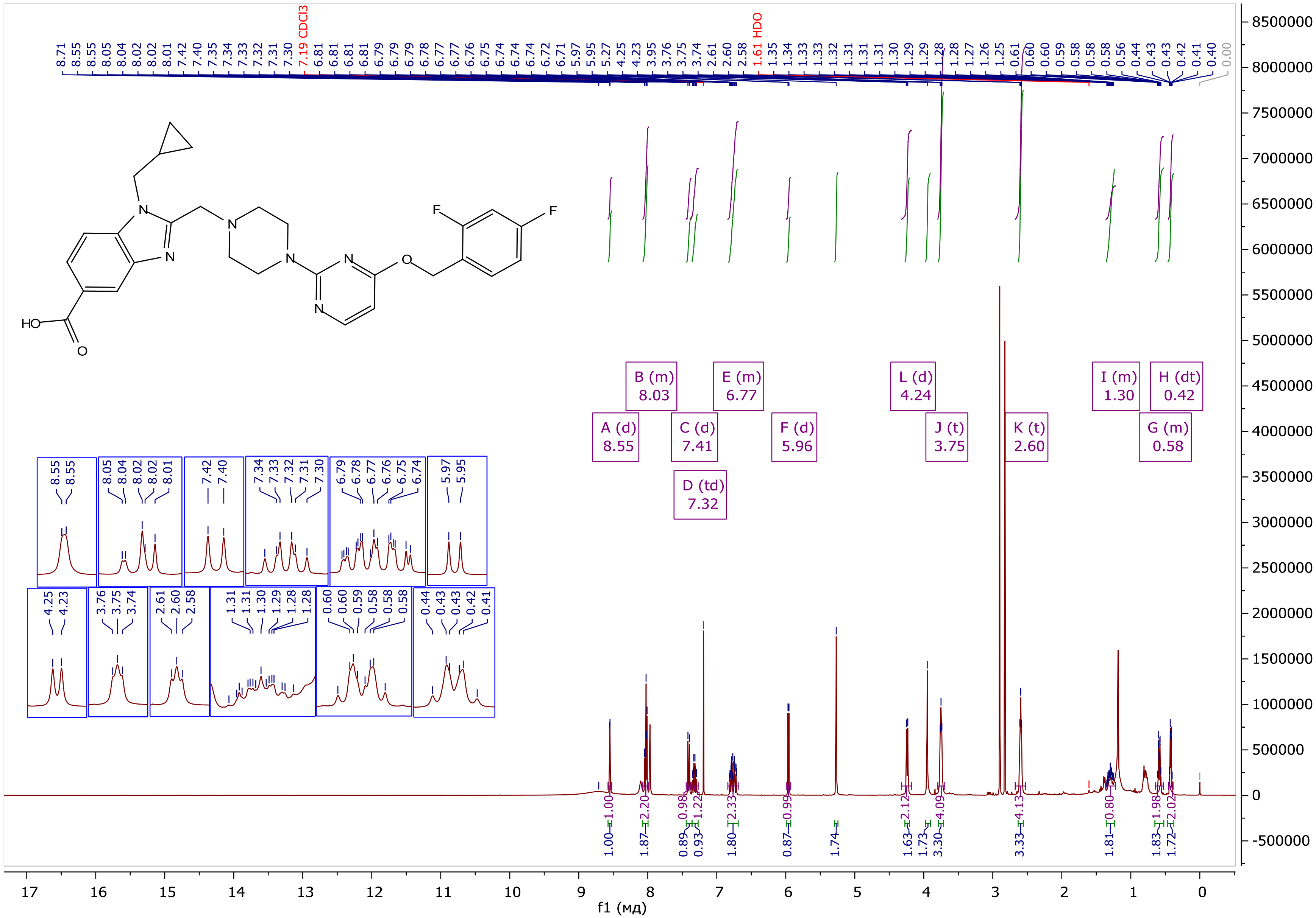

^{13}C NMR spectrum of compound **12t**

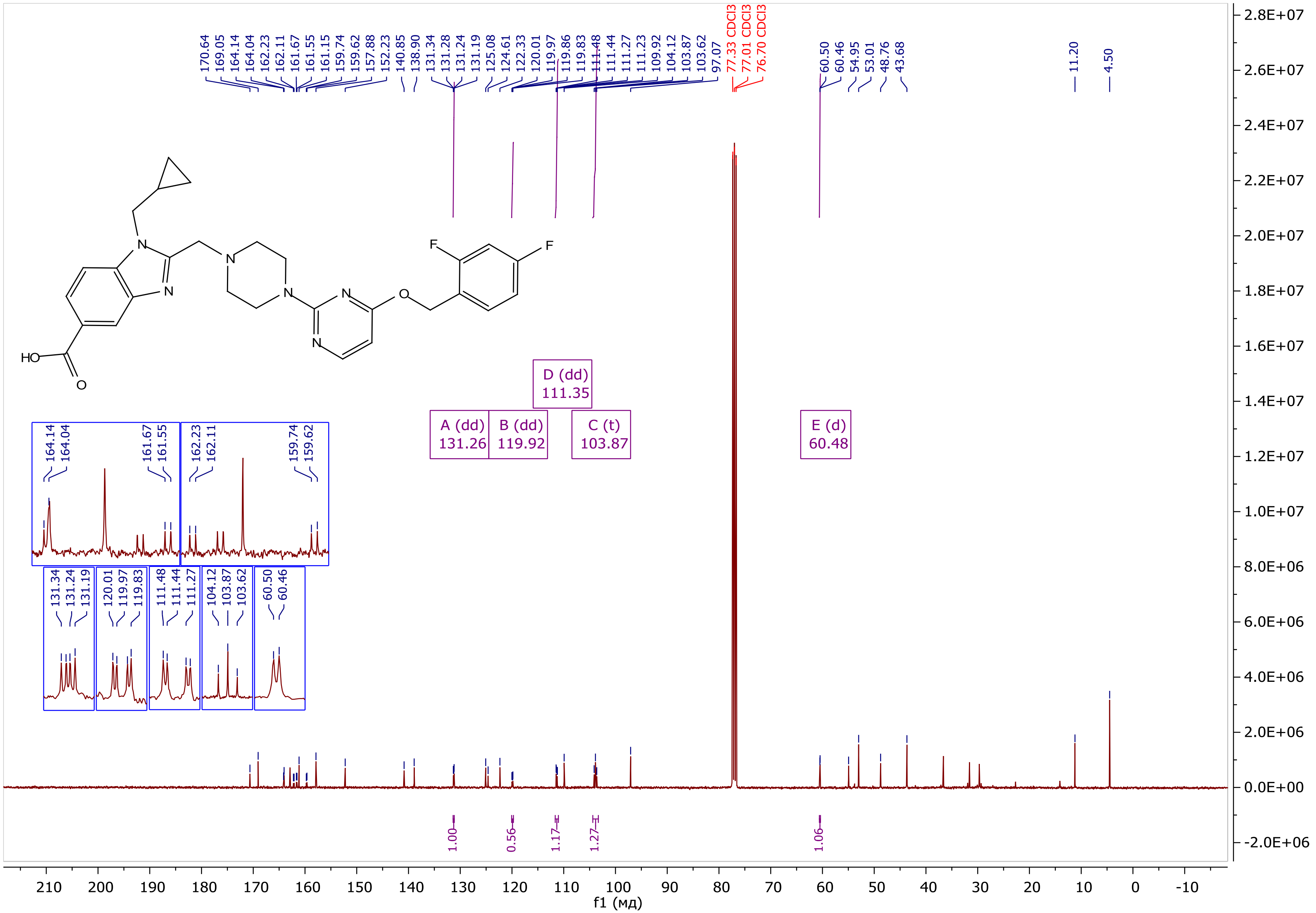

^{1}H NMR spectrum of compound **12u**

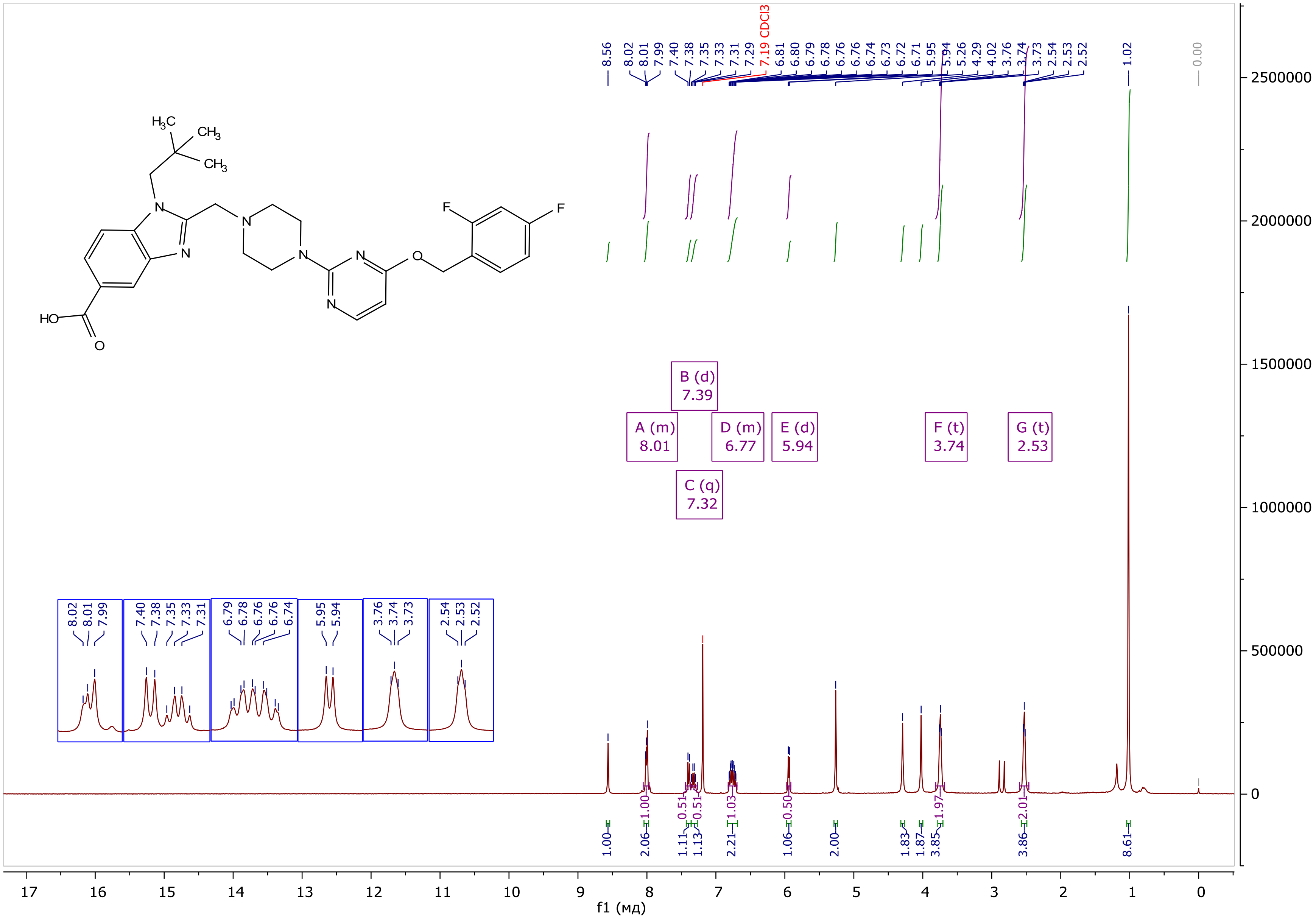

^{13}C NMR spectrum of compound **12u**

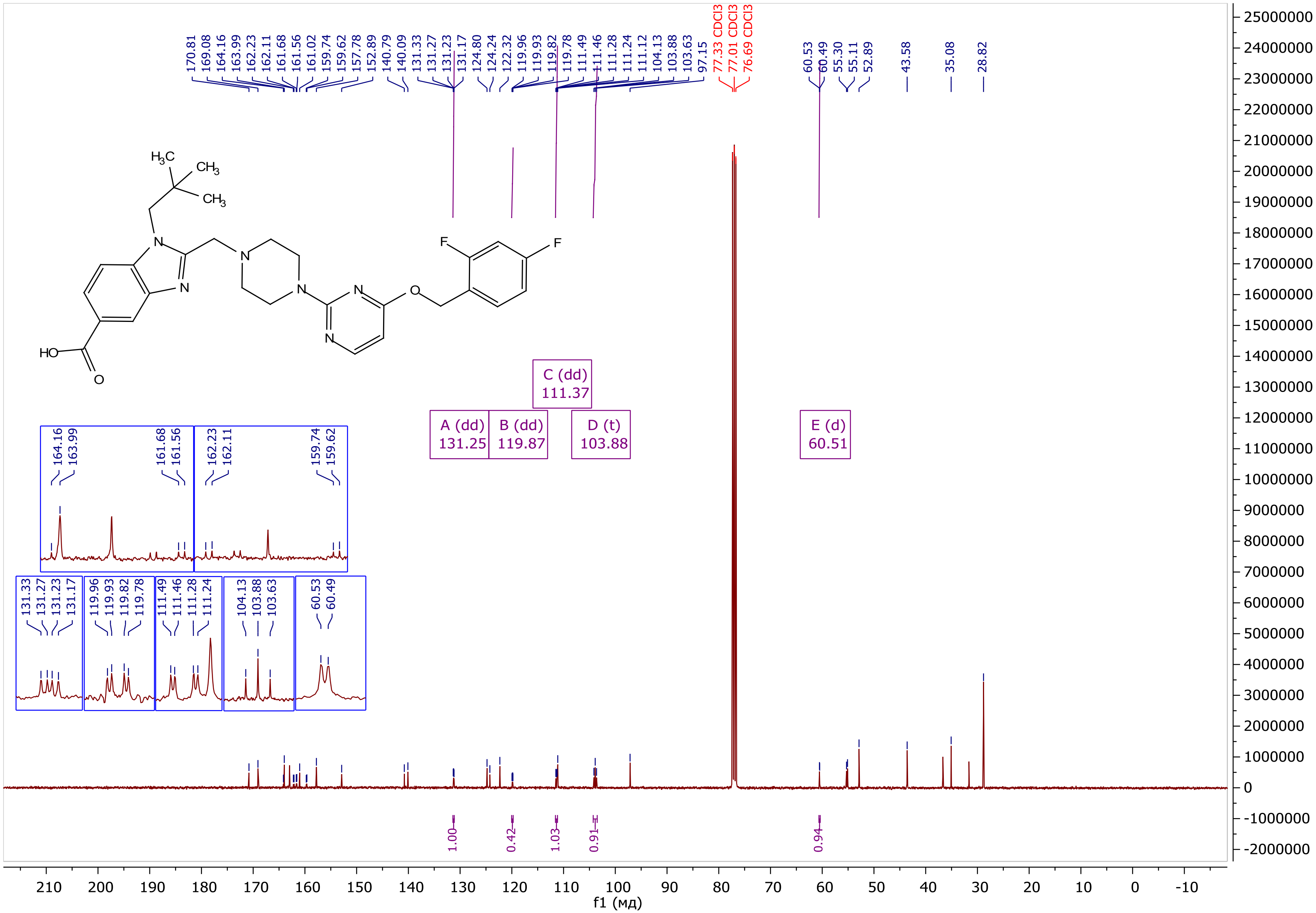

^{1}H NMR spectrum of compound **12v**

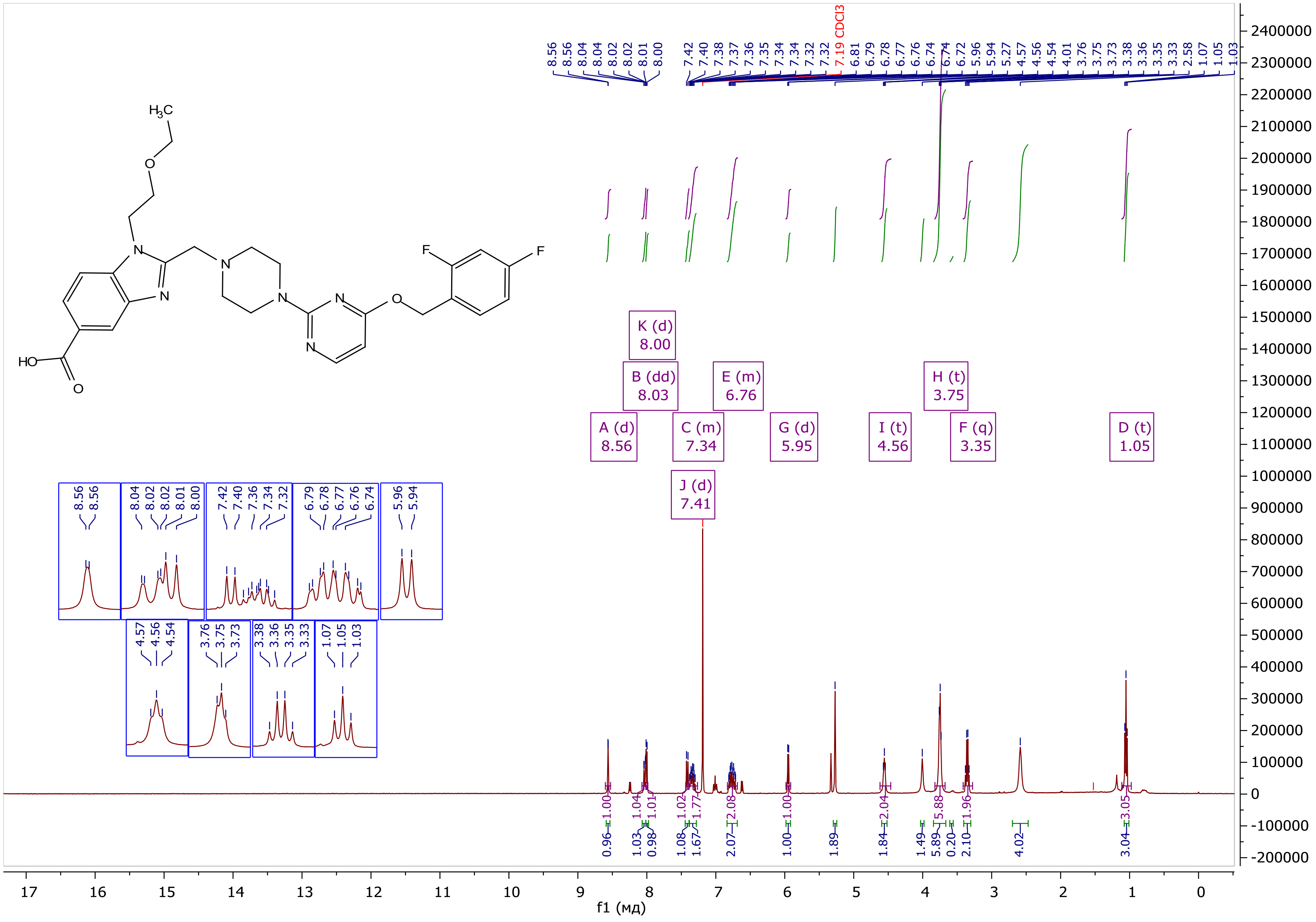

^{13}C NMR spectrum of compound **12v**

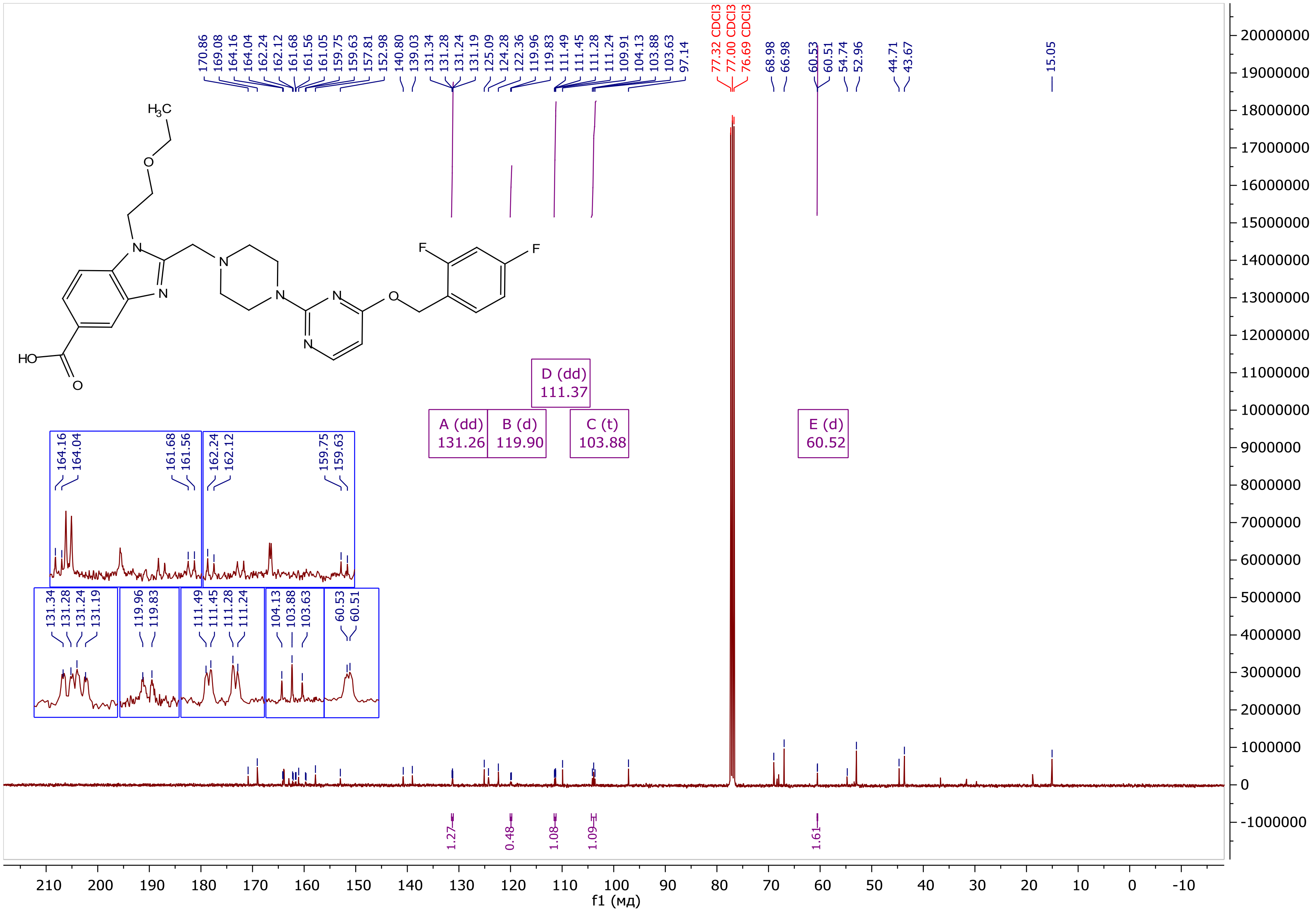

^{1}H NMR spectrum of compound **12w**

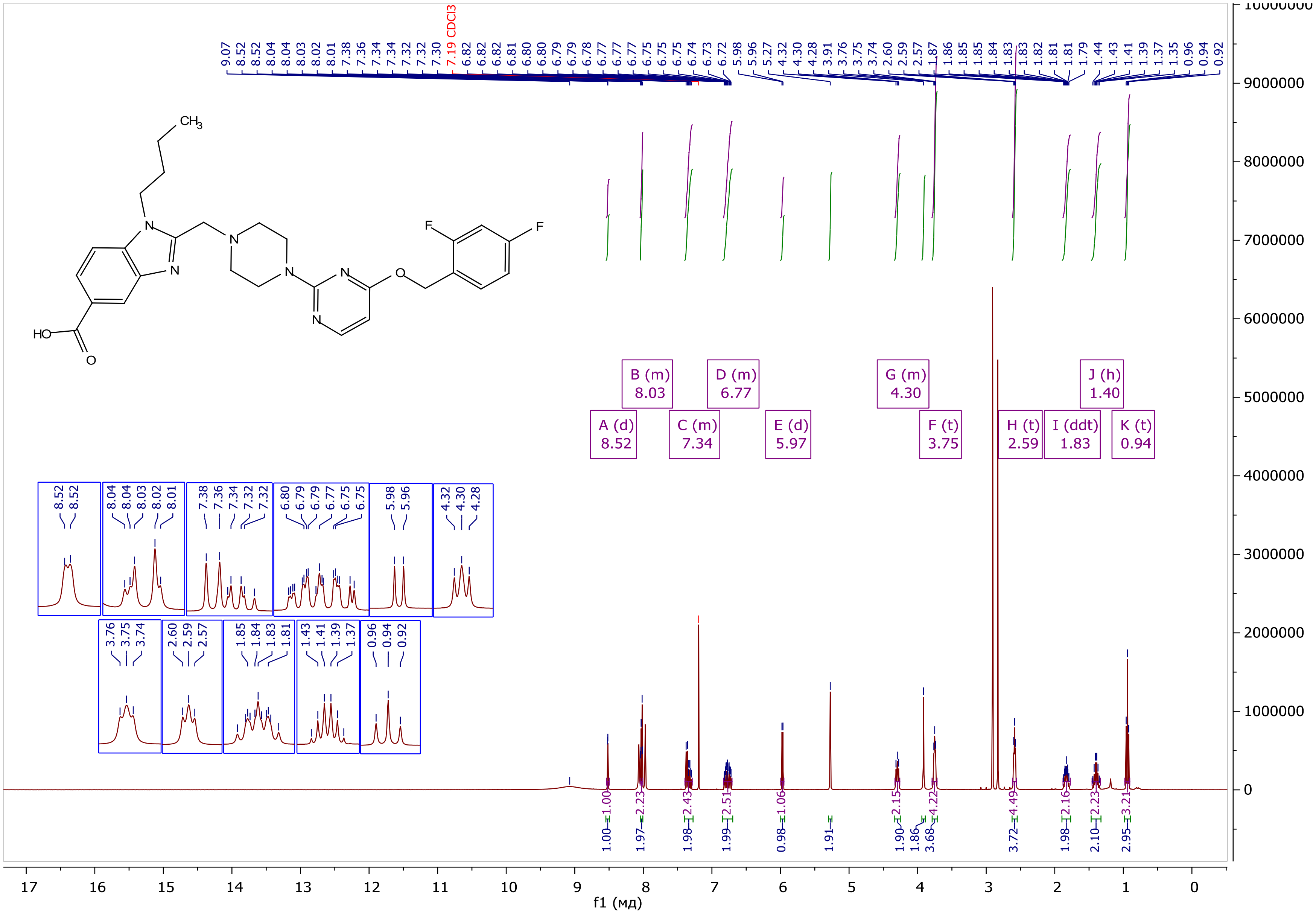

^{13}C NMR spectrum of compound **12w**

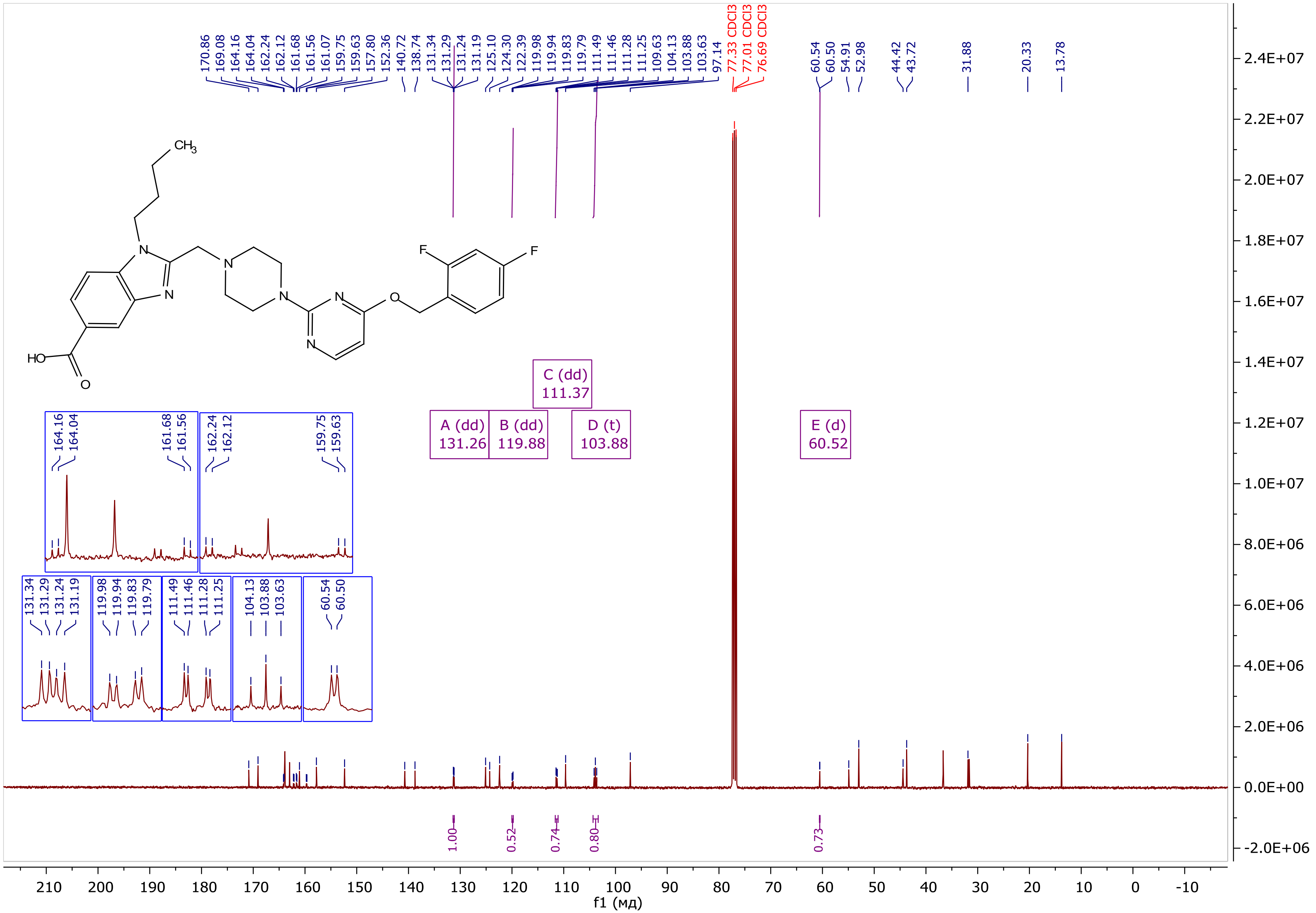

^{1}H NMR spectrum of compound **12x**

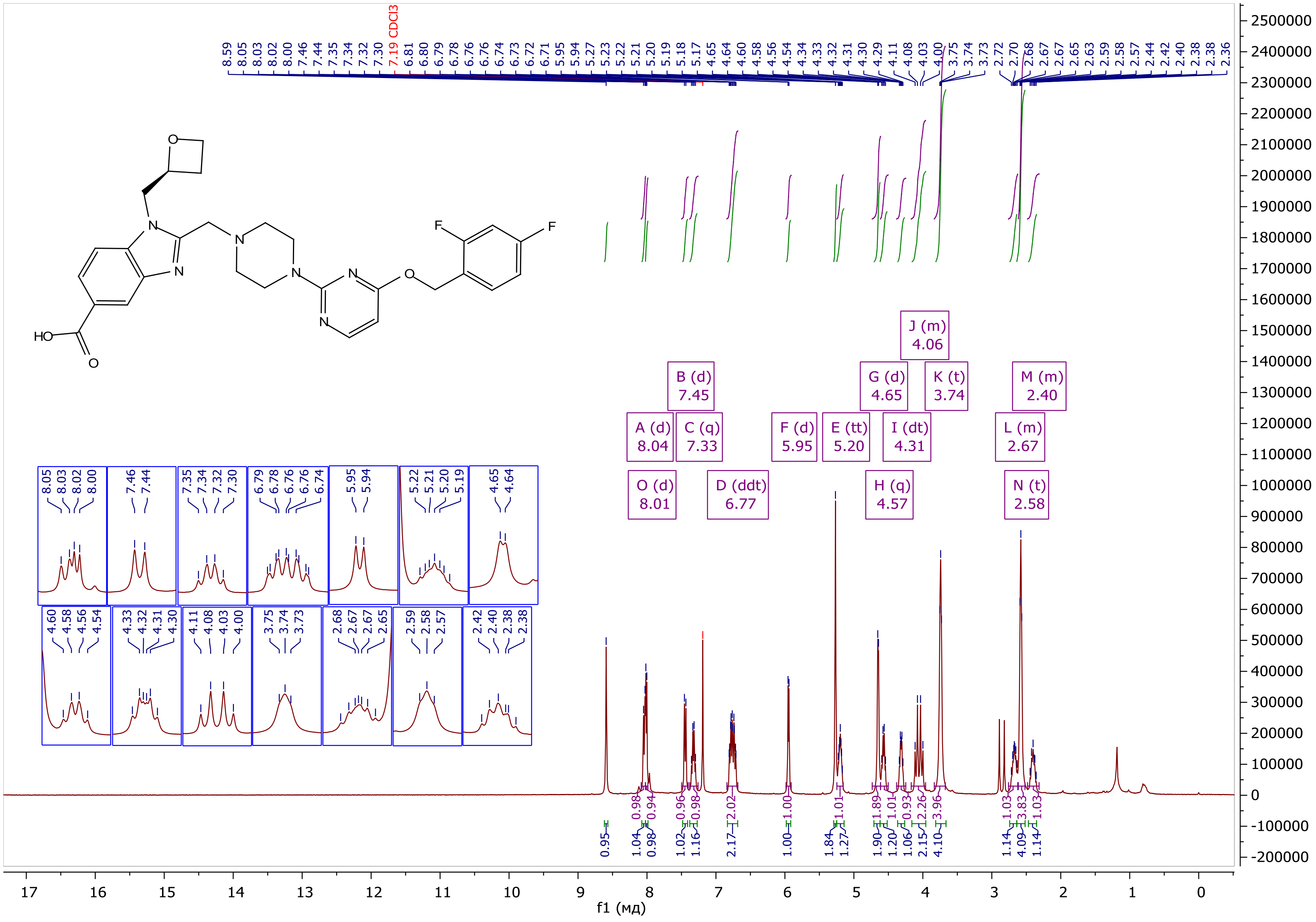

^{13}C NMR spectrum of compound **12x**

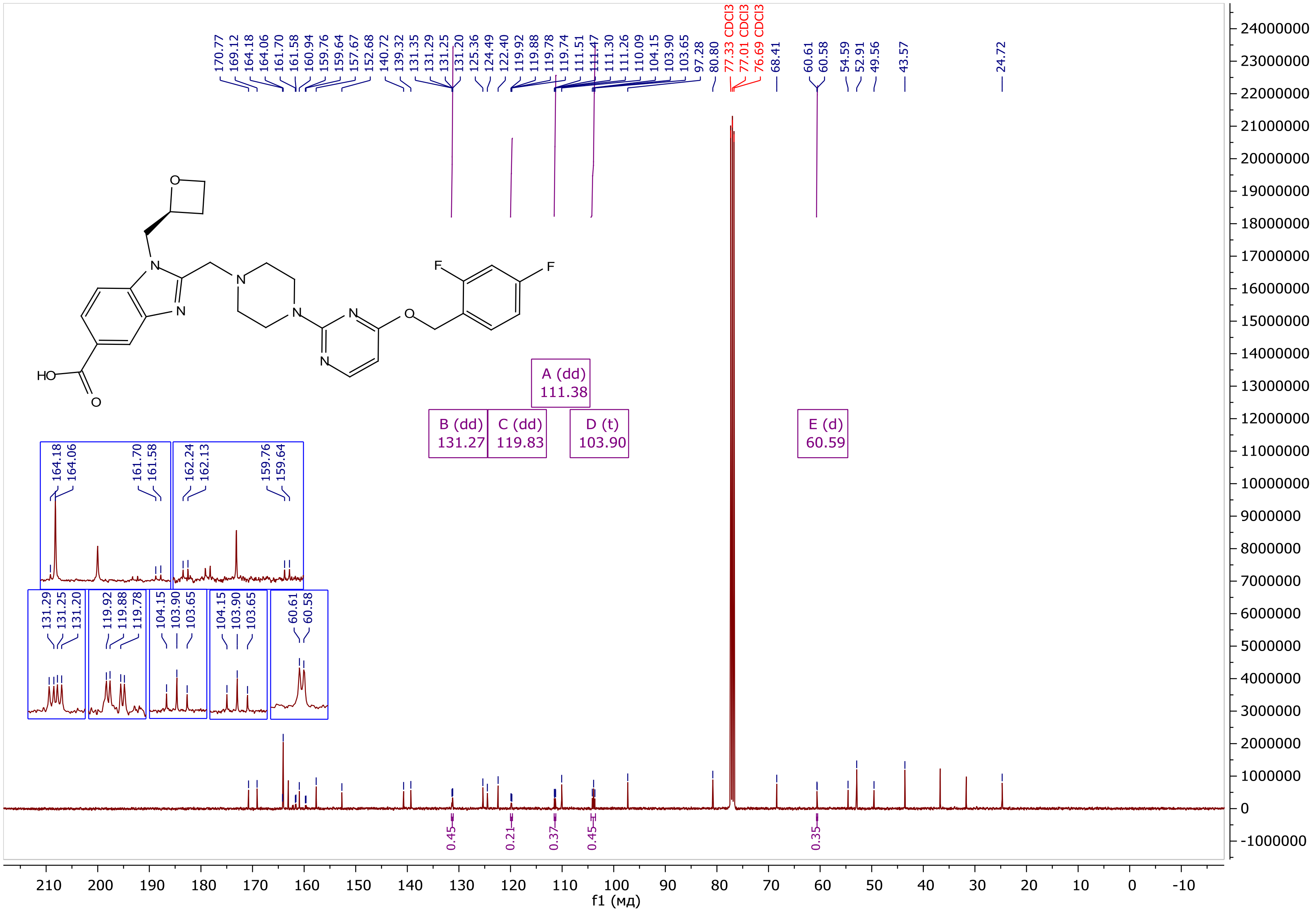

^{1}H NMR spectrum of compound **12y**

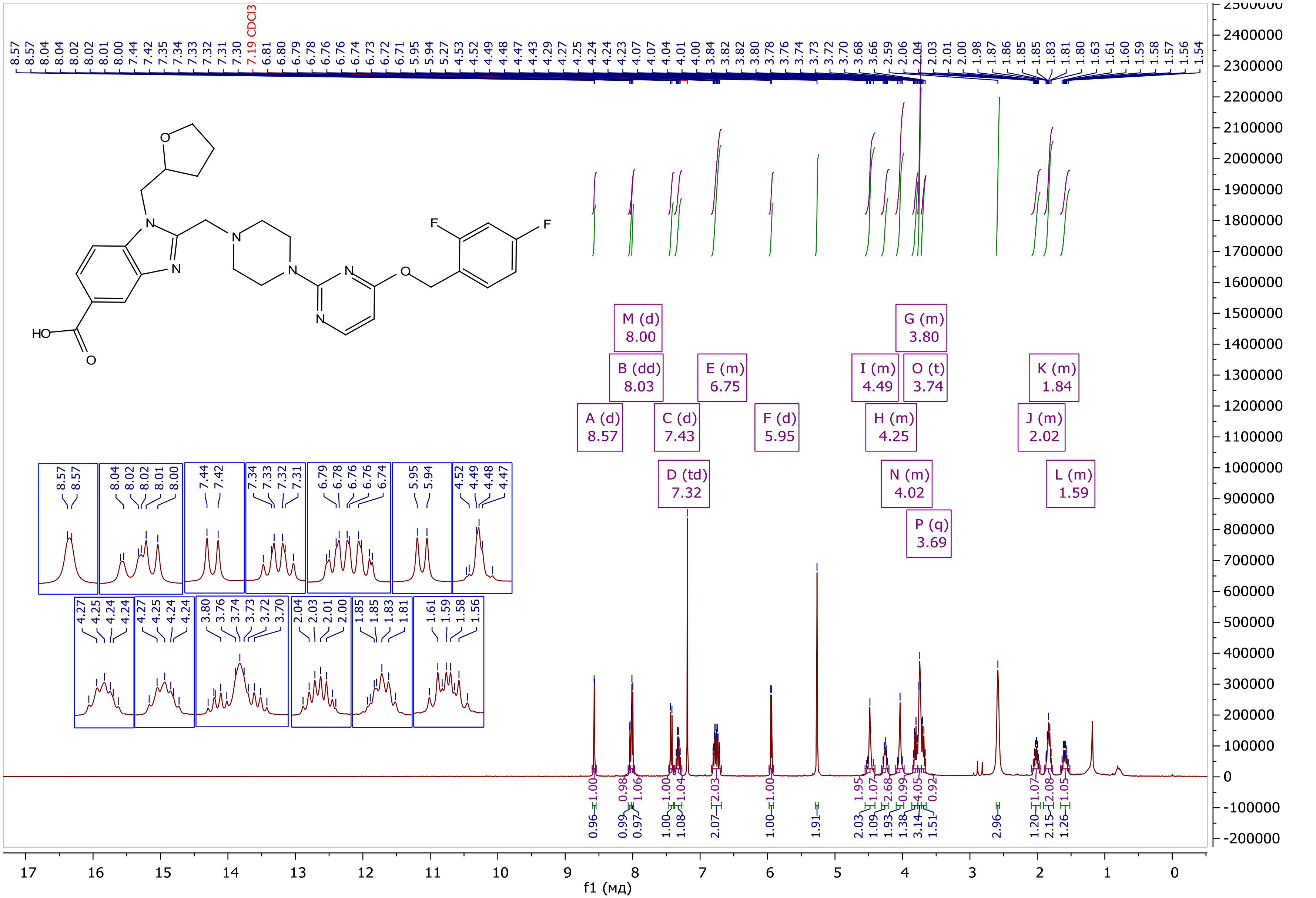

^{1}H NMR spectrum of compound **12z**

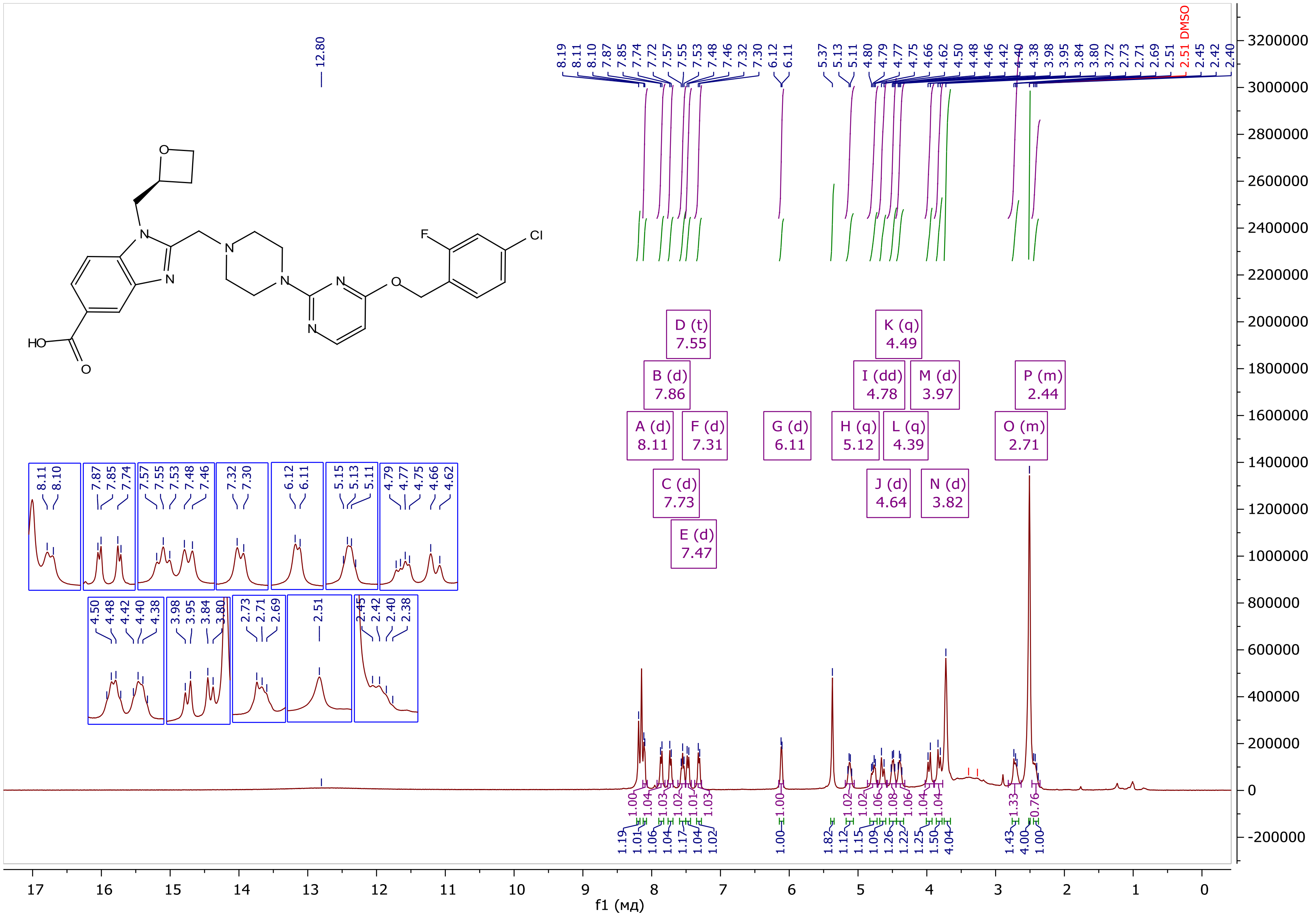

^{13}C NMR spectrum of compound **12z**

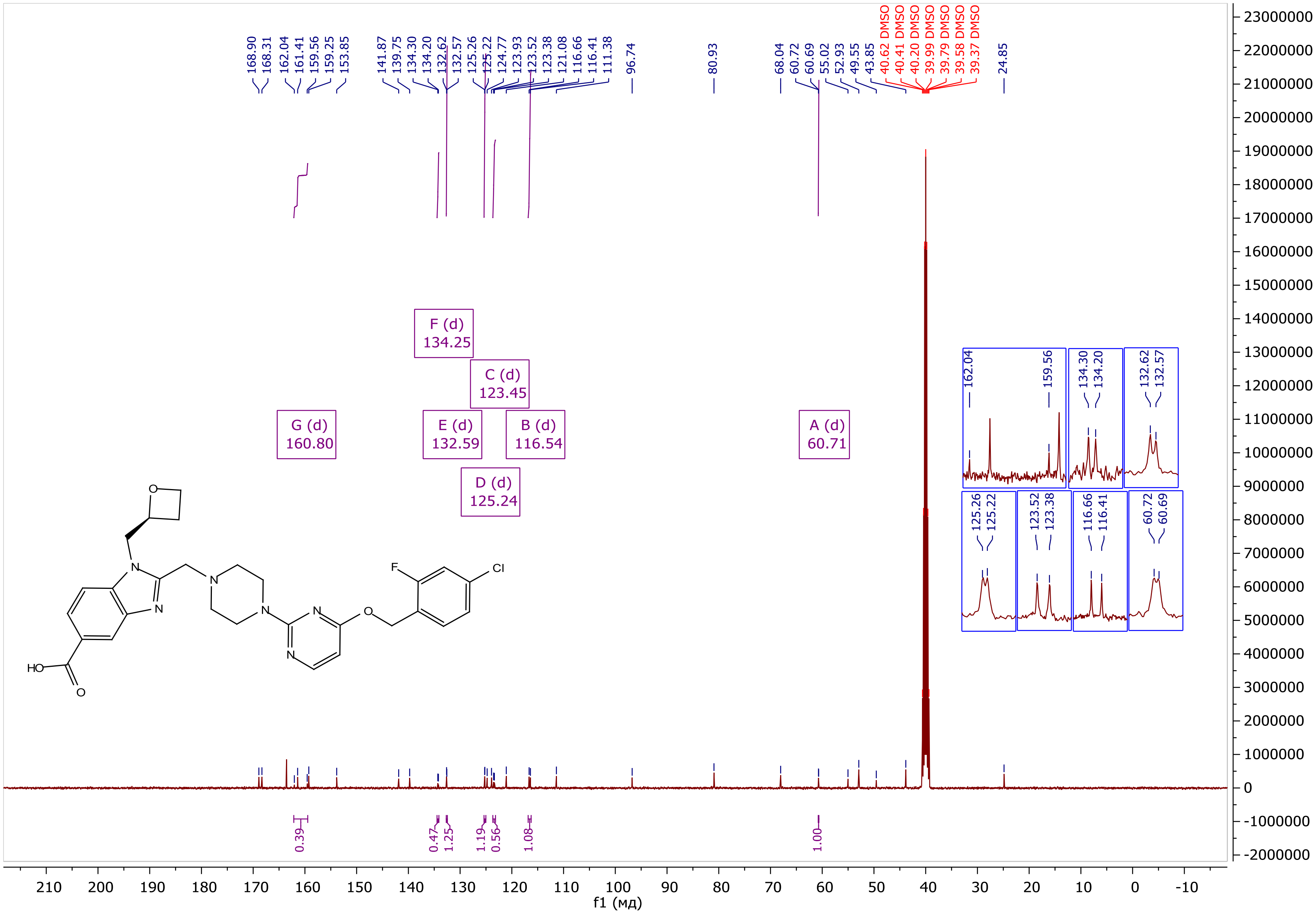

^{1}H NMR spectrum of compound **12aa**

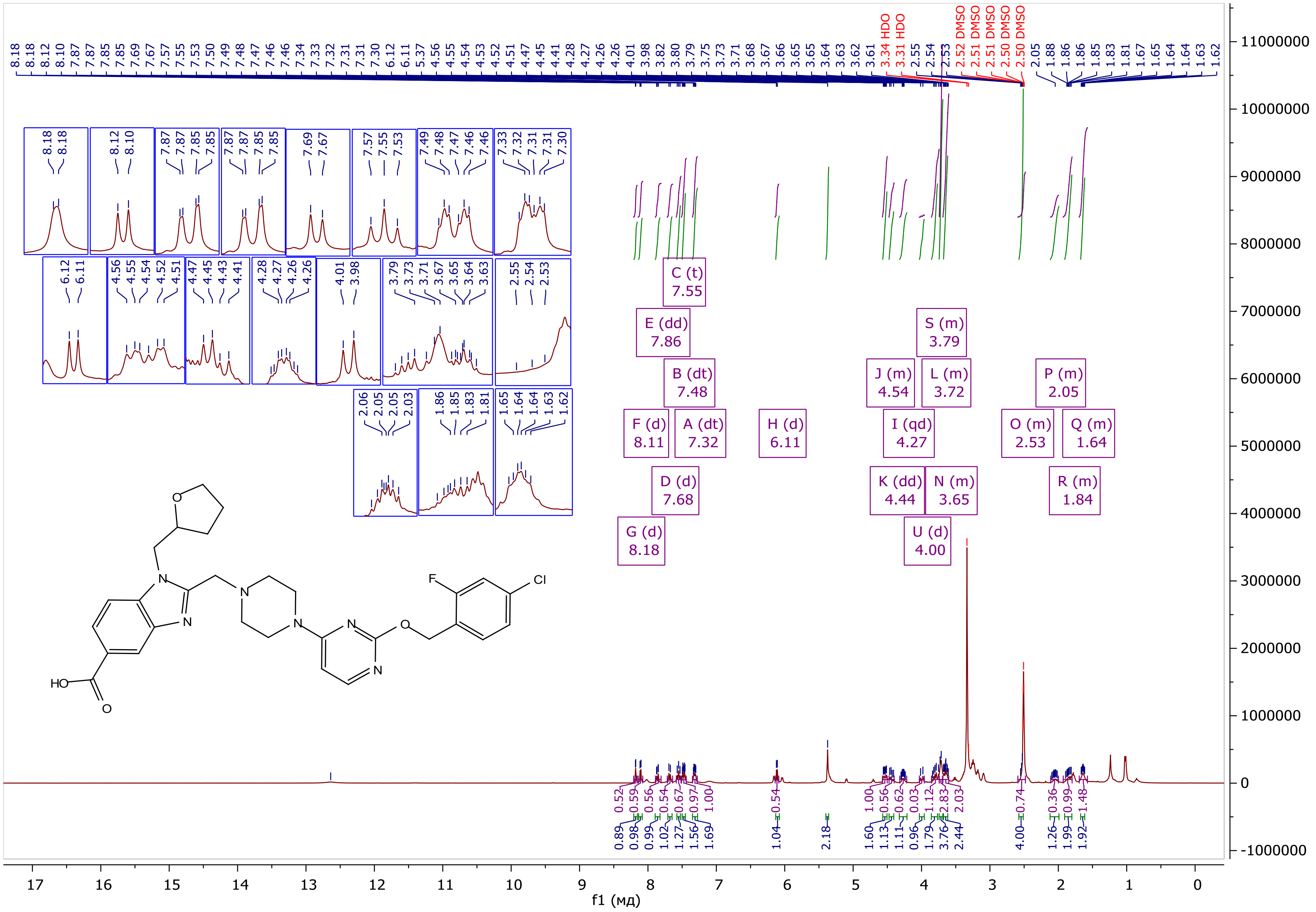

^{13}C NMR spectrum of compound **12aa**

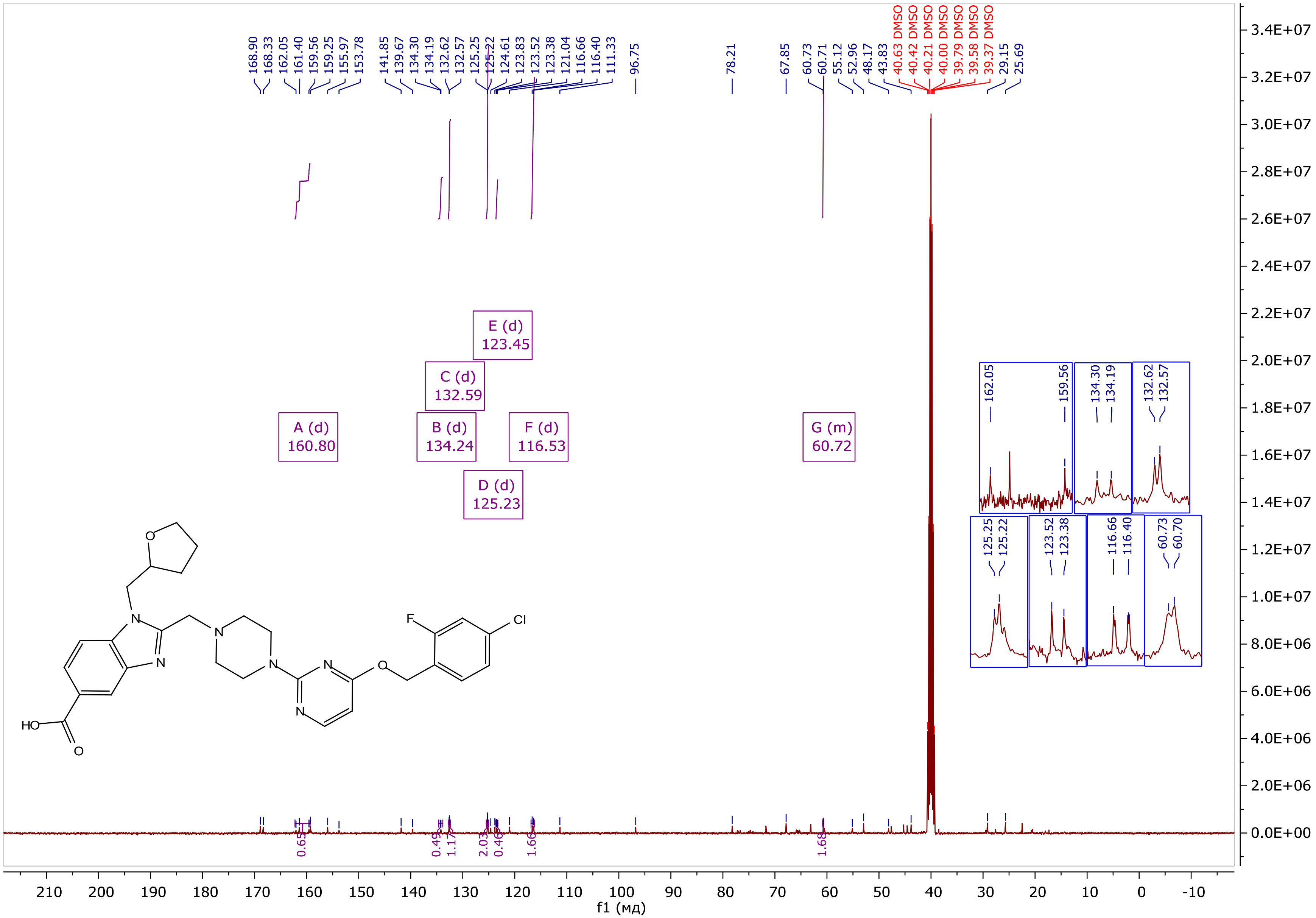

^{1}H NMR spectrum of compound **12r'**

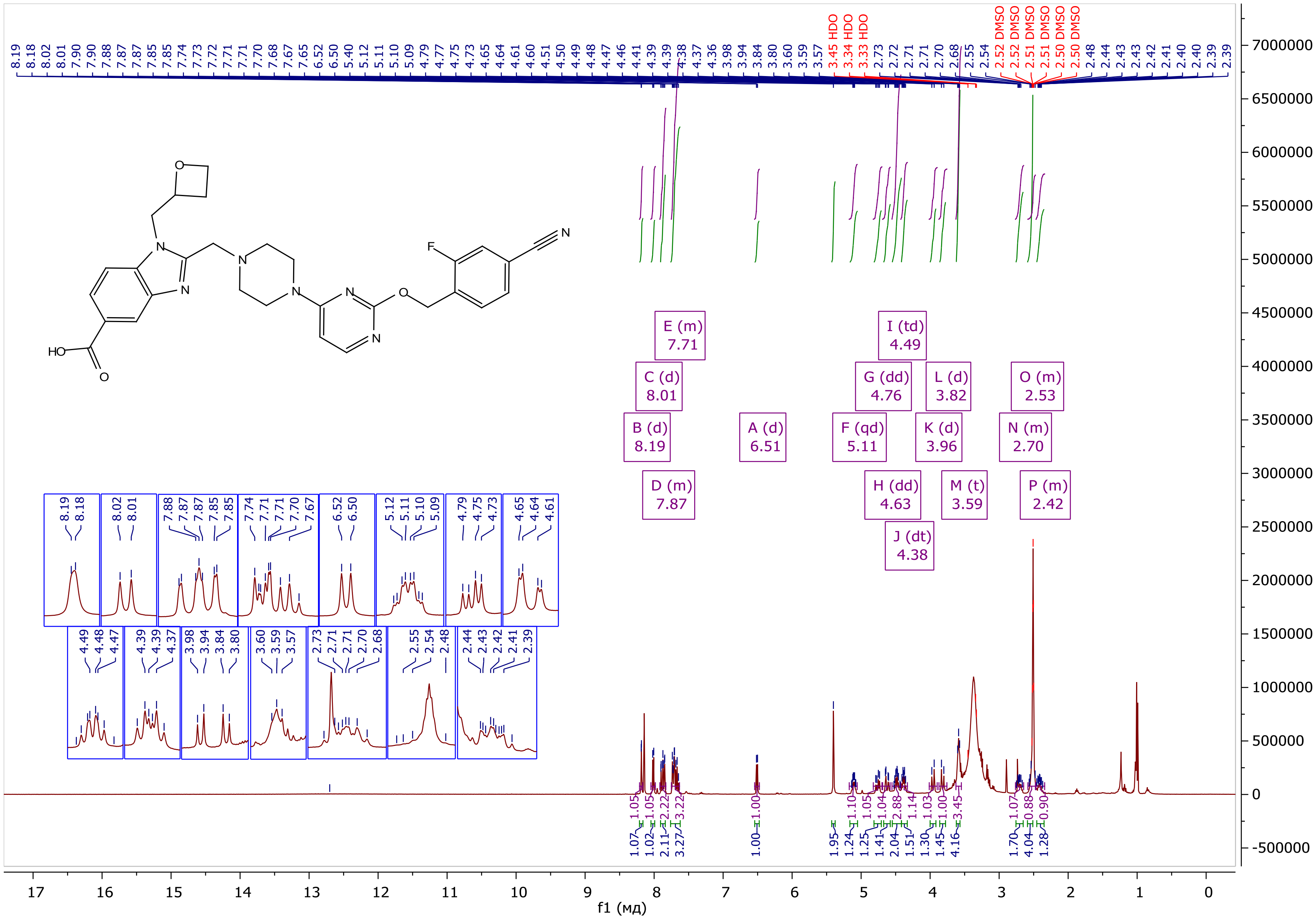

^{13}C NMR spectrum of compound **12r'**

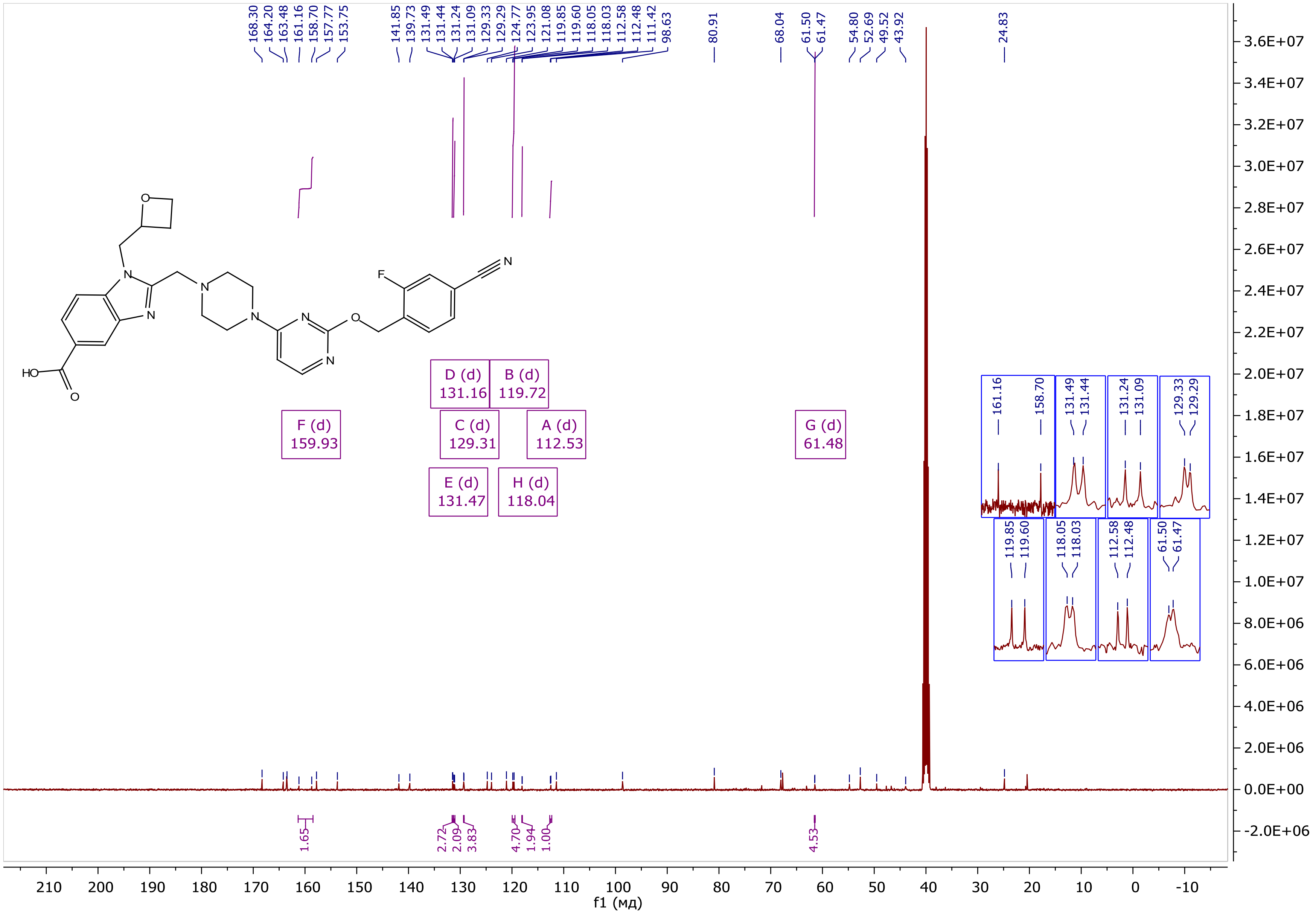

^{1}H NMR spectrum of compound **12s'**

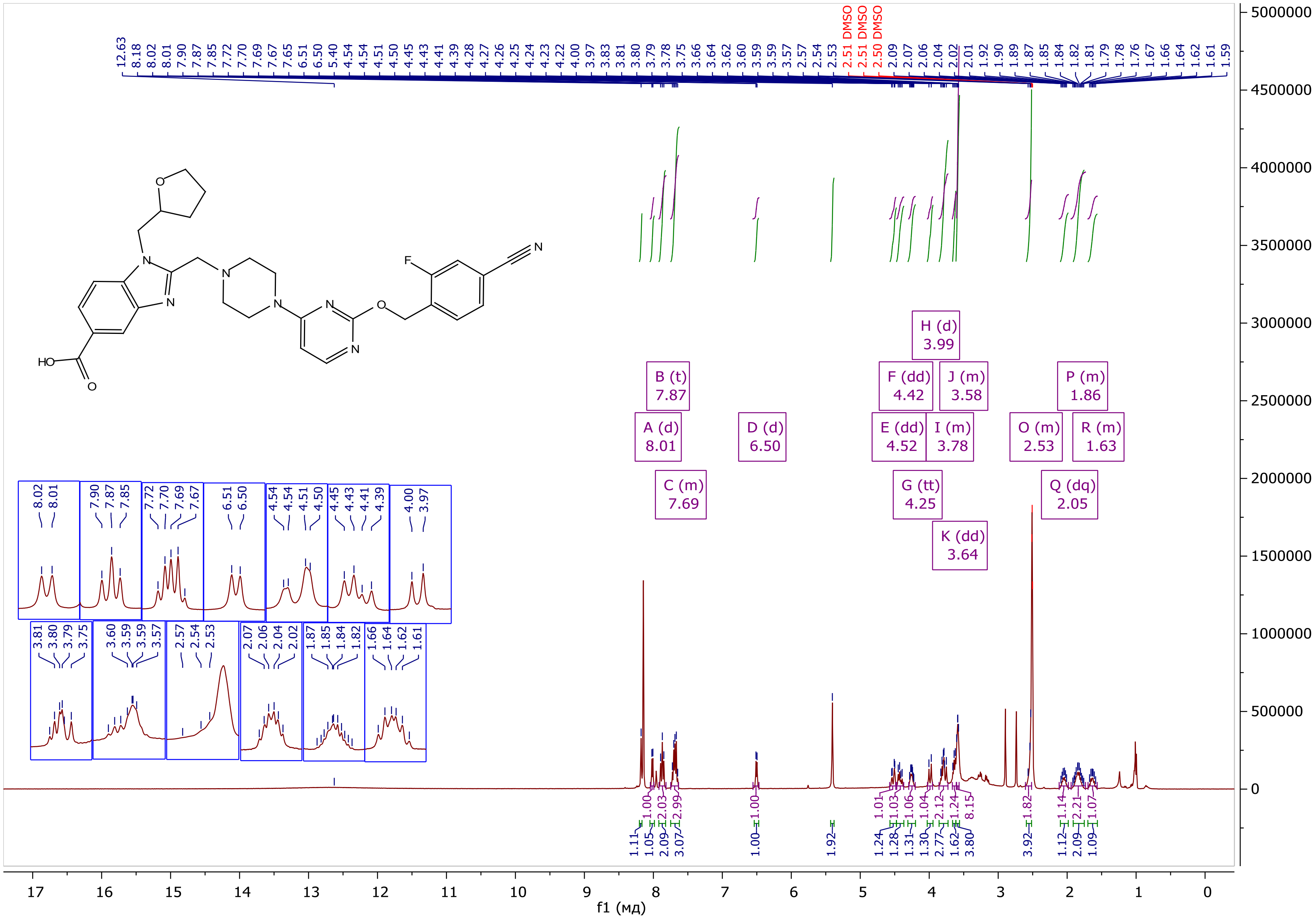

^{13}C NMR spectrum of compound **12s'**

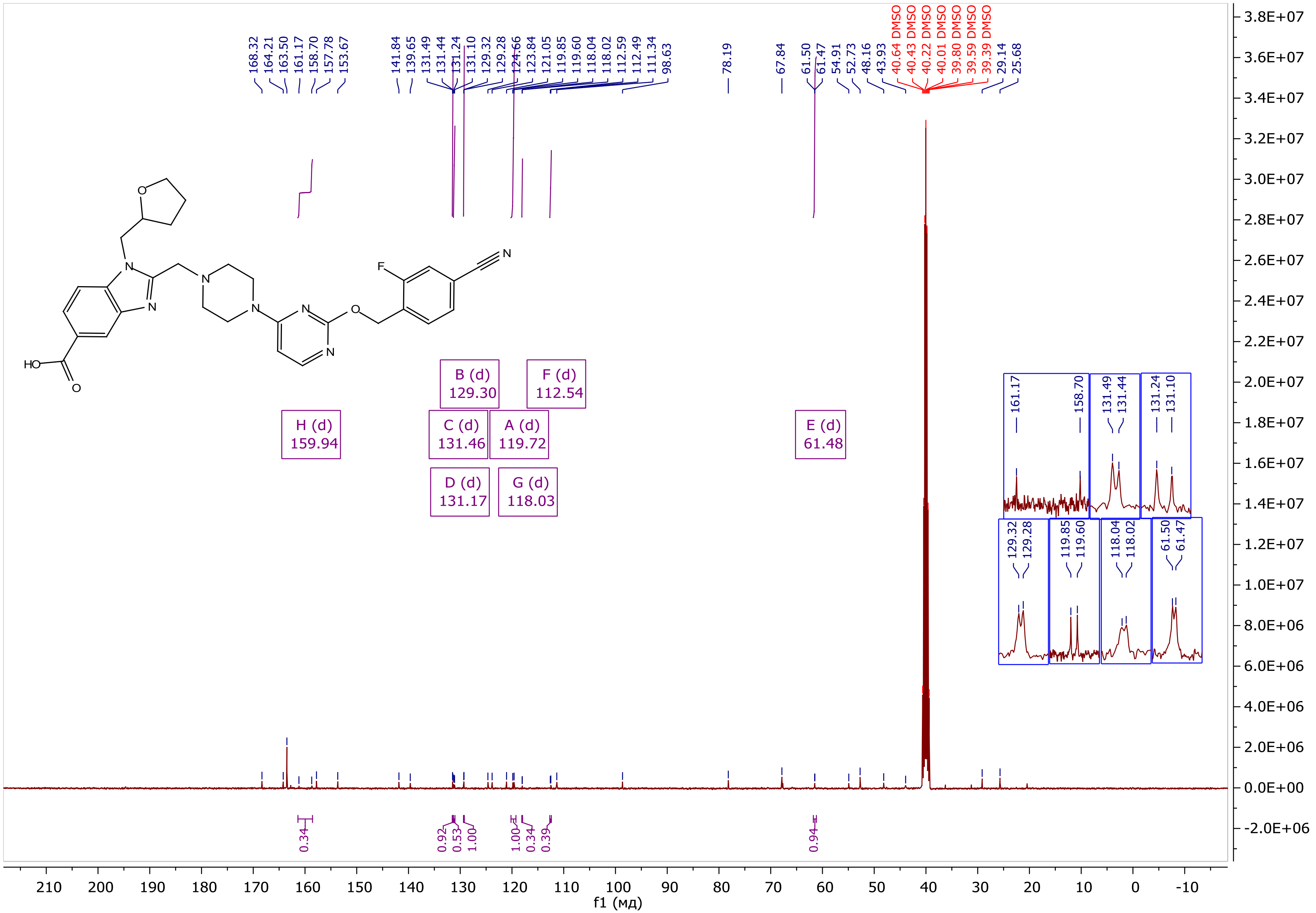

^{1}H NMR spectrum of compound **12z'**

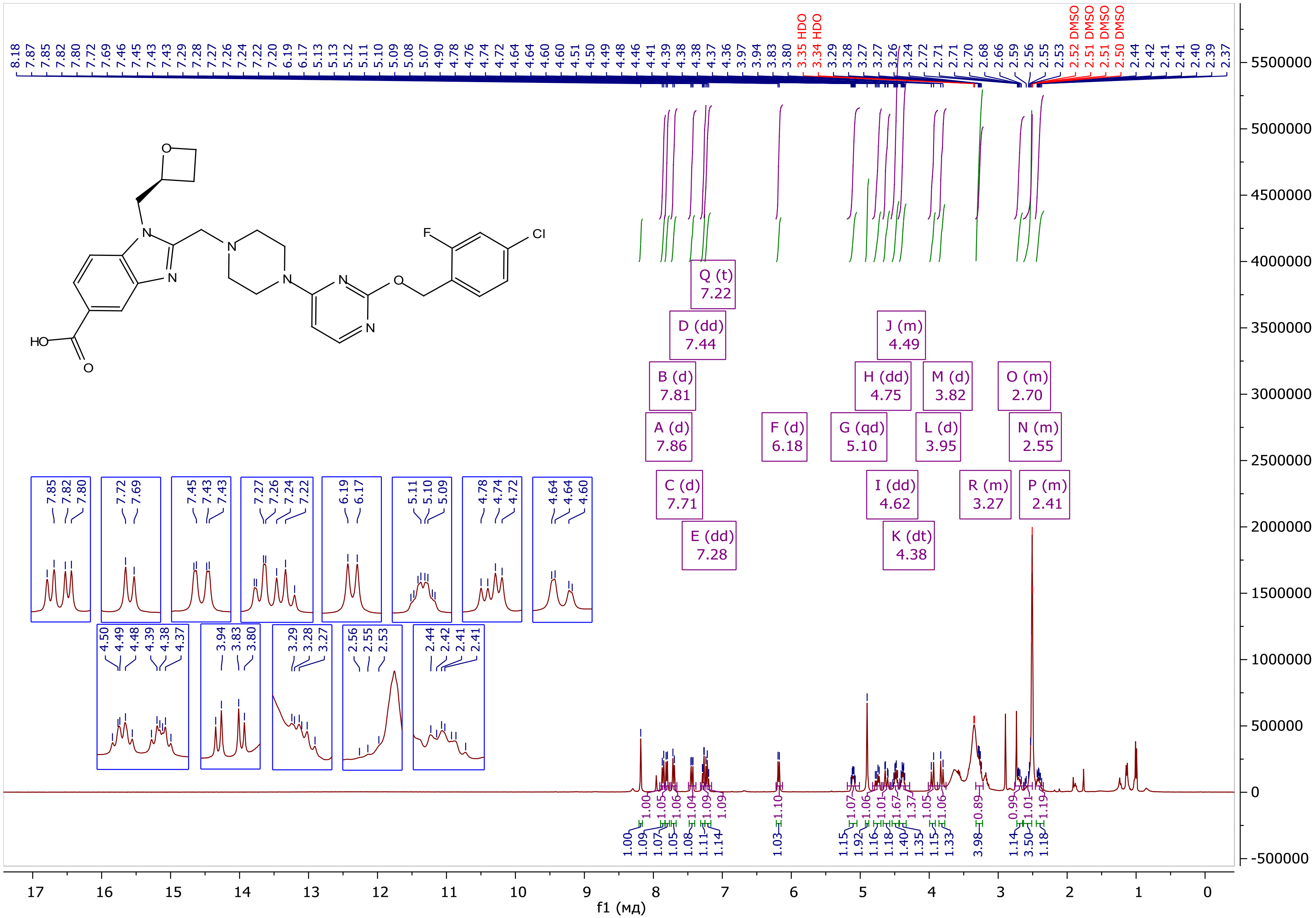

^{13}C NMR spectrum of compound **12z'**

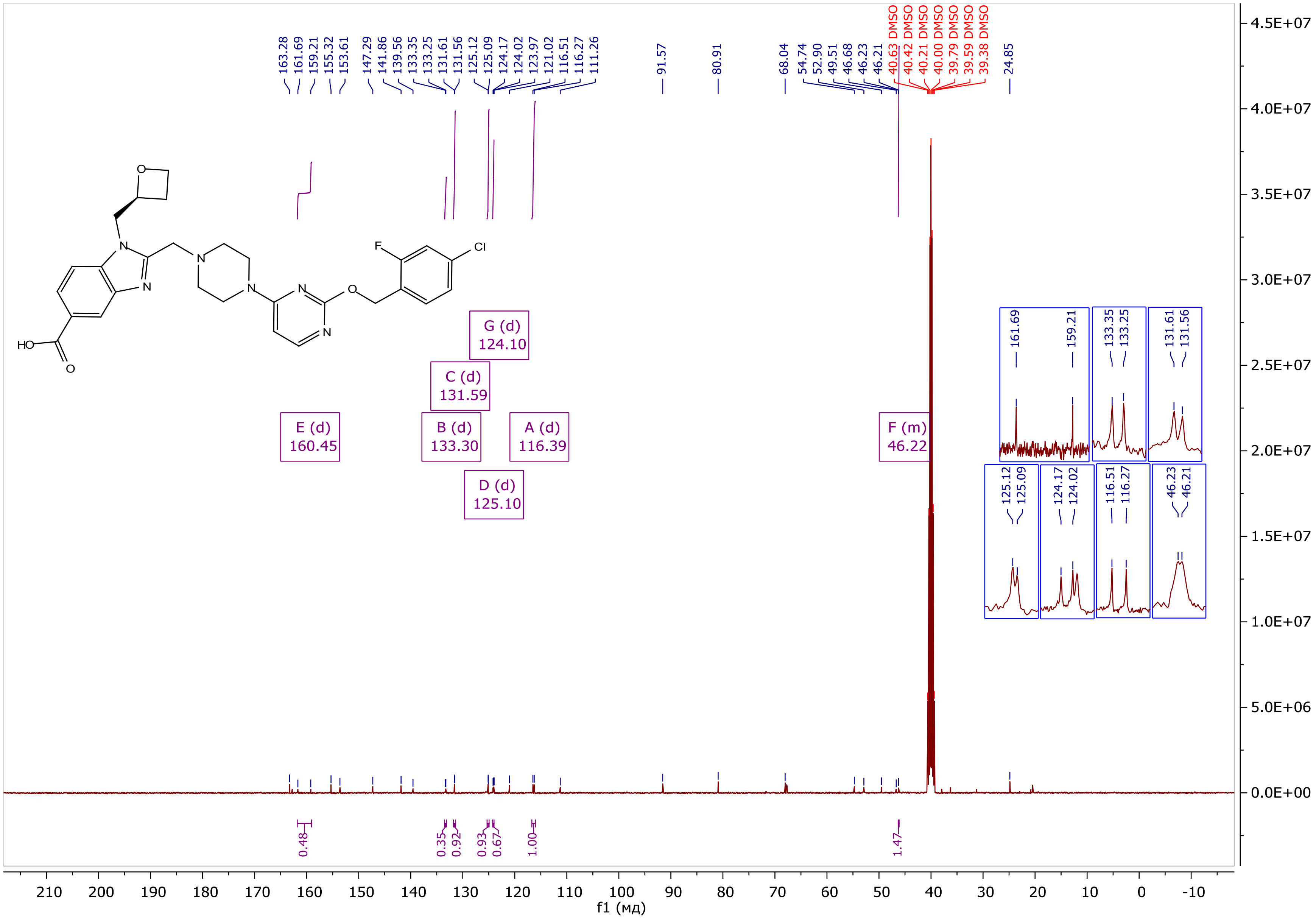

^{1}H NMR spectrum of compound **12aa'**

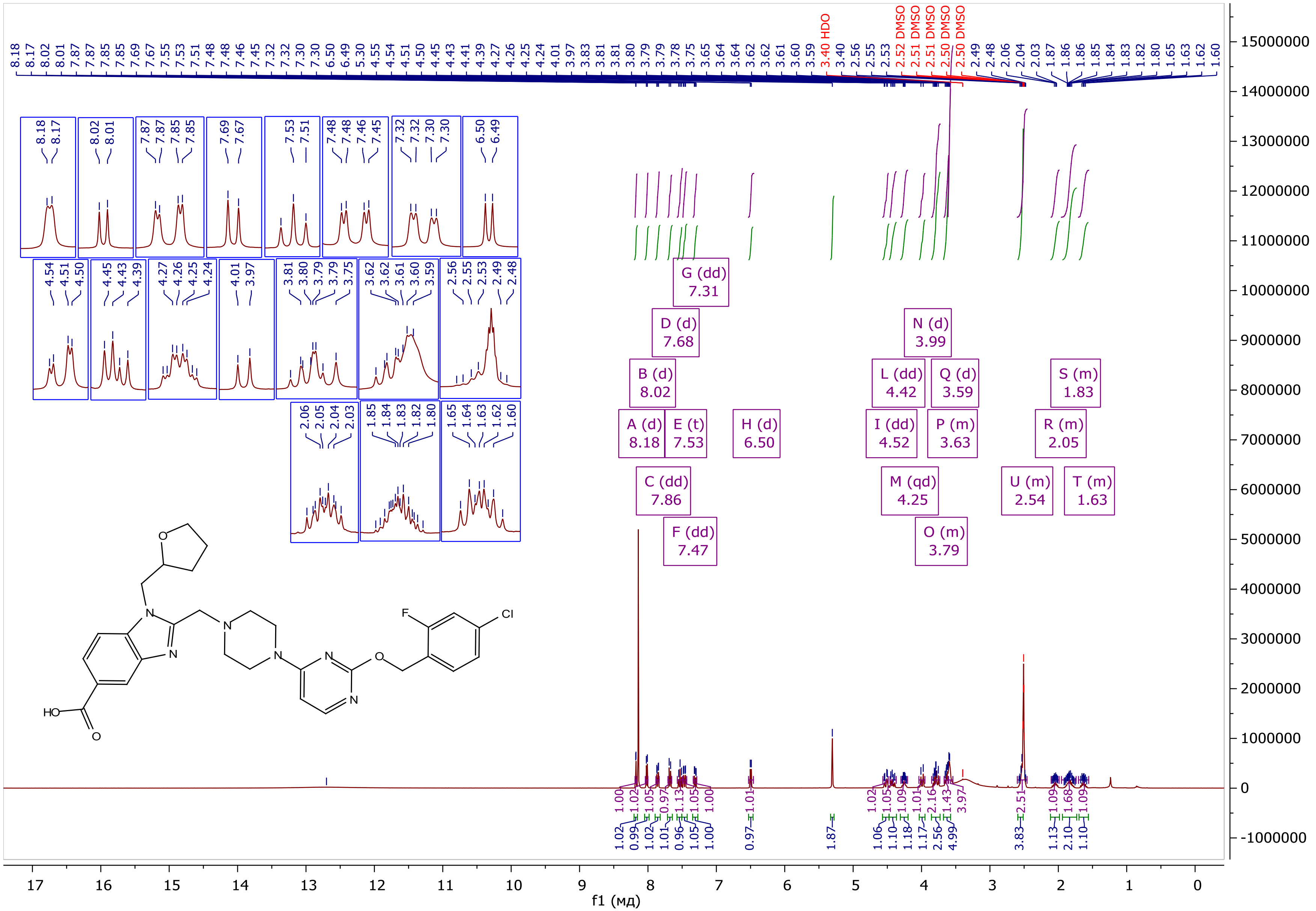

^{13}C NMR spectrum of compound **12aa'**

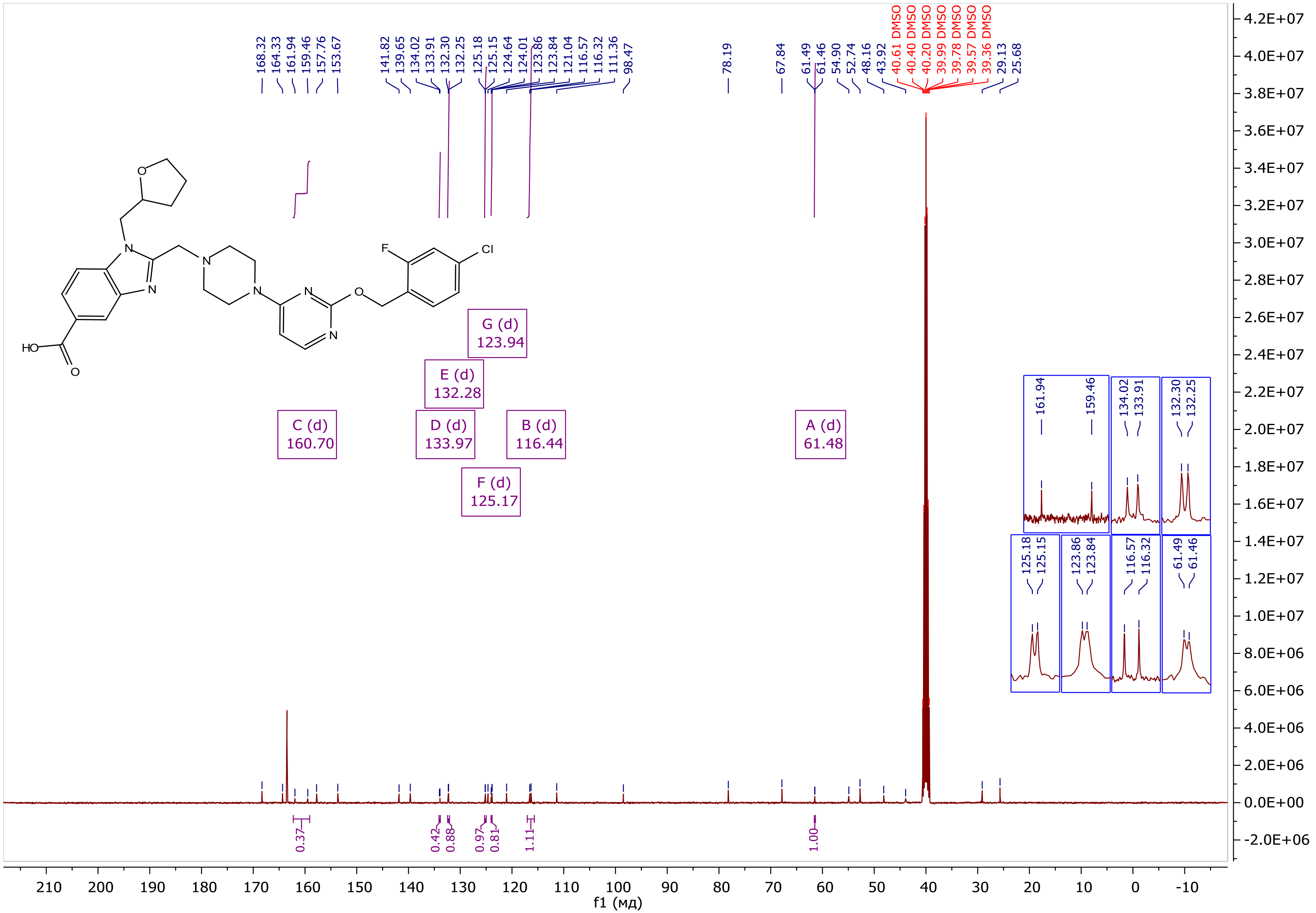

Target 5-(1*H*-benzo[*d*]imidazol-5-yl)-3-methyl-1,2,4-oxadiazole derivatives 13a-d

General procedure to synthesis of 5-(1H-benzo[d]imidazol-5-yl)-3-methyl-1,2,4-oxadiazole derivatives **13a-d**. Compounds **13a-d** were obtained from the corresponding 1,2,4-oxadiazole derivative **28a-c** and the corresponding pyrimidine derivative **22a-b**, as described above for compounds **23a-y**.

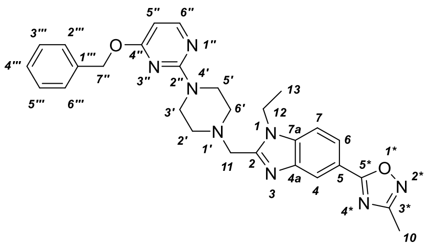

5-(2-((4-(4-(Benzyloxy)pyrimidin-2-yl)piperazin-1-yl)methyl)-1-ethyl-1H-benzo[d]imidazol-5-yl)-3-methyl-1,2,4-oxadiazole **13a**, white solid, 65 mg, 70%. **^{1}H NMR (400 MHz, CDCl$_3$, *δ ppm*)**: 8.44 (1H, *d*, *J* = 1.5 Hz, 4-CH), 8.01-7.98 (2H, *m*, 6"-CH, 6-CH), 7.41 (1H, *d*, *J* = 8.5 Hz, 7-CH), 7.32-7.21 (5H, *m*, 2'''-CH, 3'''-CH, 4'''-CH, 5'''-CH, 6'''-CH), 5.97 (1H, *d*, *J* = 5.6 Hz, 5"-CH), 5.25 (2H, *s*, 7"-CH$_2$), 4.35 (2H, *q*, *J* = 7.2 Hz, 12-CH$_2$), 3.81 (2H, *s*, 11-CH$_2$), 3.75-3.72 (4H, *m*, 3'-CH$_2$, 5'-CH$_2$), 2.55-2.52 (4H, *m*, 2'-CH$_2$, 6'-CH$_2$), 2.41 (3H, *s*, 10-CH$_3$), 1.46 (3H, *t*, *J* = 7.4 Hz, 13-CH$_3$). **^{13}C NMR (101 MHz, CDCl$_3$, *δ ppm*)**: 176.1 (C-5*), 169.2 (C-4"), 167.6 (C-3*), 161.4 (C-2"), 158.0 (C-6"), 152.7 (C-2), 142.3 (C-4a), 138.2 (C-7a), 136.7 (C-1'''), 128.4 (C-3''', C-5'''), 127.9 (C-4'''), 127.8 (C-2''', C-6'''), 122.7 (C-6), 120.3 (C-4), 118.2 (C-5), 110.0 (C-7), 97.0 (C-5"), 67.2 (C-7"), 55.7 (C-11), 53.1 (C-2', C-6'), 43.7 (C-3', C-5'), 39.2 (C-12), 15.1 (C-13), 11.7 (C-10). **HRMS** (**ESI+**): found *m/z* 511.2733 [M + H]$^+$; calculated $C_{28}H_{31}N_8O_2^+$ 511.2492.

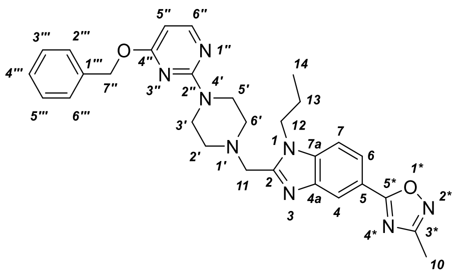

5-(2-((4-(4-(Benzyloxy)pyrimidin-2-yl)piperazin-1-yl)methyl)-1-propyl-1H-benzo[d]imidazol-5-yl)-3-methyl-1,2,4-oxadiazole **13b**, white solid, 65 mg, 75%. **^{1}H NMR (400 MHz, CDCl$_3$, *δ ppm*)**: 8.43 (1H, *d*, *J* = 1.5 Hz, 4-CH), 8.00-7.98 (2H, *m*, 6"-CH, 6-CH), 7.41 (1H, *d*, *J* = 8.5 Hz, 7-CH), 7.33-7.21 (5H, *m*, 2'''-CH, 3'''-CH, 4'''-CH, 5'''-CH, 6'''-CH), 5.97 (1H, *d*, *J* = 5.6 Hz, 5"-CH), 5.25 (2H, *s*, 7"-CH$_2$), 4.27-4.23 (2H, *m*, 12-CH$_2$), 3.81 (2H, *s*, 11-CH$_2$), 3.75-3.72 (4H, *m*, 3'-CH$_2$, 5'-CH$_2$), 2.55-2.52 (4H, *m*, 2'-CH$_2$, 6'-CH$_2$), 2.41 (3H, *s*, 10-CH$_3$), 1.89 (2H, *h*, *J* = 7.5 Hz, 13-CH$_2$), 0.97 (3H, *t*, *J* = 7.4 Hz, 14-CH$_3$). **^{13}C NMR (101 MHz, CDCl$_3$, *δ ppm*)**: 176.2 (C-5*), 169.3 (C-4"), 167.7 (C-3*), 161.5 (C-2"), 158.1 (C-6"), 153.0 (C-2), 142.4 (C-4a), 138.8 (C-7a), 136.8 (C-1'''), 128.5 (C-3''', C-5'''), 128.0 (C-4'''), 127.9 (C-2''', C-6'''), 122.7 (C-6), 120.4 (C-4), 118.2 (C-5), 110.3 (C-7), 97.1 (C-5"), 67.3 (C-7"), 55.9 (C-11), 53.2 (C-2', C-6'), 46.1 (C-12), 43.8 (C-3', C-5'), 23.2 (C-13), 11.8 (C-10), 11.5 (C-14). **HRMS** (**ESI+**): found *m/z* 525.2888 [M + H]$^+$; calculated $C_{29}H_{33}N_8O_2^+$ 525.2648.

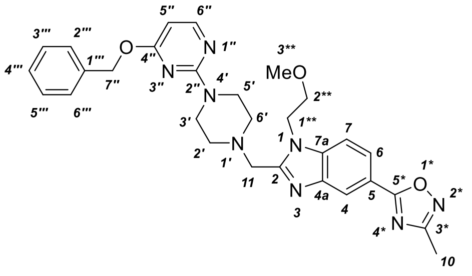

5-(2-((4-(4-(Benzyloxy)pyrimidin-2-yl)piperazin-1-yl)methyl)-1-(2-methoxyethyl)-1H-benzo[d]imidazol-5-yl)-3-methyl-1,2,4-oxadiazole **13c**, white solid, 60 mg, 70%. **^{1}H NMR (400 MHz, CDCl$_3$, *δ ppm*)**: 8.44 (1H, *d*, *J* = 1.5 Hz, 4-CH), 8.00-7.98 (2H, *m*, 6"-CH, 6-CH), 7.45 (1H, *d*, *J* = 8.5 Hz, 7-CH), 7.33-7.21 (5H, *m*, 2'''-CH, 3'''-CH, 4'''-CH, 5'''-CH, 6'''-CH), 5.97 (1H, *d*, *J* = 5.7 Hz, 5"-CH), 5.25 (2H, *s*, 7"-CH$_2$), 4.53 (2H, *t*, *J* = 5.4 Hz, 1**-CH$_2$), 3.87 (2H, *s*, 11-CH$_2$), 3.74-3.69 (6H, *m*, 3'-CH$_2$, 5'-CH$_2$, 2**-CH$_2$), 3.23 (3H, *s*, 3**-CH$_3$), 2.54-2.51 (4H, *m*, 2'-CH$_2$, 6'-CH$_2$), 2.41 (3H, *s*, 10-CH$_3$). **^{13}C NMR (101 MHz, CDCl$_3$, *δ ppm*)**: 176.2 (C-5*), 169.3 (C-4"), 167.7 (C-3*), 161.5 (C-2"), 158.0 (C-6"), 153.6 (C-2), 142.2 (C-4a), 138.9 (C-7a), 136.8 (C-1'''), 128.5 (C-3''', C-5'''), 128.0 (C-4'''), 127.9 (C-2''', C-6'''), 122.8 (C-6), 120.2 (C-4), 118.4 (C-5), 110.6 (C-7), 97.1 (C-5"), 71.3 (C-2**), 67.3 (C-7"), 59.2 (C-3**), 55.6 (C-11), 53.1 (C-2', C-6'), 44.4 (C-1**), 43.8 (C-3', C-5'), 11.8 (C-10). **HRMS** (**ESI+**): found *m/z* 541.2665 [M + H]$^+$; calculated $C_{29}H_{33}N_8O_3^+$ 541.2597.

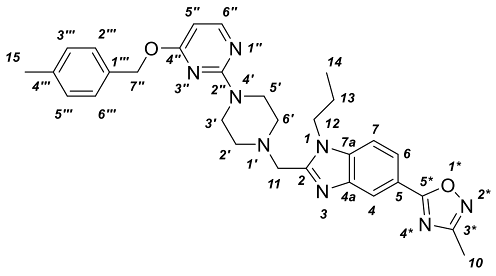

3-Methyl-5-(2-((4-(4-((4-methylbenzyl)oxy)pyrimidin-2-yl)piperazin-1-yl)methyl)-1-propyl-1H-benzo[d]imidazol-5-yl)-1,2,4-oxadiazole **13d**, white solid, 70 mg, 75%. **^{1}H NMR (400 MHz, CDCl$_3$, *δ ppm*)**: 8.44 (1H, *d*, *J* = 1.4 Hz, 4-CH), 8.01-7.97 (2H, *m*, 6"-CH, 7-CH), 7.41 (1H, *d*, *J* = 8.5 Hz, 6-CH), 7.22-7.20 (2H, *m*, 2'''-CH, 6'''-CH), 7.12-7.08 (2H, *m*, 3'''-CH, 5'''-CH), 5.95 (1H, *d*, *J* = 5.6 Hz, 5"-CH), 5.21 (2H, *s*, 7"-CH$_2$), 4.27-4.23 (2H, *m*, 12-CH$_2$), 3.83 (2H, *s*, 11-CH$_2$), 3.75-3.73 (4H, *m*, 3'-CH$_2$, 5'-CH$_2$), 2.56-2.53 (4H, *m*, 2'-CH$_2$, 6'-CH$_2$), 2.41 (3H, *s*, 10-CH$_3$), 2.27 (3H, *s*, 15-CH$_3$), 1.89 (2H, *h*, *J* = 7.3 Hz, 13-CH$_2$), 0.97 (3H, *t*, *J* = 7.4 Hz, 14-CH$_3$). **^{13}C NMR (101 MHz, CDCl$_3$, *δ ppm*)**: 176.2 (C-5*), 169.4 (C-4"), 167.7 (C-3*), 161.5 (C-2"), 158.0 (C-6"), 153.0 (C-2), 142.2 (C-4a), 138.7 (C-7a), 137.8 (C-1'''), 133.7 (C-4'''), 129.2 (C-3''', C-5'''), 128.1 (C-2''', C-6'''), 122.7 (C-7), 120.3 (C-5), 118.3 (C-4), 110.4 (C-6), 97.2 (C-5"), 67.3 (C-7"), 55.8 (C-11), 53.2 (C-2', C-6'), 46.1 (C-12), 43.8 (C-3', C-5'), 23.2 (C-13), 21.2 (C-15), 11.8 (C-10), 11.5 (C-14). **HRMS** (**ESI+**): found *m/z* 539.2868 [M + H]$^+$; calculated $C_{30}H_{35}N_8O_2^+$ 539.2805.

^{1}H NMR spectrum of compound **13a**

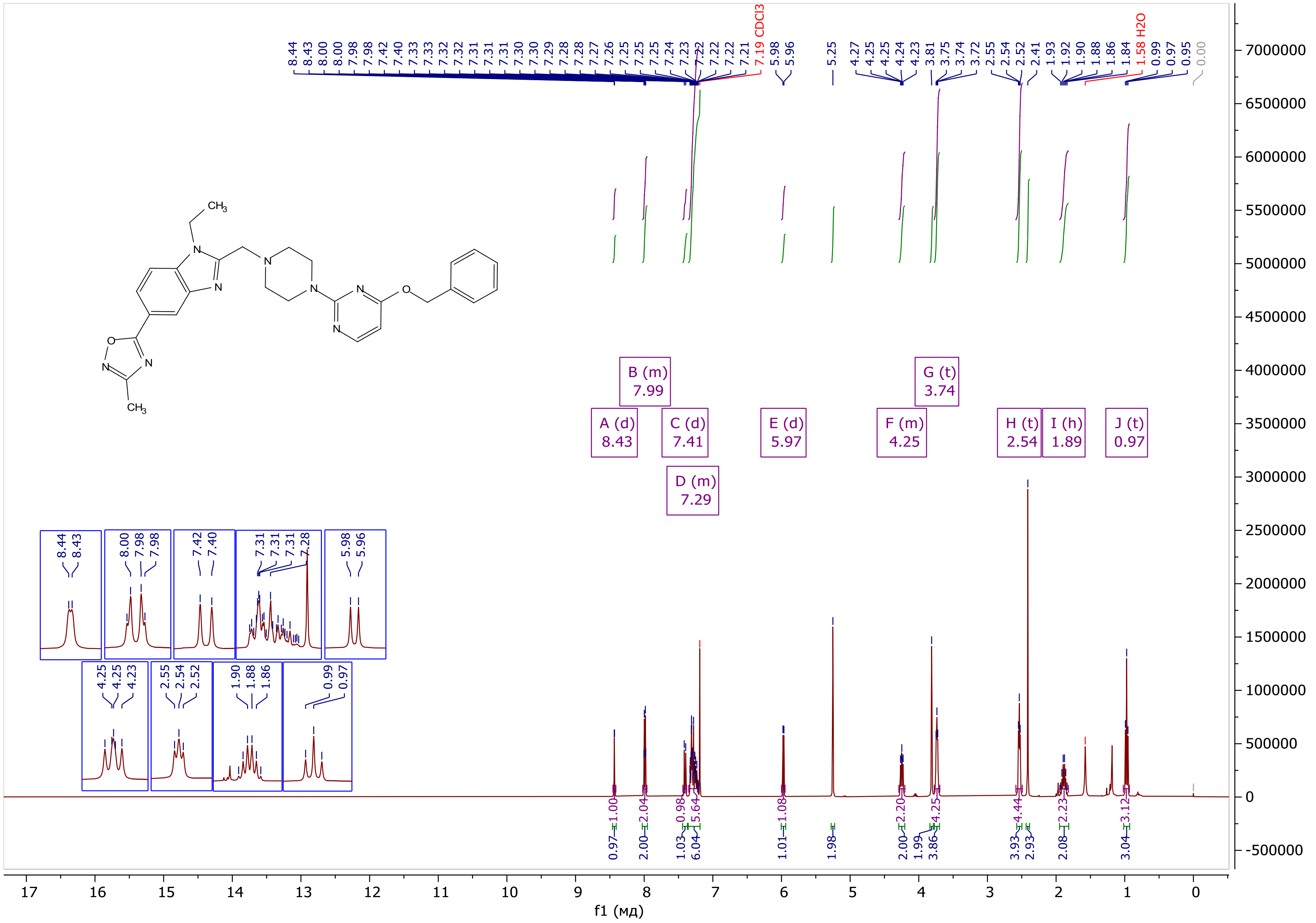

^{13}C NMR spectrum of compound **13a**

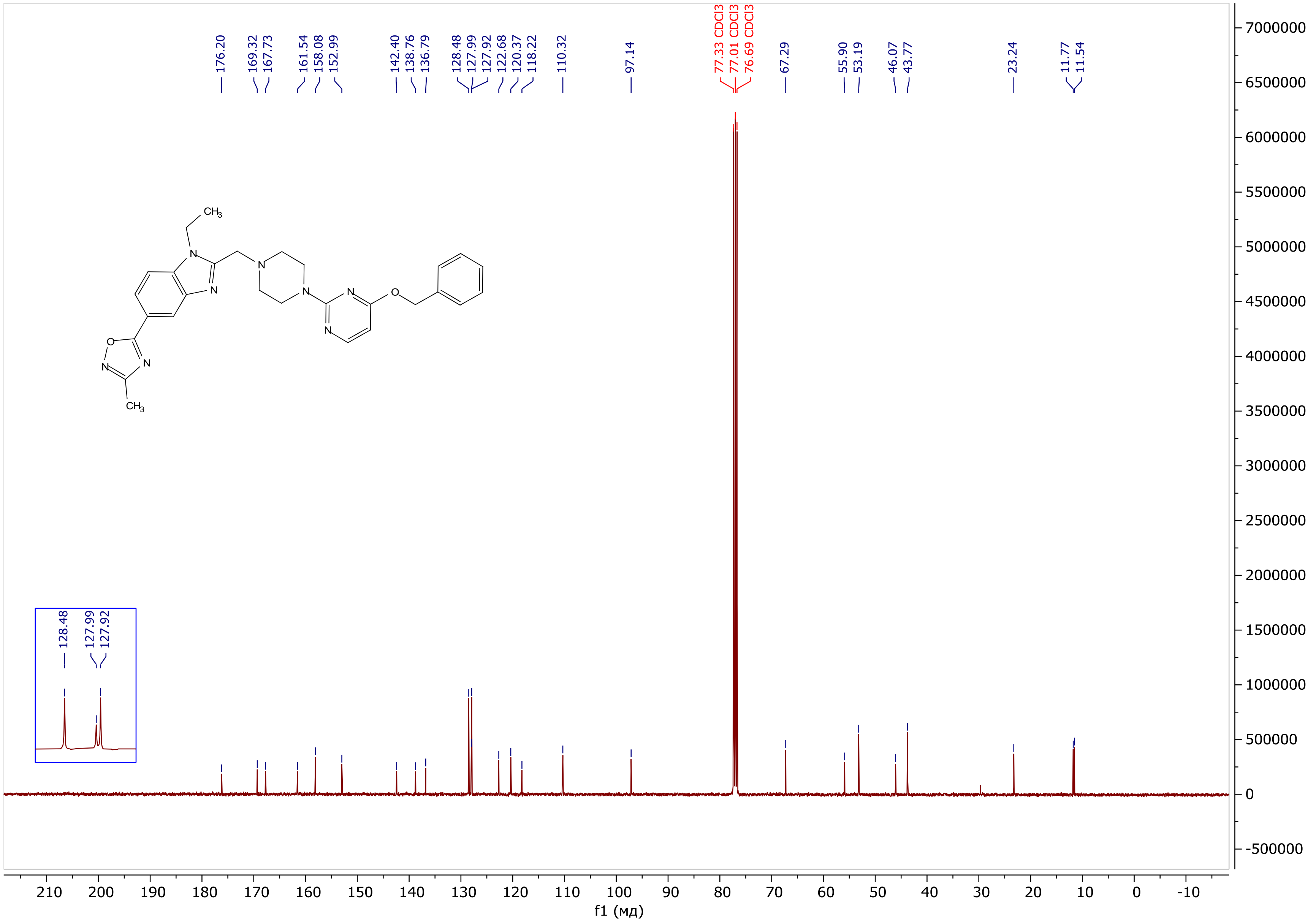

^{1}H NMR spectrum of compound **13b**

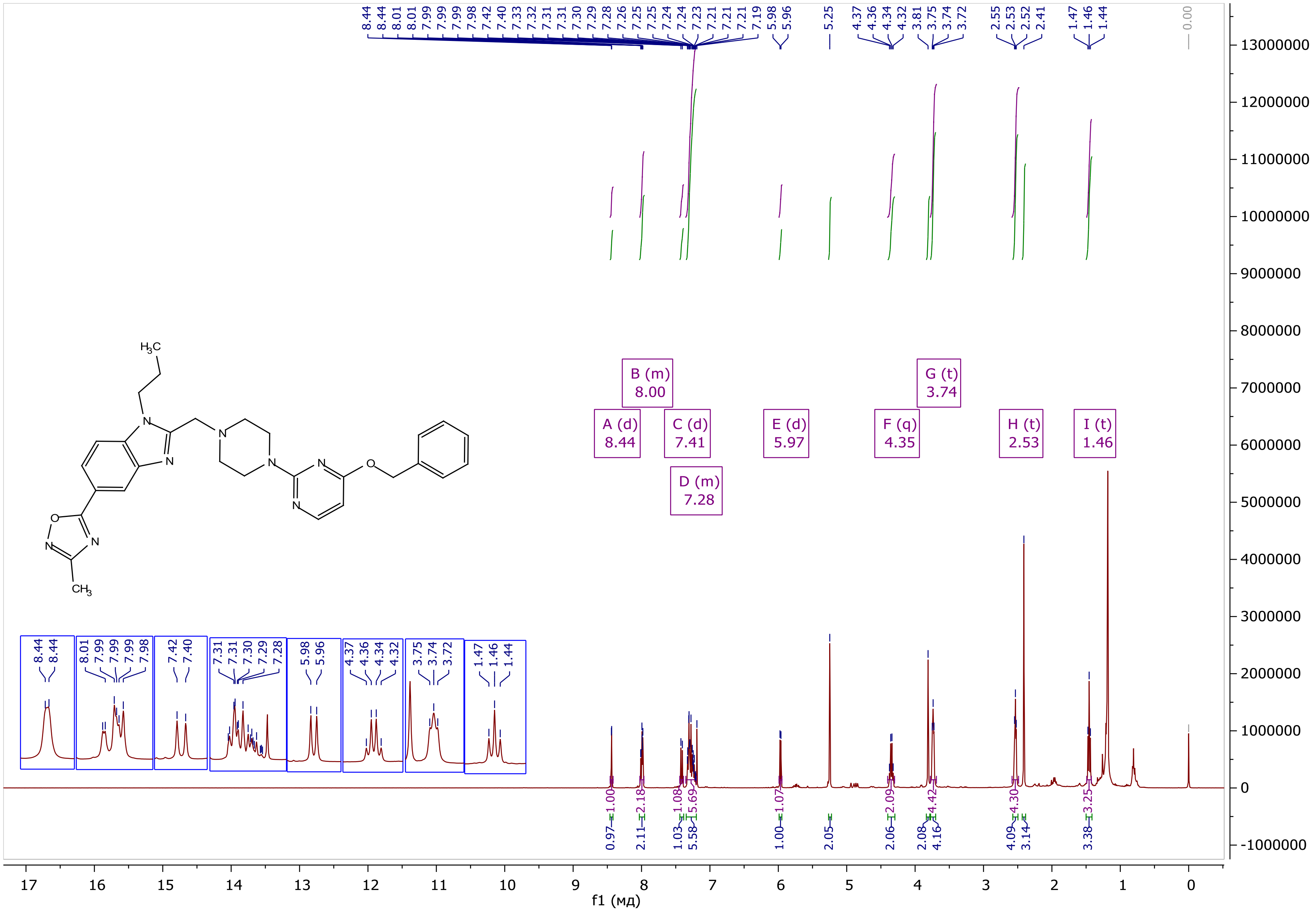

^{13}C NMR spectrum of compound **13b**

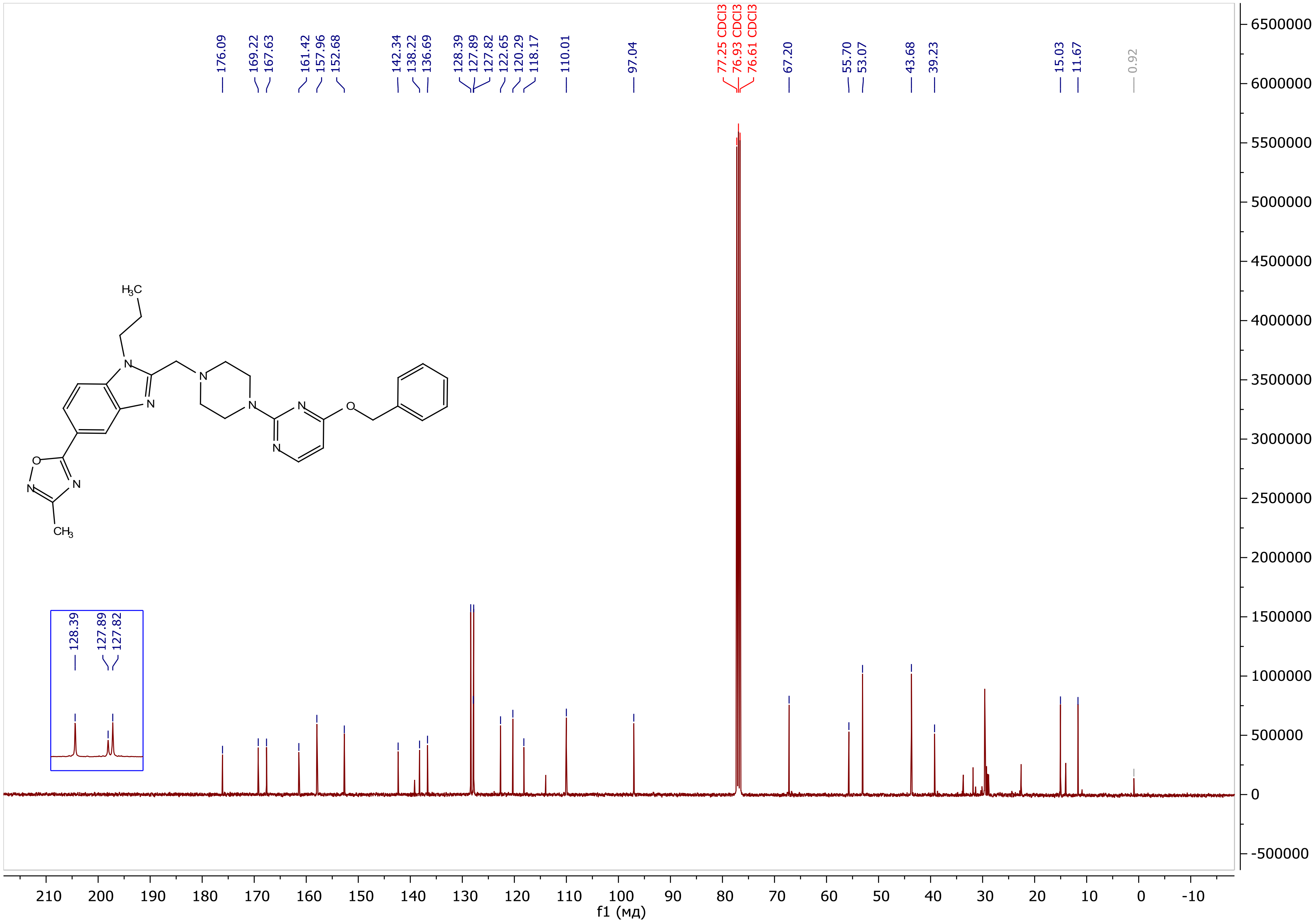

^{1}H NMR spectrum of compound **13c**

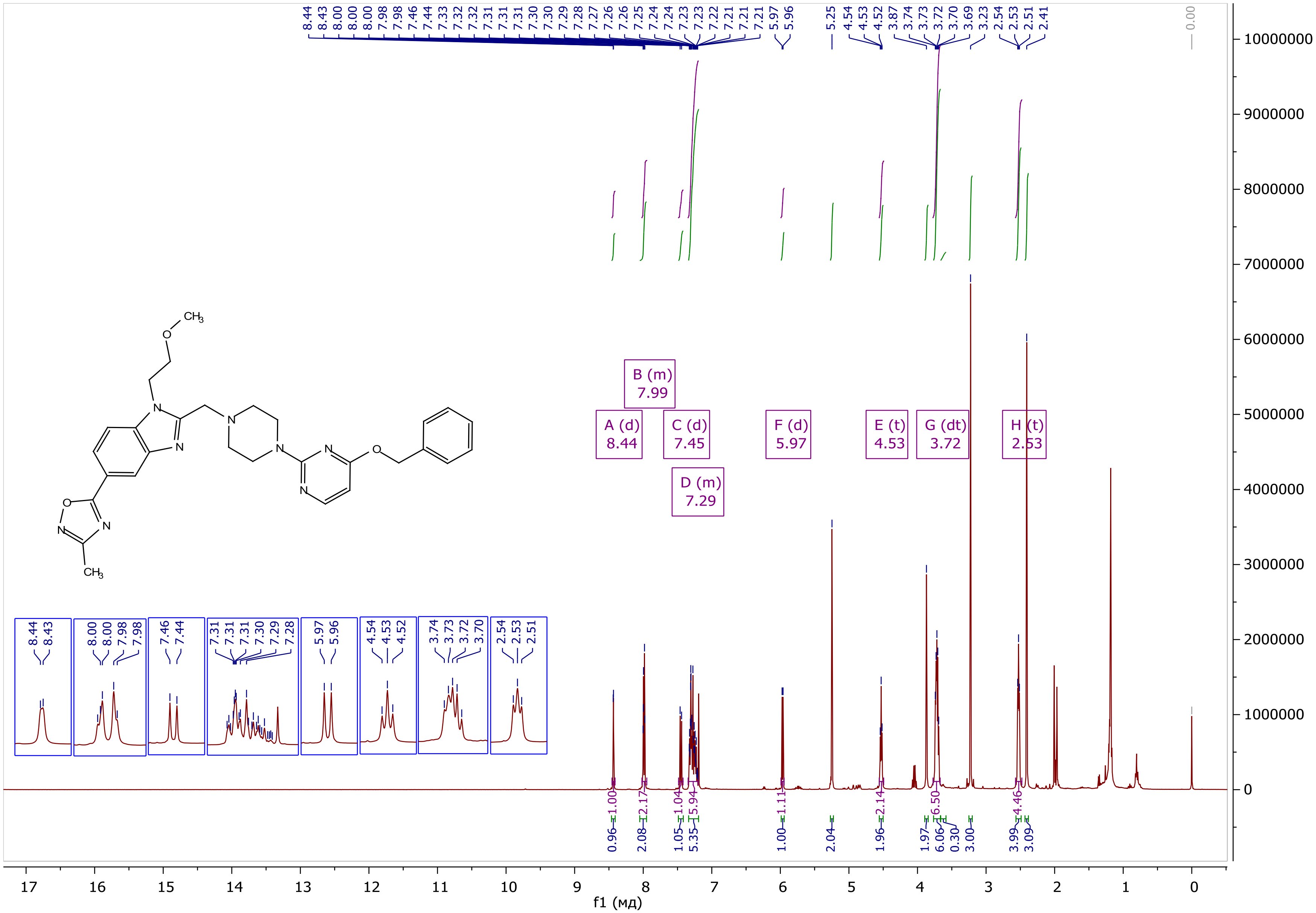

^{13}C NMR spectrum of compound **13c**

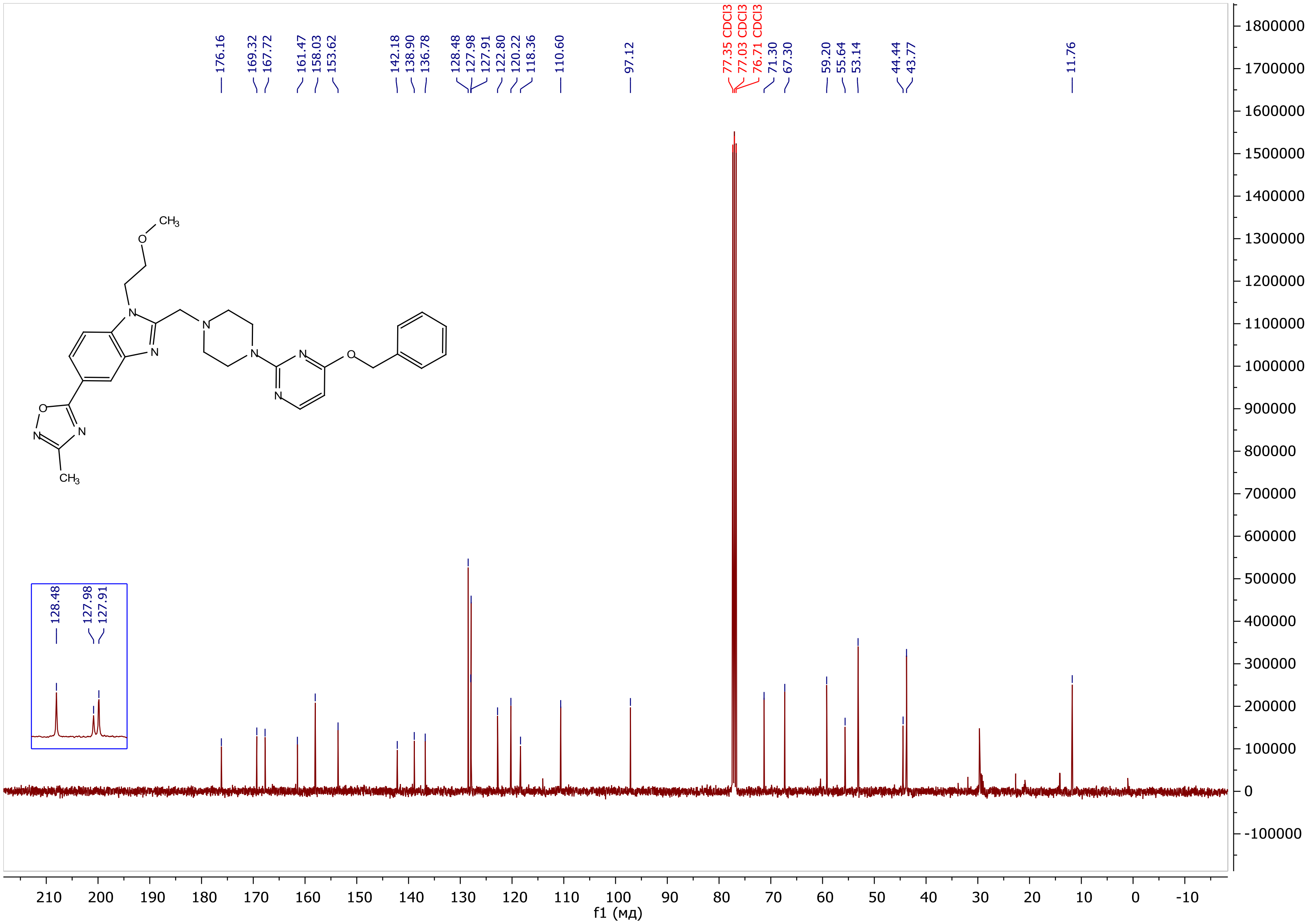

^{1}H NMR spectrum of compound **13d**

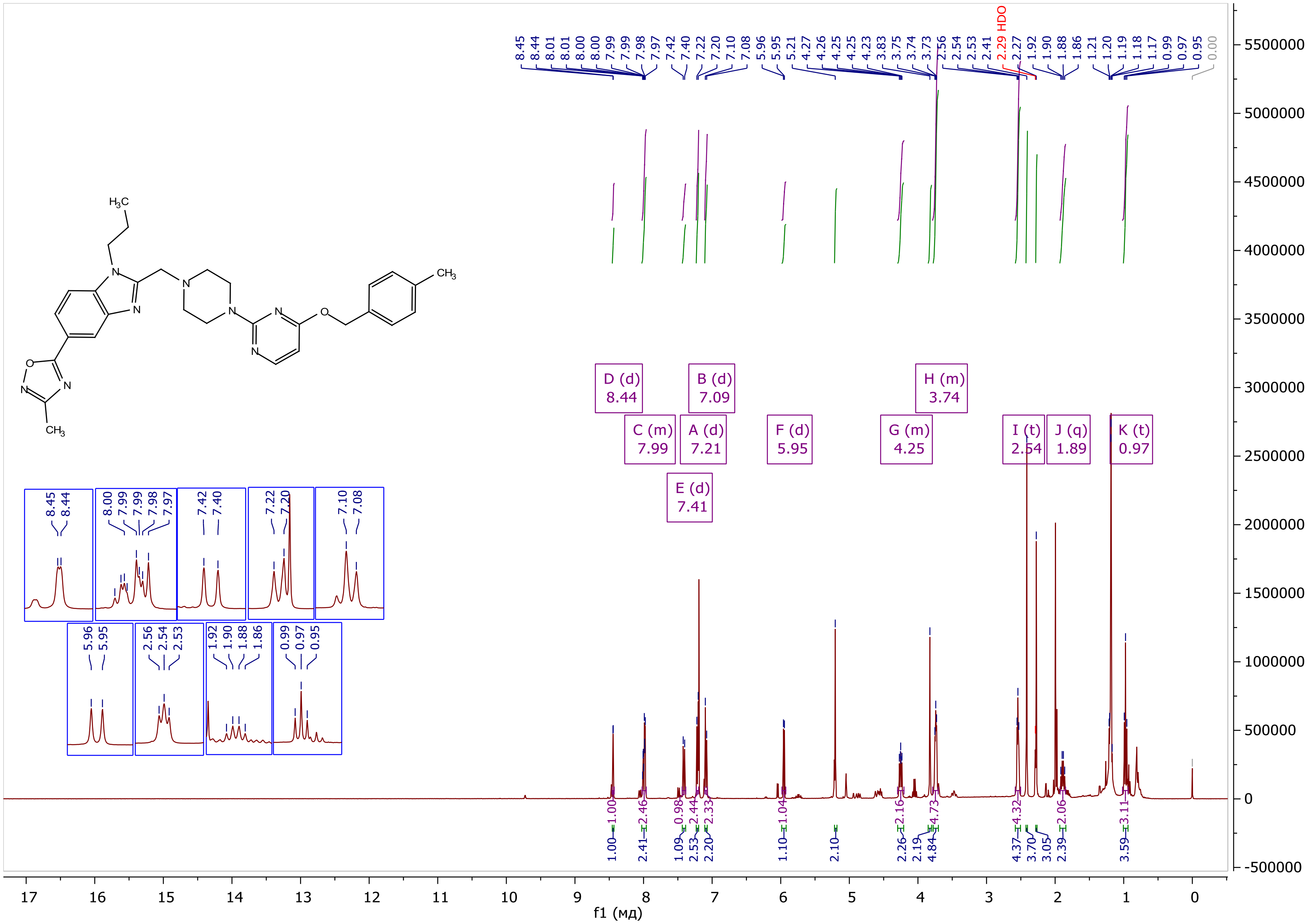

^{13}C NMR spectrum of compound **13d**

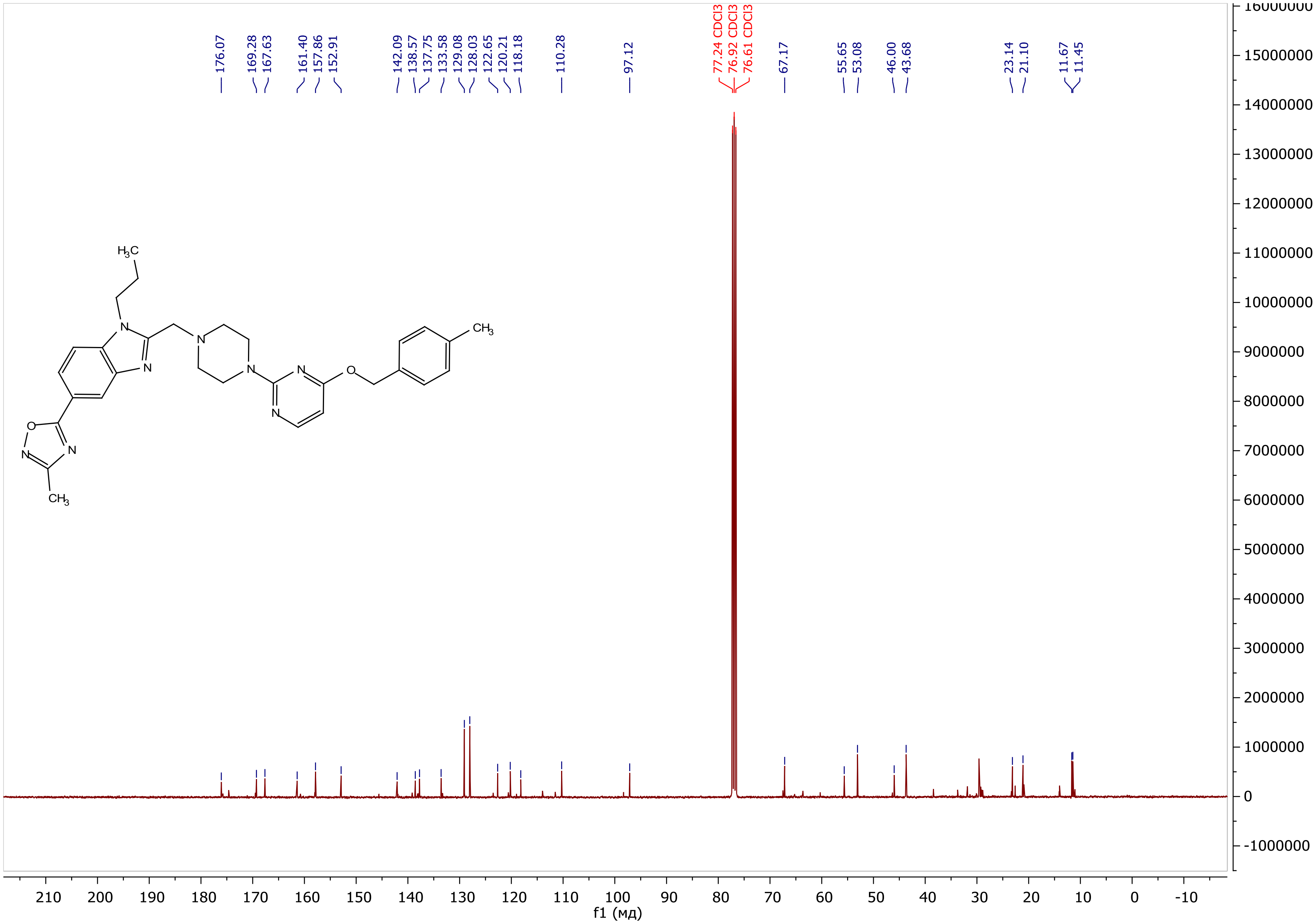

HRMS spectra copies

Compound	HRMS spectra (MS1, MS2)
**18a**	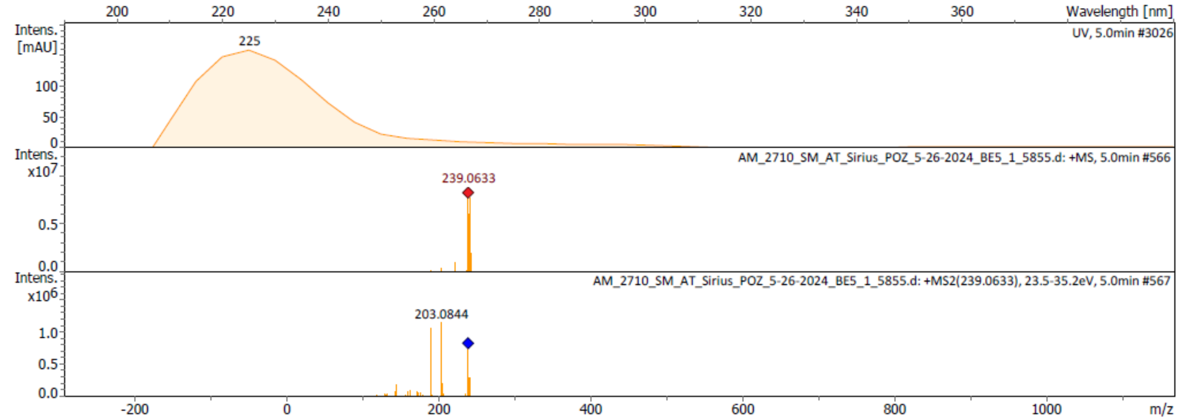
**18b**	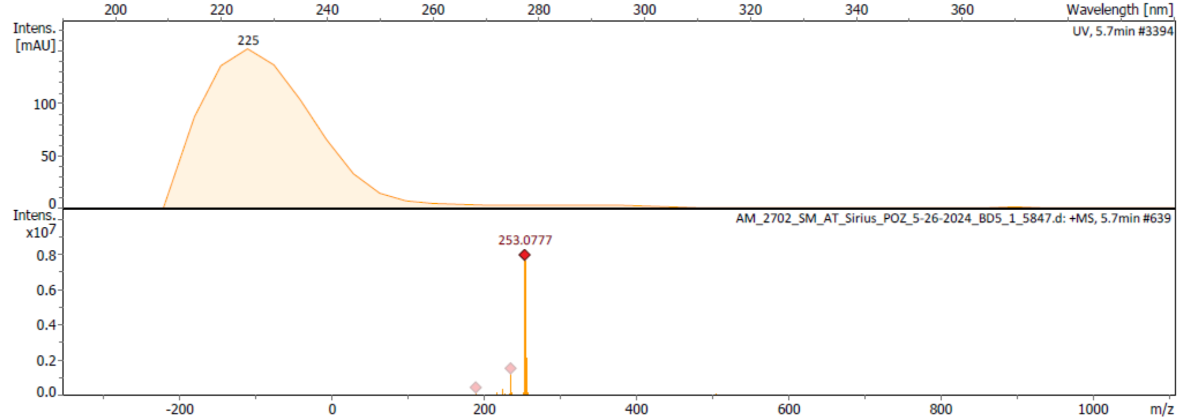
**18c**	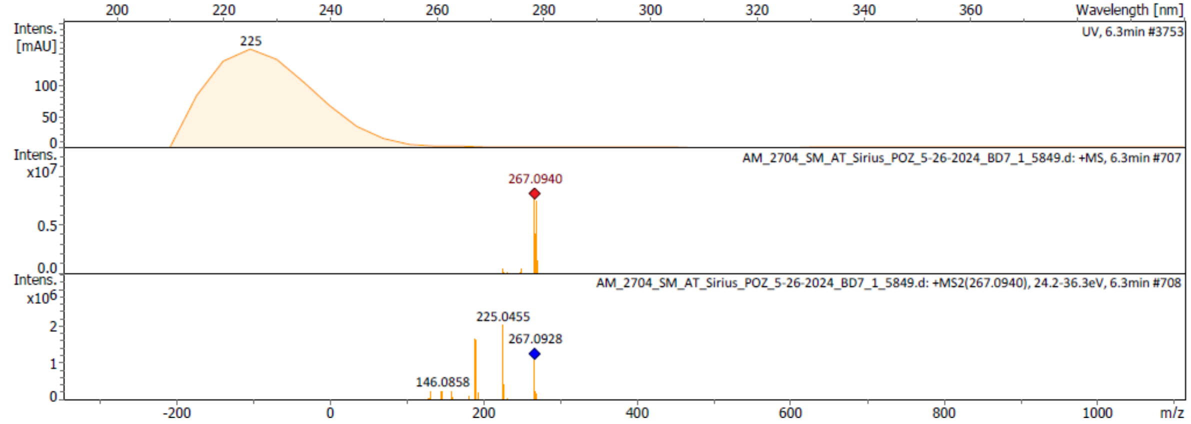

18d

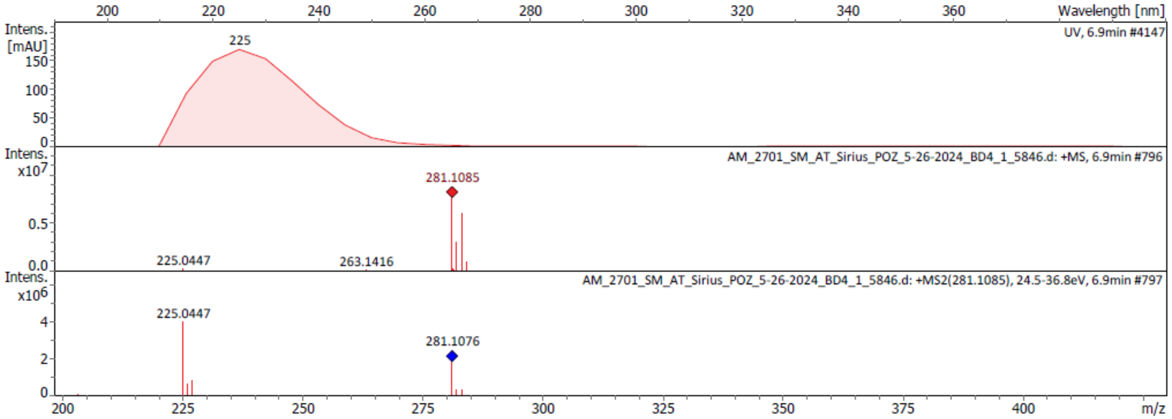
Wavelength [nm]
Intens. [mAU]
UV, 6.9min #4147
225
AM_2701_SM_AT_Sirius_POZ_5-26-2024_BD4_1_5846.d: +MS, 6.9min #796
281.1085
225.0447
263.1416
AM_2701_SM_AT_Sirius_POZ_5-26-2024_BD4_1_5846.d: +MS2(281.1085), 24.5-36.8eV, 6.9min #797
225.0447
281.1076
m/z

18e

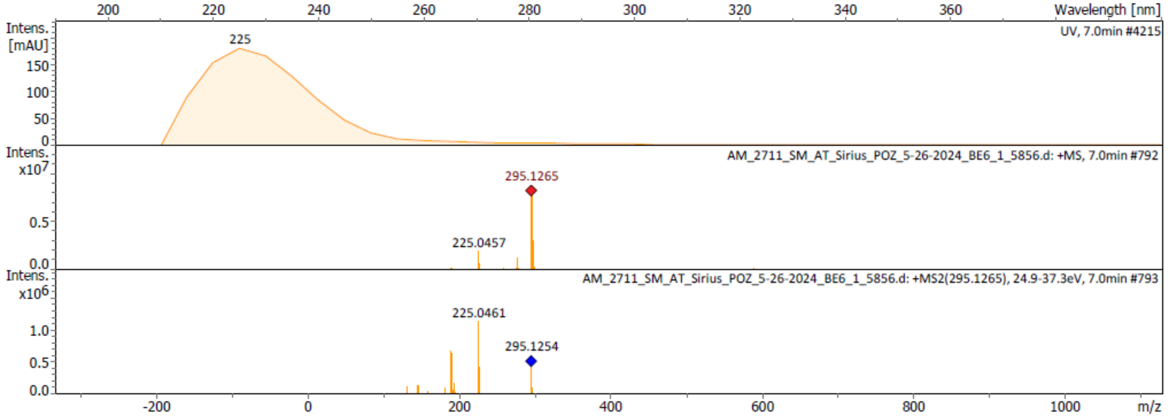
Wavelength [nm]
Intens. [mAU]
UV, 7.0min #4215
225
AM_2711_SM_AT_Sirius_POZ_5-26-2024_BE6_1_5856.d: +MS, 7.0min #792
295.1265
225.0457
AM_2711_SM_AT_Sirius_POZ_5-26-2024_BE6_1_5856.d: +MS2(295.1265), 24.9-37.3eV, 7.0min #793
225.0461
295.1254
m/z

18f

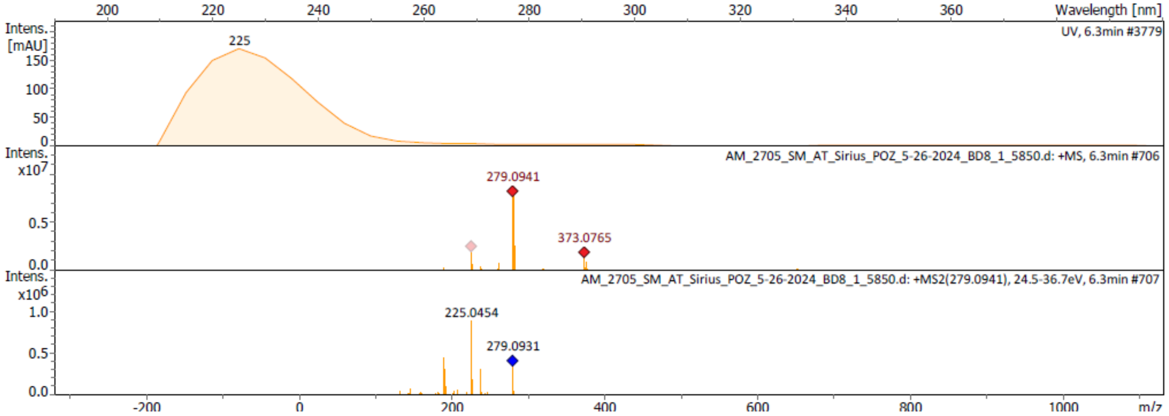
Wavelength [nm]
Intens. [mAU]
UV, 6.3min #3779
225
AM_2705_SM_AT_Sirius_POZ_5-26-2024_BD8_1_5850.d: +MS, 6.3min #706
279.0941
373.0765
AM_2705_SM_AT_Sirius_POZ_5-26-2024_BD8_1_5850.d: +MS2(279.0941), 24.5-36.7eV, 6.3min #707
225.0454
279.0931
m/z

18g

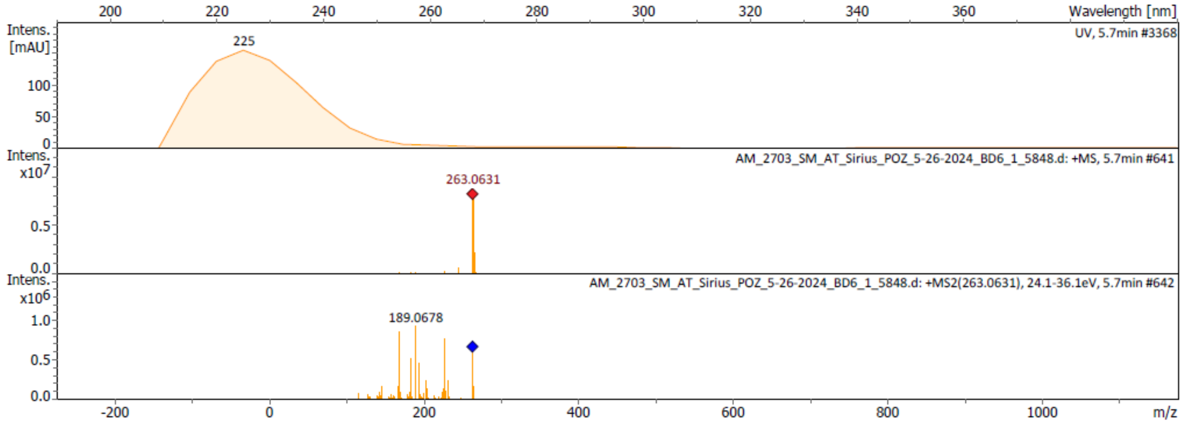
Wavelength [nm]
UV, 5.7min #3368
225
AM_2703_SM_AT_Sirius_POZ_5-26-2024_BD6_1_5848.d: +MS, 5.7min #641
263.0631
AM_2703_SM_AT_Sirius_POZ_5-26-2024_BD6_1_5848.d: +MS2(263.0631), 24.1-36.1eV, 5.7min #642
189.0678
Intens. [mAU]
Intens. x10^7
Intens. x10^6
m/z

18h

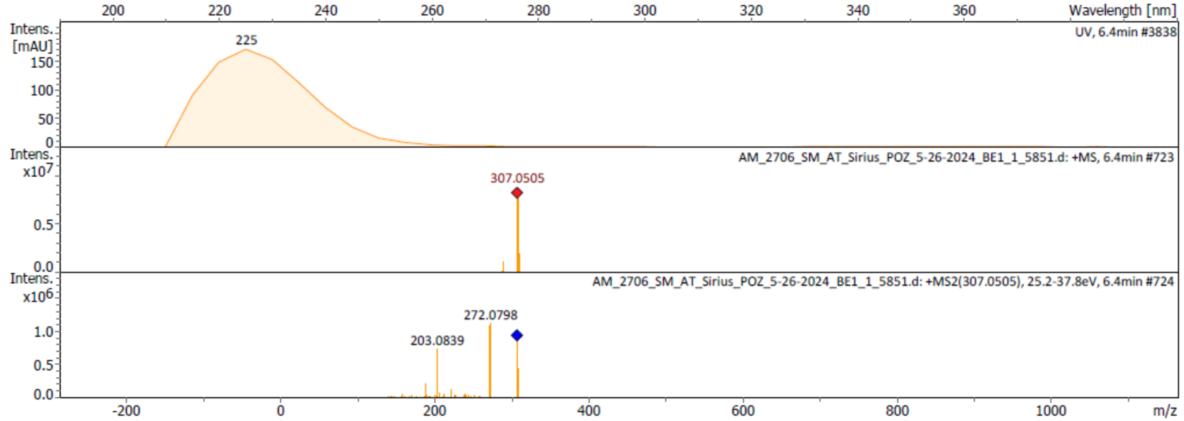
Wavelength [nm]
UV, 6.4min #3838
225
AM_2706_SM_AT_Sirius_POZ_5-26-2024_BE1_1_5851.d: +MS, 6.4min #723
307.0505
AM_2706_SM_AT_Sirius_POZ_5-26-2024_BE1_1_5851.d: +MS2(307.0505), 25.2-37.8eV, 6.4min #724
272.0798
203.0839
Intens. [mAU]
Intens. x10^7
Intens. x10^6
m/z

18i

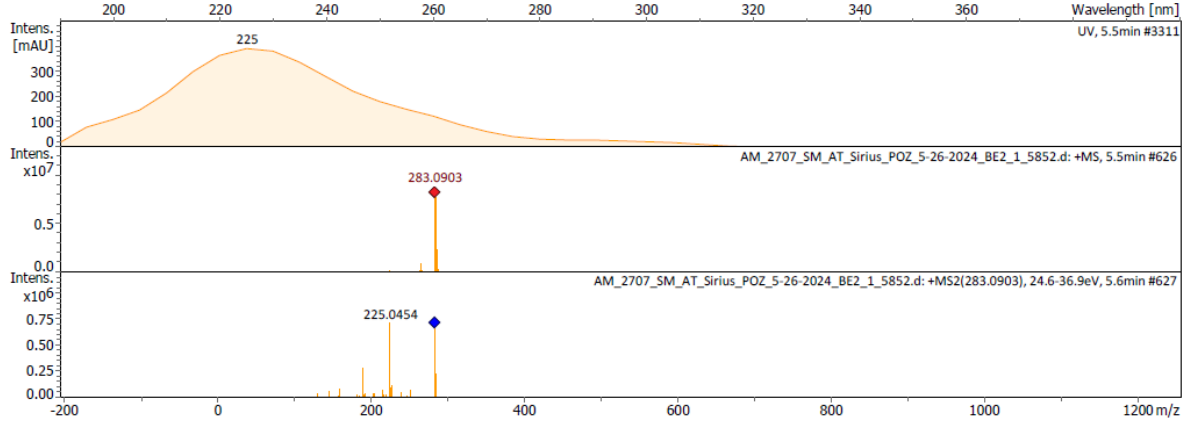
Wavelength [nm]
UV, 5.5min #3311
225
AM_2707_SM_AT_Sirius_POZ_5-26-2024_BE2_1_5852.d: +MS, 5.5min #626
283.0903
AM_2707_SM_AT_Sirius_POZ_5-26-2024_BE2_1_5852.d: +MS2(283.0903), 24.6-36.9eV, 5.6min #627
225.0454
Intens. [mAU]
Intens. x10^7
Intens. x10^6
m/z

18j

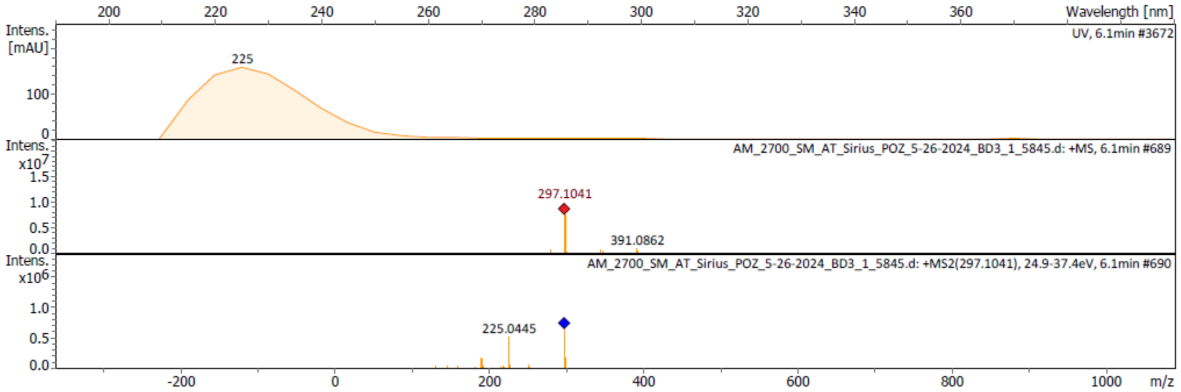

Wavelength [nm]
Intens. [mAU]
UV, 6.1min #3672
225
AM_2700_SM_AT_Sirius_POZ_5-26-2024_BD3_1_5845.d: +MS, 6.1min #689
297.1041
391.0862
AM_2700_SM_AT_Sirius_POZ_5-26-2024_BD3_1_5845.d: +MS2(297.1041), 24.9-37.4eV, 6.1min #690
225.0445
m/z

18k

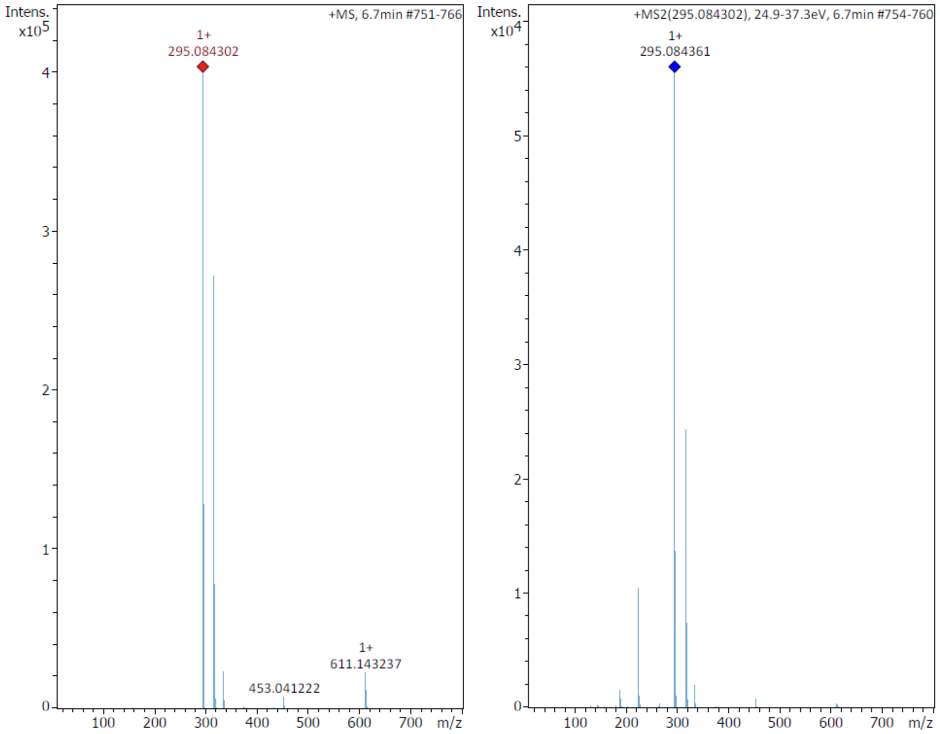

+MS, 6.7min #751-766
1+
295.084302
453.041222
1+
611.143237
+MS2(295.084302), 24.9-37.3eV, 6.7min #754-760
1+
295.084361
m/z

18l

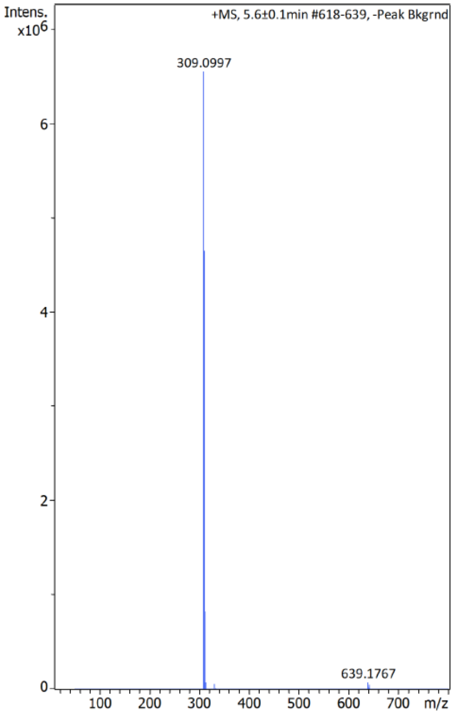

+MS, 5.6±0.1min #618-639, -Peak Bkgrnd
309.0997
639.1767
m/z

Compound	HRMS spectra (MS1, MS2)
**28a**	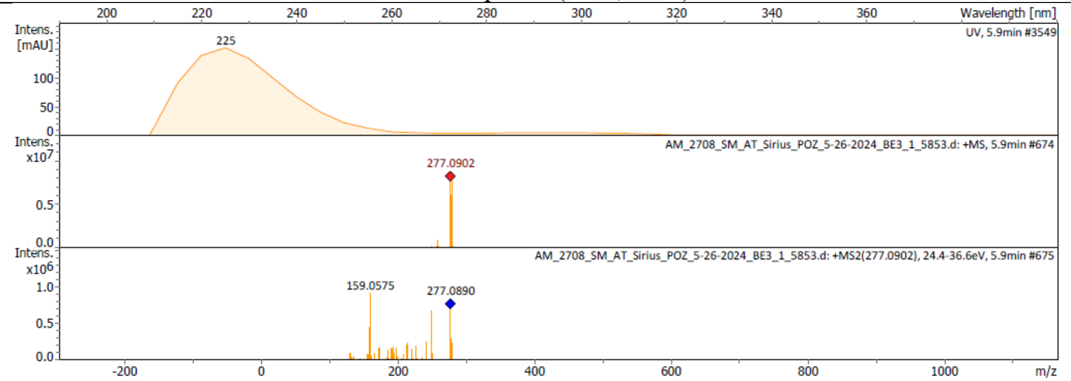
**28b**	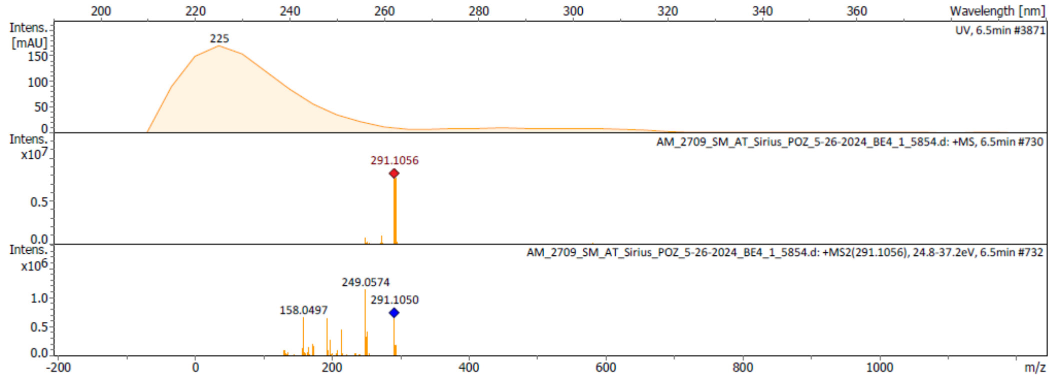
**28c**	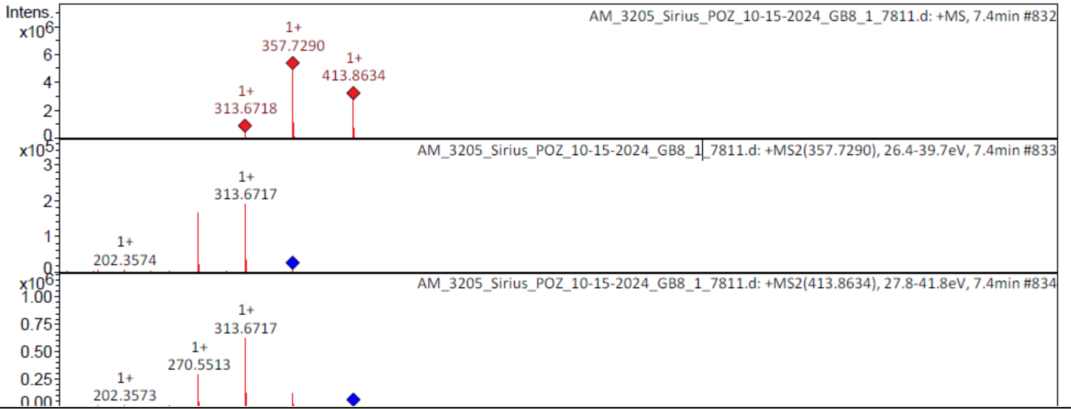

Cmpd	HRMS spectra (MS1, MS2)	Cmpd	HRMS spectra (MS1, MS2)
**21d**	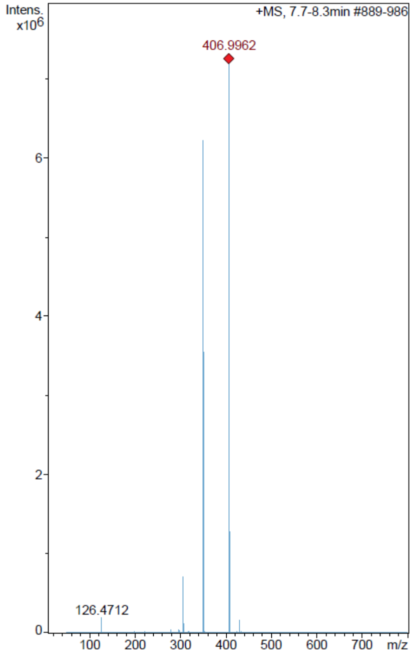 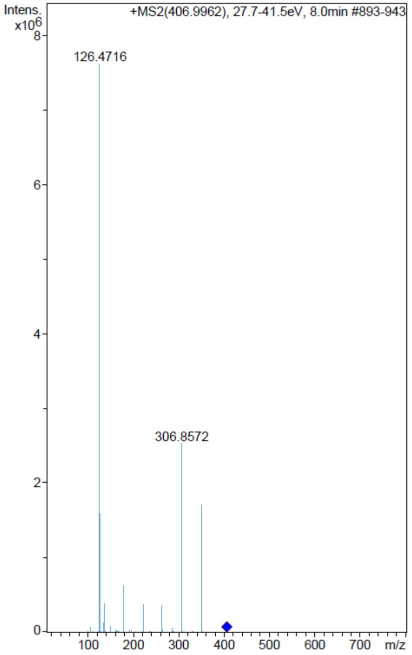	**21e**	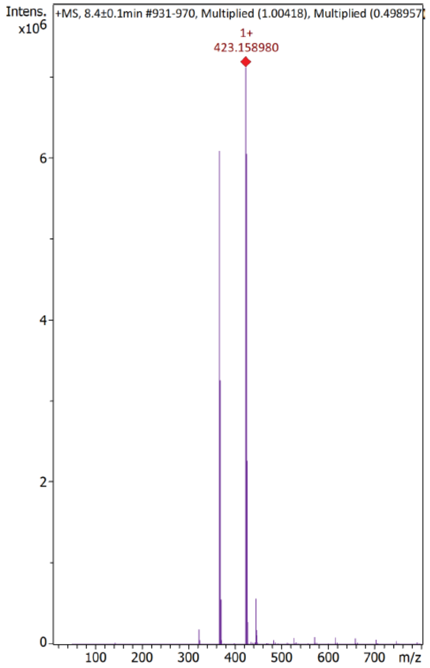 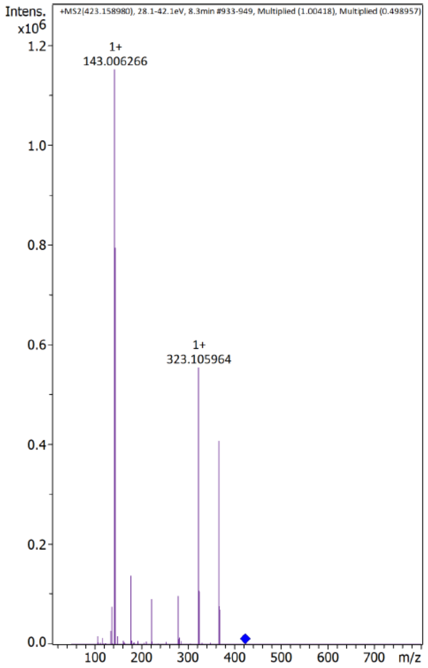
**21c'**	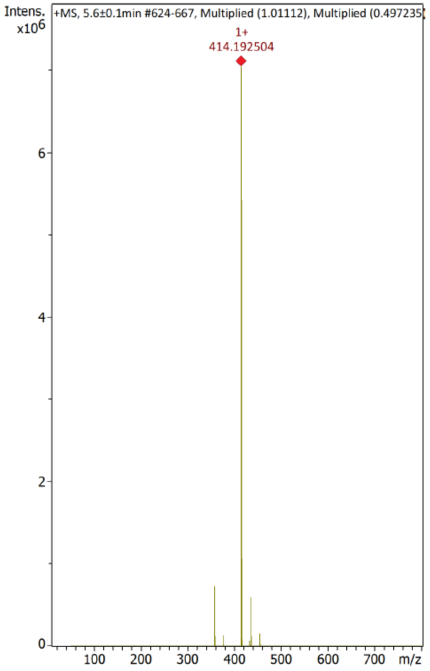 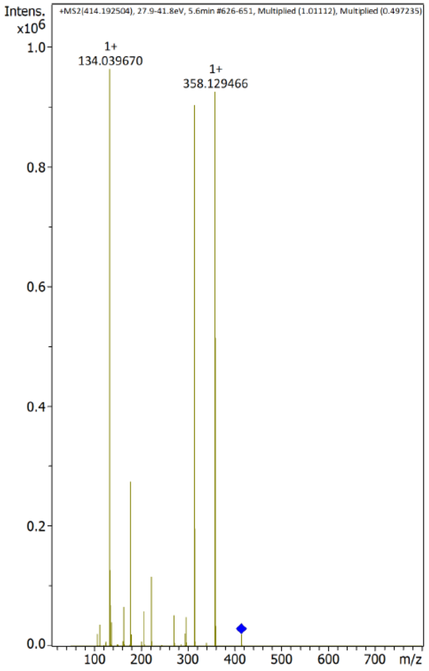	**21e'**	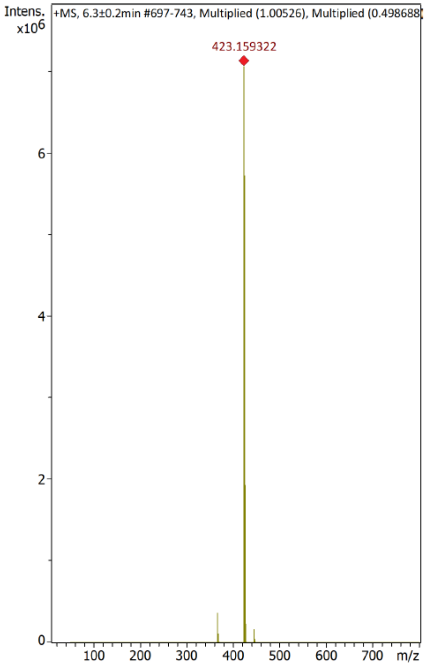 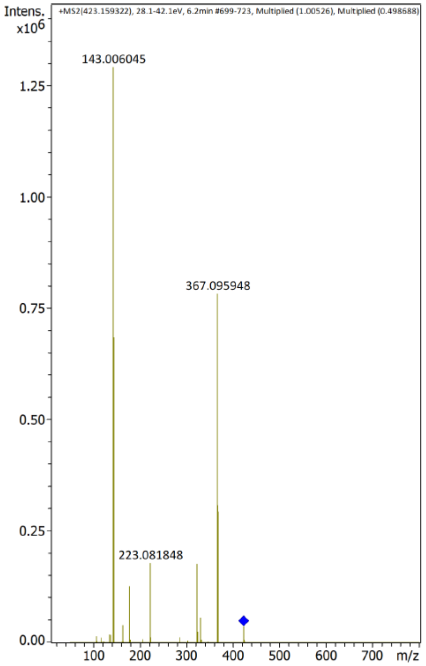

22c

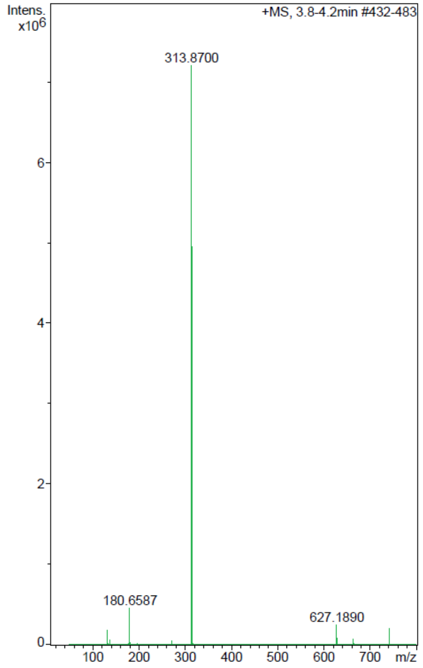

22d

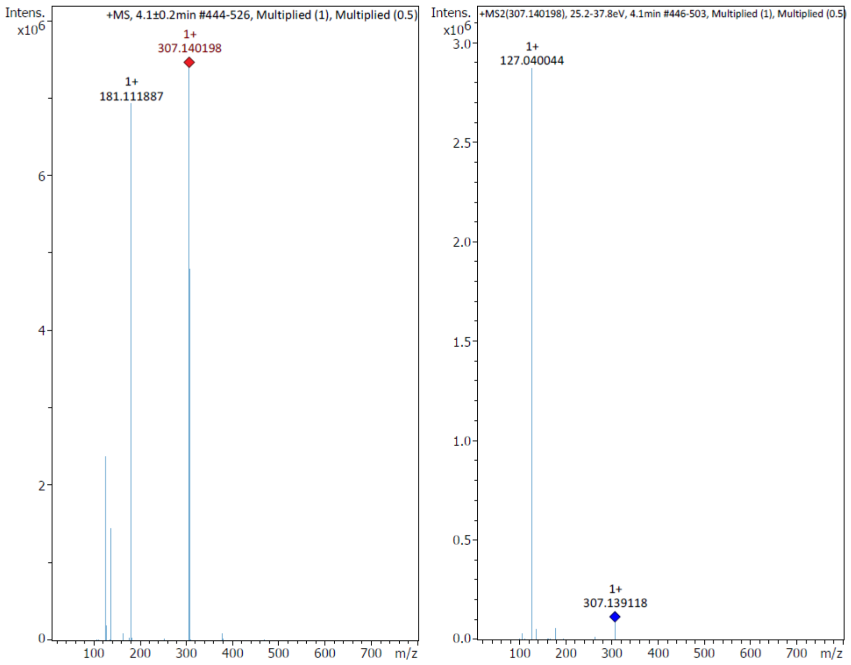

Compound	HRMS spectra (MS1, MS2)
**23a**	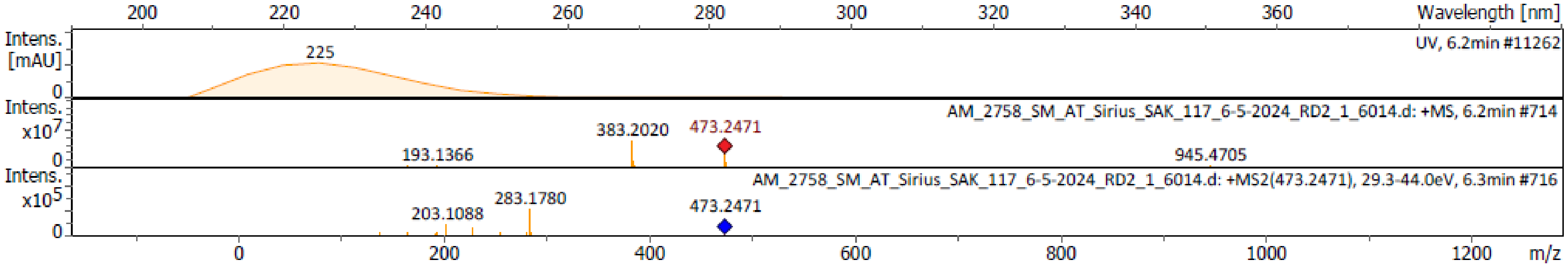
**23b**	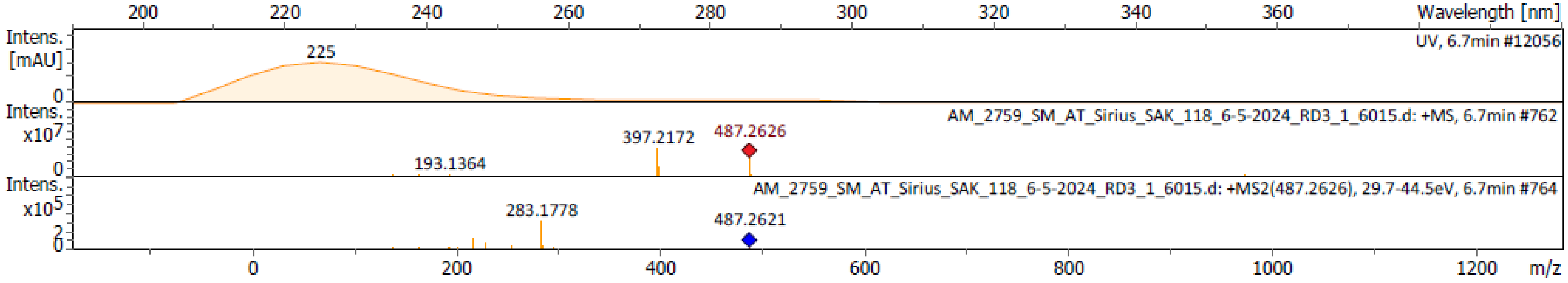

23c

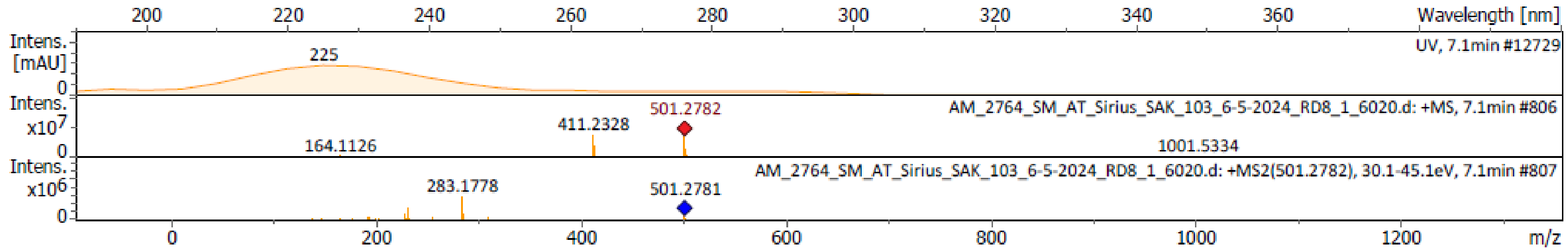

23d

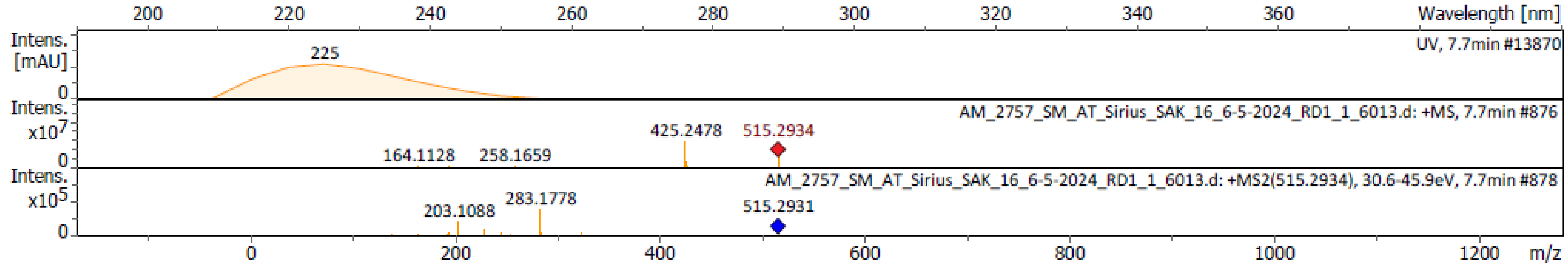

23e

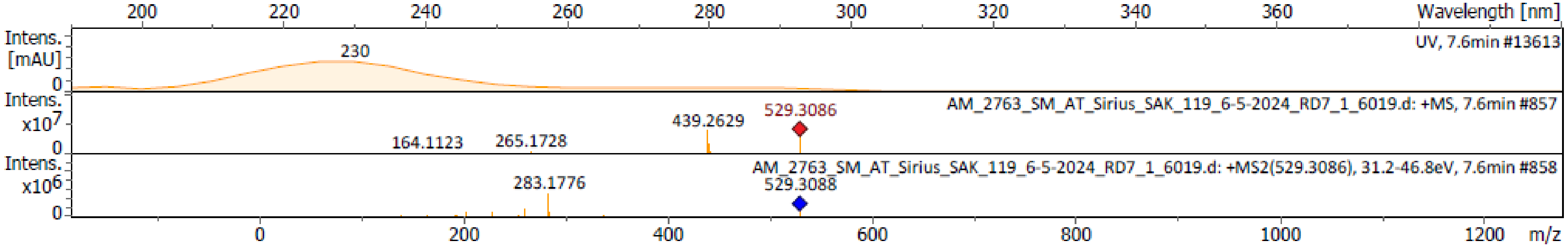

23f

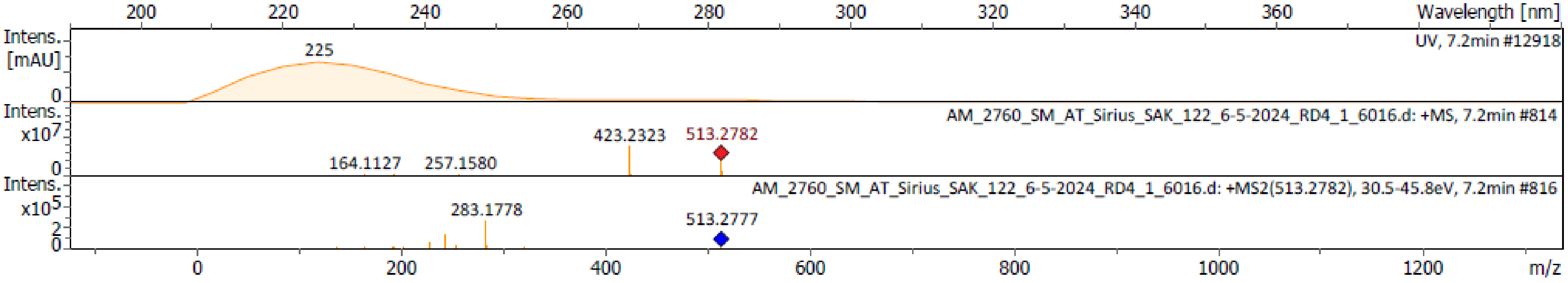

23g

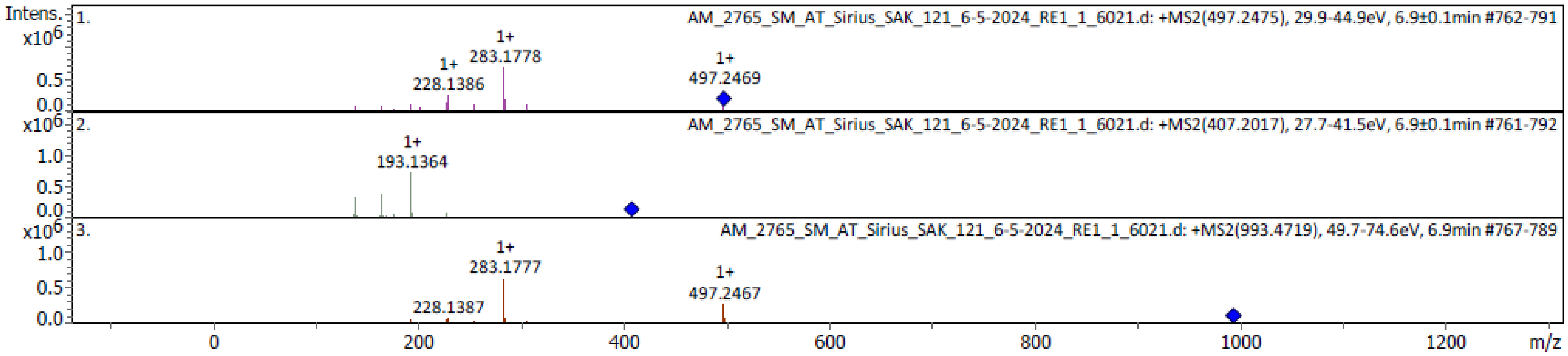

23h

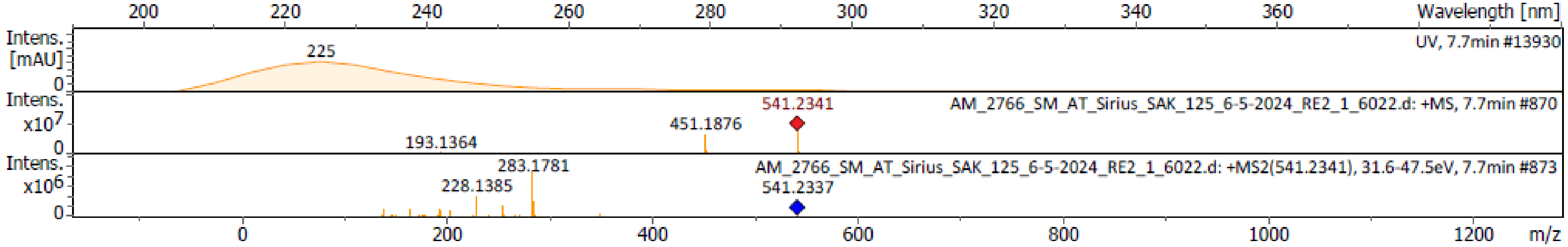

23i

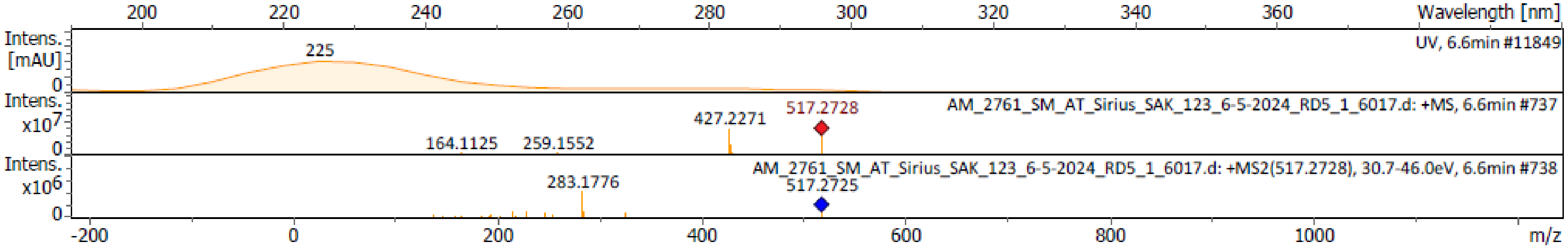

23j

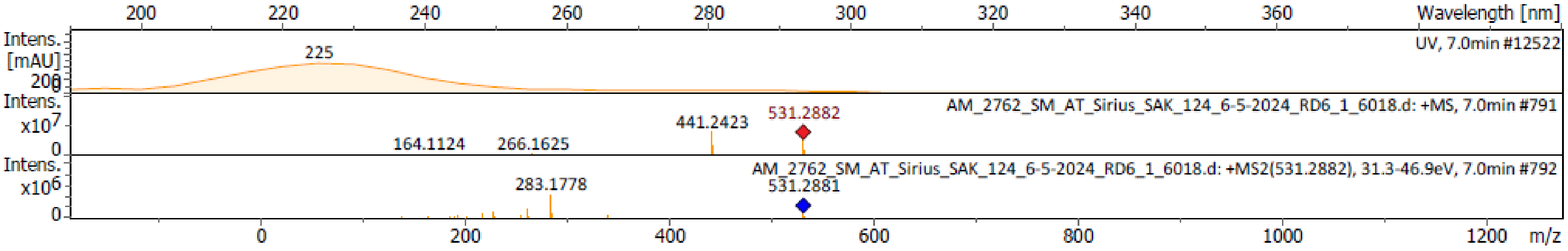

23k

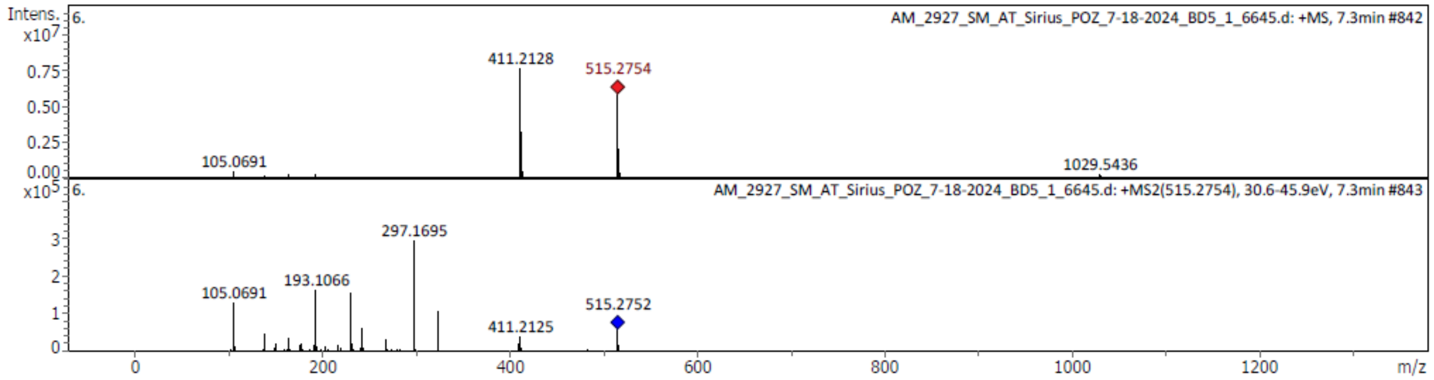
Intens.
x10^7
6.
AM_2927_SM_AT_Sirius_POZ_7-18-2024_BD5_1_6645.d: +MS, 7.3min #842
411.2128
515.2754
105.0691
1029.5436
0.75
0.50
0.25
0.00
x10^5
6.
AM_2927_SM_AT_Sirius_POZ_7-18-2024_BD5_1_6645.d: +MS2(515.2754), 30.6-45.9eV, 7.3min #843
297.1695
193.1066
105.0691
411.2125
515.2752
3
2
1
0
0
200
400
600
800
1000
1200
m/z

23l

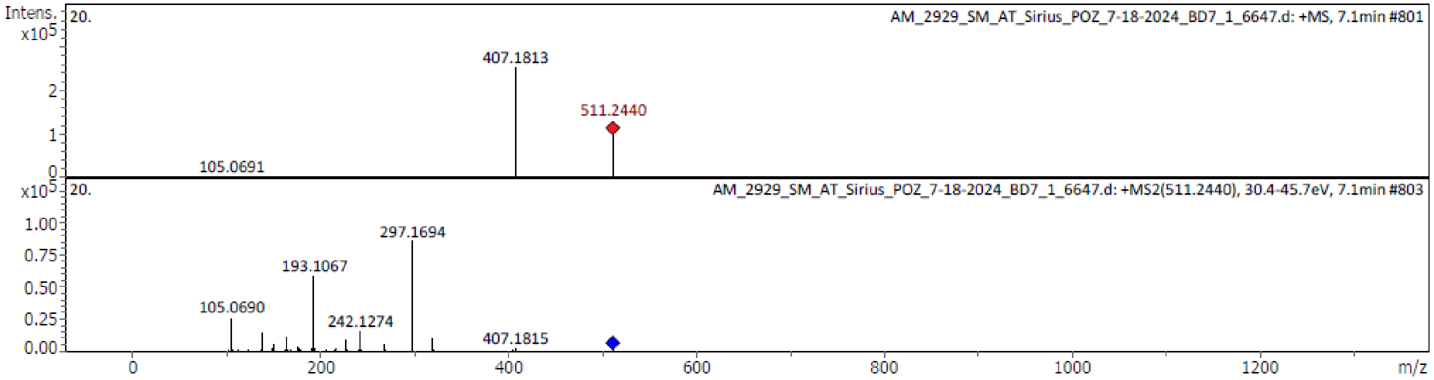
Intens.
x10^5
20.
AM_2929_SM_AT_Sirius_POZ_7-18-2024_BD7_1_6647.d: +MS, 7.1min #801
407.1813
511.2440
105.0691
2
1
0
x10^5
20.
AM_2929_SM_AT_Sirius_POZ_7-18-2024_BD7_1_6647.d: +MS2(511.2440), 30.4-45.7eV, 7.1min #803
297.1694
193.1067
105.0690
242.1274
407.1815
1.00
0.75
0.50
0.25
0.00
0
200
400
600
800
1000
1200
m/z

23m

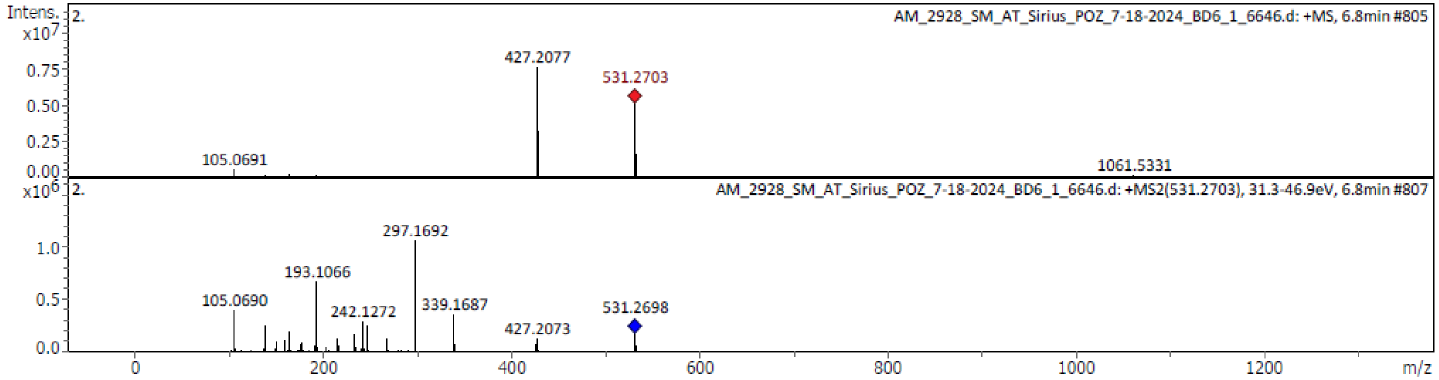
Intens.
x10^7
2.
AM_2928_SM_AT_Sirius_POZ_7-18-2024_BD6_1_6646.d: +MS, 6.8min #805
427.2077
531.2703
105.0691
1061.5331
0.75
0.50
0.25
0.00
x10^6
2.
AM_2928_SM_AT_Sirius_POZ_7-18-2024_BD6_1_6646.d: +MS2(531.2703), 31.3-46.9eV, 6.8min #807
297.1692
193.1066
105.0690
242.1272
339.1687
427.2073
531.2698
1.0
0.5
0.0
0
200
400
600
800
1000
1200
m/z

23n

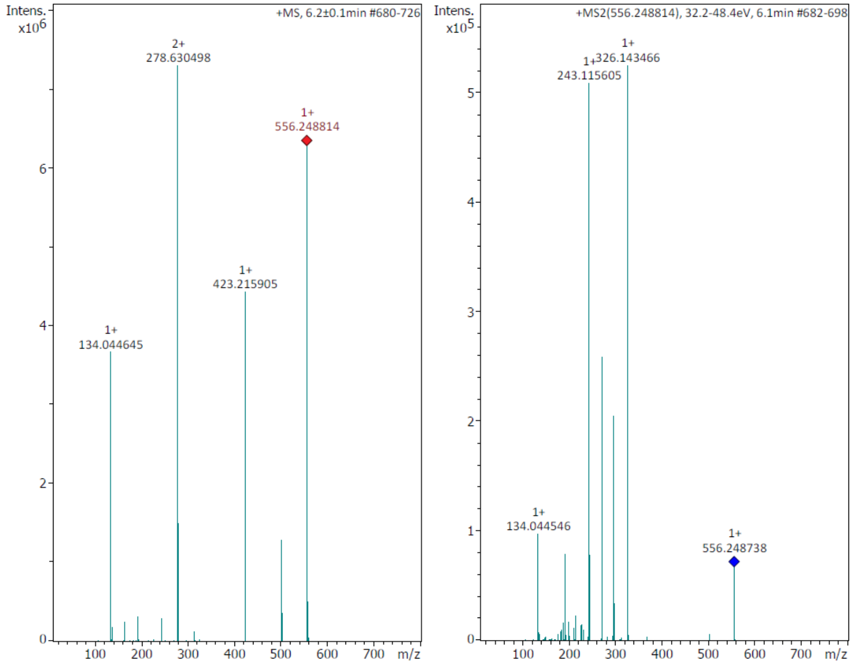

23o

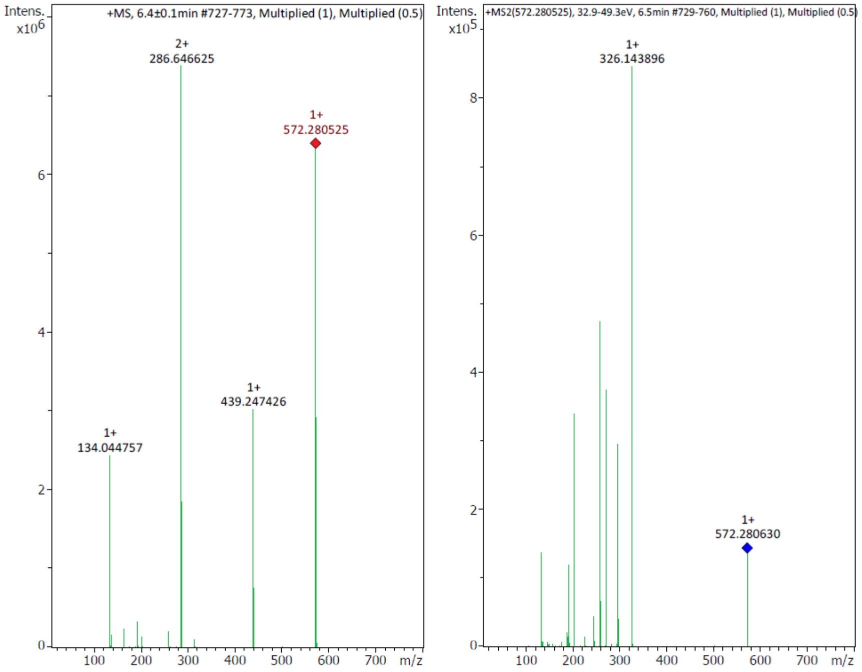

23p

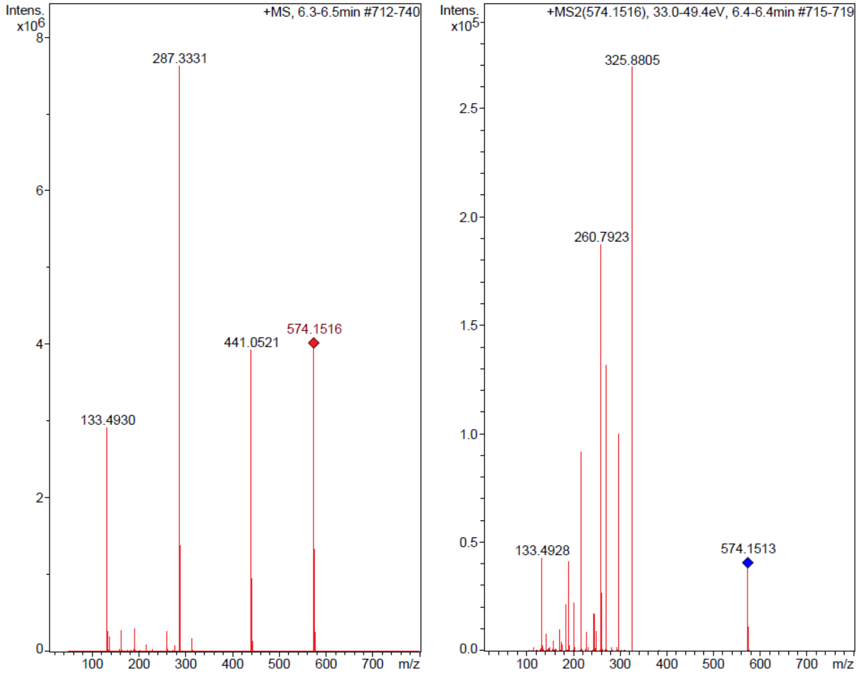

23q

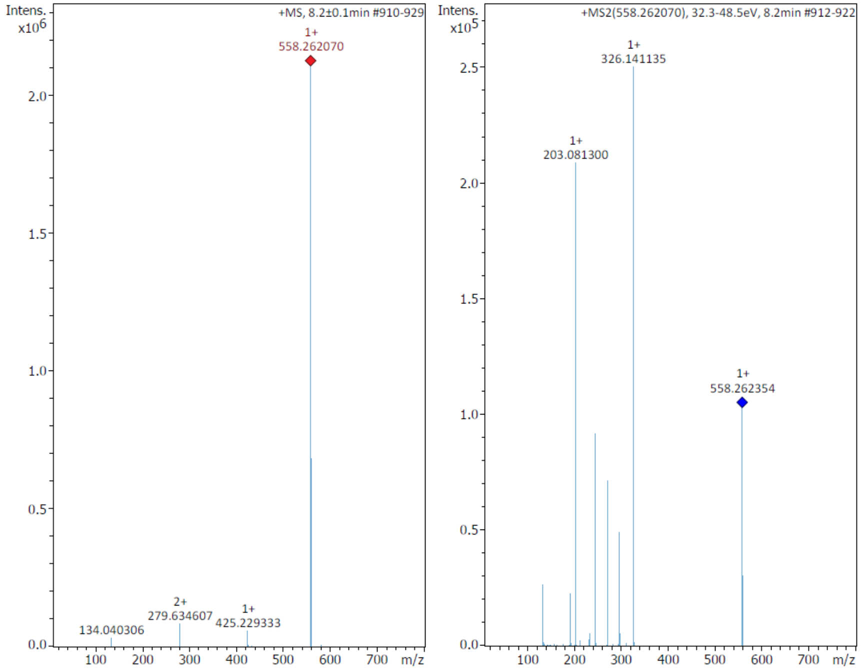

23r

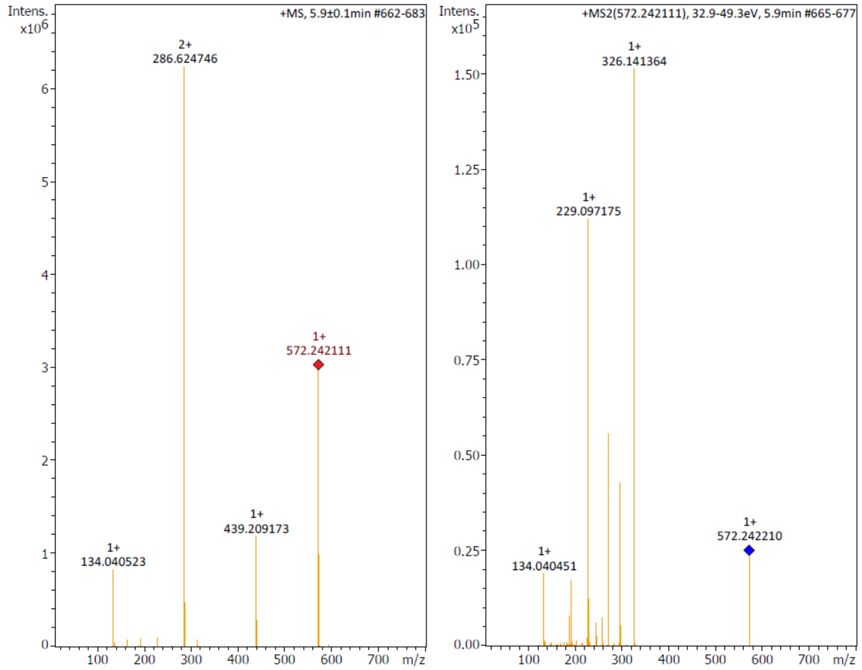

23s

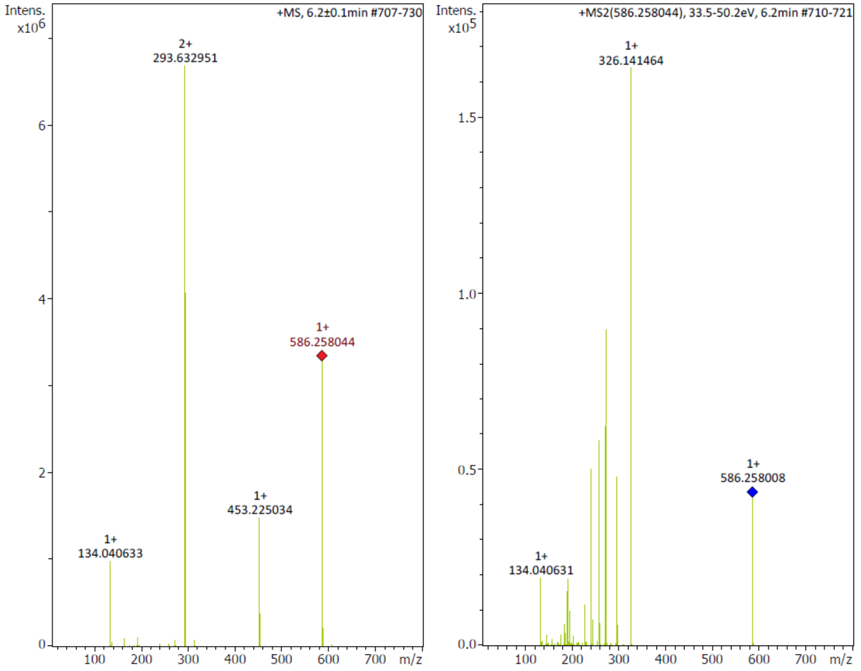

23t

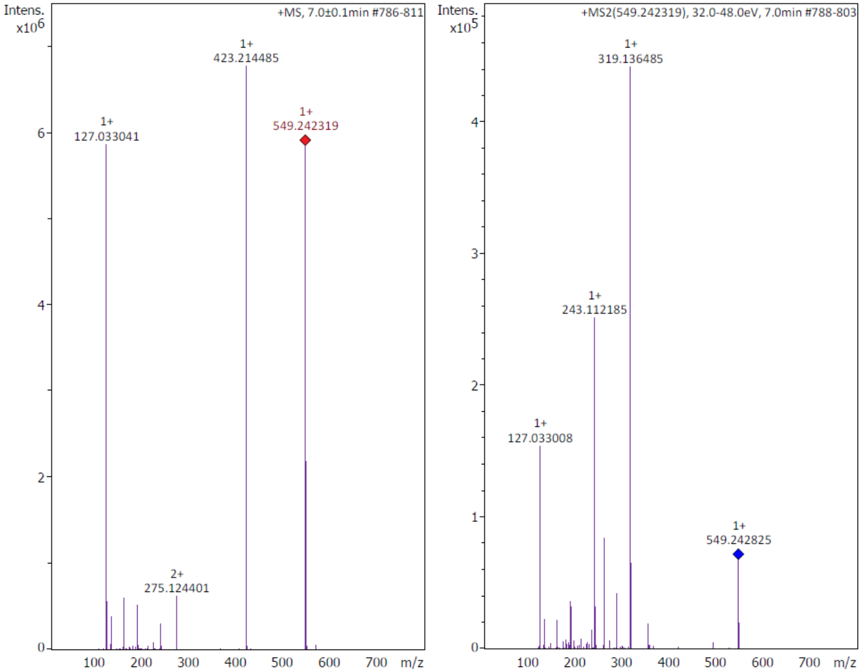

23u

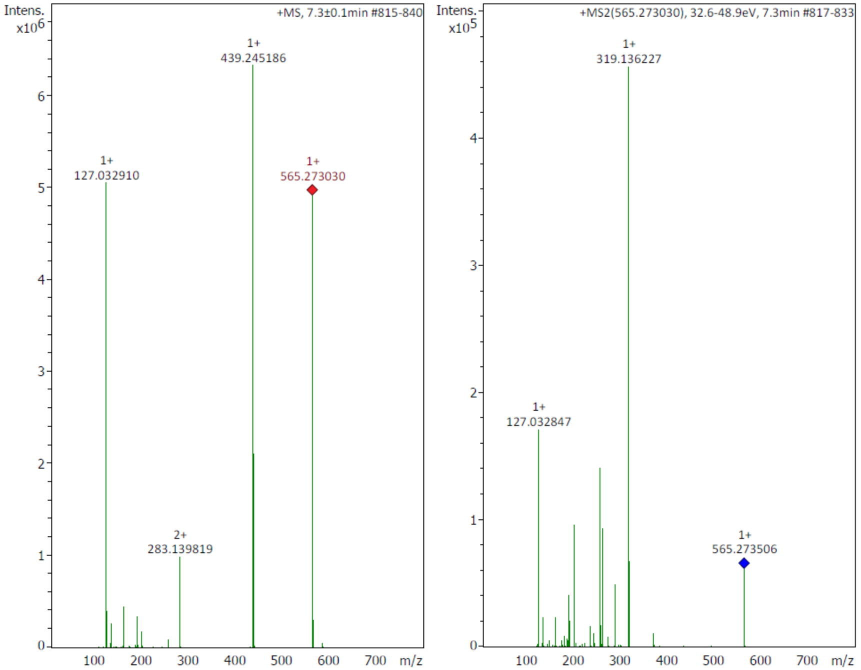

23v

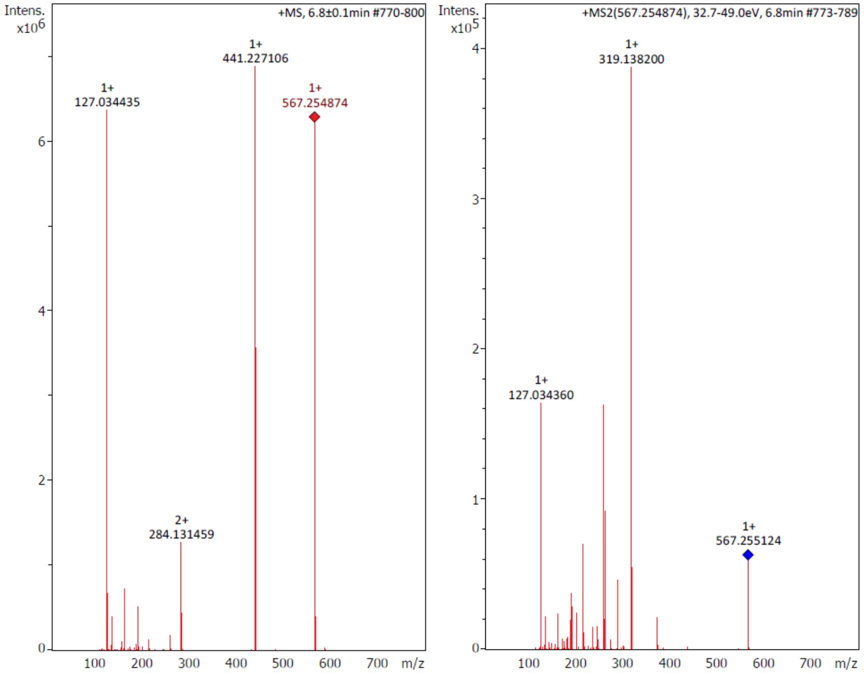

23w

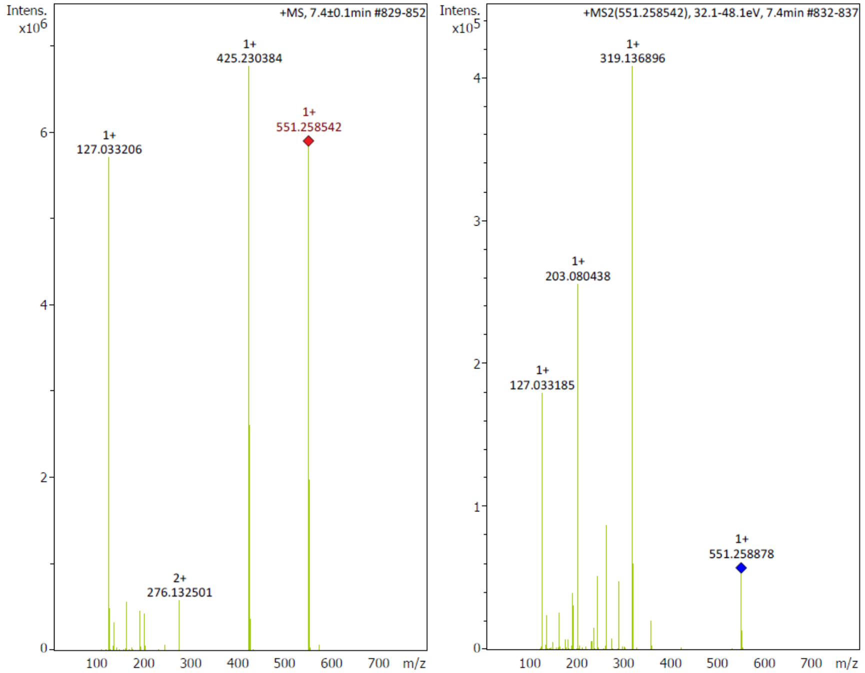

23x

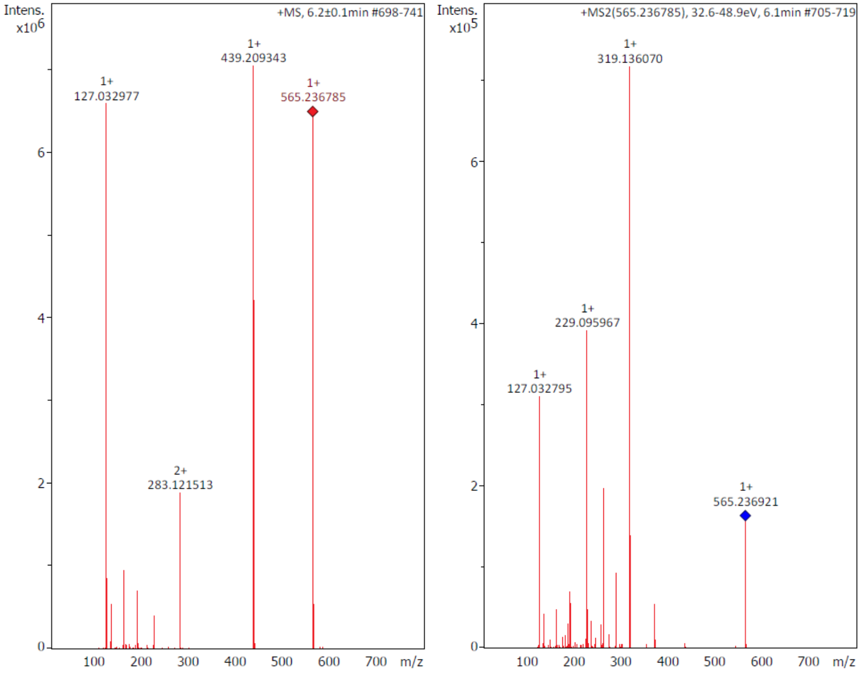

23y

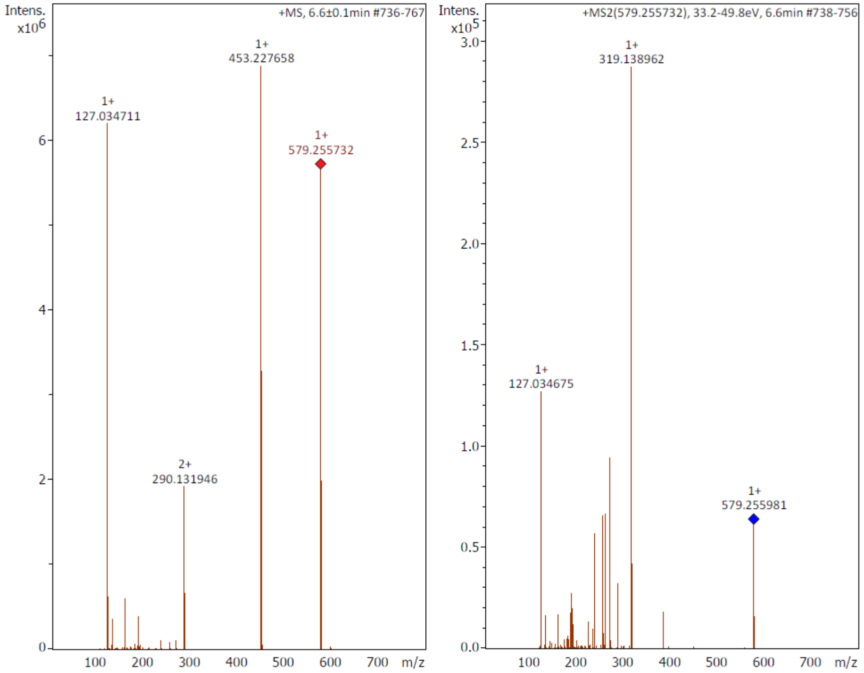

23z

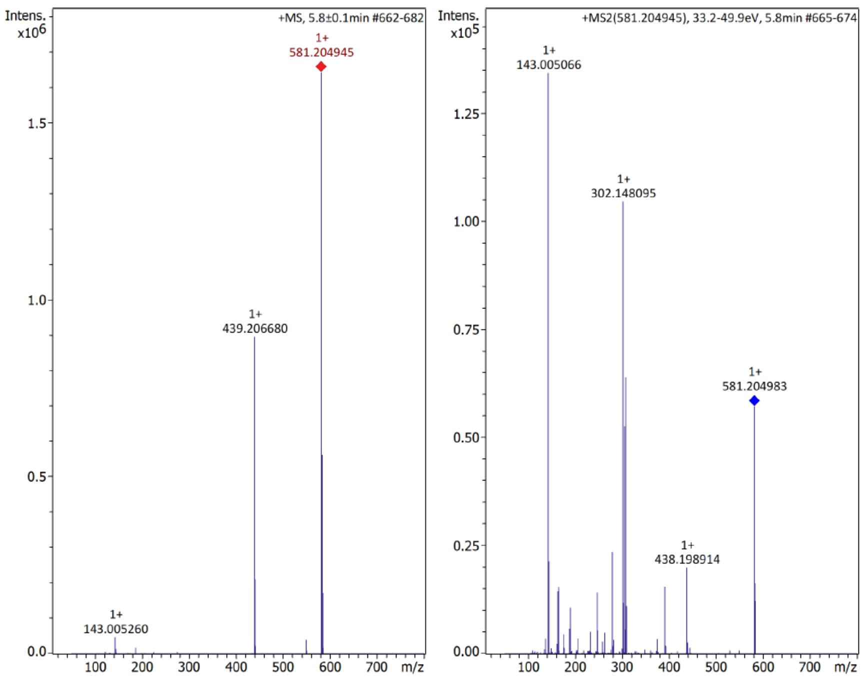

23aa

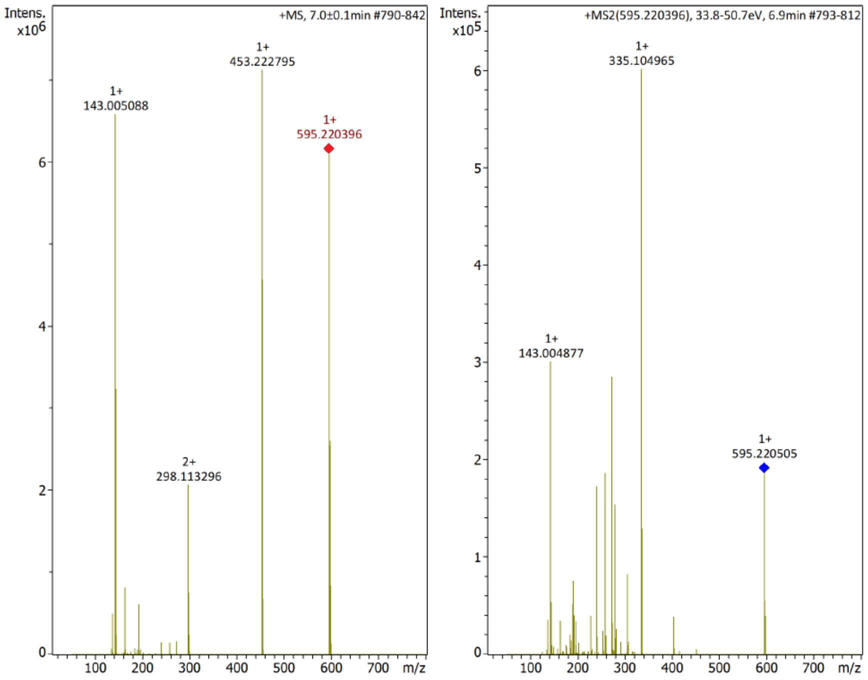

23r'

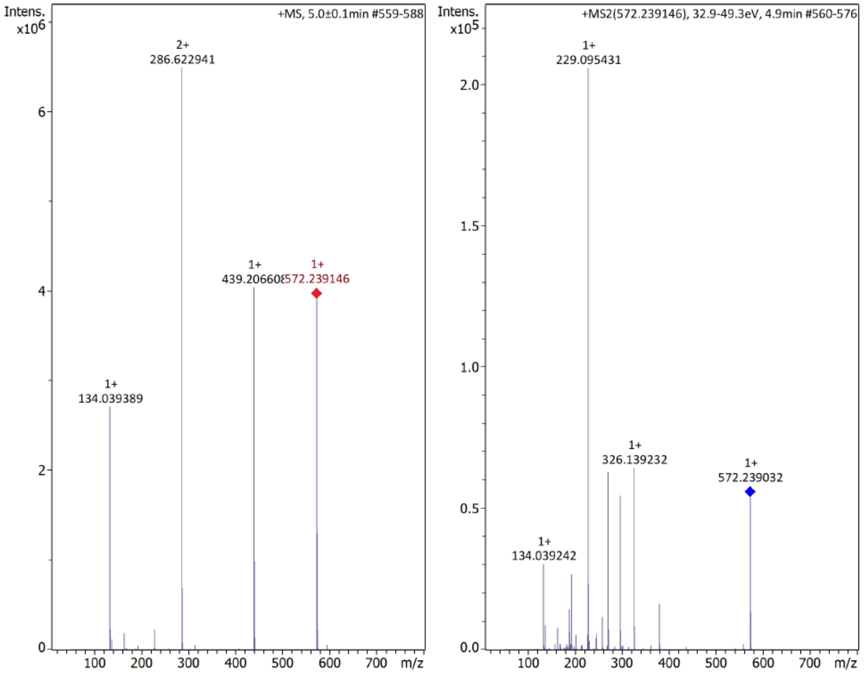

23s'

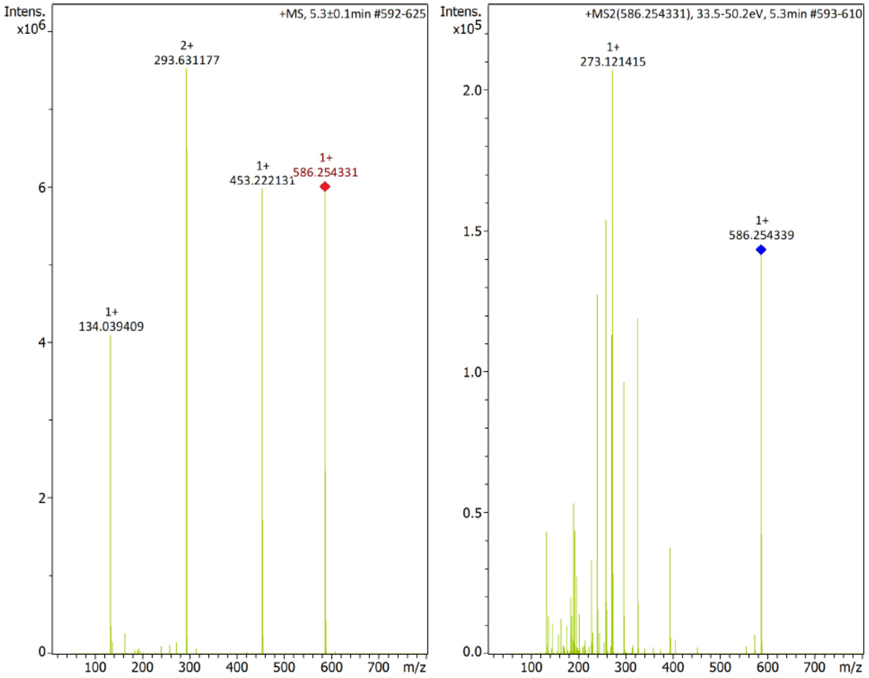

23z'

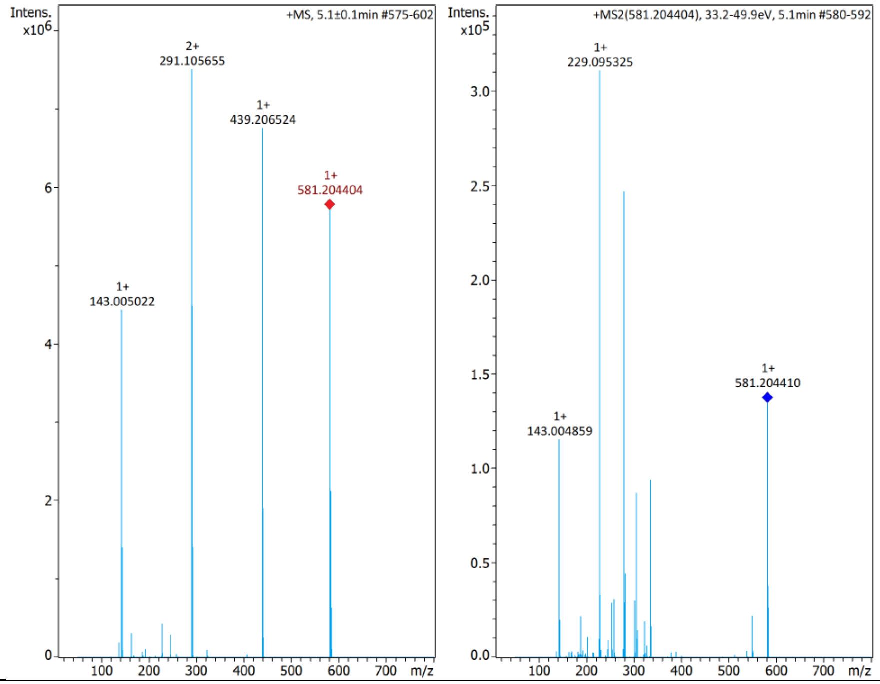

23aa'

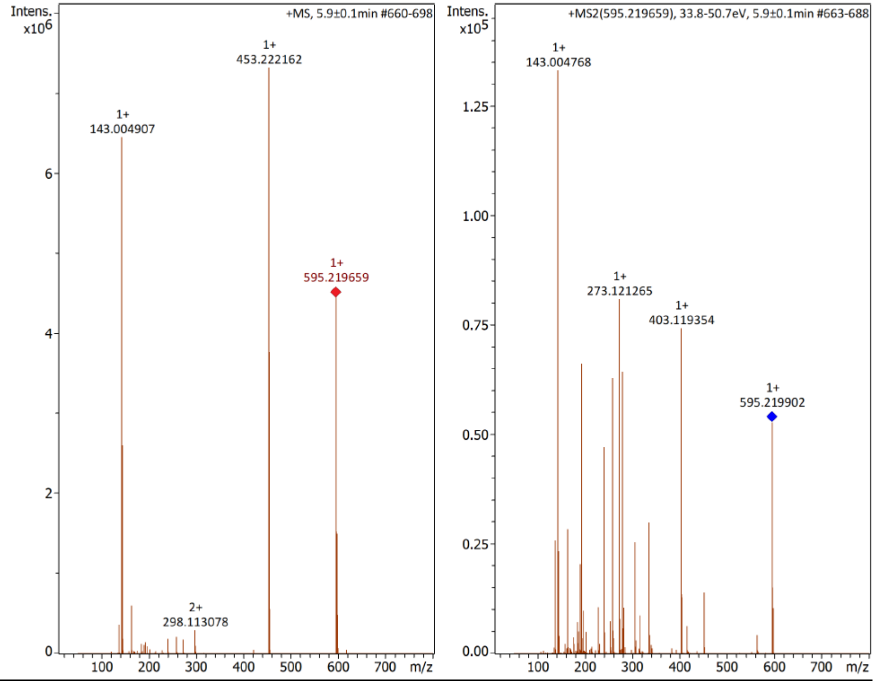

**Compound**	HRMS spectra (MS1, MS2)
**12a**	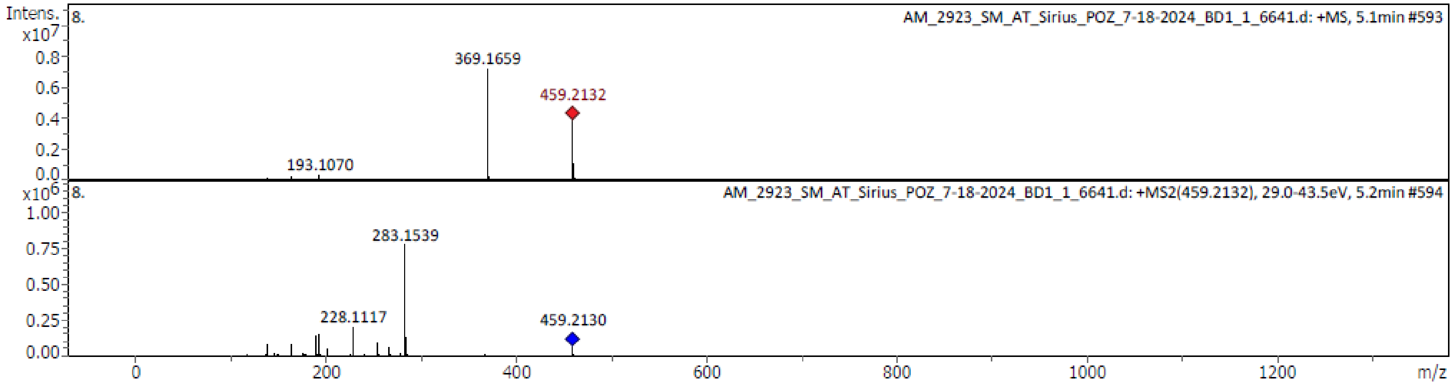

12b

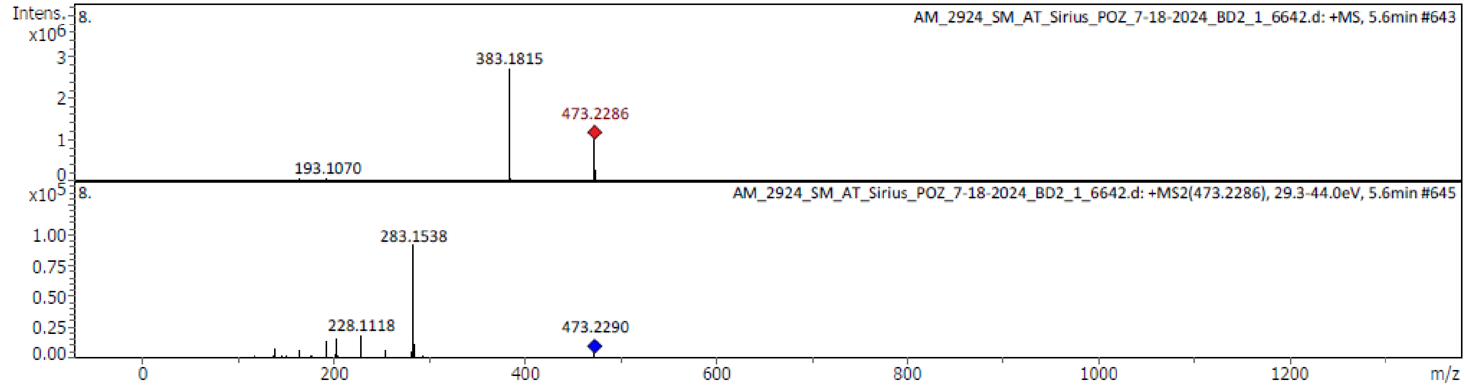

Intens. x10^6
AM_2924_SM_AT_Sirius_POZ_7-18-2024_BD2_1_6642.d: +MS, 5.6min #643
383.1815
473.2286
193.1070
x10^5
AM_2924_SM_AT_Sirius_POZ_7-18-2024_BD2_1_6642.d: +MS2(473.2286), 29.3-44.0eV, 5.6min #645
283.1538
228.1118
473.2290
m/z

12c

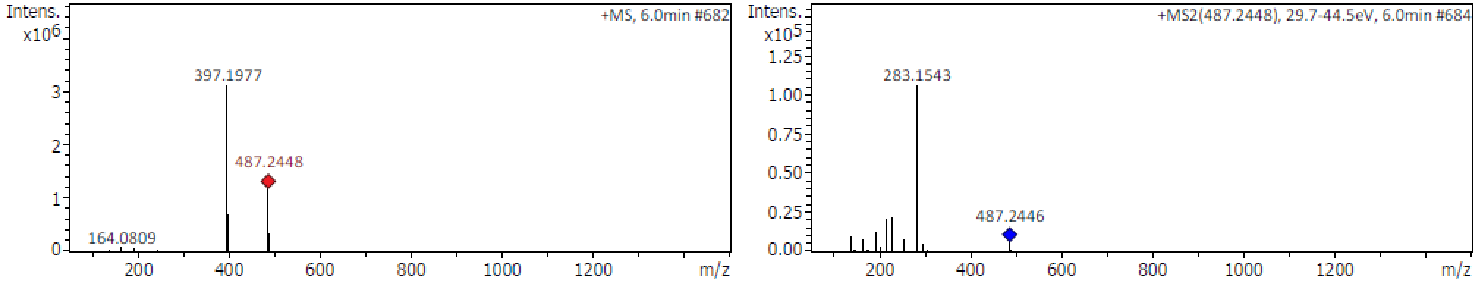

Intens. x10^6
+MS, 6.0min #682
397.1977
487.2448
164.0809
m/z
Intens. x10^5
+MS2(487.2448), 29.7-44.5eV, 6.0min #684
283.1543
487.2446
m/z

12d

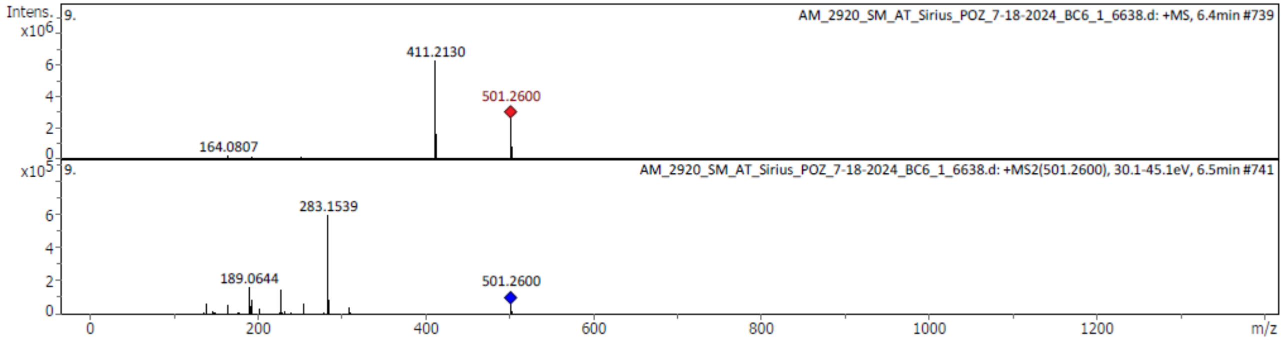

Intens. x10^6
AM_2920_SM_AT_Sirius_POZ_7-18-2024_BC6_1_6638.d: +MS, 6.4min #739
411.2130
501.2600
164.0807
x10^5
AM_2920_SM_AT_Sirius_POZ_7-18-2024_BC6_1_6638.d: +MS2(501.2600), 30.1-45.1eV, 6.5min #741
283.1539
189.0644
501.2600
m/z

12e

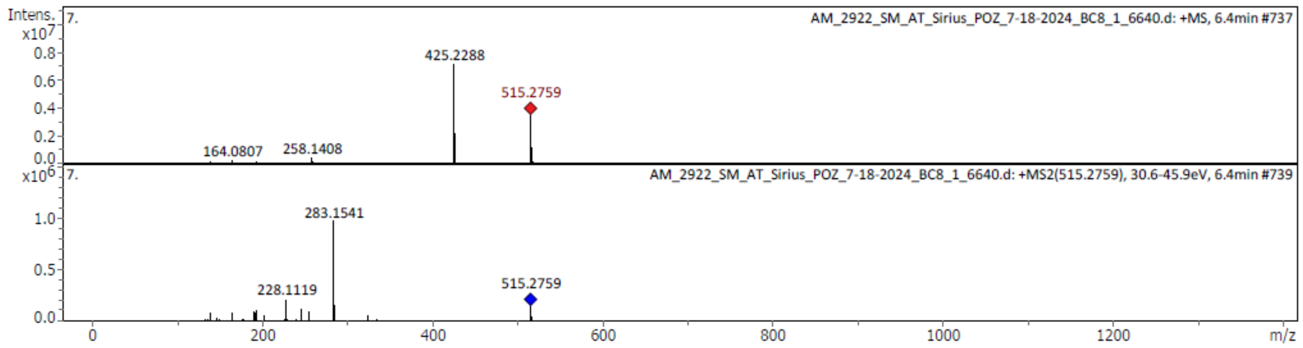
Intens.
x10^7
AM_2922_SM_AT_Sirius_POZ_7-18-2024_BC8_1_6640.d: +MS, 6.4min #737
425.2288
515.2759
164.0807
258.1408
x10^6
AM_2922_SM_AT_Sirius_POZ_7-18-2024_BC8_1_6640.d: +MS2(515.2759), 30.6-45.9eV, 6.4min #739
283.1541
228.1119
515.2759
m/z

12f

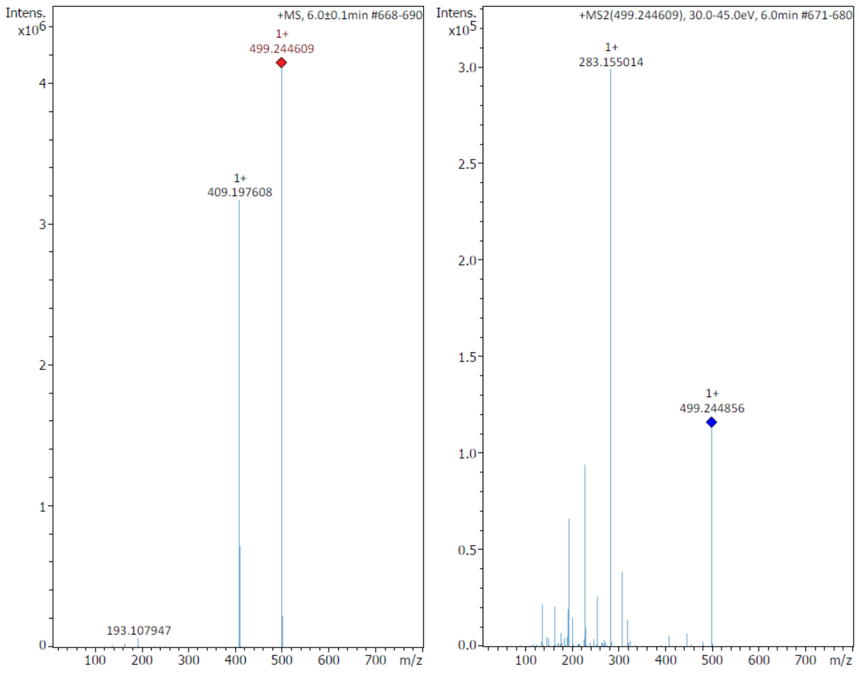
Intens.
x10^6
+MS, 6.0±0.1min #668-690
1+
499.244609
1+
409.197608
193.107947
m/z
Intens.
x10^5
+MS2(499.244609), 30.0-45.0eV, 6.0min #671-680
1+
283.155014
1+
499.244856
m/z

12g

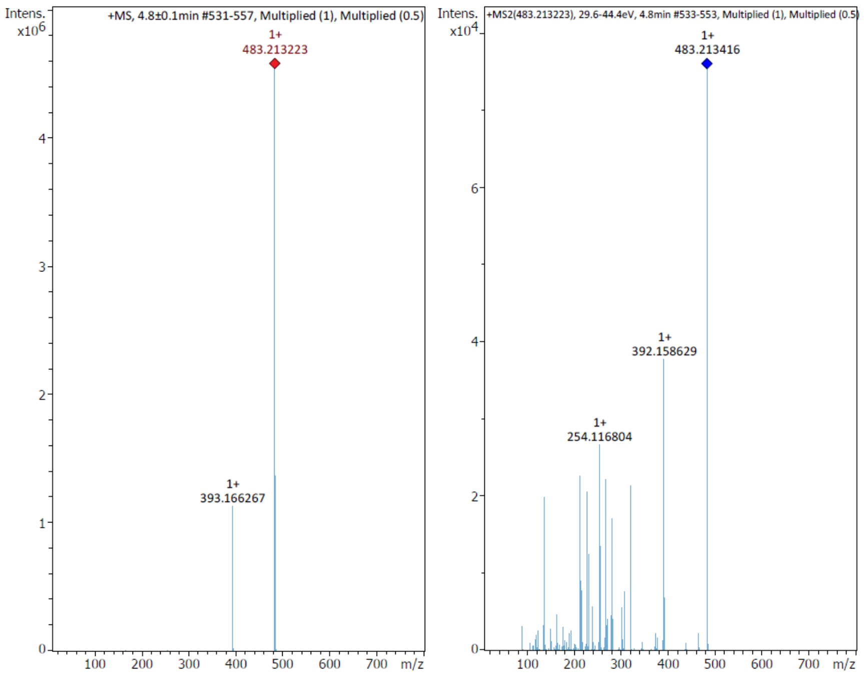
Intens.
x10^6
+MS, 4.8±0.1min #531-557, Multiplied (1), Multiplied (0.5)
1+
483.213223
1+
393.166267
m/z
Intens.
x10^4
+MS2(483.213223), 29.6-44.4eV, 4.8min #533-553, Multiplied (1), Multiplied (0.5)
1+
483.213416
1+
392.158629
1+
254.116804
m/z

12h

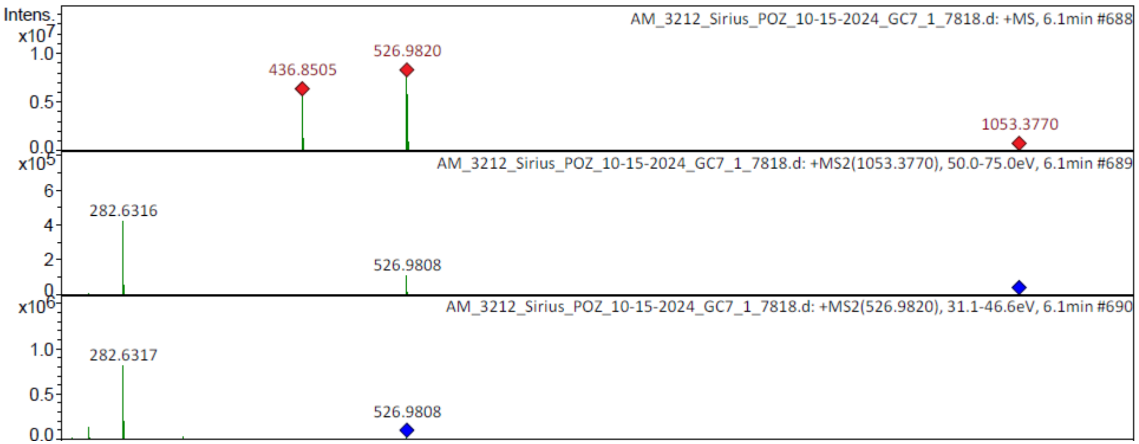

Intens. x10^7
AM_3212_Sirius_POZ_10-15-2024_GC7_1_7818.d: +MS, 6.1min #688
436.8505
526.9820
1053.3770
x10^5
AM_3212_Sirius_POZ_10-15-2024_GC7_1_7818.d: +MS2(1053.3770), 50.0-75.0eV, 6.1min #689
282.6316
526.9808
x10^6
AM_3212_Sirius_POZ_10-15-2024_GC7_1_7818.d: +MS2(526.9820), 31.1-46.6eV, 6.1min #690
282.6317
526.9808

12i

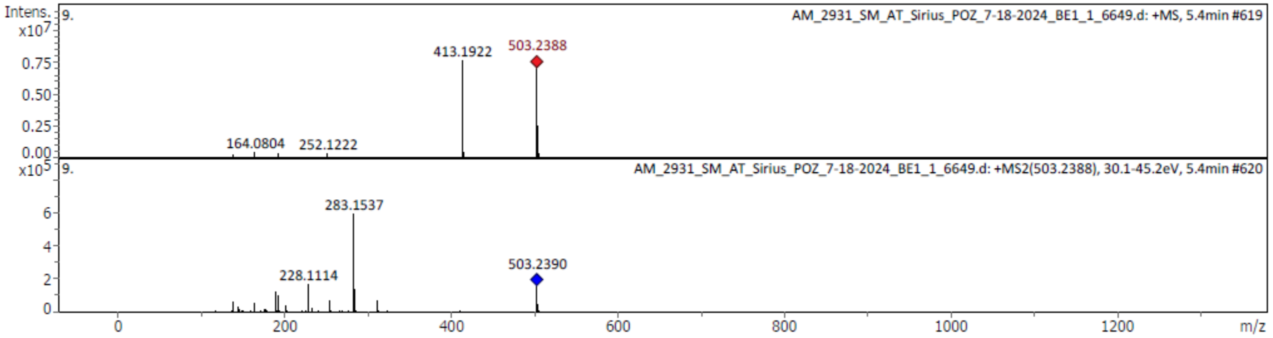

Intens. x10^7
AM_2931_SM_AT_Sirius_POZ_7-18-2024_BE1_1_6649.d: +MS, 5.4min #619
413.1922
503.2388
164.0804
252.1222
x10^5
AM_2931_SM_AT_Sirius_POZ_7-18-2024_BE1_1_6649.d: +MS2(503.2388), 30.1-45.2eV, 5.4min #620
283.1537
228.1114
503.2390
m/z

12j

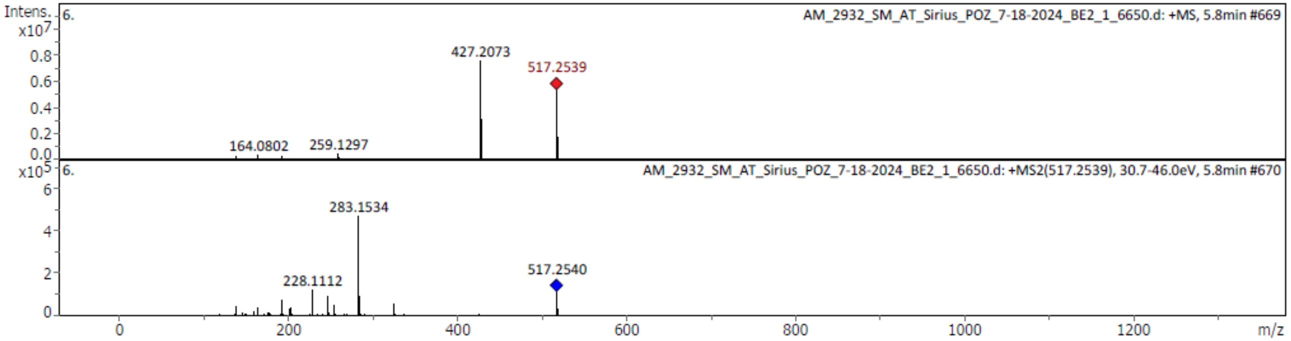

Intens. x10^7
AM_2932_SM_AT_Sirius_POZ_7-18-2024_BE2_1_6650.d: +MS, 5.8min #669
427.2073
517.2539
164.0802
259.1297
x10^5
AM_2932_SM_AT_Sirius_POZ_7-18-2024_BE2_1_6650.d: +MS2(517.2539), 30.7-46.0eV, 5.8min #670
283.1534
228.1112
517.2540
m/z

12k

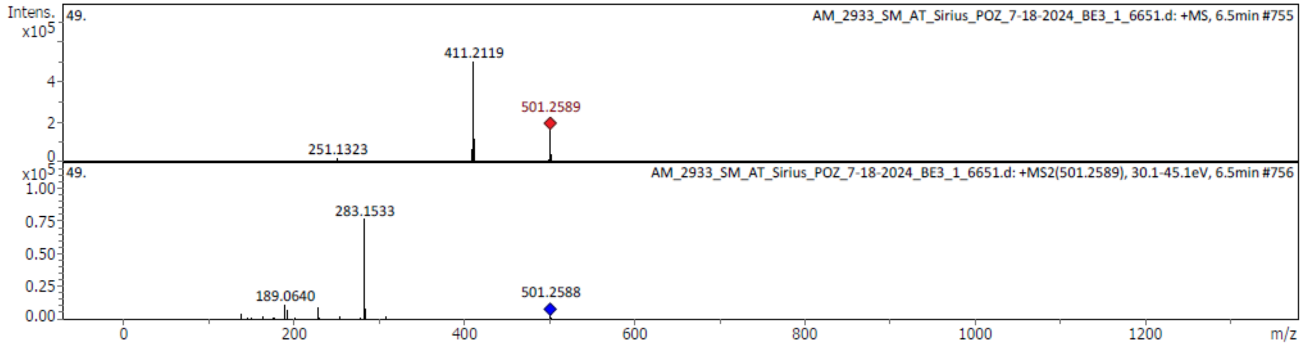

Intens.
x10^5
49.
AM_2933_SM_AT_Sirius_POZ_7-18-2024_BE3_1_6651.d: +MS, 6.5min #755
411.2119
501.2589
251.1323
49.
AM_2933_SM_AT_Sirius_POZ_7-18-2024_BE3_1_6651.d: +MS2(501.2589), 30.1-45.1eV, 6.5min #756
283.1533
189.0640
501.2588
m/z

12l

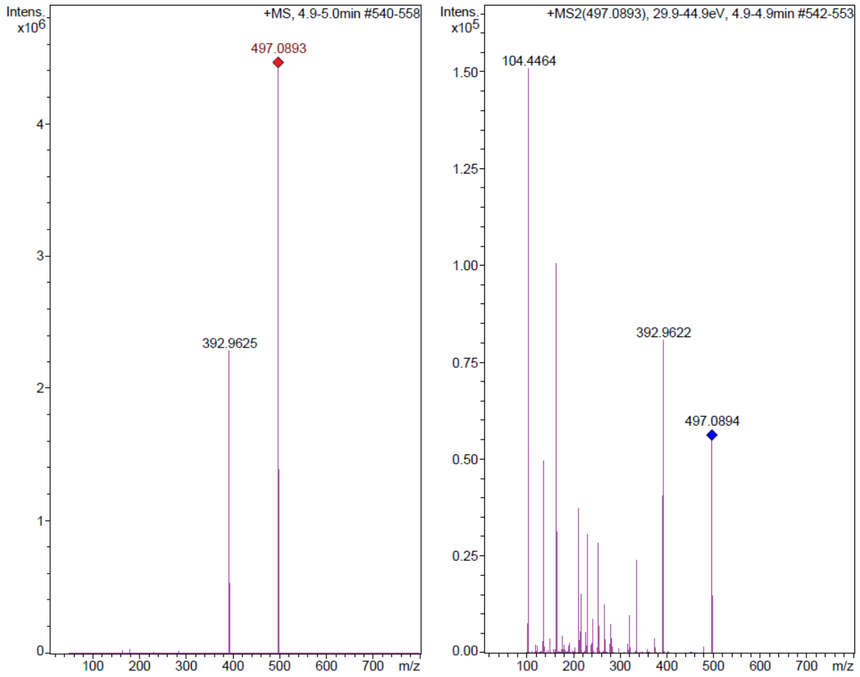

Intens.
x10^6
+MS, 4.9-5.0min #540-558
497.0893
392.9625
m/z
Intens.
x10^5
+MS2(497.0893), 29.9-44.9eV, 4.9-4.9min #542-553
104.4464
392.9622
497.0894
m/z

12m

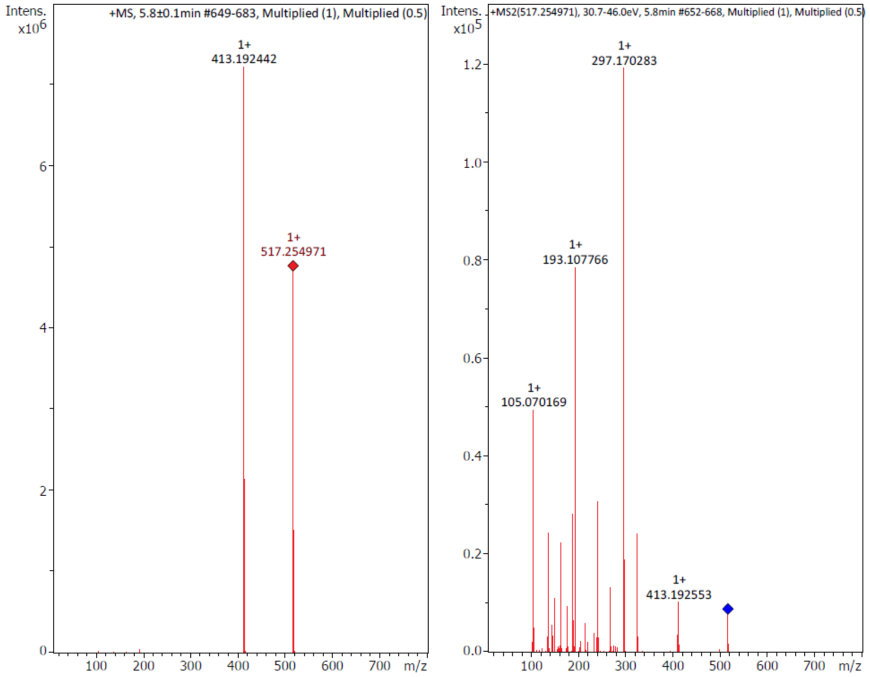

Intens.
x10^6
+MS, 5.8±0.1min #649-683, Multiplied (1), Multiplied (0.5)
1+
413.192442
1+
517.254971
m/z
Intens.
x10^5
+MS2(517.254971), 30.7-46.0eV, 5.8min #652-668, Multiplied (1), Multiplied (0.5)
1+
297.170283
1+
193.107766
1+
105.070169
1+
413.192553
m/z

12n

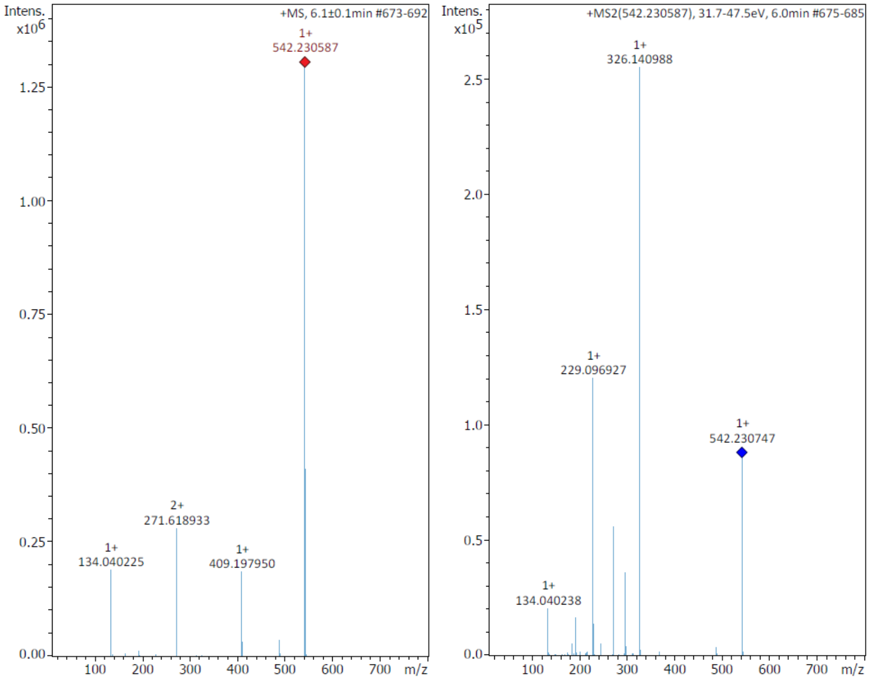

12o

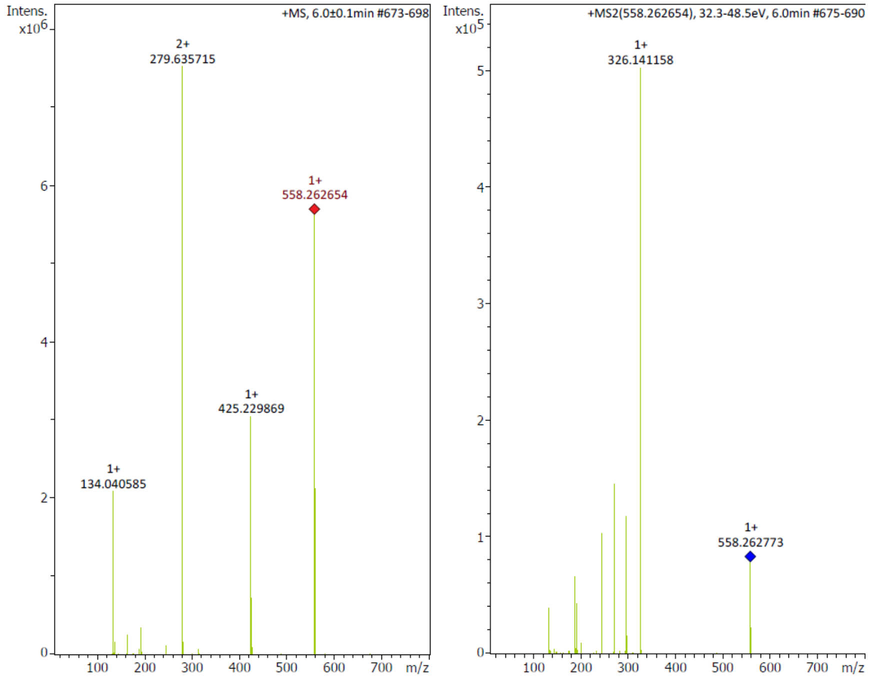

12p

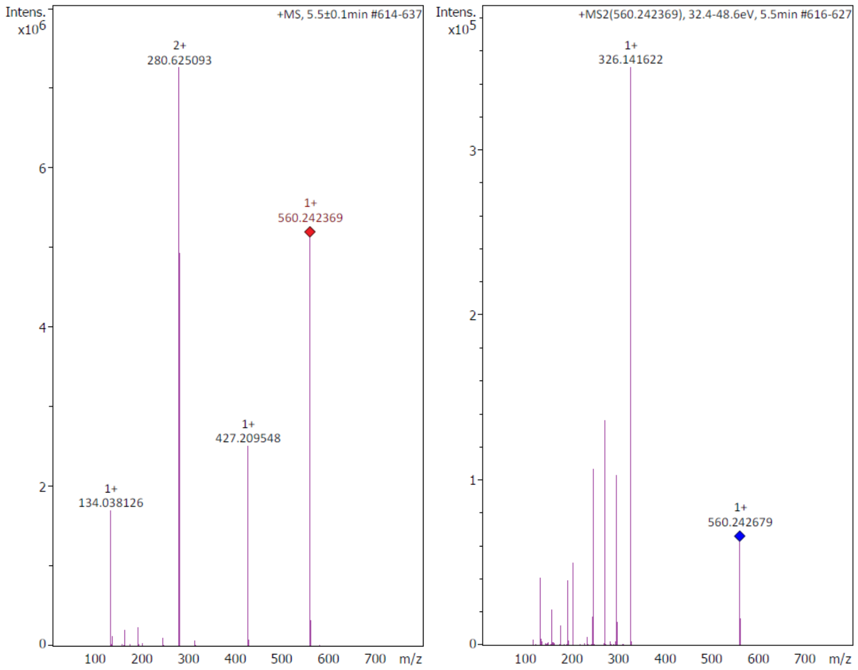

12q

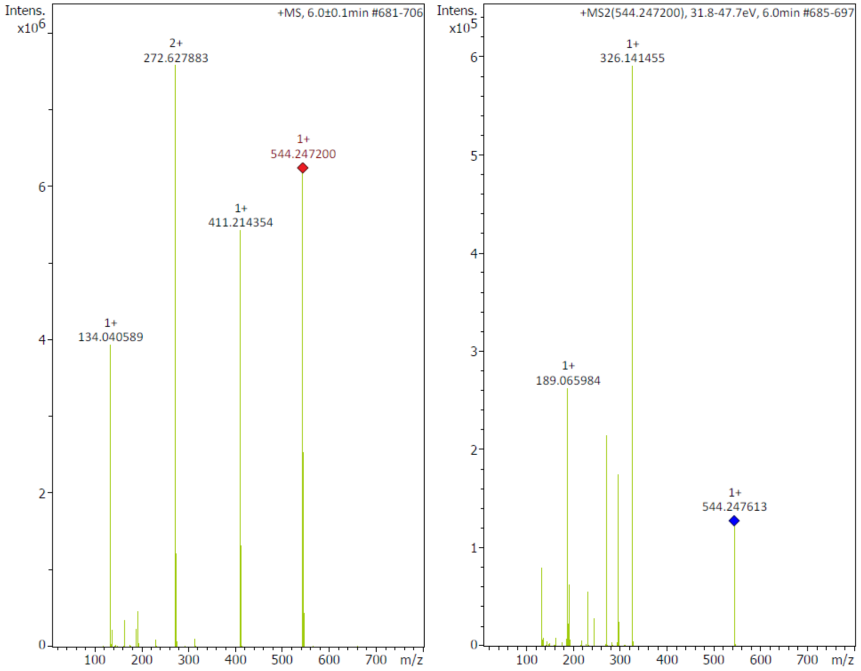

12r

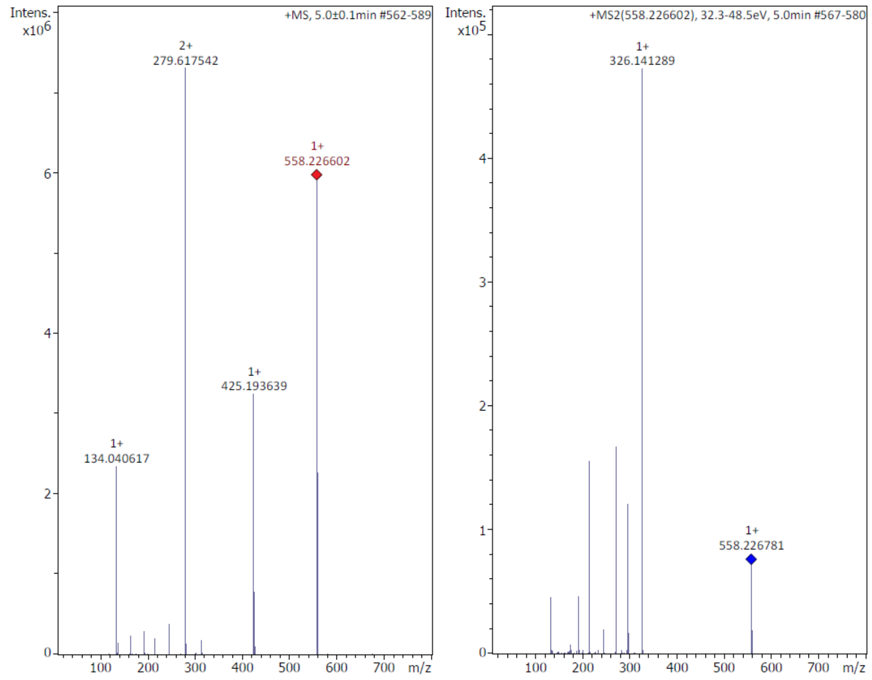

12s

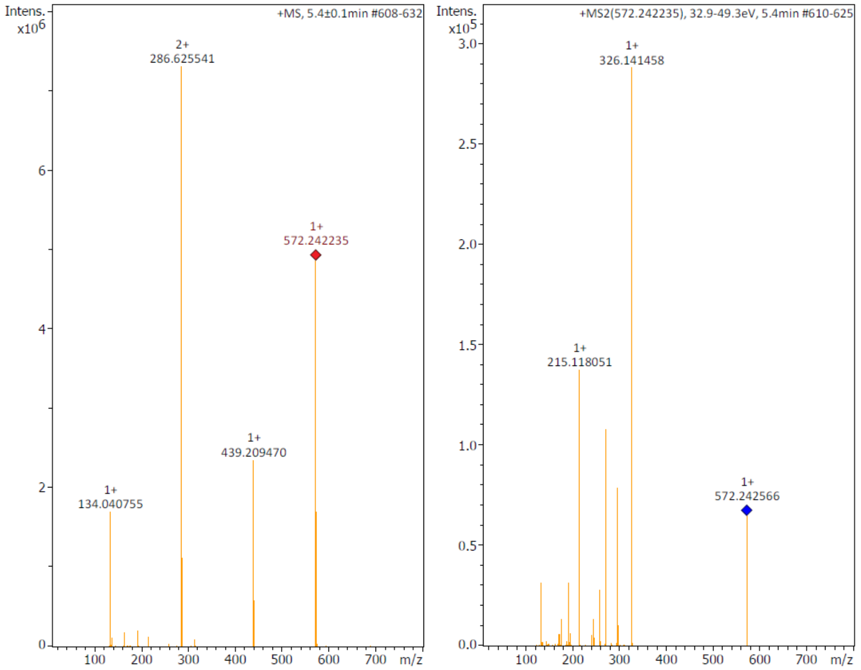

12t

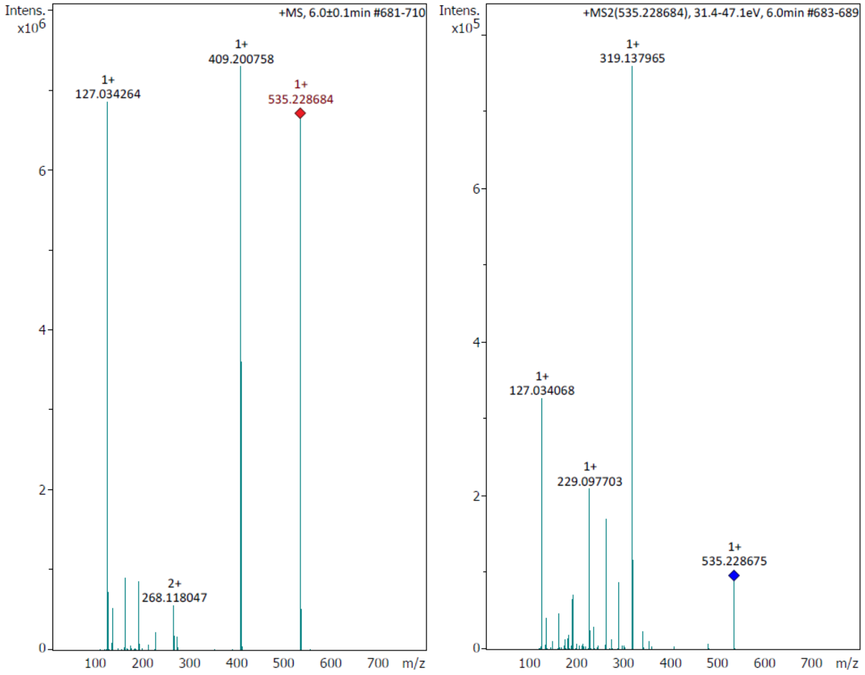

12u

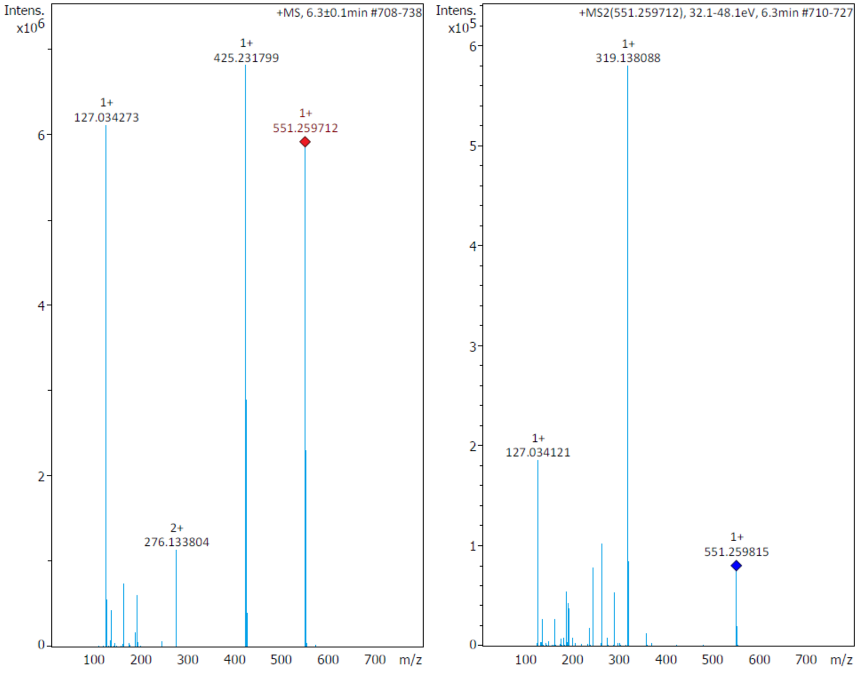

12v

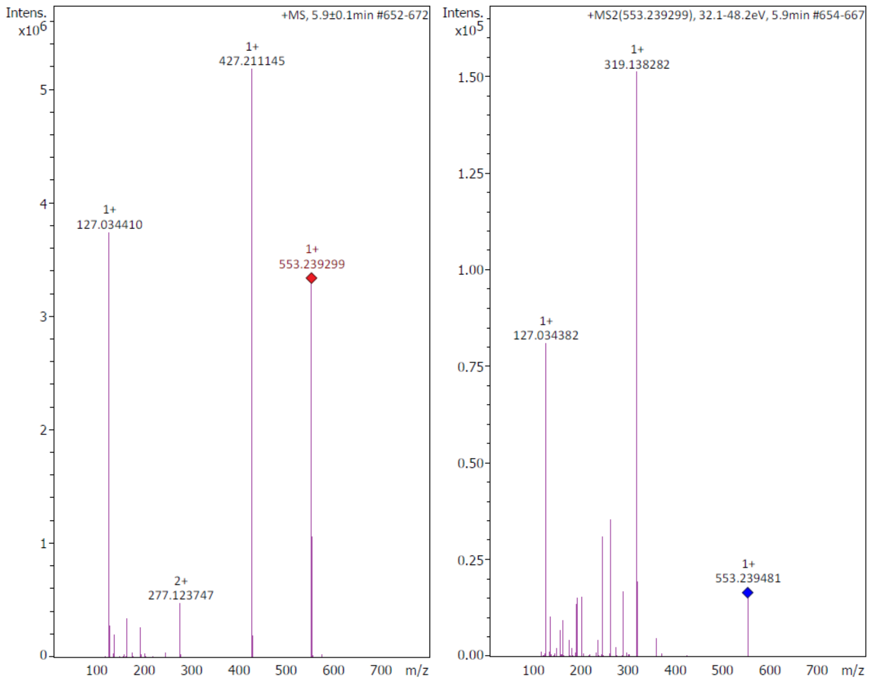

12w

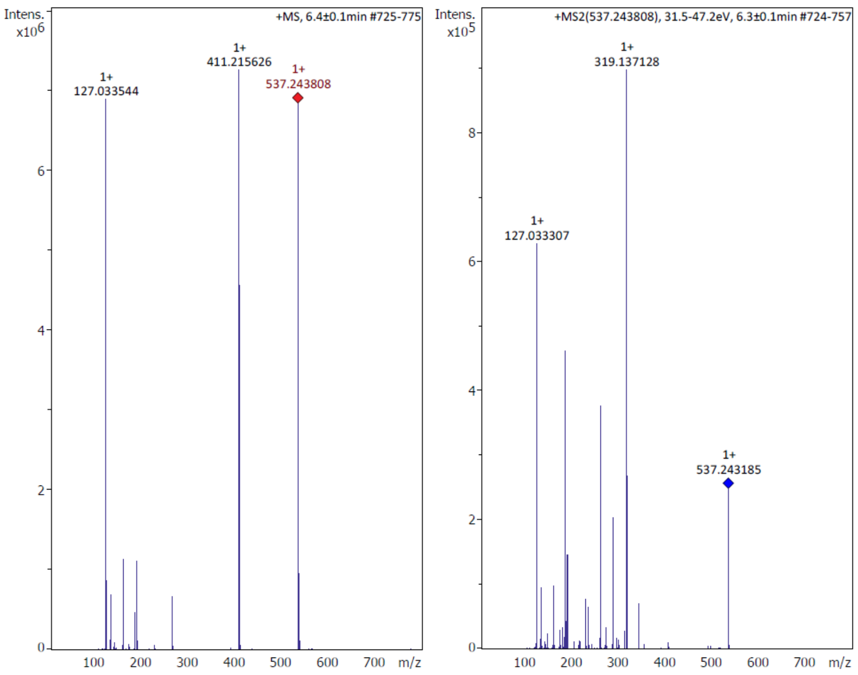

12x

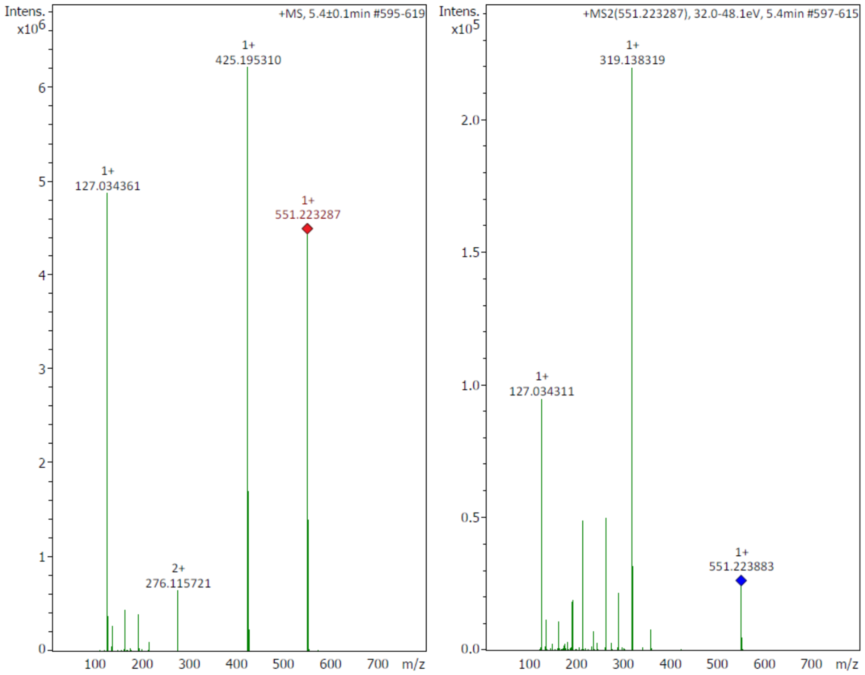

12y

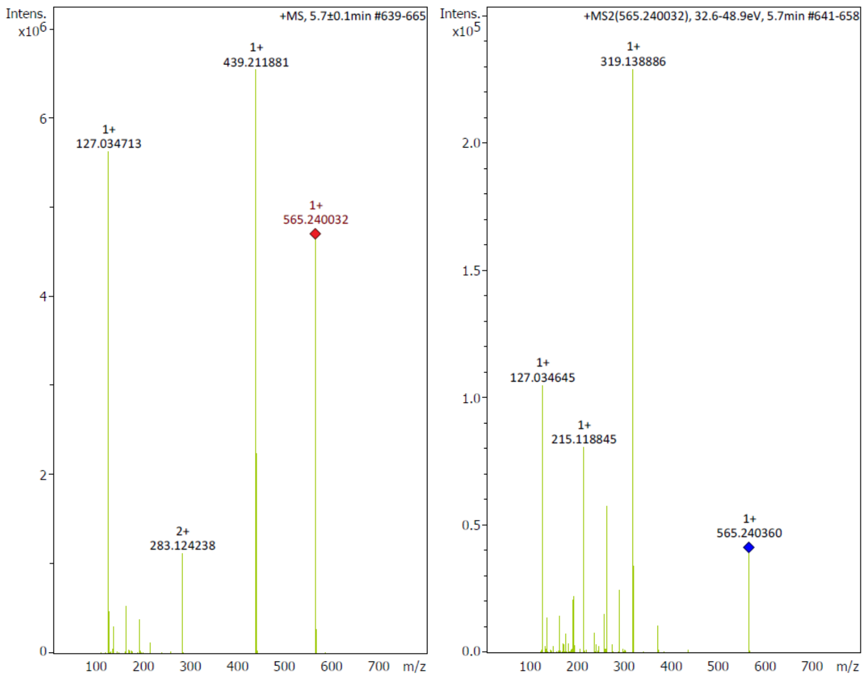

12z

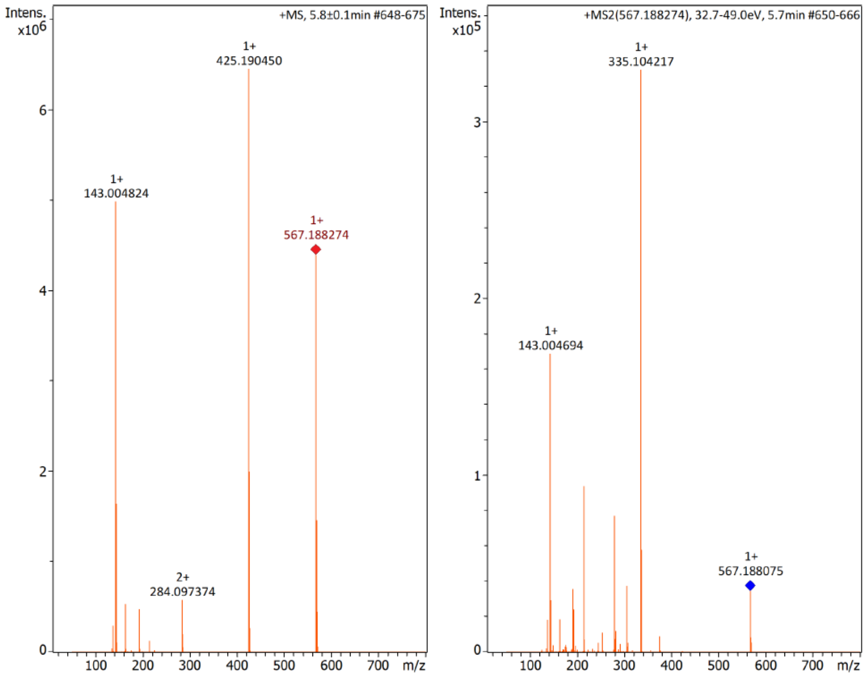

12aa

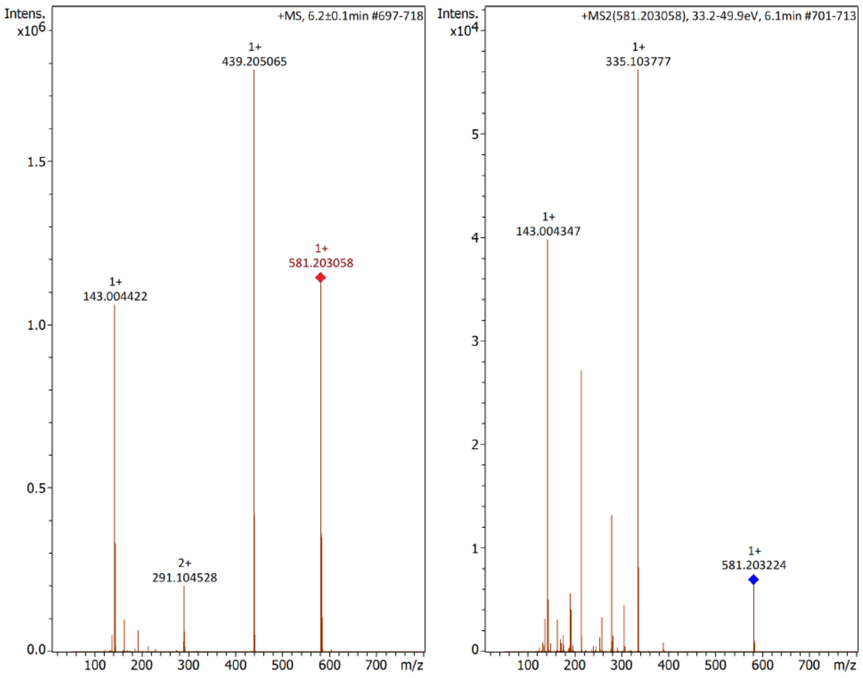

12r'

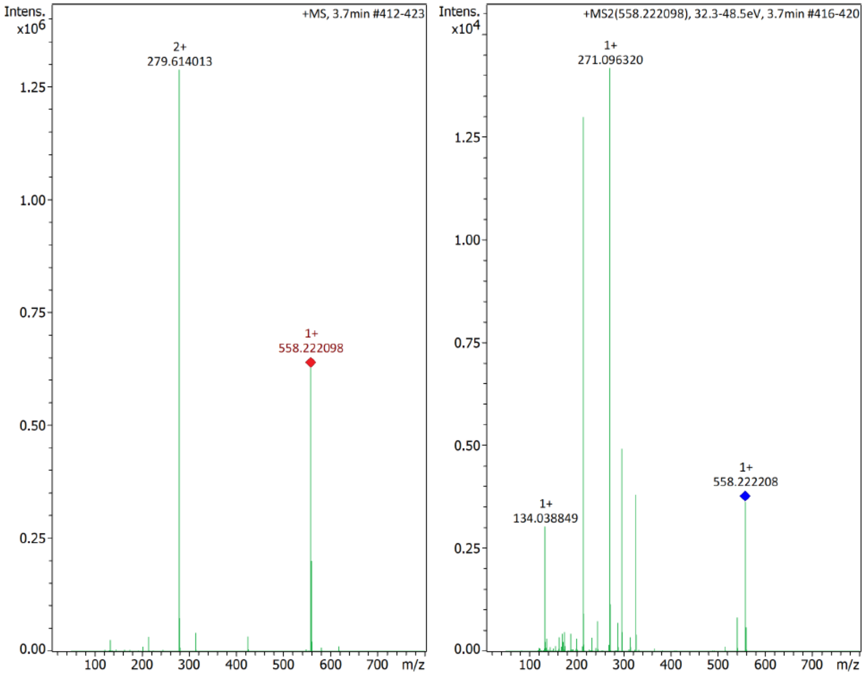

12s'

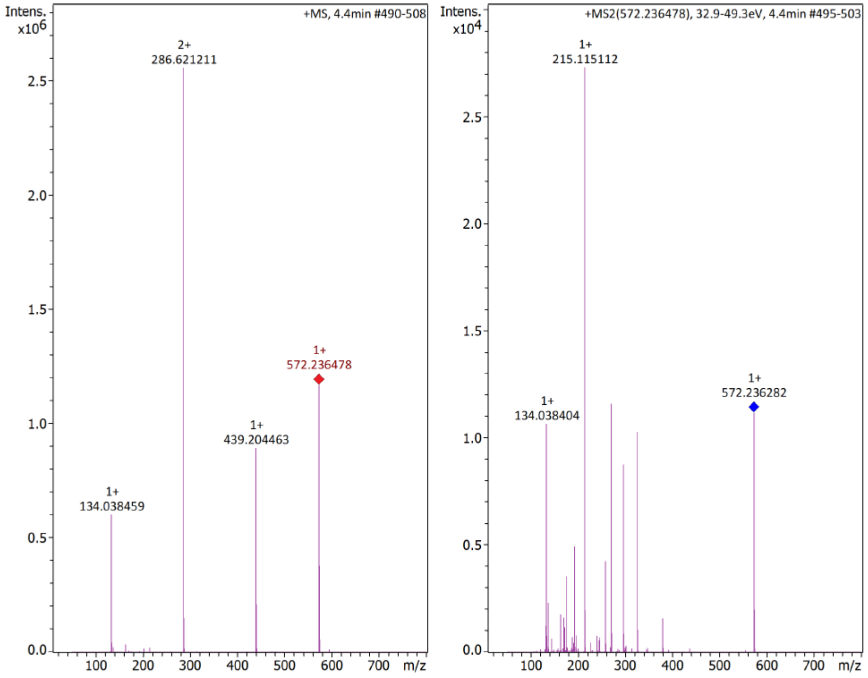

12z'

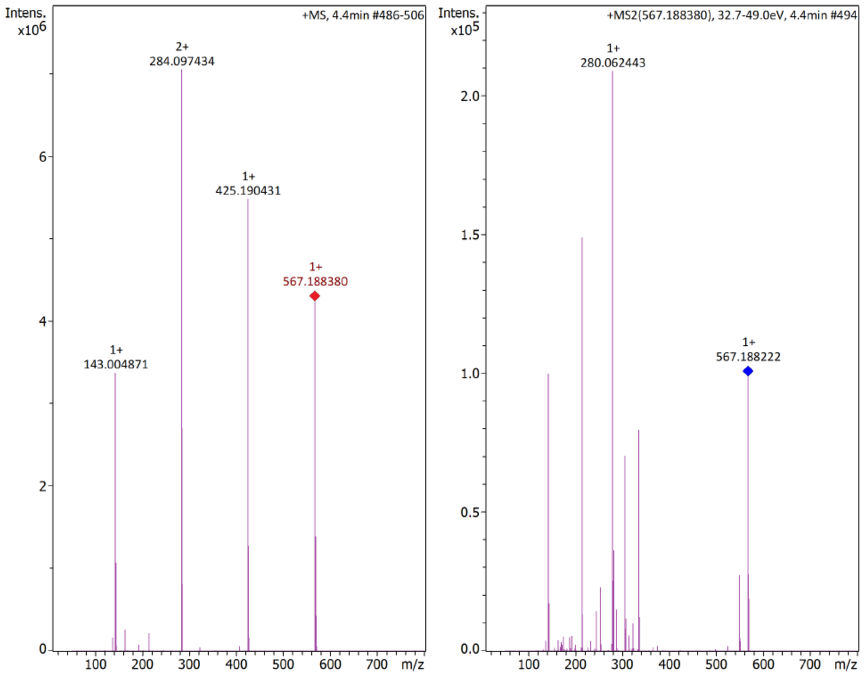

Intens. x10^6
+MS, 4.4min #486-506
2+ 284.097434
1+ 425.190431
1+ 567.188380
1+ 143.004871
Intens. x10^5
+MS2(567.188380), 32.7-49.0eV, 4.4min #494
1+ 280.062443
1+ 567.188222
m/z

12aa'

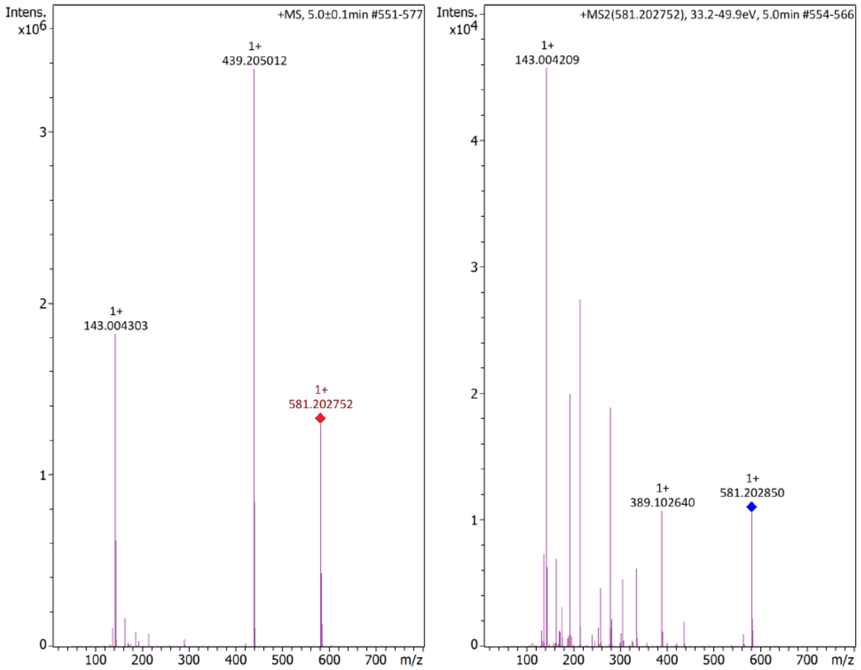

Intens. x10^6
+MS, 5.0±0.1min #551-577
1+ 439.205012
1+ 143.004303
1+ 581.202752
Intens. x10^4
+MS2(581.202752), 33.2-49.9eV, 5.0min #554-566
1+ 143.004209
1+ 389.102640
1+ 581.202850
m/z

Compound	HRMS spectra (MS1, MS2)
**13a**	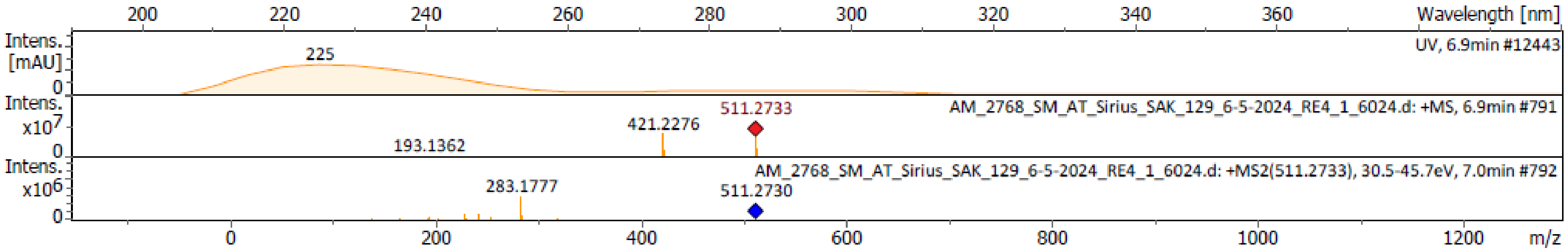
**13b**	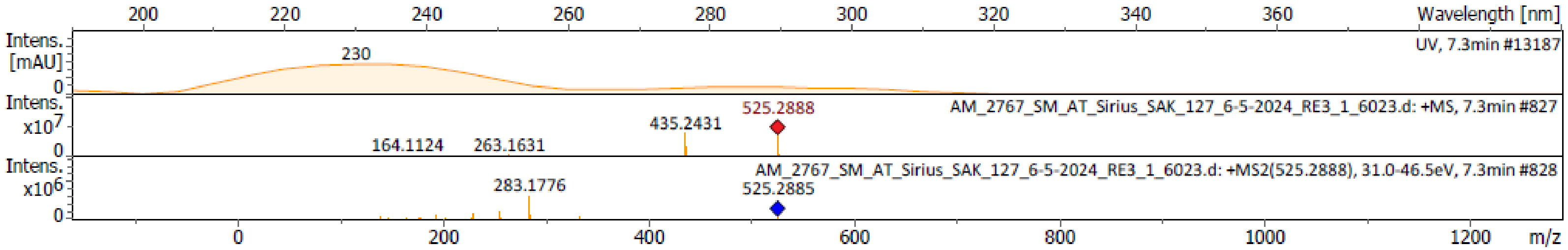
**13c**	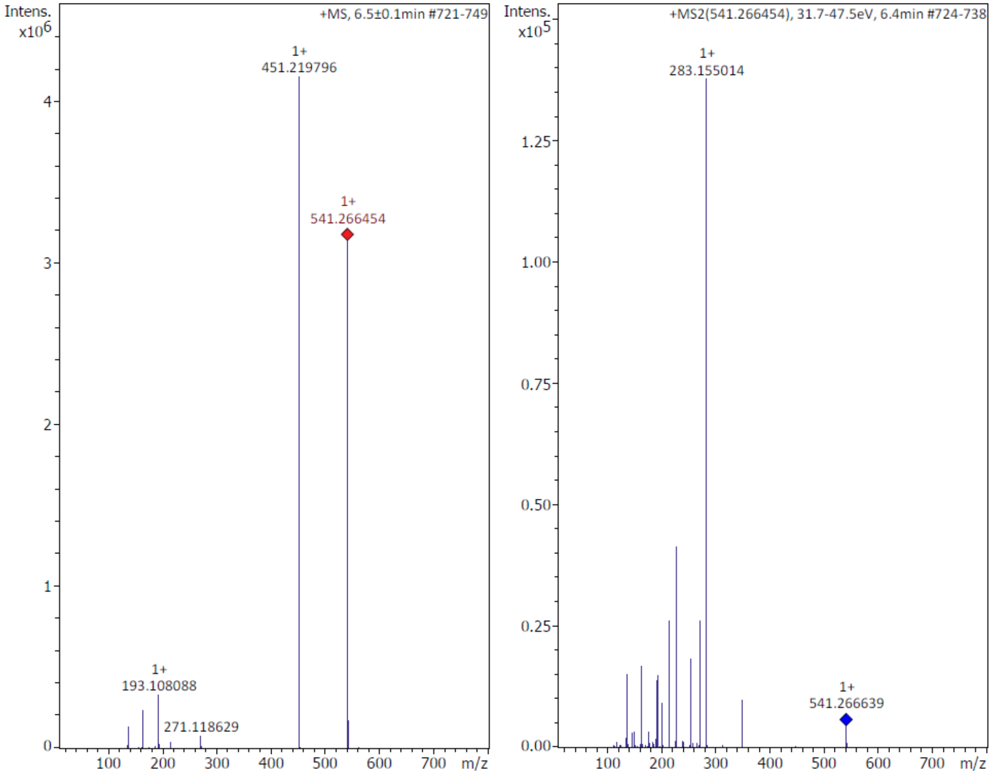

13d

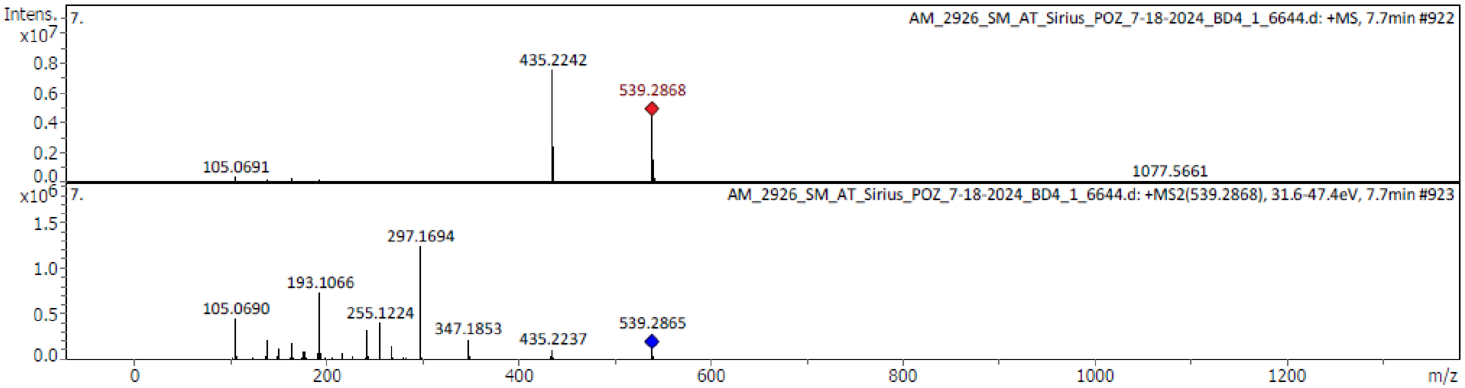

Intens.
x10^7
AM_2926_SM_AT_Sirius_POZ_7-18-2024_BD4_1_6644.d: +MS, 7.7min #922
435.2242
539.2868
105.0691
1077.5661
x10^6
AM_2926_SM_AT_Sirius_POZ_7-18-2024_BD4_1_6644.d: +MS2(539.2868), 31.6-47.4eV, 7.7min #923
297.1694
193.1066
105.0690
255.1224
347.1853
435.2237
539.2865
m/z
